# Impact of coronavirus disease 2019 on the number of newly diagnosed cancer patients and examinations and surgeries performed for cancer in Japan: a nationwide study

**DOI:** 10.1186/s12885-022-10417-6

**Published:** 2022-12-13

**Authors:** Takeshi Terashima, Hiroshi Konishi, Yasunori Sato, Muneki Igarashi, Takafumi Yanagibashi, Ryo Konno, Hideyuki Saya, Yuichiro Doki, Tadao Kakizoe

**Affiliations:** 1 Department of Respiratory Medicine, Tokyo Dental College, Ichikawa General Hospital, 5-11-13 Sugano, Ichikawa, Chiba, 272-0824 Japan; 2 Japan Cancer Society, Tokyo, Japan; 3 Department of Preventive Medicine and Public Health, Keio University School of Medicine, Tokyo, Japan; 4 Department of Gastroenterology, Tokai University School of Medicine, Tokyo Hospital, Tokyo, Japan; 5 Department of Gastroenterology, Kanagawa Prefectural Ashigarakami Hospital, Kanagawa, Japan; 6 Department of Obstetrics and Gynecology, Jichi Medical University, Saitama Medical Center, Saitama, Japan; 7 Division of Gene Regulation, Institute for Advanced Medical Research, Graduate School of Medicine, Keio University, Tokyo, Japan; 8 Department of Gastroenterological Surgery, Graduate School of Medicine, Osaka University, Osaka, Japan

**Keywords:** COVID-19, Cancer, Examination, Diagnosis, Surgery, Survey

## Abstract

**Background:**

The coronavirus disease 2019 (COVID-19) pandemic has rapidly and dramatically influenced healthcare across Japan. However, the influence of the COVID-19 pandemic on the number of newly diagnosed cancer, surgical treatment, and diagnostic examination for cancer types have not been completely investigated all over Japan. This study aimed to analyze the number of cases before and during the COVID-19 pandemic.

**Methods:**

This retrospective study was a survey that asked to provide the number of cases diagnosed with gastric, colorectal, lung, breast, and cervical cancer between January 2019 and December 2020. The survey was sent to tertiary healthcare hospitals, including national cancer institutions, university hospitals, and general hospitals, all over Japan. Data obtained from 105 of 486 surveyed hospitals were evaluated, and the number of cases in each quarter in 2020 was compared with that in the equivalent quarter in 2019.

**Results:**

In the second quarter (Q2), significant reductions were observed in the median number of newly diagnosed cases from 2019 to 2020: gastric cancer, 26.7% (43 vs. 32, *p* <  0.001); colorectal cancer, 17.9% (52 vs. 40, *p* <  0.001); lung cancer, 12.3% (53.5 vs. 47, *p* <  0.001); and breast cancer, 13.1% (43 vs. 35.5, *p* <  0.001). A significant reduction of 11.4% (9 vs. 8, *p* = 0.03) was observed in the third quarter (Q3) for cervical cancer. In Q2, the number of cases decreased by 30.9% (25 vs. 15, *p* <  0.001) for stage I gastric cancer, by 27.3% (12 vs. 9, *p* <  0.001) for stage I colorectal cancer, and by 17.6% (13 vs. 10, *p* <  0.001) for stage II breast cancer. The magnitude of reduction was significant for the localized stages of gastric, colorectal, and breast cancer according to diagnostic examinations in Q2 and surgical and endoscopic treatment in Q3 rather than that for lung or cervical cancer.

**Conclusions:**

COVID-19 has prolonged collateral effects on cancer care, including examination, diagnosis, and surgery, with significant effects on gastric cancer, followed by colorectal, lung, breast, and cervical cancer in Japan.

**Supplementary Information:**

The online version contains supplementary material available at 10.1186/s12885-022-10417-6.

## Background

The first case of coronavirus disease 2019 (COVID-19) was identified in China in December 2019, followed by a spread of the disease throughout Asian countries, leading to a worldwide pandemic. In Japan, the first case was confirmed in January 2020, and preventive measures, including hand washing, sanitizing public items, social distancing, and universal masking, have been implemented since February 2020. The number of confirmed cases increased in March, and the Japanese government proclaimed a state of emergency on April 7, 2020. The Japanese Health and Welfare Department announced that not all medical checks and screening examinations need to be performed during an emergency. The COVID-19 pandemic has rapidly and dramatically influenced healthcare across Japan; many hospital outbreaks resulted in poor outcomes, including death in hospitalized patients with comorbidities at a high risk of developing severe disease. The Japanese Society of Gastrointestinal Endoscopy issued guidelines to cease non-emergency endoscopic activity in March 2020. Consequently, visits, examinations, and procedures were restricted to prevent nosocomial transmission between patients and healthcare providers even after the state of emergency, which ended on May 25, 2020. Moreover, patients were hesitant to visit hospitals because of the risk of infection in the hospital or outside their homes [1].

The number of newly diagnosed cancer cases decreased during the pandemic in several countries. There was a 46.4% decrease in the number of cancer cases of six cancer types (breast, colorectal, lung, pancreatic, gastric, and esophageal) during the pandemic in the United States (US) [2]. Additionally, there was a 12% and 58% decrease in endoscopic activity and weekly diagnosed cancers, respectively, in the United Kingdom (UK) [3]. Although confirmed COVID-19 cases were fewer in Japan than in the US or the UK, the impact of the pandemic was not negligible, and the collateral effects could not be disregarded. The delay in diagnosis and treatment of cancer due to the COVID-19 pandemic could cause a poor prognosis for patients with cancer [4]. Therefore, this study aimed to analyze the number of newly diagnosed cancer cases and patients undergoing surgery, endoscopic procedures, and diagnostic examinations for cancer before and during the COVID-19 pandemic in Japan.

## Methods

### Study design and setting

This retrospective study was a survey among tertiary healthcare hospitals, including national cancer institutions, teaching hospitals affiliated with medical universities, and general hospitals across Japan. The survey on the monthly number of patients diagnosed with gastric, colorectal, lung, breast, and cervical cancer between January 2019 and December 2020 was conducted at the hospital level. Additionally, the questionnaire included the number of surgeries, endoscopic procedures, and diagnostic examinations and the time (days) from diagnosis to surgery. Database managers of the hospitals were responsible for filling out this questionnaire. The survey form was sent to 486 hospital representatives in June 2021, and responses were collected from July to August 2021.

The number of cases was assessed during 3-month periods as follows: first quarter (Q1: January–March), second quarter (Q2: April–June), third quarter (Q3: July–September), and fourth quarter (Q4: October–December) in 2019 and 2020. To account for seasonal variation, we assessed the percentage change in evaluated parameters for each quarter in 2020 compared with that for the equivalent quarter in 2019. The change in each quarter showed the following: Q1: the very early effect of the COVID-19 pandemic, Q2: the effect of the state of emergency and restriction of screening by the government, Q3: the delayed effect, and Q4: the effect that was not ameliorated in half a year after the end of the emergency.

The study did not include any individual patient-specific information other than aggregate data at the hospital level, and patient consent was waived owing to the retrospective nature of this study.

### Statistical analysis

The numbers of patients newly diagnosed with cancer and those undergoing surgery, endoscopic procedures, and diagnostic examinations in each quarter in 2020 were compared with that in the equivalent quarter in 2019 using the Wilcoxon signed-rank test. Significance was defined as a two-tailed *p* value of less than 0.05. The collected data were analyzed using JMP software, version 9.0.2, for Windows (Tokyo, Japan).

## Results

Among the 486 hospitals, replies to the questionnaire were obtained from 105 hospitals (21.6%); of which, 9.5% (*n* = 10) were from cancer specialized institutions; 25.7% (*n* = 27), from teaching hospitals associated with medical universities; and 64.8% (*n* = 68), from general hospitals. The distribution by area was 14.3% (*n* = 15) from Hokkaido and Tohoku, 23.8% (*n* = 25) from Kanto, which includes Tokyo (the most populated area in Japan), 18.1% (*n* = 19) from Chubu and Hokuriku, 15.1% (*n* = 16) from the Kinki, which includes Osaka (the second populated area), 11.4% (*n* = 12) from Shikoku and Chugoku, and 17.1% (*n* = 18) from Kyushu and Okinawa. The response rates in the areas ranged from 19.0 to 26.3%, and there was no difference.

**Fig. 1:**
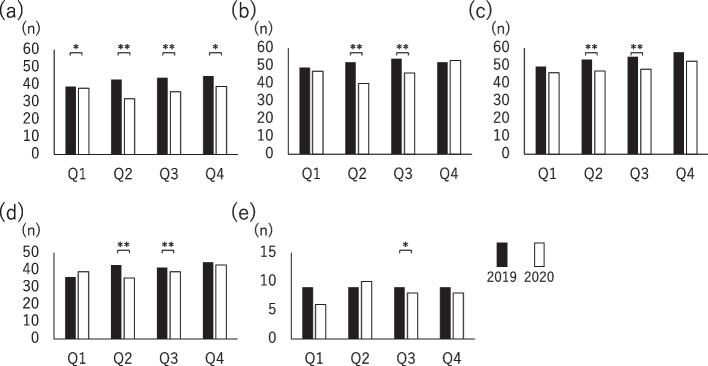
The number of cancer cases initially diagnosed in each quarter. The number of gastric (**a**), colorectal (**b**), lung (**c**), breast (**d**), and cervical (**e**) cancer in the first (Q1), second (Q2), third (Q3), and fourth (Q4) quarter in 2019 (black bar) and 2020 (white bar). Each bar shows the median. ** *p* <  0.001, * *p* <  0.05

The median (interquartile range [IQR]) of the number of newly diagnosed cancer cases during each quarter in 2019 and 2020 and the percentage change (% change: (number of 2020 – number of 2019)/number of 2019 × 100) are shown in Table 1. Significant reductions in the number of cancer cases, except cervical cancer cases, observed in 2019 and 2020 were in Q2 as follows: 26.7% (43 [25.5–62.5] vs. 32 [19–43.5], *p* <  0.001) in gastric cancer, 17.9% (52 [31–71] vs. 40 [25–58], *p* <  0.001) in colorectal cancer, 12.3% (53.5 [34.75–74.75] vs. 47 [32.25–67.25], *p* <  0.001) in lung cancer, and 13.1% (43 [25.25–68] vs. 35.5 [20.35–55.75], *p* <  0.001) in breast cancer (Fig. 1). Regarding cervical cancer, a significant reduction in the number of cases of 11.4% was observed only in Q3 (9 [4–19.5] vs. 8 [3–17.5], *p* = 0.03). The magnitude of reduction was significant in Q2, and a decrease was also observed in Q3: 13.5% (44 [28–63] vs. 36 [23.5–54.5], *p* <  0.001) in gastric cancer, 3.3% (54 [32–75] vs. 46 [28–66], *p* <  0.001) in colorectal cancer, 11.4% (55 [39.5–78.75] vs. 48 [36–67], *p* <  0.001) in lung cancer, and 11.0% (41.5 [27.25–73.5] vs. 39 [22.25–60.75], *p* <  0.001) in breast cancer. In Q1 and Q4, the reduction was significant only for gastric cancer; the magnitude of reduction was low as 4.0% (39 [25–56] vs. 38 [22.5–57], *p* = 0.03) in Q1 and 3.7% (45 [24–63] vs. 39 [23–60.5], *p* = 0.03) in Q4. The magnitude of reduction was the most significant in gastric cancer, followed by colorectal, lung, breast, and cervical cancer.

**Table 1:** Number of newly diagnosed cancer during each quarter in 2019 and 2020

			Q1 (Jan.-Mar.)	Q2 (Apr.-June)	Q3 (July-Sep.)	Q4 (Oct.-Dec.)
Gastric cancer	All stages	2019	39 (25-56)	43 (25.5-62.5)	44 (28-63)	45 (24-63)
		2020	38 (22.5-57)	32 (19-43.5)	36 (23.5-54.5)	39 (23-60.5)
		Change, %	−4.0 (−19.4-8.1)	−26.7 (− 41- -7.4)	−13.5 (− 28.5- -0.6)	−3.7 (− 22.2-9.8)
		*P* value	0.03	< 0.001	< 0.001	0.03
	Stage I	2019	22 (12-35)	25 (14-38)	26 (17-40)	26 (13-42)
		2020	21 (10-36)	15 (9-26)	20 (12-34)	22 (12-37)
		Change, %	−4.1 (−24.6-16.7)	− 30.9 (− 54- -10.8)	−18.2 (− 46.9-0.8)	−13.1 (− 25.2-11.1)
		*P* value	0.06	< 0.001	< 0.001	0.002
	Stage II	2019	3 (1-5)	3 (2-5)	3 (1-6)	3 (1-5)
		2020	3 (1-5)	2 (1-4)	3 (1-5)	3 (2-5)
		Change, %	0 (−40.1-67.9)	−33.3 (−66.7-50)	0 (−50-100)	0 (− 50-100)
		*P* value	0.84	0.003	0.30	0.35
	Stage III	2019	3 (2-4)	3 (1-6)	4 (2-6)	3 (1-5)
		2020	3 (1-5)	3 (2-5)	4 (2-5)	3 (1-6)
		Change, %	−33.3 (−66.7-100)	0 (−50-50)	−11.3 (−42.9-50)	0 (−42.5-100)
		*P* value	0.94	0.03	0.22	0.26
	Stage IV	2019	10 (1-10)	7 (4-11)	7 (4-10)	6 (3-10)
		2020	6 (1-10)	6 (3-9)	6 (4-10)	6 (4-9)
		Change, %	0 (−33.3-41.5)	7.4 (−47.9-28.6)	0 (−42.9-40)	0 (−33.3-40)
		*P* value	0.50	0.12	0.55	0.87
Colorectal cancer	All stages	2019	49 (30-72)	52 (31-71)	54 (32-75)	52 (34-71)
		2020	47 (30-70)	40 (25-58)	46 (28-66)	53 (30-70)
		Change, %	−3.4 (−16.7-12.9)	−17.9 (− 33.3- -7.4)	−3.3 (−24.1-2.9)	−1.8 (− 15-12.5)
		*P* value	0.06	< 0.001	< 0.001	0.31
	Stage I	2019	13 (8-18)	12 (7-18)	13 (7-20.5)	13 (8-18.5)
		2020	12 (6-17)	9 (5-13)	10 (7-16.5)	12 (8-19)
		Change, %	−13.3 (−30.8-16.7)	−27.3 (−50-0)	− 13.4 (−42.6-10.7)	−5.7 (− 31.2-30.8)
		*P* value	0.04	< 0.001	< 0.001	0.29
	Stage II	2019	7 (4-12.5)	9 (6-13)	8 (4.5-12.5)	9 (5-13)
		2020	7 (4-13)	6 (4-10.5)	8 (4-11)	8 (5-13)
		Change, %	−7.8 (−35.7-40)	−33.3 (−50-11.9)	−12.5 (−40.0-22.2)	−10.0 (− 33.3-50)
		*P* value	0.37	< 0.001	0.06	0.48
	Stage III	2019	9 (5-15)	9 (6-15)	11 (6-15.5)	10 (5-15.5)
		2020	10 (6-14)	8 (5-13)	9 (4-15.5)	10 (7-15.5)
		Change, %	−9.1 (−32.9-50)	−13.3 (−38.0-16.7)	−6.7 (−42.9-25)	10.0 (−25.0-40)
		*P* value	0.50	< 0.001	0.06	0.65
	Stage IV	2019	8 (5-11)	9 (5.5-12)	9 (5.5-14)	8 (5-12)
		2020	8 (5-11)	8 (5-11)	9 (5.5 − 12.5)	8 (4.5-12)
		Change, %	0 (−28.6-33.3)	-12.5 (− 33.3-25)	0 (−33.3-28.6)	−7.1 (− 32.4-30.3)
		*P* value	0.82	< 0.05	0.20	0.92
Lung cancer	All stages	2019	49.5 (33.25-72.25)	53.5 (34.75-74.75)	55 (39.5-78.75)	57.5 (36.5-74.75)
		2020	46 (35-68)	47 (32.25-67.25)	48 (36-67)	52.5 (35-75)
		Change, %	−2.3 (−16.2-14.0)	−12.3 (− 24.4-3.6)	−11.4 (− 23.0-8.4)	−1.4 (− 15.5-9.4)
		*P* value	0.12	< 0.001	< 0.001	0.11
	Stage I	2019	18 (10-28)	18 (11.75-27.25)	19.5 (11.75-28)	21.5 (12.75-32.25)
		2020	18 (11-27)	16 (9-23)	18.5 (10.75-25.5)	20 (12-32)
		Change, %	−4.7 (−25-45.5)	−8.8 (−28.6-12.5)	−6.3 (−30.8-24.7)	0 (− 20-23)
		*P* value	0.65	0.001	0.06	0.37
	Stage II	2019	4 (2-7)	3 (1-6.25)	4 (2-7)	4 (2-8)
		2020	3 (2-5)	3 (2-5.25)	4 (2-6)	4 (2-7)
		Change, %	−16.7 (−50.1-33.3)	0 (−50-100)	−13.3 (−50-40.7)	−12.5 (−50-45.8)
		*P* value	0.05	0.41	0.08	0.07
	Stage III	2019	8 (5-11)	8 (1.5-13)	8 (5-12)	8 (4-12)
		2020	7 (4-12)	7.5 (3.75-11)	7 (4-12)	8 (4-12.5)
		Change, %	−3.3 (−34.4-55.2)	−11.1 (−50-28.6)	−9.5 (−33.3-31.8)	0 (−27.1-49.3)
		*P* value	0.98	0.03	0.16	0.87
	Stage IV	2019	15.5 (10-22)	17 (10.75-25)	16 (11-24.5)	14 (9-23)
		2020	13 (9-22)	15 (9-22)	15 (9.75-21)	15 (9-22.25)
		Change, %	0 (−23.0-25.8)	−8.2 (−46.2-27.5)	−18.8 (−33.3-22.2)	0 (−23.1-35.8)
		*P* value	0.53	0.02	0.007	0.98
Breast cancer	All stages	2019	36 (22.5-64)	43 (25.25-68)	41.5 (27.25-73.5)	44.5 (26.5-70)
		2020	39 (22.5-66)	35.5 (20.35-55.75)	39 (22.25-60.75)	43 (23.25-74.25)
		Change, %	3.4 (−10.7-20)	−13.1 (−29.2-0)	− 11.0 (−23.7-5.8)	−4.1 (−19.7-20.4)
		*P* value	0.26	< 0.001	< 0.001	0.12
	Stage I	2019	15 (8.5-26.25)	17 (8-25.25)	18 (11-31)	19 (10-30.25)
		2020	17 (8.75-27)	14 (5.75-22.25)	15 (7-25)	17 (9-30)
		Change, %	11.8 (−21.8-37.1)	−13.7 (−42.8-11.3)	−16.6 (−37.2-3.5)	− 4.8 (−23.2-26.6)
		*P* value	0.28	< 0.001	< 0.001	0.02
	Stage II	2019	11 (5.75-17)	13 (6.75-31.5)	13 (7.75-20)	13 (7-18)
		2020	11 (6-17.5)	10 (5.75-16)	11 (6.75-18.25)	11.5 (5.75-21.25)
		Change, %	0 (−24.0-39.6)	−17.6 (−37.1-2.9)	−4.7 (−30.3-27.7)	0 (−29-38)
		*P* value	0.94	< 0.001	0.10	0.87
	Stage III	2019	3 (2-6)	3 (2-6.25)	4 (1-6)	3 (2-6.25)
		2020	3 (1-5)	3 (2-5)	3 (1-6)	6 (3-9)
		Change, %	0 (−40-50)	0 (−42.9-82.3)	0 (−42.9-100)	0 (− 42.9-50)
		*P* value	0.54	0.36	0.73	0.08
	Stage IV	2019	2 (1-3)	2 (1-4.25)	2 (1-4)	2 (1-4)
		2020	2 (1-5)	2 (1-4)	2.5 (1-4.25)	2 (1-4)
		Change, %	25 (−50-100)	0 (−66.7-73.3)	0 (−47.5-100)	33.3 (−66.7-100)
		*P* value	0.16	0.58	0.46	0.08
Cervical cancer	All stages	2019	9 (4-21.5)	9 (4-18.5)	9 (4-19.5)	9 (4-19)
		2020	6 (4-20.5)	10 (4-17.5)	8 (3-17.5)	8 (3.5-22)
		Change, %	0 (−36.7-35.4)	0 (−21.1-50)	−11.4 (−34.7-29.3)	0 (−31.3-31)
		*P* value	0.86	0.67	0.03	0.39
	Stage I	2019	2 (1-4)	2 (0-4)	2 (1-4)	3 (0.5-4)
		2020	2 (1-4)	2 (1-4)	2 (1-4)	2 (1-4)
		Change, %	0 (−50-50)	0 (−41.7-100)	0 (−40-66.7)	0 (−36.5-100)
		*P* value	0.35	0.29	0.58	0.71
	Stage II	2019	1 (0-2)	1 (0-2)	1 (0-2)	1 (0-2)
		2020	1 (0-2)	1 (0-2)	1 (0-2)	1 (0-2)
		Change, %	0 (−31.3-100)	0 (−100-100)	0 (−25-29.2)	0 (−66.7-100)
		*P* value	0.41	0.50	0.59	0.43
	Stage III	2019	1 (0-3)	1 (0-3)	1 (0-3)	1 (0-3)
		2020	1 (0-3)	1 (0-2)	1 (0-3)	1 (0-3)
		Change, %	0 (−16.3-100)	0 (−100-50)	0 (−36.7-100)	0 (−50-62.5)
		*P* value	0.66	0.68	0.81	0.51
	Stage IV	2019	1 (0-2)	1 (0-1)	1 (0-2)	1 (0-2)
		2020	0 (0-1.5)	1 (0-2)	1 (0-1.5)	1 (0-2)
		Change, %	0 (−100-100)	0 (−36.7-100)	0 (−50-0)	0 (−70.8-100)
		*P* value	0.47	0.74	0.62	0.36

Number showed the median (IQR)

Statistical analysis was performed by comparing the number of each hospital during each quarter in 2020 with the equivalent quarter in 2019 using Wilcoxon signed-rank test. Significance was defined as a *p* value of less than 0.05

The magnitude of reduction was significant in the hospitals in the populated area (Tokyo metropolitan and Osaka prefecture), where the number of confirmed cases of COVID-19 was high. Additionally, in Q2, a reduction was observed in the cases, of which 34.4% was in gastric, 38.3% in colorectal, 18.7% in lung, 35.4% in breast, and 14.4% in cervical cancer in the populated area, and 25.5% in gastric, 15.6% in colorectal, 11.6% in lung, 9.7 in breast and 0% in cervical cancer in the other areas. The difference in the reduction was significant for colorectal (*p* = 0.005) and breast (*p* = 0.002) cancer.

Substantial variation was observed across cancer types and stages of cancer. Regarding stage I gastric cancer, the number of cases decreased by 30.9% (25 [14–38] vs. 15 [9–26], *p* <  0.001). In Q3 and Q4, the reduction was significant only for stage I. In colorectal cancer, the number of cases decreased by 27.3% (12 [7–18] vs. 9 [5–13], *p* <  0.001) in stage I, by 33.3% (9 [6–13] vs. 6 [4–10.5], *p* <  0.001) in stage II, by 13.3% (9 [6–15] vs. 8 [5–13], *p* <  0.001) in stage III, and by 12.5% (9 [5.5–12] vs. 8 [5–11], *p* <  0.05) in stage IV in Q2. In Q3, the reduction was significant only in stage I. Regarding lung cancer, the number of cases decreased by 8.8% (18 [11.75–27.25] vs. 16 [9–23], *p* = 0.001) in stage I, by 11.1% (8 [1.5–13] vs. 7.5 [3.75–11], *p* = 0.03) in stage III, and by 8.2% (17 [10.75–25] vs. 15 [9–22], *p* = 0.02) in stage IV in Q2. In Q3, the reduction was significant only in stage IV. In breast cancer, the reduction was significant only in stage I by 13.7% (17 [8–25.25] vs. 14 [5.75–22.25], *p* <  0.001) and in stage II by 17.6% (13 [6.75–31.5] vs. 10 [5.75–16], *p* <  0.001) in Q2. In Q3 and Q4, the reduction was significant only in stage I. The magnitude of reduction was significant in the localized stages (stages I and II) for gastric, colorectal, and breast cancer but not for lung or cervical cancer. The magnitude of reduction was the most significant in Q2, followed by Q3, Q4, and Q1.

Table 2 shows the median (IQR) number of surgeries and endoscopic procedures during each quarter in 2019 and 2020 and the percentage change. The reduction in surgeries for gastric cancer was 25.5% (16 [8–25] vs. 12 [6.5–18], *p* <  0.001) in Q3, followed by 15.2% (16 [9–24] vs. 14 [8–20.5], *p* <  0.001) in Q4, and 14.3% (13 [8–21.5] vs. 12 [7–18.5], *p* = 0.003) in Q2. The number of gastric cancer cases treated using endoscopic procedures decreased by 19.7% (16 [8–27] vs. 13 [7–22.5], *p* <  0.001) in Q2 and 19.4% (16 [9.5–25.5] vs. 15 [7–23], *p* <  0.001) in Q3. Surgeries for colorectal cancer showed a reduction of 13.2% (31 [18–44.25] vs. 27.5 [19–39], *p* <  0.001) in Q2 and 15.3% (31.5 [20–43.25] vs. 28 [15–39.25], *p* <  0.001) in Q3. For the number of patients with colorectal cancer treated using endoscopic procedures, the reduction was the most significant in Q2 at 22.6% (22 [9–50] vs. 18 [6.75–41.75], *p* <  0.001), followed by Q3. For lung and breast cancers, the reduction appeared after a delay. The number of surgeries for lung cancer showed a significant reduction of 16.7% (27 [16.25–39] vs. 24 [14–35], *p* <  0.001) in Q3 and 6.4% (28 [18–40.75] vs. 26 [15–37.75], *p* = 0.008) in Q4. The number of surgeries for breast cancer showed a significant decrease of 9.7% (34 [18–54] vs. 32.5 [15.5–47.75], *p* <  0.001) in Q3 and 10.2% (36.5 [20.25–56.75] vs. 33.5 [19.5–53.5], *p* <  0.001) in Q4. There was no significant reduction in the number of surgeries for cervical cancer in any quarter in 2020. The reduction in the number of surgical and endoscopic treatments started in Q2, became largest in Q3, and continued over Q4 for most cancer types. The magnitude of reduction in the number of surgeries showed a similar pattern as that noted with the number of newly diagnosed cases.

**Table 2:** Number of surgery and endoscopic procedure during each quarter in 2019 and 2020

			Q1 (Jan.-Mar.)	Q2 (Apr.-June)	Q3 (July-Sep.)	Q4 (Oct.-Dec.)
Gastric cancer	Surgery	2019	14 (7-22.5)	13 (8-21.5)	16 (8-25)	16 (9-24)
		2020	14 (7.5-21)	12 (7-18.5)	12 (6.5-18)	14 (8-20.5)
		Change, %	−4.6 (−21.9-36.1)	−14.3 (−36.3-16.4)	−25.5 (− 44.5- -0.7)	−15.2 (− 31.0-11.4)
		*P* value	0.413	0.003	< 0.001	< 0.001
	Endoscopic procedure	2019	17 (7-26)	16 (8-27)	16 (9.5-25.5)	17 (8-28.5)
		2020	17 (10-28)	13 (7-22.5)	15 (7-23)	16 (7-26.5)
		Change, %	8.3 (−18.3-41.6)	−19.7 (−35.6-13.1)	− 19.4 (−40-7.9)	−8 (−29.6-19.7)
		*P* value	0.012	< 0.001	< 0.001	0.19
Colorectal cancer	Surgery	2019	30 (19-39.75)	31 (18-44.25)	31.5 (20-43.25)	32.5 (19-44)
		2020	31 (20.75-42)	27.5 (19-39)	28 (15-39.25)	29.5 (18-46.25)
		Change, %	3.8 (−17.2-30.6)	−13.2 (−26.1-11.1)	−15.3 (− 26.3-3.3)	−4.3 (− 18.4-14.4)
		*P* value	0.40	< 0.001	< 0.001	0.153
	Endoscopic procedure	2019	20.5 (9-56.5)	22 (9-50)	20.5 (8.75-57.75)	20.5 (9.75-61)
		2020	22 (10.5-57)	18 (6.75-41.75)	20 (7-51.25)	22 (7.75-58.5)
		Change, %	11.1 (−13.5-50.4)	−22.6 (−36.6-5.8)	−9.2 (−31.0-5.5)	− 1.5 (− 24.0-26.8)
		*P* value	0.02	< 0.001	0.001	0.61
Lung cancer	Surgery	2019	26 (14-36.75)	24(15.25-37)	27 (16.25-39)	28 (18-40.75)
		2020	24.5 (18.25-37.75)	22 (14.25-36)	24 (14-35)	26 (15-37.75)
		Change, %	4.7 (−16.7-35.0)	−2.0 (−23.5-20)	−16.7(−29.4-11.1)	−6.4 (−21.4-7.3)
		*P* value	0.26	0.21	< 0.001	0.008
Breast cancer	Surgery	2019	32 (15.25-49.75)	34.5 (18.25-51)	34 (18-54)	36.5 (20.25-56.75)
		2020	34.5 (17-58.75)	32 (14-48)	32.5 (15.5-47.75)	33.5 (19.5-53.5)
		Change, %	7.5 (−7.9-26.7)	−5.9 (−23.0-13)	−9.7 (−23.5-7.7)	−10.2 (−25.5-3.2)
		*P* value	< 0.001	0.08	< 0.001	< 0.001
Cervical cancer	Surgery	2019	10 (3-21)	10 (4.5-19)	10 (4-17.5)	10 (4-19.5)
		2020	9 (5-20)	11 (4-19)	10 (3.5-20)	10 (5-17.5)
		Change, %	0 (−21.9-60)	0 (−35.5-42.5)	0 (−38.8-28.5)	0 (−30.3-50)
		*P* value	0.87	0.26	0.25	0.87

Number showed the median (IQR)

Statistical analysis was performed by comparing the number of each hospital during each quarter in 2020 with the equivalent quarter in 2019 using Wilcoxon signed-rank test. Significance was defined as a *p* value of less than 0.05

Table 3 shows the median (IQR) number of diagnostic examinations during each quarter in 2019 and 2020 and the percentage change. A reduction in diagnostic examinations was observed for all cancer types, and in Q2, significant reductions were observed as follows: 27.3% (720 [269–1136] vs. 504 [174–754], *p* <  0.001) for gastric endoscopy, 27.5% (330 [124.5–562] vs. 251 [88–367], *p* <  0.001) for colorectal endoscopy, 24.6% (49.5 [28.5–77.75] vs. 39.5 [17.25–63], *p* <  0.001) for bronchoscopy, 13.0% (57 [23.35–86.25] vs. 46.5 [15.25–69.75], *p* <  0.001) for breast biopsy, and 6.3% (53 [16–146] vs. 47 [16–119], *p* <  0.001) for colposcopy for cervical biopsy. The reduction became largest in Q2 and continued over Q3 and Q4 for gastric endoscopy, bronchoscopy, and breast biopsy. The magnitude of reduction was significant for endoscopy (examinations for gastric, colorectal, and lung cancer).

**Table 3:** Number of diagnostic examination during each quarter in 2019 and 2020

	Q1 (Jan.-Mar.)	Q2 (Apr.-Jun.)	Q3 (Jul.-Sep.)	Q4 (Oct.-Dec.)
Endoscopy				
2019	675 (206-1075)	720 (269-1136)	735 (259-1204)	721 (237-1193)
2020	643 (212-1071)	504 (174-754)	668 (223-1095)	738 (228-1117)
Change, %	−1.0 (− 8.6-8.1)	−27.3 (− 39.4- -13.6)	− 8.5 (− 16.7- -1.8)	−3.7 (− 10.8-3.7)
*P* value	0.31	< 0.001	< 0.001	0.02
Colonoscopy				
2019	321 (106-537.5)	330 (124.5-562)	347 (132-573.5)	328 (119-563.5)
2020	313 (121-503.5)	251 (88-367)	310 (123.5-496.5)	331 (126-527.5)
Change, %	1.0 (−8.4-11.7)	−27.5 (−40.4- -11.3)	−12.1(−19.8- -3.1)	−1.2 (−9.0-8.4)
*P* value	0.69	< 0.001	< 0.001	0.10
Bronchoscopy				
2019	46 (24.75-76.5)	49.5 (28.5-77.75)	54 (29-90.25)	51.5 (25.5-88.5)
2020	45 (22.5-80.5)	39.5 (17.25-63)	42 (23-69.5)	50 (22.75-74.25)
Change, %	3.3 (−14.1-20.9)	−24.6 (−40.2- -5.5)	− 16.3 (−33.3-0)	−11.3 (− 33.7-10)
*P* value	0.25	< 0.001	< 0.001	< 0.001
Breast biopsy				
2019	49 (17-87.25)	57 (23.35-86.25)	57.5 (24.5-88.5)	61.5 (25.25-85)
2020	50.5 (19.25-87.75)	46.5 (15.25-69.75)	55 (21-85.5)	56.5 (18.25-95)
Change, %	−5.0 (−23.3-15.8)	−13.0 (−33.3-2.1)	−11.3 (−25.0-1.8)	−4.1 (−21.5-17.2)
*P* value	0.65	< 0.001	< 0.001	0.02
Colposcopy				
2019	54 (16-170)	53 (16-146)	61 (19-150)	60 (24-160)
2020	56 (19-147)	47 (16-119)	56 (14-144)	81 (19-166)
Change, %	−6.1 (−18.8-15.3)	−6.3 (−28.1-5.9)	− 1.0 (− 16.1-18.2)	0 (−23.3-16.3)
*P* value	0.07	< 0.001	0.31	0.90

Number showed the median (IQR)

Statistical analysis was performed by comparing the number of each hospital during each quarter in 2020 with the equivalent quarter in 2019 using Wilcoxon signed-rank test. Significance was defined as a *p* value of less than 0.05

Table 4 shows the mean (standard deviation [SD]) of the days from diagnosis to surgery, and there was no difference in this regard for any type of cancer between 2019 and 2020.

**Table 4:** The days from the diagnosis to surgery

		Mean + SD (days)
Gastric cancer	2019	48.3 ± 21.5
	2020	50.5 ± 24.3
	*P* value	0.628
Colorectal cancer	2019	40.8 ± 28.3
	2020	40.0 ± 21.7
	*P* value	0.729
Lung cancer	2019	42.4 ± 31.9
	2020	42.8 ± 28.5
	*P* value	0.999
Breast cancer	2019	63.8 ± 40.4
	2020	63.7 ± 35.8
	*P* value	0.962
Cervical cancer	2019	55.8 ± 30.9
	2020	53.9 + 27.2
	*P* value	0.433

Number showed the mean ± SD of the days from the diagnosis to surgery

## Discussion

This study showed that the number of patients newly diagnosed with cancer and those undergoing surgery, endoscopic procedures, and diagnostic examinations for gastric, colorectal, lung, breast, and cervical cancer markedly decreased during the COVID-19 pandemic in Japan. This reduction continued until the end of 2020. Rapid assessments and countermeasures for the effects of the COVID-19 pandemic on cancer care are important issues.

This study included gastric, colorectal, lung, breast, and cervical cancer because screening for these cancer types is recommended by the Japanese Health and Welfare Department and is believed may be influenced by the COVID-19 pandemic. To our knowledge, this is the first nationwide study surveying the number of newly diagnosed cancer cases and patients who underwent surgeries, endoscopic procedures, and examinations for these five cancer types, which accounts for half of the cases and deaths for all cancer types in Japan [5]. In the US, these five types of cancer accounted for 682,430 of 1,735,350 (39%) newly diagnosed cases and 261,050 of 609,640 (43%) deaths cause in 2018 [6]. We believe the number of cases for these five types of cancers offered enough information to evaluate the influence of the COVID-19 pandemic on all cancer types.

In 2020, the reductions were the largest for the diagnostic examination in Q2, followed by the newly diagnosed cases. The reduction in surgical and endoscopic treatments began in Q2, became largest in Q3, and continued over Q4 for most cancer types. Notably, the diagnostic examinations were thought to be directly influenced by the state of emergency and the screening restriction in Q2. The number of newly diagnosed cases was reduced because of the decreased number of examinations. Finally, the number of surgeries and endoscopic procedures was thought to decrease because of the reduction in the number of newly diagnosed cases. An alternative possibility was that the reduction in the number of surgeries was because of the decreased function of operating rooms in hospitals. An international study that included 61 countries reported that the time interval from diagnosis to surgery was associated with an increased likelihood of non-operation during the lockdown period [7]. However, this was unlikely because there was no difference in the days from diagnosis to surgery for any cancer type in the comparison between 2019 and 2020.

A reduction in the number of newly diagnosed cases was observed in all five cancer types surveyed. The reduction was the most significant for gastric cancer, followed by colorectal, lung, breast, and cervical cancer, as reflected by the reduction in the diagnostic examination, which was the most significant for gastric endoscopy, followed by colonoscopy, bronchoscopy, breast biopsy, and colposcopy. The standard screening tests are gastrointestinal endoscopy for gastric cancer, fecal occult blood test or colonoscopy for colorectal cancer, chest X-ray or computed tomography (CT) scan for lung cancer, mammography for breast cancer, and Papanicolaou test for cervical cancer [8–11]. In contrast to this study, a US report showed that the reduction of newly diagnosed cases from March to April 2020 was greatest for breast cancer, followed by colorectal, lung, and gastric cancer [2]. Moreover, in the US, there was a sharp decline in the number of screenings by 90.8% for breast cancer and 79.3% for colorectal cancer in April [12]. According to another report from the US, the number of colonoscopies decreased by 45% and chest CT scans by 10% [13]. In Japan, there was a decline in the number of cancer cases detected through screening by 60.3% for gastric, 42.1% for colorectal, 32.4% for lung, 42.9% for breast, and 38.7% for cervical cancer in May 2020 compared with the average cases between 2016 and 2019 [14]. Our study suggested that one of the possible reasons for the difference in the magnitude of reduction in diagnostic examination and newly diagnosed cases between the cancer types was the reduction in the magnitude of screening tests. Conversely, considering the cancer types with a significant reduction in other countries, another possible reason could be the variation in the incidence of cancer types between countries.

Moreover, the steps and diagnostic examination after the screening test are gastrointestinal endoscopy and biopsy for gastric cancer, colonoscopy and biopsy for colorectal cancer, CT scan, bronchoscopy or percutaneous biopsy for lung cancer, echogram and breast biopsy for breast cancer, and biopsy under colposcopy for cervical cancer [15]. Considering the risk of transmission during these and endoscopic examinations, the collateral effect on the diagnostic process was higher for gastric and colorectal cancer than for cervical, breast, and lung cancer [3].

The reduction was significant in the early stages of gastric, colorectal, and breast cancer but not in lung and cervical cancer. Based on the study in Japan, the proportion of asymptomatic cases in stage I was reported to be high, of which 59.4% for gastric, 55.5% for colorectal, 78.9% for lung, 39.8% for breast, and 48.6% for cervical cancer [16]. In Austria, there were more symptomatic cases of newly diagnosed breast cancer during the pandemic than pre-pandemic period [17]. In France, the breast cancer cases detected after the lockdown was more symptomatic and demonstrated bigger tumor sizes and higher rates of node invasion than those detected before the lockdown [18]. In asymptomatic patients with cancer in the early stages, a screening test or medical check is an important tool for diagnosis. Consequently, the reduction was significant in the early stages of gastric, colorectal, and breast cancers.

The reduction in examinations and the number of newly diagnosed cases were the largest in Q2 and continued until Q4 of 2020. The significant reduction in Q2 was thought to be induced by the state of emergency from April to May 2020. Although the magnitude of the reduction decreased in Q4, some cases that were supposed to be diagnosed without the pandemic were missed in 2020 and moved to the following years. Patients with undiagnosed cancer may need encouragement to undergo screening tests or diagnostic examinations with the assurance that these medical facilities have very little risk of transmission.

This study has some limitations. First, survey responses were obtained from 21.6% of the facilities. The percentage of the number of cases reported by these hospitals in 2019 per the number of cases registered in the national database in 2018 was 17.5% for all five cancer types combined, with 15.5% for gastric cancer, 14.4% for colorectal cancer, 18.7% for lung cancer, 20.7% for breast cancer, and 44.0% for cervical cancer, which suggested that the total number of cases in this study per annual registration cases in Japan was approximately 20%. Although the coverage rate was not high, it was thought that the results of this study reflected the real situation in Japan because the variation in the area and characteristics of the facilities was small. However, it is difficult to conclude that the results of this study have implications for other areas or countries in the world because there are differences in the severity of the pandemic and the medical systems used across countries. For instance, low-income countries experienced more pronounced variation in the fragility of cancer surgery and more collateral effects than high-income countries during the COVID-19 pandemic [7]. Second, factors other than a pandemic could induce a reduction. However, there were no natural disasters, sharp reductions in the population, or factors that decreased the prevalence of cancer during the study. Third, the number of cancer cases in 2019 could influence our conclusion. This possibility, however, was considered implausible because the report from the Japanese national clinical database showed that the number of patients who initiated treatment gradually increased from 2016 to 2019 and decreased in 2020 by 5.8% from 2019 and 1.9% compared with the average number between 2016 and 2019 [14]. Fourth, the extent to which the reduction observed in this study will influence prognosis is unclear. The time between preoperative diagnostic CT scan imaging and surgical treatment was reported to be associated with an increased risk of lung cancer recurrence [19]. Patients who underwent surgical treatment within 12 weeks of diagnosis had better overall survival than those who underwent procedures after more than 12 weeks [20]. Delayed diagnosis and surgery were believed to cause poor outcomes in many cancer types [21]. In Japan, there have been no clear reports showing the change in treatment patterns due to delayed diagnosis, except the report that indicated a greater decrease in radiotherapy compared with other treatments [22]. Further investigations and long-term follow-up are required to elucidate the effects of the findings of this study on patient outcomes. Furthermore, the surgical preparedness index, a tool used for assessing the ability of a certain hospital to maintain capacity during system stress, such as that imposed by the COVID-19 pandemic, should be taken into consideration to address the current backlogs, support delayed recovery from the pandemic, and prepare for future stress-imposing events [23].

## Conclusions

This study showed that the number of patients newly diagnosed with cancer and those who underwent surgery, endoscopic procedures, and diagnostic examinations for gastric, colorectal, lung, breast, and cervical cancer markedly decreased during the COVID-19 pandemic in Japan. Notably, this reduction continued until the end of 2020, and the rapid assessments and countermeasures for the effects of the COVID-19 pandemic on cancer care are important issues. Further investigation is needed to elucidate the recovery and prognostic effects in the future.

## Supplementary Information

**Additional file 1.**

## Data Availability

All data generated or analyzed during this study are included in this published article and its supplementary information files.

## Abbreviations

COVID-19: Coronavirus disease 2019
US: The United States
UK: The United Kingdom
Q1: First quarter (January–March)
Q2: Second quarter (April–June)
Q3: Third quarter (July–September)
Q4: Fourth quarter (October–December)
IQR: interquartile range
SD: standard deviation
CT: computed tomography
