# Supplementary material for: Impact of coronavirus disease 2019 on the number of newly diagnosed cancer patients and examinations and surgeries performed for cancer in Japan: a nationwide study
Source: BMC Cancer. 2022 Dec 13;22:1303. doi: 10.1186/s12885-022-10417-6 (PMC9745275; doi:10.1186/s12885-022-10417-6)
Supplement: Supplementary file 1 — Additional file 1. [file 12885_2022_10417_MOESM1_ESM.docx]

Appendix

Table 1. Number of newly diagnosed gastric cancer during each month in 2019.

Table 2. Number of newly diagnosed gastric cancer during each month in 2020.

Table 3. Number of newly diagnosed stage I gastric cancer during each month in 2019.

Table 4. Number of newly diagnosed stage I gastric cancer during each month in 2020.

Table 5. Number of newly diagnosed stage II gastric cancer during each month in 2019.

Table 6. Number of newly diagnosed stage II gastric cancer during each month in 2020.

Table 7. Number of newly diagnosed stage III gastric cancer during each month in 2019.

Table 8. Number of newly diagnosed stage III gastric cancer during each month in 2020.

Table 9. Number of newly diagnosed stage IV gastric cancer during each month in 2019.

Table 10. Number of newly diagnosed stage IV gastric cancer during each month in 2020.

Table 11. Number of newly diagnosed colorectal cancer during each month in 2019.

Table 12. Number of newly diagnosed colorectal cancer during each month in 2020.

Table 13. Number of newly diagnosed stage I colorectal cancer during each month in 2019.

Table 14. Number of newly diagnosed stage I colorectal cancer during each month in 2020.

Table 15. Number of newly diagnosed stage II colorectal cancer during each month in 2019.

Table 16. Number of newly diagnosed stage II colorectal cancer during each month in 2020.

Table 17. Number of newly diagnosed stage III colorectal cancer during each month in 2019.

Table 18. Number of newly diagnosed stage III colorectal cancer during each month in 2020.

Table 19. Number of newly diagnosed stage IV colorectal cancer during each month in 2019.

Table 20. Number of newly diagnosed stage IV colorectal cancer during each month in 2020.

Table 21. Number of newly diagnosed lung cancer during each month in 2019.

Table 22. Number of newly diagnosed lung cancer during each month in 2020.

Table 23. Number of newly diagnosed stage I lung cancer during each month in 2019.

Table 24. Number of newly diagnosed stage I lung cancer during each month in 2020.

Table 25. Number of newly diagnosed stage II lung cancer during each month in 2019.

Table 26. Number of newly diagnosed stage II lung cancer during each month in 2020.

Table 27. Number of newly diagnosed stage III lung cancer during each month in 2019.

Table 28. Number of newly diagnosed stage III lung cancer during each month in 2020.

Table 29. Number of newly diagnosed stage IV lung cancer during each month in 2019.

Table 30. Number of newly diagnosed stage IV lung cancer during each month in 2020.

Table 31. Number of newly diagnosed breast cancer during each month in 2019.

Table 32. Number of newly diagnosed breast cancer during each month in 2020.

Table 33. Number of newly diagnosed stage I breast cancer during each month in 2019.

Table 34. Number of newly diagnosed stage I breast cancer during each month in 2020.

Table 35. Number of newly diagnosed stage II breast cancer during each month in 2019.

Table 36. Number of newly diagnosed stage II breast cancer during each month in 2020.

Table 37. Number of newly diagnosed stage III breast cancer during each month in 2019.

Table 38. Number of newly diagnosed stage III breast cancer during each month in 2020.

Table 39. Number of newly diagnosed stage IV breast cancer during each month in 2019.

Table 40. Number of newly diagnosed stage IV breast cancer during each month in 2020.

Table 41. Number of newly diagnosed cervical cancer breast during each month in 2019.

Table 42. Number of newly diagnosed cervical cancer cancer during each month in 2020.

Table 43. Number of newly diagnosed stage I cervical cancer cancer during each month in 2019.

Table 44. Number of newly diagnosed stage I cervical cancer cancer during each month in 2020.

Table 45. Number of newly diagnosed stage II cervical cancer cancer during each month in 2019.

Table 46. Number of newly diagnosed stage II cervical cancer cancer during each month in 2020.

Table 47. Number of newly diagnosed stage III cervical cancer cancer during each month in 2019.

Table 48. Number of newly diagnosed stage III cervical cancer cancer during each month in 2020.

Table 49. Number of newly diagnosed stage IV cervical cancer cancer during each month in 2019.

Table 50. Number of newly diagnosed stage IV cervical cancer cancer during each month in 2020.

Table 51. Number of gastric cancer surgery during each month in 2019.

Table 52. Number of gastric cancer surgery during each month in 2020.

Table 53. Number of gastric cancer endoscopic procedure during each month in 2019.

Table 54. Number of gastric cancer endoscopic procedure during each month in 2020.

Table 55. Number of colorectal cancer surgery during each month in 2019.

Table 56. Number of colorectal cancer surgery during each month in 2020.

Table 57. Number of colorectal cancer endoscopic procedure during each month in 2019.

Table 58. Number of colorectal cancer endoscopic procedure during each month in 2020.

Table 59. Number of lung cancer surgery during each month in 2019.

Table 60. Number of lung cancer surgery during each month in 2020.

Table 61. Number of breast cancer surgery during each month in 2019.

Table 62. Number of breast cancer surgery during each month in 2020.

Table 63. Number of cervical cancer surgery during each month in 2019.

Table 64. Number of cervical cancer surgery during each month in 2020.

Table 65. Number of endoscopy during each month in 2019.

Table 66. Number of endoscopy during each month in 2020.

Table 67. Number of colonoscopy during each month in 2019.

Table 68. Number of colonoscopy during each month in 2020.

Table 69. Number of bronchoscopy during each month in 2019.

Table 70. Number of bronchoscopy during each month in 2020.

Table 71. Number of breast biopsy during each month in 2019.

Table 72. Number of breast biopsy during each month in 2020.

Table 73. Number of colposcopy during each month in 2019.

Table 74. Number of colposcopy during each month in 2020.

Table 1. Number of newly diagnosed gastric cancer during each month in 2019.

|  | Month | | | | | | | | | | | |
| --- | --- | --- | --- | --- | --- | --- | --- | --- | --- | --- | --- | --- |
| Hospital | 1 | 2 | 3 | 4 | 5 | 6 | 7 | 8 | 9 | 10 | 11 | 12 |
| 1 | 1 | 3 | 3 | 4 | 5 | 4 | 4 | 5 | 8 | 2 | 3 | 6 |
| 2 | 92 | 82 | 101 | 94 | 78 | 83 | 109 | 86 | 104 | 106 | 89 | 95 |
| 3 | 6 | 5 | 5 | 10 | 10 | 7 | 9 | 9 | 5 | 1 | 6 | 5 |
| 4 | 15 | 12 | 19 | 26 | 14 | 18 | 19 | 17 | 8 | 23 | 23 | 21 |
| 5 | 5 | 10 | 9 | 5 | 6 | 5 | 11 | 13 | 10 | 5 | 9 | 13 |
| 6 | 6 | 12 | 12 | 12 | 9 | 19 | 13 | 13 | 13 | 25 | 12 | 9 |
| 7 | 10 | 14 | 12 | 20 | 13 | 21 | 26 | 15 | 12 | 13 | 16 | 18 |
| 8 | 3 | 2 | 0 | 0 | 3 | 2 | 3 | 2 | 1 | 1 | 3 | 1 |
| 9 | 9 | 14 | 11 | 13 | 11 | 9 | 11 | 18 | 9 | 9 | 13 | 12 |
| 10 | 11 | 11 | 10 | 9 | 9 | 15 | 17 | 9 | 8 | 19 | 15 | 16 |
| 11 | 16 | 22 | 23 | 30 | 20 | 24 | 30 | 18 | 21 | 18 | 21 | 20 |
| 12 | 5 | 2 | 2 | 2 | 4 | 6 | 5 | 9 | 3 | 5 | 6 | 3 |
| 13 | 17 | 22 | 10 | 26 | 19 | 17 | 28 | 24 | 22 | 38 | 19 | 13 |
| 14 | 9 | 9 | 7 | 19 | 11 | 16 | 22 | 13 | 12 | 23 | 16 | 16 |
| 15 | 31 | 19 | 33 | 36 | 22 | 26 | 34 | 32 | 33 | 28 | 38 | 21 |
| 16 | 37 | 26 | 42 | 48 | 22 | 35 | 45 | 26 | 23 | 36 | 32 | 32 |
| 17 |  | 1 |  |  |  | 2 |  |  |  | 1 |  |  |
| 18 | 3 | 9 | 7 | 7 | 7 | 3 | 4 | 8 | 12 | 13 | 3 | 13 |
| 19 | 6 | 7 | 8 | 8 | 13 | 10 | 14 | 6 | 11 | 6 | 11 | 6 |
| 20 | 60 | 53 | 44 | 47 | 52 | 54 | 62 | 56 | 56 | 55 | 62 | 49 |
| 21 | 15 | 13 | 13 | 17 | 16 | 9 | 19 | 16 | 10 | 20 | 7 | 13 |
| 22 | 5 | 4 | 5 | 5 | 8 | 12 | 10 | 6 | 12 | 4 | 8 | 7 |
| 23 | 50 | 45 | 67 | 60 | 71 | 66 | 68 | 51 | 69 | 43 | 50 | 60 |
| 24 | 14 | 24 | 16 | 23 | 26 | 20 | 20 | 18 | 16 | 16 | 20 | 18 |
| 25 | 16 | 20 | 33 | 28 | 18 | 26 | 21 | 29 | 14 | 22 | 22 | 27 |
| 26 | 19 | 15 | 5 | 17 | 14 | 17 | 24 | 11 | 14 | 15 | 10 | 17 |
| 27 | 3 | 6 | 4 | 9 | 4 | 2 | 9 | 2 | 5 | 7 | 5 | 3 |
| 28 | 11 | 10 | 13 | 15 | 12 | 9 | 13 | 18 | 14 | 23 | 12 | 15 |
| 29 | 44 | 27 | 38 | 28 | 28 | 35 | 43 | 28 | 14 | 27 | 23 | 25 |
| 30 | 6 | 10 | 12 | 8 | 5 | 15 | 15 | 13 | 7 | 15 | 9 | 13 |
| 31 | 13 | 11 | 14 | 16 | 10 | 10 | 9 | 11 | 8 | 18 | 18 | 10 |
| 32 | 6 | 10 | 7 | 6 | 6 | 8 | 11 | 11 | 11 | 5 | 5 | 6 |
| 33 | 7 | 8 | 13 | 6 | 8 | 11 | 11 | 9 | 10 | 13 | 7 | 9 |
| 34 |  |  |  |  |  |  |  |  |  |  |  |  |
| 35 | 14 | 14 | 26 | 13 | 20 | 25 | 18 | 22 | 23 | 19 | 16 | 15 |
| 36 | 12 | 10 | 10 | 11 | 14 | 7 | 10 | 14 | 13 | 10 | 12 | 9 |
| 37 | 24 | 35 | 28 | 28 | 34 | 33 | 41 | 39 | 40 | 35 | 35 | 40 |
| 38 | 27 | 26 | 31 | 25 | 23 | 27 | 23 | 13 | 23 | 22 | 20 | 22 |
| 39 |  |  |  |  |  |  |  |  |  |  |  |  |
| 40 | 6 | 11 | 9 | 10 | 10 | 7 | 15 | 12 | 16 | 13 | 7 | 14 |
| 41 | 25 | 16 | 15 | 26 | 19 | 13 | 21 | 22 | 20 | 21 | 21 | 13 |
| 42 | 13 | 10 | 11 | 11 | 15 | 10 | 12 | 13 | 17 | 8 | 6 | 7 |
| 43 | 15 | 18 | 17 | 14 | 10 | 13 | 14 | 18 | 13 | 16 | 18 | 11 |
| 44 | 7 | 3 | 1 | 3 | 4 | 4 | 4 | 6 | 0 | 3 | 0 | 3 |
| 45 | 12 | 12 | 16 | 14 | 11 | 11 | 13 | 7 | 12 | 14 | 20 | 5 |
| 46 | 17 | 13 | 18 | 19 | 20 | 14 | 20 | 15 | 24 | 22 | 19 | 25 |
| 47 | 6 | 6 | 6 | 8 | 8 | 1 | 8 | 3 | 3 | 7 | 3 | 5 |
| 48 | 15 | 14 | 18 | 19 | 18 | 12 | 22 | 15 | 15 | 22 | 12 | 22 |
| 49 | 7 | 16 | 13 | 8 | 15 | 5 | 16 | 12 | 8 | 10 | 12 | 14 |
| 50 | 3 | 4 | 4 | 8 | 3 | 5 | 3 | 0 | 3 | 7 | 3 | 2 |
| 51 | 17 | 16 | 23 | 22 | 28 | 21 | 34 | 28 | 31 | 36 | 31 | 29 |
| 52 | 18 | 12 | 22 | 28 | 15 | 24 | 27 | 23 | 20 | 25 | 20 | 17 |
| 53 | 32 | 14 | 19 | 22 | 21 | 20 | 30 | 17 | 18 | 23 | 17 | 25 |
| 54 | 61 | 58 | 63 | 54 | 61 | 68 | 87 | 65 | 75 | 75 | 75 | 68 |
| 55 | 9 | 10 | 9 | 13 | 7 | 11 | 9 | 5 | 12 | 9 | 13 | 11 |
| 56 | 26 | 33 | 26 | 24 | 26 | 19 | 26 | 28 | 30 | 7 | 16 | 22 |
| 57 | 40 | 39 | 35 | 30 | 34 | 36 | 42 | 31 | 21 | 19 | 26 | 26 |
| 58 | 8 | 4 | 11 | 11 | 9 | 6 | 15 | 12 | 16 | 19 | 10 | 21 |
| 59 | 8 | 14 | 6 | 5 | 10 | 8 | 13 | 8 | 8 | 9 | 6 | 4 |
| 60 | 15 | 12 | 10 | 17 | 15 | 13 | 6 | 18 | 6 | 6 | 16 | 13 |
| 61 | 15 | 9 | 10 | 18 | 16 | 9 | 18 | 12 | 15 | 20 | 10 | 15 |
| 62 | 20 | 12 | 20 | 14 | 18 | 17 | 19 | 14 | 23 | 15 | 25 | 13 |
| 63 | 10 | 8 | 7 | 8 | 9 | 11 | 3 | 18 | 6 | 9 | 7 | 9 |
| 64 |  |  |  |  |  |  |  |  |  |  |  |  |
| 65 | 23 | 29 | 19 | 17 | 28 | 23 | 37 | 39 | 17 | 21 | 29 | 23 |
| 66 | 1 | 0 | 2 | 2 | 1 | 4 | 4 | 6 | 3 | 1 | 1 | 1 |
| 67 | 13 | 7 | 8 | 8 | 12 | 9 | 12 | 13 | 9 | 11 | 11 | 6 |
| 68 | 26 | 26 | 30 | 15 | 28 | 21 | 25 | 25 | 24 | 22 | 29 | 25 |
| 69 | 25 | 24 | 27 | 28 | 20 | 19 | 29 | 29 | 25 | 22 | 23 | 34 |
| 70 | 0 | 2 | 3 | 6 | 3 | 2 | 5 | 7 | 2 | 4 | 4 | 6 |
| 71 | 18 | 12 | 20 | 15 | 16 | 19 | 23 | 14 | 14 | 17 | 13 | 19 |
| 72 | 19 | 29 | 24 | 17 | 23 | 21 | 20 | 22 | 19 | 20 | 24 | 24 |
| 73 | 13 | 19 | 14 | 13 | 13 | 11 | 13 | 12 | 5 | 11 | 12 | 12 |
| 74 | 24 | 25 | 23 | 19 | 22 | 31 | 19 | 19 | 24 | 24 | 22 | 18 |
| 75 | 5 | 5 | 1 | 2 | 1 | 1 | 2 | 1 | 2 | 3 | 1 | 1 |
| 76 | 17 | 13 | 14 | 12 | 16 | 18 | 13 | 14 | 30 | 16 | 16 | 11 |
| 77 | 3 | 1 | 2 | 4 | 4 | 2 | 0 | 2 | 2 | 8 | 3 | 6 |
| 78 |  |  |  |  |  |  |  |  |  |  |  |  |
| 79 | 27 | 21 | 22 | 24 | 14 | 24 | 23 | 29 | 28 | 28 | 25 | 22 |
| 80 | 16 | 20 | 10 | 23 | 25 | 20 | 21 | 18 | 28 | 15 | 16 | 16 |
| 81 | 12 | 8 | 30 | 16 | 16 | 15 | 21 | 14 | 13 | 24 | 17 | 21 |
| 82 |  |  |  |  |  |  |  |  |  |  |  |  |
| 83 |  |  |  |  |  |  |  |  |  |  |  |  |
| 84 | 21 | 14 | 22 | 18 | 20 | 16 | 25 | 13 | 12 | 19 | 15 | 27 |
| 85 | 14 | 10 | 10 | 17 | 15 | 14 | 17 | 9 | 12 | 15 | 9 | 13 |
| 86 | 13 | 16 | 13 | 12 | 21 | 18 | 9 | 19 | 16 | 22 | 19 | 14 |
| 87 | 7 | 5 | 6 | 8 | 5 | 9 | 11 | 6 | 7 | 8 | 1 | 6 |
| 88 | 11 | 8 | 9 | 8 | 14 | 12 | 13 | 11 | 9 | 11 | 13 | 11 |
| 89 | 52 | 47 | 48 | 36 | 46 | 59 | 54 | 54 | 40 | 50 | 45 | 54 |
| 90 | 11 | 17 | 9 | 20 | 12 | 15 | 14 | 17 | 13 | 15 | 11 | 13 |
| 91 | 22 | 8 | 10 | 15 | 17 | 29 | 11 | 20 | 7 | 14 | 11 | 13 |
| 92 |  |  |  |  |  |  |  |  |  |  |  |  |
| 93 |  |  |  |  |  |  |  |  |  |  |  |  |
| 94 | 5 | 6 | 6 | 9 | 10 | 8 | 6 | 9 | 12 | 6 | 5 | 7 |
| 95 | 6 | 9 | 3 | 8 | 1 | 2 | 8 | 5 | 4 | 2 | 1 | 4 |
| 96 | 9 | 15 | 16 | 4 | 8 | 12 | 9 | 10 | 9 | 8 | 6 | 8 |
| 97 | 2 | 3 | 4 | 3 | 2 | 4 | 3 | 6 | 1 | 3 | 0 | 1 |
| 98 | 17 | 13 | 15 | 8 | 6 | 5 | 11 | 9 | 8 | 7 | 10 | 9 |
| 99 | 5 | 10 | 15 | 8 | 6 | 3 | 7 | 5 | 3 | 4 | 9 | 2 |
| 100 | 19 | 16 | 18 | 19 | 11 | 17 | 17 | 14 | 23 | 22 | 13 | 11 |
| 101 | 18 | 11 | 10 | 12 | 11 | 11 | 14 | 26 | 16 | 12 | 17 | 16 |
| 102 | 9 | 9 | 24 | 11 | 12 | 20 | 22 | 17 | 22 | 10 | 23 | 14 |
| 103 | 9 | 8 | 8 | 16 | 19 | 16 | 21 | 13 | 12 | 16 | 17 | 26 |
| 104 | 24 | 20 | 19 | 21 | 26 | 19 | 24 | 25 | 27 | 37 | 25 | 26 |
| 105 | 9 | 10 | 13 | 11 | 16 | 17 | 19 | 9 | 9 | 15 | 13 | 14 |

Table 2. Number of newly diagnosed gastric cancer during each month in 2020.

|  | Month | | | | | | | | | | | |
| --- | --- | --- | --- | --- | --- | --- | --- | --- | --- | --- | --- | --- |
| Hospital | 1 | 2 | 3 | 4 | 5 | 6 | 7 | 8 | 9 | 10 | 11 | 12 |
| 1 | 5 | 2 | 6 | 3 | 3 | 4 | 4 | 6 | 4 | 5 | 4 | 1 |
| 2 | 83 | 91 | 79 | 51 | 32 | 51 | 79 | 66 | 76 | 80 | 70 | 75 |
| 3 | 7 | 5 | 5 | 8 | 2 | 6 | 4 | 12 | 12 | 12 | 8 | 8 |
| 4 | 20 | 12 | 16 | 13 | 11 | 10 | 7 | 8 | 11 | 20 | 12 | 14 |
| 5 | 11 | 5 | 13 | 7 | 6 | 5 | 8 | 7 | 7 | 15 | 13 | 7 |
| 6 | 9 | 6 | 11 | 12 | 13 | 18 | 7 | 3 | 19 | 15 | 9 | 14 |
| 7 | 17 | 18 | 13 | 13 | 10 | 11 | 13 | 17 | 19 | 12 | 16 | 16 |
| 8 | 1 | 3 | 4 | 7 | 9 | 3 | 4 | 5 | 7 | 6 | 5 | 10 |
| 9 | 11 | 9 | 9 | 9 | 4 | 12 | 15 | 8 | 10 | 7 | 9 | 7 |
| 10 | 7 | 11 | 9 | 10 | 9 | 9 | 7 | 6 | 11 | 13 | 6 | 10 |
| 11 | 14 | 17 | 12 | 14 | 11 | 15 | 16 | 15 | 24 | 20 | 19 | 21 |
| 12 | 9 | 6 | 5 | 2 | 10 | 7 | 6 | 2 | 3 | 6 | 3 | 5 |
| 13 | 23 | 19 | 20 | 14 | 12 | 16 | 20 | 17 | 24 | 19 | 17 | 25 |
| 14 | 16 | 8 | 9 | 5 | 13 | 16 | 9 | 18 | 17 | 16 | 12 | 11 |
| 15 | 29 | 30 | 31 | 25 | 7 | 26 | 28 | 21 | 29 | 26 | 29 | 34 |
| 16 | 26 | 40 | 35 | 40 | 22 | 19 | 40 | 29 | 37 | 37 | 43 | 39 |
| 17 |  |  |  |  |  | 2 |  |  |  |  | 1 |  |
| 18 | 3 | 2 | 7 | 6 | 5 | 8 | 8 | 11 | 4 | 6 | 10 | 9 |
| 19 | 8 | 6 | 7 | 9 | 6 | 7 | 6 | 5 | 11 | 5 | 7 | 7 |
| 20 | 54 | 51 | 51 | 17 | 18 | 36 | 37 | 35 | 43 | 39 | 48 | 43 |
| 21 | 5 | 17 | 16 | 7 | 8 | 14 | 16 | 15 | 10 | 8 | 11 | 9 |
| 22 | 6 | 9 | 2 | 5 | 3 | 11 | 7 | 9 | 3 | 13 | 14 | 5 |
| 23 | 44 | 56 | 68 | 33 | 34 | 45 | 39 | 46 | 48 | 55 | 44 | 61 |
| 24 | 16 | 13 | 23 | 14 | 13 | 20 | 16 | 14 | 17 | 21 | 15 | 16 |
| 25 | 17 | 19 | 28 | 15 | 12 | 23 | 18 | 18 | 25 | 25 | 19 | 22 |
| 26 | 9 | 13 | 10 | 8 | 10 | 7 | 14 | 16 | 3 | 11 | 13 | 17 |
| 27 | 7 | 1 | 6 | 2 | 4 | 5 | 3 | 2 | 1 | 0 | 6 | 5 |
| 28 | 10 | 5 | 14 | 6 | 4 | 7 | 15 | 8 | 17 | 10 | 11 | 8 |
| 29 | 24 | 25 | 35 | 26 | 18 | 18 | 30 | 23 | 37 | 31 | 41 | 30 |
| 30 | 9 | 9 | 11 | 10 | 4 | 8 | 10 | 13 | 9 | 11 | 15 | 16 |
| 31 | 7 | 12 | 6 | 8 | 5 | 5 | 8 | 9 | 13 | 13 | 14 | 10 |
| 32 | 4 | 4 | 3 | 3 | 5 | 3 | 7 | 5 | 6 | 9 | 6 | 7 |
| 33 | 14 | 7 | 12 | 13 | 12 | 10 | 15 | 4 | 12 | 18 | 5 | 10 |
| 34 |  |  |  |  |  |  |  |  |  |  |  |  |
| 35 | 21 | 19 | 14 | 15 | 17 | 15 | 19 | 21 | 14 | 19 | 9 | 12 |
| 36 | 11 | 11 | 13 | 17 | 13 | 14 | 12 | 10 | 19 | 15 | 13 | 14 |
| 37 | 35 | 24 | 31 | 20 | 21 | 33 | 32 | 30 | 28 | 33 | 37 | 35 |
| 38 | 17 | 14 | 11 | 10 | 12 | 20 | 21 | 10 | 17 | 21 | 18 | 23 |
| 39 |  |  |  |  |  |  |  |  |  |  |  |  |
| 40 | 7 | 4 | 8 | 7 | 10 | 6 | 17 | 8 | 14 | 19 | 6 | 10 |
| 41 | 21 | 21 | 23 | 16 | 15 | 8 | 17 | 20 | 14 | 14 | 8 | 12 |
| 42 | 11 | 14 | 8 | 6 | 10 | 3 | 6 | 13 | 8 | 7 | 7 | 9 |
| 43 | 14 | 13 | 11 | 12 | 15 | 12 | 23 | 12 | 18 | 13 | 10 | 6 |
| 44 | 2 | 1 | 4 | 2 | 3 | 2 | 1 | 3 | 6 | 2 | 2 | 7 |
| 45 | 16 | 14 | 9 | 10 | 6 | 8 | 14 | 7 | 13 | 17 | 9 | 13 |
| 46 | 22 | 22 | 17 | 21 | 20 | 18 | 23 | 16 | 21 | 27 | 23 | 19 |
| 47 | 3 | 0 | 2 | 5 | 2 | 4 | 3 | 4 | 4 | 0 | 2 | 10 |
| 48 | 16 | 17 | 11 | 10 | 11 | 16 | 15 | 13 | 10 | 14 | 10 | 12 |
| 49 | 13 | 5 | 7 | 11 | 7 | 14 | 3 | 7 | 13 | 6 | 6 | 8 |
| 50 | 1 | 3 | 3 | 0 | 1 | 3 | 4 | 2 | 2 | 2 | 3 | 3 |
| 51 | 19 | 29 | 26 | 33 | 16 | 28 | 32 | 34 | 20 | 25 | 29 | 28 |
| 52 | 17 | 14 | 25 | 16 | 9 | 11 | 15 | 19 | 19 | 17 | 20 | 9 |
| 53 | 17 | 20 | 18 | 22 | 9 | 17 | 20 | 18 | 25 | 30 | 17 | 18 |
| 54 | 71 | 69 | 71 | 47 | 52 | 55 | 57 | 53 | 58 | 82 | 69 | 63 |
| 55 | 10 | 10 | 10 | 8 | 6 | 4 | 7 | 11 | 9 | 4 | 7 | 4 |
| 56 | 22 | 18 | 25 | 16 | 9 | 16 | 21 | 24 | 21 | 24 | 21 | 20 |
| 57 | 34 | 22 | 36 | 30 | 21 | 46 | 32 | 25 | 26 | 43 | 25 | 35 |
| 58 | 18 | 14 | 17 | 11 | 9 | 9 | 9 | 10 | 10 | 15 | 16 | 22 |
| 59 | 5 | 9 | 9 | 5 | 9 | 9 | 9 | 9 | 7 | 8 | 8 | 4 |
| 60 | 8 | 6 | 10 | 8 | 7 | 7 | 9 | 6 | 4 | 8 | 11 | 5 |
| 61 | 7 | 7 | 9 | 16 | 5 | 14 | 11 | 15 | 8 | 17 | 9 | 6 |
| 62 | 9 | 15 | 13 | 18 | 7 | 10 | 14 | 16 | 19 | 16 | 15 | 21 |
| 63 | 10 | 10 | 4 | 6 | 8 | 7 | 12 | 7 | 7 | 8 | 11 | 4 |
| 64 |  |  |  |  |  |  |  |  |  |  |  |  |
| 65 | 24 | 18 | 26 | 7 | 10 | 15 | 23 | 18 | 27 | 26 | 18 | 22 |
| 66 | 0 | 1 | 5 | 0 | 0 | 2 | 5 | 1 | 0 | 1 | 1 | 1 |
| 67 | 8 | 7 | 9 | 6 | 4 | 14 | 14 | 9 | 14 | 8 | 6 | 4 |
| 68 | 27 | 21 | 22 | 15 | 13 | 22 | 27 | 20 | 19 | 19 | 23 | 22 |
| 69 | 30 | 24 | 17 | 30 | 19 | 12 | 23 | 27 | 31 | 30 | 24 | 33 |
| 70 | 2 | 3 | 4 | 1 | 4 | 3 | 1 | 4 | 2 | 2 | 3 | 2 |
| 71 | 17 | 18 | 10 | 8 | 13 | 16 | 11 | 7 | 12 | 9 | 18 | 9 |
| 72 | 18 | 18 | 22 | 14 | 9 | 17 | 23 | 18 | 16 | 26 | 18 | 23 |
| 73 | 9 | 12 | 8 | 14 | 5 | 6 | 9 | 7 | 9 | 12 | 12 | 8 |
| 74 | 18 | 21 | 20 | 23 | 23 | 25 | 11 | 14 | 24 | 25 | 16 | 17 |
| 75 | 2 | 4 | 4 | 1 |  |  | 4 | 1 | 1 |  |  |  |
| 76 | 20 | 19 | 25 | 20 | 12 | 19 | 13 | 16 | 25 | 17 | 17 | 17 |
| 77 | 2 | 1 | 1 | 3 | 2 | 1 | 4 | 2 | 3 | 1 | 2 | 3 |
| 78 |  |  |  |  |  |  |  |  |  |  |  |  |
| 79 | 29 | 14 | 18 | 9 | 5 | 12 | 17 | 19 | 19 | 32 | 25 | 26 |
| 80 | 15 | 19 | 13 | 12 | 12 | 18 | 19 | 15 | 22 | 23 | 13 | 13 |
| 81 | 19 | 17 | 17 | 7 | 8 | 12 | 18 | 6 | 16 | 16 | 14 | 18 |
| 82 |  |  |  |  |  |  |  |  |  |  |  |  |
| 83 |  |  |  |  |  |  |  |  |  |  |  |  |
| 84 | 11 | 23 | 18 | 15 | 6 | 12 | 21 | 17 | 16 | 13 | 15 | 16 |
| 85 | 14 | 14 | 8 | 6 | 12 | 9 | 9 | 14 | 16 | 14 | 18 | 17 |
| 86 | 23 | 18 | 21 | 16 | 12 | 18 | 12 | 18 | 14 | 21 | 16 | 16 |
| 87 | 13 | 3 | 5 | 6 | 8 | 8 | 6 | 9 | 6 | 7 | 4 | 6 |
| 88 | 10 | 11 | 7 | 12 | 7 | 15 | 11 | 12 | 11 | 20 | 8 | 13 |
| 89 | 50 | 44 | 54 | 34 | 33 | 36 | 56 | 33 | 49 | 55 | 43 | 57 |
| 90 | 14 | 13 | 15 | 9 | 6 | 9 | 10 | 7 | 14 | 16 | 15 | 17 |
| 91 | 9 | 17 | 12 | 5 | 12 | 22 | 13 | 11 | 12 | 15 | 9 | 10 |
| 92 |  |  |  |  |  |  |  |  |  |  |  |  |
| 93 |  |  |  |  |  |  |  |  |  |  |  |  |
| 94 | 5 | 6 | 7 | 2 | 3 | 6 | 7 | 11 | 5 | 4 | 4 | 11 |
| 95 | 6 | 1 | 2 | 4 | 3 | 6 | 6 | 2 | 4 | 6 | 1 | 6 |
| 96 | 19 | 11 | 15 | 4 | 5 | 5 | 9 | 13 | 10 | 9 | 14 | 8 |
| 97 | 4 | 0 | 1 | 2 | 2 | 4 | 1 | 2 | 2 | 1 | 2 | 1 |
| 98 | 8 | 3 | 11 | 8 | 6 | 5 | 13 | 11 | 10 | 10 | 4 | 9 |
| 99 | 5 | 6 | 1 | 4 | 5 | 7 | 8 | 6 | 7 | 8 | 9 | 2 |
| 100 | 12 | 11 | 21 | 17 | 18 | 18 | 15 | 18 | 18 | 15 | 8 | 14 |
| 101 | 14 | 12 | 14 | 13 | 11 | 14 | 15 | 11 | 10 | 15 | 13 | 15 |
| 102 | 10 | 14 | 20 | 8 | 4 | 15 | 23 | 8 | 18 | 16 | 11 | 16 |
| 103 | 13 | 15 | 16 | 11 | 13 | 16 | 11 | 8 | 12 | 13 | 17 | 16 |
| 104 | 22 | 16 | 15 | 16 | 16 | 20 | 18 | 20 | 29 | 28 | 29 | 34 |
| 105 | 11 | 9 | 11 | 11 | 7 | 17 | 13 | 17 | 12 | 27 | 21 | 14 |

Table 3. Number of newly diagnosed stage I gastric cancer during each month in 2019.

|  | Month | | | | | | | | | | | |
| --- | --- | --- | --- | --- | --- | --- | --- | --- | --- | --- | --- | --- |
| Hospital | 1 | 2 | 3 | 4 | 5 | 6 | 7 | 8 | 9 | 10 | 11 | 12 |
| 1 | 0 | 1 | 1 | 2 | 4 | 2 | 2 | 3 | 6 | 0 | 3 | 3 |
| 2 | 63 | 62 | 81 | 75 | 62 | 63 | 84 | 64 | 75 | 73 | 72 | 71 |
| 3 | 3 | 3 | 2 | 7 | 6 | 6 | 3 | 7 | 0 | 0 | 1 | 1 |
| 4 | 7 | 6 | 10 | 13 | 5 | 6 | 11 | 9 | 4 | 8 | 11 | 6 |
| 5 | 5 | 5 | 9 | 4 | 4 | 1 | 4 | 10 | 3 | 2 | 4 | 11 |
| 6 | 1 | 5 | 5 | 7 | 4 | 9 | 11 | 9 | 6 | 11 | 8 | 7 |
| 7 | 3 | 8 | 9 | 9 | 6 | 10 | 16 | 9 | 8 | 7 | 9 | 14 |
| 8 | 2 | 0 | 0 | 0 | 1 | 0 | 1 | 0 | 0 | 1 | 2 | 1 |
| 9 | 3 | 5 | 5 | 5 | 7 | 5 | 6 | 10 | 3 | 7 | 5 | 5 |
| 10 | 5 | 5 | 4 | 7 | 7 | 12 | 10 | 5 | 5 | 8 | 10 | 7 |
| 11 | 10 | 15 | 13 | 21 | 14 | 20 | 20 | 14 | 14 | 13 | 16 | 14 |
| 12 | 2 | 0 | 1 | 1 | 3 | 4 | 3 | 4 | 2 | 2 | 4 | 3 |
| 13 | 11 | 7 | 6 | 12 | 13 | 10 | 18 | 18 | 13 | 23 | 14 | 7 |
| 14 | 3 | 4 | 5 | 8 | 7 | 10 | 13 | 7 | 5 | 15 | 11 | 8 |
| 15 | 23 | 14 | 29 | 27 | 16 | 19 | 28 | 23 | 26 | 19 | 31 | 17 |
| 16 | 27 | 18 | 33 | 36 | 16 | 30 | 33 | 21 | 15 | 25 | 18 | 23 |
| 17 |  |  |  |  |  |  |  |  |  |  |  |  |
| 18 | 3 | 5 | 4 | 5 | 5 | 3 | 2 | 4 | 4 | 9 | 1 | 10 |
| 19 | 3 | 4 | 5 | 5 | 9 | 8 | 7 | 5 | 6 | 5 | 8 | 3 |
| 20 | 41 | 39 | 31 | 36 | 39 | 41 | 50 | 40 | 43 | 41 | 50 | 33 |
| 21 | 5 | 6 | 9 | 7 | 9 | 4 | 7 | 6 | 4 | 9 | 4 | 10 |
| 22 | 2 | 2 | 2 | 3 | 3 | 6 | 7 | 5 | 6 | 2 | 6 | 2 |
| 23 | 34 | 31 | 49 | 46 | 47 | 43 | 44 | 30 | 42 | 29 | 34 | 37 |
| 24 | 7 | 16 | 9 | 12 | 15 | 12 | 14 | 10 | 12 | 7 | 12 | 12 |
| 25 | 7 | 13 | 14 | 13 | 11 | 17 | 7 | 16 | 8 | 10 | 16 | 19 |
| 26 | 14 | 12 | 2 | 9 | 10 | 10 | 20 | 6 | 6 | 12 | 6 | 16 |
| 27 | 2 | 2 | 0 | 1 | 0 | 1 | 0 | 0 | 0 | 2 | 1 | 1 |
| 28 | 3 | 6 | 10 | 6 | 6 | 3 | 10 | 14 | 8 | 16 | 8 | 9 |
| 29 |  |  |  |  |  |  |  |  |  |  |  |  |
| 30 | 4 | 6 | 9 | 5 | 4 | 12 | 9 | 11 | 4 | 8 | 7 | 9 |
| 31 | 6 | 5 | 8 | 10 | 4 | 4 | 4 | 8 | 1 | 11 | 9 | 7 |
| 32 | 4 | 4 | 5 | 3 | 4 | 8 | 7 | 7 | 5 | 4 | 5 | 2 |
| 33 | 3 | 3 | 6 | 4 | 3 | 4 | 7 | 5 | 7 | 8 | 3 | 7 |
| 34 |  |  |  |  |  |  |  |  |  |  |  |  |
| 35 | 8 | 6 | 18 | 10 | 13 | 19 | 9 | 14 | 15 | 15 | 10 | 8 |
| 36 | 11 | 7 | 9 | 8 | 13 | 6 | 7 | 11 | 10 | 10 | 10 | 7 |
| 37 | 15 | 26 | 22 | 22 | 24 | 25 | 33 | 31 | 30 | 29 | 28 | 26 |
| 38 |  |  |  |  |  |  |  |  |  |  |  |  |
| 39 |  | 1 | 2 | 1 | 2 | 2 | 3 | 2 | 1 | 5 | 2 | 1 |
| 40 | 6 | 5 | 3 | 7 | 8 | 6 | 9 | 9 | 12 | 11 | 5 | 6 |
| 41 | 19 | 12 | 12 | 18 | 15 | 7 | 14 | 16 | 17 | 14 | 14 | 11 |
| 42 | 11 | 6 | 9 | 8 | 12 | 5 | 8 | 8 | 6 | 7 | 4 | 2 |
| 43 | 9 | 14 | 13 | 9 | 5 | 6 | 9 | 15 | 8 | 11 | 12 | 9 |
| 44 | 3 | 1 | 0 | 0 | 2 | 1 | 1 | 3 | 0 | 1 | 0 | 0 |
| 45 | 8 | 5 | 12 | 6 | 4 | 10 | 10 | 5 | 10 | 8 | 14 | 5 |
| 46 | 10 | 10 | 12 | 13 | 6 | 8 | 9 | 9 | 14 | 12 | 12 | 15 |
| 47 | 5 | 6 | 6 | 7 | 6 | 1 | 7 | 3 | 2 | 5 | 3 | 5 |
| 48 | 9 | 5 | 10 | 9 | 10 | 5 | 8 | 9 | 8 | 15 | 7 | 14 |
| 49 | 3 | 13 | 12 | 6 | 8 | 4 | 13 | 11 | 4 | 7 | 5 | 11 |
| 50 | 1 | 1 | 2 | 2 | 1 | 2 | 1 | 0 | 0 | 2 | 3 | 1 |
| 51 | 11 | 11 | 12 | 14 | 17 | 13 | 25 | 20 | 20 | 22 | 19 | 17 |
| 52 | 13 | 8 | 12 | 17 | 9 | 17 | 18 | 16 | 14 | 15 | 13 | 15 |
| 53 | 16 | 8 | 16 | 17 | 16 | 11 | 20 | 10 | 15 | 12 | 9 | 21 |
| 54 | 35 | 36 | 34 | 33 | 32 | 43 | 61 | 37 | 47 | 47 | 43 | 53 |
| 55 | 4 | 5 | 6 | 8 | 5 | 6 | 7 | 2 | 8 | 6 | 9 | 6 |
| 56 | 20 | 22 | 22 | 15 | 20 | 16 | 20 | 22 | 26 | 6 | 11 | 15 |
| 57 | 25 | 27 | 22 | 22 | 20 | 23 | 30 | 20 | 11 | 13 | 23 | 17 |
| 58 | 5 | 2 | 6 | 6 | 5 | 3 | 11 | 8 | 12 | 14 | 9 | 17 |
| 59 | 6 | 9 | 3 | 4 | 6 | 5 | 9 | 2 | 6 | 5 | 4 | 2 |
| 60 | 9 | 8 | 7 | 10 | 9 | 6 | 4 | 10 | 3 | 4 | 9 | 5 |
| 61 | 5 | 6 | 10 | 11 | 10 | 4 | 10 | 8 | 13 | 9 | 6 | 10 |
| 62 |  |  |  |  |  |  |  |  |  |  |  |  |
| 63 | 7 | 4 | 5 | 7 | 6 | 6 | 3 | 15 | 3 | 6 | 6 | 4 |
| 64 | 19 | 12 | 11 | 15 | 9 | 13 | 22 | 12 | 13 | 17 | 12 | 16 |
| 65 | 18 | 22 | 13 | 10 | 13 | 17 | 25 | 29 | 13 | 16 | 25 | 20 |
| 66 | 1 | 0 | 2 | 2 | 0 | 3 | 4 | 4 | 2 | 0 | 1 | 1 |
| 67 | 4 | 2 | 5 | 4 | 8 | 7 | 9 | 7 | 5 | 5 | 5 | 4 |
| 68 | 17 | 21 | 23 | 10 | 19 | 13 | 18 | 19 | 17 | 12 | 24 | 16 |
| 69 | 15 | 16 | 18 | 17 | 10 | 12 | 21 | 21 | 17 | 8 | 16 | 23 |
| 70 |  |  | 1 | 4 | 1 | 1 | 2 | 4 | 2 | 1 | 1 | 4 |
| 71 | 14 | 9 | 14 | 7 | 14 | 10 | 13 | 7 | 7 | 15 | 11 | 11 |
| 72 | 17 | 16 | 18 | 13 | 12 | 12 | 10 | 15 | 12 | 11 | 15 | 20 |
| 73 | 8 | 10 | 11 | 10 | 8 | 6 | 9 | 7 | 1 | 8 | 8 | 7 |
| 74 | 19 | 20 | 16 | 9 | 12 | 22 | 13 | 13 | 19 | 19 | 13 | 11 |
| 75 | 5 | 5 | 1 | 2 | 1 | 1 | 2 |  | 2 | 3 | 1 | 1 |
| 76 | 14 | 10 | 4 | 8 | 11 | 11 | 11 | 7 | 22 | 10 | 8 | 8 |
| 77 | 2 | 1 |  | 4 | 3 |  |  | 2 | 2 | 6 |  | 2 |
| 78 | 1 |  | 1 |  | 1 |  | 1 | 3 | 1 | 2 | 1 | 2 |
| 79 | 18 | 14 | 17 | 12 | 11 | 15 | 12 | 18 | 21 | 22 | 18 | 19 |
| 80 | 10 | 15 | 8 | 19 | 14 | 14 | 10 | 11 | 21 | 11 | 12 | 8 |
| 81 | 11 | 7 | 27 | 13 | 14 | 11 | 16 | 7 | 11 | 15 | 14 | 17 |
| 82 |  |  |  |  |  |  |  |  |  |  |  |  |
| 83 | 4 | 11 | 5 | 7 | 12 | 11 | 7 | 6 | 5 | 10 | 13 | 6 |
| 84 | 12 | 7 | 16 | 11 | 13 | 10 | 15 | 9 | 7 | 12 | 10 | 15 |
| 85 | 10 | 3 | 8 | 11 | 7 | 9 | 10 | 5 | 6 | 10 | 4 | 12 |
| 86 | 8 | 12 | 9 | 8 | 11 | 12 | 5 | 12 | 11 | 17 | 13 | 10 |
| 87 | 2 | 2 | 3 | 4 | 1 | 6 | 4 | 1 | 3 | 4 | 1 | 4 |
| 88 | 6 | 6 | 4 | 3 | 10 | 3 | 7 | 9 | 2 | 9 | 6 | 5 |
| 89 | 33 | 33 | 30 | 22 | 26 | 37 | 36 | 36 | 23 | 30 | 26 | 31 |
| 90 | 7 | 9 | 5 | 13 | 12 | 8 | 7 | 15 | 5 | 8 | 5 | 9 |
| 91 | 14 | 3 | 5 | 9 | 10 | 19 | 4 | 10 | 4 | 7 | 6 | 8 |
| 92 |  |  |  |  |  |  |  |  |  |  |  |  |
| 93 | 7 | 9 | 6 | 9 | 8 | 2 | 5 | 4 | 5 | 10 | 5 | 8 |
| 94 | 3 | 4 | 2 | 2 | 5 | 3 | 4 | 3 | 7 | 2 | 3 | 3 |
| 95 | 2 | 4 | 1 | 3 | 0 | 1 | 5 | 2 | 2 | 2 | 1 | 2 |
| 96 | 5 | 10 | 13 | 2 | 5 | 8 | 7 | 8 | 6 | 5 | 5 | 6 |
| 97 | 0 | 1 | 1 | 2 | 0 | 2 | 3 | 4 | 1 | 1 | 0 | 1 |
| 98 | 11 | 10 | 6 | 4 | 3 | 2 | 8 | 5 | 4 | 2 | 4 | 4 |
| 99 | 1 | 4 | 8 | 3 | 0 | 2 | 3 | 3 | 2 | 3 | 4 | 1 |
| 100 | 15 | 13 | 12 | 15 | 9 | 13 | 13 | 9 | 16 | 14 | 9 | 7 |
| 101 | 13 | 4 | 5 | 7 | 7 | 4 | 10 | 13 | 9 | 6 | 8 | 10 |
| 102 | 7 | 8 | 14 | 10 | 5 | 11 | 13 | 9 | 13 | 5 | 15 | 7 |
| 103 | 5 | 4 | 7 | 12 | 13 | 11 | 15 | 8 | 5 | 11 | 12 | 20 |
| 104 | 7 | 15 | 9 | 13 | 12 | 12 | 14 | 14 | 17 | 14 | 15 | 14 |
| 105 | 5 | 7 | 12 | 8 | 10 | 12 | 14 | 6 | 7 | 11 | 10 | 12 |

Table 4. Number of newly diagnosed stage I gastric cancer during each month in 2020.

|  | Month | | | | | | | | | | | |
| --- | --- | --- | --- | --- | --- | --- | --- | --- | --- | --- | --- | --- |
| Hospital | 1 | 2 | 3 | 4 | 5 | 6 | 7 | 8 | 9 | 10 | 11 | 12 |
| 1 | 2 | 1 | 4 | 2 | 3 | 3 | 2 | 3 | 2 | 4 | 3 | 3 |
| 2 | 59 | 66 | 61 | 34 | 14 | 44 | 51 | 53 | 56 | 55 | 46 | 62 |
| 3 | 1 | 4 | 2 | 5 | 1 | 2 | 4 | 4 | 7 | 9 | 4 | 4 |
| 4 | 8 | 7 | 8 | 5 | 6 | 5 | 4 | 2 | 2 | 9 | 4 | 8 |
| 5 | 6 | 2 | 9 | 5 | 3 | 3 | 6 | 7 | 2 | 12 | 8 | 3 |
| 6 | 7 | 3 | 4 | 8 | 12 | 8 | 4 | 2 | 9 | 4 | 6 | 10 |
| 7 | 12 | 12 | 9 | 10 | 7 | 6 | 11 | 11 | 10 | 10 | 10 | 9 |
| 8 | 0 | 2 | 1 | 4 | 8 | 1 | 2 | 0 | 1 | 3 | 2 | 7 |
| 9 | 8 | 7 | 6 | 7 | 1 | 7 | 5 | 4 | 1 | 3 | 5 | 4 |
| 10 | 6 | 5 | 3 | 7 | 5 | 6 | 5 | 2 | 7 | 7 | 4 | 7 |
| 11 | 9 | 15 | 6 | 10 | 6 | 7 | 10 | 10 | 15 | 9 | 12 | 16 |
| 12 | 4 | 3 | 3 | 0 | 7 | 4 | 3 | 1 | 1 | 3 | 3 | 3 |
| 13 | 11 | 7 | 10 | 7 | 7 | 6 | 11 | 12 | 11 | 12 | 7 | 16 |
| 14 | 7 | 4 | 4 | 1 | 6 | 7 | 6 | 12 | 9 | 9 | 9 | 6 |
| 15 | 20 | 22 | 23 | 19 | 7 | 18 | 19 | 12 | 19 | 21 | 18 | 23 |
| 16 | 20 | 31 | 24 | 28 | 13 | 13 | 28 | 20 | 25 | 22 | 33 | 27 |
| 17 |  |  |  |  |  |  |  |  |  |  |  |  |
| 18 | 0 | 2 | 4 | 2 | 2 | 5 | 3 | 7 | 2 | 4 | 9 | 6 |
| 19 | 4 | 3 | 2 | 3 | 4 | 6 | 5 | 2 | 8 | 3 | 6 | 4 |
| 20 | 40 | 42 | 44 | 11 | 12 | 21 | 26 | 24 | 29 | 31 | 34 | 27 |
| 21 | 2 | 6 | 8 | 2 | 5 | 6 | 9 | 11 | 5 | 4 | 9 | 5 |
| 22 | 3 | 2 |  | 4 | 2 | 6 | 5 | 3 | 3 | 7 | 6 | 4 |
| 23 | 32 | 33 | 44 | 18 | 17 | 25 | 19 | 26 | 34 | 40 | 34 | 47 |
| 24 | 14 | 7 | 15 | 8 | 8 | 13 | 7 | 10 | 9 | 13 | 8 | 10 |
| 25 | 8 | 11 | 12 | 9 | 4 | 9 | 9 | 8 | 14 | 12 | 13 | 12 |
| 26 | 7 | 6 | 6 | 8 | 6 | 5 | 9 | 11 |  | 5 | 8 | 12 |
| 27 | 6 | 0 | 2 | 1 | 1 | 3 | 0 | 1 | 0 | 0 | 1 | 1 |
| 28 | 7 | 3 | 10 | 3 | 2 | 3 | 10 | 5 | 10 | 4 | 7 | 5 |
| 29 |  |  |  |  |  |  |  |  |  |  |  |  |
| 30 | 5 | 8 | 10 | 5 | 4 | 5 | 6 | 7 | 5 | 8 | 10 | 11 |
| 31 | 2 | 11 | 4 | 5 | 1 | 2 | 3 | 3 | 6 | 8 | 9 | 5 |
| 32 | 2 | 2 | 1 | 2 | 2 | 2 | 3 | 1 | 2 | 4 | 3 | 4 |
| 33 | 6 | 4 | 4 | 11 | 5 | 6 | 10 | 2 | 11 | 10 | 3 | 7 |
| 34 |  |  |  |  |  |  |  |  |  |  |  |  |
| 35 | 13 | 13 | 13 | 11 | 10 | 8 | 15 | 11 | 8 | 12 | 6 | 9 |
| 36 | 7 | 10 | 8 | 15 | 11 | 10 | 8 | 9 | 16 | 10 | 10 | 13 |
| 37 | 28 | 16 | 26 | 13 | 16 | 25 | 22 | 17 | 21 | 14 | 23 | 29 |
| 38 |  |  |  |  |  |  |  |  |  |  |  |  |
| 39 | 4 | 2 | 1 | 3 |  | 1 | 1 | 2 | 4 | 3 | 1 | 2 |
| 40 | 1 | 4 | 7 | 6 | 4 | 3 | 11 | 6 | 7 | 11 | 4 | 5 |
| 41 | 14 | 16 | 13 | 13 | 11 | 2 | 13 | 15 | 10 | 11 | 5 | 6 |
| 42 | 7 | 8 | 5 | 3 | 5 | 1 | 3 | 4 | 6 | 3 | 5 | 3 |
| 43 | 9 | 6 | 6 | 6 | 8 | 6 | 13 | 7 | 9 | 10 | 5 | 4 |
| 44 | 1 | 1 | 2 | 1 | 2 | 0 | 0 | 2 | 4 | 1 | 1 | 1 |
| 45 | 9 | 12 | 6 | 5 | 2 | 4 | 8 | 5 | 8 | 14 | 6 | 10 |
| 46 | 16 | 14 | 11 | 10 | 9 | 6 | 8 | 10 | 10 | 20 | 15 | 10 |
| 47 | 2 |  | 2 | 4 | 2 | 4 | 3 | 3 | 4 |  | 2 | 8 |
| 48 | 7 | 8 | 7 | 4 | 3 | 4 | 6 | 7 | 4 | 10 | 7 | 7 |
| 49 | 8 | 5 | 3 | 7 | 4 | 12 | 0 | 6 | 7 | 3 | 3 | 7 |
| 50 | 1 | 1 | 0 | 0 | 0 | 2 | 2 | 1 | 1 | 1 | 3 | 0 |
| 51 | 11 | 22 | 18 | 21 | 10 | 19 | 23 | 27 | 17 | 18 | 21 | 18 |
| 52 | 11 | 9 | 16 | 11 | 6 | 8 | 12 | 14 | 13 | 10 | 14 | 5 |
| 53 | 8 | 13 | 9 | 15 | 5 | 10 | 16 | 9 | 19 | 22 | 10 | 16 |
| 54 | 45 | 47 | 40 | 28 | 27 | 29 | 33 | 32 | 40 | 54 | 49 | 37 |
| 55 | 6 | 4 | 7 | 5 | 2 | 2 | 5 | 7 | 4 | 2 | 3 | 3 |
| 56 | 12 | 15 | 17 | 7 | 5 | 13 | 17 | 16 | 18 | 16 | 18 | 15 |
| 57 | 21 | 18 | 25 | 23 | 18 | 39 | 22 | 22 | 17 | 29 | 19 | 26 |
| 58 | 14 | 11 | 14 | 8 | 4 | 5 | 6 | 5 | 6 | 11 | 12 | 13 |
| 59 | 4 | 7 | 8 | 4 | 5 | 3 | 7 | 6 | 5 | 6 | 5 | 4 |
| 60 | 4 | 4 | 5 | 3 | 4 | 1 | 5 | 5 | 2 | 4 | 7 | 4 |
| 61 | 4 | 5 | 7 | 12 | 3 | 10 | 5 | 8 | 5 | 9 | 6 | 3 |
| 62 |  |  |  |  |  |  |  |  |  |  |  |  |
| 63 | 9 | 10 | 1 | 4 | 4 | 6 | 11 | 6 | 1 | 7 | 7 | 3 |
| 64 | 11 | 10 | 6 | 8 | 5 | 14 | 12 | 6 | 9 | 15 | 10 | 14 |
| 65 | 16 | 8 | 14 | 5 | 5 | 7 | 10 | 13 | 13 | 20 | 13 | 18 |
| 66 | 0 | 1 | 2 | 0 | 0 | 1 | 1 | 1 | 0 | 1 | 1 | 1 |
| 67 | 6 | 5 | 7 | 2 | 2 | 1 | 9 | 5 | 11 | 7 | 2 | 2 |
| 68 | 21 | 17 | 18 | 6 | 6 | 15 | 12 | 11 | 11 | 12 | 19 | 18 |
| 69 | 16 | 11 | 12 | 15 | 9 | 7 | 10 | 16 | 17 | 18 | 11 | 12 |
| 70 | 1 | 1 | 2 |  | 2 | 2 | 1 |  | 1 |  | 3 |  |
| 71 | 9 | 11 | 6 | 4 | 12 | 8 | 7 | 4 | 9 | 4 | 14 | 8 |
| 72 | 10 | 15 | 13 | 5 | 4 | 11 | 13 | 12 | 12 | 18 | 10 | 10 |
| 73 | 7 | 9 | 2 | 5 | 2 | 2 | 6 | 4 | 5 | 6 | 5 | 4 |
| 74 | 13 | 15 | 14 | 14 | 9 | 13 | 7 | 9 | 16 | 22 | 11 | 14 |
| 75 | 2 | 4 | 4 | 1 |  |  | 3 | 1 | 1 |  |  |  |
| 76 | 12 | 12 | 18 | 10 | 3 | 10 | 9 | 11 | 15 | 10 | 10 | 13 |
| 77 |  | 1 | 1 |  | 1 |  |  |  |  |  | 1 | 2 |
| 78 | 1 |  |  |  | 1 | 2 | 3 | 3 | 2 | 2 |  | 2 |
| 79 | 17 | 10 | 10 | 4 | 2 | 4 | 10 | 13 | 12 | 26 | 14 | 18 |
| 80 | 9 | 15 | 8 | 9 | 7 | 12 | 10 | 11 | 16 | 11 | 8 | 7 |
| 81 | 13 | 11 | 10 | 4 |  | 6 | 12 | 2 | 11 | 12 | 12 | 12 |
| 82 |  |  |  |  |  |  |  |  |  |  |  |  |
| 83 | 6 | 6 | 8 | 6 | 11 | 7 | 9 | 12 | 7 | 8 | 11 | 15 |
| 84 | 7 | 14 | 15 | 11 | 2 | 7 | 13 | 13 | 8 | 7 | 8 | 6 |
| 85 | 11 | 8 | 5 | 4 | 2 | 4 | 5 | 8 | 9 | 8 | 11 | 8 |
| 86 | 13 | 9 | 16 | 7 | 6 | 13 | 9 | 11 | 8 | 18 | 10 | 10 |
| 87 | 8 |  | 3 | 3 | 3 | 3 | 4 | 2 | 4 | 5 | 1 | 4 |
| 88 | 3 | 6 | 3 | 4 | 1 | 7 | 4 | 9 | 6 | 6 | 4 | 7 |
| 89 | 29 | 19 | 35 | 17 | 21 | 20 | 32 | 20 | 25 | 34 | 26 | 32 |
| 90 | 8 | 9 | 8 | 6 | 4 | 4 | 4 | 5 | 10 | 11 | 7 | 10 |
| 91 | 5 | 13 | 6 | 3 | 9 | 15 | 7 | 9 | 6 | 6 | 6 | 7 |
| 92 |  |  |  |  |  |  |  |  |  |  |  |  |
| 93 | 4 | 11 | 7 | 5 | 2 | 8 | 6 | 3 | 3 | 7 | 8 | 4 |
| 94 | 2 | 4 | 4 | 0 | 2 | 2 | 4 | 6 | 3 | 3 | 1 | 4 |
| 95 | 4 | 1 | 2 | 1 | 2 | 4 | 4 | 0 | 1 | 3 | 0 | 3 |
| 96 | 10 | 8 | 10 | 2 | 1 | 1 | 8 | 8 | 3 | 6 | 9 | 2 |
| 97 | 1 | 0 | 0 | 0 | 0 | 1 | 0 | 1 | 1 | 0 | 0 | 0 |
| 98 | 1 | 1 | 5 | 5 | 2 | 2 | 5 | 5 | 4 | 5 | 3 | 5 |
| 99 | 2 | 3 | 1 | 3 | 5 | 4 | 2 | 2 | 1 | 5 | 2 | 1 |
| 100 | 7 | 7 | 18 | 12 | 14 | 12 | 9 | 16 | 16 | 9 | 7 | 10 |
| 101 | 5 | 5 | 5 | 8 | 6 | 6 | 7 | 5 | 5 | 6 | 5 | 11 |
| 102 | 2 | 0 | 0 | 0 | 0 | 0 | 1 | 0 | 1 | 0 | 1 | 0 |
| 103 | 10 | 11 | 11 | 8 | 5 | 12 | 9 | 5 | 4 | 7 | 11 | 13 |
| 104 | 12 | 7 | 6 | 8 | 8 | 12 | 9 | 11 | 20 | 17 | 16 | 20 |
| 105 | 8 | 6 | 5 | 4 | 4 | 14 | 9 | 14 | 4 | 18 | 13 | 9 |

Table 5. Number of newly diagnosed stage II gastric cancer during each month in 2019.

|  | Month | | | | | | | | | | | |
| --- | --- | --- | --- | --- | --- | --- | --- | --- | --- | --- | --- | --- |
| Hospital | 1 | 2 | 3 | 4 | 5 | 6 | 7 | 8 | 9 | 10 | 11 | 12 |
| 1 | 0 | 0 | 0 | 0 | 0 | 0 | 1 | 1 | 0 | 0 | 0 | 0 |
| 2 | 7 | 2 | 4 | 7 | 6 | 4 | 8 | 5 | 5 | 8 | 5 | 5 |
| 3 | 0 | 0 | 0 | 1 | 1 | 0 | 1 | 0 | 1 | 0 | 2 | 1 |
| 4 | 1 | 0 | 0 | 1 | 1 | 3 | 1 | 1 | 0 | 3 | 0 | 3 |
| 5 | 0 | 2 | 0 | 0 | 1 | 1 | 1 | 1 | 2 | 0 | 1 | 2 |
| 6 | 1 | 2 | 2 | 1 | 2 | 2 | 0 | 0 | 1 | 3 | 2 | 0 |
| 7 | 2 | 1 | 1 | 1 | 1 | 3 | 5 | 0 | 0 | 2 | 1 | 0 |
| 8 | 0 | 1 | 0 | 0 | 0 | 0 | 1 | 0 | 0 | 0 | 0 | 0 |
| 9 | 0 | 1 | 0 | 2 | 1 | 1 | 1 | 3 | 2 | 0 | 4 | 2 |
| 10 |  | 3 | 1 |  |  | 1 | 1 | 1 |  | 3 | 1 | 1 |
| 11 | 3 | 1 | 5 | 1 | 1 | 1 | 4 | 1 | 1 | 3 | 1 | 2 |
| 12 | 0 | 0 | 1 | 1 | 0 | 0 | 0 | 2 | 0 | 0 | 0 | 0 |
| 13 | 2 | 1 | 0 | 2 | 1 | 1 | 2 | 4 | 3 | 1 | 2 | 0 |
| 14 | 0 | 2 | 1 | 4 | 0 | 1 | 2 | 2 | 3 | 2 | 0 | 1 |
| 15 | 3 | 2 | 2 | 2 | 3 | 1 | 0 | 4 | 1 | 4 | 0 | 3 |
| 16 | 3 | 1 | 3 | 1 | 0 | 1 | 1 | 1 | 1 | 4 | 4 | 1 |
| 17 |  |  |  |  |  |  |  |  |  |  |  |  |
| 18 | 0 | 0 | 0 | 0 | 0 | 0 | 0 | 1 | 5 | 1 | 1 | 0 |
| 19 | 0 | 0 | 1 | 2 | 0 | 0 | 1 | 0 | 0 | 0 | 1 | 1 |
| 20 | 3 | 3 | 4 | 4 | 1 | 2 | 5 | 5 | 3 | 3 | 2 | 1 |
| 21 | 2 | 0 | 1 | 1 | 2 | 2 | 2 | 2 | 0 | 3 | 1 | 1 |
| 22 | 1 | 2 | 1 |  | 1 | 1 |  |  |  |  |  | 1 |
| 23 | 3 | 3 | 3 | 0 | 5 | 3 | 4 | 2 | 1 | 3 | 1 | 7 |
| 24 | 1 | 1 | 1 | 2 | 7 | 2 | 0 | 3 | 1 | 1 | 3 | 4 |
| 25 | 3 | 3 | 7 | 1 | 1 | 4 | 4 | 2 | 2 | 4 | 1 | 0 |
| 26 |  |  | 1 | 2 | 1 |  | 2 | 2 | 2 | 1 |  | 1 |
| 27 | 0 | 1 | 2 | 2 | 2 | 0 | 1 | 1 | 0 | 2 | 2 | 0 |
| 28 | 3 | 0 | 1 | 0 | 1 | 2 | 2 | 1 | 1 | 2 | 1 | 1 |
| 29 |  |  |  |  |  |  |  |  |  |  |  |  |
| 30 | 1 | 0 | 1 | 1 | 0 | 1 | 1 | 1 | 0 | 3 | 0 | 2 |
| 31 | 1 | 0 | 1 | 0 | 1 | 0 | 0 | 0 | 1 | 2 | 1 | 1 |
| 32 | 1 | 2 |  |  |  |  |  | 2 | 3 |  |  | 1 |
| 33 | 2 | 2 | 3 |  | 1 | 3 | 1 |  | 1 | 1 |  |  |
| 34 |  |  |  |  |  |  |  |  |  |  |  |  |
| 35 | 0 | 2 | 3 | 0 | 2 | 2 | 1 | 4 | 2 | 3 | 2 | 2 |
| 36 |  | 2 |  |  | 1 | 1 | 1 |  | 2 |  |  |  |
| 37 | 1 | 0 | 1 | 3 | 3 | 2 | 1 | 3 | 3 | 3 | 2 | 3 |
| 38 |  |  |  |  |  |  |  |  |  |  |  |  |
| 39 |  |  |  |  |  |  |  | 1 |  |  |  |  |
| 40 | 0 | 2 | 1 | 1 | 1 | 0 | 0 | 1 | 0 | 0 | 1 | 0 |
| 41 | 3 | 1 | 0 | 0 | 1 | 4 | 0 | 1 | 1 | 4 | 2 | 0 |
| 42 | 1 | 0 | 1 | 0 | 0 | 2 | 1 | 0 | 0 | 1 | 0 | 0 |
| 43 | 2 | 1 | 2 | 1 | 1 | 3 | 2 | 0 | 0 | 1 | 3 | 1 |
| 44 | 0 | 1 | 0 | 3 | 0 | 0 | 0 | 1 | 0 | 0 | 0 | 2 |
| 45 | 1 | 2 | 1 | 3 | 2 | 1 | 1 | 0 | 0 | 0 | 1 | 0 |
| 46 | 1 | 2 | 1 | 3 | 2 | 0 | 1 | 1 | 3 | 3 | 1 | 3 |
| 47 |  |  |  | 1 |  |  | 1 |  | 1 | 1 |  |  |
| 48 | 0 | 2 | 2 | 2 | 1 | 0 | 1 | 2 | 1 | 0 | 1 | 1 |
| 49 | 2 | 1 | 1 | 1 | 1 | 1 | 1 | 0 | 1 | 0 | 1 | 0 |
| 50 | 0 | 0 | 0 | 2 | 0 | 1 | 0 | 0 | 0 | 1 | 0 | 0 |
| 51 | 0 | 2 | 2 | 1 | 1 | 1 | 3 | 1 | 4 | 4 | 2 | 3 |
| 52 | 2 | 3 | 2 | 3 | 2 | 2 | 2 | 4 | 1 | 3 | 1 | 2 |
| 53 | 0 | 1 | 2 | 2 | 0 | 2 | 1 | 2 | 1 | 1 | 2 | 2 |
| 54 | 6 | 4 | 5 | 0 | 2 | 4 | 3 | 3 | 5 | 3 | 4 | 0 |
| 55 | 2 | 0 | 2 | 0 | 2 | 2 | 0 | 1 | 1 | 0 | 0 | 2 |
| 56 | 2 | 5 | 1 | 3 | 1 | 0 | 2 | 2 | 2 | 0 | 4 | 1 |
| 57 | 5 | 2 | 3 | 3 | 6 | 2 | 4 | 2 | 4 | 2 | 1 | 2 |
| 58 | 1 | 0 | 1 | 2 | 0 | 0 | 1 | 1 | 0 | 1 | 1 | 2 |
| 59 | 0 | 0 | 0 | 0 | 1 | 0 | 1 | 3 | 1 | 1 | 1 | 0 |
| 60 | 1 | 1 | 1 | 2 | 2 | 3 | 1 | 2 | 0 | 0 | 2 | 4 |
| 61 | 2 | 1 | 0 | 1 | 2 | 1 | 3 | 1 | 1 | 2 | 1 | 1 |
| 62 |  |  |  |  |  |  |  |  |  |  |  |  |
| 63 | 1 |  |  |  | 1 | 2 |  | 1 |  |  | 1 | 2 |
| 64 | 1 | 2 |  | 2 | 4 | 2 | 1 |  | 1 | 1 |  | 1 |
| 65 | 0 | 2 | 0 | 1 | 3 | 2 | 2 | 3 | 0 | 0 | 0 | 0 |
| 66 | 0 | 0 | 0 | 0 | 1 | 1 | 0 | 1 | 0 | 0 | 0 | 0 |
| 67 | 1 | 0 | 1 | 0 | 0 | 1 | 1 | 3 | 0 | 2 | 2 | 0 |
| 68 | 2 | 1 | 2 | 0 | 1 | 0 | 2 | 2 | 3 | 1 | 1 | 2 |
| 69 | 4 | 5 | 2 | 2 | 2 | 2 | 2 | 1 | 4 | 6 | 2 | 2 |
| 70 |  |  |  |  |  |  |  |  |  |  | 1 |  |
| 71 | 0 | 2 | 0 | 1 | 0 | 1 | 2 | 1 | 4 | 0 | 0 | 2 |
| 72 | 1 | 2 | 1 | 0 | 5 | 0 | 3 | 1 | 2 | 1 | 0 | 0 |
| 73 |  | 4 | 1 |  | 2 |  | 1 | 3 |  |  |  | 1 |
| 74 | 1 | 2 | 3 | 6 | 2 | 3 | 0 | 2 | 3 | 2 | 2 | 0 |
| 75 |  |  |  |  |  |  |  |  |  |  |  |  |
| 76 | 0 | 0 | 2 | 0 | 0 | 1 | 0 | 0 | 1 | 3 | 2 | 2 |
| 77 |  |  |  |  |  | 1 |  |  |  |  | 1 | 2 |
| 78 |  |  |  |  |  | 1 |  | 3 |  |  |  |  |
| 79 | 1 | 2 | 2 | 2 | 1 | 1 | 4 | 2 | 0 | 2 | 3 | 0 |
| 80 | 2 | 1 | 0 | 1 | 1 | 0 | 2 | 3 | 3 | 2 | 1 | 1 |
| 81 |  |  | 1 | 1 |  |  |  | 3 | 1 | 2 |  | 2 |
| 82 |  |  |  |  |  |  |  |  |  |  |  |  |
| 83 | 0 | 0 | 0 | 1 | 1 | 1 | 1 | 0 | 2 | 0 | 1 | 2 |
| 84 | 3 | 4 | 1 | 3 | 1 | 2 | 1 | 1 | 0 | 2 | 1 | 3 |
| 85 | 1 | 2 | 0 | 2 | 0 | 1 | 2 | 2 | 1 | 0 | 0 | 0 |
| 86 | 1 | 1 | 1 | 0 | 2 | 0 | 3 | 2 | 1 | 2 | 3 | 1 |
| 87 |  | 2 |  |  | 2 | 1 | 1 | 2 |  |  |  |  |
| 88 | 1 | 2 | 0 | 0 | 0 | 1 | 0 | 0 | 0 | 0 | 0 | 3 |
| 89 | 1 | 4 | 2 | 1 | 2 | 4 | 3 | 6 | 3 | 5 | 4 | 4 |
| 90 | 1 | 1 | 0 | 2 | 0 | 0 | 1 | 0 | 0 | 0 | 0 | 0 |
| 91 | 0 | 1 | 0 | 1 | 0 | 0 | 2 | 3 | 0 | 1 | 1 | 0 |
| 92 |  |  |  |  |  |  |  |  |  |  |  |  |
| 93 | 0 | 1 | 2 | 2 | 1 | 0 | 0 | 0 | 0 | 0 | 1 | 1 |
| 94 | 1 | 0 | 0 | 3 | 3 | 1 | 1 | 0 | 0 | 1 | 0 | 1 |
| 95 | 1 | 0 | 0 | 0 | 0 | 0 | 0 | 0 | 0 | 0 | 0 | 0 |
| 96 | 1 | 2 | 0 | 0 | 2 | 0 | 1 | 0 | 1 | 1 | 0 | 0 |
| 97 | 1 | 0 | 0 | 1 | 0 | 2 | 0 | 0 | 0 | 0 | 0 | 0 |
| 98 | 2 | 2 | 2 | 3 | 2 | 0 | 0 | 1 | 0 | 2 | 3 | 1 |
| 99 | 0 | 0 | 1 | 1 | 0 | 0 | 1 | 0 | 1 | 0 | 1 | 0 |
| 100 | 1 | 1 | 3 | 1 | 0 | 1 | 1 | 0 | 2 | 2 | 2 | 1 |
| 101 |  | 2 |  | 1 | 1 |  |  | 4 | 2 | 1 | 2 | 2 |
| 102 | 0 | 0 | 2 | 0 | 2 | 1 | 2 | 2 | 2 | 2 | 1 | 1 |
| 103 | 2 | 1 | 0 | 3 | 1 | 2 | 3 | 1 | 1 | 2 | 1 | 2 |
| 104 | 5 | 0 | 1 | 3 | 3 | 0 | 5 | 2 | 0 | 6 | 0 | 6 |
| 105 | 2 | 1 | 0 | 1 | 1 | 2 | 2 | 0 | 0 | 1 | 1 | 0 |

Table 6. Number of newly diagnosed stage II gastric cancer during each month in 2020.

|  | Month | | | | | | | | | | | |
| --- | --- | --- | --- | --- | --- | --- | --- | --- | --- | --- | --- | --- |
| Hospital | 1 | 2 | 3 | 4 | 5 | 6 | 7 | 8 | 9 | 10 | 11 | 12 |
| 1 | 2 | 0 | 0 | 0 | 0 | 0 | 0 | 0 | 0 | 0 | 0 | 0 |
| 2 | 6 | 3 | 5 | 4 | 2 | 3 | 6 | 2 | 4 | 6 | 8 | 1 |
| 3 | 1 | 0 | 0 | 1 | 0 | 0 | 0 | 1 | 1 | 0 | 1 | 0 |
| 4 | 2 | 0 | 0 | 1 | 0 | 0 | 1 | 1 | 1 | 1 | 1 | 1 |
| 5 | 1 | 0 | 1 | 0 | 0 | 0 | 0 | 0 | 2 | 1 | 0 | 0 |
| 6 | 1 | 2 | 2 | 0 | 0 | 0 | 0 | 0 | 1 | 2 | 2 | 2 |
| 7 | 0 | 3 | 2 | 0 | 1 | 1 | 0 | 0 | 5 | 0 | 3 | 0 |
| 8 | 0 | 0 | 1 | 1 | 0 | 0 | 0 | 0 | 1 | 1 | 0 | 1 |
| 9 | 1 | 1 | 1 | 0 | 1 | 1 | 3 | 1 | 0 | 0 | 2 | 1 |
| 10 | 1 | 1 | 3 |  |  |  |  |  |  |  | 1 |  |
| 11 | 1 | 0 | 1 | 0 | 0 | 3 | 0 | 0 | 1 | 2 | 1 | 1 |
| 12 | 1 | 0 | 0 | 0 | 0 | 0 | 2 | 0 | 0 | 1 | 0 | 1 |
| 13 | 2 | 2 | 1 | 1 | 1 | 1 | 0 | 1 | 2 | 2 | 3 | 2 |
| 14 | 2 | 1 | 1 | 2 | 1 | 0 | 0 | 0 | 1 | 1 | 2 | 1 |
| 15 | 5 | 3 | 4 | 2 | 0 | 1 | 3 | 1 | 2 | 0 | 1 | 2 |
| 16 | 1 | 2 | 2 | 0 | 0 | 1 | 3 | 2 | 5 | 2 | 1 | 3 |
| 17 |  |  |  |  |  |  |  |  |  |  |  |  |
| 18 | 0 | 0 | 0 | 0 | 0 | 2 | 1 | 0 | 0 | 0 | 0 | 2 |
| 19 | 0 | 2 | 1 | 0 | 2 | 1 | 0 | 0 | 0 | 1 | 0 | 1 |
| 20 | 5 | 2 | 1 | 1 | 1 | 2 | 4 | 1 | 5 | 1 | 4 | 3 |
| 21 | 1 | 4 | 0 | 0 | 2 | 1 | 1 | 1 | 0 | 1 | 1 | 2 |
| 22 | 1 | 1 |  |  | 1 |  |  | 1 |  | 3 | 2 | 1 |
| 23 | 0 | 3 | 2 | 3 | 1 | 3 | 6 | 2 | 1 | 2 | 1 | 3 |
| 24 | 2 | 2 | 0 | 4 | 0 | 2 | 1 | 2 | 1 | 2 | 1 | 1 |
| 25 | 3 | 1 | 4 | 2 | 4 | 2 | 1 | 2 | 5 | 4 | 2 | 3 |
| 26 | 1 | 3 |  |  |  | 1 |  |  | 1 | 2 | 1 | 2 |
| 27 | 0 | 0 | 0 | 0 | 1 | 0 | 0 | 0 | 0 | 0 | 1 | 0 |
| 28 | 1 | 0 | 1 | 0 | 0 | 0 | 2 | 0 | 4 | 1 | 0 | 0 |
| 29 |  |  |  |  |  |  |  |  |  |  |  |  |
| 30 | 1 | 0 | 1 | 2 | 0 | 0 | 0 | 0 | 1 | 1 | 1 | 0 |
| 31 | 1 | 0 | 0 | 0 | 2 | 0 | 1 | 2 | 0 | 0 | 1 | 1 |
| 32 |  |  |  | 1 |  | 1 | 2 |  |  | 1 | 2 | 1 |
| 33 | 2 | 1 | 1 | 0 | 1 | 1 | 2 | 0 | 0 | 2 | 1 | 0 |
| 34 |  |  |  |  |  |  |  |  |  |  |  |  |
| 35 | 3 | 1 | 0 | 1 | 1 | 0 | 2 | 3 | 2 | 1 | 1 | 3 |
| 36 |  | 1 | 2 | 1 | 1 |  | 2 | 1 | 2 | 1 | 1 |  |
| 37 | 2 | 2 | 1 | 4 | 2 | 0 | 2 | 4 | 1 | 4 | 2 | 1 |
| 38 |  |  |  |  |  |  |  |  |  |  |  |  |
| 39 |  |  |  |  | 1 | 1 | 2 |  |  | 1 |  |  |
| 40 | 1 | 0 | 0 | 0 | 2 | 1 | 1 | 1 | 1 | 1 | 0 | 1 |
| 41 | 2 | 1 | 3 | 1 | 1 | 1 | 2 | 1 | 1 | 0 | 0 | 0 |
| 42 | 2 | 2 | 1 | 3 | 2 | 1 | 0 | 3 | 1 | 2 | 1 | 1 |
| 43 | 0 | 4 | 0 | 2 | 2 | 1 | 1 | 0 | 4 | 1 | 0 | 0 |
| 44 | 1 | 0 | 0 | 0 | 0 | 1 | 0 | 1 | 1 | 0 | 0 | 2 |
| 45 | 2 | 1 | 1 | 0 | 0 | 0 | 0 | 0 | 2 | 0 | 1 | 1 |
| 46 | 1 | 2 | 1 | 3 | 1 | 3 | 3 | 1 | 3 | 2 | 0 | 1 |
| 47 | 1 |  |  | 1 |  |  |  | 1 |  |  |  |  |
| 48 | 1 | 1 | 0 | 0 | 1 | 5 | 1 | 0 | 1 | 1 | 1 | 0 |
| 49 | 1 | 0 | 0 | 0 | 1 | 0 | 0 | 0 | 0 | 1 | 2 | 0 |
| 50 | 0 | 2 | 0 | 0 | 0 | 0 | 0 | 0 | 0 | 0 | 0 | 2 |
| 51 | 4 | 1 | 1 | 2 | 1 | 2 | 5 | 0 | 1 | 1 | 0 | 1 |
| 52 | 2 | 4 | 1 | 0 | 1 | 1 | 0 | 3 | 2 | 1 | 1 | 2 |
| 53 | 1 | 1 | 0 | 3 | 0 | 3 | 2 | 1 | 0 | 2 | 4 | 1 |
| 54 | 1 | 1 | 9 | 1 | 4 | 8 | 7 | 5 | 1 | 6 | 3 | 3 |
| 55 | 0 | 0 | 0 | 0 | 0 | 0 | 0 | 0 | 0 | 0 | 0 | 0 |
| 56 | 1 | 0 | 1 | 1 | 2 | 1 | 2 | 0 | 0 | 5 | 0 | 1 |
| 57 | 1 | 1 | 2 | 3 | 0 | 1 | 1 | 0 | 5 | 3 | 1 | 3 |
| 58 | 1 | 0 | 2 | 2 | 1 | 2 | 2 | 0 | 1 | 1 | 0 | 5 |
| 59 | 1 | 0 | 0 | 0 | 0 | 0 | 0 | 0 | 0 | 0 | 0 | 0 |
| 60 | 1 | 1 | 1 | 2 | 0 | 4 | 1 | 1 | 1 | 0 | 1 | 1 |
| 61 | 2 | 1 | 1 | 0 | 0 | 1 | 1 | 2 | 1 | 1 | 2 | 0 |
| 62 |  |  |  |  |  |  |  |  |  |  |  |  |
| 63 |  |  |  |  | 2 |  | 1 |  | 1 | 1 |  |  |
| 64 | 1 | 1 | 1 |  | 1 | 3 | 2 |  |  | 2 | 4 | 2 |
| 65 | 1 | 1 | 3 | 1 | 1 | 1 | 4 | 3 | 5 | 1 | 0 | 0 |
| 66 | 0 | 0 | 3 | 0 | 0 | 0 | 0 | 0 | 0 | 0 | 0 | 0 |
| 67 | 1 | 1 | 1 | 0 | 0 | 0 | 3 | 1 | 0 | 0 | 0 | 0 |
| 68 | 2 | 1 | 1 | 2 | 3 | 2 | 3 | 2 | 2 | 1 | 1 | 1 |
| 69 | 6 | 2 | 0 | 1 | 3 | 1 | 3 | 0 | 4 | 1 | 2 | 1 |
| 70 |  | 1 |  | 1 |  |  |  |  |  | 1 |  | 1 |
| 71 | 3 | 2 | 2 | 0 | 0 | 2 | 0 | 1 | 0 | 2 | 1 | 0 |
| 72 | 2 | 0 | 2 | 1 | 1 | 1 | 1 | 2 | 0 | 2 | 2 | 3 |
| 73 |  | 2 | 1 | 4 |  |  | 1 |  |  | 2 | 1 | 2 |
| 74 | 3 | 2 | 3 | 3 | 1 | 4 | 3 | 1 | 4 | 2 | 0 | 0 |
| 75 |  |  |  |  |  |  | 1 |  |  |  |  |  |
| 76 | 1 | 2 | 2 | 1 | 1 | 3 | 1 | 1 | 1 | 2 | 1 | 1 |
| 77 |  |  |  | 2 |  |  |  | 2 | 2 | 1 | 1 |  |
| 78 |  |  | 1 |  |  |  | 1 |  |  |  | 1 |  |
| 79 | 0 | 0 | 1 | 0 | 0 | 2 | 3 | 2 | 2 | 3 | 2 | 1 |
| 80 | 1 | 1 | 1 | 0 | 1 | 2 | 3 | 0 | 0 | 3 | 1 | 1 |
| 81 |  | 2 | 2 | 1 | 3 | 1 | 1 |  | 1 | 1 |  | 2 |
| 82 |  |  |  |  |  |  |  |  |  |  |  |  |
| 83 | 2 | 2 | 1 | 4 | 0 | 0 | 0 | 4 | 3 | 1 | 0 | 6 |
| 84 | 1 | 2 | 0 | 1 | 0 | 1 | 3 | 0 | 1 | 2 | 0 | 1 |
| 85 | 2 | 1 | 0 | 1 | 0 | 0 | 1 | 3 | 5 | 2 | 2 | 6 |
| 86 | 4 | 1 | 2 | 3 | 2 | 1 | 0 | 4 | 2 | 1 | 2 | 2 |
| 87 |  |  |  |  | 1 | 2 |  | 2 |  |  |  |  |
| 88 | 1 | 1 | 0 | 2 | 0 | 2 | 2 | 0 | 0 | 1 | 2 | 0 |
| 89 | 3 | 4 | 4 | 2 | 2 | 1 | 2 | 3 | 4 | 3 | 4 | 2 |
| 90 | 0 | 0 | 1 | 1 | 0 | 1 | 2 | 0 | 2 | 4 | 1 | 0 |
| 91 | 1 | 0 | 0 | 1 | 1 | 3 | 1 | 2 | 0 | 0 | 0 | 1 |
| 92 |  |  |  |  |  |  |  |  |  |  |  |  |
| 93 | 1 | 1 | 1 | 0 | 2 | 0 | 1 | 3 | 1 | 2 | 1 | 0 |
| 94 | 0 | 0 | 0 | 1 | 0 | 2 | 0 | 0 | 1 | 1 | 0 | 1 |
| 95 | 1 | 0 | 0 | 0 | 0 | 0 | 0 | 0 | 0 | 0 | 0 | 2 |
| 96 | 3 | 1 | 2 | 0 | 0 | 1 | 0 | 0 | 3 | 3 | 0 | 1 |
| 97 | 1 | 0 | 0 | 0 | 0 | 0 | 0 | 0 | 1 | 0 | 1 | 0 |
| 98 | 1 | 1 | 2 | 2 | 1 | 1 | 1 | 3 | 0 | 2 | 0 | 1 |
| 99 | 0 | 0 | 0 | 0 | 0 | 2 | 1 | 0 | 3 | 1 | 2 | 1 |
| 100 | 0 | 1 | 1 | 0 | 0 | 0 | 1 | 0 | 2 | 1 | 0 | 1 |
| 101 | 1 | 1 |  | 1 |  | 1 | 1 |  | 1 | 3 | 1 | 1 |
| 102 | 0 | 4 | 2 | 3 | 0 | 1 | 0 | 0 | 1 | 1 | 2 | 2 |
| 103 | 1 | 1 | 0 | 0 | 1 | 0 | 1 | 1 | 0 | 1 | 1 | 0 |
| 104 | 2 | 1 | 4 | 0 | 0 | 0 | 4 | 4 | 4 | 0 | 3 | 3 |
| 105 | 0 | 0 | 1 | 2 | 1 | 0 | 0 | 2 | 3 | 4 | 4 | 0 |

Table 7. Number of newly diagnosed stage III gastric cancer during each month in 2019.

|  | Month | | | | | | | | | | | |
| --- | --- | --- | --- | --- | --- | --- | --- | --- | --- | --- | --- | --- |
| Hospital | 1 | 2 | 3 | 4 | 5 | 6 | 7 | 8 | 9 | 10 | 11 | 12 |
| 1 | 0 | 0 | 0 | 1 | 0 | 0 | 0 | 0 | 0 | 0 | 0 | 0 |
| 2 | 12 | 3 | 5 | 4 | 5 | 6 | 5 | 3 | 8 | 12 | 2 | 5 |
| 3 | 0 | 0 | 1 | 1 | 2 | 0 | 1 | 1 | 1 | 0 | 1 | 1 |
| 4 | 1 | 1 | 1 | 2 | 2 | 6 | 2 | 0 | 1 | 2 | 4 | 1 |
| 5 | 0 | 0 | 0 | 1 | 0 | 1 | 1 | 0 | 2 | 1 | 3 | 0 |
| 6 | 0 | 1 | 2 | 1 | 1 | 2 | 1 | 1 | 2 | 4 | 0 | 2 |
| 7 | 1 | 0 | 1 | 2 | 1 | 2 | 1 | 5 | 1 | 1 | 0 | 3 |
| 8 | 1 | 1 | 0 | 0 | 0 | 0 | 0 | 1 | 1 | 0 | 0 | 0 |
| 9 | 1 | 1 | 1 | 2 | 0 | 2 | 2 | 1 | 0 | 1 | 2 | 2 |
| 10 | 1 | 2 | 3 |  | 1 |  | 3 |  |  | 2 | 1 | 1 |
| 11 | 1 | 1 | 1 | 4 | 2 | 1 | 1 | 1 | 2 | 1 | 1 | 0 |
| 12 | 0 | 0 | 0 | 0 | 0 | 1 | 1 | 1 | 0 | 0 | 0 | 0 |
| 13 | 1 | 2 | 1 | 4 | 2 | 1 | 1 | 0 | 0 | 4 | 0 | 1 |
| 14 | 3 | 0 | 1 | 2 | 2 | 3 | 1 | 1 | 2 | 1 | 1 | 2 |
| 15 | 1 | 0 | 0 | 3 | 0 | 3 | 1 | 1 | 1 | 1 | 2 | 0 |
| 16 | 1 | 5 | 0 | 2 | 3 | 1 | 4 | 1 | 0 | 0 | 3 | 1 |
| 17 |  |  |  |  |  |  |  |  |  |  |  |  |
| 18 | 0 | 2 | 2 | 1 | 1 | 0 | 1 | 2 | 2 | 0 | 0 | 0 |
| 19 | 2 | 1 | 0 | 0 | 0 | 1 | 1 | 0 | 0 | 0 | 0 | 0 |
| 20 | 7 | 3 | 4 | 1 | 3 | 4 | 4 | 2 | 4 | 4 | 5 | 4 |
| 21 | 1 | 2 | 0 | 4 | 2 | 2 | 3 | 2 | 2 | 2 | 1 | 0 |
| 22 |  |  | 1 | 1 | 1 |  | 1 |  | 2 |  | 1 | 2 |
| 23 | 7 | 2 | 5 | 5 | 5 | 9 | 9 | 3 | 8 | 4 | 3 | 2 |
| 24 | 1 | 3 | 2 | 3 | 1 | 1 | 4 | 1 | 2 | 5 | 2 | 1 |
| 25 | 2 | 4 | 3 | 6 | 1 | 3 | 4 | 4 | 1 | 4 | 3 | 4 |
| 26 | 1 | 1 |  | 1 | 1 | 3 | 1 | 2 | 3 |  |  |  |
| 27 | 1 | 0 | 2 | 1 | 1 | 0 | 2 | 0 | 0 | 2 | 1 | 0 |
| 28 | 3 | 1 | 0 | 5 | 2 | 1 | 0 | 0 | 2 | 3 | 0 | 1 |
| 29 |  |  |  |  |  |  |  |  |  |  |  |  |
| 30 | 0 | 1 | 2 | 1 | 0 | 0 | 2 | 0 | 0 | 1 | 0 | 0 |
| 31 | 1 | 2 | 0 | 1 | 1 | 0 | 1 | 0 | 1 | 1 | 2 | 0 |
| 32 |  | 1 |  | 1 |  |  |  |  | 1 | 1 |  | 2 |
| 33 | 2 | 1 | 1 | 1 | 1 | 1 | 2 | 2 | 1 | 3 | 2 | 2 |
| 34 |  |  |  |  |  |  |  |  |  |  |  |  |
| 35 | 3 | 1 | 2 | 1 | 1 | 1 | 2 | 2 | 3 | 0 | 0 | 1 |
| 36 |  |  |  | 1 |  |  | 2 | 3 |  |  |  |  |
| 37 | 4 | 5 | 2 | 2 | 2 | 3 | 3 | 1 | 4 | 2 | 1 | 3 |
| 38 |  |  |  |  |  |  |  |  |  |  |  |  |
| 39 |  |  |  |  |  | 1 |  |  |  |  |  | 1 |
| 40 | 0 | 2 | 1 | 0 | 0 | 0 | 0 | 0 | 0 | 0 | 1 | 0 |
| 41 | 1 | 0 | 1 | 0 | 1 | 1 | 2 | 1 | 0 | 1 | 1 | 1 |
| 42 | 0 | 1 | 1 | 1 | 1 | 1 | 1 | 1 | 9 | 0 | 1 | 1 |
| 43 | 0 | 2 | 0 | 0 | 3 | 1 | 1 | 2 | 2 | 3 | 0 | 0 |
| 44 | 0 | 0 | 0 | 0 | 0 | 0 | 0 | 0 | 0 | 2 | 0 | 1 |
| 45 | 2 | 2 | 1 | 1 | 0 | 0 | 1 | 0 | 1 | 4 | 3 | 0 |
| 46 | 1 | 1 | 0 | 1 | 2 | 3 | 3 | 1 | 0 | 3 | 1 | 1 |
| 47 |  |  |  |  | 1 |  |  |  |  | 1 |  |  |
| 48 | 1 | 3 | 1 | 4 | 1 | 0 | 5 | 2 | 1 | 0 | 0 | 1 |
| 49 | 0 | 2 | 0 | 0 | 1 | 0 | 1 | 1 | 2 | 2 | 5 | 2 |
| 50 | 0 | 0 | 0 | 2 | 0 | 2 | 0 | 0 | 2 | 1 | 0 | 0 |
| 51 | 2 | 1 | 4 | 3 | 4 | 3 | 3 | 3 | 1 | 5 | 1 | 5 |
| 52 | 1 | 0 | 2 | 5 | 4 | 1 | 4 | 2 | 2 | 2 | 1 | 0 |
| 53 | 0 | 2 | 0 | 2 | 2 | 3 | 3 | 2 | 0 | 2 | 3 | 0 |
| 54 | 5 | 9 | 8 | 6 | 4 | 6 | 5 | 6 | 8 | 7 | 7 | 2 |
| 55 | 2 | 2 | 0 | 2 | 0 | 0 | 0 | 0 | 1 | 1 | 0 | 2 |
| 56 | 1 | 4 | 3 | 2 | 0 | 0 | 0 | 1 | 0 | 0 | 0 | 1 |
| 57 | 0 | 3 | 3 | 2 | 1 | 3 | 3 | 4 | 3 | 1 | 2 | 0 |
| 58 | 1 | 1 | 1 | 1 | 2 | 0 | 0 | 1 | 2 | 2 | 0 | 1 |
| 59 | 0 | 3 | 2 | 1 | 0 | 0 | 1 | 1 | 0 | 0 | 0 | 2 |
| 60 | 1 | 1 | 1 | 3 | 0 | 2 | 1 | 4 | 0 | 0 | 3 | 2 |
| 61 | 0 | 0 | 0 | 1 | 0 | 1 | 1 | 2 | 0 | 4 | 0 | 0 |
| 62 |  |  |  |  |  |  |  |  |  |  |  |  |
| 63 |  |  |  |  | 1 |  |  |  | 1 |  |  |  |
| 64 |  |  | 3 | 2 | 2 | 2 | 1 | 2 | 1 | 2 | 3 | 1 |
| 65 | 0 | 3 | 2 | 3 | 5 | 2 | 4 | 2 | 2 | 1 | 1 | 1 |
| 66 | 0 | 0 | 0 | 0 | 0 | 0 | 0 | 0 | 0 | 0 | 0 | 0 |
| 67 | 3 | 0 | 1 | 2 | 1 | 0 | 0 | 1 | 0 | 2 | 2 | 0 |
| 68 | 0 | 0 | 3 | 1 | 0 | 2 | 2 | 1 | 0 | 2 | 1 | 0 |
| 69 | 1 | 0 | 1 | 4 | 4 | 2 | 4 | 6 | 1 | 1 | 2 | 4 |
| 70 |  |  |  |  |  | 1 |  |  |  |  |  |  |
| 71 | 1 | 0 | 3 | 2 | 1 | 3 | 3 | 3 | 1 | 0 | 1 | 1 |
| 72 | 0 | 5 | 1 | 1 | 2 | 3 | 2 | 2 | 3 | 2 | 1 | 0 |
| 73 |  | 2 | 1 | 1 | 2 |  |  | 2 | 1 | 2 | 2 | 2 |
| 74 | 0 | 2 | 0 | 2 | 4 | 1 | 1 | 3 | 1 | 1 | 1 | 4 |
| 75 |  |  |  |  |  |  |  | 1 |  |  |  |  |
| 76 | 1 | 2 | 5 | 2 | 0 | 2 | 2 | 3 | 3 | 1 | 2 | 1 |
| 77 |  |  |  |  | 1 | 1 |  |  |  | 1 | 1 | 1 |
| 78 |  |  |  |  |  |  |  |  |  |  |  | 1 |
| 79 | 3 | 1 | 1 | 2 | 0 | 2 | 3 | 1 | 1 | 0 | 1 | 1 |
| 80 | 3 | 3 | 1 | 1 | 5 | 2 | 4 | 4 | 1 | 2 | 1 | 4 |
| 81 | 1 |  | 1 |  | 1 | 2 | 4 | 2 |  | 4 | 1 | 1 |
| 82 |  |  |  |  |  |  |  |  |  |  |  |  |
| 83 | 1 | 1 | 2 | 3 | 3 | 3 | 2 | 2 | 4 | 2 | 5 | 1 |
| 84 | 0 | 1 | 1 | 0 | 2 | 1 | 3 | 0 | 3 | 2 | 1 | 2 |
| 85 | 0 | 2 | 1 | 1 | 1 | 0 | 3 | 2 | 4 | 1 | 2 | 0 |
| 86 | 0 | 1 | 1 | 0 | 1 | 3 | 0 | 4 | 1 | 1 | 2 | 1 |
| 87 | 1 | 1 | 1 | 2 | 1 |  | 1 |  |  |  |  |  |
| 88 | 0 | 0 | 1 | 2 | 1 | 3 | 2 | 2 | 2 | 1 | 0 | 0 |
| 89 | 5 | 2 | 5 | 6 | 4 | 1 | 6 | 3 | 3 | 1 | 5 | 5 |
| 90 | 2 | 0 | 0 | 1 | 0 | 0 | 0 | 0 | 2 | 0 | 1 | 0 |
| 91 | 0 | 0 | 1 | 1 | 1 | 1 | 2 | 1 | 1 | 1 | 2 | 1 |
| 92 |  |  |  |  |  |  |  |  |  |  |  |  |
| 93 | 0 | 0 | 1 | 0 | 1 | 0 | 0 | 2 | 0 | 1 | 1 | 0 |
| 94 | 0 | 0 | 0 | 1 | 0 | 0 | 1 | 1 | 0 | 1 | 0 | 0 |
| 95 | 1 | 1 | 0 | 0 | 0 | 0 | 2 | 0 | 0 | 0 | 0 | 1 |
| 96 | 1 | 1 | 0 | 1 | 1 | 2 | 0 | 2 | 2 | 1 | 1 | 1 |
| 97 | 1 | 1 | 1 | 0 | 0 | 0 | 0 | 0 | 0 | 0 | 0 | 0 |
| 98 | 4 | 0 | 2 | 1 | 0 | 0 | 1 | 0 | 2 | 2 | 2 | 3 |
| 99 | 1 | 1 | 1 | 2 | 2 | 0 | 1 | 0 | 0 | 0 | 2 | 0 |
| 100 | 1 | 0 | 1 | 0 | 0 | 1 | 1 | 2 | 1 | 1 | 0 | 0 |
| 101 | 1 |  | 2 | 1 | 1 | 1 | 1 | 1 | 2 |  | 1 | 1 |
| 102 | 0 | 1 | 4 | 0 | 2 | 5 | 1 | 2 | 1 | 0 | 3 | 2 |
| 103 | 1 | 2 | 0 | 1 | 1 | 2 | 1 | 3 | 0 | 0 | 1 | 1 |
| 104 | 3 | 0 | 1 | 2 | 2 | 2 | 3 | 2 | 1 | 6 | 2 | 2 |
| 105 | 2 | 0 | 0 | 2 | 4 | 0 | 1 | 0 | 2 | 1 | 1 | 0 |

Table 8. Number of newly diagnosed stage III gastric cancer during each month in 2020.

|  | Month | | | | | | | | | | | |
| --- | --- | --- | --- | --- | --- | --- | --- | --- | --- | --- | --- | --- |
| Hospital | 1 | 2 | 3 | 4 | 5 | 6 | 7 | 8 | 9 | 10 | 11 | 12 |
| 1 | 1 | 0 | 0 | 1 | 0 | 1 | 1 | 0 | 0 | 0 | 1 | 0 |
| 2 | 8 | 11 | 3 | 4 | 3 | 2 | 7 | 5 | 4 | 7 | 7 | 4 |
| 3 | 0 | 1 | 1 | 2 | 1 | 4 | 0 | 2 | 2 | 1 | 2 | 2 |
| 4 | 2 | 0 | 2 | 0 | 1 | 0 | 1 | 0 | 1 | 2 | 1 | 0 |
| 5 | 0 | 0 | 1 | 1 | 0 | 0 | 0 | 0 | 1 | 2 | 3 | 3 |
| 6 | 0 | 0 | 1 | 2 | 0 | 4 | 0 | 0 | 3 | 3 | 0 | 2 |
| 7 | 1 | 0 | 0 | 1 | 0 | 1 | 0 | 3 | 0 | 2 | 0 | 2 |
| 8 | 0 | 1 | 0 | 1 | 1 | 1 | 0 | 2 | 1 | 0 | 0 | 0 |
| 9 | 0 | 0 | 0 | 2 | 1 | 1 | 1 | 0 | 3 | 2 | 1 | 0 |
| 10 |  | 3 | 1 | 2 | 1 | 1 | 1 | 2 | 2 | 2 |  | 1 |
| 11 | 1 | 0 | 1 | 1 | 2 | 4 | 2 | 3 | 2 | 3 | 2 | 1 |
| 12 | 2 | 2 | 1 | 0 | 1 | 1 | 1 | 1 | 1 | 1 | 0 | 0 |
| 13 | 4 | 1 | 2 | 2 | 1 | 1 | 2 | 1 | 5 | 1 | 3 | 1 |
| 14 | 2 | 1 | 1 | 0 | 3 | 0 | 0 | 2 | 0 | 3 | 0 | 1 |
| 15 | 1 | 0 | 2 | 1 | 0 | 4 | 0 | 1 | 1 | 1 | 4 | 2 |
| 16 | 0 | 1 | 3 | 3 | 4 | 2 | 2 | 2 | 0 | 2 | 4 | 1 |
| 17 |  |  |  |  |  | 1 |  |  |  |  |  |  |
| 18 | 0 | 0 | 1 | 2 | 0 | 1 | 1 | 2 | 1 | 1 | 1 | 0 |
| 19 | 1 | 0 | 2 | 3 | 0 | 0 | 1 | 1 | 3 | 0 | 1 | 0 |
| 20 | 5 | 2 | 3 | 1 | 1 | 5 | 2 | 3 | 4 | 2 | 6 | 6 |
| 21 | 1 | 2 | 1 | 1 | 0 | 1 | 3 | 0 | 1 | 0 | 0 | 1 |
| 22 |  | 1 | 1 |  |  | 3 | 2 | 1 |  | 2 | 1 |  |
| 23 | 0 | 8 | 10 | 3 | 4 | 6 | 5 | 11 | 6 | 7 | 4 | 4 |
| 24 | 0 | 1 | 2 | 0 | 2 | 4 | 2 | 1 | 5 | 3 | 3 | 2 |
| 25 | 1 | 0 | 2 | 2 | 1 | 4 | 1 | 1 | 3 | 3 | 2 | 4 |
| 26 | 1 | 1 | 1 |  | 2 |  | 2 | 2 | 1 | 1 | 1 | 2 |
| 27 | 1 | 0 | 1 | 0 | 2 | 1 | 1 | 1 | 0 | 0 | 2 | 1 |
| 28 | 1 | 0 | 1 | 0 | 0 | 1 | 1 | 1 | 2 | 1 | 0 | 0 |
| 29 |  |  |  |  |  |  |  |  |  |  |  |  |
| 30 | 2 | 0 | 0 | 1 | 0 | 1 | 1 | 2 | 0 | 0 | 0 | 1 |
| 31 | 0 | 1 | 2 | 1 | 0 | 0 | 1 | 1 | 1 | 2 | 3 | 2 |
| 32 | 1 |  |  |  | 1 |  | 1 | 1 | 1 | 1 |  | 1 |
| 33 | 2 |  | 3 |  | 1 |  | 2 |  |  | 2 |  | 1 |
| 34 |  |  |  |  |  |  |  |  |  |  |  |  |
| 35 | 2 | 4 | 0 | 1 | 2 | 3 | 1 | 3 | 2 | 2 | 0 | 0 |
| 36 | 3 |  |  |  |  |  | 2 |  | 1 | 1 | 1 |  |
| 37 | 0 | 3 | 0 | 1 | 0 | 4 | 6 | 4 | 0 | 3 | 4 | 3 |
| 38 |  |  |  |  |  |  |  |  |  |  |  |  |
| 39 |  |  |  | 1 | 1 |  |  |  | 1 |  |  |  |
| 40 | 3 | 0 | 0 | 1 | 1 | 1 | 2 | 0 | 2 | 1 | 1 | 0 |
| 41 | 4 | 2 | 0 | 1 | 2 | 0 | 1 | 1 | 1 | 1 | 0 | 2 |
| 42 | 0 | 1 | 1 | 0 | 2 | 1 | 2 | 3 | 0 | 1 | 1 | 3 |
| 43 | 2 | 0 | 1 | 1 | 0 | 0 | 3 | 1 | 0 | 0 | 1 | 1 |
| 44 | 0 | 0 | 0 | 1 | 0 | 1 | 0 | 0 | 0 | 0 | 0 | 1 |
| 45 | 0 | 1 | 1 | 3 | 0 | 0 | 1 | 2 | 2 | 1 | 1 | 1 |
| 46 | 0 | 3 | 1 | 1 | 3 | 3 | 4 | 1 | 3 | 1 | 2 | 2 |
| 47 |  |  |  |  |  |  |  |  |  |  |  |  |
| 48 | 3 | 1 | 2 | 0 | 1 | 2 | 2 | 2 | 1 | 1 | 0 | 1 |
| 49 | 2 | 0 | 0 | 0 | 0 | 1 | 1 | 1 | 4 | 1 | 1 | 0 |
| 50 | 0 | 0 | 1 | 0 | 0 | 0 | 0 | 0 | 1 | 1 | 0 | 0 |
| 51 | 2 | 0 | 0 | 2 | 1 | 4 | 1 | 2 | 1 | 2 | 3 | 4 |
| 52 | 1 | 1 | 1 | 4 | 1 | 1 | 1 | 0 | 3 | 3 | 2 | 1 |
| 53 | 3 | 1 | 1 | 1 | 1 | 0 | 0 | 2 | 2 | 1 | 0 | 1 |
| 54 | 2 | 7 | 6 | 2 | 2 | 4 | 6 | 3 | 1 | 3 | 4 | 4 |
| 55 | 0 | 1 | 1 | 0 | 1 | 1 | 0 | 3 | 2 | 0 | 1 | 0 |
| 56 | 4 | 0 | 2 | 3 | 1 | 1 | 0 | 3 | 0 | 1 | 1 | 1 |
| 57 | 1 | 1 | 3 | 2 | 1 | 1 | 0 | 1 | 2 | 4 | 3 | 1 |
| 58 | 2 | 0 | 1 | 0 | 1 | 0 | 1 | 2 | 0 | 1 | 2 | 1 |
| 59 | 0 | 1 | 0 | 0 | 0 | 2 | 0 | 2 | 0 | 1 | 1 | 0 |
| 60 | 1 | 0 | 2 | 2 | 1 | 0 | 0 | 0 | 1 | 1 | 1 | 0 |
| 61 | 0 | 0 | 0 | 1 | 1 | 1 | 3 | 1 | 0 | 4 | 1 | 2 |
| 62 |  |  |  |  |  |  |  |  |  |  |  |  |
| 63 | 1 |  | 1 |  | 2 |  |  | 1 | 3 |  | 1 |  |
| 64 | 2 | 2 | 3 | 4 | 1 | 3 |  | 2 | 3 | 5 | 3 | 2 |
| 65 | 5 | 5 | 5 | 1 | 3 | 1 | 3 | 1 | 2 | 3 | 2 | 2 |
| 66 | 0 | 0 | 0 | 0 | 0 | 1 | 2 | 0 | 0 | 0 | 0 | 0 |
| 67 | 0 | 0 | 0 | 1 | 1 | 0 | 1 | 2 | 2 | 0 | 1 | 1 |
| 68 | 1 | 1 | 0 | 0 | 1 | 2 | 4 | 2 | 1 | 1 | 0 | 2 |
| 69 | 5 | 7 | 2 | 7 | 3 | 2 | 2 | 7 | 4 | 1 | 4 | 6 |
| 70 | 1 |  |  |  |  |  |  |  |  |  |  | 1 |
| 71 | 2 | 0 | 1 | 2 | 0 | 0 | 2 | 1 | 1 | 2 | 0 | 1 |
| 72 | 2 | 1 | 0 | 2 | 1 | 3 | 3 | 0 | 1 | 3 | 3 | 0 |
| 73 |  | 1 | 1 | 2 |  |  |  | 1 | 1 |  | 2 | 1 |
| 74 | 0 | 1 | 1 | 2 | 2 | 0 | 0 | 1 | 3 | 0 | 2 | 2 |
| 75 |  |  |  |  |  |  |  |  |  |  |  |  |
| 76 | 3 | 2 | 0 | 4 | 0 | 1 | 0 | 1 | 2 | 1 | 2 | 0 |
| 77 | 1 |  |  |  |  |  |  |  |  |  |  |  |
| 78 | 1 |  |  |  |  |  |  |  |  |  |  |  |
| 79 | 3 | 1 | 2 | 3 | 2 | 1 | 2 | 1 | 1 | 1 | 4 | 4 |
| 80 | 2 | 3 | 0 | 2 | 0 | 0 | 2 | 1 | 1 | 2 | 1 | 0 |
| 81 | 3 |  | 2 | 2 | 1 | 1 | 3 | 1 |  | 1 |  | 1 |
| 82 |  |  |  |  |  |  |  |  |  |  |  |  |
| 83 | 0 | 1 | 2 | 1 | 0 | 1 | 3 | 2 | 2 | 4 | 5 | 2 |
| 84 | 1 | 3 | 1 | 0 | 2 | 2 | 1 | 2 | 2 | 0 | 4 | 1 |
| 85 | 1 | 1 | 1 | 0 | 2 | 1 | 0 | 0 | 1 | 0 | 3 | 2 |
| 86 | 2 | 3 | 2 | 0 | 2 | 0 | 1 | 1 | 1 | 0 | 3 | 0 |
| 87 | 1 |  | 1 | 1 | 2 | 2 | 2 | 2 | 1 |  | 2 | 2 |
| 88 | 1 | 2 | 2 | 2 | 1 | 1 | 0 | 0 | 0 | 4 | 0 | 2 |
| 89 | 6 | 9 | 4 | 5 | 2 | 5 | 6 | 4 | 4 | 2 | 4 | 8 |
| 90 | 2 | 1 | 5 | 0 | 0 | 1 | 0 | 0 | 1 | 0 | 1 | 1 |
| 91 | 0 | 0 | 0 | 0 | 1 | 3 | 2 | 0 | 2 | 1 | 0 | 0 |
| 92 |  |  |  |  |  |  |  |  |  |  |  |  |
| 93 | 2 | 0 | 1 | 0 | 2 | 2 | 2 | 0 | 0 | 0 | 0 | 0 |
| 94 | 1 | 0 | 0 | 0 | 0 | 1 | 1 | 1 | 0 | 0 | 1 | 1 |
| 95 | 0 | 0 | 0 | 2 | 1 | 0 | 2 | 1 | 1 | 0 | 0 | 1 |
| 96 | 2 | 1 | 1 | 1 | 2 | 0 | 0 | 2 | 0 | 0 | 2 | 2 |
| 97 | 1 | 0 | 0 | 0 | 0 | 0 | 0 | 0 | 0 | 0 | 0 | 0 |
| 98 | 1 | 0 | 1 | 0 | 1 | 0 | 2 | 0 | 1 | 0 | 0 | 3 |
| 99 | 2 | 0 | 0 | 0 | 0 | 1 | 0 | 1 | 1 | 1 | 0 | 0 |
| 100 | 0 | 1 | 0 | 1 | 2 | 2 | 1 | 1 | 0 | 2 | 0 | 0 |
| 101 | 2 | 2 | 3 | 2 |  | 1 |  |  | 3 |  |  | 1 |
| 102 | 0 | 1 | 2 | 0 | 0 | 2 | 2 | 0 | 0 | 5 | 1 | 1 |
| 103 | 0 | 1 | 2 | 0 | 2 | 1 | 0 | 1 | 1 | 2 | 2 | 2 |
| 104 | 1 | 1 | 1 | 1 | 1 | 3 | 0 | 2 | 1 | 1 | 2 | 3 |
| 105 | 1 | 1 | 1 | 0 | 1 | 1 | 2 | 0 | 3 | 2 | 1 | 2 |

Table 9. Number of newly diagnosed stage IV gastric cancer during each month in 2019.

|  | Month | | | | | | | | | | | |
| --- | --- | --- | --- | --- | --- | --- | --- | --- | --- | --- | --- | --- |
| Hospital | 1 | 2 | 3 | 4 | 5 | 6 | 7 | 8 | 9 | 10 | 11 | 12 |
| 1 | 0 | 2 | 2 | 0 | 1 | 2 | 1 | 1 | 1 | 1 | 0 | 1 |
| 2 | 10 | 15 | 11 | 8 | 5 | 10 | 12 | 14 | 16 | 13 | 10 | 14 |
| 3 | 2 | 2 | 2 | 0 | 1 | 1 | 3 | 1 | 2 | 1 | 1 | 3 |
| 4 | 5 | 4 | 8 | 7 | 3 | 3 | 4 | 5 | 2 | 7 | 7 | 7 |
| 5 | 0 | 3 | 0 | 0 | 1 | 2 | 5 | 2 | 3 | 2 | 1 | 0 |
| 6 | 4 | 4 | 3 | 3 | 2 | 6 | 1 | 3 | 4 | 7 | 2 | 0 |
| 7 | 3 | 5 | 1 | 6 | 3 | 4 | 3 | 0 | 2 | 1 | 4 | 1 |
| 8 | 0 | 0 | 0 | 0 | 2 | 2 | 1 | 1 | 0 | 0 | 1 | 0 |
| 9 | 5 | 7 | 5 | 4 | 3 | 1 | 2 | 4 | 4 | 1 | 2 | 3 |
| 10 | 5 | 1 | 2 | 2 | 1 | 2 | 3 | 3 | 3 | 6 | 3 | 7 |
| 11 | 2 | 5 | 4 | 4 | 3 | 2 | 5 | 2 | 4 | 1 | 3 | 4 |
| 12 | 3 | 2 | 0 | 0 | 1 | 1 | 1 | 2 | 1 | 3 | 2 | 0 |
| 13 | 2 | 8 | 2 | 2 | 2 | 2 | 3 | 1 | 0 | 7 | 2 | 2 |
| 14 | 2 | 3 | 0 | 4 | 2 | 1 | 3 | 1 | 1 | 4 | 3 | 5 |
| 15 | 4 | 3 | 2 | 4 | 3 | 3 | 5 | 4 | 5 | 4 | 5 | 1 |
| 16 | 6 | 2 | 6 | 9 | 3 | 3 | 7 | 3 | 7 | 7 | 7 | 7 |
| 17 |  |  |  |  |  | 1 |  |  |  |  |  |  |
| 18 | 0 | 2 | 1 | 1 | 1 | 0 | 1 | 0 | 0 | 3 | 1 | 2 |
| 19 | 1 | 2 | 1 | 1 | 2 | 1 | 4 | 1 | 5 | 1 | 2 | 1 |
| 20 | 9 | 8 | 5 | 6 | 9 | 7 | 3 | 9 | 6 | 7 | 5 | 11 |
| 21 | 4 | 2 | 2 | 4 | 2 | 1 | 5 | 6 | 3 | 3 | 1 | 2 |
| 22 | 2 |  | 1 | 1 | 3 | 5 | 2 | 1 | 3 | 1 | 1 | 2 |
| 23 | 4 | 4 | 4 | 6 | 7 | 3 | 5 | 9 | 9 | 5 | 5 | 8 |
| 24 | 5 | 4 | 4 | 6 | 3 | 5 | 2 | 4 | 1 | 3 | 3 | 1 |
| 25 | 4 | 0 | 8 | 7 | 4 | 2 | 6 | 7 | 3 | 3 | 2 | 4 |
| 26 | 4 | 2 | 2 | 5 | 2 | 4 | 1 | 1 | 3 | 2 | 4 |  |
| 27 | 0 | 3 | 0 | 5 | 1 | 1 | 6 | 1 | 5 | 1 | 1 | 2 |
| 28 | 2 | 2 | 1 | 4 | 3 | 3 | 0 | 2 | 1 | 1 | 1 | 2 |
| 29 |  |  |  |  |  |  |  |  |  |  |  |  |
| 30 | 1 | 3 | 0 | 1 | 1 | 2 | 3 | 1 | 3 | 3 | 2 | 2 |
| 31 | 2 | 4 | 4 | 2 | 3 | 2 | 2 | 3 | 4 | 2 | 3 | 0 |
| 32 | 1 | 3 | 2 | 2 | 2 |  | 4 | 2 | 2 |  |  | 1 |
| 33 |  | 2 | 3 | 1 | 3 | 3 | 1 | 2 | 1 | 1 | 2 |  |
| 34 |  |  |  |  |  |  |  |  |  |  |  |  |
| 35 | 3 | 5 | 3 | 2 | 4 | 3 | 6 | 2 | 3 | 1 | 4 | 4 |
| 36 | 1 | 1 | 1 | 2 |  |  |  |  | 1 |  | 2 | 2 |
| 37 | 4 | 4 | 3 | 1 | 5 | 3 | 4 | 4 | 3 | 1 | 4 | 8 |
| 38 |  |  |  |  |  |  |  |  |  |  |  |  |
| 39 |  |  | 1 | 3 |  |  |  | 1 | 3 |  | 2 | 1 |
| 40 | 0 | 2 | 4 | 1 | 1 | 1 | 6 | 2 | 2 | 2 | 0 | 8 |
| 41 | 2 | 3 | 2 | 8 | 2 | 1 | 5 | 4 | 2 | 2 | 4 | 1 |
| 42 | 1 | 3 | 0 | 2 | 2 | 2 | 2 | 4 | 2 | 0 | 1 | 4 |
| 43 | 4 | 1 | 2 | 4 | 1 | 3 | 2 | 1 | 3 | 1 | 3 | 1 |
| 44 | 4 | 1 | 1 | 0 | 2 | 3 | 3 | 2 | 0 | 0 | 0 | 0 |
| 45 | 1 | 3 | 2 | 4 | 5 | 0 | 1 | 2 | 1 | 2 | 2 | 0 |
| 46 | 3 | 0 | 2 | 1 | 8 | 1 | 3 | 2 | 6 | 2 | 2 | 2 |
| 47 | 1 |  |  |  | 1 |  |  |  |  |  |  |  |
| 48 | 1 | 3 | 2 | 4 | 5 | 5 | 6 | 2 | 3 | 3 | 3 | 3 |
| 49 | 2 | 0 | 0 | 1 | 5 | 0 | 1 | 0 | 1 | 1 | 1 | 1 |
| 50 | 1 | 2 | 1 | 1 | 2 | 0 | 1 | 0 | 1 | 0 | 0 | 0 |
| 51 | 3 | 1 | 4 | 3 | 3 | 3 | 3 | 4 | 5 | 4 | 6 | 2 |
| 52 | 2 | 1 | 6 | 3 | 0 | 4 | 3 | 1 | 3 | 5 | 5 | 0 |
| 53 | 16 | 3 | 1 | 1 | 3 | 4 | 6 | 3 | 2 | 8 | 3 | 2 |
| 54 | 7 | 8 | 10 | 7 | 16 | 11 | 11 | 13 | 9 | 12 | 14 | 11 |
| 55 | 1 | 3 | 1 | 3 | 0 | 3 | 2 | 2 | 2 | 2 | 4 | 1 |
| 56 | 1 | 1 | 0 | 3 | 3 | 2 | 4 | 2 | 2 | 1 | 1 | 3 |
| 57 | 10 | 7 | 7 | 3 | 7 | 8 | 5 | 5 | 3 | 3 | 0 | 7 |
| 58 | 1 | 1 | 3 | 1 | 1 | 3 | 3 | 2 | 1 | 2 | 0 | 1 |
| 59 | 2 | 2 | 1 | 0 | 3 | 3 | 2 | 2 | 1 | 3 | 1 | 0 |
| 60 | 3 | 2 | 1 | 2 | 4 | 2 | 0 | 2 | 3 | 2 | 2 | 2 |
| 61 | 8 | 2 | 0 | 5 | 4 | 3 | 4 | 1 | 1 | 5 | 3 | 4 |
| 62 |  |  |  |  |  |  |  |  |  |  |  |  |
| 63 | 2 | 3 | 2 | 1 | 1 | 3 |  | 1 | 2 | 1 |  | 2 |
| 64 | 2 | 6 | 1 | 3 | 3 | 3 | 2 | 2 |  | 2 | 3 | 6 |
| 65 | 5 | 2 | 4 | 3 | 7 | 2 | 6 | 5 | 2 | 4 | 3 | 2 |
| 66 | 0 | 0 | 0 | 0 | 0 | 0 | 0 | 1 | 1 | 1 | 0 | 0 |
| 67 | 5 | 5 | 1 | 2 | 3 | 1 | 2 | 2 | 4 | 2 | 2 | 2 |
| 68 | 6 | 4 | 2 | 3 | 7 | 5 | 2 | 2 | 4 | 7 | 3 | 5 |
| 69 | 5 | 3 | 6 | 5 | 4 | 3 | 2 | 1 | 3 | 7 | 3 | 5 |
| 70 |  |  | 1 | 2 | 1 |  | 2 | 2 |  | 1 |  | 1 |
| 71 | 3 | 1 | 3 | 5 | 1 | 5 | 5 | 3 | 2 | 2 | 1 | 5 |
| 72 | 0 | 5 | 4 | 1 | 4 | 5 | 2 | 3 | 1 | 3 | 5 | 2 |
| 73 | 3 | 1 | 1 | 2 |  | 4 | 2 |  | 1 | 1 |  | 2 |
| 74 | 4 | 1 | 4 | 2 | 4 | 5 | 5 | 1 | 1 | 2 | 6 | 3 |
| 75 |  |  |  |  |  |  |  |  |  |  |  |  |
| 76 | 2 | 1 | 3 | 1 | 4 | 3 | 0 | 3 | 4 | 1 | 4 | 0 |
| 77 | 1 |  | 2 |  |  |  |  |  |  | 1 | 1 | 1 |
| 78 |  | 1 |  | 1 |  | 1 | 2 |  |  | 1 | 1 |  |
| 79 | 3 | 3 | 2 | 6 | 1 | 6 | 1 | 4 | 4 | 3 | 3 | 1 |
| 80 | 1 | 1 | 1 | 2 | 5 | 4 | 5 | 0 | 3 | 0 | 2 | 3 |
| 81 |  | 1 | 1 | 2 | 1 | 2 | 1 | 2 | 1 | 3 | 2 | 1 |
| 82 |  |  |  |  |  |  |  |  |  |  |  |  |
| 83 | 2 | 0 | 4 | 1 | 1 | 1 | 5 | 3 | 3 | 3 | 1 | 3 |
| 84 | 5 | 2 | 3 | 3 | 3 | 2 | 4 | 2 | 1 | 2 | 3 | 6 |
| 85 | 3 | 3 | 1 | 3 | 7 | 4 | 2 | 0 | 1 | 4 | 3 | 1 |
| 86 | 4 | 2 | 2 | 4 | 7 | 3 | 1 | 1 | 3 | 2 | 1 | 2 |
| 87 | 4 |  | 2 | 2 | 1 | 2 | 4 | 3 | 3 | 4 |  | 2 |
| 88 | 1 | 0 | 2 | 2 | 1 | 3 | 4 | 0 | 2 | 0 | 2 | 0 |
| 89 | 13 | 8 | 11 | 7 | 14 | 17 | 9 | 9 | 11 | 14 | 10 | 14 |
| 90 | 1 | 6 | 4 | 3 | 0 | 5 | 5 | 1 | 3 | 5 | 4 | 3 |
| 91 | 5 | 3 | 3 | 3 | 4 | 6 | 2 | 4 | 1 | 3 | 0 | 3 |
| 92 |  |  |  |  |  |  |  |  |  |  |  |  |
| 93 | 1 | 2 | 2 | 0 | 3 | 1 | 2 | 0 | 1 | 2 | 3 | 3 |
| 94 | 1 | 2 | 3 | 3 | 0 | 4 | 0 | 5 | 4 | 2 | 2 | 1 |
| 95 | 2 | 4 | 2 | 5 | 1 | 1 | 1 | 2 | 1 | 0 | 0 | 1 |
| 96 | 2 | 2 | 3 | 1 | 0 | 2 | 1 | 0 | 0 | 1 | 0 | 1 |
| 97 | 0 | 1 | 2 | 0 | 2 | 0 | 0 | 2 | 0 | 2 | 0 | 0 |
| 98 | 0 | 1 | 5 | 0 | 1 | 3 | 2 | 3 | 2 | 1 | 1 | 0 |
| 99 | 2 | 3 | 4 | 2 | 0 | 1 | 2 | 2 | 0 | 1 | 1 | 1 |
| 100 | 2 | 2 | 2 | 3 | 2 | 2 | 1 | 3 | 3 | 4 | 2 | 3 |
| 101 | 2 | 3 | 2 | 1 | 1 | 1 | 1 | 4 | 2 | 2 | 1 | 3 |
| 102 | 1 | 0 | 2 | 1 | 1 | 1 | 6 | 4 | 6 | 2 | 4 | 3 |
| 103 | 1 | 1 | 0 | 0 | 3 | 0 | 3 | 1 | 4 | 3 | 3 | 2 |
| 104 | 8 | 3 | 7 | 2 | 2 | 4 | 1 | 5 | 8 | 10 | 7 | 4 |
| 105 | 0 | 2 | 1 | 0 | 1 | 3 | 2 | 3 | 0 | 2 | 1 | 2 |

Table 10. Number of newly diagnosed stage IV gastric cancer during each month in 2020.

|  | Month | | | | | | | | | | | |
| --- | --- | --- | --- | --- | --- | --- | --- | --- | --- | --- | --- | --- |
| Hospital | 1 | 2 | 3 | 4 | 5 | 6 | 7 | 8 | 9 | 10 | 11 | 12 |
| 1 | 0 | 1 | 1 | 0 | 0 | 0 | 1 | 3 | 0 | 0 | 0 | 0 |
| 2 | 10 | 11 | 10 | 9 | 13 | 2 | 15 | 6 | 12 | 12 | 9 | 8 |
| 3 | 4 | 0 | 2 | 0 | 0 | 0 | 0 | 4 | 2 | 1 | 1 | 2 |
| 4 | 5 | 2 | 6 | 7 | 3 | 3 | 1 | 3 | 5 | 6 | 3 | 4 |
| 5 | 4 | 3 | 2 | 1 | 3 | 2 | 2 | 0 | 2 | 0 | 2 | 1 |
| 6 | 1 | 1 | 4 | 2 | 1 | 6 | 3 | 1 | 6 | 6 | 1 | 0 |
| 7 | 4 | 3 | 2 | 1 | 2 | 3 | 1 | 3 | 3 | 0 | 3 | 5 |
| 8 | 1 | 0 | 2 | 1 | 0 | 1 | 2 | 3 | 4 | 2 | 3 | 2 |
| 9 | 2 | 1 | 2 | 0 | 1 | 3 | 6 | 3 | 6 | 2 | 1 | 2 |
| 10 |  | 2 | 2 | 1 | 3 | 2 | 1 | 2 | 2 | 4 | 1 | 2 |
| 11 | 3 | 2 | 4 | 3 | 3 | 1 | 4 | 2 | 6 | 6 | 4 | 3 |
| 12 | 2 | 1 | 1 | 2 | 2 | 2 | 0 | 0 | 1 | 1 | 0 | 1 |
| 13 | 1 | 4 | 3 | 3 | 3 | 5 | 5 | 0 | 3 | 4 | 3 | 2 |
| 14 | 2 | 2 | 2 | 0 | 2 | 6 | 1 | 4 | 5 | 2 | 1 | 3 |
| 15 | 3 | 5 | 2 | 3 | 0 | 3 | 6 | 7 | 7 | 4 | 6 | 7 |
| 16 | 5 | 6 | 6 | 9 | 5 | 3 | 7 | 5 | 7 | 11 | 5 | 8 |
| 17 |  |  |  |  |  | 1 |  |  |  |  |  |  |
| 18 | 2 | 0 | 2 | 2 | 3 | 0 | 3 | 2 | 1 | 1 | 0 | 1 |
| 19 | 3 | 1 | 2 | 3 | 0 | 0 | 0 | 2 | 0 | 1 | 0 | 2 |
| 20 | 4 | 5 | 3 | 4 | 4 | 8 | 5 | 7 | 5 | 5 | 4 | 7 |
| 21 | 1 | 5 | 6 | 3 | 1 | 5 | 3 | 3 | 4 | 2 | 1 | 0 |
| 22 | 2 | 5 |  | 1 |  | 2 |  | 2 |  | 1 | 4 |  |
| 23 | 5 | 9 | 7 | 6 | 7 | 6 | 5 | 4 | 5 | 3 | 4 | 3 |
| 24 | 0 | 3 | 6 | 2 | 3 | 1 | 6 | 1 | 2 | 3 | 3 | 3 |
| 25 | 4 | 7 | 10 | 2 | 3 | 7 | 7 | 7 | 3 | 6 | 2 | 3 |
| 26 |  | 3 | 3 |  | 2 | 1 | 3 | 3 | 1 | 3 | 3 | 1 |
| 27 | 0 | 1 | 3 | 1 | 0 | 1 | 2 | 0 | 1 | 0 | 2 | 3 |
| 28 | 1 | 2 | 1 | 3 | 2 | 1 | 1 | 1 | 1 | 2 | 2 | 2 |
| 29 |  |  |  |  |  |  |  |  |  |  |  |  |
| 30 | 1 | 1 | 0 | 2 | 0 | 2 | 3 | 4 | 3 | 2 | 4 | 4 |
| 31 | 1 | 0 | 0 | 1 | 2 | 3 | 3 | 2 | 4 | 3 | 1 | 1 |
| 32 | 1 | 2 | 2 |  | 2 |  | 1 | 3 | 3 | 3 | 1 | 1 |
| 33 | 4 | 2 | 4 | 2 | 5 | 3 | 1 | 2 | 1 | 4 | 1 | 2 |
| 34 |  |  |  |  |  |  |  |  |  |  |  |  |
| 35 | 3 | 1 | 1 | 2 | 4 | 4 | 1 | 4 | 2 | 4 | 2 | 0 |
| 36 | 1 |  | 3 | 1 | 1 | 4 |  |  |  | 3 | 1 | 1 |
| 37 | 5 | 3 | 4 | 2 | 3 | 4 | 2 | 5 | 6 | 12 | 8 | 2 |
| 38 |  |  |  |  |  |  |  |  |  |  |  |  |
| 39 |  | 1 |  | 1 |  | 1 |  |  | 2 |  | 2 | 3 |
| 40 | 2 | 0 | 1 | 0 | 3 | 1 | 1 | 1 | 3 | 6 | 1 | 3 |
| 41 | 1 | 2 | 7 | 1 | 1 | 5 | 1 | 3 | 2 | 2 | 3 | 4 |
| 42 | 2 | 3 | 1 | 0 | 1 | 0 | 1 | 3 | 1 | 1 | 0 | 2 |
| 43 | 3 | 3 | 4 | 3 | 5 | 5 | 6 | 4 | 5 | 2 | 4 | 1 |
| 44 | 0 | 0 | 2 | 0 | 1 | 0 | 1 | 0 | 1 | 1 | 1 | 3 |
| 45 | 5 | 0 | 1 | 2 | 4 | 4 | 5 | 0 | 1 | 2 | 1 | 1 |
| 46 | 0 | 3 | 2 | 6 | 6 | 3 | 7 | 1 | 4 | 3 | 3 | 3 |
| 47 |  |  |  |  |  |  |  |  |  |  |  | 2 |
| 48 | 5 | 6 | 2 | 4 | 5 | 3 | 6 | 3 | 2 | 2 | 2 | 4 |
| 49 | 2 | 0 | 4 | 4 | 2 | 1 | 2 | 0 | 2 | 1 | 0 | 1 |
| 50 | 0 | 0 | 1 | 0 | 0 | 0 | 2 | 0 | 0 | 0 | 0 | 0 |
| 51 | 2 | 5 | 5 | 5 | 4 | 2 | 1 | 4 | 1 | 4 | 3 | 5 |
| 52 | 3 | 0 | 7 | 1 | 1 | 1 | 2 | 2 | 1 | 3 | 3 | 1 |
| 53 | 5 | 5 | 8 | 3 | 3 | 4 | 2 | 6 | 4 | 5 | 3 | 0 |
| 54 | 16 | 7 | 10 | 8 | 15 | 7 | 6 | 8 | 11 | 9 | 9 | 13 |
| 55 | 4 | 3 | 2 | 3 | 3 | 1 | 2 | 1 | 3 | 2 | 3 | 1 |
| 56 | 3 | 2 | 3 | 5 | 1 | 1 | 2 | 4 | 3 | 2 | 1 | 3 |
| 57 | 11 | 2 | 6 | 2 | 2 | 5 | 9 | 2 | 2 | 7 | 2 | 5 |
| 58 | 1 | 3 | 0 | 1 | 2 | 2 | 0 | 1 | 2 | 2 | 2 | 2 |
| 59 | 0 | 1 | 1 | 1 | 4 | 4 | 2 | 1 | 2 | 1 | 2 | 0 |
| 60 | 0 | 1 | 1 | 1 | 2 | 2 | 3 | 0 | 0 | 2 | 2 | 0 |
| 61 | 1 | 1 | 1 | 3 | 1 | 2 | 2 | 4 | 2 | 3 | 0 | 1 |
| 62 |  |  |  |  |  |  |  |  |  |  |  |  |
| 63 |  |  | 2 | 2 |  |  |  |  | 2 |  | 2 |  |
| 64 |  | 2 | 8 | 3 | 2 | 4 | 1 | 1 | 3 | 5 | 7 | 3 |
| 65 | 2 | 4 | 4 | 0 | 1 | 6 | 6 | 1 | 7 | 2 | 3 | 2 |
| 66 | 0 | 0 | 0 | 0 | 0 | 0 | 2 | 0 | 0 | 0 | 0 | 0 |
| 67 | 1 | 1 | 1 | 3 | 1 | 3 | 1 | 1 | 1 | 1 | 3 | 1 |
| 68 | 3 | 1 | 2 | 7 | 3 | 3 | 5 | 2 | 5 | 5 | 3 | 1 |
| 69 | 3 | 4 | 3 | 7 | 4 | 2 | 8 | 4 | 6 | 10 | 7 | 14 |
| 70 |  |  | 1 |  | 2 | 1 |  |  | 1 |  |  |  |
| 71 | 3 | 5 | 1 | 2 | 1 | 6 | 2 | 1 | 2 | 1 | 3 | 0 |
| 72 | 3 | 2 | 6 | 5 | 2 | 2 | 3 | 3 | 1 | 3 | 2 | 7 |
| 73 | 2 |  | 3 | 2 | 3 | 3 | 1 | 1 | 3 | 3 | 2 | 1 |
| 74 | 2 | 3 | 2 | 4 | 11 | 8 | 1 | 3 | 1 | 1 | 3 | 1 |
| 75 |  |  |  |  |  |  |  |  |  |  |  |  |
| 76 | 4 | 2 | 3 | 4 | 8 | 4 | 2 | 3 | 6 | 3 | 3 | 2 |
| 77 | 1 |  |  | 1 | 1 | 1 | 4 |  | 1 |  |  | 1 |
| 78 |  |  |  |  |  |  | 1 |  |  | 2 |  |  |
| 79 | 6 | 3 | 2 | 1 | 1 | 4 | 1 | 3 | 3 | 1 | 2 | 2 |
| 80 | 3 | 0 | 4 | 1 | 4 | 4 | 4 | 3 | 5 | 7 | 3 | 5 |
| 81 | 3 | 4 | 3 |  | 4 | 4 | 2 | 3 | 4 | 2 | 2 | 3 |
| 82 |  |  |  |  |  |  |  |  |  |  |  |  |
| 83 | 3 | 1 | 3 | 1 | 1 | 2 | 1 | 0 | 1 | 1 | 2 | 1 |
| 84 | 1 | 4 | 2 | 1 | 2 | 2 | 3 | 2 | 5 | 3 | 2 | 7 |
| 85 | 0 | 4 | 2 | 1 | 8 | 4 | 3 | 3 | 1 | 4 | 2 | 1 |
| 86 | 4 | 5 | 1 | 3 | 2 | 4 | 2 | 2 | 3 | 2 | 1 | 4 |
| 87 | 4 | 1 | 1 | 2 | 2 | 1 |  | 3 |  | 2 | 1 |  |
| 88 | 3 | 1 | 2 | 2 | 1 | 2 | 2 | 0 | 3 | 3 | 0 | 3 |
| 89 | 12 | 12 | 11 | 10 | 8 | 10 | 16 | 6 | 16 | 16 | 9 | 15 |
| 90 | 1 | 0 | 1 | 2 | 2 | 1 | 3 | 2 | 0 | 1 | 5 | 1 |
| 91 | 1 | 3 | 5 | 1 | 1 | 0 | 2 | 0 | 3 | 5 | 2 | 1 |
| 92 |  |  |  |  |  |  |  |  |  |  |  |  |
| 93 | 1 | 2 | 1 | 1 | 2 | 5 | 1 | 1 | 4 | 1 | 0 | 2 |
| 94 | 2 | 2 | 2 | 0 | 1 | 1 | 2 | 3 | 1 | 0 | 2 | 5 |
| 95 | 0 | 0 | 0 | 1 | 0 | 2 | 0 | 1 | 2 | 3 | 1 | 0 |
| 96 | 4 | 1 | 2 | 1 | 2 | 3 | 1 | 3 | 4 | 0 | 3 | 3 |
| 97 | 1 | 0 | 1 | 2 | 2 | 3 | 1 | 1 | 0 | 1 | 1 | 1 |
| 98 | 5 | 1 | 3 | 1 | 2 | 2 | 5 | 3 | 5 | 3 | 1 | 0 |
| 99 | 1 | 3 | 0 | 1 | 0 | 0 | 5 | 2 | 2 | 1 | 2 | 0 |
| 100 | 3 | 2 | 2 | 3 | 2 | 4 | 3 | 1 | 0 | 3 | 1 | 2 |
| 101 | 2 | 2 | 1 | 1 | 3 | 3 | 3 | 1 |  | 2 | 2 |  |
| 102 | 2 | 3 | 2 | 2 | 3 | 1 | 3 | 0 | 6 | 3 | 1 | 4 |
| 103 | 2 | 1 | 1 | 3 | 4 | 2 | 1 | 1 | 5 | 2 | 3 | 1 |
| 104 | 4 | 4 | 4 | 5 | 5 | 4 | 3 | 1 | 2 | 9 | 5 | 7 |
| 105 | 2 | 2 | 4 | 3 | 1 | 2 | 2 | 1 | 2 | 3 | 3 | 3 |

Table 11. Number of newly diagnosed colorectal cancer during each month in 2019.

|  | Month | | | | | | | | | | | |
| --- | --- | --- | --- | --- | --- | --- | --- | --- | --- | --- | --- | --- |
| Hospital | 1 | 2 | 3 | 4 | 5 | 6 | 7 | 8 | 9 | 10 | 11 | 12 |
| 1 | 19 | 12 | 3 | 6 | 6 | 9 | 4 | 12 | 5 | 15 | 12 | 5 |
| 2 | 73 | 86 | 84 | 81 | 66 | 86 | 71 | 62 | 72 | 65 | 84 | 69 |
| 3 | 20 | 15 | 12 | 13 | 16 | 16 | 28 | 15 | 17 | 18 | 10 | 17 |
| 4 | 17 | 24 | 21 | 23 | 27 | 20 | 26 | 18 | 17 | 24 | 10 | 25 |
| 5 | 8 | 11 | 9 | 10 | 11 | 8 | 13 | 10 | 9 | 7 | 10 | 12 |
| 6 | 20 | 20 | 16 | 21 | 17 | 25 | 22 | 26 | 24 | 25 | 14 | 16 |
| 7 | 12 | 30 | 23 | 19 | 25 | 21 | 15 | 13 | 13 | 16 | 22 | 27 |
| 8 |  |  |  |  |  |  |  |  |  |  |  |  |
| 9 | 16 | 21 | 18 | 21 | 19 | 16 | 22 | 10 | 22 | 24 | 19 | 26 |
| 10 | 12 | 12 | 12 | 10 | 16 | 12 | 25 | 9 | 5 | 17 | 20 | 8 |
| 11 | 18 | 17 | 18 | 20 | 16 | 22 | 28 | 29 | 30 | 20 | 17 | 11 |
| 12 | 7 | 5 | 2 | 5 | 5 | 5 | 4 | 4 | 7 | 3 | 7 | 5 |
| 13 | 28 | 29 | 20 | 29 | 18 | 24 | 35 | 21 | 24 | 15 | 25 | 25 |
| 14 | 33 | 29 | 22 | 22 | 28 | 34 | 26 | 25 | 19 | 28 | 16 | 26 |
| 15 | 24 | 29 | 19 | 26 | 25 | 14 | 20 | 24 | 20 | 24 | 21 | 21 |
| 16 | 27 | 25 | 29 | 30 | 26 | 28 | 33 | 28 | 25 | 23 | 30 | 27 |
| 17 | 1 |  |  |  |  | 1 |  |  |  | 1 | 1 |  |
| 18 | 10 | 8 | 11 | 20 | 12 | 9 | 9 | 12 | 10 | 13 | 15 | 18 |
| 19 | 8 | 6 | 11 | 6 | 9 | 6 | 8 | 11 | 4 | 9 | 10 | 17 |
| 20 | 41 | 31 | 42 | 38 | 32 | 38 | 32 | 44 | 35 | 44 | 46 | 37 |
| 21 | 12 | 14 | 21 | 15 | 18 | 16 | 26 | 23 | 11 | 16 | 16 | 25 |
| 22 | 7 | 5 | 14 | 14 | 7 | 11 | 6 | 16 | 12 | 7 | 20 | 9 |
| 23 |  |  |  |  |  |  |  |  |  |  |  |  |
| 24 | 10 | 25 | 26 | 20 | 13 | 15 | 28 | 17 | 17 | 31 | 18 | 13 |
| 25 | 32 | 28 | 47 | 34 | 24 | 25 | 31 | 36 | 29 | 35 | 34 | 33 |
| 26 | 14 | 12 | 8 | 13 | 5 | 6 | 8 | 7 | 12 | 13 | 9 | 9 |
| 27 | 7 | 4 | 6 | 8 | 3 | 9 | 6 | 6 | 12 | 7 | 5 | 9 |
| 28 | 14 | 18 | 15 | 6 | 9 | 15 | 14 | 9 | 16 | 18 | 19 | 15 |
| 29 | 46 | 42 | 34 | 43 | 39 | 30 | 40 | 34 | 39 | 31 | 31 | 33 |
| 30 | 7 | 9 | 13 | 11 | 4 | 6 | 9 | 12 | 13 | 15 | 9 | 7 |
| 31 | 23 | 20 | 13 | 29 | 28 | 18 | 18 | 17 | 11 | 22 | 17 | 10 |
| 32 | 6 | 11 | 9 | 15 | 13 | 9 | 15 | 6 | 12 | 17 | 11 | 16 |
| 33 | 16 | 20 | 13 | 13 | 13 | 12 | 12 | 10 | 10 | 22 | 13 | 16 |
| 34 | 13 | 15 | 19 | 15 | 23 | 19 | 15 | 19 | 13 | 18 | 15 | 9 |
| 35 | 15 | 30 | 20 | 25 | 27 | 11 | 21 | 24 | 13 | 20 | 17 | 19 |
| 36 | 11 | 4 | 7 | 9 | 6 | 4 | 14 | 12 | 9 | 9 | 14 | 15 |
| 37 | 13 | 20 | 15 | 22 | 14 | 21 | 20 | 24 | 24 | 23 | 18 | 19 |
| 38 | 48 | 34 | 39 | 42 | 36 | 39 | 40 | 37 | 34 | 40 | 27 | 38 |
| 39 |  |  |  |  |  |  |  |  |  |  |  |  |
| 40 | 23 | 27 | 22 | 25 | 20 | 27 | 36 | 20 | 29 | 23 | 20 | 28 |
| 41 | 13 | 17 | 21 | 25 | 22 | 18 | 22 | 12 | 18 | 17 | 23 | 25 |
| 42 | 12 | 8 | 9 | 8 | 6 | 11 | 16 | 8 | 6 | 9 | 11 | 18 |
| 43 | 21 | 10 | 19 | 22 | 25 | 21 | 23 | 21 | 13 | 13 | 17 | 19 |
| 44 | 4 | 7 | 6 | 8 | 2 | 7 | 7 | 7 | 1 | 3 | 3 | 6 |
| 45 | 10 | 12 | 9 | 22 | 19 | 24 | 21 | 7 | 12 | 29 | 19 | 12 |
| 46 | 25 | 24 | 21 | 23 | 28 | 30 | 22 | 24 | 24 | 13 | 22 | 22 |
| 47 | 2 | 2 | 7 | 1 | 8 | 11 | 9 | 7 | 9 | 11 | 4 | 5 |
| 48 | 18 | 23 | 26 | 26 | 33 | 12 | 20 | 20 | 20 | 23 | 17 | 21 |
| 49 | 9 | 7 | 10 | 9 | 11 | 11 | 13 | 11 | 4 | 7 | 11 | 20 |
| 50 | 2 | 6 | 7 | 2 | 4 | 4 | 7 | 5 | 3 | 8 | 3 | 5 |
| 51 | 34 | 37 | 36 | 32 | 36 | 37 | 48 | 44 | 49 | 47 | 32 | 39 |
| 52 | 19 | 18 | 25 | 20 | 18 | 11 | 20 | 19 | 21 | 20 | 21 | 24 |
| 53 | 25 | 22 | 17 | 23 | 19 | 24 | 25 | 21 | 30 | 23 | 24 | 30 |
| 54 | 91 | 110 | 74 | 78 | 90 | 71 | 81 | 82 | 93 | 103 | 101 | 129 |
| 55 | 24 | 35 | 20 | 15 | 29 | 24 | 25 | 20 | 11 | 26 | 20 | 18 |
| 56 | 31 | 33 | 30 | 31 | 29 | 28 | 35 | 28 | 28 | 23 | 19 | 40 |
| 57 | 45 | 40 | 53 | 43 | 47 | 38 | 45 | 25 | 34 | 40 | 38 | 32 |
| 58 | 12 | 15 | 16 | 14 | 14 | 24 | 9 | 7 | 18 | 13 | 17 | 20 |
| 59 | 10 | 11 | 9 | 10 | 11 | 14 | 14 | 12 | 7 | 17 | 5 | 11 |
| 60 | 21 | 20 | 22 | 22 | 20 | 23 | 20 | 29 | 28 | 26 | 30 | 32 |
| 61 | 11 | 10 | 13 | 14 | 11 | 19 | 12 | 6 | 11 | 11 | 15 | 14 |
| 62 | 34 | 30 | 31 | 27 | 22 | 29 | 30 | 25 | 25 | 36 | 20 | 36 |
| 63 | 10 | 5 | 8 | 7 | 10 | 3 | 6 | 9 | 10 | 9 | 10 | 8 |
| 64 |  |  |  |  |  |  |  |  |  |  |  |  |
| 65 | 22 | 25 | 23 | 16 | 22 | 16 | 19 | 35 | 21 | 23 | 24 | 27 |
| 66 | 4 | 5 | 3 | 2 | 6 | 5 | 2 | 9 | 8 | 9 | 1 | 4 |
| 67 | 10 | 8 | 7 | 15 | 7 | 7 | 8 | 4 | 15 | 8 | 11 | 5 |
| 68 | 26 | 30 | 37 | 30 | 33 | 28 | 37 | 23 | 22 | 16 | 38 | 29 |
| 69 | 19 | 32 | 39 | 35 | 20 | 28 | 30 | 27 | 27 | 42 | 28 | 35 |
| 70 | 4 | 5 | 3 | 6 | 6 | 9 | 3 | 3 | 10 | 3 | 5 | 4 |
| 71 | 9 | 8 | 20 | 17 | 11 | 15 | 15 | 13 | 8 | 14 | 7 | 11 |
| 72 | 46 | 44 | 26 | 27 | 26 | 28 | 37 | 21 | 29 | 22 | 34 | 25 |
| 73 | 17 | 20 | 21 | 17 | 20 | 24 | 22 | 16 | 16 | 23 | 17 | 17 |
| 74 | 28 | 33 | 31 | 29 | 29 | 36 | 25 | 34 | 21 | 26 | 27 | 35 |
| 75 | 1 | 2 | 1 | 0 | 3 | 1 | 1 | 1 | 2 | 1 | 1 | 2 |
| 76 | 13 | 7 | 16 | 19 | 11 | 18 | 16 | 16 | 24 | 19 | 21 | 16 |
| 77 | 0 | 3 | 2 | 5 | 6 | 4 | 3 | 2 | 4 | 3 | 3 | 3 |
| 78 |  |  |  |  |  |  |  |  |  |  |  |  |
| 79 | 22 | 16 | 17 | 19 | 19 | 19 | 19 | 32 | 23 | 20 | 25 | 35 |
| 80 | 10 | 15 | 16 | 14 | 15 | 16 | 16 | 22 | 21 | 17 | 15 | 13 |
| 81 | 10 | 16 | 14 | 12 | 4 | 10 | 18 | 15 | 17 | 25 | 18 | 16 |
| 82 |  |  |  |  |  |  |  |  |  |  |  |  |
| 83 |  |  |  |  |  |  |  |  |  |  |  |  |
| 84 | 22 | 25 | 30 | 25 | 24 | 25 | 19 | 27 | 26 | 14 | 23 | 15 |
| 85 | 9 | 18 | 13 | 14 | 13 | 12 | 17 | 9 | 12 | 15 | 9 | 13 |
| 86 | 23 | 17 | 24 | 23 | 11 | 33 | 13 | 22 | 31 | 15 | 16 | 17 |
| 87 | 11 | 15 | 15 | 11 | 30 | 8 | 12 | 9 | 15 | 7 | 12 | 15 |
| 88 | 18 | 13 | 13 | 15 | 22 | 14 | 22 | 20 | 13 | 12 | 26 | 15 |
| 89 | 47 | 64 | 53 | 41 | 39 | 50 | 35 | 42 | 48 | 40 | 40 | 51 |
| 90 | 22 | 14 | 25 | 19 | 28 | 17 | 26 | 31 | 23 | 20 | 13 | 17 |
| 91 | 28 | 29 | 26 | 22 | 27 | 26 | 30 | 23 | 19 | 24 | 16 | 29 |
| 92 |  |  |  |  |  |  |  |  |  |  |  |  |
| 93 |  |  |  |  |  |  |  |  |  |  |  |  |
| 94 | 10 | 5 | 15 | 9 | 17 | 8 | 11 | 10 | 11 | 9 | 11 | 5 |
| 95 | 7 | 19 | 9 | 7 | 10 | 5 | 5 | 12 | 5 | 8 | 7 | 5 |
| 96 | 11 | 16 | 13 | 14 | 10 | 15 | 12 | 12 | 8 | 18 | 11 | 7 |
| 97 | 6 | 1 | 3 | 2 | 8 | 2 | 6 | 1 | 4 | 4 | 6 | 3 |
| 98 | 11 | 6 | 12 | 7 | 15 | 13 | 13 | 10 | 6 | 11 | 10 | 10 |
| 99 | 8 | 11 | 13 | 13 | 12 | 12 | 10 | 9 | 12 | 6 | 10 | 13 |
| 100 | 13 | 13 | 13 | 19 | 7 | 14 | 15 | 17 | 19 | 13 | 18 | 9 |
| 101 | 20 | 20 | 29 | 23 | 16 | 20 | 24 | 21 | 26 | 24 | 20 | 27 |
| 102 |  |  |  |  |  |  |  |  |  |  |  |  |
| 103 | 22 | 13 | 18 | 18 | 28 | 20 | 26 | 33 | 16 | 29 | 21 | 27 |
| 104 | 37 | 28 | 36 | 42 | 45 | 34 | 45 | 33 | 38 | 38 | 26 | 42 |
| 105 | 14 | 19 | 17 | 11 | 13 | 18 | 17 | 9 | 19 | 11 | 23 | 18 |

Table 12. Number of newly diagnosed colorectal cancer during each month in 2020.

|  | Month | | | | | | | | | | | |
| --- | --- | --- | --- | --- | --- | --- | --- | --- | --- | --- | --- | --- |
| Hospital | 1 | 2 | 3 | 4 | 5 | 6 | 7 | 8 | 9 | 10 | 11 | 12 |
| 1 | 6 | 16 | 12 | 6 | 11 | 6 | 10 | 4 | 7 | 7 | 7 | 6 |
| 2 | 83 | 53 | 84 | 70 | 36 | 46 | 43 | 44 | 63 | 71 | 70 | 78 |
| 3 | 15 | 13 | 19 | 10 | 14 | 14 | 7 | 15 | 14 | 19 | 12 | 22 |
| 4 | 13 | 23 | 23 | 22 | 19 | 16 | 16 | 15 | 21 | 26 | 16 | 24 |
| 5 | 12 | 6 | 4 | 5 | 3 | 12 | 6 | 5 | 10 | 9 | 14 | 15 |
| 6 | 18 | 22 | 17 | 17 | 13 | 26 | 20 | 9 | 9 | 26 | 19 | 19 |
| 7 | 12 | 17 | 15 | 14 | 13 | 21 | 17 | 17 | 12 | 24 | 21 | 26 |
| 8 |  |  |  |  |  |  |  |  |  |  |  |  |
| 9 | 14 | 14 | 20 | 18 | 11 | 17 | 22 | 10 | 19 | 20 | 12 | 18 |
| 10 | 14 | 17 | 16 | 12 | 7 | 11 | 13 | 13 | 16 | 16 | 10 | 9 |
| 11 | 15 | 12 | 18 | 7 | 13 | 13 | 24 | 25 | 27 | 18 | 21 | 16 |
| 12 | 2 | 6 | 6 | 4 | 3 | 6 | 2 | 4 | 5 | 3 | 5 | 2 |
| 13 | 24 | 16 | 20 | 20 | 16 | 29 | 25 | 19 | 24 | 22 | 21 | 15 |
| 14 | 23 | 13 | 16 | 23 | 13 | 26 | 27 | 25 | 31 | 21 | 21 | 29 |
| 15 | 15 | 10 | 20 | 12 | 12 | 12 | 19 | 11 | 21 | 23 | 17 | 25 |
| 16 | 36 | 22 | 26 | 32 | 37 | 27 | 35 | 12 | 27 | 21 | 28 | 20 |
| 17 |  |  |  |  |  | 1 |  |  | 1 |  |  | 2 |
| 18 | 9 | 14 | 16 | 14 | 9 | 18 | 11 | 15 | 16 | 13 | 8 | 19 |
| 19 | 7 | 4 | 11 | 6 | 3 | 6 | 9 | 9 | 5 | 5 | 4 | 6 |
| 20 | 36 | 32 | 41 | 16 | 15 | 28 | 24 | 26 | 30 | 51 | 26 | 37 |
| 21 | 21 | 12 | 9 | 13 | 10 | 11 | 22 | 21 | 21 | 16 | 20 | 15 |
| 22 | 10 | 18 | 18 | 6 | 5 | 7 | 9 | 6 | 11 | 8 | 9 | 9 |
| 23 |  |  |  |  |  |  |  |  |  |  |  |  |
| 24 | 22 | 17 | 18 | 18 | 16 | 24 | 28 | 16 | 25 | 27 | 14 | 17 |
| 25 | 24 | 33 | 26 | 20 | 14 | 24 | 30 | 14 | 22 | 23 | 23 | 36 |
| 26 | 5 | 10 | 13 | 7 | 8 | 11 | 6 | 10 | 12 | 9 | 8 | 14 |
| 27 | 8 | 9 | 10 | 1 | 6 | 10 | 4 | 5 | 5 | 7 | 10 | 12 |
| 28 | 8 | 14 | 20 | 14 | 7 | 14 | 20 | 12 | 18 | 18 | 21 | 16 |
| 29 | 27 | 30 | 42 | 29 | 29 | 35 | 25 | 36 | 34 | 33 | 24 | 35 |
| 30 | 8 | 6 | 14 | 13 | 4 | 11 | 12 | 7 | 9 | 18 | 6 | 5 |
| 31 | 14 | 19 | 22 | 19 | 11 | 20 | 16 | 18 | 12 | 20 | 16 | 18 |
| 32 | 10 | 5 | 11 | 7 | 6 | 8 | 7 | 9 | 11 | 7 | 12 | 6 |
| 33 | 9 | 11 | 9 | 12 | 22 | 14 | 19 | 9 | 8 | 23 | 14 | 10 |
| 34 | 15 | 15 | 17 | 7 | 6 | 11 | 12 | 14 | 22 | 21 | 10 | 16 |
| 35 | 27 | 18 | 26 | 19 | 17 | 23 | 17 | 20 | 14 | 29 | 11 | 15 |
| 36 | 9 | 8 | 10 | 13 | 13 | 7 | 10 | 6 | 15 | 15 | 7 | 12 |
| 37 | 18 | 29 | 13 | 21 | 23 | 19 | 16 | 18 | 17 | 21 | 21 | 25 |
| 38 | 52 | 40 | 37 | 21 | 16 | 35 | 25 | 20 | 33 | 27 | 26 | 32 |
| 39 |  |  |  |  |  |  |  |  |  |  |  |  |
| 40 | 24 | 27 | 21 | 25 | 25 | 33 | 22 | 28 | 32 | 27 | 23 | 23 |
| 41 | 20 | 17 | 16 | 23 | 16 | 18 | 30 | 16 | 27 | 14 | 25 | 12 |
| 42 | 6 | 9 | 19 | 15 | 5 | 13 | 18 | 6 | 13 | 14 | 6 | 9 |
| 43 | 19 | 12 | 15 | 11 | 10 | 17 | 14 | 17 | 11 | 16 | 16 | 20 |
| 44 | 6 | 3 | 4 | 4 | 3 | 7 | 8 | 3 | 3 | 3 | 6 | 14 |
| 45 | 11 | 22 | 11 | 12 | 7 | 20 | 12 | 14 | 15 | 18 | 15 | 16 |
| 46 | 18 | 27 | 25 | 29 | 18 | 28 | 31 | 19 | 22 | 27 | 23 | 31 |
| 47 | 2 | 9 | 8 | 6 | 7 | 5 | 9 | 12 | 5 | 5 | 7 | 16 |
| 48 | 20 | 21 | 15 | 13 | 17 | 20 | 22 | 13 | 27 | 24 | 13 | 21 |
| 49 | 6 | 7 | 4 | 6 | 5 | 7 | 5 | 4 | 5 | 5 | 10 | 7 |
| 50 | 6 | 7 | 3 | 4 | 4 | 3 | 3 | 4 | 6 | 2 | 4 | 3 |
| 51 | 34 | 37 | 42 | 28 | 22 | 30 | 33 | 37 | 36 | 38 | 44 | 57 |
| 52 | 8 | 16 | 15 | 25 | 5 | 17 | 14 | 15 | 17 | 28 | 21 | 24 |
| 53 | 21 | 13 | 34 | 18 | 14 | 14 | 19 | 26 | 20 | 35 | 15 | 20 |
| 54 | 90 | 86 | 70 | 82 | 45 | 79 | 81 | 73 | 79 | 90 | 104 | 92 |
| 55 | 30 | 26 | 15 | 5 | 19 | 23 | 15 | 23 | 21 | 25 | 16 | 18 |
| 56 | 27 | 26 | 29 | 27 | 10 | 27 | 18 | 24 | 20 | 28 | 26 | 29 |
| 57 | 30 | 48 | 37 | 28 | 20 | 26 | 21 | 25 | 29 | 30 | 31 | 45 |
| 58 | 15 | 12 | 22 | 16 | 14 | 17 | 15 | 12 | 18 | 18 | 21 | 19 |
| 59 | 14 | 9 | 16 | 8 | 8 | 8 | 5 | 6 | 12 | 11 | 7 | 7 |
| 60 | 19 | 21 | 25 | 14 | 23 | 17 | 14 | 13 | 14 | 14 | 11 | 23 |
| 61 | 9 | 15 | 15 | 8 | 11 | 8 | 13 | 14 | 12 | 22 | 9 | 12 |
| 62 | 34 | 22 | 37 | 28 | 16 | 23 | 14 | 17 | 30 | 22 | 27 | 36 |
| 63 | 14 | 9 | 10 | 6 | 9 | 6 | 6 | 5 | 8 | 8 | 9 | 9 |
| 64 |  |  |  |  |  |  |  |  |  |  |  |  |
| 65 | 28 | 27 | 24 | 14 | 7 | 20 | 22 | 25 | 19 | 20 | 24 | 35 |
| 66 | 2 | 4 | 1 | 2 | 2 | 3 | 6 | 6 | 6 | 7 | 6 | 2 |
| 67 | 7 | 14 | 8 | 20 | 8 | 10 | 8 | 8 | 6 | 10 | 15 | 13 |
| 68 | 25 | 25 | 29 | 25 | 20 | 29 | 20 | 22 | 23 | 36 | 26 | 29 |
| 69 | 24 | 35 | 21 | 31 | 22 | 29 | 28 | 42 | 36 | 50 | 36 | 43 |
| 70 | 6 | 4 | 2 | 5 | 5 | 6 | 9 | 4 | 2 | 3 | 1 | 3 |
| 71 | 16 | 12 | 11 | 10 | 8 | 6 | 9 | 13 | 18 | 9 | 14 | 13 |
| 72 | 34 | 31 | 21 | 17 | 12 | 16 | 21 | 25 | 30 | 37 | 31 | 50 |
| 73 | 10 | 15 | 21 | 13 | 8 | 11 | 15 | 13 | 15 | 15 | 15 | 17 |
| 74 | 38 | 31 | 33 | 34 | 29 | 21 | 39 | 27 | 35 | 33 | 22 | 21 |
| 75 | 0 | 1 | 0 | 1 | 0 | 1 | 1 | 0 | 0 | 0 | 0 | 0 |
| 76 | 15 | 18 | 11 | 12 | 11 | 18 | 11 | 22 | 16 | 25 | 16 | 20 |
| 77 | 6 | 2 | 5 | 2 | 5 | 3 | 4 | 3 | 4 | 1 | 5 | 5 |
| 78 |  |  |  |  |  |  |  |  |  |  |  |  |
| 79 | 32 | 25 | 29 | 11 | 4 | 10 | 19 | 23 | 18 | 22 | 20 | 26 |
| 80 | 14 | 7 | 14 | 10 | 15 | 13 | 16 | 11 | 18 | 25 | 14 | 12 |
| 81 | 12 | 19 | 19 | 6 | 9 | 16 | 16 | 16 | 15 | 16 | 6 | 12 |
| 82 |  |  |  |  |  |  |  |  |  |  |  |  |
| 83 |  |  |  |  |  |  |  |  |  |  |  |  |
| 84 | 24 | 24 | 14 | 15 | 15 | 27 | 32 | 30 | 24 | 25 | 22 | 28 |
| 85 | 14 | 14 | 8 | 6 | 12 | 9 | 9 | 14 | 16 | 14 | 18 | 17 |
| 86 | 20 | 18 | 21 | 15 | 15 | 26 | 25 | 16 | 17 | 17 | 16 | 23 |
| 87 | 13 | 17 | 9 | 5 | 5 | 12 | 6 | 13 | 11 | 4 | 5 | 13 |
| 88 | 25 | 14 | 13 | 10 | 14 | 17 | 22 | 17 | 17 | 15 | 11 | 25 |
| 89 | 42 | 37 | 36 | 39 | 31 | 45 | 35 | 42 | 29 | 40 | 42 | 47 |
| 90 | 20 | 12 | 30 | 23 | 13 | 22 | 21 | 13 | 11 | 12 | 17 | 31 |
| 91 | 21 | 22 | 22 | 22 | 14 | 21 | 22 | 19 | 19 | 26 | 22 | 21 |
| 92 |  |  |  |  |  |  |  |  |  |  |  |  |
| 93 |  |  |  |  |  |  |  |  |  |  |  |  |
| 94 | 6 | 9 | 11 | 9 | 7 | 6 | 6 | 4 | 10 | 13 | 9 | 8 |
| 95 | 8 | 6 | 6 | 8 | 10 | 8 | 3 | 8 | 6 | 9 | 5 | 5 |
| 96 | 16 | 18 | 17 | 12 | 7 | 14 | 13 | 16 | 18 | 22 | 15 | 18 |
| 97 | 5 | 8 | 5 | 2 | 3 | 2 | 1 | 2 | 1 | 3 | 1 | 7 |
| 98 | 8 | 14 | 10 | 8 | 13 | 14 | 13 | 5 | 6 | 13 | 13 | 9 |
| 99 | 12 | 12 | 15 | 7 | 12 | 7 | 9 | 8 | 11 | 10 | 5 | 11 |
| 100 | 12 | 9 | 9 | 18 | 9 | 9 | 19 | 11 | 12 | 19 | 10 | 19 |
| 101 | 21 | 18 | 20 | 22 | 15 | 15 | 16 | 21 | 30 | 18 | 21 | 22 |
| 102 |  |  |  |  |  |  |  |  |  |  |  |  |
| 103 | 26 | 19 | 30 | 20 | 18 | 13 | 16 | 21 | 16 | 17 | 25 | 12 |
| 104 | 29 | 28 | 39 | 34 | 30 | 39 | 33 | 23 | 32 | 34 | 42 | 26 |
| 105 | 8 | 12 | 9 | 12 | 13 | 15 | 11 | 8 | 9 | 15 | 24 | 16 |

Table 13. Number of newly diagnosed stage I colorectal cancer during each month in 2019.

|  | Month | | | | | | | | | | | |
| --- | --- | --- | --- | --- | --- | --- | --- | --- | --- | --- | --- | --- |
| Hospital | 1 | 2 | 3 | 4 | 5 | 6 | 7 | 8 | 9 | 10 | 11 | 12 |
| 1 | 5 | 3 | 0 | 1 | 2 | 2 | 0 | 2 | 2 | 3 | 1 | 2 |
| 2 | 25 | 21 | 24 | 27 | 20 | 27 | 20 | 21 | 27 | 27 | 29 | 27 |
| 3 | 2 | 3 | 2 | 1 | 1 | 1 | 3 | 2 | 5 | 6 | 3 | 4 |
| 4 | 0 | 5 | 4 | 7 | 6 | 4 | 2 | 4 | 3 | 3 | 1 | 4 |
| 5 | 3 | 5 | 1 | 6 | 2 | 2 | 3 | 2 | 2 | 1 | 4 | 3 |
| 6 | 4 | 3 | 3 | 2 | 1 | 6 | 3 | 7 | 4 | 6 | 2 | 0 |
| 7 | 2 | 5 | 6 | 3 | 3 | 4 | 4 | 1 | 1 | 5 | 6 | 7 |
| 8 |  |  |  |  |  |  |  |  |  |  |  |  |
| 9 | 4 | 6 | 6 | 8 | 8 | 2 | 5 | 4 | 5 | 8 | 6 | 8 |
| 10 | 3 | 4 | 5 | 3 | 5 | 4 | 8 | 4 | 1 | 2 | 9 | 2 |
| 11 | 13 | 4 | 9 | 7 | 5 | 7 | 11 | 10 | 13 | 7 | 6 | 3 |
| 12 | 1 | 1 | 1 | 2 | 1 | 2 | 0 | 1 | 3 | 0 | 2 | 3 |
| 13 | 6 | 7 | 3 | 7 | 1 | 2 | 5 | 4 | 4 | 2 | 6 | 6 |
| 14 | 9 | 7 | 2 | 3 | 7 | 5 | 1 | 5 | 2 | 4 | 3 | 6 |
| 15 | 11 | 9 | 8 | 9 | 9 | 6 | 6 | 6 | 8 | 8 | 6 | 5 |
| 16 | 10 | 6 | 5 | 7 | 3 | 12 | 14 | 10 | 1 | 6 | 9 | 8 |
| 17 |  |  |  |  |  |  |  |  |  |  |  |  |
| 18 | 4 | 4 | 7 | 8 | 7 | 2 | 5 | 5 | 3 | 8 | 5 | 7 |
| 19 | 2 | 3 | 2 | 2 | 2 | 1 | 3 | 3 | 3 | 4 | 3 | 6 |
| 20 | 13 | 12 | 10 | 13 | 7 | 8 | 7 | 15 | 9 | 9 | 16 | 14 |
| 21 | 0 | 3 | 4 | 4 | 2 | 2 | 3 | 5 | 0 | 4 | 4 | 9 |
| 22 | 2 | 1 | 3 | 5 | 2 |  |  | 1 | 4 | 2 | 5 | 3 |
| 23 |  |  |  |  |  |  |  |  |  |  |  |  |
| 24 | 6 | 10 | 8 | 7 | 8 | 6 | 8 | 6 | 9 | 5 | 4 | 4 |
| 25 | 10 | 6 | 11 | 9 | 4 | 7 | 1 | 12 | 7 | 8 | 9 | 8 |
| 26 | 8 | 3 | 4 | 3 | 1 | 3 | 2 | 1 | 6 | 3 | 2 | 3 |
| 27 | 2 | 0 | 0 | 0 | 1 | 2 | 0 | 2 | 4 | 2 | 0 | 1 |
| 28 | 4 | 5 | 4 | 1 | 0 | 3 | 1 | 0 | 3 | 5 | 2 | 4 |
| 29 |  |  |  |  |  |  |  |  |  |  |  |  |
| 30 | 2 | 4 | 6 | 3 | 0 | 3 | 5 | 1 | 3 | 6 | 3 | 2 |
| 31 | 5 | 4 | 4 | 4 | 7 | 1 | 4 | 3 | 2 | 5 | 5 | 1 |
| 32 | 3 | 4 | 3 | 9 | 5 | 4 | 8 | 4 | 5 | 6 | 8 | 8 |
| 33 | 6 | 8 | 4 | 4 | 1 | 4 | 3 | 2 | 3 | 4 | 1 | 3 |
| 34 | 6 | 9 | 13 | 7 | 7 | 9 | 4 | 9 | 8 | 7 | 7 | 6 |
| 35 | 0 | 4 | 5 | 5 | 5 | 5 | 5 | 5 | 3 | 3 | 4 | 6 |
| 36 | 4 | 1 | 3 | 4 | 2 | 1 | 5 | 2 | 1 | 2 | 3 | 6 |
| 37 | 5 | 8 | 8 | 7 | 4 | 6 | 8 | 10 | 7 | 11 | 9 | 6 |
| 38 |  |  |  |  |  |  |  |  |  |  |  |  |
| 39 |  | 2 | 5 | 3 | 3 | 1 | 3 | 4 | 1 | 1 | 2 |  |
| 40 | 6 | 9 | 3 | 11 | 7 | 10 | 7 | 4 | 7 | 7 | 9 | 11 |
| 41 | 5 | 4 | 3 | 10 | 6 | 9 | 5 | 5 | 5 | 9 | 6 | 5 |
| 42 | 5 | 3 | 3 | 1 | 1 | 5 | 6 | 5 | 4 | 3 | 4 | 6 |
| 43 | 4 | 5 | 6 | 6 | 8 | 6 | 3 | 6 | 5 | 3 | 5 | 8 |
| 44 | 1 | 0 | 0 | 1 | 0 | 1 | 0 | 0 | 0 | 1 | 0 | 3 |
| 45 | 0 | 5 | 3 | 6 | 4 | 6 | 4 | 2 | 3 | 6 | 8 | 4 |
| 46 | 4 | 5 | 1 | 2 | 5 | 8 | 3 | 5 | 6 | 5 | 2 | 6 |
| 47 |  | 2 | 2 | 1 | 3 | 5 | 4 | 3 | 2 | 5 | 1 | 2 |
| 48 | 4 | 3 | 4 | 4 | 7 | 0 | 7 | 2 | 1 | 5 | 5 | 4 |
| 49 | 5 | 2 | 3 | 2 | 2 | 4 | 4 | 2 | 0 | 2 | 2 | 6 |
| 50 | 1 | 1 | 0 | 1 | 0 | 1 | 1 | 0 | 0 | 2 | 1 | 2 |
| 51 | 7 | 7 | 4 | 3 | 6 | 5 | 7 | 6 | 8 | 6 | 5 | 5 |
| 52 | 5 | 6 | 7 | 7 | 5 | 3 | 5 | 6 | 6 | 3 | 6 | 8 |
| 53 | 11 | 10 | 8 | 7 | 5 | 9 | 11 | 5 | 14 | 7 | 6 | 5 |
| 54 | 11 | 20 | 11 | 12 | 15 | 10 | 9 | 12 | 13 | 20 | 20 | 24 |
| 55 | 6 | 11 | 5 | 8 | 4 | 4 | 7 | 8 | 2 | 8 | 4 | 2 |
| 56 | 6 | 10 | 12 | 7 | 3 | 9 | 9 | 5 | 6 | 9 | 7 | 14 |
| 57 | 14 | 13 | 19 | 16 | 22 | 15 | 16 | 10 | 18 | 13 | 18 | 10 |
| 58 | 0 | 2 | 1 | 3 | 3 | 6 | 0 | 4 | 3 | 4 | 2 | 4 |
| 59 | 3 | 4 | 4 | 2 | 3 | 3 | 8 | 3 | 5 | 7 | 3 | 4 |
| 60 | 3 | 4 | 6 | 3 | 3 | 3 | 5 | 1 | 5 | 7 | 9 | 7 |
| 61 | 5 | 2 | 4 | 4 | 4 | 4 | 2 | 1 | 1 | 3 | 2 | 2 |
| 62 |  |  |  |  |  |  |  |  |  |  |  |  |
| 63 | 4 |  | 2 | 3 | 4 | 1 |  | 3 | 1 | 2 | 2 | 2 |
| 64 | 7 | 5 | 7 | 4 | 2 | 8 | 9 | 4 | 12 | 2 | 6 | 6 |
| 65 | 9 | 11 | 5 | 2 | 8 | 5 | 7 | 16 | 4 | 10 | 8 | 11 |
| 66 | 3 | 2 | 2 | 0 | 2 | 2 | 1 | 3 | 3 | 3 | 1 | 0 |
| 67 | 5 | 1 | 1 | 1 | 2 | 3 | 5 | 0 | 2 | 3 | 3 | 1 |
| 68 | 3 | 3 | 11 | 5 | 7 | 4 | 7 | 2 | 8 | 3 | 5 | 8 |
| 69 | 5 | 7 | 12 | 5 | 6 | 10 | 9 | 9 | 8 | 14 | 7 | 11 |
| 70 | 1 | 2 | 1 |  | 2 | 3 |  | 2 | 4 |  | 2 | 1 |
| 71 | 8 | 1 | 2 | 6 | 4 | 3 | 5 | 2 | 2 | 5 | 0 | 3 |
| 72 | 11 | 13 | 6 | 9 | 11 | 10 | 11 | 5 | 8 | 3 | 7 | 6 |
| 73 |  | 7 | 7 | 7 | 8 | 9 | 13 | 2 | 6 | 7 | 1 | 6 |
| 74 | 18 | 17 | 18 | 10 | 16 | 17 | 12 | 18 | 11 | 15 | 12 | 17 |
| 75 |  | 1 |  |  | 3 | 1 | 1 | 1 |  | 1 | 1 | 1 |
| 76 | 5 | 4 | 4 | 5 | 3 | 3 | 3 | 4 | 5 | 2 | 6 | 6 |
| 77 |  |  | 1 | 1 | 1 |  | 2 |  | 1 |  |  | 1 |
| 78 |  |  |  |  |  |  | 2 |  |  | 1 |  |  |
| 79 | 9 | 3 | 6 | 1 | 1 | 6 | 4 | 14 | 6 | 5 | 5 | 10 |
| 80 | 2 | 3 | 4 | 7 | 5 | 3 | 4 | 10 | 9 | 8 | 2 | 3 |
| 81 | 2 | 6 | 5 | 1 | 4 | 1 | 4 | 5 | 4 | 5 | 6 | 4 |
| 82 |  |  |  |  |  |  |  |  |  |  |  |  |
| 83 | 1 | 1 | 3 | 1 | 4 | 0 | 2 | 1 | 2 | 2 | 3 | 1 |
| 84 | 6 | 7 | 6 | 5 | 3 | 10 | 4 | 7 | 10 | 4 | 7 | 2 |
| 85 | 1 | 8 | 4 | 4 | 10 | 5 | 10 | 5 | 6 | 10 | 4 | 12 |
| 86 | 5 | 2 | 9 | 8 | 1 | 12 | 5 | 10 | 9 | 4 | 2 | 4 |
| 87 |  | 1 | 6 | 3 | 3 |  | 5 | 1 | 2 |  |  | 3 |
| 88 | 5 | 1 | 3 | 2 | 2 | 3 | 3 | 5 | 3 | 1 | 4 | 3 |
| 89 | 14 | 17 | 14 | 15 | 7 | 15 | 11 | 14 | 15 | 16 | 14 | 21 |
| 90 | 1 | 4 | 3 | 5 | 3 | 3 | 2 | 7 | 4 | 5 | 1 | 2 |
| 91 | 7 | 3 | 4 | 3 | 3 | 5 | 5 | 8 | 0 | 6 | 2 | 3 |
| 92 |  |  |  |  |  |  |  |  |  |  |  |  |
| 93 | 5 | 6 | 3 | 6 | 4 | 3 | 2 | 5 | 5 | 2 | 4 | 2 |
| 94 | 2 | 0 | 3 | 1 | 2 | 1 | 1 | 0 | 1 | 1 | 1 | 1 |
| 95 | 1 | 6 | 2 | 2 | 1 | 2 | 0 | 1 | 0 | 2 | 3 | 3 |
| 96 |  | 5 | 4 | 6 | 2 | 5 | 4 | 7 | 2 | 6 | 6 | 4 |
| 97 | 1 | 0 | 1 | 0 | 4 | 1 | 1 | 0 | 3 | 2 | 3 | 1 |
| 98 | 8 | 3 | 6 | 3 | 7 | 8 | 6 | 6 | 4 | 8 | 5 | 5 |
| 99 | 2 | 3 | 3 | 1 | 1 | 3 | 2 | 3 | 2 | 2 | 2 | 1 |
| 100 | 7 | 8 | 3 | 7 | 4 | 5 | 3 | 6 | 9 | 5 | 8 | 6 |
| 101 | 4 | 6 | 4 | 5 |  | 3 | 2 | 4 | 6 | 6 | 3 | 4 |
| 102 |  |  |  |  |  |  |  |  |  |  |  |  |
| 103 | 7 | 3 | 3 | 2 | 6 | 3 | 4 | 10 | 2 | 5 | 6 | 6 |
| 104 | 12 | 9 | 6 | 7 | 4 | 10 | 10 | 4 | 12 | 9 | 10 | 13 |
| 105 | 4 | 10 | 4 | 6 | 4 | 7 | 3 | 2 | 6 | 2 | 5 | 5 |

Table 14. Number of newly diagnosed stage I colorectal cancer during each month in 2020.

|  | Month | | | | | | | | | | | |
| --- | --- | --- | --- | --- | --- | --- | --- | --- | --- | --- | --- | --- |
| Hospital | 1 | 2 | 3 | 4 | 5 | 6 | 7 | 8 | 9 | 10 | 11 | 12 |
| 1 | 2 | 6 | 1 | 2 | 1 | 2 | 2 | 1 | 3 | 1 | 3 | 0 |
| 2 | 29 | 16 | 34 | 25 | 14 | 12 | 13 | 10 | 26 | 17 | 27 | 22 |
| 3 | 3 | 3 | 0 | 0 | 2 | 0 | 1 | 2 | 4 | 2 | 3 | 5 |
| 4 | 3 | 2 | 2 | 4 | 3 | 0 | 1 | 1 | 3 | 2 | 4 | 3 |
| 5 | 4 | 2 | 3 | 1 | 0 | 2 | 3 | 2 | 7 | 2 | 4 | 6 |
| 6 | 3 | 5 | 2 | 1 | 3 | 4 | 4 | 2 | 2 | 1 | 3 | 4 |
| 7 | 2 | 3 | 6 | 2 | 3 | 0 | 3 | 4 | 3 | 5 | 4 | 8 |
| 8 |  |  |  |  |  |  |  |  |  |  |  |  |
| 9 | 6 | 6 | 7 | 7 | 2 | 2 | 7 | 4 | 7 | 7 | 6 | 4 |
| 10 | 2 | 4 | 7 | 4 | 2 |  | 3 | 3 | 4 | 6 | 2 | 1 |
| 11 | 6 | 5 | 7 | 3 | 3 | 4 | 10 | 10 | 11 | 8 | 6 | 4 |
| 12 | 1 | 1 | 3 | 0 | 1 | 0 | 0 | 2 | 0 | 1 | 1 | 0 |
| 13 | 3 | 2 | 4 | 3 | 2 | 5 | 5 | 1 | 2 | 3 | 3 | 3 |
| 14 | 4 | 4 | 2 | 3 | 2 | 3 | 2 | 6 | 7 | 4 | 3 | 4 |
| 15 | 4 | 5 | 4 | 2 | 6 | 4 | 4 | 3 | 8 | 8 | 3 | 5 |
| 16 | 12 | 6 | 4 | 7 | 9 | 7 | 6 | 0 | 8 | 7 | 5 | 8 |
| 17 |  |  |  |  |  |  |  |  |  |  |  |  |
| 18 | 4 | 5 | 4 | 3 | 3 | 7 | 3 | 4 | 4 | 3 | 2 | 5 |
| 19 | 1 | 3 | 1 | 1 | 1 | 1 | 2 | 0 | 2 | 1 | 1 | 1 |
| 20 | 10 | 12 | 15 | 7 | 2 | 4 | 5 | 6 | 9 | 19 | 11 | 16 |
| 21 | 2 | 1 | 3 | 2 | 3 | 0 | 2 | 1 | 0 | 3 | 5 | 3 |
| 22 |  | 1 | 3 |  | 1 | 2 | 3 | 2 | 2 | 1 |  | 3 |
| 23 |  |  |  |  |  |  |  |  |  |  |  |  |
| 24 | 6 | 7 | 6 | 7 | 4 | 8 | 9 | 3 | 9 | 7 | 6 | 1 |
| 25 | 6 | 7 | 4 | 3 | 0 | 2 | 7 | 5 | 6 | 4 | 6 | 8 |
| 26 |  | 3 | 6 | 2 | 5 | 2 | 4 | 2 | 2 | 3 | 2 | 5 |
| 27 | 1 | 2 | 3 | 1 | 1 | 2 | 1 | 1 | 0 | 1 | 2 | 1 |
| 28 | 3 | 3 | 1 | 4 | 2 | 4 | 6 | 4 | 4 | 3 | 4 | 3 |
| 29 |  |  |  |  |  |  |  |  |  |  |  |  |
| 30 | 3 | 2 | 4 | 7 | 0 | 4 | 2 | 2 | 4 | 7 | 2 | 1 |
| 31 | 3 | 4 | 4 | 4 | 3 | 4 | 4 | 4 | 2 | 2 | 1 | 1 |
| 32 | 6 | 1 | 4 | 3 |  | 1 | 2 | 5 | 2 | 3 | 3 | 1 |
| 33 | 1 | 3 | 1 | 3 | 9 | 2 | 4 | 2 | 4 | 8 | 3 | 3 |
| 34 | 9 | 10 | 11 | 3 | 4 | 4 | 3 | 6 | 10 | 13 | 4 | 10 |
| 35 | 13 | 3 | 4 | 4 | 4 | 8 | 2 | 6 | 4 | 8 | 1 | 1 |
| 36 | 1 | 7 | 3 | 5 | 3 | 5 | 3 | 1 | 3 | 4 | 3 | 4 |
| 37 | 6 | 9 | 2 | 7 | 10 | 6 | 5 | 6 | 4 | 5 | 7 | 10 |
| 38 |  |  |  |  |  |  |  |  |  |  |  |  |
| 39 | 2 | 1 |  |  |  | 2 | 1 |  |  | 4 | 1 |  |
| 40 | 8 | 9 | 4 | 3 | 9 | 6 | 6 | 5 | 10 | 7 | 6 | 11 |
| 41 | 9 | 3 | 5 | 9 | 3 | 7 | 5 | 6 | 7 | 2 | 8 | 2 |
| 42 | 2 | 2 | 7 | 2 | 0 | 2 | 5 | 2 | 2 | 6 | 2 | 3 |
| 43 | 6 | 1 | 6 | 4 | 4 | 6 | 4 | 4 | 6 | 4 | 7 | 8 |
| 44 | 0 | 0 | 0 | 1 | 0 | 3 | 2 | 1 | 1 | 1 | 2 | 4 |
| 45 | 2 | 4 | 3 | 6 | 0 | 3 | 2 | 4 | 5 | 4 | 5 | 4 |
| 46 | 3 | 2 | 8 | 3 | 3 | 5 | 9 | 11 | 5 | 8 | 5 | 4 |
| 47 |  |  |  |  |  |  |  |  |  |  |  |  |
| 48 | 2 | 7 | 2 | 3 | 5 | 1 | 4 | 5 |  | 3 | 4 | 5 |
| 49 | 7 | 3 | 4 | 3 | 3 | 2 | 2 | 3 | 3 | 3 | 2 | 6 |
| 50 | 1 | 4 | 0 | 0 | 0 | 1 | 1 | 0 | 1 | 1 | 1 | 3 |
| 51 | 1 | 2 | 1 | 0 | 0 | 1 | 0 | 1 | 1 | 0 | 0 | 0 |
| 52 | 5 | 4 | 7 | 9 | 3 | 4 | 7 | 3 | 6 | 6 | 9 | 2 |
| 53 | 4 | 5 | 3 | 2 | 0 | 6 | 5 | 5 | 3 | 6 | 6 | 4 |
| 54 | 9 | 5 | 10 | 10 | 2 | 3 | 5 | 8 | 5 | 18 | 4 | 5 |
| 55 | 15 | 20 | 6 | 12 | 6 | 11 | 11 | 5 | 16 | 11 | 19 | 11 |
| 56 | 8 | 5 | 1 | 1 | 5 | 7 | 7 | 8 | 3 | 9 | 4 | 3 |
| 57 | 6 | 8 | 7 | 7 | 5 | 5 | 9 | 13 | 8 | 8 | 11 | 13 |
| 58 | 8 | 13 | 15 | 13 | 5 | 5 | 5 | 6 | 13 | 13 | 11 | 19 |
| 59 | 3 | 2 | 9 | 5 | 2 | 2 | 5 | 3 | 2 | 4 | 4 | 4 |
| 60 | 4 | 5 | 5 | 2 | 4 | 2 | 1 | 0 | 6 | 3 | 2 | 4 |
| 61 | 4 | 1 | 3 | 5 | 6 | 1 | 6 | 2 | 2 | 6 | 6 | 9 |
| 62 | 2 | 5 | 5 | 3 | 2 | 2 | 3 | 5 | 2 | 4 | 3 | 5 |
| 63 |  |  |  |  |  |  |  |  |  |  |  |  |
| 64 | 4 | 3 | 3 | 3 | 2 | 2 |  | 1 | 3 |  | 2 | 1 |
| 65 | 2 | 7 | 6 | 4 | 1 | 2 | 2 | 4 | 3 | 6 | 2 | 5 |
| 66 | 5 | 9 | 10 | 3 | 0 | 5 | 5 | 8 | 5 | 8 | 6 | 9 |
| 67 | 1 | 2 | 0 | 2 | 0 | 1 | 3 | 4 | 3 | 1 | 3 | 1 |
| 68 | 3 | 6 | 3 | 3 | 3 | 5 | 1 | 4 | 1 | 1 | 3 | 6 |
| 69 | 3 | 3 | 3 | 5 | 3 | 3 | 1 | 2 | 4 | 10 | 2 | 4 |
| 70 | 10 | 10 | 8 | 10 | 5 | 8 | 9 | 6 | 7 | 16 | 10 | 11 |
| 71 |  | 2 | 1 |  | 1 | 2 | 2 | 1 |  | 1 |  | 1 |
| 72 | 3 | 6 | 3 | 2 | 2 | 2 | 2 | 2 | 4 | 5 | 5 | 5 |
| 73 | 6 | 9 | 5 | 8 | 1 | 4 | 3 | 2 | 9 | 9 | 7 | 11 |
| 74 | 4 | 6 | 4 | 4 | 1 | 3 | 1 | 2 | 4 | 3 | 2 | 4 |
| 75 | 20 | 17 | 23 | 19 | 9 | 11 | 18 | 13 | 19 | 17 | 11 | 13 |
| 76 |  |  |  |  |  |  |  |  |  |  |  |  |
| 77 | 4 | 2 | 3 | 1 | 3 | 7 | 3 | 4 | 2 | 3 | 4 | 2 |
| 78 | 3 | 1 | 1 |  | 3 |  | 1 |  |  | 1 | 3 | 1 |
| 79 | 1 |  | 1 | 1 |  | 1 | 1 | 1 |  |  |  |  |
| 80 | 8 | 7 | 9 | 0 | 1 | 2 | 4 | 4 | 3 | 13 | 7 | 9 |
| 81 | 7 | 1 | 5 | 4 | 1 | 4 | 4 | 2 | 4 | 8 | 4 | 5 |
| 82 | 2 | 9 | 5 | 1 | 3 | 5 | 8 | 5 | 1 | 5 | 1 | 3 |
| 83 | 2 | 1 | 2 | 4 | 4 | 1 | 3 | 0 | 4 | 2 | 2 | 4 |
| 84 | 3 | 8 | 5 | 6 | 3 | 3 | 11 | 8 | 4 | 6 | 5 | 8 |
| 85 | 11 | 8 | 5 | 4 | 2 | 4 | 5 | 8 | 9 | 8 | 11 | 8 |
| 86 | 6 | 5 | 4 | 5 | 1 | 8 | 2 | 6 | 5 | 9 | 4 | 7 |
| 87 | 2 | 2 |  |  | 1 | 1 | 1 | 3 | 1 |  | 2 | 1 |
| 88 | 6 | 5 | 1 | 1 | 5 | 3 | 4 | 3 | 3 | 5 | 2 | 5 |
| 89 | 13 | 19 | 6 | 7 | 7 | 15 | 6 | 12 | 12 | 9 | 14 | 12 |
| 90 | 1 | 0 | 3 | 3 | 1 | 4 | 3 | 3 | 1 | 3 | 2 | 9 |
| 91 | 6 | 4 | 2 | 2 | 3 | 8 | 6 | 2 | 1 | 6 | 5 | 4 |
| 92 |  |  |  |  |  |  |  |  |  |  |  |  |
| 93 | 5 | 2 | 5 | 3 | 2 | 5 | 0 | 4 | 2 | 6 | 2 | 0 |
| 94 | 1 | 2 | 3 | 3 | 0 | 1 | 1 | 0 | 2 | 1 | 0 | 2 |
| 95 | 1 | 1 | 2 | 3 | 2 | 2 | 1 | 1 | 0 | 3 | 2 | 1 |
| 96 | 6 | 7 | 6 | 4 | 1 | 5 | 7 | 5 | 6 | 7 | 8 | 6 |
| 97 | 0 | 0 | 1 | 0 | 1 | 1 | 1 | 1 | 1 | 2 | 1 | 2 |
| 98 | 4 | 6 | 6 | 4 | 6 | 7 | 8 | 3 | 3 | 11 | 8 | 4 |
| 99 | 3 | 1 | 2 | 0 | 0 | 1 | 0 | 4 | 2 | 4 | 3 | 2 |
| 100 | 5 | 5 | 4 | 8 | 5 | 3 | 5 | 5 | 7 | 8 | 7 | 10 |
| 101 |  | 1 | 3 | 3 | 2 | 4 |  | 5 | 6 | 3 | 1 | 4 |
| 102 |  |  |  |  |  |  |  |  |  |  |  |  |
| 103 | 6 | 2 | 6 | 6 | 3 | 2 | 3 | 5 | 1 | 1 | 9 | 1 |
| 104 | 7 | 4 | 8 | 5 | 4 | 6 | 5 | 8 | 4 | 11 | 9 | 3 |
| 105 | 4 | 6 | 3 | 2 | 1 | 2 | 4 | 2 | 4 | 5 | 6 | 3 |

Table 15. Number of newly diagnosed stage II colorectal cancer during each month in 2019.

|  | Month | | | | | | | | | | | |
| --- | --- | --- | --- | --- | --- | --- | --- | --- | --- | --- | --- | --- |
| Hospital | 1 | 2 | 3 | 4 | 5 | 6 | 7 | 8 | 9 | 10 | 11 | 12 |
| 1 | 5 | 2 | 1 | 1 | 1 | 2 | 0 | 3 | 0 | 3 | 2 | 1 |
| 2 | 16 | 18 | 18 | 15 | 19 | 13 | 13 | 12 | 13 | 14 | 22 | 16 |
| 3 | 5 | 2 | 2 | 3 | 5 | 1 | 3 | 3 | 1 | 0 | 1 | 1 |
| 4 | 2 | 1 | 0 | 1 | 2 | 2 | 2 | 1 | 2 | 3 | 0 | 5 |
| 5 | 0 | 2 | 1 | 3 | 4 | 0 | 3 | 0 | 0 | 3 | 2 | 3 |
| 6 | 2 | 5 | 4 | 2 | 1 | 6 | 0 | 6 | 3 | 1 | 2 | 3 |
| 7 | 3 | 5 | 2 | 4 | 4 | 4 | 2 | 2 | 3 | 1 | 7 | 2 |
| 8 |  |  |  |  |  |  |  |  |  |  |  |  |
| 9 | 6 | 4 | 5 | 2 | 4 | 5 | 5 | 3 | 5 | 7 | 4 | 11 |
| 10 | 4 | 3 | 4 | 1 | 3 | 5 | 5 |  | 2 | 8 | 6 | 1 |
| 11 | 2 | 2 | 4 | 7 | 3 | 2 | 6 | 8 | 4 | 4 | 3 | 3 |
| 12 | 2 | 1 | 0 | 1 | 0 | 1 | 0 | 1 | 2 | 0 | 1 | 0 |
| 13 | 1 | 2 | 3 | 4 | 2 | 3 | 2 | 2 | 6 | 3 | 6 | 2 |
| 14 | 5 | 2 | 7 | 4 | 6 | 7 | 8 | 4 | 4 | 3 | 2 | 5 |
| 15 | 6 | 9 | 3 | 3 | 8 | 3 | 5 | 9 | 5 | 6 | 5 | 8 |
| 16 | 0 | 1 | 4 | 4 | 4 | 2 | 3 | 4 | 3 | 3 | 5 | 4 |
| 17 |  |  |  |  |  |  |  |  |  |  |  |  |
| 18 | 1 | 0 | 0 | 2 | 1 | 1 | 0 | 2 | 3 | 0 | 4 | 4 |
| 19 | 0 | 1 | 3 | 2 | 2 | 2 | 0 | 2 | 0 | 1 | 0 | 1 |
| 20 | 9 | 10 | 15 | 12 | 5 | 10 | 13 | 7 | 8 | 7 | 7 | 11 |
| 21 | 8 | 4 | 9 | 4 | 5 | 1 | 4 | 3 | 0 | 1 | 5 | 5 |
| 22 |  | 3 | 4 | 4 |  | 4 | 2 | 4 | 1 |  | 3 | 1 |
| 23 |  |  |  |  |  |  |  |  |  |  |  |  |
| 24 | 3 | 3 | 7 | 7 | 2 | 2 | 3 | 3 | 2 | 9 | 1 | 5 |
| 25 | 8 | 4 | 3 | 7 | 5 | 5 | 3 | 3 | 4 | 7 | 6 | 4 |
| 26 | 3 | 3 | 1 | 3 |  |  | 2 | 1 | 2 | 4 | 1 | 2 |
| 27 | 0 | 3 | 2 | 4 | 0 | 3 | 0 | 2 | 2 | 3 | 0 | 3 |
| 28 | 3 | 5 | 1 | 1 | 1 | 2 | 1 | 1 | 2 | 4 | 4 | 1 |
| 29 |  |  |  |  |  |  |  |  |  |  |  |  |
| 30 | 1 | 2 | 2 | 4 | 0 | 0 | 0 | 3 | 1 | 2 | 1 | 1 |
| 31 | 2 | 2 | 0 | 4 | 4 | 0 | 1 | 2 | 0 | 0 | 1 | 1 |
| 32 |  | 3 | 1 | 2 | 3 | 1 | 1 |  | 4 | 4 |  | 1 |
| 33 | 6 | 5 | 3 | 4 | 5 | 5 | 4 | 6 | 3 | 8 | 4 | 4 |
| 34 | 5 | 2 | 1 | 7 | 6 | 6 | 4 | 3 | 2 | 6 | 3 | 3 |
| 35 | 6 | 12 | 6 | 12 | 13 | 4 | 11 | 9 | 6 | 5 | 7 | 3 |
| 36 | 3 | 2 |  | 1 | 1 |  | 4 | 3 | 2 | 3 | 1 | 2 |
| 37 | 2 | 0 | 1 | 3 | 5 | 5 | 2 | 1 | 3 | 5 | 1 | 1 |
| 38 |  |  |  |  |  |  |  |  |  |  |  |  |
| 39 | 2 | 4 |  |  | 3 | 2 |  | 3 | 3 | 4 |  | 2 |
| 40 | 8 | 7 | 9 | 6 | 5 | 6 | 15 | 5 | 7 | 8 | 5 | 8 |
| 41 | 4 | 5 | 3 | 3 | 3 | 4 | 4 | 1 | 2 | 3 | 3 | 7 |
| 42 | 1 | 1 | 1 | 4 | 1 | 1 | 1 | 1 | 1 | 4 | 2 | 0 |
| 43 | 5 | 0 | 4 | 5 | 9 | 3 | 10 | 1 | 1 | 4 | 6 | 6 |
| 44 | 1 | 3 | 3 | 2 | 2 | 3 | 1 | 3 | 1 | 0 | 3 | 2 |
| 45 | 3 | 1 | 0 | 2 | 4 | 6 | 5 | 2 | 2 | 13 | 3 | 0 |
| 46 | 2 | 2 | 7 | 7 | 4 | 11 | 4 | 6 | 4 | 2 | 3 | 4 |
| 47 | 1 |  | 2 |  | 2 | 4 | 1 |  | 2 | 3 |  | 1 |
| 48 | 2 | 6 | 5 | 7 | 11 | 1 | 3 | 2 | 7 | 4 | 2 | 4 |
| 49 | 3 | 0 | 1 | 0 | 3 | 1 | 2 | 5 | 1 | 2 | 3 | 7 |
| 50 | 1 | 1 | 1 | 0 | 2 | 1 | 0 | 1 | 1 | 1 | 0 | 0 |
| 51 | 4 | 3 | 8 | 4 | 7 | 6 | 6 | 9 | 8 | 4 | 2 | 2 |
| 52 | 4 | 2 | 5 | 5 | 3 | 1 | 5 | 5 | 0 | 4 | 1 | 2 |
| 53 | 2 | 2 | 4 | 2 | 2 | 6 | 7 | 2 | 4 | 3 | 7 | 8 |
| 54 | 8 | 12 | 9 | 7 | 9 | 10 | 11 | 9 | 12 | 13 | 8 | 12 |
| 55 | 0 | 8 | 5 | 1 | 8 | 1 | 4 | 2 | 3 | 2 | 5 | 2 |
| 56 | 8 | 5 | 4 | 7 | 5 | 4 | 3 | 5 | 3 | 6 | 5 | 8 |
| 57 | 7 | 6 | 8 | 9 | 4 | 7 | 9 | 5 | 8 | 9 | 7 | 8 |
| 58 | 2 | 1 | 3 | 2 | 2 | 4 | 2 | 0 | 2 | 1 | 2 | 1 |
| 59 | 2 | 2 | 0 | 3 | 2 | 4 | 2 | 4 | 0 | 7 | 0 | 3 |
| 60 | 1 | 1 | 3 | 1 | 3 | 4 | 3 | 5 | 1 | 2 | 4 | 3 |
| 61 | 4 | 6 | 3 | 5 | 4 | 4 | 7 | 1 | 5 | 4 | 4 | 5 |
| 62 |  |  |  |  |  |  |  |  |  |  |  |  |
| 63 |  | 2 | 1 |  | 1 | 2 | 1 | 2 | 2 |  | 3 | 1 |
| 64 | 4 |  | 1 | 3 |  | 3 | 3 | 6 | 1 |  | 6 | 3 |
| 65 | 3 | 7 | 4 | 4 | 8 | 5 | 3 | 6 | 5 | 4 | 2 | 6 |
| 66 | 0 | 2 | 0 | 1 | 1 | 1 | 0 | 3 | 0 | 1 | 0 | 0 |
| 67 | 1 | 4 | 0 | 2 | 4 | 3 | 0 | 2 | 6 | 1 | 2 | 1 |
| 68 | 2 | 6 | 6 | 3 | 4 | 5 | 4 | 1 | 1 | 1 | 9 | 6 |
| 69 | 3 | 5 | 1 | 5 | 2 | 3 | 8 | 4 | 1 | 8 | 6 | 6 |
| 70 | 2 |  | 2 | 1 | 2 |  | 1 |  | 2 | 2 | 1 | 1 |
| 71 | 0 | 0 | 3 | 4 | 2 | 1 | 3 | 3 | 2 | 3 | 5 | 6 |
| 72 | 4 | 3 | 0 | 2 | 3 | 1 | 4 | 3 | 2 | 4 | 5 | 0 |
| 73 |  | 1 | 2 | 2 | 2 | 4 | 1 | 4 | 1 | 4 | 5 | 2 |
| 74 | 3 | 9 | 4 | 5 | 4 | 9 | 6 | 9 | 2 | 1 | 2 | 5 |
| 75 |  |  |  |  |  |  |  |  | 1 |  |  | 1 |
| 76 | 1 | 1 | 1 | 1 | 1 | 1 | 1 | 2 | 5 | 3 | 3 | 2 |
| 77 |  | 2 | 1 | 1 | 5 | 2 | 1 | 1 |  | 3 |  | 2 |
| 78 |  | 1 |  |  | 1 | 1 | 2 | 2 |  | 2 |  | 1 |
| 79 | 1 | 3 | 4 | 3 | 1 | 2 | 2 | 3 | 1 | 3 | 1 | 4 |
| 80 | 1 | 4 | 2 | 2 | 2 | 6 | 1 | 2 | 4 | 3 | 5 | 4 |
| 81 | 5 | 1 | 4 | 4 |  | 2 | 5 | 4 | 6 | 9 | 5 | 4 |
| 82 |  |  |  |  |  |  |  |  |  |  |  |  |
| 83 | 5 | 0 | 3 | 2 | 3 | 4 | 3 | 3 | 4 | 2 | 2 | 1 |
| 84 | 5 | 3 | 8 | 5 | 4 | 4 | 5 | 5 | 5 | 0 | 6 | 3 |
| 85 | 2 | 1 | 4 | 3 | 0 | 3 | 2 | 2 | 1 | 0 | 0 | 0 |
| 86 | 3 | 3 | 5 | 4 | 1 | 3 | 1 | 1 | 2 | 2 | 6 | 4 |
| 87 | 2 | 2 | 1 | 2 | 5 | 3 | 3 | 2 | 3 | 1 | 3 | 2 |
| 88 | 3 | 1 | 1 | 3 | 6 | 0 | 5 | 1 | 2 | 0 | 3 | 1 |
| 89 | 11 | 18 | 12 | 11 | 14 | 11 | 11 | 4 | 9 | 10 | 11 | 11 |
| 90 | 5 | 3 | 4 | 2 | 1 | 3 | 3 | 3 | 2 | 1 | 2 | 5 |
| 91 | 4 | 0 | 3 | 5 | 5 | 5 | 6 | 2 | 2 | 1 | 2 | 4 |
| 92 |  |  |  |  |  |  |  |  |  |  |  |  |
| 93 | 3 | 1 | 1 | 2 | 2 | 2 | 3 | 0 | 0 | 0 | 1 | 2 |
| 94 | 1 | 0 | 1 | 2 | 3 | 0 | 3 | 1 | 2 | 1 | 1 | 3 |
| 95 | 1 | 3 | 1 | 2 | 2 | 3 | 1 | 1 | 1 | 3 | 0 | 1 |
| 96 | 3 | 2 | 4 | 1 | 1 | 2 | 0 | 0 | 0 | 4 | 2 | 0 |
| 97 | 1 | 1 | 0 | 1 | 3 | 1 | 1 | 0 | 0 | 0 | 2 | 1 |
| 98 | 2 | 0 | 1 | 2 | 5 | 2 | 3 | 3 | 0 | 1 | 0 | 1 |
| 99 | 1 | 2 | 3 | 1 | 2 | 0 | 0 | 2 | 3 | 2 | 5 | 2 |
| 100 | 0 | 0 | 2 | 2 | 1 | 1 | 1 | 3 | 0 | 2 | 3 | 0 |
| 101 | 2 |  | 4 | 5 | 1 | 4 | 8 | 1 | 5 | 6 | 2 | 5 |
| 102 |  |  |  |  |  |  |  |  |  |  |  |  |
| 103 | 4 | 2 | 6 | 2 | 9 | 5 | 7 | 6 | 2 | 7 | 3 | 5 |
| 104 | 5 | 4 | 7 | 10 | 11 | 7 | 5 | 8 | 8 | 6 | 4 | 10 |
| 105 | 4 | 1 | 5 | 1 | 3 | 4 | 3 | 2 | 3 | 4 | 4 | 5 |

Table 16. Number of newly diagnosed stage II colorectal cancer during each month in 2020.

|  | Month | | | | | | | | | | | |
| --- | --- | --- | --- | --- | --- | --- | --- | --- | --- | --- | --- | --- |
| Hospital | 1 | 2 | 3 | 4 | 5 | 6 | 7 | 8 | 9 | 10 | 11 | 12 |
| 1 | 1 | 1 | 4 | 0 | 3 | 0 | 0 | 0 | 2 | 1 | 0 | 0 |
| 2 | 19 | 11 | 19 | 17 | 7 | 9 | 9 | 9 | 7 | 17 | 12 | 20 |
| 3 | 3 | 1 | 1 | 1 | 3 | 1 | 0 | 2 | 1 | 3 | 1 | 4 |
| 4 | 0 | 0 | 3 | 1 | 1 | 2 | 1 | 3 | 0 | 4 | 1 | 1 |
| 5 | 1 | 1 | 1 | 0 | 0 | 4 | 0 | 1 | 0 | 0 | 3 | 1 |
| 6 | 2 | 3 | 1 | 3 | 3 | 1 | 1 | 2 | 1 | 5 | 3 | 2 |
| 7 | 1 | 5 | 1 | 2 | 4 | 4 | 3 | 1 | 2 | 5 | 5 | 2 |
| 8 |  |  |  |  |  |  |  |  |  |  |  |  |
| 9 | 5 | 2 | 6 | 5 | 4 | 7 | 5 | 3 | 3 | 8 | 2 | 5 |
| 10 | 6 | 2 | 5 | 1 | 5 | 3 | 3 | 4 | 2 | 3 | 1 | 3 |
| 11 | 3 | 2 | 2 | 2 | 2 | 4 | 3 | 2 | 5 | 3 | 3 | 2 |
| 12 | 0 | 2 | 1 | 1 | 0 | 0 | 1 | 0 | 1 | 1 | 3 | 1 |
| 13 | 3 | 2 | 3 | 3 | 2 | 6 | 3 | 1 | 4 | 3 | 3 | 2 |
| 14 | 6 | 2 | 1 | 9 | 3 | 1 | 10 | 2 | 6 | 3 | 5 | 5 |
| 15 | 5 | 4 | 4 | 2 | 2 | 2 | 5 | 1 | 5 | 4 | 1 | 7 |
| 16 | 5 | 4 | 5 | 2 | 6 | 3 | 3 | 3 | 4 | 2 | 6 | 2 |
| 17 |  |  |  |  |  |  |  |  |  |  |  |  |
| 18 | 3 | 2 | 6 | 3 | 2 | 3 | 2 | 4 | 3 | 4 | 4 | 6 |
| 19 | 2 | 0 | 5 | 1 | 0 | 3 | 2 | 3 | 1 | 1 | 0 | 0 |
| 20 | 5 | 3 | 11 | 1 | 4 | 8 | 4 | 5 | 5 | 11 | 6 | 9 |
| 21 | 4 | 3 | 2 | 2 | 4 | 0 | 6 | 6 | 3 | 5 | 4 | 1 |
| 22 | 3 | 4 | 6 | 2 |  | 1 | 1 |  | 1 | 1 | 2 | 3 |
| 23 |  |  |  |  |  |  |  |  |  |  |  |  |
| 24 | 3 | 3 | 6 | 4 | 6 | 6 | 4 | 2 | 7 | 5 | 2 | 5 |
| 25 | 2 | 7 | 4 | 6 | 10 | 7 | 6 | 3 | 7 | 2 | 1 | 4 |
| 26 | 1 | 3 | 2 |  |  | 4 |  | 3 | 2 | 1 | 3 | 2 |
| 27 | 0 | 0 | 1 | 0 | 1 | 2 | 1 | 2 | 1 | 2 | 3 | 5 |
| 28 | 0 | 1 | 5 | 1 | 0 | 1 | 1 | 1 | 3 | 1 | 2 | 2 |
| 29 |  |  |  |  |  |  |  |  |  |  |  |  |
| 30 | 1 | 0 | 2 | 1 | 0 | 3 | 2 | 2 | 2 | 2 | 2 | 2 |
| 31 | 1 | 1 | 5 | 0 | 1 | 3 | 3 | 1 | 3 | 3 | 3 | 2 |
| 32 |  | 1 |  |  |  | 1 |  | 1 | 5 | 1 | 2 | 2 |
| 33 | 4 | 3 | 2 | 6 | 8 | 3 | 6 | 3 | 0 | 4 | 5 | 1 |
| 34 | 3 | 2 | 4 | 2 | 2 | 3 | 5 | 4 | 2 | 4 | 2 | 2 |
| 35 | 7 | 4 | 9 | 7 | 6 | 4 | 7 | 3 | 3 | 6 | 5 | 5 |
| 36 | 2 |  | 4 | 2 | 4 | 2 | 3 | 3 | 4 | 2 | 1 | 2 |
| 37 | 1 | 9 | 6 | 7 | 8 | 5 | 2 | 2 | 6 | 2 | 3 | 7 |
| 38 |  |  |  |  |  |  |  |  |  |  |  |  |
| 39 | 1 | 2 | 3 | 1 | 2 |  | 1 | 1 | 1 |  | 1 |  |
| 40 | 10 | 4 | 6 | 7 | 2 | 14 | 7 | 6 | 9 | 8 | 5 | 5 |
| 41 | 3 | 5 | 2 | 6 | 3 | 3 | 6 | 2 | 7 | 3 | 3 | 1 |
| 42 | 0 | 3 | 5 | 3 | 1 | 3 | 9 | 2 | 9 | 2 | 1 | 1 |
| 43 | 1 | 4 | 3 | 3 | 2 | 4 | 4 | 7 | 2 | 3 | 2 | 4 |
| 44 | 3 | 1 | 1 | 0 | 2 | 2 | 3 | 0 | 0 | 1 | 1 | 5 |
| 45 | 5 | 5 | 4 | 1 | 1 | 4 | 5 | 2 | 4 | 4 | 2 | 4 |
| 46 | 4 | 7 | 9 | 6 | 3 | 5 | 7 | 3 | 5 | 6 | 6 | 10 |
| 47 |  | 1 | 1 | 2 |  | 3 | 1 | 2 |  |  | 2 | 6 |
| 48 | 2 | 2 | 3 | 4 | 2 | 4 | 7 | 4 | 4 | 6 | 2 | 1 |
| 49 | 2 | 1 | 1 | 1 | 2 | 3 | 1 | 1 | 2 | 1 | 3 | 0 |
| 50 | 1 | 1 | 0 | 0 | 2 | 1 | 0 | 0 | 1 | 1 | 0 | 0 |
| 51 | 3 | 9 | 5 | 2 | 4 | 4 | 3 | 2 | 6 | 4 | 6 | 11 |
| 52 | 0 | 3 | 2 | 6 | 0 | 2 | 3 | 3 | 4 | 5 | 7 | 5 |
| 53 | 4 | 4 | 6 | 1 | 2 | 3 | 3 | 7 | 1 | 7 | 3 | 3 |
| 54 | 9 | 2 | 9 | 8 | 7 | 5 | 9 | 8 | 8 | 17 | 13 | 7 |
| 55 | 3 | 4 | 3 | 0 | 1 | 5 | 0 | 1 | 7 | 3 | 0 | 4 |
| 56 | 5 | 7 | 7 | 3 | 1 | 5 | 3 | 3 | 3 | 4 | 6 | 1 |
| 57 | 9 | 11 | 4 | 5 | 2 | 5 | 5 | 3 | 2 | 4 | 8 | 6 |
| 58 | 0 | 1 | 2 | 1 | 1 | 2 | 2 | 2 | 2 | 3 | 4 | 3 |
| 59 | 3 | 1 | 2 | 3 | 1 | 0 | 0 | 1 | 1 | 4 | 3 | 1 |
| 60 | 2 | 4 | 3 | 3 | 4 | 4 | 3 | 5 | 0 | 3 | 3 | 4 |
| 61 | 1 | 6 | 5 | 0 | 5 | 4 | 3 | 2 | 2 | 8 | 2 | 2 |
| 62 |  |  |  |  |  |  |  |  |  |  |  |  |
| 63 | 2 | 2 |  |  | 3 | 1 | 2 | 2 | 2 | 2 | 1 | 1 |
| 64 | 1 | 1 | 3 | 2 | 2 | 2 | 2 | 1 | 2 | 2 | 1 | 4 |
| 65 | 11 | 5 | 5 | 2 | 5 | 5 | 10 | 5 | 5 | 4 | 3 | 13 |
| 66 | 0 | 1 | 0 | 0 | 1 | 1 | 0 | 2 | 2 | 0 | 1 | 0 |
| 67 | 1 | 1 | 2 | 5 | 1 | 1 | 2 | 2 | 1 | 4 | 7 | 4 |
| 68 | 2 | 2 | 6 | 4 | 2 | 8 | 4 | 1 | 5 | 5 | 4 | 6 |
| 69 | 3 | 4 | 5 | 5 | 2 | 2 | 8 | 4 | 1 | 10 | 3 | 4 |
| 70 | 1 |  |  |  |  | 1 | 3 |  |  | 1 |  | 1 |
| 71 | 4 | 2 | 2 | 3 | 1 | 2 | 1 | 2 | 3 | 1 | 6 | 1 |
| 72 | 3 | 0 | 5 | 0 | 2 | 1 | 1 | 2 | 1 | 7 | 4 | 10 |
| 73 | 1 | 2 | 3 | 1 | 2 | 2 | 2 | 2 | 4 | 5 | 3 | 7 |
| 74 | 9 | 6 | 3 | 4 | 3 | 1 | 8 | 1 | 7 | 4 | 4 | 1 |
| 75 |  |  |  | 1 |  |  | 1 |  |  |  |  |  |
| 76 | 5 | 2 | 1 | 3 | 1 | 0 | 1 | 2 | 2 | 5 | 5 | 3 |
| 77 |  |  |  | 1 |  | 1 | 2 | 2 | 2 |  |  | 2 |
| 78 |  |  |  |  |  |  |  | 1 |  |  | 2 |  |
| 79 | 1 | 1 | 1 | 0 | 1 | 1 | 4 | 1 | 0 | 0 | 2 | 3 |
| 80 | 1 | 0 | 3 | 2 | 1 | 2 | 4 | 3 | 1 | 4 | 3 | 1 |
| 81 | 6 | 4 | 7 |  | 3 | 5 | 1 | 4 | 6 | 5 | 2 | 5 |
| 82 |  |  |  |  |  |  |  |  |  |  |  |  |
| 83 | 3 | 0 | 3 | 2 | 0 | 2 | 1 | 1 | 1 | 1 | 3 | 2 |
| 84 | 9 | 3 | 1 | 2 | 3 | 4 | 6 | 0 | 1 | 3 | 4 | 5 |
| 85 | 2 | 1 | 0 | 1 | 0 | 0 | 1 | 3 | 5 | 2 | 2 | 6 |
| 86 | 5 | 5 | 7 | 3 | 2 | 6 | 7 | 1 | 4 | 3 | 6 | 9 |
| 87 | 2 | 3 | 1 | 2 | 1 | 3 | 1 | 3 | 3 |  |  | 3 |
| 88 | 1 | 1 | 2 | 3 | 1 | 2 | 1 | 2 | 5 | 1 | 2 | 7 |
| 89 | 6 | 4 | 10 | 7 | 4 | 9 | 5 | 7 | 5 | 10 | 7 | 8 |
| 90 | 3 | 1 | 1 | 2 | 2 | 2 | 4 | 3 | 2 | 0 | 2 | 5 |
| 91 | 2 | 2 | 2 | 1 | 2 | 0 | 2 | 0 | 4 | 1 | 2 | 2 |
| 92 |  |  |  |  |  |  |  |  |  |  |  |  |
| 93 | 2 | 1 | 4 | 0 | 1 | 0 | 0 | 1 | 0 | 0 | 2 | 4 |
| 94 | 0 | 1 | 3 | 1 | 0 | 1 | 0 | 1 | 0 | 5 | 2 | 2 |
| 95 | 1 | 1 | 0 | 3 | 4 | 1 | 1 | 3 | 2 | 3 | 1 | 2 |
| 96 | 2 | 1 | 2 | 0 | 1 | 1 | 0 | 2 | 4 | 3 | 1 | 2 |
| 97 | 1 | 2 | 1 | 1 | 1 | 0 | 0 | 1 | 0 | 0 | 0 | 2 |
| 98 | 1 | 1 | 1 | 2 | 2 | 3 | 2 | 1 | 2 | 2 | 0 | 2 |
| 99 | 4 | 3 | 3 | 4 | 0 | 2 | 5 | 2 | 3 | 2 | 0 | 3 |
| 100 | 3 | 0 | 0 | 0 | 0 | 1 | 2 | 1 | 0 | 2 | 0 | 2 |
| 101 | 6 | 2 | 3 | 2 | 3 | 1 | 5 | 4 | 2 | 2 | 10 | 1 |
| 102 |  |  |  |  |  |  |  |  |  |  |  |  |
| 103 | 5 | 3 | 6 | 6 | 7 | 5 | 5 | 6 | 2 | 4 | 5 | 3 |
| 104 | 3 | 6 | 8 | 7 | 4 | 6 | 9 | 5 | 5 | 2 | 10 | 6 |
| 105 | 1 | 0 | 2 | 3 | 6 | 5 | 3 | 2 | 2 | 5 | 6 | 5 |

Table 17. Number of newly diagnosed stage III colorectal cancer during each month in 2019.

|  | Month | | | | | | | | | | | |
| --- | --- | --- | --- | --- | --- | --- | --- | --- | --- | --- | --- | --- |
| Hospital | 1 | 2 | 3 | 4 | 5 | 6 | 7 | 8 | 9 | 10 | 11 | 12 |
| 1 | 3 | 1 | 2 | 1 | 0 | 2 | 2 | 3 | 0 | 4 | 2 | 1 |
| 2 | 22 | 29 | 22 | 22 | 13 | 23 | 11 | 19 | 15 | 10 | 18 | 15 |
| 3 | 0 | 3 | 0 | 0 | 3 | 2 | 6 | 2 | 5 | 5 | 1 | 4 |
| 4 | 4 | 6 | 5 | 5 | 5 | 5 | 5 | 6 | 6 | 7 | 4 | 4 |
| 5 | 2 | 3 | 4 | 1 | 2 | 4 | 5 | 6 | 5 | 3 | 3 | 4 |
| 6 | 1 | 4 | 3 | 3 | 5 | 4 | 4 | 4 | 4 | 6 | 2 | 3 |
| 7 | 5 | 10 | 6 | 0 | 3 | 3 | 1 | 1 | 3 | 5 | 4 | 5 |
| 8 |  |  |  |  |  |  |  |  |  |  |  |  |
| 9 | 4 | 3 | 3 | 4 | 6 | 6 | 7 | 0 | 6 | 5 | 7 | 3 |
| 10 | 3 | 3 | 2 | 4 | 3 | 1 | 4 | 3 |  | 4 | 3 | 1 |
| 11 | 1 | 6 | 3 | 2 | 6 | 9 | 8 | 3 | 4 | 6 | 5 | 0 |
| 12 | 2 | 2 | 1 | 1 | 2 | 2 | 0 | 1 | 1 | 1 | 2 | 0 |
| 13 | 6 | 13 | 6 | 7 | 2 | 6 | 5 | 7 | 3 | 3 | 2 | 5 |
| 14 | 8 | 3 | 5 | 5 | 4 | 6 | 5 | 4 | 4 | 7 | 3 | 6 |
| 15 | 4 | 9 | 3 | 9 | 5 | 2 | 7 | 2 | 7 | 5 | 7 | 4 |
| 16 | 8 | 3 | 4 | 4 | 1 | 3 | 3 | 3 | 3 | 4 | 0 | 4 |
| 17 |  |  |  |  |  |  |  |  |  |  |  |  |
| 18 | 1 | 1 | 1 | 4 | 1 | 2 | 2 | 1 | 2 | 3 | 3 | 6 |
| 19 | 4 | 2 | 4 | 0 | 2 | 1 | 1 | 5 | 1 | 1 | 3 | 6 |
| 20 | 7 | 5 | 7 | 4 | 8 | 8 | 8 | 10 | 12 | 14 | 13 | 2 |
| 21 | 0 | 1 | 0 | 2 | 2 | 5 | 4 | 6 | 4 | 5 | 4 | 2 |
| 22 | 1 | 1 | 4 |  | 2 | 2 | 2 | 4 | 4 | 3 | 3 | 3 |
| 23 |  |  |  |  |  |  |  |  |  |  |  |  |
| 24 | 1 | 6 | 7 | 3 | 1 | 2 | 7 | 5 | 4 | 10 | 10 | 3 |
| 25 | 4 | 3 | 15 | 11 | 7 | 3 | 9 | 9 | 9 | 8 | 9 | 7 |
| 26 | 2 | 3 | 1 | 3 |  | 1 | 2 | 2 | 3 | 1 | 3 | 1 |
| 27 | 2 | 0 | 0 | 3 | 1 | 1 | 2 | 1 | 1 | 1 | 2 | 2 |
| 28 | 1 | 5 | 4 | 0 | 3 | 5 | 3 | 2 | 1 | 1 | 5 | 4 |
| 29 |  |  |  |  |  |  |  |  |  |  |  |  |
| 30 | 2 | 2 | 4 | 1 | 2 | 2 | 3 | 6 | 5 | 1 | 4 | 2 |
| 31 | 6 | 2 | 3 | 3 | 4 | 4 | 3 | 2 | 3 | 5 | 2 | 1 |
| 32 | 1 | 1 |  |  | 1 | 2 |  | 1 |  | 3 |  | 2 |
| 33 | 4 | 4 | 3 | 1 | 5 | 1 | 1 | 0 | 1 | 2 | 2 | 5 |
| 34 | 2 | 2 | 4 | 1 | 5 | 3 | 4 | 4 | 3 | 3 | 2 | 0 |
| 35 | 4 | 11 | 5 | 6 | 6 | 1 | 2 | 4 | 2 | 10 | 5 | 2 |
| 36 | 1 |  | 2 | 2 | 2 | 1 | 2 | 2 | 4 | 2 | 4 | 6 |
| 37 | 2 | 8 | 6 | 4 | 3 | 6 | 3 | 6 | 9 | 5 | 3 | 5 |
| 38 |  |  |  |  |  |  |  |  |  |  |  |  |
| 39 | 1 | 1 | 2 |  | 1 | 2 | 1 | 2 | 2 | 2 |  | 1 |
| 40 | 3 | 5 | 2 | 2 | 3 | 1 |  | 1 | 2 | 2 | 1 | 2 |
| 41 | 2 | 3 | 12 | 6 | 7 | 4 | 7 | 2 | 4 | 4 | 9 | 11 |
| 42 | 3 | 3 | 3 | 1 | 2 | 3 | 7 | 2 | 1 | 2 | 3 | 6 |
| 43 | 7 | 1 | 7 | 3 | 6 | 7 | 5 | 4 | 6 | 4 | 4 | 3 |
| 44 | 1 | 2 | 1 | 3 | 0 | 3 | 3 | 2 | 0 | 1 | 0 | 0 |
| 45 | 5 | 1 | 2 | 9 | 3 | 5 | 8 | 0 | 2 | 5 | 5 | 6 |
| 46 | 5 | 6 | 8 | 4 | 5 | 2 | 8 | 7 | 5 | 6 | 8 | 4 |
| 47 | 1 |  | 2 |  | 2 |  | 3 | 2 | 3 | 3 | 2 |  |
| 48 | 3 | 2 | 8 | 6 | 5 | 4 | 4 | 8 | 2 | 3 | 4 | 3 |
| 49 | 1 | 3 | 4 | 4 | 5 | 2 | 3 | 2 | 1 | 2 | 2 | 5 |
| 50 | 0 | 1 | 0 | 0 | 1 | 2 | 4 | 1 | 0 | 1 | 1 | 2 |
| 51 | 6 | 8 | 2 | 10 | 5 | 4 | 5 | 8 | 5 | 5 | 1 | 5 |
| 52 | 4 | 3 | 4 | 3 | 5 | 2 | 2 | 2 | 3 | 6 | 6 | 7 |
| 53 | 4 | 5 | 4 | 8 | 7 | 4 | 3 | 9 | 7 | 5 | 9 | 9 |
| 54 | 24 | 19 | 12 | 17 | 22 | 11 | 23 | 13 | 20 | 20 | 30 | 27 |
| 55 | 4 | 5 | 3 | 3 | 6 | 6 | 2 | 2 | 3 | 5 | 3 | 6 |
| 56 | 3 | 4 | 2 | 2 | 5 | 2 | 6 | 4 | 8 | 3 | 2 | 8 |
| 57 | 16 | 14 | 13 | 9 | 11 | 6 | 10 | 2 | 6 | 12 | 6 | 11 |
| 58 | 7 | 3 | 4 | 3 | 2 | 6 | 2 | 0 | 8 | 2 | 1 | 6 |
| 59 | 3 | 1 | 2 | 1 | 5 | 0 | 3 | 2 | 2 | 3 | 1 | 3 |
| 60 | 2 | 3 | 6 | 7 | 1 | 3 | 3 | 7 | 4 | 3 | 3 | 4 |
| 61 | 1 | 1 | 2 | 0 | 1 | 7 | 0 | 3 | 2 | 3 | 6 | 6 |
| 62 |  |  |  |  |  |  |  |  |  |  |  |  |
| 63 | 4 | 1 | 4 | 2 | 1 |  | 3 | 2 | 4 | 4 | 2 | 3 |
| 64 | 1 | 3 | 2 | 2 | 2 | 5 | 4 | 6 | 5 | 3 | 2 | 3 |
| 65 | 6 | 5 | 7 | 4 | 4 | 1 | 6 | 8 | 5 | 4 | 9 | 6 |
| 66 | 1 | 0 | 1 | 1 | 2 | 1 | 1 | 1 | 3 | 3 | 0 | 2 |
| 67 | 2 | 1 | 2 | 4 | 0 | 1 | 1 | 1 | 2 | 3 | 1 | 0 |
| 68 | 7 | 5 | 6 | 2 | 7 | 11 | 5 | 4 | 3 | 3 | 5 | 4 |
| 69 | 5 | 11 | 14 | 15 | 5 | 7 | 7 | 7 | 4 | 9 | 7 | 5 |
| 70 |  |  |  | 2 | 1 | 3 |  |  | 2 |  | 1 |  |
| 71 | 0 | 2 | 5 | 3 | 2 | 7 | 1 | 4 | 1 | 4 | 2 | 1 |
| 72 | 8 | 6 | 11 | 4 | 4 | 4 | 5 | 4 | 8 | 6 | 6 | 7 |
| 73 |  | 1 | 3 | 2 | 5 | 3 | 5 | 4 | 4 | 2 | 6 | 3 |
| 74 | 6 | 3 | 6 | 7 | 4 | 8 | 3 | 4 | 6 | 8 | 8 | 10 |
| 75 | 1 |  |  |  |  |  |  |  | 1 |  |  |  |
| 76 | 3 | 2 | 1 | 5 | 3 | 5 | 4 | 3 | 7 | 5 | 0 | 4 |
| 77 |  | 1 |  | 2 |  | 2 |  | 1 | 1 |  | 1 |  |
| 78 | 1 | 1 | 2 | 2 | 2 | 1 | 2 |  | 1 |  |  | 1 |
| 79 | 5 | 6 | 3 | 8 | 7 | 3 | 3 | 6 | 7 | 7 | 8 | 11 |
| 80 | 2 | 4 | 4 | 3 | 5 | 4 | 6 | 6 | 4 | 2 | 5 | 3 |
| 81 |  | 8 | 3 | 5 |  | 4 | 5 | 3 | 4 | 8 | 4 | 5 |
| 82 |  |  |  |  |  |  |  |  |  |  |  |  |
| 83 | 4 | 5 | 7 | 6 | 1 | 3 | 3 | 9 | 7 | 5 | 3 | 9 |
| 84 | 3 | 8 | 4 | 5 | 7 | 1 | 3 | 6 | 4 | 6 | 5 | 3 |
| 85 | 4 | 0 | 4 | 6 | 0 | 1 | 3 | 2 | 4 | 1 | 2 | 0 |
| 86 | 7 | 9 | 9 | 8 | 8 | 17 | 4 | 9 | 12 | 6 | 5 | 8 |
| 87 | 1 | 1 | 1 |  | 7 |  | 1 | 2 | 1 | 1 | 1 | 4 |
| 88 | 3 | 4 | 3 | 3 | 7 | 3 | 9 | 3 | 3 | 5 | 8 | 6 |
| 89 | 14 | 14 | 15 | 6 | 9 | 12 | 5 | 10 | 14 | 7 | 7 | 10 |
| 90 | 4 | 2 | 6 | 4 | 7 | 5 | 7 | 5 | 4 | 5 | 7 | 3 |
| 91 | 1 | 3 | 1 | 3 | 3 | 3 | 5 | 2 | 3 | 5 | 1 | 4 |
| 92 |  |  |  |  |  |  |  |  |  |  |  |  |
| 93 | 2 | 1 | 1 | 4 | 1 | 1 | 0 | 0 | 0 | 1 | 2 | 1 |
| 94 | 3 | 3 | 2 | 0 | 4 | 2 | 1 | 2 | 5 | 0 | 1 | 0 |
| 95 | 2 | 3 | 1 | 0 | 3 | 0 | 3 | 3 | 2 | 1 | 1 | 0 |
| 96 | 5 | 6 | 4 | 3 | 5 | 6 | 6 | 3 | 3 | 3 | 1 | 0 |
| 97 | 2 | 0 | 1 | 0 | 1 | 0 | 4 | 1 | 0 | 1 | 1 | 1 |
| 98 | 0 | 1 | 2 | 1 | 0 | 2 | 0 | 1 | 1 | 0 | 2 | 2 |
| 99 | 3 | 4 | 2 | 2 | 3 | 3 | 3 | 2 | 3 | 1 | 3 | 3 |
| 100 | 1 | 0 | 2 | 5 | 0 | 4 | 6 | 5 | 2 | 3 | 4 | 1 |
| 101 | 3 | 5 | 1 | 2 | 2 | 3 | 5 | 2 | 4 | 1 | 2 | 2 |
| 102 |  |  |  |  |  |  |  |  |  |  |  |  |
| 103 | 1 | 4 | 4 | 4 | 5 | 5 | 6 | 4 | 5 | 1 | 4 | 4 |
| 104 | 3 | 6 | 3 | 5 | 4 | 4 | 5 | 6 | 5 | 3 | 2 | 4 |
| 105 | 3 | 5 | 5 | 3 | 4 | 3 | 6 | 3 | 6 | 2 | 6 | 6 |

Table 18. Number of newly diagnosed stage III colorectal cancer during each month in 2020.

|  | Month | | | | | | | | | | | |
| --- | --- | --- | --- | --- | --- | --- | --- | --- | --- | --- | --- | --- |
| Hospital | 1 | 2 | 3 | 4 | 5 | 6 | 7 | 8 | 9 | 10 | 11 | 12 |
| 1 | 0 | 4 | 2 | 2 | 3 | 0 | 3 | 0 | 1 | 1 | 0 | 3 |
| 2 | 17 | 15 | 17 | 18 | 8 | 11 | 8 | 8 | 19 | 17 | 20 | 18 |
| 3 | 4 | 5 | 3 | 4 | 3 | 7 | 2 | 2 | 2 | 5 | 3 | 2 |
| 4 | 3 | 6 | 4 | 5 | 3 | 4 | 4 | 1 | 4 | 5 | 2 | 2 |
| 5 | 5 | 1 | 0 | 1 | 2 | 4 | 2 | 0 | 2 | 4 | 6 | 7 |
| 6 | 3 | 5 | 5 | 3 | 1 | 5 | 0 | 0 | 0 | 8 | 4 | 3 |
| 7 | 2 | 2 | 3 | 3 | 1 | 4 | 5 | 2 | 0 | 6 | 3 | 7 |
| 8 |  |  |  |  |  |  |  |  |  |  |  |  |
| 9 | 3 | 3 | 3 | 3 | 4 | 7 | 7 | 1 | 5 | 1 | 2 | 5 |
| 10 | 5 | 7 | 3 | 3 |  | 5 | 2 | 3 | 5 | 5 | 4 | 2 |
| 11 | 2 | 3 | 3 | 0 | 4 | 3 | 5 | 8 | 5 | 2 | 4 | 7 |
| 12 | 1 | 1 | 2 | 3 | 1 | 3 | 0 | 1 | 2 | 1 | 1 | 0 |
| 13 | 6 | 8 | 7 | 5 | 4 | 5 | 3 | 3 | 6 | 6 | 4 | 5 |
| 14 | 3 | 2 | 6 | 3 | 4 | 6 | 4 | 6 | 7 | 3 | 5 | 4 |
| 15 | 3 | 6 | 9 | 5 | 1 | 3 | 6 | 7 | 5 | 7 | 9 | 8 |
| 16 | 6 | 3 | 4 | 8 | 3 | 3 | 7 | 1 | 7 | 1 | 5 | 4 |
| 17 |  |  |  |  |  | 1 |  |  |  |  |  |  |
| 18 | 1 | 4 | 1 | 3 | 2 | 4 | 2 | 1 | 3 | 1 | 1 | 5 |
| 19 | 2 | 0 | 4 | 2 | 2 | 1 | 5 | 1 | 2 | 1 | 2 | 2 |
| 20 | 7 | 11 | 8 | 4 | 2 | 7 | 9 | 7 | 8 | 11 | 3 | 5 |
| 21 | 3 | 5 | 0 | 4 | 0 | 3 | 6 | 3 | 7 | 7 | 4 | 2 |
| 22 | 3 | 8 | 3 | 1 | 1 | 2 | 1 |  | 3 | 4 | 2 | 1 |
| 23 |  |  |  |  |  |  |  |  |  |  |  |  |
| 24 | 9 | 4 | 4 | 6 | 3 | 5 | 10 | 4 | 6 | 7 | 3 | 9 |
| 25 | 7 | 6 | 3 | 5 | 2 | 8 | 3 | 1 | 5 | 10 | 6 | 11 |
| 26 | 3 | 2 | 2 | 2 | 2 | 1 | 2 | 1 | 1 | 1 | 2 | 4 |
| 27 | 2 | 2 | 3 | 0 | 1 | 3 | 2 | 1 | 4 | 2 | 0 | 1 |
| 28 | 2 | 2 | 2 | 2 | 2 | 2 | 3 | 1 | 3 | 3 | 5 | 1 |
| 29 |  |  |  |  |  |  |  |  |  |  |  |  |
| 30 | 2 | 3 | 5 | 3 | 3 | 2 | 4 | 1 | 0 | 2 | 1 | 2 |
| 31 | 3 | 3 | 3 | 2 | 2 | 1 | 3 | 2 | 3 | 2 | 4 | 4 |
| 32 | 3 | 1 | 5 |  | 1 |  |  |  | 2 |  | 1 |  |
| 33 | 2 | 4 | 1 | 1 | 4 | 3 | 4 | 0 | 0 | 3 | 2 | 3 |
| 34 | 2 | 2 | 2 | 1 | 0 | 1 | 1 | 3 | 6 | 3 | 3 | 2 |
| 35 | 5 | 9 | 8 | 2 | 5 | 6 | 4 | 5 | 5 | 8 | 5 | 6 |
| 36 | 3 |  | 2 | 4 | 1 |  | 1 |  | 3 | 5 | 1 | 4 |
| 37 | 4 | 5 | 3 | 2 | 3 | 3 | 4 | 3 | 5 | 4 | 8 | 5 |
| 38 |  |  |  |  |  |  |  |  |  |  |  |  |
| 39 |  |  | 1 |  | 3 | 1 | 3 | 2 | 1 | 1 |  | 4 |
| 40 | 1 | 3 | 1 | 2 | 2 | 2 |  | 3 | 6 | 3 | 3 | 2 |
| 41 | 7 | 6 | 6 | 6 | 6 | 7 | 14 | 6 | 9 | 5 | 10 | 8 |
| 42 | 1 | 1 | 4 | 4 | 1 | 2 | 2 | 2 | 1 | 5 | 2 | 3 |
| 43 | 3 | 3 | 4 | 2 | 2 | 4 | 4 | 4 | 1 | 3 | 5 | 3 |
| 44 | 2 | 2 | 2 | 0 | 0 | 1 | 0 | 1 | 1 | 0 | 3 | 4 |
| 45 | 3 | 5 | 2 | 3 | 4 | 6 | 3 | 3 | 5 | 6 | 4 | 7 |
| 46 | 4 | 4 | 2 | 5 | 1 | 10 | 7 | 2 | 6 | 5 | 4 | 4 |
| 47 |  | 1 | 3 | 1 | 1 |  | 4 | 4 | 2 | 1 | 1 | 3 |
| 48 | 2 | 6 | 2 | 2 | 8 | 5 | 5 | 0 | 15 | 4 | 2 | 2 |
| 49 | 2 | 0 | 2 | 2 | 1 | 3 | 2 | 3 | 1 | 2 | 4 | 1 |
| 50 | 1 | 0 | 1 | 1 | 0 | 1 | 1 | 2 | 1 | 1 | 3 | 3 |
| 51 | 3 | 3 | 7 | 2 | 3 | 8 | 6 | 8 | 4 | 8 | 7 | 8 |
| 52 | 0 | 3 | 3 | 6 | 2 | 3 | 1 | 2 | 0 | 8 | 4 | 7 |
| 53 | 6 | 1 | 12 | 2 | 3 | 2 | 7 | 6 | 7 | 5 | 6 | 4 |
| 54 | 15 | 18 | 12 | 12 | 7 | 14 | 22 | 14 | 20 | 15 | 27 | 19 |
| 55 | 3 | 9 | 4 | 0 | 6 | 1 | 1 | 8 | 4 | 2 | 5 | 1 |
| 56 | 7 | 6 | 2 | 10 | 2 | 7 | 3 | 3 | 3 | 9 | 4 | 3 |
| 57 | 8 | 12 | 8 | 5 | 6 | 8 | 7 | 8 | 5 | 8 | 8 | 10 |
| 58 | 0 | 2 | 2 | 3 | 3 | 2 | 3 | 1 | 3 | 5 | 4 | 5 |
| 59 | 1 | 2 | 7 | 3 | 0 | 4 | 2 | 1 | 4 | 2 | 0 | 1 |
| 60 | 5 | 5 | 9 | 1 | 2 | 2 | 2 | 1 | 4 | 4 | 2 | 5 |
| 61 | 3 | 4 | 3 | 3 | 1 | 1 | 4 | 4 | 5 | 4 | 2 | 2 |
| 62 |  |  |  |  |  |  |  |  |  |  |  |  |
| 63 | 3 | 3 | 5 | 1 | 3 | 1 | 3 | 1 | 3 | 5 | 3 | 3 |
| 64 | 1 | 2 | 3 | 2 | 5 | 5 | 8 | 2 | 4 | 10 | 3 | 2 |
| 65 | 7 | 4 | 8 | 4 | 1 | 4 | 6 | 7 | 4 | 4 | 11 | 8 |
| 66 | 0 | 0 | 1 | 0 | 0 | 0 | 0 | 0 | 1 | 4 | 2 | 1 |
| 67 | 2 | 4 | 1 | 5 | 3 | 1 | 1 | 1 | 3 | 4 | 2 | 1 |
| 68 | 0 | 3 | 7 | 2 | 3 | 4 | 6 | 7 | 5 | 4 | 4 | 6 |
| 69 | 1 | 8 | 5 | 7 | 6 | 8 | 6 | 11 | 12 | 5 | 12 | 17 |
| 70 | 1 |  | 1 | 1 | 1 |  |  | 1 |  | 1 |  |  |
| 71 | 6 | 2 | 2 | 2 | 1 | 0 | 3 | 6 | 3 | 1 | 2 | 4 |
| 72 | 8 | 7 | 6 | 4 | 3 | 5 | 3 | 5 | 9 | 5 | 5 | 4 |
| 73 | 1 | 2 | 3 | 4 | 2 | 3 | 7 | 1 |  | 3 | 4 | 4 |
| 74 | 4 | 7 | 5 | 6 | 11 | 6 | 10 | 7 | 3 | 9 | 3 | 6 |
| 75 |  | 1 |  |  |  | 1 |  |  |  |  |  |  |
| 76 | 1 | 6 | 3 | 3 | 2 | 4 | 3 | 4 | 2 | 5 | 2 | 4 |
| 77 | 1 | 1 | 1 | 1 | 1 |  | 1 | 1 | 1 |  | 2 | 1 |
| 78 | 1 | 1 | 1 | 1 | 1 | 1 | 2 | 1 |  |  |  | 1 |
| 79 | 13 | 9 | 9 | 5 | 0 | 3 | 6 | 11 | 9 | 4 | 4 | 11 |
| 80 | 2 | 3 | 3 | 3 | 9 | 4 | 5 | 2 | 9 | 7 | 2 | 3 |
| 81 | 2 | 5 | 3 | 3 | 1 | 3 | 2 | 2 | 5 | 2 | 1 | 1 |
| 82 |  |  |  |  |  |  |  |  |  |  |  |  |
| 83 | 12 | 5 | 11 | 4 | 2 | 4 | 6 | 4 | 5 | 5 | 5 | 10 |
| 84 | 6 | 4 | 2 | 5 | 2 | 7 | 4 | 11 | 4 | 2 | 5 | 5 |
| 85 | 1 | 1 | 1 | 0 | 2 | 1 | 0 | 0 | 1 | 0 | 3 | 2 |
| 86 | 8 | 5 | 5 | 3 | 9 | 8 | 11 | 6 | 3 | 2 | 4 | 3 |
| 87 | 2 | 1 |  |  | 1 | 4 | 1 | 3 |  | 2 | 1 | 4 |
| 88 | 6 | 2 | 3 | 2 | 1 | 4 | 10 | 3 | 3 | 3 | 3 | 6 |
| 89 | 9 | 5 | 11 | 11 | 4 | 8 | 11 | 9 | 4 | 13 | 9 | 13 |
| 90 | 4 | 4 | 6 | 3 | 6 | 3 | 4 | 1 | 1 | 1 | 7 | 3 |
| 91 | 3 | 3 | 2 | 3 | 2 | 5 | 0 | 4 | 0 | 5 | 7 | 0 |
| 92 |  |  |  |  |  |  |  |  |  |  |  |  |
| 93 | 1 | 0 | 2 | 1 | 1 | 1 | 5 | 3 | 4 | 1 | 3 | 1 |
| 94 | 1 | 4 | 2 | 2 | 0 | 2 | 2 | 1 | 5 | 1 | 0 | 3 |
| 95 | 5 | 3 | 2 | 0 | 1 | 3 | 0 | 1 | 1 | 1 | 1 | 1 |
| 96 | 3 | 1 | 7 | 5 | 4 | 2 | 2 | 6 | 3 | 8 | 4 | 5 |
| 97 | 3 | 2 | 3 | 1 | 1 | 1 | 0 | 0 | 0 | 1 | 0 | 3 |
| 98 | 2 | 2 | 1 | 1 | 1 | 0 | 2 | 0 | 0 | 0 | 0 | 2 |
| 99 | 2 | 4 | 2 | 1 | 7 | 1 | 0 | 0 | 2 | 2 | 1 | 3 |
| 100 | 0 | 1 | 1 | 5 | 2 | 1 | 5 | 1 | 3 | 6 | 3 | 0 |
| 101 |  | 2 | 3 | 5 | 2 |  | 1 | 2 | 7 | 1 | 3 | 3 |
| 102 |  |  |  |  |  |  |  |  |  |  |  |  |
| 103 | 7 | 6 | 5 | 1 | 2 | 1 | 2 | 3 | 4 | 5 | 5 | 5 |
| 104 | 1 | 2 | 2 | 6 | 4 | 4 | 3 | 2 | 9 | 7 | 6 | 4 |
| 105 | 2 | 3 | 2 | 5 | 3 | 4 | 2 | 3 | 2 | 2 | 5 | 4 |

Table 19. Number of newly diagnosed stage IV colorectal cancer during each month in 2019.

|  | Month | | | | | | | | | | | |
| --- | --- | --- | --- | --- | --- | --- | --- | --- | --- | --- | --- | --- |
| Hospital | 1 | 2 | 3 | 4 | 5 | 6 | 7 | 8 | 9 | 10 | 11 | 12 |
| 1 | 3 | 4 | 0 | 1 | 2 | 0 | 0 | 1 | 1 | 0 | 2 | 1 |
| 2 | 10 | 18 | 20 | 17 | 14 | 23 | 27 | 10 | 17 | 14 | 15 | 11 |
| 3 | 3 | 1 | 2 | 2 | 3 | 2 | 4 | 3 | 2 | 2 | 0 | 0 |
| 4 | 3 | 2 | 4 | 1 | 6 | 5 | 9 | 4 | 3 | 6 | 2 | 3 |
| 5 | 3 | 1 | 3 | 0 | 3 | 2 | 2 | 2 | 2 | 0 | 1 | 2 |
| 6 | 2 | 0 | 2 | 7 | 5 | 2 | 5 | 4 | 6 | 5 | 3 | 4 |
| 7 | 0 | 4 | 1 | 3 | 2 | 3 | 4 | 4 | 0 | 3 | 2 | 5 |
| 8 |  |  |  |  |  |  |  |  |  |  |  |  |
| 9 | 2 | 8 | 4 | 7 | 1 | 3 | 5 | 3 | 6 | 4 | 2 | 4 |
| 10 | 2 | 2 | 1 | 2 | 5 | 2 | 8 | 2 | 2 | 3 | 2 | 4 |
| 11 | 2 | 5 | 2 | 4 | 2 | 4 | 3 | 8 | 9 | 3 | 3 | 5 |
| 12 | 2 | 1 | 0 | 1 | 2 | 0 | 4 | 1 | 1 | 2 | 2 | 2 |
| 13 | 3 | 0 | 2 | 3 | 5 | 4 | 3 | 3 | 5 | 1 | 2 | 2 |
| 14 | 6 | 4 | 1 | 3 | 3 | 6 | 4 | 7 | 1 | 6 | 4 | 4 |
| 15 | 3 | 2 | 5 | 5 | 3 | 3 | 2 | 7 | 0 | 5 | 3 | 4 |
| 16 | 5 | 7 | 5 | 6 | 6 | 4 | 6 | 4 | 8 | 4 | 5 | 6 |
| 17 |  |  |  |  |  |  |  |  |  |  |  |  |
| 18 | 2 | 1 | 2 | 5 | 2 | 3 | 2 | 4 | 2 | 2 | 2 | 1 |
| 19 | 2 | 0 | 1 | 2 | 2 | 2 | 4 | 1 | 0 | 2 | 3 | 2 |
| 20 | 12 | 4 | 10 | 9 | 12 | 12 | 4 | 12 | 6 | 14 | 10 | 10 |
| 21 | 1 | 2 | 4 | 2 | 5 | 4 | 6 | 3 | 4 | 3 | 0 | 3 |
| 22 | 2 |  | 2 | 4 | 2 | 5 | 1 | 3 | 3 | 1 | 4 | 1 |
| 23 |  |  |  |  |  |  |  |  |  |  |  |  |
| 24 | 0 | 6 | 4 | 3 | 2 | 5 | 10 | 3 | 2 | 7 | 3 | 1 |
| 25 | 4 | 8 | 9 | 4 | 5 | 5 | 8 | 5 | 3 | 4 | 1 | 4 |
| 26 | 1 | 3 | 2 | 4 | 4 | 2 | 2 | 3 | 1 | 5 | 3 | 3 |
| 27 | 3 | 1 | 4 | 1 | 1 | 3 | 4 | 1 | 5 | 1 | 3 | 3 |
| 28 | 1 | 0 | 2 | 2 | 2 | 1 | 2 | 2 | 3 | 4 | 2 | 1 |
| 29 |  |  |  |  |  |  |  |  |  |  |  |  |
| 30 | 2 | 1 | 1 | 3 | 2 | 1 | 1 | 2 | 4 | 6 | 1 | 2 |
| 31 | 2 | 1 | 0 | 2 | 4 | 2 | 4 | 3 | 1 | 1 | 2 | 2 |
| 32 | 2 | 3 | 5 | 4 | 4 | 2 | 6 | 1 | 3 | 4 | 3 | 5 |
| 33 | 0 | 3 | 3 | 4 | 2 | 2 | 4 | 2 | 3 | 8 | 6 | 4 |
| 34 | 0 | 2 | 1 | 0 | 5 | 1 | 3 | 3 | 0 | 2 | 3 | 0 |
| 35 | 5 | 3 | 4 | 2 | 3 | 1 | 3 | 6 | 2 | 2 | 1 | 8 |
| 36 | 3 | 1 | 2 | 2 | 1 | 2 | 3 | 5 | 2 | 2 | 6 | 1 |
| 37 | 4 | 4 | 0 | 8 | 2 | 4 | 7 | 7 | 5 | 2 | 5 | 7 |
| 38 |  |  |  |  |  |  |  |  |  |  |  |  |
| 39 | 1 |  |  |  |  | 1 | 2 | 1 | 1 | 1 | 1 |  |
| 40 | 2 | 3 | 3 | 3 | 2 | 1 | 4 | 5 | 5 | 1 | 1 | 4 |
| 41 | 2 | 5 | 3 | 6 | 6 | 1 | 6 | 4 | 7 | 1 | 5 | 2 |
| 42 | 3 | 1 | 2 | 2 | 2 | 2 | 2 | 0 | 0 | 0 | 2 | 6 |
| 43 | 5 | 4 | 2 | 8 | 2 | 5 | 5 | 10 | 1 | 2 | 2 | 2 |
| 44 | 1 | 2 | 2 | 2 | 0 | 0 | 3 | 2 | 0 | 1 | 0 | 1 |
| 45 | 2 | 5 | 4 | 5 | 8 | 7 | 4 | 3 | 5 | 5 | 3 | 2 |
| 46 | 6 | 3 | 2 | 7 | 6 | 5 | 5 | 3 | 3 |  | 5 | 6 |
| 47 |  |  | 1 |  | 1 | 2 | 1 | 2 | 2 |  | 1 | 2 |
| 48 | 4 | 7 | 5 | 4 | 5 | 4 | 2 | 3 | 3 | 7 | 3 | 8 |
| 49 | 0 | 2 | 2 | 3 | 1 | 4 | 4 | 2 | 2 | 1 | 4 | 2 |
| 50 | 0 | 1 | 2 | 1 | 0 | 0 | 0 | 3 | 0 | 2 | 0 | 0 |
| 51 | 2 | 5 | 4 | 3 | 0 | 5 | 7 | 6 | 2 | 5 | 8 | 3 |
| 52 | 4 | 3 | 4 | 3 | 3 | 4 | 3 | 2 | 4 | 1 | 2 | 5 |
| 53 | 8 | 5 | 1 | 6 | 5 | 5 | 4 | 5 | 5 | 8 | 2 | 8 |
| 54 | 6 | 17 | 8 | 17 | 13 | 10 | 9 | 8 | 16 | 9 | 10 | 14 |
| 55 | 7 | 3 | 3 | 2 | 3 | 5 | 3 | 5 | 1 | 2 | 3 | 2 |
| 56 | 4 | 4 | 5 | 4 | 4 | 1 | 8 | 5 | 3 | 2 | 0 | 3 |
| 57 | 8 | 7 | 13 | 9 | 10 | 10 | 10 | 8 | 2 | 6 | 7 | 3 |
| 58 | 2 | 3 | 2 | 1 | 3 | 1 | 3 | 1 | 2 | 1 | 3 | 1 |
| 59 | 2 | 4 | 3 | 4 | 1 | 7 | 1 | 3 | 0 | 0 | 1 | 1 |
| 60 | 4 | 2 | 1 | 4 | 7 | 8 | 3 | 5 | 6 | 6 | 7 | 3 |
| 61 | 1 | 1 | 4 | 5 | 2 | 4 | 3 | 1 | 3 | 1 | 3 | 1 |
| 62 |  |  |  |  |  |  |  |  |  |  |  |  |
| 63 | 2 | 2 | 1 | 2 | 4 |  | 1 | 2 | 2 | 3 | 1 | 2 |
| 64 | 5 | 2 | 1 | 1 | 2 | 5 | 2 | 1 |  | 4 | 1 | 3 |
| 65 | 4 | 2 | 7 | 6 | 2 | 5 | 3 | 5 | 7 | 5 | 5 | 4 |
| 66 | 0 | 1 | 0 | 0 | 1 | 1 | 0 | 2 | 2 | 2 | 0 | 2 |
| 67 | 2 | 2 | 4 | 8 | 1 | 0 | 2 | 1 | 5 | 1 | 5 | 3 |
| 68 | 4 | 3 | 3 | 5 | 3 | 4 | 6 | 3 | 4 | 3 | 4 | 3 |
| 69 | 6 | 9 | 12 | 10 | 7 | 8 | 6 | 7 | 14 | 11 | 8 | 13 |
| 70 | 1 | 3 |  | 1 | 1 | 2 | 2 | 1 | 2 | 1 |  | 1 |
| 71 | 1 | 5 | 10 | 4 | 3 | 4 | 6 | 4 | 3 | 2 | 0 | 1 |
| 72 | 7 | 7 | 1 | 4 | 4 | 4 | 7 | 3 | 2 | 5 | 6 | 3 |
| 73 | 2 | 5 | 4 | 3 | 3 | 5 | 2 | 3 |  | 6 | 3 | 4 |
| 74 | 1 | 4 | 3 | 7 | 5 | 2 | 4 | 3 | 2 | 2 | 5 | 3 |
| 75 |  | 1 | 1 |  |  |  |  |  |  |  |  |  |
| 76 | 0 | 0 | 4 | 2 | 3 | 4 | 3 | 3 | 4 | 4 | 4 | 1 |
| 77 |  |  |  | 1 |  |  |  |  | 2 |  | 2 |  |
| 78 | 1 | 1 | 1 | 1 | 1 |  |  |  | 1 | 2 |  | 1 |
| 79 | 2 | 0 | 2 | 3 | 6 | 3 | 8 | 5 | 4 | 2 | 5 | 2 |
| 80 | 5 | 4 | 6 | 2 | 3 | 3 | 5 | 4 | 4 | 4 | 3 | 3 |
| 81 | 3 | 1 | 2 | 2 |  | 3 | 4 | 3 | 3 | 3 | 3 | 3 |
| 82 |  |  |  |  |  |  |  |  |  |  |  |  |
| 83 | 1 | 4 | 2 | 2 | 3 | 3 | 5 | 3 | 6 | 2 | 1 | 4 |
| 84 | 3 | 2 | 9 | 6 | 3 | 5 | 2 | 3 | 1 | 0 | 1 | 1 |
| 85 | 2 | 9 | 1 | 1 | 3 | 3 | 2 | 0 | 1 | 4 | 3 | 1 |
| 86 | 8 | 3 | 1 | 3 | 1 | 1 | 3 | 2 | 8 | 3 | 3 | 1 |
| 87 | 2 | 3 | 1 |  | 5 |  |  |  | 4 | 3 | 4 | 1 |
| 88 | 1 | 2 | 3 | 0 | 1 | 5 | 3 | 4 | 3 | 1 | 3 | 2 |
| 89 | 8 | 15 | 12 | 9 | 9 | 12 | 8 | 14 | 10 | 7 | 8 | 9 |
| 90 | 3 | 1 | 6 | 3 | 5 | 0 | 9 | 4 | 5 | 3 | 1 | 3 |
| 91 | 2 | 3 | 4 | 3 | 5 | 1 | 4 | 2 | 7 | 4 | 3 | 5 |
| 92 |  |  |  |  |  |  |  |  |  |  |  |  |
| 93 | 3 | 1 | 4 | 6 | 3 | 1 | 3 | 1 | 3 | 3 | 1 | 6 |
| 94 | 0 | 1 | 4 | 1 | 3 | 4 | 1 | 3 | 1 | 5 | 4 | 0 |
| 95 | 2 | 3 | 1 | 2 | 2 | 0 | 0 | 2 | 0 | 1 | 2 | 0 |
| 96 | 3 | 3 | 1 | 4 | 2 | 2 | 2 | 2 | 3 | 5 | 2 | 3 |
| 97 | 2 | 0 | 1 | 1 | 0 | 0 | 0 | 0 | 1 | 1 | 0 | 0 |
| 98 | 1 | 2 | 3 | 1 | 3 | 1 | 4 | 0 | 1 | 2 | 3 | 2 |
| 99 | 1 | 2 | 3 | 3 | 2 | 2 | 2 | 1 | 3 | 0 | 0 | 4 |
| 100 | 1 | 1 | 3 | 1 | 2 | 2 | 1 | 2 | 2 | 1 | 3 | 0 |
| 101 | 4 | 2 | 8 | 2 | 4 | 4 | 4 | 7 | 3 | 5 | 7 | 5 |
| 102 |  |  |  |  |  |  |  |  |  |  |  |  |
| 103 | 4 | 1 | 5 | 6 | 2 | 0 | 4 | 5 | 0 | 4 | 3 | 7 |
| 104 | 3 | 2 | 8 | 7 | 8 | 4 | 5 | 4 | 3 | 5 | 4 | 4 |
| 105 | 3 | 3 | 3 | 1 | 2 | 4 | 5 | 2 | 4 | 3 | 8 | 2 |

Table 20. Number of newly diagnosed stage IV colorectal cancer during each month in 2020.

|  | Month | | | | | | | | | | | |
| --- | --- | --- | --- | --- | --- | --- | --- | --- | --- | --- | --- | --- |
| Hospital | 1 | 2 | 3 | 4 | 5 | 6 | 7 | 8 | 9 | 10 | 11 | 12 |
| 1 | 0 | 2 | 3 | 0 | 3 | 2 | 0 | 0 | 0 | 1 | 0 | 0 |
| 2 | 18 | 11 | 14 | 10 | 7 | 14 | 13 | 17 | 11 | 20 | 11 | 18 |
| 3 | 2 | 2 | 4 | 1 | 2 | 2 | 3 | 3 | 2 | 1 | 1 | 2 |
| 4 | 4 | 4 | 4 | 4 | 5 | 5 | 5 | 4 | 3 | 6 | 4 | 10 |
| 5 | 2 | 2 | 0 | 3 | 1 | 2 | 1 | 2 | 1 | 3 | 1 | 1 |
| 6 | 1 | 3 | 6 | 3 | 1 | 3 | 5 | 2 | 1 | 6 | 3 | 1 |
| 7 | 3 | 4 | 2 | 1 | 2 | 4 | 1 | 1 | 2 | 1 | 3 | 1 |
| 8 |  |  |  |  |  |  |  |  |  |  |  |  |
| 9 | 0 | 3 | 4 | 3 | 1 | 1 | 3 | 2 | 4 | 4 | 2 | 4 |
| 10 | 1 | 4 | 1 | 4 |  | 3 | 5 | 3 | 5 | 2 | 3 | 3 |
| 11 | 4 | 2 | 6 | 2 | 4 | 2 | 6 | 5 | 6 | 5 | 8 | 3 |
| 12 | 0 | 2 | 0 | 0 | 1 | 3 | 1 | 1 | 2 | 0 | 0 | 1 |
| 13 | 3 | 2 | 1 | 1 | 4 | 6 | 8 | 4 | 3 | 3 | 4 | 2 |
| 14 | 5 | 1 | 2 | 3 | 1 | 7 | 8 | 4 | 4 | 2 | 0 | 6 |
| 15 | 3 | 1 | 3 | 3 | 3 | 3 | 4 | 0 | 3 | 4 | 4 | 5 |
| 16 | 5 | 6 | 8 | 10 | 6 | 8 | 6 | 2 | 5 | 5 | 7 | 4 |
| 17 |  |  |  |  |  |  |  |  |  |  |  |  |
| 18 | 1 | 2 | 5 | 4 | 2 | 3 | 4 | 6 | 6 | 5 | 1 | 3 |
| 19 | 2 | 1 | 1 | 2 | 0 | 0 | 0 | 3 | 0 | 1 | 1 | 2 |
| 20 | 14 | 6 | 7 | 4 | 7 | 9 | 6 | 8 | 8 | 10 | 6 | 7 |
| 21 | 5 | 1 | 2 | 4 | 3 | 3 | 3 | 5 | 2 | 0 | 1 | 5 |
| 22 | 3 | 3 | 1 | 1 | 1 | 2 | 3 | 2 | 4 | 2 | 1 | 2 |
| 23 |  |  |  |  |  |  |  |  |  |  |  |  |
| 24 | 4 | 3 | 2 | 1 | 3 | 5 | 5 | 7 | 3 | 8 | 3 | 2 |
| 25 | 4 | 9 | 3 | 2 | 1 | 4 | 5 | 4 | 2 | 1 | 3 | 8 |
| 26 | 1 | 2 | 3 | 3 | 1 | 4 |  | 4 | 7 | 4 | 1 | 3 |
| 27 | 5 | 5 | 3 | 0 | 3 | 3 | 0 | 1 | 0 | 2 | 5 | 5 |
| 28 | 0 | 1 | 3 | 2 | 1 | 1 | 3 | 2 | 2 | 8 | 3 | 0 |
| 29 |  |  |  |  |  |  |  |  |  |  |  |  |
| 30 | 2 | 1 | 3 | 2 | 1 | 2 | 4 | 2 | 3 | 7 | 1 | 0 |
| 31 | 0 | 2 | 2 | 2 | 0 | 4 | 3 | 1 | 0 | 2 | 0 | 1 |
| 32 | 1 | 2 | 2 | 4 | 5 | 6 | 5 | 3 | 2 | 3 | 6 | 3 |
| 33 | 2 | 1 | 5 | 2 | 1 | 6 | 5 | 4 | 4 | 8 | 4 | 3 |
| 34 | 1 | 1 | 0 | 1 | 0 | 3 | 3 | 1 | 4 | 1 | 1 | 2 |
| 35 | 2 | 2 | 5 | 6 | 2 | 5 | 4 | 6 | 2 | 7 | 0 | 3 |
| 36 | 3 | 1 | 1 | 2 | 5 |  | 3 | 2 | 5 | 4 | 2 | 2 |
| 37 | 7 | 6 | 2 | 5 | 2 | 5 | 5 | 7 | 2 | 10 | 3 | 3 |
| 38 |  |  |  |  |  |  |  |  |  |  |  |  |
| 39 | 2 |  |  | 2 | 3 | 2 | 4 | 1 | 1 | 2 | 1 |  |
| 40 | 1 | 5 | 4 | 2 | 7 | 1 | 3 | 6 | 3 | 5 | 6 | 3 |
| 41 | 1 | 3 | 3 | 2 | 4 | 1 | 5 | 2 | 4 | 4 | 4 | 1 |
| 42 | 3 | 3 | 3 | 6 | 3 | 6 | 2 | 0 | 1 | 1 | 1 | 2 |
| 43 | 9 | 4 | 2 | 2 | 2 | 3 | 2 | 2 | 2 | 6 | 2 | 5 |
| 44 | 1 | 0 | 1 | 3 | 1 | 1 | 3 | 1 | 1 | 1 | 0 | 1 |
| 45 | 1 | 8 | 2 | 2 | 2 | 7 | 2 | 5 | 1 | 4 | 4 | 1 |
| 46 | 4 | 8 | 4 | 7 | 6 | 1 | 3 | 1 | 4 | 6 | 6 | 4 |
| 47 |  |  | 2 |  | 1 | 1 |  | 1 | 3 | 1 |  | 2 |
| 48 | 1 | 5 | 4 | 1 | 3 | 5 | 3 | 4 | 1 | 8 | 4 | 5 |
| 49 | 1 | 2 | 1 | 3 | 2 | 0 | 1 | 0 | 1 | 1 | 2 | 3 |
| 50 | 0 | 2 | 0 | 0 | 0 | 0 | 2 | 0 | 0 | 0 | 0 | 0 |
| 51 | 5 | 3 | 4 | 7 | 3 | 2 | 2 | 4 | 5 | 4 | 5 | 5 |
| 52 | 3 | 1 | 3 | 3 | 3 | 2 | 2 | 3 | 7 | 2 | 1 | 0 |
| 53 | 2 | 3 | 6 | 5 | 7 | 6 | 4 | 5 | 7 | 5 | 2 | 8 |
| 54 | 15 | 14 | 7 | 12 | 6 | 16 | 11 | 10 | 15 | 18 | 14 | 11 |
| 55 | 4 | 1 | 1 | 1 | 2 | 2 | 1 | 2 | 4 | 1 | 3 | 3 |
| 56 | 3 | 3 | 5 | 3 | 0 | 4 | 1 | 3 | 2 | 3 | 1 | 4 |
| 57 | 5 | 12 | 10 | 5 | 7 | 8 | 4 | 8 | 9 | 5 | 4 | 10 |
| 58 | 0 | 2 | 1 | 2 | 3 | 1 | 1 | 1 | 4 | 2 | 2 | 2 |
| 59 | 6 | 1 | 2 | 0 | 3 | 2 | 2 | 4 | 1 | 2 | 2 | 1 |
| 60 | 3 | 5 | 5 | 0 | 3 | 6 | 2 | 0 | 4 | 1 | 0 | 0 |
| 61 | 3 | 0 | 2 | 2 | 3 | 1 | 3 | 3 | 3 | 6 | 2 | 3 |
| 62 |  |  |  |  |  |  |  |  |  |  |  |  |
| 63 | 5 | 1 | 2 | 2 | 1 | 2 | 1 | 1 |  | 1 | 2 | 3 |
| 64 | 3 | 1 | 5 |  | 1 | 5 | 5 | 2 | 4 | 5 | 2 | 4 |
| 65 | 5 | 9 | 1 | 5 | 1 | 6 | 1 | 5 | 5 | 4 | 4 | 5 |
| 66 | 1 | 1 | 0 | 0 | 1 | 1 | 3 | 0 | 0 | 2 | 0 | 0 |
| 67 | 1 | 3 | 2 | 7 | 1 | 3 | 4 | 1 | 1 | 1 | 3 | 2 |
| 68 | 4 | 4 | 2 | 3 | 2 | 4 | 3 | 4 | 1 | 3 | 3 | 4 |
| 69 | 10 | 13 | 3 | 9 | 9 | 11 | 5 | 21 | 16 | 19 | 11 | 11 |
| 70 | 3 | 1 |  | 1 | 2 | 1 | 1 | 2 | 1 |  | 1 |  |
| 71 | 3 | 2 | 4 | 3 | 4 | 2 | 3 | 3 | 8 | 2 | 1 | 3 |
| 72 | 7 | 6 | 1 | 3 | 4 | 5 | 1 | 7 | 5 | 5 | 4 | 9 |
| 73 | 2 | 2 | 5 | 1 | 2 | 2 | 5 | 6 | 6 | 4 | 4 |  |
| 74 | 5 | 1 | 2 | 5 | 6 | 3 | 3 | 6 | 6 | 3 | 4 | 1 |
| 75 |  |  |  |  |  |  |  |  |  |  |  |  |
| 76 | 4 | 3 | 2 | 2 | 5 | 5 | 4 | 6 | 4 | 4 | 2 | 6 |
| 77 | 2 |  | 3 |  | 1 | 2 |  |  | 1 |  |  | 1 |
| 78 |  | 1 |  | 1 |  | 2 | 1 | 1 |  | 1 |  |  |
| 79 | 4 | 3 | 4 | 2 | 2 | 2 | 3 | 3 | 2 | 1 | 3 | 1 |
| 80 | 4 | 3 | 3 | 1 | 4 | 3 | 3 | 4 | 4 | 6 | 5 | 3 |
| 81 | 2 | 1 | 4 | 2 | 2 | 3 | 5 | 5 | 3 | 4 | 2 | 3 |
| 82 |  |  |  |  |  |  |  |  |  |  |  |  |
| 83 | 1 | 5 | 3 | 6 | 2 | 2 | 5 | 3 | 6 | 1 | 3 | 1 |
| 84 | 2 | 4 | 2 | 0 | 2 | 7 | 4 | 4 | 4 | 4 | 4 | 3 |
| 85 | 0 | 4 | 2 | 1 | 8 | 4 | 3 | 3 | 1 | 4 | 2 | 1 |
| 86 | 1 | 3 | 5 | 4 | 3 | 4 | 5 | 3 | 5 | 3 | 2 | 4 |
| 87 | 2 | 3 | 1 | 2 | 2 | 3 | 2 | 2 | 3 | 1 | 1 | 3 |
| 88 | 5 | 2 | 4 | 1 | 5 | 2 | 3 | 3 | 3 | 1 | 1 | 3 |
| 89 | 14 | 9 | 9 | 14 | 16 | 13 | 13 | 14 | 8 | 8 | 12 | 14 |
| 90 | 3 | 2 | 6 | 2 | 2 | 6 | 0 | 1 | 1 | 1 | 0 | 2 |
| 91 | 2 | 6 | 5 | 4 | 3 | 1 | 4 | 4 | 4 | 4 | 2 | 5 |
| 92 |  |  |  |  |  |  |  |  |  |  |  |  |
| 93 | 3 | 2 | 1 | 1 | 3 | 1 | 1 | 4 | 2 | 1 | 3 | 4 |
| 94 | 0 | 1 | 1 | 1 | 3 | 1 | 1 | 2 | 3 | 0 | 2 | 1 |
| 95 | 0 | 1 | 0 | 2 | 1 | 1 | 1 | 3 | 2 | 0 | 1 | 0 |
| 96 | 5 | 9 | 2 | 3 | 1 | 6 | 4 | 3 | 5 | 4 | 2 | 5 |
| 97 | 1 | 4 | 0 | 0 | 0 | 0 | 0 | 0 | 0 | 0 | 0 | 0 |
| 98 | 1 | 5 | 2 | 1 | 4 | 4 | 1 | 1 | 1 | 0 | 5 | 1 |
| 99 | 1 | 3 | 4 | 1 | 2 | 2 | 1 | 1 | 3 | 1 | 0 | 2 |
| 100 | 2 | 2 | 3 | 2 | 0 | 2 | 4 | 3 | 2 | 3 | 0 | 5 |
| 101 | 4 | 2 | 2 | 3 | 2 | 2 | 4 | 2 | 6 | 7 | 2 | 2 |
| 102 |  |  |  |  |  |  |  |  |  |  |  |  |
| 103 | 4 | 4 | 4 | 5 | 2 | 4 | 3 | 3 | 5 | 4 | 4 | 2 |
| 104 | 2 | 4 | 7 | 4 | 6 | 4 | 6 | 3 | 3 | 6 | 2 | 3 |
| 105 | 1 | 3 | 2 | 3 | 3 | 4 | 2 | 1 | 1 | 3 | 7 | 4 |

Table 21. Number of newly diagnosed lung cancer during each month in 2019.

|  | Month | | | | | | | | | | | |
| --- | --- | --- | --- | --- | --- | --- | --- | --- | --- | --- | --- | --- |
| Hospital | 1 | 2 | 3 | 4 | 5 | 6 | 7 | 8 | 9 | 10 | 11 | 12 |
| 1 | 14 | 18 | 8 | 8 | 11 | 13 | 15 | 14 | 16 | 23 | 24 | 27 |
| 2 | 43 | 47 | 41 | 43 | 34 | 44 | 46 | 36 | 41 | 57 | 39 | 41 |
| 3 | 2 | 3 | 2 | 5 | 7 | 2 | 4 | 4 | 5 | 1 | 4 | 6 |
| 4 | 24 | 36 | 34 | 29 | 21 | 27 | 24 | 30 | 31 | 32 | 35 | 25 |
| 5 | 12 | 8 | 13 | 8 | 3 | 13 | 7 | 11 | 6 | 15 | 3 | 6 |
| 6 | 15 | 19 | 26 | 13 | 15 | 19 | 19 | 15 | 11 | 22 | 16 | 19 |
| 7 | 15 | 14 | 17 | 18 | 10 | 6 | 11 | 12 | 16 | 18 | 21 | 6 |
| 8 | 7 | 6 | 8 | 7 | 5 | 7 | 6 | 9 | 14 | 6 | 4 | 9 |
| 9 | 18 | 7 | 16 | 18 | 18 | 11 | 12 | 15 | 25 | 12 | 14 | 16 |
| 10 | 15 | 7 | 13 | 7 | 5 | 14 | 23 | 10 | 11 | 7 | 9 | 9 |
| 11 | 42 | 33 | 32 | 40 | 29 | 30 | 35 | 31 | 21 | 26 | 23 | 20 |
| 12 | 3 | 1 | 1 | 2 | 3 | 3 | 5 | 5 | 5 | 1 | 2 | 4 |
| 13 | 20 | 25 | 28 | 27 | 29 | 19 | 29 | 29 | 25 | 28 | 26 | 24 |
| 14 | 22 | 22 | 20 | 13 | 16 | 18 | 18 | 11 | 20 | 12 | 18 | 15 |
| 15 | 35 | 37 | 29 | 26 | 29 | 28 | 30 | 27 | 38 | 39 | 29 | 20 |
| 16 | 32 | 28 | 29 | 36 | 30 | 28 | 35 | 29 | 33 | 31 | 25 | 18 |
| 17 | 14 | 15 | 11 | 15 | 12 | 15 | 9 | 19 | 16 | 7 | 12 | 22 |
| 18 | 13 | 16 | 18 | 25 | 24 | 20 | 14 | 13 | 21 | 20 | 27 | 12 |
| 19 | 7 | 5 | 19 | 9 | 9 | 11 | 12 | 7 | 7 | 8 | 7 | 14 |
| 20 | 86 | 88 | 86 | 81 | 79 | 98 | 90 | 85 | 88 | 103 | 101 | 96 |
| 21 | 15 | 15 | 24 | 20 | 24 | 16 | 20 | 26 | 22 | 23 | 19 | 22 |
| 22 | 18 | 18 | 19 | 16 | 19 | 24 | 19 | 13 | 19 | 23 | 20 | 11 |
| 23 |  |  |  |  |  |  |  |  |  |  |  |  |
| 24 | 35 | 24 | 18 | 41 | 28 | 20 | 39 | 25 | 34 | 28 | 30 | 37 |
| 25 | 19 | 13 | 16 | 19 | 20 | 17 | 10 | 19 | 15 | 12 | 17 | 15 |
| 26 | 21 | 15 | 23 | 21 | 16 | 18 | 18 | 32 | 19 | 29 | 33 | 27 |
| 27 | 15 | 8 | 10 | 18 | 14 | 12 | 16 | 21 | 9 | 18 | 13 | 18 |
| 28 | 13 | 14 | 16 | 13 | 14 | 17 | 20 | 24 | 16 | 24 | 24 | 10 |
| 29 | 56 | 36 | 57 | 50 | 32 | 53 | 64 | 51 | 32 | 59 | 46 | 52 |
| 30 | 14 | 11 | 22 | 13 | 11 | 16 | 22 | 11 | 17 | 12 | 27 | 6 |
| 31 | 14 | 16 | 17 | 12 | 12 | 13 | 16 | 8 | 17 | 13 | 15 | 12 |
| 32 | 15 | 12 | 18 | 5 | 6 | 11 | 11 | 12 | 8 | 10 | 10 | 11 |
| 33 | 10 | 16 | 13 | 15 | 11 | 16 | 19 | 17 | 20 | 11 | 14 | 11 |
| 34 |  |  |  |  |  |  |  |  |  |  |  |  |
| 35 | 19 | 19 | 15 | 18 | 24 | 25 | 17 | 17 | 11 | 18 | 17 | 16 |
| 36 | 12 | 10 | 10 | 11 | 14 | 7 | 10 | 14 | 13 | 10 | 12 | 9 |
| 37 | 41 | 28 | 43 | 33 | 31 | 30 | 43 | 38 | 46 | 45 | 43 | 38 |
| 38 | 48 | 34 | 34 | 38 | 36 | 41 | 36 | 37 | 36 | 39 | 30 | 46 |
| 39 |  |  |  |  |  |  |  |  |  |  |  |  |
| 40 | 24 | 22 | 22 | 24 | 26 | 16 | 33 | 24 | 23 | 21 | 21 | 30 |
| 41 | 10 | 12 | 8 | 13 | 6 | 8 | 12 | 13 | 4 | 13 | 13 | 8 |
| 42 | 16 | 12 | 16 | 16 | 13 | 24 | 16 | 21 | 22 | 15 | 14 | 21 |
| 43 | 14 | 14 | 19 | 13 | 28 | 21 | 25 | 13 | 17 | 20 | 28 | 11 |
| 44 | 5 | 11 | 10 | 11 | 13 | 4 | 12 | 11 | 7 | 8 | 9 | 8 |
| 45 | 15 | 14 | 20 | 18 | 11 | 12 | 17 | 12 | 20 | 12 | 15 | 20 |
| 46 | 13 | 17 | 16 | 19 | 14 | 15 | 16 | 12 | 17 | 23 | 15 | 19 |
| 47 |  |  |  |  |  |  |  |  |  |  |  |  |
| 48 | 13 | 37 | 23 | 23 | 26 | 25 | 38 | 31 | 24 | 24 | 27 | 31 |
| 49 | 16 | 7 | 8 | 10 | 8 | 19 | 12 | 11 | 7 | 8 | 16 | 16 |
| 50 | 0 | 3 | 1 | 1 | 2 | 1 | 3 | 1 | 1 | 1 | 0 | 3 |
| 51 | 15 | 19 | 23 | 13 | 16 | 26 | 22 | 23 | 25 | 21 | 21 | 20 |
| 52 | 21 | 23 | 20 | 20 | 12 | 27 | 26 | 17 | 27 | 19 | 22 | 20 |
| 53 | 21 | 22 | 24 | 23 | 40 | 37 | 36 | 22 | 25 | 31 | 30 | 39 |
| 54 | 71 | 61 | 74 | 61 | 82 | 72 | 90 | 82 | 60 | 101 | 86 | 71 |
| 55 | 17 | 19 | 14 | 7 | 16 | 8 | 14 | 14 | 16 | 18 | 15 | 20 |
| 56 | 42 | 51 | 54 | 52 | 48 | 44 | 46 | 52 | 46 | 64 | 47 | 51 |
| 57 | 39 | 37 | 43 | 40 | 41 | 28 | 39 | 41 | 40 | 61 | 39 | 54 |
| 58 | 15 | 9 | 18 | 25 | 29 | 23 | 23 | 14 | 19 | 25 | 26 | 12 |
| 59 | 22 | 16 | 15 | 22 | 12 | 14 | 23 | 25 | 23 | 20 | 14 | 18 |
| 60 | 22 | 23 | 21 | 11 | 21 | 28 | 20 | 21 | 26 | 20 | 27 | 24 |
| 61 | 18 | 11 | 22 | 22 | 16 | 22 | 17 | 16 | 22 | 22 | 33 | 19 |
| 62 | 38 | 39 | 27 | 29 | 30 | 43 | 41 | 33 | 33 | 40 | 36 | 31 |
| 63 |  |  |  |  |  |  |  |  |  |  |  |  |
| 64 |  |  |  |  |  |  |  |  |  |  |  |  |
| 65 | 19 | 21 | 19 | 18 | 20 | 16 | 20 | 20 | 16 | 37 | 26 | 14 |
| 66 | 5 | 2 | 0 | 2 | 7 | 1 | 11 | 5 | 6 | 6 | 3 | 6 |
| 67 | 22 | 25 | 20 | 29 | 16 | 25 | 26 | 20 | 21 | 28 | 27 | 19 |
| 68 | 13 | 13 | 6 | 9 | 13 | 10 | 13 | 9 | 20 | 14 | 7 | 7 |
| 68 | 27 | 35 | 31 | 28 | 32 | 27 | 23 | 23 | 24 | 21 | 31 | 38 |
| 70 | 1 | 4 | 2 | 4 | 4 | 2 | 7 | 0 | 4 | 1 | 3 | 1 |
| 71 | 10 | 6 | 12 | 16 | 12 | 12 | 9 | 14 | 10 | 13 | 11 | 12 |
| 72 | 35 | 23 | 27 | 26 | 21 | 30 | 31 | 36 | 22 | 19 | 24 | 38 |
| 73 | 18 | 14 | 28 | 13 | 19 | 17 | 16 | 16 | 22 | 15 | 31 | 12 |
| 74 | 22 | 36 | 35 | 37 | 29 | 28 | 37 | 39 | 44 | 31 | 25 | 44 |
| 75 |  |  |  |  |  |  |  |  |  |  |  |  |
| 76 | 20 | 16 | 14 | 27 | 18 | 28 | 19 | 16 | 25 | 20 | 25 | 19 |
| 77 | 2 | 1 | 1 | 4 | 3 | 3 | 3 | 4 | 1 | 2 | 4 | 3 |
| 78 |  |  |  |  |  |  |  |  |  |  |  |  |
| 79 | 28 | 20 | 23 | 24 | 38 | 20 | 32 | 23 | 23 | 25 | 28 | 22 |
| 80 | 16 | 10 | 18 | 18 | 29 | 14 | 33 | 23 | 28 | 19 | 29 | 21 |
| 81 | 10 | 7 | 22 | 18 | 13 | 18 | 20 | 14 | 20 | 25 | 13 | 19 |
| 82 | 11 | 13 | 18 | 16 | 7 | 14 | 9 | 10 | 10 | 11 | 14 | 10 |
| 83 |  |  |  |  |  |  |  |  |  |  |  |  |
| 84 | 10 | 16 | 22 | 24 | 12 | 19 | 17 | 18 | 20 | 14 | 12 | 16 |
| 85 | 22 | 24 | 26 | 24 | 24 | 25 | 26 | 25 | 28 | 24 | 20 | 18 |
| 86 | 19 | 18 | 13 | 15 | 15 | 22 | 27 | 17 | 23 | 27 | 24 | 17 |
| 87 | 11 | 10 | 12 | 14 | 12 | 7 | 14 | 13 | 9 | 10 | 12 | 10 |
| 88 | 11 | 6 | 13 | 22 | 10 | 8 | 14 | 17 | 18 | 16 | 15 | 14 |
| 89 | 79 | 63 | 62 | 80 | 60 | 60 | 85 | 77 | 53 | 83 | 71 | 76 |
| 90 | 20 | 21 | 19 | 13 | 20 | 19 | 24 | 15 | 17 | 23 | 24 | 17 |
| 91 | 20 | 30 | 33 | 25 | 25 | 29 | 32 | 24 | 18 | 20 | 26 | 30 |
| 92 |  |  |  |  |  |  |  |  |  |  |  |  |
| 93 |  |  |  |  |  |  |  |  |  |  |  |  |
| 94 | 4 | 5 | 11 | 10 | 6 | 3 | 3 | 3 | 6 | 6 | 12 | 5 |
| 95 | 4 | 3 | 4 | 5 | 8 | 6 | 8 | 4 | 4 | 7 | 6 | 1 |
| 96 | 10 | 13 | 10 | 11 | 10 | 13 | 10 | 12 | 8 | 8 | 12 | 6 |
| 97 |  |  |  |  |  |  |  |  |  |  |  |  |
| 98 | 9 | 11 | 14 | 13 | 13 | 22 | 14 | 7 | 10 | 10 | 19 | 9 |
| 99 | 10 | 11 | 5 | 5 | 10 | 7 | 4 | 7 | 6 | 5 | 9 | 18 |
| 100 | 19 | 32 | 24 | 20 | 21 | 14 | 19 | 16 | 17 | 18 | 21 | 14 |
| 101 | 27 | 22 | 27 | 30 | 15 | 20 | 20 | 18 | 19 | 20 | 25 | 26 |
| 102 |  |  |  |  |  |  |  |  |  |  |  |  |
| 103 | 12 | 15 | 12 | 20 | 22 | 16 | 18 | 20 | 17 | 21 | 22 | 17 |
| 104 | 18 | 22 | 20 | 20 | 26 | 17 | 31 | 16 | 22 | 25 | 21 | 24 |
| 105 | 13 | 7 | 10 | 14 | 13 | 14 | 14 | 18 | 15 | 13 | 17 | 18 |

Table 22. Number of newly diagnosed lung cancer during each month in 2020.

|  | Month | | | | | | | | | | | |
| --- | --- | --- | --- | --- | --- | --- | --- | --- | --- | --- | --- | --- |
| Hospital | 1 | 2 | 3 | 4 | 5 | 6 | 7 | 8 | 9 | 10 | 11 | 12 |
| 1 | 14 | 14 | 13 | 13 | 17 | 16 | 12 | 8 | 16 | 15 | 9 | 7 |
| 2 | 40 | 32 | 38 | 39 | 24 | 32 | 39 | 23 | 37 | 37 | 43 | 45 |
| 3 | 4 | 6 | 3 | 6 | 6 | 6 | 1 | 2 | 4 | 8 | 3 | 7 |
| 4 | 26 | 25 | 23 | 26 | 23 | 30 | 35 | 22 | 31 | 25 | 33 | 22 |
| 5 | 10 | 6 | 11 | 9 | 6 | 9 | 10 | 8 | 7 | 10 | 12 | 5 |
| 6 | 27 | 14 | 15 | 15 | 12 | 20 | 15 | 11 | 20 | 23 | 15 | 22 |
| 7 | 12 | 13 | 7 | 10 | 8 | 15 | 15 | 9 | 11 | 14 | 11 | 10 |
| 8 | 9 | 9 | 9 | 5 | 5 | 9 | 11 | 8 | 7 | 8 | 9 | 8 |
| 9 | 23 | 15 | 14 | 17 | 13 | 11 | 12 | 11 | 17 | 14 | 15 | 10 |
| 10 | 9 | 10 | 9 | 12 | 9 | 7 | 16 | 11 | 11 | 19 | 7 | 1 |
| 11 | 32 | 36 | 26 | 25 | 20 | 26 | 34 | 24 | 21 | 27 | 29 | 29 |
| 12 | 3 | 6 | 3 | 3 | 2 | 1 | 6 | 5 | 0 | 6 | 5 | 3 |
| 13 | 24 | 36 | 34 | 19 | 15 | 34 | 29 | 14 | 24 | 34 | 15 | 26 |
| 14 | 13 | 13 | 13 | 11 | 6 | 22 | 18 | 23 | 15 | 21 | 15 | 20 |
| 15 | 26 | 27 | 18 | 38 | 20 | 29 | 34 | 25 | 27 | 26 | 34 | 31 |
| 16 | 24 | 25 | 21 | 27 | 15 | 22 | 31 | 14 | 31 | 26 | 25 | 25 |
| 17 | 17 | 11 | 7 | 9 | 4 | 8 | 8 | 4 | 8 | 15 | 8 | 16 |
| 18 | 13 | 13 | 16 | 13 | 17 | 18 | 16 | 19 | 19 | 27 | 18 | 18 |
| 19 | 7 | 4 | 11 | 9 | 10 | 11 | 10 | 9 | 11 | 7 | 14 | 10 |
| 20 | 86 | 94 | 80 | 44 | 54 | 88 | 99 | 74 | 107 | 104 | 82 | 99 |
| 21 | 26 | 15 | 16 | 10 | 11 | 23 | 9 | 15 | 15 | 23 | 7 | 6 |
| 22 | 21 | 11 | 26 | 11 | 20 | 16 | 18 | 10 | 8 | 19 | 12 | 16 |
| 23 |  |  |  |  |  |  |  |  |  |  |  |  |
| 24 | 27 | 31 | 30 | 23 | 23 | 22 | 22 | 24 | 20 | 33 | 18 | 14 |
| 25 | 12 | 10 | 17 | 7 | 12 | 18 | 14 | 10 | 20 | 24 | 20 | 13 |
| 26 | 18 | 17 | 24 | 28 | 11 | 30 | 21 | 18 | 23 | 26 | 30 | 22 |
| 27 | 15 | 10 | 11 | 10 | 11 | 16 | 13 | 10 | 14 | 20 | 14 | 17 |
| 28 | 7 | 17 | 12 | 17 | 16 | 26 | 24 | 21 | 20 | 21 | 19 | 11 |
| 29 | 54 | 52 | 37 | 38 | 26 | 40 | 37 | 31 | 30 | 49 | 40 | 47 |
| 30 | 17 | 10 | 19 | 15 | 10 | 14 | 14 | 11 | 17 | 20 | 15 | 25 |
| 31 | 8 | 15 | 14 | 17 | 11 | 18 | 10 | 15 | 9 | 10 | 21 | 9 |
| 32 | 4 | 11 | 5 | 12 | 8 | 7 | 8 | 7 | 9 | 9 | 7 | 16 |
| 33 | 9 | 14 | 19 | 8 | 11 | 11 | 16 | 13 | 20 | 19 | 8 | 8 |
| 34 |  |  |  |  |  |  |  |  |  |  |  |  |
| 35 | 17 | 13 | 27 | 16 | 16 | 23 | 30 | 20 | 25 | 17 | 17 | 17 |
| 36 | 11 | 11 | 13 | 17 | 13 | 14 | 12 | 10 | 19 | 15 | 13 | 14 |
| 37 | 30 | 33 | 45 | 41 | 27 | 41 | 39 | 36 | 44 | 46 | 37 | 45 |
| 38 | 34 | 33 | 23 | 32 | 27 | 33 | 26 | 26 | 29 | 37 | 29 | 34 |
| 39 |  |  |  |  |  |  |  |  |  |  |  |  |
| 40 | 24 | 23 | 18 | 19 | 10 | 21 | 16 | 14 | 16 | 31 | 23 | 18 |
| 41 | 6 | 6 | 7 | 8 | 8 | 6 | 15 | 9 | 13 | 12 | 10 | 13 |
| 42 | 9 | 13 | 18 | 14 | 7 | 15 | 17 | 17 | 20 | 13 | 15 | 24 |
| 43 | 15 | 17 | 14 | 22 | 14 | 20 | 15 | 17 | 15 | 14 | 15 | 21 |
| 44 | 7 | 8 | 7 | 2 | 8 | 12 | 8 | 7 | 9 | 11 | 6 | 14 |
| 45 | 12 | 12 | 15 | 16 | 18 | 10 | 16 | 10 | 11 | 17 | 10 | 4 |
| 46 | 18 | 15 | 12 | 9 | 12 | 16 | 13 | 14 | 11 | 15 | 15 | 16 |
| 47 |  |  |  |  |  |  |  |  |  |  |  |  |
| 48 | 24 | 22 | 18 | 19 | 12 | 19 | 25 | 14 | 15 | 16 | 24 | 20 |
| 49 | 10 | 12 | 14 | 19 | 9 | 19 | 8 | 6 | 14 | 13 | 9 | 14 |
| 50 | 3 | 2 | 2 | 0 | 1 | 2 | 0 | 1 | 1 | 1 | 3 | 2 |
| 51 | 16 | 10 | 19 | 25 | 18 | 14 | 15 | 18 | 21 | 24 | 23 | 18 |
| 52 | 20 | 21 | 13 | 24 | 12 | 19 | 18 | 18 | 20 | 31 | 21 | 16 |
| 53 | 21 | 26 | 21 | 35 | 32 | 28 | 41 | 27 | 22 | 42 | 31 | 34 |
| 54 | 75 | 73 | 67 | 59 | 58 | 82 | 64 | 57 | 66 | 94 | 66 | 77 |
| 55 | 5 | 19 | 18 | 12 | 11 | 14 | 17 | 12 | 9 | 15 | 20 | 5 |
| 56 | 43 | 43 | 36 | 42 | 32 | 47 | 44 | 38 | 41 | 54 | 58 | 45 |
| 57 | 49 | 45 | 36 | 35 | 16 | 30 | 31 | 20 | 34 | 42 | 31 | 34 |
| 58 | 19 | 18 | 22 | 15 | 20 | 25 | 15 | 21 | 21 | 26 | 11 | 30 |
| 59 | 16 | 16 | 20 | 28 | 12 | 17 | 16 | 21 | 16 | 19 | 10 | 8 |
| 60 | 18 | 29 | 29 | 19 | 18 | 22 | 22 | 24 | 15 | 13 | 17 | 23 |
| 61 | 22 | 17 | 14 | 19 | 19 | 31 | 22 | 22 | 23 | 27 | 19 | 29 |
| 62 | 35 | 36 | 43 | 31 | 29 | 29 | 46 | 34 | 54 | 44 | 37 | 36 |
| 63 |  |  |  |  |  |  |  |  |  |  |  |  |
| 64 |  |  |  |  |  |  |  |  |  |  |  |  |
| 65 | 20 | 23 | 16 | 13 | 10 | 22 | 9 | 21 | 22 | 18 | 20 | 15 |
| 66 | 3 | 5 | 10 | 3 | 3 | 3 | 5 | 4 | 4 | 4 | 9 | 3 |
| 67 | 12 | 11 | 9 | 15 | 16 | 13 | 12 | 14 | 18 | 23 | 17 | 16 |
| 68 | 11 | 10 | 7 | 3 | 1 | 7 | 10 | 13 | 10 | 12 | 10 | 15 |
| 68 | 38 | 45 | 44 | 38 | 34 | 43 | 40 | 44 | 41 | 51 | 49 | 51 |
| 70 | 3 | 2 | 1 | 1 | 2 | 4 | 2 | 3 | 5 | 2 | 6 | 3 |
| 71 | 10 | 11 | 18 | 11 | 13 | 11 | 13 | 16 | 10 | 17 | 10 | 6 |
| 72 | 35 | 26 | 14 | 14 | 19 | 36 | 42 | 27 | 28 | 34 | 30 | 30 |
| 73 | 17 | 14 | 11 | 9 | 8 | 15 | 12 | 8 | 19 | 15 | 23 | 10 |
| 74 | 39 | 38 | 42 | 48 | 30 | 38 | 33 | 39 | 50 | 42 | 37 | 26 |
| 75 |  |  |  |  |  |  |  |  |  |  |  |  |
| 76 | 17 | 17 | 16 | 30 | 11 | 14 | 16 | 16 | 26 | 19 | 17 | 23 |
| 77 | 2 | 1 | 3 | 1 | 2 | 3 | 1 | 1 | 8 | 0 | 1 | 6 |
| 78 |  |  |  |  |  |  |  |  |  | 1 |  |  |
| 79 | 22 | 23 | 21 | 17 | 13 | 17 | 27 | 28 | 32 | 31 | 20 | 16 |
| 80 | 26 | 10 | 16 | 16 | 11 | 22 | 19 | 18 | 21 | 22 | 23 | 18 |
| 81 | 18 | 11 | 10 | 5 | 11 | 21 | 20 | 20 | 21 | 16 | 18 | 15 |
| 82 | 10 | 11 | 16 | 2 | 15 | 15 | 10 | 13 | 13 | 9 | 8 | 10 |
| 83 |  |  |  |  |  |  |  |  |  |  |  |  |
| 84 | 16 | 13 | 9 | 18 | 6 | 19 | 21 | 10 | 17 | 23 | 14 | 17 |
| 85 | 32 | 28 | 30 | 29 | 13 | 23 | 24 | 32 | 31 | 37 | 22 | 24 |
| 86 | 20 | 19 | 21 | 26 | 19 | 20 | 14 | 17 | 13 | 25 | 27 | 18 |
| 87 | 11 | 9 | 9 | 11 | 11 | 9 | 13 | 9 |  | 3 | 4 | 11 |
| 88 | 17 | 12 | 11 | 14 | 13 | 13 | 13 | 9 | 14 | 17 | 14 | 11 |
| 89 | 66 | 60 | 73 | 74 | 50 | 73 | 76 | 76 | 73 | 85 | 80 | 81 |
| 90 | 22 | 27 | 19 | 18 | 22 | 19 | 30 | 19 | 18 | 27 | 22 | 21 |
| 91 | 38 | 19 | 24 | 30 | 21 | 24 | 18 | 28 | 13 | 18 | 25 | 20 |
| 92 |  |  |  |  |  |  |  |  |  |  |  |  |
| 93 |  |  |  |  |  |  |  |  |  |  |  |  |
| 94 | 4 | 8 | 1 | 8 | 9 | 6 | 1 | 5 | 7 | 3 | 5 | 9 |
| 95 | 8 | 3 | 4 | 4 | 3 | 1 | 4 | 4 | 2 | 4 | 4 | 5 |
| 96 | 13 | 17 | 20 | 18 | 9 | 14 | 15 | 12 | 12 | 15 | 20 | 11 |
| 97 |  |  |  |  |  |  |  |  |  |  |  |  |
| 98 | 13 | 16 | 10 | 4 | 9 | 7 | 10 | 6 | 11 | 5 | 12 | 8 |
| 99 | 7 | 7 | 3 | 5 | 8 | 8 | 14 | 7 | 7 | 12 | 9 | 6 |
| 100 | 21 | 16 | 28 | 49 | 35 | 30 | 22 | 18 | 25 | 24 | 16 | 22 |
| 101 | 23 | 16 | 24 | 19 | 18 | 17 | 23 | 26 | 16 | 26 | 27 | 28 |
| 102 |  |  |  |  |  |  |  |  |  |  |  |  |
| 103 | 9 | 15 | 11 | 9 | 18 | 21 | 20 | 16 | 12 | 7 | 22 | 17 |
| 104 | 16 | 24 | 24 | 17 | 11 | 34 | 26 | 21 | 28 | 32 | 26 | 27 |
| 105 | 16 | 8 | 11 | 13 | 14 | 9 | 14 | 10 | 21 | 17 | 10 | 13 |

Table 23. Number of newly diagnosed stage I lung cancer during each month in 2019.

|  | Month | | | | | | | | | | | |
| --- | --- | --- | --- | --- | --- | --- | --- | --- | --- | --- | --- | --- |
| Hospital | 1 | 2 | 3 | 4 | 5 | 6 | 7 | 8 | 9 | 10 | 11 | 12 |
| 1 | 1 | 5 | 3 | 3 | 4 | 7 | 4 | 3 | 10 | 11 | 13 | 11 |
| 2 | 24 | 19 | 25 | 26 | 12 | 24 | 19 | 17 | 21 | 30 | 25 | 25 |
| 3 | 0 | 0 | 0 | 2 | 3 | 0 | 0 | 1 | 0 | 0 | 2 | 0 |
| 4 | 11 | 14 | 12 | 12 | 6 | 10 | 9 | 13 | 11 | 10 | 16 | 10 |
| 5 | 3 | 3 | 4 | 5 | 3 | 5 | 4 | 4 | 1 | 7 | 0 | 2 |
| 6 | 7 | 10 | 12 | 6 | 6 | 7 | 8 | 8 | 5 | 11 | 6 | 7 |
| 7 | 6 | 5 | 7 | 9 | 3 | 2 | 5 | 3 | 5 | 4 | 8 | 3 |
| 8 |  |  |  |  |  |  |  |  |  |  |  |  |
| 9 | 10 | 4 | 1 | 4 | 3 | 6 | 5 | 2 | 11 | 8 | 9 | 6 |
| 10 | 5 | 3 | 5 | 1 | 1 | 5 | 10 | 6 | 6 | 3 | 4 | 3 |
| 11 | 19 | 14 | 12 | 19 | 8 | 13 | 11 | 9 | 8 | 14 | 12 | 5 |
| 12 | 0 | 0 | 1 | 0 | 3 | 2 | 0 | 1 | 2 | 1 | 1 | 0 |
| 13 | 10 | 10 | 15 | 9 | 15 | 4 | 12 | 16 | 15 | 12 | 7 | 13 |
| 14 | 10 | 5 | 5 | 3 | 6 | 6 | 7 | 4 | 3 | 4 | 8 | 5 |
| 15 | 16 | 10 | 9 | 7 | 12 | 10 | 11 | 10 | 16 | 12 | 6 | 7 |
| 16 | 16 | 11 | 10 | 12 | 13 | 11 | 8 | 7 | 12 | 13 | 6 | 8 |
| 17 | 6 | 8 | 5 | 8 | 5 | 4 | 4 | 6 | 3 | 3 | 6 | 9 |
| 18 | 2 | 9 | 5 | 10 | 6 | 4 | 4 | 8 | 8 | 7 | 9 | 3 |
| 19 | 3 | 2 | 9 | 2 | 3 | 3 | 4 | 1 | 3 | 5 | 3 | 2 |
| 20 | 40 | 47 | 44 | 34 | 30 | 48 | 47 | 47 | 41 | 46 | 47 | 40 |
| 21 | 6 | 4 | 10 | 6 | 7 | 7 | 11 | 12 | 8 | 11 | 6 | 7 |
| 22 | 6 | 5 | 6 | 5 | 5 | 8 | 7 | 6 | 4 | 12 | 9 | 3 |
| 23 |  |  |  |  |  |  |  |  |  |  |  |  |
| 24 | 5 | 13 | 4 | 15 | 11 | 7 | 18 | 11 | 10 | 6 | 10 | 12 |
| 25 | 5 | 4 | 4 | 3 | 7 | 7 | 3 | 9 | 10 | 2 | 8 | 7 |
| 26 | 6 | 5 | 10 | 7 | 4 | 8 | 5 | 11 | 5 | 14 | 13 | 9 |
| 27 | 9 | 4 | 4 | 7 | 7 | 6 | 5 | 8 | 4 | 7 | 9 | 12 |
| 28 | 4 | 6 | 7 | 6 | 5 | 8 | 8 | 15 | 5 | 7 | 13 | 3 |
| 29 |  |  |  |  |  |  |  |  |  |  |  |  |
| 30 | 8 | 6 | 4 | 8 | 4 | 7 | 11 | 6 | 9 | 3 | 14 | 4 |
| 31 | 4 | 9 | 10 | 9 | 4 | 5 | 9 | 1 | 4 | 4 | 6 | 5 |
| 32 | 3 | 4 | 3 | 2 | 1 | 1 | 2 | 2 | 2 | 2 | 1 | 3 |
| 33 | 2 | 4 | 3 | 4 | 4 | 7 | 6 | 6 | 8 | 4 | 5 | 7 |
| 34 |  |  |  |  |  |  |  |  |  |  |  |  |
| 35 | 5 | 7 | 7 | 6 | 7 | 12 | 7 | 2 | 2 | 5 | 8 | 8 |
| 36 | 11 | 7 | 9 | 8 | 13 | 6 | 7 | 11 | 10 | 10 | 10 | 7 |
| 37 | 24 | 12 | 14 | 17 | 17 | 16 | 24 | 22 | 26 | 27 | 22 | 17 |
| 38 |  |  |  |  |  |  |  |  |  |  |  |  |
| 39 | 1 | 1 |  |  |  |  |  | 1 |  |  | 1 | 1 |
| 40 | 12 | 14 | 6 | 12 | 15 | 11 | 13 | 15 | 10 | 10 | 11 | 12 |
| 41 | 4 | 7 | 3 | 11 | 3 | 2 | 5 | 6 | 1 | 5 | 10 | 3 |
| 42 | 7 | 6 | 10 | 7 | 4 | 8 | 9 | 8 | 8 | 6 | 5 | 13 |
| 43 | 5 | 8 | 11 | 6 | 14 | 9 | 10 | 5 | 7 | 8 | 11 | 3 |
| 44 | 0 | 4 | 3 | 4 | 2 | 1 | 6 | 4 | 3 | 2 | 4 | 4 |
| 45 | 7 | 6 | 5 | 6 | 3 | 5 | 4 | 3 | 7 | 3 | 10 | 6 |
| 46 | 4 | 0 | 5 | 4 | 4 | 6 | 2 | 4 | 7 | 5 | 3 | 5 |
| 47 |  |  |  |  |  |  |  |  |  |  |  |  |
| 48 | 5 | 15 | 7 | 10 | 10 | 9 | 13 | 16 | 6 | 10 | 7 | 14 |
| 49 | 5 | 4 | 2 | 4 | 3 | 8 | 2 | 3 | 2 | 2 | 7 | 7 |
| 50 | 0 | 1 | 0 | 1 | 0 | 0 | 1 | 0 | 0 | 0 | 0 | 0 |
| 51 | 6 | 10 | 9 | 7 | 4 | 14 | 9 | 11 | 14 | 13 | 13 | 8 |
| 52 | 9 | 8 | 11 | 8 | 2 | 13 | 12 | 6 | 19 | 15 | 13 | 15 |
| 53 | 10 | 6 | 13 | 9 | 11 | 13 | 12 | 5 | 10 | 11 | 14 | 17 |
| 54 | 29 | 22 | 25 | 23 | 26 | 19 | 28 | 26 | 22 | 34 | 30 | 26 |
| 55 | 8 | 10 | 8 | 1 | 6 | 4 | 4 | 6 | 6 | 6 | 10 | 10 |
| 56 | 21 | 30 | 36 | 21 | 20 | 19 | 27 | 24 | 25 | 33 | 23 | 26 |
| 57 | 16 | 8 | 17 | 17 | 10 | 11 | 18 | 16 | 15 | 23 | 18 | 23 |
| 58 | 9 | 3 | 4 | 10 | 19 | 10 | 9 | 7 | 8 | 15 | 15 | 6 |
| 59 | 9 | 11 | 8 | 16 | 4 | 6 | 12 | 16 | 11 | 13 | 5 | 9 |
| 60 | 8 | 11 | 9 | 4 | 10 | 9 | 8 | 8 | 6 | 8 | 11 | 8 |
| 61 | 9 | 4 | 7 | 7 | 8 | 5 | 7 | 6 | 7 | 9 | 14 | 7 |
| 62 |  |  |  |  |  |  |  |  |  |  |  |  |
| 63 |  |  |  |  |  |  |  |  |  |  |  |  |
| 64 | 11 | 12 | 7 | 11 | 10 | 13 | 8 | 10 | 5 | 8 | 15 | 11 |
| 65 | 6 | 9 | 11 | 4 | 8 | 6 | 11 | 9 | 7 | 19 | 16 | 10 |
| 66 | 1 | 1 | 0 | 0 | 2 | 0 | 4 | 1 | 2 | 2 | 0 | 2 |
| 67 | 3 | 6 | 8 | 7 | 6 | 11 | 9 | 5 | 6 | 15 | 9 | 7 |
| 68 | 5 | 3 | 2 | 2 | 2 | 3 | 3 | 4 | 5 | 5 | 2 | 4 |
| 68 | 2 | 5 | 4 | 7 | 3 | 1 | 4 | 2 | 3 | 2 | 3 | 1 |
| 70 | 1 |  |  | 1 | 1 | 1 | 2 |  |  | 1 |  |  |
| 71 | 2 | 2 | 0 | 1 | 1 | 2 | 3 | 7 | 1 | 5 | 4 | 3 |
| 72 | 14 | 9 | 11 | 5 | 8 | 9 | 10 | 13 | 7 | 7 | 11 | 16 |
| 73 | 5 | 3 | 7 | 5 | 8 | 9 | 5 | 4 | 10 | 8 | 13 | 2 |
| 74 | 8 | 11 | 21 | 13 | 7 | 9 | 18 | 21 | 15 | 9 | 11 | 21 |
| 75 |  |  |  |  |  |  |  |  |  |  |  |  |
| 76 | 6 | 6 | 7 | 11 | 6 | 5 | 6 | 3 | 6 | 10 | 5 | 5 |
| 77 |  | 1 | 1 | 1 |  |  |  | 1 |  |  | 2 |  |
| 78 |  |  |  | 2 | 1 |  | 1 | 2 |  |  |  |  |
| 79 | 14 | 11 | 11 | 13 | 19 | 6 | 21 | 8 | 13 | 9 | 12 | 6 |
| 80 | 6 | 2 | 3 | 4 | 8 | 5 | 15 | 12 | 14 | 10 | 14 | 9 |
| 81 | 5 | 2 | 9 | 4 | 5 | 5 | 4 | 6 | 8 | 7 | 3 | 2 |
| 82 | 6 | 7 | 13 | 10 | 5 | 8 | 5 | 4 | 7 | 7 | 4 | 7 |
| 83 | 7 | 3 | 5 | 6 | 4 | 2 | 3 | 4 | 3 | 4 | 4 | 12 |
| 84 | 4 | 10 | 6 | 10 | 5 | 5 | 5 | 7 | 7 | 6 | 6 | 5 |
| 85 | 9 | 12 | 13 | 10 | 5 | 7 | 6 | 4 | 14 | 11 | 7 | 3 |
| 86 | 8 | 8 | 7 | 10 | 7 | 11 | 17 | 12 | 18 | 18 | 11 | 8 |
| 87 | 2 | 1 | 3 | 2 | 3 | 2 | 6 | 5 | 1 | 6 | 3 | 2 |
| 88 | 0 | 0 | 4 | 9 | 3 | 2 | 8 | 11 | 8 | 5 | 9 | 5 |
| 89 | 36 | 28 | 17 | 27 | 32 | 19 | 28 | 26 | 20 | 33 | 24 | 30 |
| 90 | 6 | 7 | 9 | 6 | 4 | 7 | 3 | 3 | 8 | 12 | 14 | 9 |
| 91 | 6 | 7 | 9 | 6 | 8 | 3 | 9 | 9 | 3 | 3 | 5 | 9 |
| 92 |  |  |  |  |  |  |  |  |  |  |  |  |
| 93 | 4 | 3 | 5 | 7 | 0 | 4 | 2 | 2 | 3 | 4 | 1 | 2 |
| 94 | 0 | 1 | 5 | 3 | 3 | 1 | 0 | 0 | 1 | 2 | 3 | 1 |
| 95 | 0 | 0 | 1 | 0 | 1 | 3 | 0 | 0 | 0 | 1 | 1 | 0 |
| 96 | 3 | 6 | 3 | 3 | 3 | 8 | 2 | 4 | 6 | 5 | 6 | 5 |
| 97 |  |  |  |  |  |  |  |  |  |  |  |  |
| 98 | 1 | 4 | 5 | 1 | 2 | 9 | 4 | 1 | 2 | 7 | 5 | 3 |
| 99 | 1 | 3 | 0 | 2 | 3 | 3 | 1 | 3 | 2 | 2 | 2 | 10 |
| 100 | 9 | 17 | 15 | 12 | 12 | 7 | 12 | 9 | 7 | 13 | 12 | 8 |
| 101 |  |  | 1 | 4 | 2 | 2 | 3 | 3 | 1 | 1 | 3 | 1 |
| 102 |  |  |  |  |  |  |  |  |  |  |  |  |
| 103 | 2 | 5 | 5 | 5 | 4 | 6 | 5 | 6 | 6 | 5 | 8 | 5 |
| 104 | 7 | 5 | 11 | 8 | 8 | 7 | 13 | 5 | 5 | 9 | 8 | 6 |
| 105 | 4 | 4 | 0 | 4 | 7 | 7 | 8 | 3 | 8 | 4 | 6 | 14 |

Table 24. Number of newly diagnosed stage I lung cancer during each month in 2020.

|  | Month | | | | | | | | | | | |
| --- | --- | --- | --- | --- | --- | --- | --- | --- | --- | --- | --- | --- |
| Hospital | 1 | 2 | 3 | 4 | 5 | 6 | 7 | 8 | 9 | 10 | 11 | 12 |
| 1 | 7 | 5 | 6 | 4 | 6 | 9 | 7 | 4 | 2 | 8 | 4 | 3 |
| 2 | 21 | 15 | 15 | 21 | 11 | 13 | 14 | 15 | 15 | 19 | 21 | 24 |
| 3 | 0 | 0 | 0 | 0 | 0 | 2 | 0 | 0 | 0 | 0 | 0 | 2 |
| 4 | 6 | 7 | 3 | 7 | 10 | 16 | 12 | 9 | 13 | 8 | 14 | 9 |
| 5 | 5 | 2 | 4 | 1 | 3 | 6 | 4 | 4 | 3 | 5 | 7 | 3 |
| 6 | 13 | 3 | 6 | 3 | 3 | 8 | 8 | 4 | 8 | 8 | 7 | 7 |
| 7 | 7 | 3 | 5 | 4 | 3 | 2 | 3 | 4 | 7 | 4 | 5 | 3 |
| 8 |  |  |  |  |  |  |  |  |  |  |  |  |
| 9 | 10 | 10 | 7 | 8 | 3 | 5 | 4 | 2 | 8 | 5 | 5 | 3 |
| 10 | 2 | 5 | 6 | 4 | 4 | 3 | 7 | 5 | 5 | 5 | 2 | 1 |
| 11 | 15 | 19 | 9 | 12 | 7 | 9 | 14 | 10 | 11 | 14 | 7 | 12 |
| 12 | 1 | 2 | 0 | 1 | 0 | 0 | 2 | 2 | 0 | 4 | 1 | 0 |
| 13 | 9 | 11 | 9 | 7 | 3 | 11 | 19 | 7 | 10 | 13 | 6 | 7 |
| 14 | 6 | 4 | 3 | 4 | 2 | 9 | 4 | 8 | 6 | 10 | 2 | 7 |
| 15 | 7 | 9 | 5 | 12 | 5 | 6 | 9 | 13 | 9 | 9 | 11 | 12 |
| 16 | 6 | 12 | 5 | 11 | 3 | 9 | 10 | 4 | 6 | 11 | 6 | 9 |
| 17 | 7 | 3 | 3 | 3 | 0 | 3 | 4 | 4 | 4 | 11 | 6 | 9 |
| 18 | 3 | 4 | 6 | 4 | 4 | 5 | 7 | 7 | 9 | 8 | 7 | 5 |
| 19 | 4 | 1 | 4 | 7 | 5 | 3 | 1 | 1 | 5 | 3 | 6 | 3 |
| 20 | 48 | 55 | 36 | 27 | 24 | 49 | 49 | 36 | 55 | 53 | 46 | 45 |
| 21 | 4 | 6 | 5 | 3 | 3 | 12 | 2 | 6 | 9 | 11 | 0 | 0 |
| 22 | 3 | 2 | 11 | 2 | 7 | 3 | 5 | 6 |  | 5 | 5 | 4 |
| 23 |  |  |  |  |  |  |  |  |  |  |  |  |
| 24 | 9 | 16 | 13 | 10 | 7 | 14 | 9 | 9 | 9 | 15 | 8 | 4 |
| 25 | 2 | 2 | 4 | 2 | 4 | 5 | 3 | 0 | 2 | 7 | 6 | 3 |
| 26 | 6 | 6 | 8 | 8 | 3 | 6 | 4 | 2 | 3 | 15 | 15 | 7 |
| 27 | 6 | 4 | 8 | 7 | 7 | 8 | 2 | 3 | 7 | 12 | 7 | 12 |
| 28 | 2 | 5 | 5 | 5 | 2 | 11 | 6 | 11 | 6 | 7 | 7 | 8 |
| 29 |  |  |  |  |  |  |  |  |  |  |  |  |
| 30 | 7 | 3 | 9 | 7 | 4 | 5 | 5 | 4 | 8 | 7 | 8 | 8 |
| 31 | 5 | 7 | 9 | 9 | 4 | 7 | 2 | 6 | 4 | 3 | 12 | 4 |
| 32 |  | 2 | 1 | 3 |  | 2 | 2 |  | 2 | 2 | 2 | 6 |
| 33 | 3 | 5 | 7 | 1 | 2 | 2 | 3 | 6 | 12 | 7 | 4 | 4 |
| 34 |  |  |  |  |  |  |  |  |  |  |  |  |
| 35 | 7 | 10 | 7 | 8 | 4 | 8 | 13 | 4 | 8 | 5 | 2 | 3 |
| 36 | 7 | 10 | 8 | 15 | 11 | 10 | 8 | 9 | 16 | 10 | 10 | 13 |
| 37 | 13 | 16 | 24 | 18 | 11 | 19 | 23 | 22 | 27 | 27 | 17 | 20 |
| 38 |  |  |  |  |  |  |  |  |  |  |  |  |
| 39 |  |  |  |  |  |  |  |  |  |  |  |  |
| 40 | 13 | 13 | 10 | 9 | 2 | 4 | 8 | 5 | 8 | 16 | 14 | 9 |
| 41 | 3 | 3 | 4 | 5 | 3 | 2 | 8 | 3 | 8 | 8 | 6 | 9 |
| 42 | 6 | 6 | 7 | 6 | 2 | 6 | 7 | 5 | 8 | 6 | 7 | 6 |
| 43 | 5 | 7 | 9 | 11 | 8 | 8 | 7 | 4 | 6 | 6 | 6 | 7 |
| 44 | 2 | 3 | 2 | 1 | 2 | 4 | 3 | 3 | 2 | 5 | 4 | 5 |
| 45 | 6 | 3 | 6 | 2 | 4 | 4 | 6 | 4 | 2 | 9 | 2 | 0 |
| 46 | 6 | 5 | 1 | 2 | 4 | 3 | 5 | 2 | 2 | 7 | 7 | 6 |
| 47 |  |  |  |  |  |  |  |  |  |  |  |  |
| 48 | 12 | 13 | 10 | 7 | 6 | 6 | 10 | 7 | 5 | 6 | 15 | 11 |
| 49 | 6 | 6 | 5 | 4 | 2 | 8 | 2 | 2 | 4 | 5 | 4 | 2 |
| 50 | 0 | 2 | 1 | 0 | 0 | 2 | 0 | 0 | 0 | 0 | 1 | 1 |
| 51 | 7 | 3 | 6 | 11 | 6 | 6 | 6 | 8 | 8 | 9 | 10 | 7 |
| 52 | 10 | 9 | 8 | 9 | 7 | 7 | 7 | 10 | 12 | 17 | 9 | 9 |
| 53 | 6 | 6 | 6 | 11 | 12 | 6 | 16 | 9 | 7 | 14 | 9 | 18 |
| 54 | 23 | 24 | 18 | 22 | 24 | 26 | 18 | 17 | 25 | 33 | 22 | 28 |
| 55 | 1 | 11 | 7 | 6 | 4 | 3 | 9 | 3 | 4 | 10 | 10 | 2 |
| 56 | 19 | 20 | 14 | 26 | 11 | 19 | 16 | 21 | 24 | 28 | 28 | 22 |
| 57 | 16 | 16 | 20 | 12 | 8 | 12 | 11 | 8 | 13 | 17 | 13 | 12 |
| 58 | 11 | 8 | 8 | 8 | 8 | 13 | 8 | 12 | 14 | 12 | 2 | 14 |
| 59 | 10 | 6 | 9 | 9 | 3 | 9 | 8 | 8 | 8 | 10 | 7 | 6 |
| 60 | 6 | 10 | 11 | 12 | 10 | 12 | 12 | 11 | 5 | 5 | 10 | 8 |
| 61 | 5 | 7 | 5 | 5 | 6 | 13 | 12 | 12 | 10 | 15 | 11 | 15 |
| 62 |  |  |  |  |  |  |  |  |  |  |  |  |
| 63 |  |  |  |  |  |  |  |  |  |  |  |  |
| 64 | 14 | 19 | 12 | 11 | 5 | 15 | 15 | 12 | 12 | 17 | 18 | 17 |
| 65 | 13 | 12 | 8 | 5 | 4 | 11 | 2 | 7 | 13 | 10 | 6 | 6 |
| 66 | 1 | 3 | 6 | 1 | 1 | 1 | 1 | 1 | 1 | 4 | 3 | 2 |
| 67 | 5 | 6 | 2 | 1 | 6 | 2 | 4 | 2 | 8 | 4 | 8 | 4 |
| 68 | 1 | 1 | 0 | 0 | 0 | 0 | 2 | 1 | 2 | 5 | 1 | 3 |
| 68 | 6 | 5 | 11 | 8 | 6 | 9 | 9 | 7 | 4 | 8 | 6 | 6 |
| 70 | 2 |  |  |  |  | 1 |  | 1 | 2 |  | 1 | 1 |
| 71 | 3 | 2 | 6 | 3 | 7 | 1 | 7 | 2 | 6 | 5 | 4 | 4 |
| 72 | 11 | 10 | 7 | 5 | 10 | 12 | 16 | 11 | 11 | 14 | 10 | 12 |
| 73 | 4 | 6 | 4 | 3 | 3 | 4 | 5 | 3 | 11 | 4 | 11 | 2 |
| 74 | 19 | 18 | 21 | 24 | 13 | 13 | 14 | 18 | 10 | 20 | 14 | 8 |
| 75 |  |  |  |  |  |  |  |  |  |  |  |  |
| 76 | 8 | 5 | 6 | 10 | 3 | 4 | 6 | 7 | 11 | 8 | 4 | 8 |
| 77 |  |  | 2 |  |  | 1 |  |  | 4 |  |  | 1 |
| 78 |  |  |  |  | 1 |  |  |  | 1 | 1 | 1 |  |
| 79 | 6 | 10 | 7 | 9 | 6 | 5 | 16 | 12 | 12 | 13 | 8 | 8 |
| 80 | 9 | 4 | 5 | 6 | 5 | 8 | 6 | 9 | 8 | 9 | 14 | 9 |
| 81 | 8 | 5 | 4 | 1 | 4 | 5 | 7 | 10 | 6 | 2 | 7 | 6 |
| 82 | 6 | 6 | 5 | 1 | 8 | 5 | 4 | 4 | 7 | 3 | 6 | 6 |
| 83 | 5 | 5 | 3 | 2 | 2 | 5 | 1 | 5 | 4 | 2 | 6 | 2 |
| 84 | 5 | 5 | 4 | 7 | 1 | 7 | 6 | 4 | 10 | 8 | 6 | 4 |
| 85 | 9 | 11 | 9 | 8 | 3 | 8 | 9 | 9 | 6 | 11 | 6 | 9 |
| 86 | 13 | 10 | 11 | 12 | 11 | 6 | 7 | 7 | 5 | 13 | 12 | 7 |
| 87 | 2 | 1 | 1 | 5 | 1 | 2 | 3 | 3 |  |  | 2 | 3 |
| 88 | 5 | 4 | 5 | 5 | 4 | 5 | 2 | 3 | 7 | 5 | 5 | 5 |
| 89 | 22 | 30 | 27 | 30 | 21 | 27 | 26 | 35 | 31 | 37 | 32 | 35 |
| 90 | 11 | 11 | 10 | 8 | 11 | 8 | 10 | 5 | 7 | 13 | 10 | 13 |
| 91 | 10 | 0 | 9 | 7 | 7 | 8 | 7 | 10 | 5 | 8 | 9 | 5 |
| 92 |  |  |  |  |  |  |  |  |  |  |  |  |
| 93 | 1 | 2 | 8 | 4 | 4 | 5 | 5 | 3 | 3 | 5 | 4 | 2 |
| 94 | 1 | 1 | 0 | 0 | 2 | 3 | 0 | 2 | 1 | 1 | 2 | 4 |
| 95 | 3 | 2 | 1 | 3 | 1 | 0 | 1 | 1 | 0 | 1 | 0 | 3 |
| 96 | 7 | 7 | 9 | 7 | 4 | 6 | 6 | 5 | 5 | 5 | 4 | 5 |
| 97 |  |  |  |  |  |  |  |  |  |  |  |  |
| 98 | 10 | 4 | 5 | 0 | 2 | 0 | 4 | 4 | 7 | 3 | 2 | 3 |
| 99 | 4 | 3 | 2 | 4 | 1 | 4 | 4 | 3 | 3 | 7 | 3 | 4 |
| 100 | 8 | 4 | 15 | 18 | 9 | 15 | 13 | 8 | 13 | 13 | 8 | 14 |
| 101 | 2 | 2 | 6 | 4 | 4 | 1 | 2 | 3 | 3 | 3 | 4 | 6 |
| 102 |  |  |  |  |  |  |  |  |  |  |  |  |
| 103 | 4 | 5 | 2 | 4 | 3 | 8 | 9 | 5 | 4 | 4 | 9 | 8 |
| 104 | 5 | 10 | 7 | 5 | 2 | 14 | 7 | 8 | 9 | 14 | 10 | 10 |
| 105 | 9 | 4 | 7 | 5 | 6 | 5 | 5 | 6 | 5 | 4 | 4 | 5 |

Table 25. Number of newly diagnosed stage II lung cancer during each month in 2019.

|  | Month | | | | | | | | | | | |
| --- | --- | --- | --- | --- | --- | --- | --- | --- | --- | --- | --- | --- |
| Hospital | 1 | 2 | 3 | 4 | 5 | 6 | 7 | 8 | 9 | 10 | 11 | 12 |
| 1 | 0 | 1 | 1 | 1 | 1 | 0 | 1 | 1 | 0 | 1 | 0 | 1 |
| 2 | 1 | 8 | 3 | 2 | 6 | 5 | 6 | 5 | 2 | 5 | 4 | 4 |
| 3 | 0 | 0 | 0 | 0 | 0 | 0 | 1 | 0 | 0 | 0 | 0 | 0 |
| 4 | 2 | 2 | 1 | 1 | 0 | 2 | 0 | 3 | 2 | 0 | 4 | 0 |
| 5 | 2 | 1 | 1 | 0 | 0 | 1 | 0 | 1 | 1 | 1 | 0 | 0 |
| 6 | 3 | 1 | 2 | 1 | 3 | 1 |  | 2 |  | 3 | 1 | 2 |
| 7 | 2 | 1 | 2 | 0 | 2 | 3 | 1 | 0 | 2 | 3 | 0 | 0 |
| 8 |  |  |  |  |  |  |  |  |  |  |  |  |
| 9 | 1 | 0 | 1 | 3 | 3 | 1 | 0 | 2 | 3 | 0 | 1 | 1 |
| 10 | 2 | 0 | 0 | 0 | 2 | 1 | 0 | 0 | 0 | 1 | 1 | 0 |
| 11 | 4 | 3 | 4 | 5 | 2 | 1 | 4 | 9 | 5 | 1 | 3 | 2 |
| 12 | 1 | 0 | 0 | 0 | 0 | 0 | 2 | 1 | 0 | 0 | 0 | 0 |
| 13 | 1 | 2 | 3 | 3 | 3 | 3 | 1 | 2 | 2 | 4 | 6 | 1 |
| 14 | 0 | 2 | 4 | 1 | 2 | 0 | 0 | 1 | 3 | 2 | 0 | 2 |
| 15 | 4 | 0 | 7 | 1 | 2 | 2 | 5 | 6 | 7 | 5 | 1 | 2 |
| 16 | 4 | 2 | 1 | 4 | 1 | 1 | 4 | 2 | 2 | 2 | 2 | 4 |
| 17 |  |  | 1 |  |  | 1 | 2 | 1 | 1 |  |  | 4 |
| 18 | 0 | 1 | 1 | 0 | 2 | 1 | 1 | 1 | 0 | 3 | 0 | 0 |
| 19 | 2 | 0 | 1 | 1 | 0 | 0 | 3 | 0 | 1 | 0 | 1 | 0 |
| 20 | 10 | 8 | 7 | 6 | 12 | 12 | 6 | 8 | 6 | 11 | 12 | 13 |
| 21 | 2 | 2 | 1 | 0 | 111 | 2 | 2 | 1 | 1 | 0 | 1 | 2 |
| 22 | 4 | 3 | 1 | 1 | 2 | 2 | 2 | 2 | 3 | 3 | 1 | 1 |
| 23 |  |  |  |  |  |  |  |  |  |  |  |  |
| 24 | 7 | 2 | 1 | 3 | 3 | 2 | 5 | 3 | 4 | 8 | 2 | 5 |
| 25 | 1 | 0 | 6 | 0 | 1 | 0 | 0 | 1 | 1 | 1 | 2 | 0 |
| 26 | 2 | 4 |  | 2 | 1 | 1 | 2 | 3 | 1 | 1 | 9 | 1 |
| 27 | 0 | 0 | 1 | 0 | 1 | 1 | 0 | 4 | 1 | 3 | 0 | 0 |
| 28 | 0 | 0 | 1 | 0 | 1 | 0 | 1 | 2 | 2 | 4 | 5 | 1 |
| 29 |  |  |  |  |  |  |  |  |  |  |  |  |
| 30 | 2 | 0 | 1 | 2 | 0 | 1 | 1 | 0 | 0 | 0 | 3 | 0 |
| 31 | 1 | 2 | 3 | 0 | 1 | 1 | 1 | 1 | 3 | 3 | 2 | 1 |
| 32 | 1 |  | 2 |  | 1 |  | 2 | 1 |  | 1 | 1 |  |
| 33 | 0 | 6 | 0 | 0 | 2 | 0 | 1 | 1 | 2 | 1 | 2 | 2 |
| 34 |  |  |  |  |  |  |  |  |  |  |  |  |
| 35 | 4 | 1 | 0 | 0 | 0 | 1 | 0 | 1 | 1 | 5 | 1 | 2 |
| 36 |  | 2 |  |  | 1 | 1 | 1 |  | 2 |  |  |  |
| 37 | 7 | 2 | 6 | 2 | 1 | 2 | 4 | 2 | 7 | 5 | 6 | 3 |
| 38 |  |  |  |  |  |  |  |  |  |  |  |  |
| 39 |  |  |  |  |  |  |  | 1 |  |  |  |  |
| 40 | 1 | 2 | 4 | 4 | 2 | 1 | 1 | 3 | 1 | 3 | 2 | 3 |
| 41 | 2 | 1 | 2 | 0 | 0 | 1 | 2 | 2 | 1 | 0 | 0 | 2 |
| 42 | 3 | 2 | 0 | 2 | 1 | 5 | 1 | 2 | 1 | 1 | 1 | 1 |
| 43 | 1 | 1 | 1 | 2 | 0 | 3 | 3 | 1 | 2 | 2 | 4 | 1 |
| 44 | 2 | 0 | 1 | 1 | 0 | 1 | 0 | 1 | 0 | 2 | 2 | 0 |
| 45 | 1 | 1 | 1 | 0 | 1 | 0 | 0 | 1 | 1 | 0 | 0 | 4 |
| 46 | 0 | 1 | 0 | 1 | 0 | 2 | 4 | 1 | 0 | 1 | 0 | 0 |
| 47 |  |  |  |  |  |  |  |  |  |  |  |  |
| 48 | 3 | 3 | 1 | 3 | 1 | 1 | 3 | 3 | 1 | 1 | 4 | 2 |
| 49 | 1 | 0 | 1 | 0 | 0 | 1 | 1 | 1 | 1 | 1 | 1 | 1 |
| 50 | 0 | 0 | 0 | 0 | 1 | 0 | 0 | 0 | 0 | 0 | 0 | 0 |
| 51 | 0 | 1 | 3 | 1 | 1 | 1 | 2 | 2 | 0 | 0 | 1 | 2 |
| 52 | 2 | 3 | 2 | 2 | 4 | 2 | 3 | 1 | 3 | 0 | 3 | 1 |
| 53 | 3 | 5 | 0 | 2 | 4 | 1 | 5 | 3 | 2 | 6 | 1 | 4 |
| 54 | 7 | 6 | 7 | 3 | 5 | 6 | 7 | 9 | 2 | 13 | 11 | 3 |
| 55 | 1 | 2 | 1 | 1 | 2 | 1 | 1 | 3 | 0 | 1 | 0 | 2 |
| 56 | 6 | 4 | 1 | 5 | 4 | 5 | 3 | 3 | 2 | 4 | 5 | 6 |
| 57 | 1 | 2 | 4 | 4 | 3 | 5 | 3 | 2 | 4 | 6 | 4 | 5 |
| 58 | 0 | 0 | 0 | 1 | 3 | 1 | 2 | 1 | 2 | 1 | 3 | 0 |
| 59 | 4 | 0 | 1 | 0 | 2 | 0 | 1 | 3 | 5 | 2 | 1 | 2 |
| 60 | 2 | 3 | 2 | 0 | 1 | 3 | 4 | 1 | 0 | 1 | 2 | 2 |
| 61 | 0 | 0 | 2 | 2 | 0 | 2 | 0 | 1 | 1 | 6 | 2 | 2 |
| 62 |  |  |  |  |  |  |  |  |  |  |  |  |
| 63 |  |  |  |  |  |  |  |  |  |  |  |  |
| 64 | 4 | 1 | 1 | 4 | 2 | 3 | 4 | 2 | 4 | 1 | 3 | 2 |
| 65 | 1 | 1 | 2 | 1 | 2 | 1 | 1 | 4 | 1 | 3 | 2 | 2 |
| 66 | 0 | 0 | 0 | 0 | 0 | 0 | 1 | 1 | 0 | 1 | 1 | 0 |
| 67 | 2 | 2 | 3 | 2 | 1 | 3 | 3 | 1 | 7 | 1 | 2 | 1 |
| 68 | 0 | 0 | 0 | 3 | 1 | 1 | 1 | 0 | 2 | 0 | 0 | 0 |
| 68 | 4 | 3 | 1 | 1 | 3 | 5 | 2 | 4 | 1 | 0 | 0 | 3 |
| 70 |  |  | 1 |  | 1 |  | 2 |  |  |  |  |  |
| 71 | 2 | 0 | 2 | 2 | 1 | 0 | 1 | 0 | 1 | 1 | 1 | 2 |
| 72 | 2 | 1 | 3 | 2 | 2 | 4 | 3 | 2 | 4 | 1 | 3 | 5 |
| 73 | 1 | 3 | 4 | 1 | 0 | 1 | 1 | 2 | 1 | 0 | 3 | 1 |
| 74 | 2 | 5 | 0 | 3 | 2 | 5 | 1 | 3 | 3 | 6 | 2 | 4 |
| 75 |  |  |  |  |  |  |  |  |  |  |  |  |
| 76 | 2 | 3 | 0 | 0 | 2 | 3 | 1 | 0 | 1 | 1 | 3 | 4 |
| 77 |  |  |  |  |  |  |  | 1 |  | 1 |  |  |
| 78 |  |  |  |  |  |  | 1 |  | 1 |  | 1 |  |
| 79 | 2 | 1 | 1 | 1 | 3 | 2 | 0 | 4 | 2 | 2 | 5 | 4 |
| 80 | 3 | 3 | 3 | 1 | 6 | 1 | 3 | 0 | 1 | 0 | 2 | 1 |
| 81 |  |  | 2 | 2 | 3 | 1 |  | 1 | 1 | 4 | 1 | 7 |
| 82 | 1 | 1 | 3 | 1 | 0 | 1 | 2 | 1 | 1 | 1 | 5 | 3 |
| 83 | 2 | 0 | 1 | 0 | 0 | 1 | 1 | 3 | 1 | 0 | 0 | 1 |
| 84 | 1 | 2 | 3 | 2 | 0 | 0 | 1 | 0 | 0 | 1 | 3 | 2 |
| 85 | 1 | 2 | 2 | 2 | 1 | 6 | 2 | 2 | 3 |  | 2 | 2 |
| 86 | 2 | 0 | 1 | 1 | 0 | 0 | 4 | 0 | 1 | 3 | 4 | 1 |
| 87 | 1 | 2 |  |  |  |  |  | 2 | 2 |  | 1 | 1 |
| 88 | 1 | 1 | 1 | 2 | 0 | 0 | 1 | 1 | 0 | 4 | 0 | 2 |
| 89 | 6 | 15 | 6 | 8 | 2 | 10 | 9 | 8 | 5 | 7 | 10 | 11 |
| 90 | 2 | 2 | 1 | 1 | 1 | 0 | 5 | 2 | 2 | 1 | 1 | 2 |
| 91 | 0 | 3 | 4 | 3 | 2 | 1 | 5 | 2 | 2 | 3 | 1 | 3 |
| 92 |  |  |  |  |  |  |  |  |  |  |  |  |
| 93 | 2 | 0 | 0 | 0 | 1 | 0 | 1 | 0 | 0 | 1 | 1 | 1 |
| 94 | 0 | 0 | 2 | 0 | 0 | 0 | 0 | 0 | 0 | 2 | 2 | 1 |
| 95 | 0 | 0 | 1 | 0 | 1 | 0 | 0 | 0 | 0 | 1 | 2 | 0 |
| 96 | 2 | 0 | 0 | 2 | 0 | 0 | 0 | 2 | 0 | 0 | 1 | 0 |
| 97 |  |  |  |  |  |  |  |  |  |  |  |  |
| 98 | 0 | 0 | 0 | 1 | 0 | 2 | 2 | 3 | 1 | 0 | 1 | 0 |
| 99 | 0 | 0 | 0 | 0 | 0 | 1 | 1 | 2 | 0 | 0 | 2 | 4 |
| 100 | 2 | 3 | 1 | 3 | 2 | 2 | 2 | 2 | 2 | 1 | 1 | 0 |
| 101 | 2 |  | 1 | 1 |  | 1 | 1 |  | 2 |  | 2 | 1 |
| 102 |  |  |  |  |  |  |  |  |  |  |  |  |
| 103 | 1 | 1 | 0 | 1 | 1 | 1 | 2 | 0 | 2 | 2 | 2 | 0 |
| 104 | 1 | 3 | 2 | 1 | 5 | 2 | 5 | 0 | 3 | 3 | 1 | 0 |
| 105 | 1 | 0 | 3 | 2 | 0 | 0 | 2 | 0 | 2 | 1 | 3 | 0 |

Table 26. Number of newly diagnosed stage II lung cancer during each month in 2020.

|  | Month | | | | | | | | | | | |
| --- | --- | --- | --- | --- | --- | --- | --- | --- | --- | --- | --- | --- |
| Hospital | 1 | 2 | 3 | 4 | 5 | 6 | 7 | 8 | 9 | 10 | 11 | 12 |
| 1 | 0 | 1 | 0 | 0 | 0 | 0 | 1 | 0 | 3 | 0 | 2 | 0 |
| 2 | 5 | 2 | 3 | 1 | 2 | 3 | 2 | 0 | 3 | 2 | 2 | 6 |
| 3 | 1 | 0 | 1 | 0 | 0 | 0 | 1 | 0 | 1 | 0 | 0 | 0 |
| 4 | 3 | 0 | 0 | 0 | 0 | 1 | 5 | 0 | 1 | 5 | 0 | 1 |
| 5 | 0 | 0 | 0 | 1 | 0 | 1 | 2 | 1 | 0 | 1 | 0 | 0 |
| 6 | 1 | 1 | 1 | 2 |  | 2 | 1 | 1 | 1 |  |  |  |
| 7 | 1 | 0 | 0 | 1 | 0 | 2 | 4 | 1 | 0 | 2 | 1 | 1 |
| 8 |  |  |  |  |  |  |  |  |  |  |  |  |
| 9 | 1 | 1 | 2 | 0 | 4 | 0 | 0 | 0 | 1 | 2 | 3 | 1 |
| 10 | 1 | 2 | 0 | 2 | 1 | 0 | 0 | 2 | 2 | 5 | 2 | 0 |
| 11 | 2 | 2 | 5 | 1 | 1 | 2 | 6 | 2 | 2 | 0 | 4 | 2 |
| 12 | 0 | 0 | 0 | 0 | 0 | 0 | 1 | 0 | 0 | 0 | 2 | 0 |
| 13 | 2 | 1 | 2 | 0 | 2 | 0 | 0 | 1 | 2 | 5 | 2 | 3 |
| 14 | 0 | 2 | 2 | 1 | 2 | 0 | 3 | 3 | 2 | 2 | 3 | 4 |
| 15 | 4 | 6 | 3 | 4 | 2 | 1 | 2 | 3 | 2 | 2 | 3 | 1 |
| 16 | 5 | 0 | 3 | 1 | 2 | 1 | 3 | 2 | 4 | 1 | 0 | 1 |
| 17 | 2 | 1 | 0 | 2 | 0 | 0 | 1 | 0 | 3 | 0 | 0 | 1 |
| 18 | 0 | 0 | 1 | 0 | 1 | 0 | 1 | 0 | 0 | 1 | 0 | 1 |
| 19 | 1 | 0 | 0 | 0 | 1 | 1 | 1 | 2 | 0 | 1 | 2 | 1 |
| 20 | 5 | 5 | 5 | 4 |  | 2 | 10 | 7 | 9 | 8 | 8 | 11 |
| 21 | 1 | 0 | 4 | 0 | 1 | 1 | 1 | 0 | 0 | 1 | 0 | 0 |
| 22 |  | 1 |  | 2 | 2 |  | 3 | 1 | 1 | 1 | 2 | 2 |
| 23 |  |  |  |  |  |  |  |  |  |  |  |  |
| 24 | 5 | 5 | 1 | 2 | 2 | 1 | 1 | 1 | 2 | 1 | 2 | 3 |
| 25 | 1 | 2 | 2 | 1 | 1 | 2 | 2 | 1 | 0 | 1 | 1 | 2 |
| 26 | 1 |  | 3 | 3 |  | 6 | 4 |  | 3 | 1 |  | 2 |
| 27 | 3 | 2 | 0 | 0 | 0 | 0 | 0 | 1 | 2 | 0 | 2 | 0 |
| 28 | 1 | 2 | 2 | 1 | 1 | 1 | 3 | 0 | 4 | 4 | 2 | 0 |
| 29 |  |  |  |  |  |  |  |  |  |  |  |  |
| 30 | 1 | 0 | 2 | 1 | 1 | 1 | 2 | 1 | 1 | 3 | 1 | 2 |
| 31 | 0 | 3 | 0 | 4 | 2 | 0 | 0 | 2 | 1 | 0 | 0 | 1 |
| 32 | 1 | 1 |  | 1 | 1 |  |  |  |  |  | 1 | 2 |
| 33 | 0 | 2 | 0 | 0 | 2 | 1 | 1 | 0 | 1 | 1 | 2 | 0 |
| 34 |  |  |  |  |  |  |  |  |  |  |  |  |
| 35 | 2 | 0 | 5 | 1 | 2 | 3 | 2 | 1 | 2 | 1 | 3 | 3 |
| 36 |  | 1 | 2 | 1 | 1 |  | 2 | 1 | 2 | 1 | 1 |  |
| 37 | 1 | 6 | 0 | 4 | 1 | 0 | 4 | 2 | 3 | 4 | 7 | 7 |
| 38 |  |  |  |  |  |  |  |  |  |  |  |  |
| 39 |  |  |  |  |  |  |  |  |  |  |  |  |
| 40 | 1 | 1 | 1 | 3 | 3 |  |  | 1 | 1 | 5 | 1 | 1 |
| 41 | 0 | 0 | 0 | 1 | 2 | 1 | 2 | 0 | 1 | 0 | 1 | 1 |
| 42 | 1 | 0 | 0 | 1 | 0 | 0 | 1 | 4 | 4 | 0 | 1 | 3 |
| 43 | 3 | 1 | 0 | 6 | 0 | 0 | 2 | 4 | 0 | 0 | 1 | 1 |
| 44 | 0 | 2 | 1 | 0 | 2 | 0 | 1 | 0 | 3 | 0 | 1 | 1 |
| 45 | 1 | 1 | 1 | 1 | 1 | 0 | 0 | 2 | 1 | 0 | 2 | 1 |
| 46 | 2 | 0 | 1 | 1 | 0 | 2 | 0 | 1 | 0 | 2 | 1 | 0 |
| 47 |  |  |  |  |  |  |  |  |  |  |  |  |
| 48 | 0 | 1 | 2 | 2 | 3 | 2 | 3 | 3 | 2 | 2 | 3 | 2 |
| 49 | 0 | 0 | 0 | 1 | 1 | 0 | 1 | 0 | 1 | 0 | 0 | 2 |
| 50 | 0 | 0 | 0 | 0 | 1 | 0 | 0 | 0 | 0 | 0 | 0 | 0 |
| 51 | 3 | 1 | 1 | 0 | 3 | 1 | 0 | 1 | 3 | 1 | 3 | 1 |
| 52 | 1 | 1 | 0 | 1 | 1 | 1 | 1 | 1 | 0 | 3 | 1 | 2 |
| 53 | 1 | 1 | 0 | 4 | 1 | 1 | 3 | 2 | 3 | 7 | 4 | 2 |
| 54 | 12 | 4 | 8 | 11 | 5 | 5 | 12 | 5 | 4 | 8 | 6 | 3 |
| 55 | 0 | 1 | 1 | 1 | 1 | 0 | 1 | 2 | 1 | 0 | 1 | 0 |
| 56 | 5 | 2 | 4 | 6 | 4 | 5 | 5 | 2 | 3 | 3 | 7 | 7 |
| 57 | 5 | 4 | 3 | 4 | 1 | 3 | 1 | 1 | 3 | 3 | 6 | 3 |
| 58 | 2 | 3 | 4 | 1 | 3 | 4 | 1 | 2 | 1 | 2 | 3 | 3 |
| 59 | 1 | 2 | 1 | 2 | 0 | 1 | 3 | 4 | 2 | 1 | 0 | 0 |
| 60 | 1 | 3 | 1 | 1 | 2 | 1 | 0 | 2 | 0 | 2 | 0 | 1 |
| 61 | 2 | 2 | 4 | 2 | 1 | 1 | 0 | 1 | 0 | 1 | 0 | 0 |
| 62 |  |  |  |  |  |  |  |  |  |  |  |  |
| 63 |  |  |  |  |  |  |  |  |  |  |  |  |
| 64 | 2 | 3 | 5 | 3 | 2 | 2 | 3 | 4 | 2 | 4 | 3 | 2 |
| 65 | 0 | 3 | 1 | 1 | 0 | 1 | 1 | 4 | 2 | 2 | 2 | 2 |
| 66 | 0 | 0 | 0 | 0 | 0 | 0 | 1 | 1 | 2 | 0 | 1 | 0 |
| 67 | 3 | 1 | 1 | 1 | 1 | 1 | 1 | 1 | 0 | 3 | 1 | 3 |
| 68 | 1 | 2 | 1 | 0 | 0 | 0 | 1 | 1 | 0 | 1 | 1 | 2 |
| 68 | 3 | 2 | 0 | 2 | 1 | 1 | 2 | 2 | 1 | 3 | 3 | 8 |
| 70 |  |  |  |  |  | 1 |  | 1 |  |  |  |  |
| 71 | 0 | 0 | 1 | 0 | 0 | 1 | 0 | 1 | 1 | 2 | 0 | 0 |
| 72 | 5 | 4 | 2 | 0 | 1 | 2 | 0 | 1 | 2 | 4 | 3 | 0 |
| 73 | 1 | 2 | 1 | 1 | 1 | 2 | 1 | 0 | 1 | 2 | 0 | 2 |
| 74 | 4 | 1 | 4 | 6 | 8 | 2 | 3 | 4 | 5 | 2 | 2 | 3 |
| 75 |  |  |  |  |  |  |  |  |  |  |  |  |
| 76 | 2 | 0 | 1 | 3 | 1 | 1 | 2 | 1 | 0 | 2 | 2 | 5 |
| 77 |  |  |  |  |  |  |  |  | 1 |  |  | 1 |
| 78 |  |  |  |  |  |  |  |  |  |  | 1 |  |
| 79 | 3 | 0 | 2 | 1 | 2 | 2 | 1 | 2 | 3 | 5 | 4 | 1 |
| 80 | 2 | 0 | 2 | 0 | 0 | 2 | 2 | 2 | 1 | 3 | 2 | 1 |
| 81 |  | 1 | 2 | 1 | 1 | 3 | 3 | 2 | 2 | 3 | 4 | 1 |
| 82 | 3 | 0 | 2 | 1 | 1 | 2 | 2 | 4 | 1 | 0 | 0 | 1 |
| 83 | 1 | 0 | 1 | 1 | 1 | 2 | 0 | 0 | 1 | 2 | 2 | 0 |
| 84 | 1 | 0 | 2 | 1 | 1 | 2 | 3 | 0 | 0 | 2 | 1 | 1 |
| 85 | 2 | 5 | 1 | 0 | 0 | 1 | 1 | 5 | 4 | 3 | 4 | 3 |
| 86 | 2 | 2 | 0 | 2 | 2 | 2 | 0 | 3 | 3 | 2 | 3 | 1 |
| 87 |  | 2 | 1 |  | 4 |  |  |  |  |  |  |  |
| 88 | 0 | 3 | 0 | 1 | 2 | 0 | 2 | 0 | 0 | 1 | 1 | 1 |
| 89 | 7 | 8 | 8 | 5 | 5 | 7 | 8 | 5 | 5 | 9 | 6 | 7 |
| 90 | 1 | 1 | 0 | 1 | 3 | 1 | 1 | 1 | 3 | 1 | 0 | 0 |
| 91 | 1 | 1 | 0 | 4 | 2 | 2 | 2 | 3 | 0 | 2 | 1 | 0 |
| 92 |  |  |  |  |  |  |  |  |  |  |  |  |
| 93 | 2 | 0 | 0 | 0 | 0 | 0 | 0 | 0 | 0 | 2 | 1 | 1 |
| 94 | 1 | 0 | 0 | 1 | 1 | 0 | 0 | 0 | 0 | 0 | 1 | 0 |
| 95 | 1 | 0 | 1 | 0 | 0 | 0 | 0 | 1 | 0 | 0 | 0 | 1 |
| 96 | 1 | 2 | 0 | 1 | 1 | 1 | 0 | 1 | 1 | 1 | 2 | 1 |
| 97 |  |  |  |  |  |  |  |  |  |  |  |  |
| 98 | 1 | 4 | 2 | 1 | 0 | 2 | 2 | 0 | 1 | 1 | 1 | 0 |
| 99 | 0 | 0 | 0 | 0 | 2 | 0 | 0 | 1 | 1 | 0 | 0 | 0 |
| 100 | 3 | 0 | 2 | 3 | 1 | 3 | 1 | 1 | 3 | 3 | 2 | 2 |
| 101 | 1 |  |  |  |  | 1 |  |  | 1 | 2 |  | 1 |
| 102 |  |  |  |  |  |  |  |  |  |  |  |  |
| 103 | 1 | 3 | 2 | 2 | 1 | 3 | 0 | 1 | 0 | 0 | 2 | 3 |
| 104 | 2 | 3 | 3 | 1 | 2 | 4 | 4 | 3 | 4 | 0 | 1 | 1 |
| 105 | 1 | 0 | 0 | 0 | 0 | 0 | 3 | 1 | 4 | 1 | 1 | 0 |

Table 27. Number of newly diagnosed stage III lung cancer during each month in 2019.

|  | Month | | | | | | | | | | | |
| --- | --- | --- | --- | --- | --- | --- | --- | --- | --- | --- | --- | --- |
| Hospital | 1 | 2 | 3 | 4 | 5 | 6 | 7 | 8 | 9 | 10 | 11 | 12 |
| 1 | 4 | 1 | 2 | 0 | 1 | 2 | 2 | 3 | 2 | 3 | 4 | 7 |
| 2 | 4 | 8 | 3 | 7 | 4 | 7 | 6 | 2 | 8 | 9 | 6 | 6 |
| 3 | 1 | 1 | 1 | 0 | 0 | 0 | 3 | 2 | 2 | 0 | 0 | 1 |
| 4 | 2 | 3 | 2 | 4 | 7 | 3 | 6 | 2 | 1 | 7 | 0 | 3 |
| 5 | 4 | 1 | 3 | 1 | 0 | 3 | 0 | 1 | 2 | 2 | 2 | 1 |
| 6 | 2 | 1 | 4 | 3 | 3 | 4 | 2 | 2 | 3 | 7 | 3 |  |
| 7 | 2 | 4 | 4 | 3 | 1 | 1 | 2 | 2 | 3 | 3 | 5 | 3 |
| 8 |  |  |  |  |  |  |  |  |  |  |  |  |
| 9 | 3 | 1 | 3 | 3 | 4 | 2 | 2 | 6 | 5 | 2 | 2 | 4 |
| 10 | 1 | 1 | 4 | 4 | 1 | 1 | 3 | 1 | 3 | 1 | 0 | 1 |
| 11 | 8 | 9 | 7 | 3 | 10 | 10 | 7 | 2 | 3 | 4 | 2 | 5 |
| 12 | 0 | 0 | 0 | 0 | 0 | 1 | 1 | 2 | 0 | 0 | 0 | 0 |
| 13 | 3 | 3 | 0 | 6 | 4 | 1 | 6 | 2 | 2 | 2 | 3 | 0 |
| 14 | 7 | 3 | 3 | 1 | 1 | 3 | 3 | 3 | 6 | 2 | 4 | 3 |
| 15 | 3 | 4 | 5 | 7 | 2 | 6 | 2 | 1 | 2 | 5 | 14 | 5 |
| 16 | 4 | 1 | 5 | 4 | 6 | 4 | 4 | 6 | 5 | 5 | 7 | 1 |
| 17 |  | 1 |  |  |  | 4 | 1 | 4 | 5 | 1 | 2 | 2 |
| 18 | 1 | 3 | 6 | 3 | 4 | 5 | 4 | 2 | 3 | 3 | 6 | 1 |
| 19 | 0 | 0 | 3 | 3 | 3 | 1 | 0 | 1 | 1 | 2 | 0 | 1 |
| 20 | 15 | 14 | 16 | 16 | 13 | 12 | 15 | 17 | 14 | 18 | 19 | 17 |
| 21 | 0 | 3 | 2 | 4 | 7 | 2 | 0 | 1 | 2 | 1 | 4 | 6 |
| 22 | 1 | 6 | 3 | 4 | 1 | 3 | 1 |  | 3 | 4 | 6 | 2 |
| 23 |  |  |  |  |  |  |  |  |  |  |  |  |
| 24 | 8 | 3 | 4 | 8 | 4 | 3 | 10 | 4 | 5 | 3 | 6 | 9 |
| 25 | 2 | 1 | 0 | 5 | 4 | 3 | 3 | 3 | 0 | 2 | 3 | 3 |
| 26 | 5 | 1 | 4 | 1 | 4 | 3 | 6 | 3 | 6 | 6 | 5 | 6 |
| 27 | 2 | 1 | 2 | 4 | 0 | 3 | 3 | 1 | 2 | 1 | 2 | 2 |
| 28 | 5 | 3 | 3 | 0 | 2 | 5 | 5 | 3 | 2 | 3 | 2 | 3 |
| 29 |  |  |  |  |  |  |  |  |  |  |  |  |
| 30 | 0 | 2 | 5 | 1 | 3 | 3 | 3 | 1 | 1 | 1 | 5 | 0 |
| 31 | 3 | 1 | 1 | 1 | 3 | 2 | 2 | 1 | 2 | 2 | 3 | 0 |
| 32 | 4 | 4 | 3 | 2 | 2 | 2 | 2 | 4 | 3 | 1 | 2 | 1 |
| 33 | 2 | 5 | 1 | 4 | 2 | 2 | 4 | 3 | 1 | 2 | 2 | 0 |
| 34 |  |  |  |  |  |  |  |  |  |  |  |  |
| 35 | 2 | 4 | 3 | 5 | 5 | 4 | 3 | 4 | 4 | 4 | 3 | 1 |
| 36 |  |  |  | 1 |  |  | 2 | 3 |  |  |  |  |
| 37 | 4 | 4 | 6 | 6 | 1 | 3 | 3 | 7 | 1 | 2 | 3 | 12 |
| 38 |  |  |  |  |  |  |  |  |  |  |  |  |
| 39 |  | 1 |  | 1 |  |  |  |  |  |  |  |  |
| 40 | 5 | 2 | 5 | 4 | 5 | 3 | 8 | 4 | 5 | 3 | 2 | 5 |
| 41 | 1 | 1 | 1 | 2 | 0 | 1 | 1 | 1 | 0 | 2 | 0 | 2 |
| 42 | 3 | 2 | 3 | 1 | 2 | 3 | 2 | 2 | 2 | 2 | 1 | 1 |
| 43 | 4 | 3 | 4 | 0 | 3 | 5 | 7 | 5 | 2 | 2 | 5 | 2 |
| 44 | 0 | 2 | 4 | 0 | 1 | 2 | 2 | 3 | 1 | 2 | 1 | 1 |
| 45 | 2 | 1 | 1 | 3 | 2 | 1 | 2 | 4 | 4 | 3 | 2 | 2 |
| 46 | 1 | 1 | 0 | 2 | 4 | 1 | 4 | 0 | 2 | 1 | 5 | 2 |
| 47 |  |  |  |  |  |  |  |  |  |  |  |  |
| 48 | 0 | 6 | 6 | 2 | 4 | 3 | 10 | 2 | 7 | 5 | 6 | 3 |
| 49 | 1 | 2 | 0 | 4 | 1 | 2 | 3 | 1 | 2 | 2 | 2 | 3 |
| 50 | 0 | 0 | 0 | 0 | 1 | 0 | 0 | 0 | 0 | 0 | 0 | 0 |
| 51 | 5 | 1 | 4 | 3 | 5 | 3 | 3 | 3 | 7 | 2 | 2 | 2 |
| 52 | 3 | 6 | 1 | 4 | 1 | 2 | 3 | 6 | 2 | 0 | 1 | 0 |
| 53 | 4 | 2 | 3 | 4 | 6 | 9 | 8 | 7 | 7 | 5 | 7 | 6 |
| 54 | 11 | 7 | 12 | 12 | 10 | 11 | 15 | 17 | 11 | 18 | 15 | 12 |
| 55 | 2 | 0 | 3 | 1 | 3 | 0 | 5 | 3 | 7 | 2 | 0 | 5 |
| 56 | 2 | 8 | 3 | 5 | 9 | 4 | 2 | 5 | 7 | 5 | 7 | 5 |
| 57 | 10 | 8 | 7 | 5 | 8 | 3 | 6 | 2 | 10 | 12 | 6 | 10 |
| 58 | 2 | 2 | 1 | 4 | 3 | 1 | 3 | 1 | 3 | 3 | 3 | 4 |
| 59 | 3 | 2 | 3 | 3 | 3 | 5 | 4 | 0 | 3 | 1 | 2 | 3 |
| 60 | 4 | 4 | 5 | 1 | 4 | 8 | 2 | 3 | 3 | 3 | 6 | 3 |
| 61 | 3 | 3 | 4 | 5 | 2 | 3 | 4 | 2 | 3 | 2 | 5 | 5 |
| 62 |  |  |  |  |  |  |  |  |  |  |  |  |
| 63 |  |  |  |  |  |  |  |  |  |  |  |  |
| 64 | 5 | 4 | 10 | 6 | 6 | 6 | 9 | 12 | 4 | 7 | 2 | 3 |
| 65 | 3 | 4 | 2 | 6 | 4 | 4 | 2 | 3 | 0 | 5 | 6 | 1 |
| 66 | 0 | 0 | 0 | 0 | 1 | 0 | 1 | 1 | 1 | 1 | 1 | 1 |
| 67 | 8 | 3 | 5 | 8 | 4 | 3 | 4 | 7 | 3 | 2 | 3 | 5 |
| 68 | 1 | 3 | 1 | 1 | 5 | 1 | 3 | 3 | 2 | 4 | 1 | 1 |
| 68 | 11 | 10 | 11 | 9 | 7 | 9 | 3 | 7 | 9 | 7 | 8 | 11 |
| 70 |  |  |  | 2 |  | 1 | 1 |  |  |  |  |  |
| 71 | 2 | 1 | 3 | 5 | 3 | 1 | 1 | 2 | 1 | 2 | 2 | 4 |
| 72 | 10 | 4 | 3 | 6 | 1 | 6 | 6 | 5 | 5 | 5 | 2 | 5 |
| 73 | 6 | 4 | 5 | 1 | 4 | 2 | 1 | 5 | 2 | 2 | 5 | 1 |
| 74 | 1 | 4 | 6 | 8 | 7 | 5 | 5 | 3 | 10 | 4 | 3 | 11 |
| 75 |  |  |  |  |  |  |  |  |  |  |  |  |
| 76 | 4 | 2 | 2 | 6 | 1 | 7 | 2 | 4 | 6 | 2 | 4 | 2 |
| 77 |  |  |  |  | 1 | 1 |  |  |  | 1 |  | 2 |
| 78 | 1 |  |  |  |  |  |  | 4 | 1 |  |  |  |
| 79 | 3 | 2 | 5 | 6 | 2 | 6 | 5 | 3 | 3 | 4 | 6 | 5 |
| 80 | 2 | 0 | 4 | 5 | 6 | 5 | 6 | 2 | 3 | 2 | 1 | 4 |
| 81 | 2 | 2 | 1 | 5 | 2 | 5 | 5 | 4 | 3 | 5 | 4 | 4 |
| 82 | 4 | 3 | 1 | 3 | 1 | 3 | 1 | 4 | 0 | 0 | 2 | 0 |
| 83 | 3 | 0 | 2 | 0 | 1 | 2 | 0 | 1 | 0 | 5 | 2 | 1 |
| 84 | 1 | 2 | 2 | 1 | 1 | 2 | 2 | 2 | 3 | 2 | 2 | 3 |
| 85 | 1 | 4 | 2 | 2 | 4 | 3 | 4 | 6 | 1 | 3 | 4 | 6 |
| 86 | 4 | 4 | 0 | 1 | 3 | 4 | 1 | 4 | 1 | 2 | 5 | 5 |
| 87 | 1 | 4 | 1 | 2 |  | 1 | 1 | 1 | 1 |  | 3 | 2 |
| 88 | 3 | 3 | 3 | 4 | 3 | 1 | 1 | 3 | 4 | 0 | 1 | 3 |
| 89 | 17 | 7 | 22 | 14 | 13 | 16 | 15 | 15 | 12 | 21 | 10 | 13 |
| 90 | 3 | 5 | 2 | 3 | 1 | 5 | 6 | 2 | 2 | 3 | 0 | 0 |
| 91 | 5 | 2 | 7 | 3 | 3 | 5 | 5 | 4 | 3 | 3 | 4 | 8 |
| 92 |  |  |  |  |  |  |  |  |  |  |  |  |
| 93 | 3 | 2 | 0 | 4 | 1 | 2 | 1 | 1 | 1 | 3 | 0 | 3 |
| 94 | 0 | 1 | 0 | 6 | 2 | 1 | 2 | 0 | 2 | 1 | 3 | 1 |
| 95 | 2 | 1 | 0 | 0 | 1 | 0 | 3 | 0 | 0 | 0 | 1 | 1 |
| 96 | 3 | 2 | 2 | 3 | 0 | 1 | 0 | 1 | 1 | 0 | 0 | 1 |
| 97 |  |  |  |  |  |  |  |  |  |  |  |  |
| 98 | 3 | 1 | 3 | 5 | 5 | 5 | 5 | 2 | 3 | 2 | 8 | 3 |
| 99 | 5 | 1 | 3 | 2 | 0 | 3 | 0 | 1 | 2 | 0 | 1 | 1 |
| 100 | 4 | 6 | 3 | 1 | 1 | 2 | 2 | 1 | 2 | 2 | 4 | 3 |
| 101 | 1 | 2 | 1 | 3 | 3 | 4 | 2 | 5 | 1 | 1 | 4 | 4 |
| 102 |  |  |  |  |  |  |  |  |  |  |  |  |
| 103 | 1 | 0 | 1 | 3 | 5 | 3 | 3 | 5 | 0 | 2 | 2 | 3 |
| 104 | 6 | 5 | 1 | 3 | 4 | 2 | 7 | 4 | 8 | 1 | 5 | 5 |
| 105 | 4 | 2 | 1 | 4 | 2 | 2 | 1 | 5 | 1 | 4 | 3 | 1 |

Table 28. Number of newly diagnosed stage III lung cancer during each month in 2020.

|  | Month | | | | | | | | | | | |
| --- | --- | --- | --- | --- | --- | --- | --- | --- | --- | --- | --- | --- |
| Hospital | 1 | 2 | 3 | 4 | 5 | 6 | 7 | 8 | 9 | 10 | 11 | 12 |
| 1 | 3 | 2 | 1 | 1 | 6 | 3 | 1 | 0 | 3 | 2 | 1 | 2 |
| 2 | 4 | 5 | 8 | 6 | 3 | 5 | 4 | 1 | 10 | 5 | 5 | 2 |
| 3 | 1 | 1 | 1 | 4 | 2 | 1 | 0 | 1 | 1 | 3 | 1 | 0 |
| 4 | 3 | 7 | 4 | 7 | 6 | 1 | 2 | 4 | 5 | 1 | 4 | 4 |
| 5 | 2 | 1 | 5 | 2 | 1 | 0 | 1 | 1 | 0 | 0 | 0 | 0 |
| 6 | 5 | 4 | 2 | 2 | 1 | 1 | 2 | 1 | 2 | 5 | 4 | 5 |
| 7 | 0 | 4 | 1 | 1 | 0 | 5 | 3 | 1 | 1 | 1 | 1 | 2 |
| 8 |  |  |  |  |  |  |  |  |  |  |  |  |
| 9 | 5 | 2 | 2 | 2 | 0 | 0 | 2 | 3 | 4 | 3 | 3 | 0 |
| 10 | 1 | 1 | 1 | 0 | 0 | 1 | 3 | 2 | 1 | 4 | 0 | 0 |
| 11 | 5 | 4 | 3 | 5 | 2 | 3 | 5 | 6 | 1 | 1 | 4 | 3 |
| 12 | 0 | 1 | 2 | 0 | 1 | 0 | 0 | 2 | 0 | 1 | 1 | 0 |
| 13 | 3 | 4 | 4 | 0 | 2 | 9 | 3 | 2 | 4 | 4 | 2 | 3 |
| 14 | 2 | 2 | 2 | 1 | 0 | 6 | 2 | 3 | 4 | 2 | 2 | 2 |
| 15 | 4 | 2 | 2 | 8 | 6 | 8 | 6 | 1 | 6 | 3 | 7 | 5 |
| 16 | 5 | 1 | 3 | 2 | 3 | 2 | 5 | 3 | 6 | 5 | 7 | 2 |
| 17 | 3 | 2 | 2 | 2 | 1 | 0 | 3 | 0 | 0 | 1 | 1 | 2 |
| 18 | 3 | 4 | 1 | 6 | 7 | 6 | 2 | 5 | 1 | 8 | 3 | 5 |
| 19 | 1 | 2 | 1 | 0 | 0 | 1 | 5 | 0 | 0 | 2 | 2 | 1 |
| 20 | 17 | 14 | 21 | 7 | 14 | 18 | 20 | 10 | 16 | 21 | 11 | 15 |
| 21 | 5 | 2 | 1 | 1 | 2 | 2 | 1 | 0 | 1 | 5 | 0 | 0 |
| 22 | 3 | 3 | 6 | 2 | 3 | 4 | 3 | 1 |  | 6 | 2 | 3 |
| 23 |  |  |  |  |  |  |  |  |  |  |  |  |
| 24 | 4 | 4 | 6 | 2 | 5 | 4 | 7 | 3 | 3 | 3 | 2 | 2 |
| 25 | 5 | 0 | 0 | 2 | 2 | 4 | 1 | 2 | 8 | 3 | 5 | 0 |
| 26 | 8 | 3 | 7 | 3 | 3 | 6 | 4 | 6 | 4 | 3 | 4 | 4 |
| 27 | 1 | 1 | 1 | 2 | 2 | 4 | 0 | 1 | 3 | 1 | 2 | 5 |
| 28 | 1 | 2 | 3 | 3 | 3 | 6 | 2 | 4 | 4 | 5 | 1 | 1 |
| 29 |  |  |  |  |  |  |  |  |  |  |  |  |
| 30 | 5 | 4 | 3 | 2 | 2 | 1 | 2 | 2 | 4 | 6 | 4 | 7 |
| 31 | 1 | 1 | 3 | 1 |  | 2 | 4 | 1 | 0 | 4 | 2 | 1 |
| 32 | 1 | 3 | 1 | 4 | 2 |  | 3 | 2 | 3 | 2 |  | 1 |
| 33 | 0 | 3 | 2 | 3 | 2 | 4 | 2 | 0 | 1 | 3 | 2 | 0 |
| 34 |  |  |  |  |  |  |  |  |  |  |  |  |
| 35 | 1 | 1 | 2 | 1 | 2 | 5 | 7 | 4 | 3 | 3 | 3 | 4 |
| 36 | 3 |  |  |  |  |  | 2 |  | 1 | 1 | 1 |  |
| 37 | 4 | 4 | 4 | 6 | 7 | 6 | 7 | 4 | 5 | 5 | 7 | 6 |
| 38 |  |  |  |  |  |  |  |  |  |  |  |  |
| 39 |  |  |  | 1 | 1 |  |  |  | 1 | 1 |  |  |
| 40 | 4 | 5 | 2 | 3 | 3 | 3 | 3 | 5 | 1 | 1 | 2 | 1 |
| 41 | 0 | 2 | 2 | 0 | 1 | 1 | 3 | 1 | 1 | 0 | 0 | 1 |
| 42 | 0 | 1 | 3 | 1 | 0 | 2 | 3 | 6 | 1 | 3 | 2 | 8 |
| 43 | 1 | 1 | 2 | 3 | 1 | 3 | 1 | 2 | 1 | 3 | 4 | 4 |
| 44 | 1 | 0 | 3 | 0 | 2 | 4 | 1 | 2 | 3 | 3 | 1 | 2 |
| 45 | 2 | 5 | 2 | 5 | 2 | 3 | 3 | 0 | 0 | 3 | 0 | 2 |
| 46 | 2 | 2 | 2 | 3 | 4 | 2 | 1 | 3 | 2 | 1 | 3 | 3 |
| 47 |  |  |  |  |  |  |  |  |  |  |  |  |
| 48 | 2 | 2 | 1 | 4 | 1 | 3 | 2 | 0 | 2 | 3 | 2 | 3 |
| 49 | 1 | 3 | 5 | 2 | 2 | 3 | 1 | 0 | 2 | 3 | 2 | 3 |
| 50 | 2 | 0 | 0 | 0 | 0 | 0 | 0 | 0 | 0 | 0 | 1 | 0 |
| 51 | 2 | 2 | 3 | 3 | 4 | 3 | 5 | 4 | 2 | 4 | 4 | 2 |
| 52 | 5 | 7 | 3 | 7 | 0 | 4 | 3 | 1 | 3 | 6 | 5 | 2 |
| 53 | 8 | 5 | 6 | 10 | 6 | 5 | 6 | 9 | 6 | 12 | 4 | 7 |
| 54 | 5 | 7 | 8 | 5 | 6 | 16 | 16 | 7 | 10 | 19 | 13 | 15 |
| 55 | 1 | 2 | 5 | 2 | 2 | 2 | 3 | 4 | 1 | 1 | 5 | 0 |
| 56 | 6 | 8 | 3 | 4 | 4 | 5 | 6 | 7 | 6 | 9 | 8 | 8 |
| 57 | 12 | 7 | 4 | 5 | 3 | 4 | 2 | 6 | 6 | 6 | 3 | 7 |
| 58 | 1 | 4 | 4 | 3 | 3 | 1 | 2 | 2 | 0 | 2 | 0 | 6 |
| 59 | 2 | 2 | 2 | 7 | 3 | 4 | 2 | 2 | 3 | 3 | 0 | 1 |
| 60 | 6 | 5 | 6 | 1 | 2 | 5 | 5 | 6 | 3 | 1 | 4 | 5 |
| 61 | 5 | 4 | 1 | 1 | 5 | 10 | 5 | 5 | 4 | 3 | 1 | 3 |
| 62 |  |  |  |  |  |  |  |  |  |  |  |  |
| 63 |  |  |  |  |  |  |  |  |  |  |  |  |
| 64 | 4 | 6 | 9 | 5 | 3 | 8 | 4 | 7 | 8 | 10 | 6 | 5 |
| 65 | 0 | 1 | 2 | 2 | 2 | 2 | 2 | 3 | 4 | 2 | 7 | 5 |
| 66 | 0 | 2 | 1 | 0 | 0 | 0 | 1 | 1 | 1 | 0 | 4 | 0 |
| 67 | 1 | 0 | 2 | 2 | 1 | 6 | 2 | 2 | 3 | 7 | 2 | 4 |
| 68 | 2 | 2 | 0 | 1 | 0 | 2 | 2 | 3 | 0 | 0 | 0 | 6 |
| 68 | 9 | 14 | 14 | 12 | 8 | 6 | 8 | 11 | 11 | 7 | 16 | 11 |
| 70 |  |  |  | 1 |  |  |  |  |  |  |  |  |
| 71 | 2 | 0 | 3 | 2 | 3 | 4 | 2 | 8 | 0 | 1 | 1 | 0 |
| 72 | 7 | 6 | 1 | 4 | 3 | 4 | 5 | 6 | 1 | 5 | 4 | 3 |
| 73 | 8 | 1 | 3 | 2 | 1 | 2 | 2 | 1 | 0 | 4 | 2 | 2 |
| 74 | 4 | 6 | 7 | 7 | 7 | 9 | 11 | 4 | 16 | 6 | 8 | 5 |
| 75 |  |  |  |  |  |  |  |  |  |  |  |  |
| 76 | 3 | 3 | 3 | 8 | 3 | 5 | 1 | 2 | 6 | 1 | 4 | 2 |
| 77 | 1 |  |  |  | 1 |  |  |  | 1 |  | 1 | 2 |
| 78 |  |  |  |  | 1 |  |  | 1 |  | 2 |  |  |
| 79 | 5 | 4 | 7 | 1 | 1 | 5 | 4 | 4 | 3 | 5 | 3 | 3 |
| 80 | 4 | 3 | 1 | 4 | 0 | 4 | 5 | 2 | 3 | 4 | 2 | 1 |
| 81 | 5 | 2 | 2 |  | 3 | 4 | 6 | 3 | 5 | 8 | 1 | 4 |
| 82 | 0 | 3 | 4 | 0 | 3 | 6 | 3 | 3 | 2 | 1 | 0 | 2 |
| 83 | 0 | 2 | 1 | 0 | 0 | 0 | 0 | 3 | 3 | 0 | 3 | 4 |
| 84 | 5 | 0 | 2 | 5 | 0 | 1 | 3 | 0 | 3 | 4 | 1 | 4 |
| 85 | 8 | 4 | 6 | 4 | 3 | 3 | 4 | 2 | 8 | 6 | 0 | 7 |
| 86 | 1 | 0 | 2 | 6 | 2 | 5 | 2 | 2 | 1 | 3 | 5 | 4 |
| 87 | 2 | 2 | 2 | 2 | 2 | 3 | 3 | 3 |  | 1 |  | 2 |
| 88 | 3 | 0 | 1 | 2 | 2 | 1 | 3 | 0 | 4 | 2 | 0 | 1 |
| 89 | 18 | 10 | 15 | 18 | 9 | 15 | 18 | 20 | 15 | 15 | 25 | 14 |
| 90 | 0 | 6 | 3 | 3 | 5 | 2 | 6 | 1 | 0 | 3 | 2 | 2 |
| 91 | 8 | 3 | 3 | 3 | 2 | 3 | 3 | 3 | 3 | 3 | 3 | 5 |
| 92 |  |  |  |  |  |  |  |  |  |  |  |  |
| 93 | 1 | 1 | 2 | 1 | 1 | 0 | 3 | 0 | 1 | 1 | 0 | 0 |
| 94 | 0 | 2 | 0 | 1 | 2 | 0 | 0 | 1 | 1 | 0 | 1 | 1 |
| 95 | 2 | 0 | 0 | 1 | 1 | 0 | 1 | 0 | 1 | 1 | 0 | 0 |
| 96 | 1 | 4 | 4 | 5 | 0 | 1 | 2 | 1 | 2 | 0 | 4 | 2 |
| 97 |  |  |  |  |  |  |  |  |  |  |  |  |
| 98 | 1 | 3 | 1 | 2 | 3 | 1 | 3 | 1 | 2 | 1 | 4 | 4 |
| 99 | 1 | 1 | 1 | 0 | 0 | 0 | 3 | 0 | 1 | 2 | 1 | 1 |
| 100 | 3 | 4 | 6 | 12 | 7 | 6 | 2 | 3 | 1 | 2 | 2 | 4 |
| 101 | 1 |  | 1 | 4 | 1 | 1 | 7 | 2 | 1 | 4 | 5 | 4 |
| 102 |  |  |  |  |  |  |  |  |  |  |  |  |
| 103 | 0 | 0 | 4 | 1 | 2 | 1 | 5 | 4 | 3 | 0 | 5 | 3 |
| 104 | 0 | 3 | 6 | 3 | 2 | 3 | 6 | 3 | 5 | 9 | 4 | 6 |
| 105 | 2 | 0 | 2 | 5 | 4 | 2 | 1 | 1 | 5 | 6 | 2 | 4 |

Table 29. Number of newly diagnosed stage IV lung cancer during each month in 2019.

|  | Month | | | | | | | | | | | |
| --- | --- | --- | --- | --- | --- | --- | --- | --- | --- | --- | --- | --- |
| Hospital | 1 | 2 | 3 | 4 | 5 | 6 | 7 | 8 | 9 | 10 | 11 | 12 |
| 1 | 2 | 9 | 2 | 2 | 2 | 4 | 4 | 7 | 2 | 5 | 3 | 5 |
| 2 | 14 | 12 | 10 | 8 | 12 | 8 | 15 | 12 | 10 | 13 | 4 | 6 |
| 3 | 0 | 1 | 1 | 3 | 4 | 1 | 0 | 1 | 2 | 1 | 2 | 4 |
| 4 | 6 | 9 | 15 | 9 | 6 | 6 | 6 | 10 | 12 | 10 | 11 | 9 |
| 5 | 3 | 3 | 5 | 2 | 0 | 4 | 3 | 5 | 2 | 5 | 1 | 3 |
| 6 | 3 | 3 | 7 | 3 | 1 | 7 | 6 | 2 | 5 | 5 | 1 | 7 |
| 7 | 4 | 4 | 3 | 5 | 4 | 0 | 3 | 6 | 6 | 8 | 7 | 0 |
| 8 |  |  |  |  |  |  |  |  |  |  |  |  |
| 9 | 4 | 2 | 5 | 8 | 8 | 2 | 5 | 5 | 6 | 2 | 2 | 5 |
| 10 | 7 | 3 | 4 | 2 | 1 | 7 | 10 | 3 | 2 | 2 | 4 | 5 |
| 11 | 11 | 7 | 9 | 13 | 9 | 6 | 13 | 11 | 5 | 7 | 6 | 8 |
| 12 | 2 | 1 | 0 | 2 | 0 | 0 | 2 | 1 | 3 | 0 | 1 | 4 |
| 13 | 3 | 8 | 5 | 4 | 5 | 9 | 7 | 7 | 5 | 9 | 10 | 7 |
| 14 | 4 | 10 | 8 | 5 | 6 | 6 | 3 | 3 | 8 | 2 | 6 | 5 |
| 15 | 12 | 23 | 8 | 11 | 13 | 10 | 12 | 10 | 13 | 17 | 8 | 6 |
| 16 | 7 | 14 | 9 | 16 | 10 | 11 | 18 | 12 | 14 | 11 | 9 | 5 |
| 17 | 4 | 4 | 2 | 6 | 3 | 2 | 1 | 7 | 5 | 1 | 4 | 2 |
| 18 | 9 | 2 | 6 | 10 | 11 | 9 | 5 | 1 | 7 | 6 | 12 | 7 |
| 19 | 1 | 3 | 5 | 2 | 3 | 7 | 4 | 5 | 2 | 1 | 3 | 9 |
| 20 | 21 | 19 | 19 | 25 | 24 | 26 | 22 | 13 | 27 | 28 | 23 | 26 |
| 21 | 7 | 5 | 10 | 9 | 7 | 5 | 5 | 9 | 10 | 7 | 4 | 7 |
| 22 | 4 | 4 | 9 | 6 | 9 | 10 | 8 | 4 | 7 | 4 | 3 | 4 |
| 23 |  |  |  |  |  |  |  |  |  |  |  |  |
| 24 | 15 | 6 | 9 | 15 | 10 | 8 | 6 | 7 | 15 | 11 | 12 | 11 |
| 25 | 10 | 6 | 6 | 10 | 7 | 7 | 3 | 5 | 4 | 5 | 4 | 3 |
| 26 | 8 | 5 | 9 | 11 | 7 | 6 | 5 | 15 | 7 | 8 | 6 | 11 |
| 27 | 4 | 3 | 3 | 7 | 6 | 2 | 8 | 8 | 2 | 7 | 2 | 4 |
| 28 | 3 | 5 | 4 | 5 | 4 | 3 | 4 | 4 | 5 | 6 | 3 | 2 |
| 29 |  |  |  |  |  |  |  |  |  |  |  |  |
| 30 | 4 | 3 | 12 | 2 | 4 | 5 | 7 | 4 | 7 | 8 | 5 | 2 |
| 31 | 4 | 3 | 2 | 2 | 3 | 3 | 2 | 4 | 6 | 3 | 4 | 3 |
| 32 | 7 | 4 | 10 | 1 | 2 | 8 | 5 | 5 | 3 | 6 | 6 | 7 |
| 33 | 6 | 1 | 9 | 7 | 3 | 7 | 8 | 7 | 9 | 4 | 5 | 2 |
| 34 |  |  |  |  |  |  |  |  |  |  |  |  |
| 35 | 8 | 7 | 5 | 7 | 12 | 8 | 7 | 10 | 4 | 4 | 5 | 5 |
| 36 | 1 | 1 | 1 | 2 |  |  |  |  | 1 |  | 2 | 2 |
| 37 | 6 | 10 | 17 | 8 | 12 | 9 | 12 | 7 | 12 | 11 | 12 | 6 |
| 38 |  |  |  |  |  |  |  |  |  |  |  |  |
| 39 |  |  |  | 1 |  | 2 | 1 | 2 |  |  | 1 | 1 |
| 40 | 4 | 4 | 5 | 4 | 4 | 1 | 11 | 2 | 6 | 4 | 6 | 10 |
| 41 | 3 | 3 | 2 | 0 | 3 | 4 | 4 | 4 | 2 | 6 | 3 | 1 |
| 42 | 3 | 2 | 3 | 6 | 6 | 8 | 4 | 9 | 11 | 6 | 7 | 6 |
| 43 | 4 | 2 | 3 | 5 | 11 | 4 | 5 | 2 | 6 | 8 | 8 | 5 |
| 44 | 3 | 5 | 2 | 6 | 10 | 0 | 4 | 3 | 3 | 2 | 2 | 3 |
| 45 | 5 | 6 | 13 | 9 | 5 | 6 | 11 | 4 | 8 | 6 | 3 | 8 |
| 46 | 3 | 8 | 4 | 6 | 2 | 4 | 5 | 2 | 4 | 6 | 1 | 5 |
| 47 |  |  |  |  |  |  |  |  |  |  |  |  |
| 48 | 4 | 11 | 5 | 6 | 10 | 10 | 11 | 7 | 8 | 5 | 7 | 11 |
| 49 | 9 | 1 | 5 | 2 | 4 | 8 | 6 | 6 | 2 | 3 | 6 | 5 |
| 50 | 0 | 0 | 1 | 0 | 0 | 0 | 1 | 0 | 0 | 0 | 0 | 1 |
| 51 | 3 | 5 | 7 | 1 | 6 | 8 | 8 | 6 | 3 | 5 | 5 | 7 |
| 52 | 4 | 4 | 2 | 5 | 4 | 6 | 5 | 3 | 2 | 4 | 5 | 3 |
| 53 | 4 | 9 | 8 | 8 | 19 | 14 | 11 | 7 | 6 | 9 | 8 | 12 |
| 54 | 21 | 20 | 24 | 20 | 38 | 29 | 35 | 24 | 20 | 31 | 25 | 25 |
| 55 | 5 | 5 | 2 | 4 | 1 | 2 | 3 | 1 | 3 | 7 | 5 | 3 |
| 56 | 9 | 7 | 8 | 10 | 7 | 10 | 5 | 18 | 8 | 10 | 8 | 10 |
| 57 | 12 | 19 | 15 | 14 | 20 | 9 | 12 | 21 | 11 | 20 | 11 | 16 |
| 58 | 3 | 2 | 12 | 7 | 4 | 10 | 8 | 5 | 5 | 6 | 5 | 2 |
| 59 | 6 | 3 | 3 | 3 | 3 | 3 | 6 | 6 | 4 | 4 | 6 | 4 |
| 60 | 8 | 4 | 5 | 5 | 6 | 7 | 6 | 8 | 17 | 8 | 8 | 10 |
| 61 | 6 | 4 | 9 | 8 | 6 | 12 | 6 | 7 | 11 | 5 | 12 | 5 |
| 62 |  |  |  |  |  |  |  |  |  |  |  |  |
| 63 |  |  |  |  |  |  |  |  |  |  |  |  |
| 64 | 11 | 9 | 9 | 12 | 7 | 5 | 15 | 17 | 5 | 7 | 11 | 10 |
| 65 | 9 | 7 | 4 | 7 | 6 | 5 | 6 | 4 | 8 | 10 | 2 | 1 |
| 66 | 4 | 1 | 0 | 2 | 4 | 1 | 6 | 2 | 3 | 2 | 1 | 3 |
| 67 | 9 | 14 | 4 | 12 | 5 | 8 | 10 | 7 | 5 | 10 | 13 | 6 |
| 68 | 6 | 5 | 3 | 3 | 4 | 5 | 5 | 2 | 10 | 5 | 2 | 2 |
| 68 | 10 | 17 | 15 | 11 | 19 | 12 | 14 | 10 | 11 | 12 | 20 | 23 |
| 70 |  | 2 |  | 1 | 1 |  | 1 |  | 1 |  | 2 |  |
| 71 | 4 | 3 | 7 | 8 | 7 | 9 | 4 | 5 | 7 | 5 | 4 | 3 |
| 72 | 6 | 8 | 10 | 13 | 7 | 10 | 11 | 13 | 4 | 4 | 7 | 10 |
| 73 | 5 | 3 | 10 | 4 | 5 | 4 | 7 | 5 | 7 | 4 | 10 | 8 |
| 74 | 10 | 14 | 8 | 12 | 12 | 9 | 12 | 10 | 16 | 11 | 8 | 6 |
| 75 |  |  |  |  |  |  |  |  |  |  |  |  |
| 76 | 7 | 4 | 3 | 10 | 8 | 10 | 7 | 7 | 12 | 6 | 8 | 7 |
| 77 | 2 |  |  | 3 | 2 | 2 | 3 | 2 | 1 |  | 2 | 1 |
| 78 | 1 |  | 4 |  |  | 2 | 4 | 2 | 1 |  | 2 |  |
| 79 | 8 | 5 | 2 | 3 | 11 | 3 | 2 | 7 | 3 | 6 | 3 | 6 |
| 80 | 5 | 5 | 8 | 8 | 9 | 3 | 9 | 9 | 10 | 7 | 2 | 7 |
| 81 | 3 | 3 | 10 | 7 | 3 | 7 | 11 | 3 | 8 | 9 | 5 | 6 |
| 82 | 0 | 2 | 1 | 2 | 1 | 2 | 1 | 1 | 2 | 3 | 3 | 0 |
| 83 | 4 | 4 | 0 | 1 | 6 | 4 | 2 | 2 | 6 | 5 | 5 | 3 |
| 84 | 3 | 1 | 8 | 9 | 5 | 10 | 8 | 7 | 7 | 4 | 1 | 5 |
| 85 | 11 | 6 | 9 | 10 | 14 | 9 | 14 | 13 | 10 | 10 | 7 | 7 |
| 86 | 5 | 6 | 5 | 3 | 5 | 7 | 5 | 1 | 3 | 4 | 4 | 3 |
| 87 | 7 | 3 | 8 | 10 | 9 | 4 | 7 | 4 | 5 | 4 | 5 | 5 |
| 88 | 7 | 1 | 5 | 4 | 3 | 5 | 4 | 2 | 4 | 4 | 2 | 2 |
| 89 | 20 | 13 | 17 | 31 | 13 | 15 | 33 | 28 | 16 | 22 | 27 | 22 |
| 90 | 8 | 7 | 6 | 2 | 11 | 6 | 8 | 7 | 5 | 7 | 6 | 4 |
| 91 | 8 | 15 | 9 | 9 | 11 | 11 | 10 | 9 | 9 | 7 | 10 | 8 |
| 92 |  |  |  |  |  |  |  |  |  |  |  |  |
| 93 | 3 | 2 | 1 | 6 | 3 | 1 | 8 | 2 | 2 | 2 | 4 | 2 |
| 94 | 3 | 1 | 4 | 0 | 1 | 0 | 1 | 3 | 2 | 1 | 5 | 2 |
| 95 | 2 | 1 | 1 | 5 | 4 | 2 | 5 | 4 | 1 | 3 | 1 | 0 |
| 96 | 2 | 5 | 5 | 3 | 7 | 4 | 8 | 5 | 1 | 3 | 5 | 0 |
| 97 |  |  |  |  |  |  |  |  |  |  |  |  |
| 98 | 5 | 6 | 6 | 6 | 6 | 6 | 3 | 1 | 4 | 1 | 5 | 3 |
| 99 | 3 | 6 | 1 | 0 | 5 | 0 | 2 | 1 | 2 | 2 | 4 | 3 |
| 100 | 4 | 6 | 5 | 3 | 6 | 3 | 3 | 4 | 4 | 2 | 3 | 3 |
| 101 | 12 | 4 | 9 | 15 | 5 | 4 | 4 | 7 | 2 | 6 | 8 | 7 |
| 102 |  |  |  |  |  |  |  |  |  |  |  |  |
| 103 | 7 | 8 | 5 | 8 | 9 | 6 | 8 | 8 | 7 | 9 | 5 | 8 |
| 104 | 2 | 8 | 6 | 8 | 9 | 5 | 4 | 6 | 6 | 10 | 7 | 10 |
| 105 | 2 | 1 | 6 | 4 | 4 | 5 | 3 | 10 | 4 | 4 | 5 | 3 |

Table 30. Number of newly diagnosed stage IV lung cancer during each month in 2020.

|  | Month | | | | | | | | | | | |
| --- | --- | --- | --- | --- | --- | --- | --- | --- | --- | --- | --- | --- |
| Hospital | 1 | 2 | 3 | 4 | 5 | 6 | 7 | 8 | 9 | 10 | 11 | 12 |
| 1 | 1 | 4 | 4 | 5 | 4 | 4 | 1 | 4 | 5 | 4 | 2 | 2 |
| 2 | 10 | 10 | 12 | 11 | 8 | 11 | 19 | 7 | 9 | 11 | 15 | 13 |
| 3 | 2 | 3 | 1 | 1 | 3 | 2 | 0 | 1 | 2 | 5 | 2 | 4 |
| 4 | 9 | 6 | 8 | 6 | 6 | 5 | 10 | 6 | 9 | 7 | 9 | 5 |
| 5 | 3 | 3 | 2 | 5 | 2 | 2 | 3 | 2 | 4 | 4 | 5 | 2 |
| 6 | 8 | 5 | 5 | 6 | 5 | 7 | 4 | 4 | 6 | 8 | 2 | 10 |
| 7 | 2 | 6 | 1 | 3 | 3 | 5 | 2 | 2 | 1 | 5 | 1 | 4 |
| 8 |  |  |  |  |  |  |  |  |  |  |  |  |
| 9 | 7 | 2 | 3 | 7 | 6 | 6 | 6 | 6 | 4 | 4 | 4 | 6 |
| 10 | 5 | 2 | 2 | 6 | 4 | 3 | 6 | 2 | 3 | 5 | 3 | 0 |
| 11 | 10 | 11 | 9 | 7 | 10 | 12 | 9 | 6 | 7 | 12 | 14 | 12 |
| 12 | 2 | 3 | 1 | 2 | 1 | 1 | 3 | 1 | 0 | 1 | 1 | 3 |
| 13 | 7 | 11 | 13 | 5 | 4 | 5 | 5 | 4 | 3 | 9 | 4 | 6 |
| 14 | 4 | 3 | 5 | 3 | 2 | 4 | 9 | 8 | 2 | 4 | 5 | 6 |
| 15 | 11 | 10 | 8 | 14 | 7 | 14 | 17 | 8 | 10 | 12 | 13 | 13 |
| 16 | 6 | 10 | 10 | 12 | 7 | 10 | 12 | 5 | 13 | 9 | 11 | 13 |
| 17 | 5 | 4 | 2 | 0 | 3 | 4 | 0 | 0 | 0 | 3 | 1 | 3 |
| 18 | 7 | 4 | 6 | 3 | 4 | 7 | 6 | 6 | 7 | 10 | 8 | 5 |
| 19 | 1 | 1 | 6 | 2 | 3 | 6 | 3 | 6 | 6 | 1 | 4 | 5 |
| 20 | 16 | 20 | 18 | 6 | 16 | 19 | 20 | 21 | 27 | 22 | 17 | 28 |
| 21 | 12 | 6 | 6 | 5 | 4 | 7 | 3 | 8 | 4 | 4 | 7 | 6 |
| 22 | 12 | 3 | 9 | 4 | 7 | 8 | 7 | 1 | 6 | 7 | 3 | 5 |
| 23 |  |  |  |  |  |  |  |  |  |  |  |  |
| 24 | 9 | 6 | 10 | 9 | 9 | 3 | 5 | 11 | 6 | 14 | 6 | 5 |
| 25 | 4 | 5 | 10 | 1 | 5 | 7 | 7 | 6 | 10 | 8 | 5 | 6 |
| 26 | 3 | 8 | 6 | 14 | 5 | 12 | 9 | 10 | 13 | 7 | 11 | 9 |
| 27 | 5 | 3 | 2 | 1 | 2 | 4 | 11 | 5 | 2 | 7 | 3 | 0 |
| 28 | 3 | 8 | 2 | 7 | 9 | 5 | 10 | 6 | 4 | 5 | 5 | 2 |
| 29 |  |  |  |  |  |  |  |  |  |  |  |  |
| 30 | 4 | 3 | 5 | 5 | 3 | 7 | 5 | 4 | 4 | 4 | 2 | 8 |
| 31 | 2 | 3 | 1 | 2 | 4 | 6 | 3 | 4 | 2 | 2 | 5 | 2 |
| 32 | 2 | 5 | 3 | 4 | 5 | 5 | 3 | 5 | 4 | 5 | 4 | 7 |
| 33 | 6 | 4 | 10 | 4 | 5 | 4 | 10 | 7 | 6 | 8 | 0 | 4 |
| 34 |  |  |  |  |  |  |  |  |  |  |  |  |
| 35 | 7 | 2 | 13 | 6 | 8 | 7 | 8 | 11 | 12 | 8 | 9 | 7 |
| 36 | 1 |  | 3 | 1 | 1 | 4 |  |  |  | 3 | 1 | 1 |
| 37 | 12 | 7 | 17 | 13 | 8 | 16 | 5 | 8 | 9 | 10 | 6 | 12 |
| 38 |  |  |  |  |  |  |  |  |  |  |  |  |
| 39 |  | 1 | 1 |  | 1 | 1 |  |  | 1 | 1 |  | 2 |
| 40 | 5 | 4 | 5 | 3 | 2 | 11 | 4 | 2 | 5 | 9 | 5 | 3 |
| 41 | 3 | 1 | 1 | 2 | 2 | 2 | 2 | 5 | 3 | 4 | 3 | 2 |
| 42 | 2 | 6 | 8 | 6 | 5 | 7 | 6 | 2 | 7 | 4 | 5 | 7 |
| 43 | 6 | 8 | 3 | 2 | 5 | 9 | 5 | 7 | 8 | 5 | 4 | 9 |
| 44 | 4 | 3 | 1 | 1 | 2 | 4 | 3 | 2 | 1 | 3 | 0 | 6 |
| 45 | 3 | 3 | 6 | 8 | 11 | 3 | 7 | 4 | 8 | 5 | 6 | 1 |
| 46 | 4 | 5 | 4 | 2 | 1 | 6 | 5 | 4 | 6 | 2 | 1 | 4 |
| 47 |  |  |  |  |  |  |  |  |  |  |  |  |
| 48 | 6 | 5 | 5 | 5 | 1 | 8 | 9 | 3 | 5 | 3 | 4 | 4 |
| 49 | 3 | 3 | 4 | 12 | 4 | 8 | 4 | 4 | 7 | 5 | 3 | 7 |
| 50 | 1 | 0 | 1 | 0 | 0 | 0 | 0 | 0 | 0 | 0 | 1 | 1 |
| 51 | 4 | 4 | 9 | 10 | 4 | 3 | 3 | 4 | 7 | 8 | 4 | 3 |
| 52 | 2 | 4 | 1 | 7 | 4 | 6 | 6 | 6 | 4 | 5 | 6 | 3 |
| 53 | 6 | 14 | 9 | 10 | 13 | 16 | 16 | 7 | 6 | 9 | 14 | 7 |
| 54 | 29 | 33 | 29 | 20 | 20 | 28 | 14 | 22 | 23 | 27 | 20 | 31 |
| 55 | 3 | 4 | 5 | 1 | 3 | 6 | 4 | 3 | 3 | 3 | 4 | 3 |
| 56 | 6 | 4 | 11 | 4 | 8 | 11 | 9 | 2 | 7 | 8 | 6 | 6 |
| 57 | 16 | 18 | 9 | 14 | 4 | 11 | 17 | 5 | 12 | 16 | 9 | 12 |
| 58 | 4 | 3 | 5 | 1 | 5 | 7 | 3 | 5 | 4 | 9 | 3 | 5 |
| 59 | 3 | 6 | 8 | 10 | 6 | 3 | 3 | 7 | 3 | 5 | 3 | 1 |
| 60 | 3 | 9 | 11 | 5 | 4 | 4 | 4 | 5 | 6 | 5 | 3 | 9 |
| 61 | 10 | 4 | 4 | 11 | 7 | 7 | 5 | 4 | 9 | 8 | 7 | 11 |
| 62 |  |  |  |  |  |  |  |  |  |  |  |  |
| 63 |  |  |  |  |  |  |  |  |  |  |  |  |
| 64 | 9 | 9 | 12 | 16 | 9 | 8 | 5 | 6 | 7 | 11 | 8 | 8 |
| 65 | 7 | 7 | 5 | 5 | 4 | 8 | 4 | 7 | 3 | 4 | 5 | 2 |
| 66 | 2 | 0 | 3 | 2 | 2 | 2 | 2 | 1 | 0 | 0 | 1 | 1 |
| 67 | 3 | 4 | 4 | 11 | 8 | 4 | 5 | 9 | 7 | 9 | 6 | 5 |
| 68 | 4 | 4 | 4 | 2 | 1 | 5 | 5 | 8 | 8 | 5 | 6 | 4 |
| 68 | 20 | 24 | 19 | 16 | 19 | 27 | 21 | 24 | 25 | 33 | 24 | 26 |
| 70 |  | 2 | 1 |  | 1 | 2 | 1 | 1 | 1 | 1 | 4 | 1 |
| 71 | 5 | 9 | 8 | 6 | 3 | 5 | 4 | 5 | 3 | 9 | 5 | 2 |
| 72 | 10 | 6 | 4 | 4 | 5 | 13 | 18 | 8 | 12 | 7 | 10 | 12 |
| 73 | 2 | 3 | 3 | 2 | 1 | 4 | 2 | 4 | 7 | 4 | 9 | 4 |
| 74 | 9 | 13 | 9 | 9 | 1 | 14 | 5 | 13 | 19 | 14 | 11 | 7 |
| 75 |  |  |  |  |  |  |  |  |  |  |  |  |
| 76 | 4 | 7 | 5 | 7 | 3 | 3 | 6 | 6 | 7 | 7 | 6 | 8 |
| 77 | 1 | 1 | 1 | 1 | 1 | 2 | 1 | 1 | 2 |  |  | 2 |
| 78 | 2 | 2 | 1 | 2 | 2 |  |  | 2 | 3 | 2 | 1 |  |
| 79 | 7 | 6 | 3 | 3 | 4 | 5 | 3 | 8 | 10 | 6 | 4 | 4 |
| 80 | 11 | 3 | 8 | 6 | 6 | 8 | 6 | 5 | 9 | 6 | 5 | 7 |
| 81 | 5 | 3 | 2 | 3 | 3 | 9 | 4 | 5 | 8 | 3 | 6 | 4 |
| 82 | 1 | 2 | 5 | 0 | 3 | 2 | 1 | 2 | 3 | 5 | 2 | 1 |
| 83 | 4 | 3 | 2 | 4 | 4 | 5 | 2 | 1 | 6 | 7 | 8 | 5 |
| 84 | 5 | 8 | 0 | 5 | 4 | 7 | 5 | 3 | 4 | 6 | 3 | 6 |
| 85 | 13 | 8 | 14 | 17 | 7 | 11 | 10 | 16 | 13 | 17 | 12 | 5 |
| 86 | 4 | 7 | 8 | 6 | 4 | 7 | 5 | 5 | 4 | 7 | 7 | 6 |
| 87 | 5 | 4 | 4 | 4 | 4 | 3 | 4 | 3 |  | 1 | 2 | 5 |
| 88 | 6 | 3 | 2 | 6 | 4 | 7 | 4 | 3 | 1 | 6 | 6 | 2 |
| 89 | 19 | 12 | 23 | 21 | 15 | 24 | 24 | 16 | 22 | 24 | 17 | 25 |
| 90 | 9 | 8 | 4 | 4 | 3 | 8 | 8 | 12 | 8 | 9 | 9 | 5 |
| 91 | 16 | 12 | 7 | 13 | 7 | 9 | 6 | 9 | 3 | 3 | 8 | 7 |
| 92 |  |  |  |  |  |  |  |  |  |  |  |  |
| 93 | 3 | 5 | 5 | 0 | 3 | 3 | 4 | 2 | 2 | 2 | 3 | 0 |
| 94 | 2 | 5 | 1 | 5 | 4 | 2 | 1 | 2 | 5 | 2 | 1 | 4 |
| 95 | 2 | 0 | 1 | 0 | 1 | 1 | 2 | 2 | 0 | 2 | 4 | 0 |
| 96 | 4 | 4 | 7 | 5 | 4 | 6 | 7 | 5 | 4 | 9 | 10 | 3 |
| 97 |  |  |  |  |  |  |  |  |  |  |  |  |
| 98 | 1 | 5 | 2 | 1 | 4 | 4 | 1 | 1 | 1 | 0 | 5 | 1 |
| 99 | 1 | 3 | 0 | 1 | 5 | 3 | 7 | 2 | 1 | 3 | 5 | 0 |
| 100 | 7 | 8 | 4 | 15 | 18 | 6 | 6 | 6 | 8 | 6 | 4 | 2 |
| 101 | 4 | 4 | 5 | 5 | 3 | 5 | 6 | 8 | 4 | 9 | 6 | 10 |
| 102 |  |  |  |  |  |  |  |  |  |  |  |  |
| 103 | 3 | 4 | 3 | 2 | 12 | 9 | 5 | 4 | 3 | 3 | 5 | 3 |
| 104 | 8 | 8 | 5 | 6 | 4 | 11 | 9 | 6 | 8 | 7 | 9 | 7 |
| 105 | 4 | 4 | 2 | 3 | 4 | 2 | 5 | 2 | 7 | 6 | 3 | 4 |

Table 31. Number of newly diagnosed breast cancer during each month in 2019.

|  | Month | | | | | | | | | | | |
| --- | --- | --- | --- | --- | --- | --- | --- | --- | --- | --- | --- | --- |
| Hospital | 1 | 2 | 3 | 4 | 5 | 6 | 7 | 8 | 9 | 10 | 11 | 12 |
| 1 | 1 | 2 | 4 | 2 | 3 | 3 | 6 | 0 | 2 | 0 | 2 | 0 |
| 2 | 110 | 81 | 91 | 110 | 98 | 89 | 94 | 99 | 87 | 102 | 104 | 92 |
| 3 | 3 | 5 | 5 | 7 | 4 | 4 | 10 | 6 | 5 | 5 | 2 | 5 |
| 4 | 34 | 46 | 40 | 39 | 35 | 30 | 49 | 42 | 29 | 40 | 48 | 42 |
| 5 | 2 | 3 | 6 | 3 | 2 | 0 | 9 | 4 | 3 | 7 | 5 | 6 |
| 6 | 23 | 13 | 16 | 29 | 35 | 23 | 21 | 17 | 19 | 30 | 22 | 17 |
| 7 | 7 | 10 | 7 | 9 | 14 | 8 | 14 | 15 | 16 | 8 | 16 | 11 |
| 8 |  |  |  |  |  |  |  |  |  |  |  |  |
| 9 | 6 | 8 | 113 | 12 | 11 | 9 | 23 | 18 | 14 | 16 | 10 | 12 |
| 10 | 10 | 8 | 15 | 22 | 27 | 27 | 11 | 17 | 23 | 14 | 21 | 16 |
| 11 | 17 | 8 | 8 | 19 | 18 | 19 | 14 | 5 | 13 | 24 | 11 | 10 |
| 12 | 3 | 3 | 4 | 0 | 3 | 3 | 4 | 7 | 2 | 2 | 3 | 10 |
| 13 | 31 | 37 | 46 | 39 | 35 | 38 | 37 | 31 | 38 | 38 | 30 | 36 |
| 14 | 8 | 10 | 10 | 5 | 13 | 12 | 15 | 13 | 11 | 17 | 11 | 11 |
| 15 | 24 | 14 | 16 | 24 | 21 | 17 | 21 | 17 | 27 | 32 | 19 | 26 |
| 16 | 33 | 28 | 31 | 20 | 31 | 24 | 36 | 33 | 29 | 38 | 27 | 29 |
| 17 | 5 | 5 | 4 | 2 | 2 | 3 | 2 | 6 | 2 | 1 | 4 | 1 |
| 18 | 15 | 14 | 16 | 15 | 10 | 16 | 13 | 15 | 12 | 17 | 11 | 16 |
| 19 | 6 | 6 | 6 | 7 | 11 | 7 | 12 | 8 | 7 | 15 | 10 | 4 |
| 20 | 42 | 47 | 48 | 38 | 53 | 39 | 49 | 43 | 43 | 52 | 45 | 48 |
| 21 | 24 | 17 | 15 | 12 | 21 | 11 | 13 | 22 | 20 | 23 | 12 | 21 |
| 22 | 20 | 25 | 19 | 28 | 21 | 26 | 20 | 33 | 23 | 28 | 26 | 24 |
| 23 |  |  |  |  |  |  |  |  |  |  |  |  |
| 24 | 14 | 12 | 4 | 17 | 22 | 14 | 14 | 20 | 22 | 18 | 14 | 18 |
| 25 | 19 | 8 | 10 | 22 | 25 | 21 | 28 | 27 | 25 | 18 | 22 | 18 |
| 26 | 14 | 13 | 15 | 16 | 11 | 16 | 15 | 11 | 11 | 20 | 7 | 6 |
| 27 | 27 | 25 | 22 | 25 | 24 | 26 | 25 | 24 | 25 | 30 | 26 | 22 |
| 28 | 9 | 8 | 7 | 8 | 19 | 6 | 28 | 15 | 22 | 10 | 7 | 5 |
| 29 | 53 | 51 | 48 | 54 | 36 | 48 | 43 | 46 | 35 | 44 | 47 | 40 |
| 30 | 5 | 6 | 3 | 11 | 8 | 5 | 10 | 8 | 10 | 8 | 4 | 5 |
| 31 | 18 | 22 | 20 | 23 | 15 | 14 | 22 | 13 | 16 | 28 | 13 | 24 |
| 32 | 14 | 12 | 18 | 21 | 13 | 16 | 20 | 17 | 12 | 11 | 17 | 19 |
| 33 | 6 | 5 | 7 | 5 | 7 | 5 | 7 | 6 | 5 | 7 | 8 | 11 |
| 34 | 10 | 13 | 12 | 18 | 11 | 20 | 20 | 17 | 12 | 25 | 10 | 17 |
| 35 | 6 | 5 | 7 | 8 | 9 | 5 | 7 | 7 | 9 | 8 | 8 | 7 |
| 36 | 14 | 11 | 9 | 14 | 13 | 10 | 13 | 9 | 18 | 21 | 12 | 20 |
| 37 | 30 | 30 | 30 | 34 | 32 | 27 | 33 | 33 | 31 | 33 | 29 | 30 |
| 38 | 18 | 14 | 19 | 23 | 23 | 21 | 25 | 29 | 20 | 25 | 27 | 18 |
| 39 |  |  |  |  |  |  |  |  |  |  |  |  |
| 40 | 21 | 17 | 25 | 31 | 27 | 30 | 32 | 28 | 27 | 47 | 34 | 40 |
| 41 | 11 | 6 | 13 | 11 | 14 | 14 | 15 | 15 | 6 | 11 | 12 | 11 |
| 42 | 13 | 5 | 16 | 9 | 10 | 12 | 9 | 11 | 6 | 11 | 12 | 15 |
| 43 | 11 | 10 | 6 | 11 | 18 | 11 | 12 | 7 | 8 | 8 | 7 | 16 |
| 44 | 3 | 2 | 0 | 4 | 4 | 2 | 4 | 2 | 7 | 7 | 2 | 6 |
| 45 | 14 | 9 | 15 | 8 | 15 | 13 | 15 | 12 | 10 | 12 | 15 | 12 |
| 46 | 22 | 27 | 22 | 47 | 38 | 17 | 36 | 31 | 32 | 25 | 14 | 26 |
| 47 |  |  |  |  |  |  |  |  |  |  |  |  |
| 48 | 12 | 12 | 5 | 14 | 9 | 4 | 10 | 15 | 6 | 8 | 11 | 12 |
| 49 | 5 | 8 | 9 | 7 | 8 | 8 | 14 | 9 | 14 | 18 | 20 | 9 |
| 50 | 2 | 1 | 1 | 1 | 3 | 1 | 1 | 0 | 1 | 4 | 2 | 2 |
| 51 | 15 | 18 | 24 | 19 | 17 | 11 | 17 | 15 | 19 | 19 | 14 | 19 |
| 52 | 22 | 14 | 19 | 25 | 20 | 22 | 26 | 27 | 24 | 23 | 21 | 26 |
| 53 | 23 | 12 | 12 | 22 | 13 | 11 | 24 | 21 | 16 | 23 | 19 | 20 |
| 54 | 43 | 55 | 48 | 51 | 40 | 40 | 50 | 60 | 43 | 61 | 61 | 56 |
| 55 | 6 | 5 | 18 | 13 | 8 | 5 | 18 | 10 | 10 | 9 | 10 | 7 |
| 56 | 48 | 52 | 38 | 45 | 35 | 47 | 39 | 47 | 51 | 54 | 50 | 48 |
| 57 | 38 | 43 | 43 | 46 | 48 | 34 | 52 | 31 | 47 | 53 | 54 | 46 |
| 58 | 12 | 8 | 15 | 7 | 10 | 11 | 12 | 8 | 13 | 11 | 16 | 9 |
| 59 | 2 | 6 | 10 | 10 | 11 | 8 | 8 | 5 | 5 | 6 | 1 | 5 |
| 60 | 19 | 18 | 14 | 8 | 10 | 15 | 15 | 14 | 13 | 23 | 13 | 18 |
| 61 | 7 | 4 | 10 | 7 | 7 | 15 | 6 | 13 | 12 | 11 | 13 | 11 |
| 62 | 29 | 28 | 17 | 23 | 28 | 28 | 30 | 23 | 21 | 25 | 21 | 26 |
| 63 | 3 | 2 | 1 | 2 | 2 |  | 4 | 3 | 8 | 5 | 3 |  |
| 64 |  |  |  |  |  |  |  |  |  |  |  |  |
| 65 | 7 | 17 | 11 | 13 | 10 | 20 | 22 | 19 | 16 | 16 | 18 | 23 |
| 66 | 1 | 7 | 4 | 4 | 4 | 2 | 4 | 2 | 7 | 1 | 2 | 2 |
| 67 | 8 | 11 | 9 | 16 | 10 | 13 | 13 | 11 | 14 | 12 | 11 | 14 |
| 68 | 19 | 15 | 18 | 19 | 23 | 18 | 20 | 26 | 10 | 35 | 20 | 22 |
| 68 | 22 | 16 | 30 | 17 | 16 | 18 | 17 | 11 | 13 | 9 | 9 | 10 |
| 70 | 3 | 4 | 4 | 3 | 3 | 1 | 7 | 4 | 6 | 4 | 3 | 4 |
| 71 | 12 | 15 | 17 | 14 | 15 | 24 | 18 | 27 | 13 | 20 | 23 | 20 |
| 72 | 22 | 17 | 25 | 21 | 17 | 22 | 16 | 20 | 20 | 17 | 13 | 31 |
| 73 |  |  |  |  |  |  |  |  |  |  |  |  |
| 74 | 38 | 36 | 41 | 31 | 28 | 33 | 47 | 35 | 31 | 40 | 25 | 29 |
| 75 | 1 | 1 | 3 | 1 | 1 | 2 | 1 | 3 | 2 | 1 | 0 | 0 |
| 76 | 34 | 33 | 26 | 25 | 32 | 33 | 35 | 34 | 22 | 38 | 35 | 35 |
| 77 | 2 | 1 | 0 | 1 | 3 | 0 | 3 | 1 | 0 | 3 | 0 | 0 |
| 78 |  |  |  |  |  |  |  |  |  |  |  |  |
| 79 | 30 | 27 | 19 | 17 | 25 | 26 | 24 | 19 | 29 | 39 | 52 | 58 |
| 80 | 13 | 8 | 14 | 18 | 14 | 13 | 25 | 20 | 18 | 15 | 15 | 11 |
| 81 | 13 | 6 | 6 | 5 | 9 | 9 | 6 | 5 | 6 | 17 | 12 | 10 |
| 82 |  |  |  |  |  |  |  |  |  |  |  |  |
| 83 |  |  |  |  |  |  |  |  |  |  |  |  |
| 84 | 21 | 25 | 25 | 20 | 19 | 28 | 27 | 27 | 30 | 26 | 32 | 25 |
| 85 | 14 | 25 | 33 | 21 | 24 | 22 | 34 | 18 | 28 | 36 | 33 | 22 |
| 86 | 12 | 12 | 9 | 12 | 17 | 7 | 9 | 10 | 11 | 16 | 13 | 11 |
| 87 | 5 | 7 | 2 | 8 | 4 | 8 | 14 | 4 | 9 | 7 | 4 | 10 |
| 88 | 16 | 12 | 11 | 19 | 22 | 16 | 12 | 14 | 11 | 12 | 12 | 10 |
| 89 | 37 | 45 | 41 | 40 | 46 | 44 | 29 | 31 | 51 | 44 | 40 | 38 |
| 90 | 21 | 19 | 16 | 29 | 13 | 16 | 18 | 19 | 25 | 21 | 33 | 12 |
| 91 | 10 | 10 | 8 | 19 | 12 | 5 | 8 | 9 | 13 | 10 | 14 | 8 |
| 92 |  |  |  |  |  |  |  |  |  |  |  |  |
| 93 |  |  |  |  |  |  |  |  |  |  |  |  |
| 94 | 4 | 4 | 6 | 3 | 5 | 3 | 0 | 10 | 7 | 3 | 6 | 2 |
| 95 | 4 | 4 | 3 | 5 | 3 | 8 | 5 | 6 | 8 | 9 | 9 | 4 |
| 96 | 0 | 0 | 0 | 1 | 1 | 2 | 0 | 2 | 0 | 3 | 0 | 0 |
| 97 |  |  |  |  |  |  |  |  |  |  |  |  |
| 98 | 12 | 14 | 7 | 17 | 13 | 11 | 12 | 11 | 10 | 4 | 16 | 15 |
| 99 | 5 | 10 | 11 | 7 | 10 | 5 | 9 | 10 | 12 | 5 | 7 | 8 |
| 100 | 7 | 7 | 14 | 11 | 8 | 13 | 11 | 12 | 9 | 10 | 6 | 12 |
| 101 | 26 | 24 | 12 | 33 | 15 | 24 | 27 | 25 | 19 | 20 | 24 | 21 |
| 102 |  |  |  |  |  |  |  |  |  |  |  |  |
| 103 | 15 | 28 | 19 | 10 | 20 | 16 | 24 | 28 | 20 | 17 | 21 | 13 |
| 104 | 22 | 26 | 21 | 19 | 33 | 22 | 27 | 4 | 4 | 18 | 15 | 13 |
| 105 | 12 | 18 | 15 | 11 | 17 | 7 | 13 | 12 | 12 | 15 | 10 | 17 |

Table 32. Number of newly diagnosed breast cancer during each month in 2020.

|  | Month | | | | | | | | | | | |
| --- | --- | --- | --- | --- | --- | --- | --- | --- | --- | --- | --- | --- |
| Hospital | 1 | 2 | 3 | 4 | 5 | 6 | 7 | 8 | 9 | 10 | 11 | 12 |
| 1 | 1 | 5 | 2 | 4 | 1 | 0 | 3 | 3 | 6 | 2 | 3 | 2 |
| 2 | 90 | 81 | 83 | 95 | 59 | 75 | 59 | 79 | 74 | 72 | 86 | 105 |
| 3 | 3 | 3 | 3 | 2 | 1 | 6 | 5 | 3 | 5 | 2 | 3 | 5 |
| 4 | 39 | 39 | 37 | 33 | 32 | 28 | 31 | 35 | 33 | 30 | 35 | 54 |
| 5 | 4 | 3 | 4 | 1 | 3 | 5 | 5 | 6 | 7 | 2 | 4 | 5 |
| 6 | 17 | 18 | 16 | 18 | 8 | 18 | 20 | 18 | 16 | 28 | 19 | 11 |
| 7 | 12 | 5 | 7 | 6 | 9 | 12 | 9 | 7 | 10 | 12 | 14 | 4 |
| 8 |  |  |  |  |  |  |  |  |  |  |  |  |
| 9 | 8 | 12 | 11 | 13 | 6 | 20 | 8 | 11 | 13 | 11 | 9 | 13 |
| 10 | 13 | 16 | 14 | 10 | 3 | 24 | 19 | 18 | 15 | 29 | 19 | 14 |
| 11 | 7 | 7 | 23 | 21 | 12 | 12 | 15 | 11 | 13 | 13 | 15 | 15 |
| 12 | 2 | 3 | 4 | 4 | 3 | 4 | 8 | 1 | 7 | 4 | 1 | 5 |
| 13 | 32 | 35 | 35 | 24 | 18 | 29 | 27 | 19 | 37 | 47 | 25 | 28 |
| 14 | 9 | 8 | 8 | 18 | 9 | 8 | 16 | 14 | 7 | 14 | 17 | 16 |
| 15 | 18 | 27 | 25 | 18 | 17 | 28 | 25 | 15 | 21 | 26 | 19 | 30 |
| 16 | 33 | 31 | 18 | 34 | 29 | 41 | 36 | 29 | 39 | 26 | 35 | 38 |
| 17 | 5 | 2 | 3 | 7 | 3 | 4 | 1 | 2 | 1 | 5 | 5 | 4 |
| 18 | 13 | 10 | 9 | 9 | 8 | 11 | 10 | 15 | 14 | 12 | 12 | 13 |
| 19 | 7 | 4 | 9 | 4 | 7 | 3 | 4 | 11 | 12 | 9 | 9 | 6 |
| 20 | 63 | 47 | 42 | 19 | 31 | 34 | 39 | 39 | 45 | 65 | 66 | 66 |
| 21 | 17 | 21 | 18 | 14 | 10 | 7 | 20 | 16 | 12 | 15 | 12 | 13 |
| 22 | 22 | 17 | 24 | 16 | 14 | 25 | 18 | 17 | 26 | 26 | 22 | 28 |
| 23 |  |  |  |  |  |  |  |  |  |  |  |  |
| 24 | 27 | 14 | 18 | 7 | 11 | 15 | 11 | 11 | 13 | 16 | 9 | 9 |
| 25 | 17 | 16 | 24 | 27 | 19 | 28 | 29 | 25 | 20 | 33 | 22 | 35 |
| 26 | 12 | 5 | 14 | 12 | 10 | 10 | 6 | 14 | 13 | 13 | 17 | 11 |
| 27 | 27 | 23 | 17 | 21 | 15 | 26 | 15 | 13 | 14 | 19 | 22 | 21 |
| 28 | 13 | 5 | 15 | 17 | 5 | 12 | 16 | 12 | 11 | 11 | 11 | 9 |
| 29 | 50 | 42 | 58 | 55 | 34 | 46 | 41 | 43 | 44 | 47 | 52 | 60 |
| 30 | 4 | 4 | 12 | 7 | 9 | 5 | 4 | 10 | 8 | 6 | 8 | 11 |
| 31 | 14 | 24 | 27 | 16 | 16 | 19 | 22 | 17 | 15 | 34 | 20 | 18 |
| 32 | 11 | 10 | 13 | 15 | 16 | 18 | 14 | 11 | 7 | 22 | 13 | 14 |
| 33 | 4 | 6 | 7 | 7 | 6 | 5 | 8 | 6 | 9 | 8 | 4 | 4 |
| 34 | 12 | 22 | 22 | 0 | 1 | 7 | 12 | 13 | 12 | 17 | 9 | 18 |
| 35 | 10 | 0 | 7 | 4 | 7 | 10 | 6 | 8 | 12 | 11 | 11 | 8 |
| 36 | 14 | 14 | 20 | 11 | 14 | 11 | 17 | 9 | 13 | 12 | 14 | 13 |
| 37 | 33 | 22 | 30 | 24 | 31 | 31 | 26 | 22 | 21 | 19 | 23 | 24 |
| 38 | 19 | 21 | 16 | 18 | 12 | 20 | 15 | 25 | 19 | 29 | 30 | 21 |
| 39 |  |  |  |  |  |  |  |  |  |  |  |  |
| 40 | 47 | 22 | 29 | 28 | 26 | 25 | 23 | 22 | 26 | 36 | 34 | 27 |
| 41 | 11 | 12 | 12 | 17 | 6 | 18 | 17 | 12 | 15 | 17 | 16 | 13 |
| 42 | 17 | 7 | 17 | 12 | 8 | 11 | 9 | 7 | 5 | 7 | 7 | 14 |
| 43 | 15 | 8 | 10 | 8 | 5 | 9 | 10 | 13 | 8 | 14 | 13 | 8 |
| 44 | 5 | 1 | 4 | 2 | 2 | 5 | 1 | 5 | 1 | 4 | 5 | 3 |
| 45 | 8 | 11 | 10 | 15 | 13 | 15 | 12 | 15 | 12 | 14 | 12 | 11 |
| 46 | 24 | 20 | 22 | 26 | 16 | 24 | 15 | 14 | 19 | 27 | 21 | 22 |
| 47 |  |  |  |  |  |  |  |  |  |  |  |  |
| 48 | 14 | 12 | 13 | 9 | 7 | 10 | 9 | 10 | 8 | 13 | 12 | 7 |
| 49 | 8 | 14 | 10 | 7 | 10 | 7 | 4 | 8 | 10 | 14 | 9 | 7 |
| 50 | 2 | 0 | 0 | 1 | 2 | 3 | 2 | 6 | 1 | 5 | 0 | 4 |
| 51 | 11 | 14 | 17 | 12 | 9 | 22 | 22 | 13 | 18 | 24 | 11 | 17 |
| 52 | 22 | 14 | 32 | 22 | 14 | 19 | 19 | 16 | 25 | 26 | 22 | 36 |
| 53 | 17 | 13 | 23 | 12 | 6 | 16 | 13 | 12 | 14 | 15 | 10 | 22 |
| 54 | 53 | 49 | 52 | 47 | 33 | 40 | 55 | 52 | 49 | 63 | 50 | 65 |
| 55 | 4 | 8 | 7 | 5 | 9 | 10 | 13 | 9 | 5 | 11 | 7 | 5 |
| 56 | 45 | 47 | 43 | 35 | 19 | 27 | 39 | 45 | 36 | 51 | 48 | 38 |
| 57 | 48 | 46 | 42 | 25 | 22 | 47 | 34 | 32 | 45 | 58 | 40 | 38 |
| 58 | 15 | 9 | 14 | 7 | 3 | 8 | 9 | 11 | 16 | 14 | 17 | 13 |
| 59 | 6 | 8 | 6 | 4 | 3 | 8 | 8 | 7 | 9 | 4 | 7 | 3 |
| 60 | 10 | 13 | 19 | 18 | 8 | 8 | 13 | 2 | 0 | 9 | 18 | 17 |
| 61 | 14 | 6 | 6 | 7 | 9 | 9 | 2 | 7 | 7 | 11 | 7 | 13 |
| 62 | 21 | 26 | 19 | 17 | 12 | 21 | 23 | 10 | 26 | 26 | 14 | 26 |
| 63 | 2 | 1 | 4 | 1 | 1 | 2 | 4 | 5 | 7 | 2 |  | 4 |
| 64 |  |  |  |  |  |  |  |  |  |  |  |  |
| 65 | 11 | 11 | 8 | 25 | 32 | 19 | 15 | 20 | 11 | 13 | 13 | 9 |
| 66 | 3 | 3 | 4 | 2 | 3 | 5 | 4 | 4 | 4 | 3 | 2 | 5 |
| 67 | 8 | 14 | 6 | 9 | 9 | 6 | 9 | 10 | 11 | 8 | 9 | 3 |
| 68 | 13 | 29 | 13 | 13 | 9 | 26 | 17 | 24 | 19 | 23 | 15 | 24 |
| 68 | 10 | 3 | 2 | 2 | 2 | 5 | 2 | 9 | 2 | 7 | 2 | 1 |
| 70 | 3 | 9 | 7 | 1 | 0 | 3 | 5 | 5 | 4 | 5 | 7 | 3 |
| 71 | 28 | 17 | 13 | 13 | 12 | 15 | 19 | 27 | 15 | 20 | 15 | 18 |
| 72 | 33 | 18 | 22 | 21 | 13 | 18 | 20 | 14 | 23 | 29 | 26 | 26 |
| 73 |  |  |  |  |  |  |  |  |  |  |  |  |
| 74 | 41 | 37 | 42 | 32 | 25 | 35 | 31 | 34 | 37 | 24 | 26 | 43 |
| 75 | 1 | 1 | 0 | 0 | 0 | 0 | 3 | 0 | 0 | 0 | 0 | 0 |
| 76 | 34 | 29 | 19 | 21 | 27 | 30 | 19 | 31 | 29 | 25 | 32 | 36 |
| 77 | 1 | 0 | 2 | 1 | 0 | 2 | 1 | 0 | 1 | 4 | 0 | 1 |
| 78 |  |  |  |  |  |  |  |  |  |  |  |  |
| 79 | 34 | 18 | 31 | 16 | 16 | 30 | 37 | 23 | 29 | 27 | 44 | 27 |
| 80 | 18 | 8 | 13 | 11 | 9 | 16 | 24 | 18 | 17 | 22 | 19 | 18 |
| 81 | 8 | 10 | 10 | 5 | 6 | 10 | 5 | 6 | 9 | 8 | 9 | 5 |
| 82 |  |  |  |  |  |  |  |  |  |  |  |  |
| 83 |  |  |  |  |  |  |  |  |  |  |  |  |
| 84 | 29 | 17 | 24 | 31 | 17 | 26 | 23 | 20 | 20 | 32 | 31 | 24 |
| 85 | 22 | 13 | 37 | 18 | 10 | 25 | 32 | 27 | 30 | 26 | 31 | 20 |
| 86 | 11 | 18 | 10 | 18 | 5 | 22 | 14 | 10 | 10 | 17 | 10 | 12 |
| 87 | 11 | 6 | 7 | 9 | 6 | 5 | 8 | 6 | 3 | 2 | 3 | 4 |
| 88 | 9 | 15 | 9 | 10 | 5 | 8 | 15 | 11 | 8 | 21 | 8 | 6 |
| 89 | 43 | 39 | 44 | 38 | 44 | 37 | 39 | 33 | 46 | 39 | 53 | 45 |
| 90 | 15 | 14 | 13 | 17 | 7 | 18 | 15 | 14 | 22 | 21 | 21 | 21 |
| 91 | 11 | 10 | 12 | 8 | 7 | 7 | 12 | 12 | 7 | 12 | 12 | 11 |
| 92 |  |  |  |  |  |  |  |  |  |  |  |  |
| 93 |  |  |  |  |  |  |  |  |  |  |  |  |
| 94 | 12 | 7 | 3 | 1 | 2 | 6 | 2 | 7 | 6 | 6 | 1 | 4 |
| 95 | 6 | 4 | 8 | 4 | 6 | 10 | 10 | 2 | 5 | 5 | 3 | 5 |
| 96 | 0 | 0 | 2 | 3 | 0 | 1 | 1 | 4 | 2 | 1 | 3 | 0 |
| 97 |  |  |  |  |  |  |  |  |  |  |  |  |
| 98 | 11 | 13 | 16 | 11 | 12 | 8 | 15 | 18 | 12 | 15 | 12 | 16 |
| 99 | 10 | 11 | 9 | 5 | 1 | 12 | 11 | 9 | 11 | 10 | 9 | 8 |
| 100 | 5 | 7 | 4 | 10 | 7 | 6 | 8 | 10 | 10 | 5 | 7 | 5 |
| 101 | 27 | 17 | 23 | 17 | 28 | 20 | 25 | 16 | 22 | 25 | 19 | 17 |
| 102 |  |  |  |  |  |  |  |  |  |  |  |  |
| 103 | 28 | 17 | 17 | 12 | 18 | 14 | 15 | 18 | 26 | 34 | 26 | 21 |
| 104 | 12 | 21 | 23 | 17 | 17 | 22 | 22 | 22 | 21 | 30 | 18 | 19 |
| 105 | 10 | 20 | 11 | 16 | 9 | 14 | 11 | 9 | 10 | 22 | 14 | 12 |

Table 33. Number of newly diagnosed stage I breast cancer during each month in 2019.

|  | Month | | | | | | | | | | | |
| --- | --- | --- | --- | --- | --- | --- | --- | --- | --- | --- | --- | --- |
| Hospital | 1 | 2 | 3 | 4 | 5 | 6 | 7 | 8 | 9 | 10 | 11 | 12 |
| 1 | 0 | 0 | 0 | 0 | 1 | 1 | 1 | 0 | 0 | 0 | 0 | 0 |
| 2 | 64 | 54 | 57 | 69 | 63 | 55 | 54 | 58 | 41 | 56 | 62 | 59 |
| 3 | 2 | 1 | 1 | 3 | 1 | 2 | 2 | 5 | 3 | 2 | 1 | 0 |
| 4 | 14 | 17 | 18 | 17 | 7 | 12 | 21 | 18 | 11 | 15 | 13 | 15 |
| 5 | 2 | 0 | 2 | 0 | 0 | 0 | 5 | 3 | 1 | 3 | 2 | 2 |
| 6 | 6 | 6 | 3 | 8 | 12 | 11 | 9 | 5 | 7 | 13 | 7 | 6 |
| 7 | 5 | 6 | 4 | 5 | 3 | 0 | 8 | 10 | 8 | 3 | 6 | 4 |
| 8 |  |  |  |  |  |  |  |  |  |  |  |  |
| 9 | 3 | 3 | 4 | 5 | 1 | 2 | 10 | 9 | 5 | 8 | 5 | 3 |
| 10 | 5 | 3 | 6 | 10 | 12 | 11 | 2 | 12 | 12 | 11 | 11 | 9 |
| 11 | 6 | 5 | 4 | 7 | 8 | 9 | 5 | 2 | 7 | 12 | 5 | 3 |
| 12 | 0 | 1 | 3 | 0 | 1 | 1 | 3 | 3 | 0 | 2 | 3 | 5 |
| 13 | 14 | 11 | 11 | 13 | 10 | 7 | 9 | 11 | 15 | 17 | 7 | 20 |
| 14 | 5 | 4 | 3 | 2 | 5 | 2 | 6 | 4 | 4 | 9 | 2 | 5 |
| 15 | 12 | 10 | 6 | 14 | 10 | 5 | 13 | 7 | 15 | 17 | 10 | 11 |
| 16 | 10 | 9 | 15 | 10 | 10 | 8 | 18 | 18 | 13 | 19 | 9 | 6 |
| 17 | 1 | 1 | 3 | 1 | 2 | 2 | 1 | 2 | 1 | 0 | 1 | 0 |
| 18 | 3 | 9 | 3 | 5 | 3 | 6 | 1 | 7 | 6 | 9 | 3 | 5 |
| 19 | 4 | 3 | 2 | 5 | 5 | 4 | 3 | 4 | 4 | 6 | 4 | 1 |
| 20 | 22 | 32 | 24 | 17 | 21 | 16 | 21 | 21 | 24 | 23 | 22 | 22 |
| 21 | 11 | 9 | 5 | 6 | 6 | 6 | 6 | 6 | 8 | 9 | 6 | 12 |
| 22 | 9 | 16 | 9 | 9 | 8 | 10 | 10 | 14 | 9 | 8 | 9 | 8 |
| 23 |  |  |  |  |  |  |  |  |  |  |  |  |
| 24 | 7 | 5 | 2 | 8 | 9 | 9 | 6 | 11 | 10 | 11 | 10 | 9 |
| 25 | 6 | 4 | 6 | 6 | 11 | 4 | 10 | 8 | 13 | 5 | 5 | 6 |
| 26 | 7 | 6 | 13 | 8 | 4 | 6 | 7 | 3 | 4 | 11 | 4 | 1 |
| 27 | 14 | 11 | 8 | 13 | 10 | 14 | 10 | 9 | 13 | 16 | 16 | 9 |
| 28 | 6 | 5 | 1 | 4 | 6 | 2 | 8 | 3 | 4 | 3 | 1 | 2 |
| 29 |  |  |  |  |  |  |  |  |  |  |  |  |
| 30 | 1 | 2 | 2 | 5 | 6 | 2 | 2 | 6 | 3 | 3 | 1 | 4 |
| 31 | 9 | 12 | 4 | 9 | 3 | 5 | 6 | 7 | 4 | 10 | 5 | 9 |
| 32 | 5 | 4 | 12 | 14 | 6 | 5 | 11 | 7 | 6 | 5 | 14 | 8 |
| 33 | 1 | 2 | 4 | 2 | 3 | 0 | 3 | 3 | 2 | 2 | 4 | 4 |
| 34 | 7 | 4 | 6 | 8 | 4 | 6 | 9 | 6 | 7 | 7 | 5 | 8 |
| 35 | 2 | 2 | 2 | 3 | 2 | 2 | 5 | 4 | 5 | 3 | 5 | 1 |
| 36 | 10 | 5 | 1 | 6 | 5 | 4 | 4 | 7 | 7 | 11 | 5 | 11 |
| 37 | 15 | 14 | 14 | 13 | 14 | 11 | 17 | 18 | 14 | 15 | 11 | 17 |
| 38 |  |  |  |  |  |  |  |  |  |  |  |  |
| 39 | 1 | 1 |  | 2 | 2 | 1 | 1 | 2 | 2 |  | 2 |  |
| 40 | 11 | 6 | 9 | 18 | 11 | 10 | 14 | 12 | 13 | 17 | 14 | 22 |
| 41 | 6 | 2 | 7 | 6 | 10 | 5 | 6 | 6 | 3 | 4 | 4 | 7 |
| 42 | 3 | 2 | 4 | 3 | 6 | 8 | 7 | 6 | 3 | 6 | 7 | 9 |
| 43 | 4 | 6 | 1 | 3 | 5 | 5 | 5 | 3 | 5 | 5 | 5 | 11 |
| 44 | 1 | 1 | 0 | 1 | 2 | 1 | 2 | 2 | 4 | 2 | 2 | 2 |
| 45 | 5 | 3 | 7 | 4 | 7 | 10 | 11 | 6 | 5 | 4 | 12 | 7 |
| 46 | 6 | 11 | 12 | 11 | 8 | 5 | 15 | 12 | 16 | 13 | 5 | 9 |
| 47 |  |  |  |  |  |  |  |  |  |  |  |  |
| 48 | 4 | 4 | 1 | 6 | 1 | 1 | 3 | 5 | 3 | 3 | 4 | 5 |
| 49 | 3 | 3 | 4 | 3 | 3 | 4 | 8 | 5 | 4 | 10 | 10 | 7 |
| 50 | 0 | 0 | 1 | 0 | 0 | 0 | 1 | 0 | 1 | 0 | 0 | 1 |
| 51 | 7 | 3 | 10 | 4 | 8 | 5 | 7 | 5 | 6 | 7 | 4 | 6 |
| 52 | 8 | 4 | 3 | 5 | 6 | 9 | 7 | 11 | 5 | 8 | 9 | 5 |
| 53 | 14 | 4 | 7 | 6 | 5 | 5 | 11 | 10 | 10 | 15 | 10 | 15 |
| 54 | 13 | 16 | 12 | 17 | 15 | 15 | 17 | 23 | 12 | 27 | 22 | 13 |
| 55 | 3 | 1 | 11 | 6 | 3 | 2 | 8 | 6 | 7 | 2 | 2 | 4 |
| 56 | 15 | 19 | 13 | 15 | 12 | 15 | 17 | 13 | 16 | 20 | 20 | 21 |
| 57 | 25 | 18 | 18 | 21 | 29 | 19 | 23 | 19 | 22 | 30 | 25 | 27 |
| 58 | 5 | 5 | 5 | 3 | 3 | 5 | 2 | 0 | 7 | 5 | 7 | 3 |
| 59 | 1 | 1 | 4 | 7 | 5 | 5 | 3 | 2 | 2 | 2 | 0 | 1 |
| 60 | 8 | 4 | 6 | 3 | 3 | 8 | 6 | 4 | 7 | 11 | 5 | 8 |
| 61 | 1 | 3 | 3 | 5 | 4 | 5 | 4 | 5 | 8 | 4 | 8 | 2 |
| 62 |  |  |  |  |  |  |  |  |  |  |  |  |
| 63 | 2 | 1 | 1 | 1 |  | 3 | 2 | 1 | 1 | 3 | 2 |  |
| 64 | 7 | 12 | 7 | 13 | 6 | 5 | 17 | 12 | 5 | 10 | 7 | 16 |
| 65 | 3 | 6 | 4 | 7 | 6 | 6 | 10 | 12 | 8 | 7 | 10 | 9 |
| 66 | 1 | 4 | 2 | 3 | 3 | 1 | 1 | 1 | 3 | 1 | 2 | 1 |
| 67 | 5 | 5 | 5 | 8 | 6 | 6 | 6 | 3 | 6 | 5 | 5 | 9 |
| 68 | 12 | 5 | 9 | 6 | 6 | 4 | 11 | 8 | 4 | 11 | 11 | 10 |
| 68 | 6 | 6 | 11 | 3 | 9 | 8 | 7 | 5 | 9 | 5 | 6 | 6 |
| 70 | 3 | 1 | 3 | 2 | 1 |  | 4 | 1 | 1 | 3 |  | 2 |
| 71 | 5 | 12 | 6 | 5 | 7 | 11 | 9 | 14 | 8 | 9 | 9 | 10 |
| 72 | 5 | 10 | 14 | 7 | 5 | 9 | 8 | 3 | 10 | 6 | 6 | 14 |
| 73 |  |  |  |  |  |  |  |  |  |  |  |  |
| 74 | 20 | 16 | 23 | 15 | 18 | 11 | 19 | 18 | 18 | 22 | 12 | 17 |
| 75 | 1 |  | 3 | 1 | 1 | 2 | 1 | 2 | 1 |  |  |  |
| 76 | 9 | 16 | 17 | 9 | 15 | 8 | 11 | 16 | 10 | 15 | 16 | 17 |
| 77 |  |  |  |  | 1 |  | 3 |  |  | 1 |  |  |
| 78 |  |  | 2 | 1 |  |  |  |  |  |  |  |  |
| 79 | 18 | 19 | 11 | 10 | 13 | 10 | 12 | 9 | 9 | 13 | 18 | 14 |
| 80 | 8 | 3 | 6 | 13 | 3 | 6 | 11 | 13 | 10 | 7 | 4 | 7 |
| 81 | 6 | 1 | 2 | 2 | 2 | 5 | 3 | 3 | 3 | 4 | 6 | 9 |
| 82 |  |  |  |  |  |  |  |  |  |  |  |  |
| 83 | 2 | 3 | 1 | 1 | 1 | 4 | 4 | 3 | 4 | 6 | 2 | 4 |
| 84 | 15 | 7 | 14 | 6 | 9 | 11 | 8 | 10 | 13 | 13 | 16 | 13 |
| 85 | 7 | 15 | 15 | 10 | 14 | 6 | 13 | 10 | 9 | 12 | 17 | 11 |
| 86 | 3 | 5 | 4 | 4 | 8 | 2 | 4 | 4 | 4 | 5 | 6 | 6 |
| 87 | 3 | 5 | 1 | 4 |  | 4 | 7 | 1 | 3 | 5 | 3 | 5 |
| 88 | 5 | 4 | 2 | 7 | 8 | 8 | 8 | 8 | 6 | 4 | 4 | 4 |
| 89 | 16 | 16 | 16 | 23 | 26 | 18 | 15 | 15 | 22 | 24 | 20 | 24 |
| 90 | 10 | 11 | 5 | 10 | 4 | 8 | 11 | 6 | 7 | 6 | 12 | 4 |
| 91 | 4 | 5 | 3 | 6 | 1 | 3 | 4 | 1 | 3 | 3 | 3 | 1 |
| 92 |  |  |  |  |  |  |  |  |  |  |  |  |
| 93 | 3 | 3 | 3 | 7 | 2 | 0 | 2 | 6 | 4 | 7 | 6 | 3 |
| 94 | 1 | 2 | 2 | 2 | 1 | 0 | 0 | 7 | 5 | 2 | 4 | 0 |
| 95 | 3 | 0 | 2 | 3 | 1 | 5 | 3 | 3 | 4 | 4 | 6 | 1 |
| 96 |  |  |  | 1 |  |  |  | 1 |  | 2 |  |  |
| 97 |  |  |  |  |  |  |  |  |  |  |  |  |
| 98 | 6 | 8 | 3 | 9 | 7 | 5 | 6 | 8 | 4 | 0 | 6 | 8 |
| 99 | 2 | 5 | 2 | 2 | 3 | 2 | 2 | 3 | 4 | 1 | 4 | 3 |
| 100 | 5 | 5 | 8 | 9 | 7 | 10 | 9 | 8 | 6 | 8 | 4 | 12 |
| 101 | 11 | 10 | 7 | 12 | 2 | 7 | 10 | 10 | 7 | 9 | 13 | 13 |
| 102 |  |  |  |  |  |  |  |  |  |  |  |  |
| 103 | 6 | 13 | 8 | 4 | 10 | 11 | 18 | 13 | 9 | 6 | 9 | 4 |
| 104 | 6 | 11 | 5 | 6 | 9 | 6 | 12 | 1 | 1 | 8 | 4 | 6 |
| 105 | 8 | 9 | 10 | 4 | 9 | 4 | 4 | 6 | 8 | 7 | 6 | 8 |

Table 34. Number of newly diagnosed stage I breast cancer during each month in 2020.

|  | Month | | | | | | | | | | | |
| --- | --- | --- | --- | --- | --- | --- | --- | --- | --- | --- | --- | --- |
| Hospital | 1 | 2 | 3 | 4 | 5 | 6 | 7 | 8 | 9 | 10 | 11 | 12 |
| 1 | 0 | 3 | 1 | 1 | 0 | 0 | 1 | 0 | 2 | 0 | 1 | 1 |
| 2 | 57 | 50 | 49 | 57 | 39 | 40 | 33 | 39 | 50 | 41 | 46 | 66 |
| 3 | 1 | 2 | 2 | 1 | 0 | 2 | 1 | 0 | 3 | 1 | 1 | 3 |
| 4 | 16 | 12 | 14 | 12 | 9 | 5 | 6 | 16 | 17 | 10 | 11 | 12 |
| 5 | 2 | 1 | 3 | 1 | 3 | 3 | 3 | 1 | 3 | 1 | 1 | 1 |
| 6 | 4 | 6 | 7 | 5 | 3 | 7 | 6 | 4 | 4 | 7 | 8 | 5 |
| 7 | 4 | 3 | 2 | 1 | 4 | 7 | 4 | 4 | 6 | 4 | 5 | 1 |
| 8 |  |  |  |  |  |  |  |  |  |  |  |  |
| 9 | 2 | 4 | 1 | 7 | 3 | 9 | 3 | 2 | 6 | 4 | 4 | 4 |
| 10 | 5 | 7 | 7 | 5 | 2 | 9 | 10 | 8 | 7 | 13 | 8 | 9 |
| 11 | 4 | 4 | 11 | 10 | 4 | 7 | 5 | 7 | 4 | 5 | 6 | 8 |
| 12 | 1 | 2 | 2 | 1 | 0 | 3 | 4 | 1 | 3 | 2 | 0 | 1 |
| 13 | 12 | 15 | 17 | 13 | 8 | 9 | 9 | 9 | 7 | 16 | 8 | 14 |
| 14 | 2 | 0 | 3 | 7 | 5 | 2 | 6 | 6 | 2 | 6 | 9 | 8 |
| 15 | 10 | 14 | 13 | 5 | 6 | 15 | 9 | 7 | 7 | 12 | 11 | 19 |
| 16 | 13 | 13 | 5 | 18 | 9 | 21 | 17 | 11 | 18 | 9 | 11 | 13 |
| 17 | 4 | 2 | 1 | 3 | 1 | 1 | 1 | 2 | 0 | 3 | 3 | 1 |
| 18 | 2 | 3 | 3 | 2 | 4 | 3 | 5 | 6 | 3 | 7 | 5 | 3 |
| 19 | 3 | 3 | 2 | 1 | 2 | 1 | 1 | 5 | 5 | 3 | 4 | 5 |
| 20 | 34 | 24 | 20 | 8 | 15 | 18 | 20 | 17 | 21 | 33 | 28 | 33 |
| 21 | 8 | 3 | 7 | 5 | 3 | 1 | 5 | 3 | 6 | 6 | 6 | 8 |
| 22 | 8 | 10 | 8 | 9 | 9 | 11 | 10 | 5 | 10 | 9 | 8 | 13 |
| 23 |  |  |  |  |  |  |  |  |  |  |  |  |
| 24 | 12 | 8 | 7 | 6 | 3 | 6 | 4 | 5 | 8 | 7 | 2 | 5 |
| 25 | 4 | 9 | 7 | 13 | 12 | 9 | 14 | 12 | 6 | 16 | 9 | 13 |
| 26 | 4 | 3 | 7 | 6 | 5 | 6 | 1 | 6 | 4 | 4 | 6 | 7 |
| 27 | 13 | 16 | 10 | 7 | 10 | 11 | 6 | 8 | 6 | 10 | 11 | 13 |
| 28 | 2 | 1 | 5 | 5 | 2 | 2 | 6 | 2 | 4 | 1 | 3 | 5 |
| 29 |  |  |  |  |  |  |  |  |  |  |  |  |
| 30 | 2 | 2 | 8 | 1 | 4 | 3 | 2 | 3 | 3 | 3 | 3 | 8 |
| 31 | 6 | 11 | 10 | 5 | 6 | 8 | 6 | 8 | 4 | 15 | 6 | 7 |
| 32 | 3 | 7 | 8 | 7 | 10 | 9 | 5 | 5 | 4 | 13 | 9 | 12 |
| 33 | 0 | 2 | 1 | 3 | 3 | 1 | 1 | 1 | 4 | 5 | 1 | 1 |
| 34 | 6 | 10 | 8 | 0 | 1 | 1 | 4 | 5 | 7 | 8 | 4 | 9 |
| 35 | 4 | 0 | 2 | 2 | 2 | 3 | 3 | 2 | 7 | 2 | 8 | 1 |
| 36 | 7 | 8 | 9 | 2 | 8 | 4 | 7 | 1 | 3 | 7 | 4 | 5 |
| 37 | 15 | 8 | 15 | 9 | 18 | 13 | 12 | 9 | 10 | 7 | 11 | 10 |
| 38 |  |  |  |  |  |  |  |  |  |  |  |  |
| 39 | 1 | 1 |  |  |  |  | 1 | 1 | 2 | 2 | 2 |  |
| 40 | 26 | 6 | 12 | 17 | 10 | 13 | 12 | 9 | 12 | 15 | 11 | 12 |
| 41 | 6 | 4 | 7 | 14 | 1 | 3 | 9 | 3 | 8 | 5 | 5 | 6 |
| 42 | 8 | 5 | 10 | 8 | 5 | 6 | 7 | 5 | 0 | 3 | 1 | 3 |
| 43 | 7 | 4 | 3 | 2 | 1 | 2 | 5 | 7 | 1 | 6 | 7 | 4 |
| 44 | 3 | 0 | 1 | 0 | 1 | 0 | 0 | 3 | 0 | 3 | 1 | 1 |
| 45 | 3 | 7 | 5 | 7 | 5 | 7 | 6 | 8 | 8 | 6 | 7 | 4 |
| 46 | 6 | 6 | 6 | 9 | 6 | 1 | 8 | 6 | 7 | 9 | 12 | 9 |
| 47 |  |  |  |  |  |  |  |  |  |  |  |  |
| 48 | 5 | 4 | 6 | 3 | 3 | 3 | 4 | 6 | 2 | 7 | 5 | 1 |
| 49 | 2 | 7 | 5 | 5 | 3 | 5 | 2 | 4 | 3 | 10 | 6 | 1 |
| 50 | 0 | 0 | 0 | 1 | 0 | 1 | 0 | 3 | 0 | 2 | 0 | 2 |
| 51 | 7 | 3 | 6 | 6 | 1 | 7 | 9 | 6 | 8 | 14 | 7 | 5 |
| 52 | 8 | 6 | 10 | 10 | 7 | 6 | 4 | 10 | 10 | 10 | 7 | 9 |
| 53 | 8 | 6 | 14 | 6 | 2 | 3 | 6 | 3 | 10 | 5 | 2 | 12 |
| 54 | 16 | 17 | 21 | 18 | 11 | 9 | 16 | 19 | 20 | 18 | 20 | 22 |
| 55 | 1 | 3 | 5 | 2 | 2 | 3 | 5 | 4 | 0 | 5 | 1 | 3 |
| 56 | 14 | 14 | 15 | 11 | 5 | 12 | 13 | 16 | 16 | 27 | 19 | 11 |
| 57 | 30 | 28 | 20 | 13 | 7 | 24 | 15 | 14 | 23 | 29 | 21 | 17 |
| 58 | 6 | 2 | 3 | 3 | 1 | 1 | 4 | 5 | 8 | 6 | 9 | 6 |
| 59 | 2 | 3 | 2 | 3 | 0 | 2 | 2 | 1 | 3 | 3 | 3 | 1 |
| 60 | 5 | 6 | 11 | 4 | 3 | 4 | 6 | 1 | 0 | 6 | 7 | 8 |
| 61 | 6 | 4 | 4 | 5 | 4 | 6 | 1 | 1 | 1 | 8 | 4 | 4 |
| 62 |  |  |  |  |  |  |  |  |  |  |  |  |
| 63 |  |  | 1 |  |  | 1 | 2 | 2 | 2 |  |  | 1 |
| 64 | 12 | 12 | 12 | 7 | 10 | 4 | 10 | 7 | 11 | 17 | 7 | 5 |
| 65 | 4 | 6 | 4 | 12 | 14 | 11 | 7 | 9 | 4 | 5 | 6 | 6 |
| 66 | 0 | 2 | 4 | 1 | 1 | 1 | 1 | 3 | 2 | 3 | 0 | 2 |
| 67 | 4 | 11 | 2 | 5 | 4 | 0 | 4 | 7 | 5 | 4 | 5 | 1 |
| 68 | 5 | 16 | 4 | 8 | 4 | 10 | 6 | 5 | 7 | 10 | 5 | 8 |
| 68 | 8 | 3 | 2 | 0 | 1 | 2 | 1 | 3 | 0 | 7 | 2 | 1 |
| 70 | 1 | 4 |  | 1 |  |  | 2 |  | 1 | 1 | 4 | 2 |
| 71 | 14 | 7 | 6 | 8 | 9 | 3 | 9 | 17 | 6 | 7 | 7 | 9 |
| 72 | 15 | 9 | 6 | 7 | 4 | 6 | 10 | 5 | 11 | 13 | 9 | 10 |
| 73 |  |  |  |  |  |  |  |  |  |  |  |  |
| 74 | 19 | 18 | 19 | 17 | 14 | 15 | 15 | 13 | 21 | 6 | 10 | 26 |
| 75 |  | 1 |  |  |  |  |  |  |  |  |  |  |
| 76 | 11 | 15 | 5 | 6 | 12 | 14 | 5 | 11 | 11 | 10 | 14 | 16 |
| 77 |  |  | 2 | 1 |  |  |  |  |  | 1 |  | 1 |
| 78 | 3 |  | 1 | 1 | 1 |  |  | 2 |  |  | 1 |  |
| 79 | 8 | 4 | 12 | 5 | 5 | 11 | 15 | 8 | 14 | 8 | 20 | 10 |
| 80 | 11 | 4 | 3 | 5 | 3 | 9 | 17 | 8 | 7 | 9 | 7 | 12 |
| 81 | 2 | 7 | 8 | 2 | 3 | 6 | 3 | 1 | 4 | 2 | 6 | 1 |
| 82 |  |  |  |  |  |  |  |  |  |  |  |  |
| 83 | 5 | 2 | 3 | 4 | 5 | 0 | 5 | 1 | 1 | 1 | 6 | 4 |
| 84 | 12 | 5 | 9 | 9 | 12 | 10 | 10 | 10 | 14 | 9 | 14 | 13 |
| 85 | 10 | 4 | 20 | 10 | 7 | 11 | 11 | 7 | 9 | 16 | 10 | 9 |
| 86 | 7 | 9 | 6 | 7 | 1 | 10 | 6 | 6 | 5 | 9 | 6 | 6 |
| 87 | 3 | 5 | 4 | 5 | 3 | 2 |  | 1 | 2 | 1 | 1 | 2 |
| 88 | 3 | 7 | 4 | 4 | 1 | 1 | 7 | 3 | 1 | 7 | 4 | 1 |
| 89 | 29 | 14 | 21 | 17 | 13 | 15 | 16 | 15 | 19 | 15 | 25 | 17 |
| 90 | 6 | 6 | 6 | 4 | 3 | 7 | 4 | 8 | 9 | 7 | 13 | 8 |
| 91 | 5 | 3 | 3 | 2 | 3 | 0 | 1 | 5 | 1 | 2 | 3 | 6 |
| 92 |  |  |  |  |  |  |  |  |  |  |  |  |
| 93 | 4 | 4 | 3 | 1 | 1 | 3 | 8 | 2 | 6 | 9 | 3 | 4 |
| 94 | 7 | 3 | 1 | 0 | 1 | 2 | 1 | 2 | 2 | 2 | 0 | 4 |
| 95 | 4 | 1 | 4 | 1 | 3 | 6 | 4 | 0 | 1 | 2 | 1 | 3 |
| 96 |  |  |  | 1 |  |  | 1 | 4 | 1 | 1 | 2 |  |
| 97 |  |  |  |  |  |  |  |  |  |  |  |  |
| 98 | 8 | 5 | 6 | 5 | 5 | 1 | 6 | 6 | 5 | 8 | 4 | 9 |
| 99 | 3 | 6 | 4 | 1 | 1 | 4 | 5 | 3 | 5 | 6 | 4 | 4 |
| 100 | 4 | 5 | 1 | 5 | 2 | 4 | 6 | 5 | 9 | 3 | 3 | 5 |
| 101 | 16 | 7 | 7 | 9 | 12 | 8 | 11 | 6 | 9 | 10 | 14 | 8 |
| 102 |  |  |  |  |  |  |  |  |  |  |  |  |
| 103 | 13 | 10 | 12 | 7 | 6 | 5 | 6 | 7 | 8 | 12 | 12 | 11 |
| 104 | 3 | 10 | 9 | 8 | 6 | 6 | 7 | 7 | 8 | 8 | 4 | 3 |
| 105 | 7 | 10 | 3 | 9 | 0 | 8 | 5 | 4 | 2 | 9 | 6 | 5 |

Table 35. Number of newly diagnosed stage II breast cancer during each month in 2019.

|  | Month | | | | | | | | | | | |
| --- | --- | --- | --- | --- | --- | --- | --- | --- | --- | --- | --- | --- |
| Hospital | 1 | 2 | 3 | 4 | 5 | 6 | 7 | 8 | 9 | 10 | 11 | 12 |
| 1 | 0 | 1 | 0 | 1 | 0 | 0 | 0 | 0 | 0 | 0 | 2 | 0 |
| 2 | 27 | 20 | 27 | 31 | 21 | 22 | 26 | 31 | 32 | 30 | 31 | 25 |
| 3 | 1 | 2 | 1 | 3 | 2 | 0 | 5 | 1 | 0 | 1 | 1 | 1 |
| 4 | 11 | 11 | 13 | 8 | 12 | 11 | 11 | 10 | 9 | 13 | 20 | 12 |
| 5 | 0 | 1 | 2 | 2 | 1 | 0 | 2 | 0 | 2 | 4 | 1 | 2 |
| 6 | 8 | 1 | 4 | 7 | 13 | 5 | 3 | 3 | 3 | 4 | 3 | 1 |
| 7 | 1 | 4 | 1 | 1 | 4 | 7 | 3 | 2 | 5 | 3 | 4 | 5 |
| 8 |  |  |  |  |  |  |  |  |  |  |  |  |
| 9 | 1 | 1 | 5 | 2 | 3 | 5 | 7 | 4 | 4 | 4 | 2 | 4 |
| 10 | 4 | 4 | 8 | 6 | 9 | 11 | 6 | 1 | 8 | 3 | 5 | 5 |
| 11 | 6 | 3 | 3 | 8 | 8 | 8 | 7 | 2 | 5 | 9 | 4 | 3 |
| 12 | 1 | 2 | 0 | 0 | 0 | 1 | 1 | 4 | 2 | 0 | 0 | 4 |
| 13 | 6 | 9 | 19 | 10 | 11 | 15 | 17 | 12 | 11 | 11 | 11 | 9 |
| 14 | 1 | 3 | 2 | 1 | 3 | 6 | 2 | 5 | 2 | 5 | 6 | 3 |
| 15 | 6 | 2 | 9 | 6 | 9 | 7 | 3 | 6 | 8 | 8 | 8 | 14 |
| 16 | 10 | 11 | 6 | 5 | 14 | 11 | 9 | 6 | 6 | 9 | 8 | 10 |
| 17 | 2 | 4 | 1 | 1 | 0 | 0 | 0 | 3 | 1 | 0 | 2 | 0 |
| 18 | 6 | 3 | 5 | 6 | 4 | 3 | 7 | 3 | 4 | 3 | 4 | 8 |
| 19 | 1 | 2 | 4 | 0 | 5 | 2 | 8 | 3 | 3 | 6 | 3 | 3 |
| 20 | 14 | 9 | 17 | 13 | 19 | 14 | 18 | 14 | 14 | 18 | 17 | 21 |
| 21 | 7 | 3 | 4 | 3 | 12 | 2 | 4 | 7 | 6 | 7 | 2 | 7 |
| 22 | 6 | 3 | 5 | 8 | 7 | 6 | 7 | 10 | 4 | 3 | 6 | 9 |
| 23 |  |  |  |  |  |  |  |  |  |  |  |  |
| 24 | 3 | 5 | 2 | 7 | 7 | 3 | 7 | 8 | 7 | 3 | 2 | 7 |
| 25 | 6 | 1 | 1 | 6 | 7 | 10 | 7 | 8 | 9 | 4 | 11 | 6 |
| 26 | 5 | 4 | 2 | 5 | 2 | 7 | 3 | 5 | 5 | 7 | 2 | 3 |
| 27 | 7 | 8 | 8 | 8 | 9 | 7 | 7 | 9 | 5 | 9 | 5 | 10 |
| 28 | 2 | 1 | 2 | 0 | 5 | 1 | 3 | 4 | 3 | 2 | 1 | 2 |
| 29 |  |  |  |  |  |  |  |  |  |  |  |  |
| 30 | 1 | 3 | 1 | 5 | 1 | 2 | 5 | 1 | 6 | 3 | 2 | 1 |
| 31 | 2 | 6 | 11 | 6 | 3 | 3 | 7 | 3 | 7 | 5 | 3 | 6 |
| 32 | 7 | 6 | 4 | 4 | 5 | 9 | 7 | 7 | 5 | 5 | 3 | 9 |
| 33 | 2 | 2 | 3 | 2 | 2 | 3 | 3 | 2 | 0 | 3 | 4 | 4 |
| 34 | 1 | 4 | 1 | 5 | 2 | 6 | 6 | 6 | 2 | 7 | 2 | 3 |
| 35 | 2 | 2 | 4 | 3 | 7 | 3 | 2 | 3 | 4 | 3 | 3 | 4 |
| 36 | 3 | 4 | 8 | 4 | 5 | 2 | 4 |  | 6 | 4 | 5 | 5 |
| 37 | 9 | 13 | 12 | 13 | 11 | 12 | 15 | 13 | 10 | 10 | 13 | 8 |
| 38 |  |  |  |  |  |  |  |  |  |  |  |  |
| 39 | 1 | 1 |  |  |  | 4 | 2 |  |  | 1 | 1 | 2 |
| 40 | 5 | 5 | 7 | 8 | 4 | 11 | 8 | 12 | 8 | 16 | 8 | 13 |
| 41 | 4 | 3 | 4 | 3 | 1 | 6 | 3 | 6 | 2 | 6 | 8 | 3 |
| 42 | 5 | 1 | 8 | 5 | 3 | 3 | 1 | 3 | 2 | 4 | 5 | 4 |
| 43 | 5 | 2 | 2 | 4 | 9 | 4 | 2 | 2 | 3 | 2 | 2 | 3 |
| 44 | 2 | 1 | 0 | 1 | 0 | 1 | 2 | 0 | 3 | 3 | 0 | 2 |
| 45 | 7 | 3 | 6 | 2 | 4 | 1 | 0 | 2 | 4 | 3 | 1 | 2 |
| 46 | 7 | 4 | 3 | 13 | 12 | 4 | 7 | 4 | 9 | 7 | 4 | 3 |
| 47 |  |  |  |  |  |  |  |  |  |  |  |  |
| 48 | 4 | 3 | 3 | 4 | 5 | 2 | 4 | 3 | 3 | 3 | 4 | 3 |
| 49 | 2 | 2 | 3 | 3 | 3 | 3 | 4 | 3 | 5 | 2 | 6 | 1 |
| 50 | 0 | 0 | 0 | 0 | 1 | 1 | 0 | 0 | 0 | 3 | 1 | 0 |
| 51 | 6 | 8 | 6 | 9 | 2 | 1 | 9 | 7 | 5 | 5 | 3 | 4 |
| 52 | 6 | 5 | 6 | 12 | 4 | 6 | 11 | 9 | 11 | 8 | 7 | 4 |
| 53 | 7 | 4 | 4 | 9 | 5 | 5 | 9 | 8 | 3 | 5 | 6 | 4 |
| 54 | 13 | 16 | 10 | 9 | 12 | 11 | 8 | 15 | 18 | 10 | 14 | 14 |
| 55 | 1 | 4 | 4 | 4 | 3 | 1 | 6 | 3 | 0 | 3 | 4 | 1 |
| 56 | 17 | 10 | 8 | 17 | 9 | 15 | 12 | 14 | 21 | 11 | 16 | 8 |
| 57 | 10 | 15 | 21 | 21 | 13 | 10 | 21 | 10 | 19 | 20 | 17 | 15 |
| 58 | 2 | 2 | 4 | 2 | 2 | 2 | 3 | 2 | 3 | 3 | 3 | 2 |
| 59 | 1 | 1 | 2 | 1 | 6 | 1 | 3 | 2 | 3 | 3 | 0 | 3 |
| 60 | 7 | 6 | 2 | 3 | 1 | 2 | 5 | 6 | 3 | 5 | 3 | 6 |
| 61 | 5 | 1 | 3 | 0 | 2 | 7 | 0 | 3 | 6 | 4 | 3 | 8 |
| 62 |  |  |  |  |  |  |  |  |  |  |  |  |
| 63 | 1 | 1 |  | 1 | 1 |  | 1 | 2 | 4 | 2 | 1 |  |
| 64 | 7 | 6 | 3 | 7 | 3 | 5 | 9 | 5 | 7 | 5 | 6 | 6 |
| 65 | 2 | 9 | 7 | 3 | 3 | 10 | 9 | 7 | 4 | 6 | 5 | 11 |
| 66 | 0 | 2 | 1 | 0 | 1 | 1 | 0 | 1 | 0 | 0 | 0 | 1 |
| 67 | 1 | 5 | 2 | 8 | 4 | 6 | 5 | 4 | 5 | 3 | 3 | 2 |
| 68 | 4 | 9 | 5 | 10 | 10 | 8 | 3 | 6 | 4 | 19 | 6 | 8 |
| 68 | 6 | 8 | 7 | 8 | 1 | 9 | 4 | 5 | 3 | 3 | 2 | 2 |
| 70 |  | 1 |  |  | 2 | 1 | 1 | 2 | 1 |  | 1 |  |
| 71 | 3 | 2 | 9 | 4 | 6 | 8 | 7 | 9 | 4 | 8 | 10 | 9 |
| 72 | 9 | 2 | 9 | 5 | 6 | 4 | 4 | 5 | 5 | 4 | 2 | 11 |
| 73 |  |  |  |  |  |  |  |  |  |  |  |  |
| 74 | 14 | 13 | 12 | 11 | 10 | 18 | 21 | 12 | 8 | 11 | 12 | 9 |
| 75 |  |  |  |  |  |  |  |  |  | 1 |  |  |
| 76 | 10 | 6 | 4 | 6 | 8 | 6 | 8 | 7 | 7 | 10 | 8 | 4 |
| 77 | 1 | 1 |  |  |  |  |  |  |  | 1 |  |  |
| 78 |  | 1 |  |  |  |  |  |  |  | 1 | 1 |  |
| 79 | 5 | 4 | 2 | 5 | 6 | 9 | 7 | 7 | 5 | 6 | 8 | 11 |
| 80 | 4 | 4 | 3 | 5 | 10 | 4 | 10 | 4 | 6 | 7 | 6 | 4 |
| 81 | 6 | 4 | 3 | 3 | 5 | 3 | 2 | 1 | 2 | 9 | 4 | 1 |
| 82 |  |  |  |  |  |  |  |  |  |  |  |  |
| 83 | 2 | 4 | 1 | 4 | 5 | 3 | 3 | 5 | 2 | 3 | 4 | 2 |
| 84 | 2 | 9 | 3 | 6 | 2 | 9 | 6 | 10 | 6 | 6 | 3 | 9 |
| 85 | 5 | 9 | 15 | 7 | 9 | 12 | 16 | 6 | 14 | 16 | 11 | 9 |
| 86 | 8 | 7 | 4 | 6 | 7 | 4 | 3 | 3 | 6 | 9 | 7 | 2 |
| 87 | 2 | 1 |  | 1 | 2 | 1 | 7 | 3 | 2 |  | 1 | 5 |
| 88 | 3 | 5 | 3 | 3 | 7 | 4 | 2 | 4 | 3 | 4 | 2 | 2 |
| 89 | 16 | 20 | 20 | 12 | 8 | 13 | 9 | 12 | 20 | 11 | 13 | 11 |
| 90 | 3 | 1 | 2 | 4 | 4 | 5 | 0 | 4 | 11 | 5 | 9 | 1 |
| 91 | 4 | 2 | 3 | 6 | 6 | 2 | 2 | 4 | 4 | 5 | 5 | 3 |
| 92 |  |  |  |  |  |  |  |  |  |  |  |  |
| 93 | 1 | 1 | 3 | 2 | 1 | 3 | 2 | 5 | 4 | 3 | 1 | 3 |
| 94 | 1 | 2 | 1 | 1 | 0 | 0 | 0 | 1 | 2 | 0 | 0 | 1 |
| 95 | 0 | 3 | 0 | 1 | 0 | 0 | 0 | 2 | 2 | 4 | 2 | 2 |
| 96 |  |  |  |  | 1 |  |  |  |  |  |  |  |
| 97 |  |  |  |  |  |  |  |  |  |  |  |  |
| 98 | 6 | 3 | 2 | 4 | 4 | 5 | 3 | 3 | 6 | 3 | 7 | 5 |
| 99 | 1 | 2 | 1 | 2 | 4 | 1 | 5 | 3 | 5 | 2 | 3 | 2 |
| 100 | 0 | 1 | 2 | 0 | 0 | 0 | 1 | 2 | 2 | 1 | 0 | 0 |
| 101 | 6 | 6 | 4 | 5 | 7 | 6 | 8 | 4 | 7 | 6 | 6 | 3 |
| 102 |  |  |  |  |  |  |  |  |  |  |  |  |
| 103 | 3 | 4 | 4 | 3 | 2 | 3 | 3 | 11 | 3 | 5 | 3 | 6 |
| 104 | 9 | 9 | 5 | 5 | 10 | 6 | 5 | 2 | 2 | 4 | 5 | 1 |
| 105 | 2 | 7 | 5 | 6 | 7 | 3 | 8 | 4 | 4 | 5 | 3 | 6 |

Table 36. Number of newly diagnosed stage II breast cancer during each month in 2020.

|  | Month | | | | | | | | | | | |
| --- | --- | --- | --- | --- | --- | --- | --- | --- | --- | --- | --- | --- |
| Hospital | 1 | 2 | 3 | 4 | 5 | 6 | 7 | 8 | 9 | 10 | 11 | 12 |
| 1 | 0 | 1 | 0 | 1 | 0 | 0 | 1 | 0 | 2 | 0 | 1 | 1 |
| 2 | 24 | 22 | 21 | 26 | 12 | 30 | 14 | 29 | 19 | 23 | 23 | 27 |
| 3 | 1 | 0 | 0 | 0 | 0 | 2 | 2 | 2 | 1 | 0 | 2 | 2 |
| 4 | 9 | 10 | 9 | 11 | 9 | 11 | 9 | 9 | 8 | 10 | 9 | 19 |
| 5 | 2 | 0 | 1 | 0 | 0 | 2 | 2 | 5 | 1 | 1 | 2 | 3 |
| 6 | 3 | 4 | 3 | 7 | 3 | 4 | 5 | 6 | 3 | 10 | 5 | 2 |
| 7 | 3 | 0 | 3 | 3 | 4 | 4 | 4 | 1 | 0 | 6 | 2 | 2 |
| 8 |  |  |  |  |  |  |  |  |  |  |  |  |
| 9 | 2 | 5 | 5 | 1 | 1 | 7 | 1 | 2 | 3 | 2 | 4 | 4 |
| 10 | 5 | 6 | 6 | 4 | 0 | 12 | 7 | 6 | 5 | 14 | 9 | 3 |
| 11 | 0 | 1 | 8 | 8 | 5 | 5 | 6 | 3 | 6 | 5 | 6 | 5 |
| 12 | 1 | 1 | 1 | 2 | 2 | 0 | 3 | 0 | 3 | 1 | 0 | 4 |
| 13 | 9 | 9 | 9 | 6 | 7 | 13 | 6 | 3 | 19 | 14 | 10 | 9 |
| 14 | 1 | 5 | 2 | 5 | 1 | 2 | 2 | 4 | 3 | 2 | 3 | 6 |
| 15 | 3 | 9 | 7 | 7 | 9 | 6 | 10 | 6 | 9 | 12 | 4 | 9 |
| 16 | 8 | 7 | 8 | 9 | 9 | 6 | 9 | 12 | 16 | 11 | 12 | 20 |
| 17 | 0 | 0 | 2 | 2 | 0 | 0 | 0 | 0 | 1 | 0 | 1 | 0 |
| 18 | 4 | 1 | 2 | 3 | 2 | 7 | 1 | 5 | 6 | 2 | 2 | 3 |
| 19 | 3 | 2 | 5 | 1 | 4 | 2 | 1 | 5 | 6 | 0 | 5 | 1 |
| 20 | 19 | 17 | 14 | 10 | 12 | 12 | 10 | 13 | 19 | 23 | 22 | 27 |
| 21 | 5 | 6 | 5 | 5 | 0 | 2 | 10 | 7 | 2 | 4 | 3 | 3 |
| 22 | 7 | 4 | 9 | 5 | 2 | 5 | 2 | 3 | 5 | 3 | 4 | 9 |
| 23 |  |  |  |  |  |  |  |  |  |  |  |  |
| 24 | 9 | 3 | 8 | 1 | 5 | 8 | 3 | 4 | 3 | 6 | 7 | 4 |
| 25 | 5 | 1 | 5 | 7 | 1 | 5 | 6 | 6 | 3 | 5 | 3 | 8 |
| 26 | 4 | 1 | 4 | 2 | 2 | 2 | 2 | 4 | 6 | 7 | 7 | 3 |
| 27 | 8 | 3 | 5 | 9 | 3 | 8 | 6 | 4 | 3 | 4 | 6 | 5 |
| 28 | 3 | 0 | 2 | 3 | 0 | 2 | 4 | 3 | 2 | 5 | 3 | 1 |
| 29 |  |  |  |  |  |  |  |  |  |  |  |  |
| 30 | 0 | 2 | 3 | 3 | 2 | 1 | 0 | 5 | 4 | 3 | 4 | 2 |
| 31 | 3 | 4 | 6 | 8 | 4 | 1 | 3 | 6 | 4 | 6 | 4 | 1 |
| 32 | 7 | 3 | 1 | 4 | 1 | 6 | 4 | 4 | 2 | 6 | 3 |  |
| 33 | 3 | 2 | 2 | 0 | 1 | 3 | 6 | 2 | 3 | 1 | 2 | 2 |
| 34 | 4 | 7 | 6 | 0 | 0 | 4 | 4 | 4 | 2 | 4 | 1 | 3 |
| 35 | 3 | 0 | 3 | 1 | 2 | 4 | 3 | 4 | 5 | 8 | 2 | 6 |
| 36 | 4 | 4 | 8 | 4 | 6 | 5 | 7 | 7 | 5 | 4 | 5 | 7 |
| 37 | 14 | 10 | 10 | 8 | 5 | 11 | 12 | 9 | 5 | 8 | 8 | 11 |
| 38 |  |  |  |  |  |  |  |  |  |  |  |  |
| 39 | 2 | 1 |  |  | 3 |  | 2 | 2 | 2 |  |  | 1 |
| 40 | 9 | 7 | 6 | 6 | 8 | 4 | 3 | 5 | 8 | 11 | 8 | 4 |
| 41 | 4 | 4 | 5 | 2 | 4 | 8 | 6 | 7 | 3 | 6 | 8 | 4 |
| 42 | 4 | 2 | 4 | 3 | 2 | 4 | 2 | 1 | 2 | 3 | 2 | 8 |
| 43 | 5 | 1 | 6 | 5 | 2 | 7 | 2 | 3 | 4 | 6 | 4 | 3 |
| 44 | 0 | 1 | 3 | 1 | 1 | 3 | 0 | 2 | 1 | 0 | 3 | 0 |
| 45 | 3 | 4 | 2 | 2 | 4 | 7 | 4 | 6 | 2 | 6 | 2 | 6 |
| 46 | 6 | 6 | 7 | 7 | 2 | 10 | 3 | 3 | 5 | 6 | 2 | 3 |
| 47 |  |  |  |  |  |  |  |  |  |  |  |  |
| 48 | 5 | 3 | 5 | 3 | 2 | 5 | 3 | 3 | 2 | 4 | 4 | 3 |
| 49 | 2 | 4 | 4 | 1 | 4 | 1 | 2 | 4 | 6 | 4 | 3 | 3 |
| 50 | 1 | 0 | 0 | 0 | 2 | 1 | 1 | 1 | 0 | 1 | 0 | 0 |
| 51 | 3 | 5 | 4 | 4 | 1 | 7 | 7 | 3 | 1 | 8 | 2 | 10 |
| 52 | 3 | 4 | 9 | 7 | 4 | 6 | 9 | 2 | 5 | 10 | 5 | 13 |
| 53 | 6 | 5 | 2 | 3 | 3 | 10 | 4 | 5 | 2 | 6 | 7 | 7 |
| 54 | 15 | 11 | 10 | 14 | 8 | 10 | 17 | 17 | 12 | 24 | 14 | 21 |
| 55 | 0 | 4 | 1 | 1 | 3 | 4 | 2 | 4 | 3 | 0 | 1 | 0 |
| 56 | 13 | 13 | 17 | 10 | 6 | 7 | 12 | 15 | 8 | 13 | 16 | 17 |
| 57 | 15 | 17 | 16 | 7 | 7 | 17 | 17 | 13 | 13 | 21 | 12 | 12 |
| 58 | 4 | 4 | 4 | 4 | 1 | 4 | 2 | 3 | 5 | 5 | 4 | 3 |
| 59 | 3 | 4 | 3 | 1 | 1 | 5 | 2 | 3 | 4 | 1 | 1 | 1 |
| 60 | 2 | 5 | 4 | 6 | 2 | 2 | 4 | 0 | 0 | 2 | 7 | 5 |
| 61 | 6 | 2 | 1 | 2 | 3 | 1 | 1 | 5 | 3 | 2 | 2 | 7 |
| 62 |  |  |  |  |  |  |  |  |  |  |  |  |
| 63 |  |  | 1 |  |  | 1 |  | 3 | 3 | 2 |  |  |
| 64 | 4 | 5 | 4 | 3 | 4 | 6 | 8 | 8 | 13 | 13 | 3 | 5 |
| 65 | 6 | 4 | 3 | 8 | 13 | 6 | 5 | 9 | 5 | 8 | 3 | 2 |
| 66 | 2 | 1 | 0 | 1 | 1 | 2 | 2 | 0 | 2 | 0 | 1 | 2 |
| 67 | 0 | 2 | 3 | 2 | 4 | 4 | 3 | 0 | 3 | 3 | 3 | 1 |
| 68 | 5 | 7 | 7 | 2 | 3 | 8 | 6 | 13 | 8 | 8 | 4 | 10 |
| 68 | 2 | 0 | 0 | 2 | 0 | 1 | 0 | 5 | 2 | 0 | 0 | 0 |
| 70 |  | 1 | 5 |  |  | 2 | 1 | 2 | 1 | 1 | 1 |  |
| 71 | 11 | 6 | 6 | 3 | 1 | 8 | 7 | 7 | 6 | 7 | 6 | 8 |
| 72 | 8 | 5 | 11 | 5 | 4 | 8 | 4 | 6 | 5 | 6 | 8 | 6 |
| 73 |  |  |  |  |  |  |  |  |  |  |  |  |
| 74 | 15 | 13 | 14 | 10 | 7 | 12 | 12 | 18 | 12 | 11 | 13 | 14 |
| 75 |  |  |  |  |  |  | 2 |  |  |  |  |  |
| 76 | 13 | 5 | 7 | 5 | 8 | 7 | 5 | 7 | 9 | 7 | 8 | 11 |
| 77 | 1 |  |  |  |  | 2 | 1 |  |  | 3 |  |  |
| 78 |  | 1 | 1 |  | 1 |  |  |  |  | 2 | 1 |  |
| 79 | 7 | 6 | 9 | 3 | 4 | 7 | 9 | 7 | 6 | 8 | 10 | 7 |
| 80 | 4 | 3 | 3 | 2 | 4 | 4 | 6 | 4 | 3 | 9 | 10 | 3 |
| 81 | 6 | 2 | 1 | 2 | 3 | 3 | 1 | 3 | 3 | 6 | 2 | 3 |
| 82 |  |  |  |  |  |  |  |  |  |  |  |  |
| 83 | 4 | 2 | 1 | 4 | 0 | 3 | 2 | 2 | 6 | 4 | 2 | 3 |
| 84 | 9 | 4 | 6 | 10 | 1 | 7 | 6 | 6 | 3 | 11 | 8 | 6 |
| 85 | 8 | 7 | 11 | 4 | 3 | 11 | 15 | 17 | 13 | 9 | 18 | 9 |
| 86 | 3 | 5 | 2 | 8 | 2 | 11 | 4 | 4 | 2 | 7 | 3 | 4 |
| 87 | 5 | 1 | 3 | 3 | 1 | 1 | 6 | 3 | 1 |  | 2 | 2 |
| 88 | 0 | 0 | 0 | 0 | 2 | 6 | 2 | 0 | 0 | 0 | 1 | 0 |
| 89 | 11 | 18 | 18 | 15 | 17 | 12 | 18 | 14 | 21 | 14 | 16 | 18 |
| 90 | 4 | 3 | 4 | 3 | 3 | 7 | 5 | 2 | 6 | 3 | 3 | 7 |
| 91 | 3 | 4 | 6 | 1 | 0 | 5 | 7 | 2 | 2 | 7 | 2 | 1 |
| 92 |  |  |  |  |  |  |  |  |  |  |  |  |
| 93 | 2 | 1 | 5 | 4 | 0 | 4 | 2 | 5 | 4 | 2 | 2 | 3 |
| 94 | 3 | 2 | 1 | 0 | 1 | 1 | 1 | 3 | 1 | 3 | 0 | 0 |
| 95 | 1 | 2 | 2 | 2 | 2 | 2 | 2 | 1 | 2 | 1 | 0 | 1 |
| 96 |  |  | 1 |  |  | 1 |  |  |  |  |  |  |
| 97 |  |  |  |  |  |  |  |  |  |  |  |  |
| 98 | 2 | 3 | 5 | 3 | 2 | 6 | 6 | 8 | 7 | 4 | 6 | 4 |
| 99 | 4 | 4 | 3 | 1 | 0 | 3 | 4 | 5 | 2 | 0 | 4 | 4 |
| 100 | 0 | 0 | 0 | 0 | 1 | 1 | 1 | 3 | 0 | 1 | 1 | 0 |
| 101 | 1 | 4 | 7 | 2 | 5 | 3 | 3 | 4 | 4 | 6 | 3 | 2 |
| 102 |  |  |  |  |  |  |  |  |  |  |  |  |
| 103 | 7 | 3 | 2 | 3 | 3 | 1 | 5 | 6 | 6 | 8 | 7 | 4 |
| 104 | 4 | 4 | 8 | 2 | 2 | 7 | 7 | 8 | 6 | 10 | 8 | 5 |
| 105 | 1 | 9 | 4 | 5 | 5 | 4 | 5 | 4 | 7 | 9 | 7 | 6 |

Table 37. Number of newly diagnosed stage III breast cancer during each month in 2019.

|  | Month | | | | | | | | | | | |
| --- | --- | --- | --- | --- | --- | --- | --- | --- | --- | --- | --- | --- |
| Hospital | 1 | 2 | 3 | 4 | 5 | 6 | 7 | 8 | 9 | 10 | 11 | 12 |
| 1 | 0 | 0 | 0 | 0 | 0 | 0 | 1 | 0 | 0 | 0 | 0 | 0 |
| 2 | 12 | 6 | 5 | 7 | 12 | 8 | 8 | 7 | 7 | 12 | 9 | 7 |
| 3 | 0 | 1 | 2 | 0 | 0 | 0 | 1 | 0 | 1 | 1 | 0 | 2 |
| 4 | 0 | 5 | 1 | 1 | 1 | 0 | 2 | 0 | 1 | 5 | 3 | 2 |
| 5 | 0 | 2 | 2 | 1 | 0 | 0 | 0 | 1 | 0 | 0 | 0 | 1 |
| 6 |  | 1 | 2 | 2 | 4 | 3 | 2 | 3 | 1 | 1 | 2 | 1 |
| 7 | 0 | 0 | 1 | 0 | 0 | 0 | 0 | 2 | 0 | 0 | 0 | 2 |
| 8 |  |  |  |  |  |  |  |  |  |  |  |  |
| 9 | 0 | 0 | 1 | 0 | 1 | 0 | 1 | 0 | 1 | 2 | 0 | 1 |
| 10 | 1 | 1 | 1 | 4 | 3 | 3 | 0 | 4 | 3 | 0 | 2 | 1 |
| 11 | 5 | 0 | 1 | 3 | 1 | 1 | 1 | 1 | 1 | 3 | 1 | 3 |
| 12 | 1 | 0 | 1 | 0 | 0 | 1 | 0 | 0 | 0 | 0 | 0 | 1 |
| 13 | 1 | 4 | 5 | 0 | 4 | 3 | 3 | 2 | 3 | 3 | 2 | 3 |
| 14 | 0 | 1 | 2 | 0 | 2 | 2 | 1 | 2 | 1 | 1 | 2 | 2 |
| 15 | 4 | 2 | 1 | 3 | 1 | 4 | 2 | 3 | 3 | 4 | 1 | 1 |
| 16 | 6 | 2 | 1 | 0 | 2 | 1 | 1 | 3 | 4 | 4 | 6 | 5 |
| 17 | 0 | 0 | 0 | 0 | 0 | 0 | 0 | 0 | 0 | 1 | 0 | 1 |
| 18 | 0 | 1 | 3 | 1 | 0 | 3 | 2 | 1 | 0 | 1 | 2 | 1 |
| 19 | 1 | 0 | 0 | 0 | 1 | 1 | 0 | 0 | 0 | 1 | 1 | 0 |
| 20 | 4 | 2 | 5 | 5 | 12 | 5 | 7 | 4 | 4 | 8 | 4 | 4 |
| 21 | 1 | 1 | 0 | 0 | 0 | 0 | 1 | 1 | 2 | 0 | 1 | 0 |
| 22 | 2 | 3 | 2 | 4 | 2 | 2 |  | 3 | 4 | 7 | 4 | 1 |
| 23 |  |  |  |  |  |  |  |  |  |  |  |  |
| 24 | 3 | 1 | 0 | 2 | 2 | 1 | 0 | 1 | 2 | 0 | 1 | 1 |
| 25 | 2 | 0 | 0 | 3 | 1 | 1 | 2 | 3 | 1 | 4 | 3 | 2 |
| 26 | 2 | 2 |  | 3 | 3 | 3 | 4 | 2 | 2 | 1 | 1 | 1 |
| 27 | 2 | 1 | 2 | 3 | 2 | 2 | 1 | 3 | 4 | 2 | 2 | 1 |
| 28 | 0 | 0 | 0 | 0 | 2 | 0 | 1 | 1 | 0 | 1 | 1 | 0 |
| 29 |  |  |  |  |  |  |  |  |  |  |  |  |
| 30 | 2 | 1 | 0 | 0 | 1 | 1 | 3 | 1 | 1 | 2 | 0 | 0 |
| 31 | 1 | 0 | 1 | 1 | 2 | 0 | 0 | 0 | 1 | 2 | 1 | 2 |
| 32 | 2 | 1 | 1 | 1 |  | 2 | 1 |  |  | 1 |  | 1 |
| 33 | 1 | 0 | 0 | 1 | 1 | 0 | 1 | 1 | 0 | 2 | 0 | 1 |
| 34 | 0 | 1 | 2 | 0 | 1 | 0 | 0 | 0 | 0 | 0 | 1 | 2 |
| 35 | 1 | 0 | 1 | 2 | 0 | 0 | 0 | 0 | 0 | 0 | 0 | 1 |
| 36 |  | 2 |  | 4 | 2 | 1 | 2 | 2 | 5 | 6 | 2 | 3 |
| 37 | 4 | 2 | 0 | 6 | 5 | 0 | 0 | 1 | 3 | 6 | 3 | 4 |
| 38 |  |  |  |  |  |  |  |  |  |  |  |  |
| 39 |  |  |  | 1 | 2 |  |  |  | 1 | 1 |  | 2 |
| 40 | 0 | 3 | 2 | 1 | 4 | 2 | 0 | 0 | 2 | 3 | 2 | 1 |
| 41 | 1 | 1 | 1 | 1 | 3 | 3 | 4 | 1 | 1 | 0 | 0 | 1 |
| 42 | 4 | 1 | 1 | 0 | 1 | 1 | 1 | 2 | 1 | 1 | 0 | 0 |
| 43 | 2 | 2 | 3 | 3 | 3 | 2 | 4 | 2 | 0 | 1 | 0 | 1 |
| 44 | 0 | 0 | 0 | 2 | 1 | 0 | 0 | 0 | 1 | 1 | 0 | 2 |
| 45 | 1 | 1 | 2 | 1 | 3 | 1 | 1 | 3 | 1 | 4 | 1 | 2 |
| 46 | 3 | 3 | 2 | 3 | 2 | 0 | 3 | 4 | 4 | 3 | 1 | 5 |
| 47 |  |  |  |  |  |  |  |  |  |  |  |  |
| 48 | 0 | 2 | 1 | 2 | 1 | 1 | 1 | 4 | 0 | 0 | 0 | 2 |
| 49 | 0 | 2 | 1 | 1 | 1 | 1 | 1 | 1 | 2 | 5 | 2 | 1 |
| 50 | 1 | 0 | 0 | 0 | 0 | 0 | 0 | 0 | 0 | 0 | 1 | 0 |
| 51 | 0 | 2 | 2 | 3 | 2 | 1 | 0 | 2 | 2 | 0 | 2 | 2 |
| 52 | 3 | 1 | 3 | 3 | 6 | 4 | 4 | 3 | 1 | 2 | 0 | 4 |
| 53 | 1 | 3 | 1 | 3 | 2 | 1 | 4 | 1 | 2 | 3 | 3 | 0 |
| 54 | 1 | 2 | 6 | 6 | 3 | 2 | 8 | 6 | 2 | 5 | 7 | 6 |
| 55 | 1 | 0 | 1 | 1 | 2 | 1 | 1 | 0 | 1 | 1 | 0 | 0 |
| 56 | 3 | 1 | 4 | 0 | 5 | 3 | 1 | 3 | 1 | 5 | 3 | 7 |
| 57 | 1 | 5 | 4 | 4 | 2 | 3 | 5 | 0 | 4 | 2 | 5 | 3 |
| 58 | 0 | 0 | 1 | 1 | 1 | 1 | 2 | 2 | 0 | 0 | 1 | 0 |
| 59 | 0 | 3 | 3 | 0 | 0 | 2 | 0 | 1 | 0 | 1 | 1 | 1 |
| 60 | 14 | 3 | 3 | 1 | 3 | 1 | 2 | 0 | 1 | 3 | 1 | 1 |
| 61 | 1 | 0 | 1 | 2 | 1 | 2 | 2 | 2 | 0 | 0 | 2 | 0 |
| 62 |  |  |  |  |  |  |  |  |  |  |  |  |
| 63 |  |  |  |  |  | 1 |  |  | 2 |  |  |  |
| 64 | 4 | 4 | 2 |  | 2 | 2 | 1 | 1 |  | 4 |  | 2 |
| 65 | 1 | 2 | 0 | 2 | 0 | 4 | 2 | 0 | 3 | 2 | 3 | 2 |
| 66 | 0 | 1 | 1 | 1 | 0 | 0 | 0 | 0 | 0 | 0 | 0 | 0 |
| 67 | 2 | 1 | 2 | 0 | 0 | 0 | 1 | 3 | 3 | 4 | 3 | 1 |
| 68 | 0 | 0 | 0 | 2 | 3 | 2 | 3 | 6 | 0 | 1 | 0 | 0 |
| 68 | 9 | 2 | 11 | 3 | 5 | 1 | 6 | 1 | 1 | 1 | 1 | 2 |
| 70 |  |  | 1 |  |  |  |  |  |  |  |  |  |
| 71 | 2 | 1 | 0 | 4 | 1 | 1 | 0 | 4 | 0 | 2 | 3 | 1 |
| 72 | 0 | 0 | 1 | 1 | 0 | 3 | 0 | 1 | 0 | 0 | 2 | 2 |
| 73 |  |  |  |  |  |  |  |  |  |  |  |  |
| 74 | 2 | 3 | 3 | 2 | 0 | 0 | 3 | 1 | 2 | 3 | 1 | 2 |
| 75 |  | 1 |  |  |  |  |  |  | 1 |  |  |  |
| 76 | 6 | 2 | 1 | 5 | 1 | 4 | 1 | 2 | 2 | 5 | 5 | 3 |
| 77 |  |  |  | 1 | 2 |  |  | 1 |  | 1 |  |  |
| 78 |  | 1 |  |  |  |  |  |  |  |  |  | 1 |
| 79 | 3 | 0 | 0 | 0 | 0 | 2 | 0 | 0 | 1 | 3 | 3 | 2 |
| 80 | 1 | 1 | 4 | 0 | 0 | 0 | 2 | 0 | 2 | 0 | 3 | 0 |
| 81 | 1 | 1 | 1 |  | 2 |  | 1 | 1 |  | 2 | 2 |  |
| 82 |  |  |  |  |  |  |  |  |  |  |  |  |
| 83 | 0 | 1 | 0 | 0 | 0 | 0 | 0 | 2 | 1 | 0 | 1 | 2 |
| 84 | 2 | 0 | 2 | 1 | 3 | 1 | 2 | 1 | 3 | 3 | 4 | 0 |
| 85 | 1 | 1 | 3 | 4 |  | 1 | 4 |  | 2 | 4 | 2 | 1 |
| 86 | 0 | 0 | 0 | 2 | 1 | 0 | 0 | 1 | 1 | 0 | 0 | 0 |
| 87 |  | 1 | 1 | 2 | 1 | 1 |  |  | 1 | 1 |  |  |
| 88 | 1 | 0 | 1 | 1 | 0 | 0 | 0 | 0 | 0 | 0 | 0 | 0 |
| 89 | 3 | 7 | 4 | 4 | 7 | 11 | 2 | 3 | 7 | 8 | 2 | 1 |
| 90 | 0 | 1 | 2 | 3 | 0 | 0 | 3 | 4 | 2 | 1 | 2 | 0 |
| 91 | 0 | 0 | 2 | 2 | 0 | 0 | 1 | 2 | 2 | 1 | 1 | 3 |
| 92 |  |  |  |  |  |  |  |  |  |  |  |  |
| 93 | 1 | 0 | 0 | 2 | 0 | 0 | 1 | 2 | 1 | 2 | 3 | 0 |
| 94 | 1 | 0 | 1 | 0 | 2 | 1 | 0 | 1 | 0 | 0 | 1 | 0 |
| 95 | 1 | 1 | 1 | 0 | 1 | 1 | 2 | 0 | 0 | 0 | 1 | 1 |
| 96 |  |  |  |  |  | 1 |  | 1 |  |  |  |  |
| 97 |  |  |  |  |  |  |  |  |  |  |  |  |
| 98 | 0 | 0 | 2 | 2 | 2 | 0 | 1 | 0 | 0 | 1 | 1 | 1 |
| 99 | 0 | 1 | 1 | 0 | 0 | 1 | 0 | 3 | 2 | 0 | 0 | 2 |
| 100 | 2 | 0 | 1 | 2 | 0 | 0 | 0 | 1 | 1 | 1 | 1 | 0 |
| 101 | 1 | 4 |  | 2 | 1 | 1 |  | 4 | 1 |  | 1 | 1 |
| 102 |  |  |  |  |  |  |  |  |  |  |  |  |
| 103 | 2 | 3 | 1 | 3 | 1 | 1 | 0 | 2 | 0 | 1 | 2 | 0 |
| 104 | 3 | 1 | 2 | 4 | 3 | 4 | 4 | 0 | 0 | 2 | 0 | 3 |
| 105 | 2 | 1 | 0 | 1 | 0 | 0 | 1 | 0 | 0 | 2 | 1 | 1 |

Table 38. Number of newly diagnosed stage III breast cancer during each month in 2020.

|  | Month | | | | | | | | | | | |
| --- | --- | --- | --- | --- | --- | --- | --- | --- | --- | --- | --- | --- |
| Hospital | 1 | 2 | 3 | 4 | 5 | 6 | 7 | 8 | 9 | 10 | 11 | 12 |
| 1 | 0 | 0 | 0 | 0 | 0 | 0 | 0 | 0 | 1 | 1 | 0 | 0 |
| 2 | 5 | 5 | 12 | 11 | 6 | 4 | 9 | 9 | 2 | 6 | 9 | 5 |
| 3 | 0 | 1 | 1 | 1 | 1 | 2 | 1 | 0 | 0 | 0 | 0 | 0 |
| 4 | 2 | 1 | 2 | 3 | 1 | 0 | 1 | 0 | 2 | 2 | 3 | 2 |
| 5 | 0 | 1 | 0 | 0 | 0 | 0 | 0 | 0 | 1 | 0 | 0 | 1 |
| 6 |  | 1 | 2 | 2 |  | 1 | 2 | 2 | 1 | 5 |  | 1 |
| 7 | 0 | 1 | 1 | 1 | 0 | 1 | 0 | 1 | 0 | 0 | 2 | 1 |
| 8 |  |  |  |  |  |  |  |  |  |  |  |  |
| 9 | 1 | 0 | 0 | 0 | 1 | 2 | 0 | 5 | 1 | 1 | 0 | 3 |
| 10 | 1 | 0 | 0 | 1 | 0 | 3 | 2 | 2 | 0 | 2 | 2 | 2 |
| 11 | 1 | 2 | 4 | 2 | 1 | 0 | 4 | 0 | 3 | 1 | 1 | 2 |
| 12 | 0 | 0 | 0 | 1 | 0 | 1 | 1 | 0 | 0 | 1 | 1 | 0 |
| 13 | 1 | 2 | 1 | 2 | 1 | 0 | 2 | 0 | 2 | 1 | 1 | 1 |
| 14 | 0 | 1 | 0 | 1 | 1 | 2 | 2 | 0 | 0 | 1 | 1 | 0 |
| 15 | 1 | 3 | 4 | 5 | 1 | 5 | 6 | 2 | 2 | 1 | 4 | 2 |
| 16 | 3 | 1 | 1 | 3 | 2 | 6 | 7 | 3 | 2 | 4 | 7 | 1 |
| 17 | 1 | 0 | 0 | 1 | 0 | 0 | 0 | 0 | 0 | 0 | 0 | 1 |
| 18 | 4 | 2 | 0 | 0 | 0 | 0 | 1 | 1 | 0 | 1 | 2 | 0 |
| 19 | 0 | 0 | 0 | 2 | 1 | 0 | 2 | 0 | 0 | 2 | 0 | 0 |
| 20 | 8 | 5 | 6 | 0 | 3 | 2 | 7 | 6 | 3 | 5 | 13 | 5 |
| 21 | 0 | 3 | 2 | 0 | 1 | 0 | 1 | 1 | 1 | 2 | 0 | 0 |
| 22 | 2 |  | 3 | 1 | 1 | 3 | 2 | 2 | 2 | 5 | 1 | 2 |
| 23 |  |  |  |  |  |  |  |  |  |  |  |  |
| 24 | 3 | 1 | 1 | 0 | 2 | 1 | 2 | 2 | 0 | 2 | 0 | 0 |
| 25 | 1 | 1 | 0 | 1 | 0 | 3 | 1 | 0 | 2 | 1 | 0 | 0 |
| 26 | 2 | 1 | 2 | 3 | 3 | 2 | 1 | 3 | 2 | 2 | 1 |  |
| 27 | 3 | 3 | 1 | 1 | 0 | 3 | 1 | 0 | 4 | 1 | 2 | 1 |
| 28 | 3 | 1 | 1 | 2 | 0 | 0 | 1 | 1 | 1 | 1 | 1 | 1 |
| 29 |  |  |  |  |  |  |  |  |  |  |  |  |
| 30 | 2 | 0 | 1 | 2 | 3 | 0 | 1 | 0 | 0 | 0 | 1 | 1 |
| 31 | 1 | 1 | 3 | 0 | 1 | 2 | 2 | 1 | 0 | 3 | 1 | 2 |
| 32 | 1 |  | 4 | 2 | 4 | 2 | 3 | 1 | 1 | 2 | 1 | 1 |
| 33 | 1 | 1 | 2 | 1 | 1 | 1 | 0 | 1 | 0 | 1 | 0 | 1 |
| 34 | 0 | 2 | 1 | 0 | 0 | 0 | 0 | 1 | 0 | 0 | 2 | 1 |
| 35 | 1 | 0 | 1 | 0 | 1 | 2 | 0 | 2 | 0 | 1 | 0 | 1 |
| 36 | 2 | 1 |  | 4 |  | 2 | 1 |  | 3 | 1 | 2 |  |
| 37 | 2 | 3 | 2 | 4 | 3 | 2 | 1 | 3 | 4 | 2 | 3 | 1 |
| 38 |  |  |  |  |  |  |  |  |  |  |  |  |
| 39 |  |  |  |  |  |  | 1 | 2 |  |  |  |  |
| 40 | 1 | 4 | 0 | 0 | 2 | 2 | 2 | 1 | 3 | 1 | 4 | 1 |
| 41 | 1 | 2 | 0 | 1 | 0 | 3 | 1 | 1 | 2 | 3 | 3 | 2 |
| 42 | 1 | 0 | 3 | 0 | 1 | 1 | 0 | 1 | 2 | 1 | 3 | 3 |
| 43 | 1 | 2 | 1 | 0 | 2 | 0 | 1 | 2 | 0 | 0 | 1 | 0 |
| 44 | 0 | 0 | 0 | 1 | 0 | 1 | 1 | 0 | 0 | 1 | 1 | 2 |
| 45 | 2 | 0 | 2 | 6 | 3 | 1 | 0 | 0 | 1 | 2 | 2 | 1 |
| 46 | 5 | 2 | 5 | 6 | 3 | 5 | 0 | 0 | 1 | 6 | 3 | 2 |
| 47 |  |  |  |  |  |  |  |  |  |  |  |  |
| 48 | 1 | 1 | 1 | 0 | 1 | 0 | 1 | 1 | 1 | 1 | 0 | 2 |
| 49 | 3 | 3 | 1 | 0 | 2 | 1 | 0 | 0 | 0 | 0 | 0 | 0 |
| 50 | 0 | 0 | 0 | 0 | 0 | 1 | 1 | 2 | 0 | 1 | 0 | 1 |
| 51 | 0 | 0 | 2 | 0 | 1 | 3 | 0 | 1 | 7 | 0 | 1 | 0 |
| 52 | 2 | 4 | 0 | 1 | 0 | 2 | 2 | 0 | 5 | 1 | 3 | 2 |
| 53 | 2 | 2 | 4 | 3 | 1 | 2 | 1 | 3 | 0 | 3 | 0 | 1 |
| 54 | 1 | 6 | 4 | 4 | 3 | 6 | 7 | 2 | 5 | 4 | 4 | 5 |
| 55 | 1 | 0 | 0 | 2 | 2 | 3 | 0 | 0 | 1 | 1 | 4 | 0 |
| 56 | 2 | 2 | 1 | 1 | 0 | 0 | 2 | 5 | 6 | 1 | 1 | 3 |
| 57 | 2 | 1 | 3 | 5 | 4 | 3 | 1 | 0 | 7 | 3 | 5 | 3 |
| 58 | 2 | 1 | 0 | 0 | 0 | 1 | 2 | 1 | 0 | 2 | 1 | 0 |
| 59 | 1 | 0 | 1 | 0 | 2 | 1 | 3 | 3 | 2 | 0 | 2 | 1 |
| 60 | 0 | 1 | 2 | 2 | 1 | 0 | 1 | 0 | 0 | 1 | 0 | 3 |
| 61 | 2 | 0 | 1 | 0 | 1 | 2 | 0 | 0 | 2 | 0 | 1 | 1 |
| 62 |  |  |  |  |  |  |  |  |  |  |  |  |
| 63 |  | 1 |  | 1 |  |  | 1 |  | 2 |  |  |  |
| 64 | 2 | 1 | 3 | 1 | 1 | 1 | 2 | 1 | 2 | 2 | 1 |  |
| 65 | 1 | 0 | 1 | 2 | 5 | 2 | 3 | 2 | 2 | 0 | 4 | 0 |
| 66 | 0 | 0 | 0 | 0 | 0 | 1 | 1 | 1 | 0 | 0 | 1 | 0 |
| 67 | 3 | 1 | 1 | 2 | 1 | 1 | 2 | 3 | 2 | 1 | 1 | 1 |
| 68 | 2 | 3 | 1 | 3 | 0 | 2 | 2 | 1 | 0 | 3 | 4 | 2 |
| 68 | 0 | 0 | 0 | 0 | 1 | 1 | 0 | 1 | 0 | 0 | 0 | 0 |
| 70 | 1 |  | 1 |  |  |  |  | 1 |  | 1 | 1 |  |
| 71 | 2 | 0 | 0 | 1 | 1 | 3 | 1 | 1 | 2 | 1 | 2 | 1 |
| 72 | 0 | 1 | 1 | 0 | 0 | 2 | 2 | 1 | 0 | 0 | 1 | 0 |
| 73 |  |  |  |  |  |  |  |  |  |  |  |  |
| 74 | 3 | 2 | 4 | 3 | 1 | 2 | 2 | 1 | 2 | 3 | 1 | 1 |
| 75 | 1 |  |  |  |  |  | 1 |  |  |  |  |  |
| 76 | 2 | 3 | 2 | 5 | 2 | 5 | 5 | 4 | 2 | 3 | 4 | 2 |
| 77 |  |  |  |  |  |  |  |  | 1 |  |  |  |
| 78 |  |  | 1 |  |  |  |  |  |  |  | 1 |  |
| 79 | 1 | 1 | 2 | 2 | 1 | 2 | 5 | 4 | 0 | 2 | 3 | 0 |
| 80 | 2 | 1 | 1 | 0 | 2 | 2 | 0 | 6 | 4 | 3 | 2 | 2 |
| 81 |  | 1 | 1 | 1 |  | 1 | 1 | 1 | 2 |  | 1 | 1 |
| 82 |  |  |  |  |  |  |  |  |  |  |  |  |
| 83 | 1 | 1 | 1 | 0 | 1 | 0 | 0 | 2 | 1 | 3 | 1 | 0 |
| 84 | 2 | 2 | 0 | 0 | 1 | 2 | 3 | 1 | 1 | 2 | 5 | 2 |
| 85 | 3 |  | 4 | 4 |  | 1 | 3 |  | 6 |  | 2 | 2 |
| 86 | 0 | 1 | 1 | 2 | 2 | 0 | 1 | 0 | 2 | 0 | 1 | 1 |
| 87 | 1 |  |  |  | 1 | 1 | 1 |  |  | 1 |  |  |
| 88 | 0 | 0 | 0 | 0 | 2 | 0 | 0 | 0 | 0 | 0 | 1 | 0 |
| 89 | 2 | 6 | 2 | 2 | 8 | 4 | 3 | 4 | 4 | 5 | 9 | 5 |
| 90 | 1 | 0 | 1 | 0 | 1 | 1 | 1 | 1 | 1 | 2 | 0 | 3 |
| 91 | 0 | 0 | 1 | 1 | 0 | 0 | 1 | 1 | 4 | 2 | 1 | 0 |
| 92 |  |  |  |  |  |  |  |  |  |  |  |  |
| 93 | 1 | 1 | 0 | 1 | 0 | 1 | 1 | 0 | 0 | 2 | 0 | 2 |
| 94 | 2 | 0 | 0 | 1 | 0 | 1 | 0 | 0 | 0 | 1 | 0 | 0 |
| 95 | 0 | 1 | 0 | 0 | 1 | 1 | 2 | 1 | 1 | 1 | 1 | 1 |
| 96 |  |  |  |  |  |  |  |  |  |  |  |  |
| 97 |  |  |  |  |  |  |  |  |  |  |  |  |
| 98 | 1 | 3 | 3 | 1 | 3 | 0 | 1 | 2 | 0 | 3 | 0 | 0 |
| 99 | 0 | 0 | 1 | 2 | 0 | 2 | 0 | 0 | 2 | 1 | 0 | 0 |
| 100 | 0 | 1 | 2 | 3 | 2 | 1 | 0 | 1 | 1 | 0 | 0 | 0 |
| 101 | 3 | 1 | 2 |  | 1 | 4 | 3 | 1 | 3 | 1 |  |  |
| 102 |  |  |  |  |  |  |  |  |  |  |  |  |
| 103 | 2 | 2 | 0 | 0 | 2 | 0 | 1 | 4 | 4 | 4 | 1 | 2 |
| 104 | 1 | 2 | 0 | 5 | 5 | 3 | 2 | 2 | 1 | 3 | 1 | 2 |
| 105 | 2 | 1 | 2 | 2 | 2 | 1 | 0 | 1 | 0 | 4 | 0 | 0 |

Table 39. Number of newly diagnosed stage IV breast cancer during each month in 2019.

|  | Month | | | | | | | | | | | |
| --- | --- | --- | --- | --- | --- | --- | --- | --- | --- | --- | --- | --- |
| Hospital | 1 | 2 | 3 | 4 | 5 | 6 | 7 | 8 | 9 | 10 | 11 | 12 |
| 1 | 0 | 0 | 0 | 0 | 0 | 0 | 0 | 0 | 0 | 0 | 0 | 0 |
| 2 | 7 | 1 | 2 | 3 | 2 | 4 | 6 | 3 | 7 | 4 | 2 | 1 |
| 3 | 0 | 1 | 0 | 1 | 1 | 1 | 2 | 0 | 0 | 0 | 0 | 1 |
| 4 | 1 | 3 | 1 | 2 | 3 | 0 | 3 | 1 | 0 | 2 | 3 | 3 |
| 5 | 0 | 0 | 0 | 0 | 1 | 0 | 2 | 0 | 0 | 0 | 2 | 1 |
| 6 | 1 | 1 |  |  | 1 |  |  | 3 |  | 1 |  | 3 |
| 7 | 0 | 0 | 0 | 0 | 1 | 0 | 0 | 0 | 0 | 1 | 3 | 0 |
| 8 |  |  |  |  |  |  |  |  |  |  |  |  |
| 9 | 1 | 1 | 2 | 1 | 2 | 0 | 0 | 0 | 0 | 1 | 1 | 1 |
| 10 | 0 | 0 | 0 | 2 | 3 | 2 | 3 | 0 | 0 | 0 | 3 | 1 |
| 11 | 0 | 0 | 0 | 1 | 1 | 1 | 1 | 0 | 0 | 0 | 1 | 1 |
| 12 | 1 | 0 | 0 | 0 | 2 | 0 | 0 | 0 | 0 | 0 | 0 | 0 |
| 13 | 0 | 3 | 1 | 1 | 2 | 2 | 1 | 1 | 2 | 0 | 2 | 0 |
| 14 | 1 | 1 | 1 | 0 | 1 | 1 | 1 | 1 | 1 | 1 | 1 | 0 |
| 15 | 2 | 0 | 0 | 1 | 1 | 1 | 3 | 1 | 1 | 3 | 0 | 0 |
| 16 | 3 | 2 | 1 | 3 | 3 | 2 | 4 | 3 | 0 | 4 | 1 | 3 |
| 17 | 0 | 0 | 0 | 0 | 0 | 0 | 0 | 0 | 0 | 0 | 0 | 0 |
| 18 | 0 | 0 | 1 | 0 | 0 | 0 | 1 | 2 | 0 | 0 | 1 | 0 |
| 19 | 0 | 1 | 0 | 1 | 0 | 0 | 0 | 1 | 0 | 1 | 0 | 0 |
| 20 | 2 | 4 | 2 | 3 | 1 | 4 | 3 | 4 | 1 | 3 | 2 | 1 |
| 21 | 1 | 3 | 3 | 0 | 1 | 0 | 0 | 2 | 0 | 0 | 1 | 0 |
| 22 |  |  |  | 1 | 1 | 3 | 2 |  | 2 | 1 | 2 |  |
| 23 |  |  |  |  |  |  |  |  |  |  |  |  |
| 24 | 1 | 1 | 0 | 0 | 4 | 1 | 1 | 0 | 3 | 4 | 1 | 1 |
| 25 | 0 | 1 | 2 | 1 | 0 | 0 | 1 | 1 | 0 | 2 | 1 | 1 |
| 26 |  | 1 |  |  | 2 |  | 1 | 1 |  | 1 |  | 1 |
| 27 | 4 | 5 | 4 | 1 | 3 | 3 | 7 | 3 | 3 | 3 | 3 | 2 |
| 28 | 0 | 0 | 1 | 0 | 0 | 0 | 1 | 0 | 0 | 1 | 0 | 1 |
| 29 |  |  |  |  |  |  |  |  |  |  |  |  |
| 30 | 1 | 0 | 0 | 1 | 0 | 0 | 0 | 0 | 0 | 0 | 1 | 0 |
| 31 | 1 | 0 | 1 | 0 | 1 | 2 | 3 | 0 | 1 | 1 | 0 | 0 |
| 32 |  | 1 | 1 | 2 | 2 |  | 1 | 3 | 1 |  |  | 1 |
| 33 | 2 | 1 | 0 | 0 | 1 | 2 | 0 | 0 | 3 | 0 | 0 | 2 |
| 34 | 0 | 1 | 3 | 1 | 1 | 3 | 2 | 0 | 1 | 4 | 0 | 0 |
| 35 | 1 | 1 | 0 | 0 | 0 | 0 | 0 | 0 | 0 | 2 | 0 | 1 |
| 36 | 1 |  |  |  | 1 | 3 | 3 |  |  |  |  | 1 |
| 37 | 2 | 1 | 4 | 2 | 2 | 4 | 1 | 1 | 4 | 2 | 2 | 1 |
| 38 |  |  |  |  |  |  |  |  |  |  |  |  |
| 39 |  | 1 |  |  |  |  |  |  |  |  |  |  |
| 40 | 1 | 0 | 0 | 0 | 2 | 1 | 1 | 0 | 0 | 0 | 1 | 1 |
| 41 | 0 | 0 | 1 | 1 | 0 | 0 | 2 | 2 | 0 | 1 | 0 | 0 |
| 42 | 1 | 1 | 3 | 1 | 0 | 0 | 0 | 0 | 0 | 0 | 0 | 2 |
| 43 | 0 | 0 | 0 | 1 | 1 | 0 | 1 | 0 | 0 | 0 | 0 | 1 |
| 44 | 0 | 0 | 0 | 0 | 1 | 0 | 0 | 0 | 0 | 1 | 0 | 0 |
| 45 | 1 | 2 | 0 | 1 | 1 | 1 | 3 | 1 | 0 | 1 | 1 | 1 |
| 46 | 1 | 0 | 0 | 2 | 6 | 2 | 2 | 1 | 1 | 0 | 0 | 1 |
| 47 |  |  |  |  |  |  |  |  |  |  |  |  |
| 48 | 3 | 2 | 0 | 1 | 0 | 0 | 1 | 2 | 0 | 1 | 0 | 0 |
| 49 | 0 | 1 | 1 | 0 | 1 | 0 | 1 | 0 | 3 | 1 | 2 | 0 |
| 50 | 0 | 0 | 0 | 0 | 1 | 0 | 0 | 0 | 0 | 0 | 0 | 0 |
| 51 | 0 | 0 | 0 | 1 | 1 | 1 | 1 | 0 | 1 | 1 | 0 | 1 |
| 52 | 0 | 1 | 0 | 1 | 1 | 1 | 0 | 0 | 1 | 1 | 0 | 2 |
| 53 | 1 | 1 | 0 | 4 | 1 | 0 | 0 | 2 | 1 | 0 | 0 | 1 |
| 54 | 5 | 5 | 3 | 6 | 1 | 1 | 2 | 4 | 2 | 2 | 2 | 2 |
| 55 | 0 | 0 | 0 | 0 | 0 | 0 | 1 | 0 | 1 | 1 | 0 | 1 |
| 56 | 0 | 1 | 1 | 2 | 1 | 2 | 1 | 4 | 2 | 0 | 1 | 2 |
| 57 | 2 | 5 | 0 | 0 | 4 | 2 | 3 | 2 | 2 | 1 | 7 | 1 |
| 58 | 0 | 1 | 0 | 0 | 1 | 0 | 0 | 0 | 1 | 0 | 3 | 0 |
| 59 | 0 | 1 | 1 | 2 | 0 | 0 | 2 | 0 | 0 | 0 | 0 | 0 |
| 60 | 0 | 1 | 0 | 1 | 0 | 2 | 1 | 0 | 1 | 3 | 1 | 1 |
| 61 | 0 | 0 | 3 | 0 | 0 | 1 | 0 | 3 | 0 | 3 | 0 | 1 |
| 62 |  |  |  |  |  |  |  |  |  |  |  |  |
| 63 |  |  |  |  | 1 |  | 1 |  | 1 |  |  |  |
| 64 | 2 | 1 | 1 | 4 | 2 | 1 | 2 |  | 4 | 1 | 1 | 3 |
| 65 | 1 | 0 | 0 | 1 | 1 | 0 | 1 | 0 | 1 | 1 | 0 | 1 |
| 66 | 0 | 0 | 0 | 0 | 0 | 0 | 3 | 0 | 4 | 0 | 0 | 0 |
| 67 | 0 | 0 | 0 | 0 | 0 | 1 | 1 | 1 | 0 | 0 | 0 | 2 |
| 68 | 1 | 0 | 0 | 0 | 1 | 1 | 0 | 0 | 0 | 1 | 1 | 2 |
| 68 | 1 | 0 | 1 | 3 | 1 | 0 | 0 | 0 | 0 | 0 | 0 | 0 |
| 70 |  |  |  |  |  |  |  |  |  |  | 1 |  |
| 71 | 2 | 0 | 2 | 1 | 1 | 4 | 2 | 0 | 1 | 1 | 1 | 0 |
| 72 | 0 | 1 | 1 | 0 | 0 | 1 | 1 | 2 | 2 | 1 | 2 | 1 |
| 73 |  |  |  |  |  |  |  |  |  |  |  |  |
| 74 | 2 | 3 | 1 | 2 | 0 | 3 | 4 | 2 | 2 | 4 | 0 | 0 |
| 75 |  |  |  |  |  |  |  | 1 |  |  |  |  |
| 76 | 2 | 0 | 1 | 1 | 4 | 1 | 2 | 2 | 1 | 0 | 0 | 1 |
| 77 | 1 |  |  |  |  |  |  |  |  |  |  |  |
| 78 |  | 1 |  |  |  |  |  |  |  |  |  |  |
| 79 | 2 | 0 | 1 | 0 | 0 | 1 | 1 | 0 | 1 | 2 | 0 | 2 |
| 80 | 0 | 0 | 1 | 0 | 1 | 3 | 2 | 3 | 0 | 1 | 2 | 0 |
| 81 |  |  |  |  |  | 1 |  |  | 1 | 2 |  |  |
| 82 |  |  |  |  |  |  |  |  |  |  |  |  |
| 83 | 1 | 0 | 1 | 1 | 1 | 0 | 1 | 0 | 0 | 0 | 0 | 0 |
| 84 | 1 | 2 | 0 | 2 | 2 | 1 | 1 | 1 | 0 | 0 | 1 | 0 |
| 85 | 1 |  |  |  | 1 | 3 | 1 | 2 | 3 | 4 | 3 | 1 |
| 86 | 1 | 0 | 1 | 0 | 1 | 1 | 2 | 2 | 0 | 2 | 0 | 3 |
| 87 |  |  |  | 1 | 1 |  |  |  | 2 | 1 |  |  |
| 88 | 2 | 1 | 1 | 2 | 2 | 0 | 0 | 0 | 0 | 0 | 1 | 0 |
| 89 | 2 | 2 | 1 | 1 | 5 | 2 | 3 | 1 | 2 | 1 | 5 | 2 |
| 90 | 2 | 1 | 0 | 3 | 1 | 0 | 0 | 0 | 0 | 1 | 3 | 0 |
| 91 | 1 | 1 | 0 | 1 | 2 | 0 | 0 | 1 | 2 | 1 | 1 | 0 |
| 92 |  |  |  |  |  |  |  |  |  |  |  |  |
| 93 | 2 | 0 | 2 | 1 | 0 | 0 | 2 | 1 | 1 | 0 | 0 | 0 |
| 94 | 0 | 0 | 2 | 0 | 1 | 1 | 0 | 1 | 0 | 0 | 1 | 0 |
| 95 | 0 | 0 | 0 | 0 | 1 | 0 | 0 | 1 | 1 | 0 | 0 | 0 |
| 96 |  |  |  |  |  | 1 |  |  |  | 1 |  |  |
| 97 |  |  |  |  |  |  |  |  |  |  |  |  |
| 98 | 0 | 3 | 0 | 2 | 0 | 1 | 2 | 0 | 0 | 0 | 2 | 1 |
| 99 | 0 | 1 | 0 | 2 | 1 | 0 | 1 | 0 | 1 | 1 | 0 | 0 |
| 100 | 0 | 0 | 2 | 0 | 1 | 2 | 0 | 0 | 0 | 0 | 1 | 0 |
| 101 | 2 | 1 |  | 1 | 1 | 2 | 1 | 1 |  |  | 2 |  |
| 102 |  |  |  |  |  |  |  |  |  |  |  |  |
| 103 | 0 | 2 | 2 | 0 | 2 | 0 | 0 | 2 | 2 | 0 | 1 | 1 |
| 104 | 2 | 0 | 3 | 1 | 4 | 0 | 4 | 1 | 0 | 2 | 3 | 0 |
| 105 | 0 | 1 | 0 | 0 | 1 | 0 | 0 | 2 | 0 | 1 | 0 | 2 |

Table 40. Number of newly diagnosed stage IV breast cancer during each month in 2020.

|  | Month | | | | | | | | | | | |
| --- | --- | --- | --- | --- | --- | --- | --- | --- | --- | --- | --- | --- |
| Hospital | 1 | 2 | 3 | 4 | 5 | 6 | 7 | 8 | 9 | 10 | 11 | 12 |
| 1 | 1 | 0 | 0 | 1 | 0 | 0 | 0 | 0 | 0 | 1 | 0 | 0 |
| 2 | 4 | 4 | 1 | 1 | 2 | 1 | 3 | 2 | 3 | 2 | 8 | 7 |
| 3 | 1 | 0 | 0 | 0 | 0 | 0 | 1 | 1 | 1 | 0 | 0 | 0 |
| 4 | 1 | 0 | 4 | 2 | 1 | 0 | 1 | 2 | 1 | 0 | 1 | 2 |
| 5 | 0 | 1 | 0 | 0 | 0 | 0 | 0 | 0 | 2 | 0 | 1 | 0 |
| 6 |  |  |  |  |  |  | 3 | 1 | 2 | 1 |  |  |
| 7 | 2 | 0 | 0 | 0 | 0 | 0 | 0 | 0 | 3 | 0 | 1 | 0 |
| 8 |  |  |  |  |  |  |  |  |  |  |  |  |
| 9 | 1 | 0 | 1 | 3 | 1 | 0 | 0 | 0 | 0 | 4 | 0 | 1 |
| 10 | 2 | 3 | 1 |  | 1 | 0 | 0 | 2 | 3 | 0 | 0 | 0 |
| 11 | 2 | 0 | 0 | 1 | 2 | 0 | 0 | 1 | 0 | 2 | 2 | 0 |
| 12 | 0 | 0 | 1 | 0 | 1 | 0 | 0 | 0 | 1 | 0 | 0 | 0 |
| 13 | 0 | 1 | 0 | 0 | 0 | 2 | 2 | 2 | 3 | 3 | 0 | 0 |
| 14 | 3 | 0 | 2 | 2 | 0 | 1 | 3 | 1 | 2 | 2 | 3 | 2 |
| 15 | 4 | 1 | 1 | 1 | 1 | 2 | 0 | 0 | 3 | 1 | 0 | 0 |
| 16 | 2 | 3 | 1 | 0 | 4 | 3 | 1 | 3 | 2 | 1 | 3 | 0 |
| 17 | 0 | 0 | 0 | 0 | 2 | 0 | 0 | 0 | 0 | 1 | 0 | 0 |
| 18 | 1 | 1 | 1 | 0 | 1 | 0 | 0 | 0 | 1 | 0 | 2 | 0 |
| 19 | 1 | 0 | 2 | 0 | 0 | 0 | 0 | 1 | 1 | 4 | 0 | 0 |
| 20 | 2 | 1 | 2 | 1 | 1 | 2 | 2 | 3 | 2 | 4 | 3 | 1 |
| 21 | 3 | 4 | 3 | 1 | 1 | 1 | 3 | 1 | 2 | 2 | 1 | 1 |
| 22 |  |  | 1 |  |  |  | 1 | 1 | 2 | 2 |  | 1 |
| 23 |  |  |  |  |  |  |  |  |  |  |  |  |
| 24 | 3 | 2 | 2 | 0 | 1 | 0 | 2 | 0 | 2 | 1 | 0 | 0 |
| 25 | 0 | 0 | 0 | 2 | 0 | 1 | 1 | 0 | 0 | 3 | 0 | 3 |
| 26 | 2 |  | 1 | 1 |  |  | 2 | 1 | 1 |  | 3 | 1 |
| 27 | 3 | 1 | 1 | 4 | 2 | 4 | 2 | 1 | 1 | 4 | 3 | 2 |
| 28 | 0 | 1 | 2 | 0 | 1 | 1 | 0 | 0 | 1 | 0 | 0 | 0 |
| 29 |  |  |  |  |  |  |  |  |  |  |  |  |
| 30 | 0 | 0 | 0 | 1 | 0 | 1 | 1 | 2 | 1 | 0 | 0 | 0 |
| 31 | 1 | 0 | 1 | 1 | 0 | 0 | 2 | 0 | 1 | 3 | 1 | 0 |
| 32 |  |  |  | 2 | 1 | 1 | 2 | 1 |  | 1 |  | 1 |
| 33 | 0 | 1 | 2 | 3 | 1 | 0 | 1 | 2 | 2 | 1 | 1 | 0 |
| 34 | 1 | 0 | 2 | 0 | 0 | 1 | 2 | 2 | 1 | 2 | 1 | 1 |
| 35 | 2 | 0 | 1 | 1 | 2 | 1 | 0 | 0 | 0 | 0 | 1 | 0 |
| 36 | 1 | 1 | 3 | 1 |  |  | 2 | 1 | 2 |  | 3 | 1 |
| 37 | 2 | 1 | 3 | 3 | 5 | 5 | 1 | 1 | 2 | 2 | 1 | 2 |
| 38 |  |  |  |  |  |  |  |  |  |  |  |  |
| 39 |  |  |  |  |  |  | 1 |  |  |  |  |  |
| 40 | 4 | 1 | 1 | 2 | 1 | 1 | 1 | 3 | 0 | 2 | 2 | 3 |
| 41 | 0 | 2 | 0 | 0 | 1 | 4 | 1 | 1 | 2 | 3 | 0 | 1 |
| 42 | 4 | 0 | 0 | 1 | 0 | 0 | 0 | 0 | 1 | 0 | 1 | 0 |
| 43 | 2 | 1 | 0 | 1 | 0 | 0 | 2 | 1 | 3 | 2 | 1 | 1 |
| 44 | 2 | 0 | 0 | 0 | 0 | 1 | 0 | 0 | 0 | 0 | 0 | 0 |
| 45 | 0 | 0 | 1 | 0 | 1 | 0 | 2 | 1 | 1 | 0 | 1 | 0 |
| 46 | 3 | 4 | 1 | 2 | 3 | 3 | 2 | 1 | 3 | 3 | 0 | 1 |
| 47 |  |  |  |  |  |  |  |  |  |  |  |  |
| 48 | 1 | 2 | 0 | 2 | 0 | 0 | 1 | 0 | 1 | 1 | 2 | 0 |
| 49 | 1 | 0 | 0 | 1 | 1 | 0 | 0 | 0 | 1 | 0 | 0 | 3 |
| 50 | 1 | 0 | 0 | 0 | 0 | 0 | 0 | 0 | 0 | 1 | 0 | 1 |
| 51 | 0 | 0 | 0 | 0 | 3 | 3 | 1 | 1 | 0 | 1 | 0 | 1 |
| 52 | 3 | 0 | 6 | 0 | 0 | 2 | 3 | 1 | 1 | 2 | 0 | 3 |
| 53 | 1 | 0 | 3 | 0 | 0 | 1 | 2 | 1 | 2 | 1 | 1 | 2 |
| 54 | 4 | 3 | 2 | 3 | 2 | 5 | 3 | 3 | 2 | 4 | 4 | 2 |
| 55 | 0 | 1 | 0 | 0 | 1 | 0 | 2 | 1 | 0 | 0 | 0 | 2 |
| 56 | 3 | 3 | 2 | 2 | 2 | 3 | 1 | 1 | 0 | 0 | 1 | 0 |
| 57 | 1 | 0 | 3 | 0 | 4 | 3 | 1 | 5 | 2 | 5 | 2 | 6 |
| 58 | 0 | 0 | 1 | 0 | 0 | 0 | 0 | 2 | 0 | 0 | 3 | 2 |
| 59 | 0 | 1 | 0 | 0 | 0 | 0 | 1 | 0 | 0 | 0 | 1 | 0 |
| 60 | 2 | 0 | 0 | 2 | 0 | 1 | 0 | 0 | 0 | 0 | 1 | 0 |
| 61 | 0 | 0 | 0 | 0 | 1 | 0 | 0 | 1 | 1 | 1 | 0 | 1 |
| 62 |  |  |  |  |  |  |  |  |  |  |  |  |
| 63 | 1 |  | 2 |  | 1 |  |  |  |  |  |  | 3 |
| 64 |  | 2 | 2 |  | 1 | 2 | 1 | 4 | 3 |  |  |  |
| 65 | 0 | 1 | 0 | 3 | 0 | 0 | 0 | 0 | 0 | 0 | 0 | 1 |
| 66 | 1 | 0 | 0 | 0 | 1 | 1 | 0 | 0 | 0 | 0 | 0 | 1 |
| 67 | 1 | 0 | 0 | 0 | 0 | 1 | 0 | 0 | 1 | 0 | 0 | 0 |
| 68 | 0 | 2 | 0 | 0 | 0 | 0 | 0 | 1 | 1 | 1 | 2 | 0 |
| 68 | 0 | 0 | 0 | 0 | 0 | 1 | 1 | 0 | 0 | 0 | 0 | 0 |
| 70 |  |  |  |  |  |  |  |  |  |  |  |  |
| 71 | 1 | 4 | 1 | 1 | 1 | 1 | 2 | 2 | 1 | 5 | 0 | 0 |
| 72 | 1 | 0 | 0 | 2 | 2 | 0 | 0 | 1 | 0 | 3 | 1 | 3 |
| 73 |  |  |  |  |  |  |  |  |  |  |  |  |
| 74 | 3 | 1 | 3 | 2 | 2 | 5 | 0 | 1 | 2 | 2 | 1 | 0 |
| 75 |  |  |  |  |  |  |  |  |  |  |  |  |
| 76 | 2 | 3 | 0 | 1 | 0 | 1 | 0 | 1 | 3 | 1 | 2 | 2 |
| 77 |  |  |  |  |  |  |  |  |  |  |  |  |
| 78 |  |  |  |  |  |  |  |  |  |  |  | 1 |
| 79 | 2 | 0 | 2 | 1 | 0 | 2 | 3 | 1 | 2 | 5 | 5 | 1 |
| 80 | 1 | 0 | 6 | 4 | 0 | 1 | 1 | 0 | 3 | 1 | 0 | 1 |
| 81 |  |  |  |  |  |  |  | 1 |  |  |  |  |
| 82 |  |  |  |  |  |  |  |  |  |  |  |  |
| 83 | 1 | 0 | 0 | 0 | 0 | 1 | 1 | 0 | 1 | 0 | 0 | 0 |
| 84 | 1 | 0 | 3 | 4 | 0 | 2 | 1 | 0 | 0 | 2 | 0 | 0 |
| 85 | 1 | 2 | 2 |  |  | 2 | 3 | 3 | 2 | 1 | 1 |  |
| 86 | 1 | 3 | 1 | 1 | 0 | 1 | 3 | 0 | 1 | 1 | 0 | 1 |
| 87 | 2 |  |  | 1 | 1 |  |  | 1 |  |  |  |  |
| 88 | 0 | 1 | 0 | 1 | 0 | 0 | 1 | 0 | 0 | 0 | 1 | 0 |
| 89 | 1 | 1 | 3 | 4 | 6 | 6 | 2 |  | 2 | 5 | 3 | 5 |
| 90 | 0 | 2 | 0 | 2 | 0 | 1 | 2 | 3 | 2 | 0 | 0 | 0 |
| 91 | 2 | 0 | 0 | 3 | 3 | 1 | 0 | 1 | 0 | 0 | 2 | 2 |
| 92 |  |  |  |  |  |  |  |  |  |  |  |  |
| 93 | 1 | 0 | 0 | 0 | 0 | 0 | 0 | 0 | 0 | 2 | 1 | 0 |
| 94 | 0 | 0 | 0 | 0 | 0 | 0 | 0 | 1 | 0 | 0 | 0 | 0 |
| 95 | 0 | 0 | 1 | 0 | 0 | 1 | 1 | 0 | 0 | 0 | 0 | 0 |
| 96 |  |  | 1 | 2 |  |  |  |  | 1 |  | 1 |  |
| 97 |  |  |  |  |  |  |  |  |  |  |  |  |
| 98 | 0 | 2 | 2 | 2 | 2 | 1 | 2 | 2 | 0 | 0 | 2 | 3 |
| 99 | 2 | 0 | 0 | 1 | 0 | 2 | 2 | 1 | 0 | 2 | 1 | 0 |
| 100 | 1 | 1 | 1 | 1 | 1 | 0 | 0 | 0 | 0 | 1 | 2 | 0 |
| 101 |  |  | 1 | 1 | 3 | 1 |  |  | 1 | 1 |  | 1 |
| 102 |  |  |  |  |  |  |  |  |  |  |  |  |
| 103 | 0 | 0 | 0 | 0 | 4 | 1 | 2 | 1 | 3 | 2 | 0 | 2 |
| 104 | 0 | 1 | 1 | 0 | 3 | 2 | 1 | 1 | 2 | 2 | 1 | 4 |
| 105 | 0 | 0 | 2 | 0 | 2 | 1 | 1 | 0 | 1 | 0 | 1 | 1 |

Table 41. Number of newly diagnosed cervical cancer during each month in 2019.

|  | Month | | | | | | | | | | | |
| --- | --- | --- | --- | --- | --- | --- | --- | --- | --- | --- | --- | --- |
| Hospital | 1 | 2 | 3 | 4 | 5 | 6 | 7 | 8 | 9 | 10 | 11 | 12 |
| 1 | 3 | 0 | 0 | 0 | 4 | 1 | 0 | 2 | 0 | 0 | 1 | 1 |
| 2 | 27 | 23 | 16 | 17 | 11 | 20 | 9 | 10 | 14 | 27 | 22 | 11 |
| 3 | 1 | 2 | 1 | 1 | 1 | 2 | 3 | 1 | 4 | 2 | 3 | 6 |
| 4 | 10 | 18 | 16 | 25 | 12 | 9 | 23 | 16 | 14 | 13 | 17 | 15 |
| 5 | 0 | 1 | 1 | 0 | 0 | 1 | 0 | 0 | 0 | 0 | 1 | 0 |
| 6 | 3 | 5 | 7 | 5 | 5 | 4 | 4 | 5 | 5 | 3 | 5 | 5 |
| 7 | 2 | 3 | 13 | 8 | 2 | 6 | 7 | 4 | 2 | 3 | 4 | 2 |
| 8 |  |  |  |  |  |  |  |  |  |  |  |  |
| 9 | 0 | 0 | 0 | 0 | 1 | 2 | 1 | 0 | 2 | 0 | 0 | 3 |
| 10 | 2 | 1 | 2 | 0 | 3 | 0 | 2 | 1 | 0 | 2 | 1 | 1 |
| 11 | 5 | 7 | 5 | 1 | 4 | 5 | 9 | 3 | 3 | 1 | 3 | 4 |
| 12 | 1 | 0 | 0 | 1 | 1 | 0 | 0 | 0 | 0 | 0 | 1 | 1 |
| 13 | 7 | 7 | 10 | 12 | 9 | 5 | 6 | 9 | 5 | 5 | 9 | 13 |
| 14 | 9 | 7 | 9 | 6 | 3 | 7 | 5 | 3 | 4 | 4 | 10 | 5 |
| 15 | 1 | 1 | 4 | 5 | 1 | 1 | 1 | 3 | 2 | 1 | 2 | 3 |
| 16 | 10 | 10 | 4 | 10 | 8 | 10 | 7 | 11 | 7 | 12 | 9 | 8 |
| 17 | 0 | 0 | 0 | 0 | 0 | 0 | 0 | 0 | 0 | 0 | 0 | 0 |
| 18 | 2 | 5 | 2 | 4 | 2 | 3 | 6 | 2 | 5 | 3 | 3 | 3 |
| 19 | 1 | 2 | 1 | 0 | 0 | 0 | 1 | 0 | 0 | 0 | 0 | 1 |
| 20 | 8 | 4 | 10 | 10 | 6 | 5 | 12 | 7 | 6 | 3 | 4 | 12 |
| 21 | 1 | 3 | 3 | 5 | 5 | 3 | 2 | 2 | 5 | 7 | 1 | 3 |
| 22 | 14 | 8 | 24 | 11 | 12 | 7 | 10 | 16 | 12 | 11 | 11 | 10 |
| 23 |  |  |  |  |  |  |  |  |  |  |  |  |
| 24 | 7 | 4 | 5 | 10 | 6 | 3 | 3 | 1 | 3 | 3 | 3 | 3 |
| 25 | 7 | 6 | 8 | 9 | 5 | 5 | 10 | 8 | 3 | 7 | 14 | 6 |
| 26 | 3 | 1 | 1 | 4 | 3 | 3 | 5 | 2 | 3 | 0 | 1 | 1 |
| 27 | 3 | 1 | 6 | 4 | 4 | 7 | 4 | 5 | 6 | 5 | 5 | 4 |
| 28 | 4 | 1 | 0 | 4 | 2 | 2 | 5 | 5 | 3 | 4 | 5 | 1 |
| 29 | 8 | 13 | 10 |  | 7 | 10 | 10 | 12 | 13 | 9 | 11 | 9 |
| 30 | 1 | 1 | 2 | 3 | 2 | 1 | 1 | 1 | 0 | 1 | 4 | 1 |
| 31 | 4 | 2 | 4 | 3 | 5 | 4 | 1 | 3 | 3 | 5 | 6 | 3 |
| 32 | 1 | 1 | 1 | 1 | 1 | 2 | 1 | 1 | 3 |  | 1 | 1 |
| 33 | 0 | 1 | 0 | 1 | 4 | 0 | 0 | 1 | 0 | 7 | 1 | 3 |
| 34 |  |  |  |  |  |  |  |  |  |  |  |  |
| 35 | 1 | 0 | 0 | 1 | 2 | 1 | 3 | 0 | 1 | 2 | 1 | 1 |
| 36 | 8 | 3 | 4 | 3 | 3 | 9 | 6 | 4 | 2 | 5 | 4 | 3 |
| 37 | 3 | 8 | 1 | 2 | 2 | 3 | 8 | 5 | 5 | 3 | 4 | 2 |
| 38 | 1 | 6 | 0 | 5 | 4 | 6 | 3 | 7 | 6 | 1 | 3 | 5 |
| 39 |  |  |  |  |  |  |  |  |  |  |  |  |
| 40 | 20 | 8 | 9 | 10 | 9 | 19 | 20 | 9 | 10 | 11 | 16 | 15 |
| 41 | 7 | 9 | 2 | 5 | 5 | 2 | 8 | 2 | 4 | 7 | 4 | 6 |
| 42 | 1 | 1 | 4 | 5 | 1 | 4 | 2 | 3 | 1 | 5 | 0 | 4 |
| 43 | 5 | 1 | 3 | 2 | 2 | 5 | 1 | 0 | 3 | 3 | 2 | 1 |
| 44 | 0 | 1 | 1 | 0 | 0 | 0 | 1 | 0 | 1 | 0 | 0 | 0 |
| 45 | 2 | 2 | 3 | 0 | 4 | 3 | 4 | 1 | 4 | 2 | 4 | 3 |
| 46 | 1 | 1 | 6 | 3 | 1 | 1 | 5 | 9 | 4 | 4 | 5 | 5 |
| 47 |  |  |  |  |  |  |  |  |  |  |  |  |
| 48 | 5 | 10 | 7 | 4 | 3 | 5 | 9 | 4 | 7 | 5 | 2 | 5 |
| 49 | 0 | 2 | 3 | 0 | 1 | 2 | 1 | 1 | 2 | 1 | 2 | 0 |
| 50 | 2 | 0 | 1 | 1 | 1 | 0 | 0 | 2 | 1 | 0 | 1 | 1 |
| 51 | 6 | 2 | 5 | 1 | 4 | 4 | 4 | 7 | 5 | 2 | 5 | 7 |
| 52 | 13 | 8 | 10 | 13 | 14 | 6 | 4 | 15 | 14 | 14 | 16 | 9 |
| 53 | 1 | 2 | 3 | 1 | 2 | 0 | 2 | 3 | 3 | 1 | 1 | 2 |
| 54 | 9 | 14 | 13 | 17 | 12 | 8 | 11 | 22 | 9 | 15 | 16 | 18 |
| 55 | 2 | 1 | 1 | 1 | 3 | 0 | 2 | 4 | 2 | 0 | 1 | 2 |
| 56 | 5 | 3 | 2 | 2 | 1 | 4 | 4 | 9 | 3 | 10 | 10 | 3 |
| 57 | 6 | 6 | 5 | 8 | 9 | 5 | 9 | 3 | 7 | 10 | 9 | 11 |
| 58 | 3 | 10 | 9 | 6 | 11 | 5 | 5 | 10 | 5 | 4 | 10 | 5 |
| 59 | 4 | 5 | 5 | 6 | 4 | 3 | 4 | 6 | 7 | 7 | 3 | 6 |
| 60 | 3 | 2 | 0 | 3 | 4 | 2 | 1 | 0 | 2 | 2 | 0 | 1 |
| 61 | 0 | 1 | 1 | 0 | 1 | 0 | 0 | 1 | 1 | 3 | 1 | 0 |
| 62 | 12 | 12 | 8 | 8 | 12 | 17 | 16 | 20 | 10 | 9 | 15 | 16 |
| 63 |  | 2 |  | 3 | 1 |  | 4 | 2 |  | 1 | 1 | 1 |
| 64 |  |  |  |  |  |  |  |  |  |  |  |  |
| 65 | 0 | 4 | 3 | 0 | 1 | 2 | 3 | 2 | 5 | 4 | 2 | 4 |
| 66 |  |  |  |  |  |  |  |  |  |  |  |  |
| 67 | 1 | 1 | 2 | 1 | 1 | 1 | 0 | 1 | 1 | 2 | 0 | 0 |
| 68 | 3 | 3 | 4 | 9 | 2 | 1 | 3 | 3 | 1 | 1 | 2 | 2 |
| 68 | 0 | 0 | 0 | 0 | 0 | 0 | 0 | 0 | 0 | 1 | 0 | 0 |
| 70 | 3 | 5 | 0 | 3 | 2 | 1 | 4 | 1 | 0 | 4 | 0 | 4 |
| 71 | 3 | 1 | 2 | 1 | 2 | 1 | 1 | 2 | 3 | 1 | 3 | 3 |
| 72 | 5 | 8 | 10 | 7 | 10 | 9 | 7 | 4 | 10 | 5 | 6 | 9 |
| 73 | 2 | 1 | 2 | 2 | 1 | 1 | 1 | 3 | 3 | 5 | 3 | 1 |
| 74 | 12 | 15 | 11 | 8 | 7 | 10 | 8 | 11 | 10 | 6 | 15 | 13 |
| 75 |  |  |  |  |  |  |  |  |  |  |  |  |
| 76 | 11 | 10 | 14 | 13 | 11 | 9 | 10 | 17 | 12 | 8 | 17 | 10 |
| 77 | 0 | 0 | 0 | 0 | 0 | 0 | 0 | 0 | 0 | 0 | 0 | 0 |
| 78 |  |  |  |  |  |  |  |  |  |  |  |  |
| 79 | 3 | 3 | 1 | 3 | 3 | 1 | 3 | 1 | 3 | 7 | 2 | 1 |
| 80 | 0 | 1 | 1 | 0 | 1 | 1 | 0 | 2 | 2 | 2 | 1 | 1 |
| 81 | 2 | 1 | 1 | 3 | 4 | 3 |  | 3 | 2 | 4 | 1 | 1 |
| 82 |  |  |  |  |  |  |  |  |  |  |  |  |
| 83 |  |  |  |  |  |  |  |  |  |  |  |  |
| 84 | 7 | 8 | 10 | 9 | 8 | 8 | 12 | 14 | 12 | 7 | 7 | 11 |
| 85 | 2 | 0 | 1 | 1 | 2 | 1 | 0 | 3 | 1 | 2 | 2 | 0 |
| 86 | 7 | 1 | 2 | 0 | 3 | 1 | 4 | 4 | 7 | 10 | 4 | 2 |
| 87 | 1 |  | 5 | 9 | 6 | 5 | 1 | 1 | 1 | 4 | 5 | 5 |
| 88 | 1 | 2 | 1 | 0 | 0 | 0 | 1 | 0 | 1 | 3 | 0 | 1 |
| 89 | 4 | 2 | 6 | 2 | 2 | 0 | 1 | 0 | 1 | 3 | 2 | 1 |
| 90 | 5 | 2 | 2 | 5 | 4 | 5 | 3 | 10 | 6 | 5 | 7 | 5 |
| 91 | 2 | 3 | 7 | 4 | 2 | 5 | 6 | 6 | 6 | 8 | 4 | 3 |
| 92 | 3 | 2 | 0 | 1 | 0 | 2 | 3 | 2 | 0 | 0 | 3 | 1 |
| 93 |  |  |  |  |  |  |  |  |  |  |  |  |
| 94 | 2 | 2 | 6 | 2 | 1 | 1 | 2 | 1 | 2 | 2 | 2 | 2 |
| 95 | 1 | 2 | 1 | 2 | 2 | 4 | 3 | 0 | 1 | 1 | 1 | 0 |
| 96 | 0 | 0 | 1 | 0 | 1 | 0 | 0 | 1 | 0 | 0 | 0 | 1 |
| 97 |  |  |  |  |  |  |  |  |  |  |  |  |
| 98 | 4 | 1 | 2 | 2 | 2 | 2 | 1 | 6 |  |  |  |  |
| 99 | 5 | 2 | 2 | 3 | 2 | 1 | 0 | 3 | 1 | 7 | 1 | 1 |
| 100 | 12 | 16 | 8 | 10 | 5 | 3 | 11 | 11 | 13 | 10 | 9 | 8 |
| 101 | 10 | 10 | 10 | 11 | 8 | 12 | 10 | 7 | 12 | 8 | 11 | 16 |
| 102 |  |  |  |  |  |  |  |  |  |  |  |  |
| 103 | 12 | 13 | 12 | 16 | 17 | 12 | 14 | 15 | 15 | 12 | 15 | 22 |
| 104 | 8 | 12 | 16 | 11 | 9 | 7 | 8 | 13 | 6 | 15 | 11 | 7 |
| 105 | 3 | 3 | 5 | 1 | 1 | 5 | 2 | 3 | 4 | 5 | 9 | 2 |

Table 42. Number of newly diagnosed cervical cancer during each month in 2020.

|  | Month | | | | | | | | | | | |
| --- | --- | --- | --- | --- | --- | --- | --- | --- | --- | --- | --- | --- |
| Hospital | 1 | 2 | 3 | 4 | 5 | 6 | 7 | 8 | 9 | 10 | 11 | 12 |
| 1 | 1 | 1 | 0 | 3 | 2 | 0 | 1 | 1 | 0 | 2 | 0 | 0 |
| 2 | 12 | 9 | 11 | 19 | 9 | 12 | 19 | 15 | 16 | 16 | 28 | 17 |
| 3 | 1 | 2 | 3 | 3 | 0 | 3 | 0 | 2 | 4 | 4 | 3 | 3 |
| 4 | 8 | 10 | 14 | 10 | 9 | 10 | 14 | 9 | 19 | 21 | 16 | 15 |
| 5 | 1 | 0 | 0 | 1 | 1 | 1 | 0 | 1 | 1 | 0 | 4 | 1 |
| 6 | 12 | 10 | 6 | 8 | 2 | 9 | 5 | 4 | 4 | 6 | 9 | 9 |
| 7 | 8 | 7 | 5 | 4 | 3 | 6 | 8 | 3 | 3 | 8 | 7 | 7 |
| 8 |  |  |  |  |  |  |  |  |  |  |  |  |
| 9 | 0 | 0 | 0 | 0 | 0 | 1 | 0 | 1 | 2 | 0 | 3 | 2 |
| 10 | 1 | 2 |  |  | 3 | 2 | 1 |  |  | 2 |  |  |
| 11 | 4 | 1 | 3 | 2 | 3 | 9 | 1 | 4 | 2 | 4 | 2 | 4 |
| 12 | 1 | 0 | 0 | 0 | 0 | 0 | 0 | 0 | 0 | 1 | 1 | 0 |
| 13 | 8 | 9 | 6 | 8 | 11 | 8 | 6 | 8 | 12 | 5 | 9 | 12 |
| 14 | 5 | 6 | 3 | 4 | 2 | 7 | 2 | 7 | 5 | 2 | 3 | 1 |
| 15 | 6 | 5 | 3 | 5 | 5 | 5 | 2 | 5 | 4 | 5 | 6 | 6 |
| 16 | 3 | 3 | 6 | 5 | 4 | 3 | 6 | 4 | 6 | 5 | 7 | 3 |
| 17 | 0 | 0 | 0 | 0 | 0 | 0 | 0 | 0 | 0 | 0 | 0 | 0 |
| 18 | 2 | 2 | 4 | 4 | 4 | 7 | 3 | 2 | 2 | 4 | 3 | 7 |
| 19 | 0 | 1 | 0 | 1 | 0 | 0 | 2 | 2 | 0 | 1 | 0 | 0 |
| 20 | 8 | 5 | 10 | 2 | 7 | 8 | 3 | 4 | 8 | 4 | 5 | 11 |
| 21 | 2 | 1 | 2 | 3 | 4 | 11 | 8 | 4 | 4 | 11 | 7 | 9 |
| 22 | 12 | 9 | 10 | 13 | 4 | 10 | 11 | 6 | 6 | 17 | 14 | 10 |
| 23 |  |  |  |  |  |  |  |  |  |  |  |  |
| 24 | 2 | 3 | 7 | 0 | 3 | 1 | 2 | 3 | 3 | 4 | 4 | 1 |
| 25 | 5 | 3 | 6 | 5 | 6 | 12 | 6 | 5 | 4 | 11 | 7 | 7 |
| 26 | 1 | 1 | 4 | 3 | 3 | 2 | 4 | 1 | 3 | 4 | 1 | 3 |
| 27 | 1 | 1 | 5 | 3 | 5 | 11 | 3 | 5 | 5 | 2 | 2 | 6 |
| 28 | 3 | 2 | 2 | 6 | 2 | 4 | 3 | 4 | 3 | 4 | 6 | 9 |
| 29 | 16 | 10 | 19 |  | 12 | 10 | 10 | 11 | 10 | 13 | 21 | 18 |
| 30 | 2 | 4 | 2 | 2 | 2 | 2 | 1 | 1 | 5 | 2 | 5 | 3 |
| 31 | 2 | 8 | 14 | 8 | 3 | 0 | 5 | 3 | 7 | 5 | 3 | 5 |
| 32 | 1 | 2 | 1 | 1 | 2 | 2 | 3 | 4 |  | 4 | 2 | 1 |
| 33 | 2 | 0 | 1 | 1 | 2 | 1 | 1 | 2 | 0 | 1 | 2 | 1 |
| 34 |  |  |  |  |  |  |  |  |  |  |  |  |
| 35 | 2 | 3 | 1 | 2 | 3 | 1 | 1 | 0 | 1 | 1 | 0 | 0 |
| 36 | 8 | 3 | 5 | 4 | 1 | 3 | 4 | 2 | 2 | 1 | 2 | 4 |
| 37 | 2 | 3 | 5 | 1 | 2 | 1 | 8 | 3 | 2 | 6 | 3 | 5 |
| 38 | 5 | 3 | 2 | 4 | 4 | 3 | 6 | 3 | 4 | 2 | 4 | 1 |
| 39 |  |  |  |  |  |  |  |  |  |  |  |  |
| 40 | 7 | 10 | 5 | 18 | 8 | 14 | 15 | 12 | 13 | 13 | 13 | 5 |
| 41 | 4 | 11 | 4 | 3 | 5 | 5 | 4 | 2 | 5 | 4 | 5 | 3 |
| 42 | 2 | 4 | 2 | 2 | 1 | 2 | 2 | 1 | 2 | 1 | 0 | 1 |
| 43 | 4 | 2 | 1 | 0 | 2 | 2 | 3 | 2 | 3 | 0 | 1 | 1 |
| 44 | 0 | 0 | 0 | 1 | 1 | 1 | 1 | 1 | 0 | 0 | 0 | 0 |
| 45 | 4 | 2 | 5 | 1 | 4 | 4 | 2 | 3 | 2 | 2 | 2 | 2 |
| 46 | 5 | 3 | 3 | 2 | 5 | 4 | 1 | 5 | 3 | 2 | 3 | 5 |
| 47 |  |  |  |  |  |  |  |  |  |  |  |  |
| 48 | 4 | 2 | 3 | 3 | 3 | 4 | 7 | 3 | 8 | 5 | 5 | 0 |
| 49 | 0 | 1 | 1 | 2 | 1 | 0 | 0 | 0 | 0 | 0 | 0 | 0 |
| 50 | 1 | 2 | 0 | 0 | 2 | 2 | 1 | 1 | 0 | 2 | 1 | 1 |
| 51 | 0 | 8 | 4 | 6 | 2 | 5 | 5 | 6 | 6 | 3 | 3 | 2 |
| 52 | 16 | 7 | 11 | 11 | 6 | 12 | 9 | 12 | 12 | 17 | 12 | 8 |
| 53 | 0 | 0 | 2 | 3 | 2 | 5 | 3 | 3 | 0 | 1 | 1 | 2 |
| 54 | 18 | 16 | 7 | 12 | 10 | 11 | 10 | 13 | 15 | 13 | 16 | 10 |
| 55 | 2 | 1 | 4 | 1 | 2 | 3 | 1 | 0 | 2 | 3 | 5 | 4 |
| 56 | 5 | 5 | 5 | 6 | 3 | 4 | 14 | 7 | 5 | 5 | 7 | 3 |
| 57 | 7 | 5 | 8 | 4 | 6 | 4 | 5 | 3 | 6 | 10 | 11 | 3 |
| 58 | 13 | 5 | 9 | 9 | 7 | 3 | 5 | 7 | 8 | 8 | 6 | 6 |
| 59 | 9 | 7 | 6 | 11 | 1 | 3 | 4 | 7 | 4 | 4 | 1 | 2 |
| 60 | 2 | 2 | 2 | 4 | 0 | 1 | 3 | 2 | 2 | 1 | 2 | 1 |
| 61 | 1 | 0 | 0 | 0 | 0 | 0 | 0 | 0 | 0 | 0 | 0 | 0 |
| 62 | 12 | 13 | 7 | 12 | 19 | 5 | 8 | 5 | 7 | 18 | 14 | 8 |
| 63 | 3 | 1 |  | 3 | 1 |  | 2 |  |  |  | 1 |  |
| 64 |  |  |  |  |  |  |  |  |  |  |  |  |
| 65 | 1 | 2 | 0 | 1 | 0 | 2 | 3 | 0 | 1 | 0 | 1 | 1 |
| 66 |  |  |  |  |  |  |  |  |  |  |  |  |
| 67 | 2 | 0 | 0 | 0 | 0 | 0 | 1 | 0 | 1 | 1 | 0 | 2 |
| 68 | 2 | 2 | 1 | 1 | 1 | 2 | 3 | 4 | 1 | 0 | 2 | 4 |
| 68 | 0 | 0 | 1 | 1 | 0 | 0 | 0 | 0 | 0 | 0 | 0 | 0 |
| 70 | 3 | 6 | 0 | 5 | 3 | 1 | 4 | 0 | 0 | 6 | 1 | 1 |
| 71 | 6 | 0 | 3 | 2 | 1 | 1 | 1 | 1 | 1 | 2 | 2 | 1 |
| 72 | 8 | 9 | 8 | 6 | 5 | 10 | 10 | 7 | 10 | 10 | 7 | 8 |
| 73 | 2 | 2 | 1 | 3 | 4 | 3 | 2 | 1 | 1 | 1 | 0 | 2 |
| 74 | 8 | 9 | 11 | 14 | 14 | 7 | 10 | 10 | 4 | 9 | 9 | 6 |
| 75 |  |  |  |  |  |  |  |  |  |  |  |  |
| 76 | 17 | 15 | 18 | 9 | 7 | 16 | 12 | 10 | 16 | 18 | 11 | 16 |
| 77 | 0 | 0 | 0 | 0 | 0 | 0 | 0 | 1 | 0 | 0 | 0 | 0 |
| 78 |  |  |  |  |  |  |  |  |  |  |  |  |
| 79 | 3 |  | 4 | 4 | 1 | 2 |  |  | 3 | 3 | 4 | 1 |
| 80 | 0 | 3 | 0 | 1 | 2 | 1 | 1 | 0 | 1 | 4 | 0 | 1 |
| 81 | 1 |  | 2 | 1 | 4 | 3 | 1 | 2 |  | 1 | 2 |  |
| 82 |  |  |  |  |  |  |  |  |  |  |  |  |
| 83 |  |  |  |  |  |  |  |  |  |  |  |  |
| 84 | 14 | 8 | 10 | 14 | 5 | 13 | 12 | 10 | 8 | 21 | 8 | 15 |
| 85 | 3 | 1 | 0 | 0 | 0 | 0 | 1 | 1 | 0 | 2 | 2 | 1 |
| 86 | 4 | 1 | 5 | 4 | 2 | 8 | 6 | 11 | 7 | 12 | 7 | 3 |
| 87 | 4 | 4 | 2 | 1 |  | 5 | 3 | 2 | 1 | 1 | 2 |  |
| 88 | 0 | 0 | 2 | 1 | 2 | 1 | 1 | 0 | 0 | 5 | 2 | 1 |
| 89 | 3 | 1 | 2 | 2 | 6 | 3 | 3 | 1 | 4 | 3 | 3 | 3 |
| 90 | 6 | 2 | 4 | 3 | 3 | 4 | 8 | 4 | 6 | 3 | 8 | 6 |
| 91 | 8 | 4 | 2 | 4 | 5 | 3 | 4 | 3 | 9 | 4 | 3 | 8 |
| 92 | 0 | 3 | 1 | 1 | 2 | 1 | 0 | 2 | 1 | 1 | 2 | 1 |
| 93 |  |  |  |  |  |  |  |  |  |  |  |  |
| 94 | 1 | 1 | 3 | 1 | 3 | 3 | 3 | 4 | 1 | 1 | 1 | 1 |
| 95 | 0 | 3 | 2 | 0 | 1 | 0 | 3 | 2 | 1 | 1 | 1 | 0 |
| 96 | 0 | 0 | 3 | 0 | 0 | 2 | 1 | 1 | 1 | 0 | 1 | 1 |
| 97 |  |  |  |  |  |  |  |  |  |  |  |  |
| 98 |  |  |  |  |  |  |  |  |  |  |  |  |
| 99 | 4 | 4 | 3 | 4 | 2 | 2 | 6 | 3 | 1 | 0 | 3 | 5 |
| 100 | 9 | 11 | 13 | 7 | 4 | 13 | 8 | 10 | 9 | 9 | 6 | 7 |
| 101 | 11 | 14 | 9 | 9 | 7 | 9 | 12 | 11 | 10 | 8 | 10 | 14 |
| 102 |  |  |  |  |  |  |  |  |  |  |  |  |
| 103 | 15 | 13 | 9 | 13 | 8 | 14 | 19 | 11 | 6 | 14 | 12 | 15 |
| 104 | 5 | 8 | 8 | 6 | 6 | 8 | 6 | 7 | 8 | 16 | 7 | 10 |
| 105 | 2 | 3 | 1 | 3 | 3 | 4 | 7 | 1 | 4 | 3 | 2 | 3 |

Table 43. Number of newly diagnosed stage I cervical cancer during each month in 2019.

|  | Month | | | | | | | | | | | |
| --- | --- | --- | --- | --- | --- | --- | --- | --- | --- | --- | --- | --- |
| Hospital | 1 | 2 | 3 | 4 | 5 | 6 | 7 | 8 | 9 | 10 | 11 | 12 |
| 1 | 0 | 0 | 0 | 0 | 0 | 0 | 0 | 0 | 0 | 0 | 0 | 0 |
| 2 | 16 | 12 | 10 | 7 | 6 | 10 | 5 | 5 | 5 | 13 | 6 | 9 |
| 3 | 1 | 0 | 1 | 0 | 0 | 0 | 0 | 0 | 1 | 2 | 0 | 0 |
| 4 | 1 | 3 | 3 | 4 | 4 | 3 | 2 | 2 | 4 | 2 | 1 | 0 |
| 5 | 0 | 1 | 0 | 0 | 0 | 0 | 0 | 0 | 0 | 0 | 1 | 0 |
| 6 |  | 1 | 1 |  | 2 | 1 |  |  |  |  |  | 1 |
| 7 | 0 | 0 | 0 | 0 | 0 | 2 | 0 | 0 | 0 | 0 | 0 | 0 |
| 8 |  |  |  |  |  |  |  |  |  |  |  |  |
| 9 | 0 | 0 | 0 | 0 | 0 | 2 | 1 | 0 | 2 | 0 | 0 | 2 |
| 10 | 1 | 1 | 1 | 0 | 0 | 0 | 2 | 0 | 0 | 0 | 0 | 0 |
| 11 | 0 | 1 | 1 | 0 | 1 | 0 | 3 | 1 | 1 | 1 | 1 | 1 |
| 12 | 0 | 0 | 0 | 1 | 1 | 0 | 0 | 0 | 0 | 0 | 1 | 1 |
| 13 | 1 | 2 | 3 | 1 | 2 | 2 | 1 | 2 | 1 | 1 | 0 | 1 |
| 14 | 0 | 0 | 0 | 1 | 1 | 0 | 1 | 0 | 1 | 0 | 2 | 1 |
| 15 | 0 | 0 | 0 | 2 | 1 | 0 | 1 | 0 | 2 | 0 | 2 | 1 |
| 16 | 3 | 2 | 2 | 2 | 1 | 0 | 1 | 1 | 1 | 3 | 1 | 2 |
| 17 | 0 | 0 | 0 | 0 | 0 | 0 | 0 | 0 | 0 | 0 | 0 | 0 |
| 18 | 0 | 0 | 1 | 1 | 0 | 0 | 3 | 0 | 0 | 0 | 0 | 0 |
| 19 | 0 | 1 | 1 | 0 | 0 | 0 | 1 | 0 | 0 | 0 | 0 | 0 |
| 20 | 1 | 0 | 0 | 4 | 1 | 1 | 5 | 2 | 3 | 1 | 1 | 2 |
| 21 | 0 | 1 | 0 | 0 | 0 | 0 | 0 | 0 | 4 | 2 | 0 | 2 |
| 22 | 2 | 1 | 2 | 1 | 2 | 1 | 1 | 3 | 1 |  | 1 | 3 |
| 23 |  |  |  |  |  |  |  |  |  |  |  |  |
| 24 | 5 | 2 | 1 | 2 | 3 | 2 | 2 | 0 | 1 | 1 | 1 | 1 |
| 25 | 2 | 1 | 1 | 3 | 0 | 0 | 3 | 1 | 1 | 1 | 6 | 4 |
| 26 |  | 1 |  | 2 | 3 | 1 | 1 |  | 3 |  | 1 |  |
| 27 | 1 | 1 | 1 | 1 | 1 | 0 | 3 | 0 | 1 | 2 | 1 | 0 |
| 28 | 3 | 1 | 0 | 0 | 0 | 0 | 0 | 1 | 0 | 2 | 1 | 0 |
| 29 |  |  |  |  |  |  |  |  |  |  |  |  |
| 30 | 1 | 1 | 1 | 0 | 0 | 0 | 0 | 0 | 0 | 0 | 4 | 1 |
| 31 | 0 | 0 | 0 | 1 | 1 | 1 | 0 | 1 | 0 | 2 | 0 | 1 |
| 32 | 1 | 1 | 1 |  | 1 | 1 | 1 | 1 | 3 |  | 1 | 1 |
| 33 | 0 | 0 | 0 | 1 | 0 | 0 | 0 | 1 | 0 | 5 | 1 | 1 |
| 34 |  |  |  |  |  |  |  |  |  |  |  |  |
| 35 | 0 | 0 | 0 | 0 | 0 | 1 | 2 | 0 | 1 | 2 | 1 | 1 |
| 36 | 6 |  |  | 1 | 1 | 3 | 2 | 2 | 1 | 1 | 1 | 1 |
| 37 | 2 | 6 | 1 | 0 | 1 | 2 | 2 | 0 | 1 | 0 | 2 | 1 |
| 38 |  |  |  |  |  |  |  |  |  |  |  |  |
| 39 |  |  |  |  |  |  |  |  |  |  |  |  |
| 40 | 3 | 1 | 2 | 3 | 0 | 3 | 4 | 0 | 4 | 4 | 1 | 4 |
| 41 | 3 | 3 | 1 | 2 | 3 | 1 | 1 | 1 | 0 | 1 | 1 | 1 |
| 42 | 0 | 0 | 2 | 2 | 0 | 1 | 0 | 1 | 1 | 1 | 0 | 1 |
| 43 | 1 | 0 | 0 | 0 | 0 | 2 | 0 | 0 | 2 | 0 | 0 | 0 |
| 44 | 0 | 1 | 0 | 0 | 0 | 0 | 0 | 0 | 0 | 0 | 0 | 0 |
| 45 | 1 | 0 | 0 | 0 | 0 | 0 | 1 | 0 | 0 | 0 | 0 | 0 |
| 46 | 0 | 0 | 0 | 0 | 0 | 0 | 0 | 0 | 1 | 0 | 0 | 0 |
| 47 |  |  |  |  |  |  |  |  |  |  |  | 1 |
| 48 | 2 | 4 | 2 | 0 | 0 | 2 | 2 | 3 | 2 | 0 | 0 | 0 |
| 49 | 0 | 0 | 2 | 0 | 1 | 1 | 1 | 0 | 2 | 0 | 2 | 0 |
| 50 | 0 | 0 | 0 | 0 | 0 | 0 | 0 | 0 | 0 | 0 | 0 | 0 |
| 51 | 1 | 0 | 0 | 0 | 1 | 2 | 1 | 1 | 2 | 1 | 0 | 0 |
| 52 | 3 | 5 | 1 | 2 | 2 | 1 | 2 | 4 | 1 | 5 | 6 | 2 |
| 53 | 1 | 1 | 1 | 0 | 1 | 0 | 0 | 1 | 2 | 0 | 1 | 1 |
| 54 | 3 | 4 | 2 | 5 | 3 | 2 | 2 | 2 | 2 | 1 | 5 | 6 |
| 55 | 0 | 0 | 0 | 0 | 0 | 0 | 0 | 0 | 0 | 0 | 0 | 0 |
| 56 | 0 | 1 | 1 | 1 | 0 | 1 | 2 | 0 | 1 | 2 | 5 | 0 |
| 57 | 2 | 0 | 3 | 4 | 6 | 4 | 2 | 1 | 1 | 3 | 4 | 6 |
| 58 | 0 | 1 | 1 | 0 | 3 | 0 | 1 | 3 | 1 | 1 | 2 | 1 |
| 59 | 3 | 1 | 3 | 3 | 2 | 1 | 2 | 4 | 4 | 2 | 2 | 2 |
| 60 | 2 | 1 | 0 | 1 | 1 | 2 | 1 | 0 | 0 | 0 | 0 | 0 |
| 61 | 0 | 1 | 1 | 0 | 1 | 0 | 0 | 0 | 0 | 1 | 0 | 0 |
| 62 |  |  |  |  |  |  |  |  |  |  |  |  |
| 63 |  | 2 |  | 2 |  |  |  |  |  | 1 | 1 |  |
| 64 | 3 | 2 | 4 | 1 | 1 | 2 |  | 1 | 4 | 1 | 3 | 4 |
| 65 | 0 | 1 | 1 | 0 | 0 | 1 | 1 | 2 | 0 | 3 | 1 | 2 |
| 66 |  |  |  |  |  |  |  |  |  |  |  |  |
| 67 | 1 |  | 2 |  |  |  |  |  |  | 2 |  |  |
| 68 | 1 | 1 | 0 | 1 | 1 | 0 | 0 | 0 | 0 | 0 | 0 | 0 |
| 68 | 0 | 0 | 0 | 0 | 0 | 0 | 0 | 0 | 0 | 1 | 0 | 0 |
| 70 |  |  |  |  |  |  |  |  |  | 1 |  |  |
| 71 | 2 | 1 | 1 | 0 | 2 | 1 | 1 | 0 | 1 | 0 | 2 | 1 |
| 72 | 0 | 0 | 1 | 2 | 0 | 2 | 0 | 0 | 2 | 3 | 2 | 1 |
| 73 | 1 | 0 | 1 | 1 | 0 | 0 | 0 | 1 | 2 | 1 | 2 | 0 |
| 74 | 6 | 10 | 7 | 3 | 1 | 8 | 5 | 6 | 7 | 3 | 6 | 8 |
| 75 |  |  |  |  |  |  |  |  |  |  |  |  |
| 76 | 0 | 1 | 5 | 0 | 1 | 3 | 4 | 7 | 1 | 1 | 6 | 2 |
| 77 |  |  |  |  |  |  |  |  |  |  |  |  |
| 78 |  |  |  |  |  |  |  |  |  |  |  |  |
| 79 | 0 | 1 | 0 | 1 | 0 | 1 | 0 | 0 | 0 | 1 | 1 | 1 |
| 80 | 0 | 0 | 0 | 0 | 0 | 0 | 0 | 1 | 1 | 0 | 0 | 0 |
| 81 | 1 | 1 | 1 | 2 | 1 | 1 |  | 1 | 1 |  |  |  |
| 82 |  |  |  |  |  |  |  |  |  |  |  |  |
| 83 | 0 | 0 | 0 | 0 | 0 | 1 | 1 | 0 | 0 | 0 | 0 | 1 |
| 84 | 2 | 0 | 2 | 1 | 1 | 0 | 3 | 1 | 1 | 3 | 2 | 1 |
| 85 | 1 |  | 1 |  | 1 |  |  | 1 |  | 1 |  |  |
| 86 | 4 | 1 | 2 | 0 | 0 | 0 | 2 | 0 | 1 | 2 | 3 | 1 |
| 87 |  |  | 1 | 1 |  |  |  |  |  |  | 1 | 1 |
| 88 | 1 | 0 | 1 | 0 | 0 | 0 | 0 | 0 | 0 | 2 | 0 | 1 |
| 89 | 1 | - | 1 | 1 | 1 | - | 1 | - | - | 1 | 1 | 1 |
| 90 | 0 | 0 | 1 | 1 | 1 | 0 | 0 | 3 | 1 | 1 | 2 | 1 |
| 91 | 0 | 0 | 0 | 2 | 2 | 0 | 1 | 0 | 0 | 1 | 1 | 1 |
| 92 | 1 | 1 | 0 | 1 | 0 | 0 | 1 | 1 | 0 | 0 | 2 | 0 |
| 93 | 0 | 1 | 1 | 0 | 0 | 0 | 1 | 0 | 0 | 0 | 0 | 0 |
| 94 | 1 | 1 | 0 | 0 | 0 | 0 | 0 | 1 | 1 | 0 | 0 | 0 |
| 95 | 0 | 0 | 0 | 0 | 0 | 0 | 0 | 0 | 0 | 0 | 0 | 0 |
| 96 |  |  |  |  |  |  |  | 1 |  |  |  |  |
| 97 |  |  |  |  |  |  |  |  |  |  |  |  |
| 98 | 4 | 0 | 1 | 1 | 1 | 2 | 1 | 2 | 1 | 0 | 2 | 1 |
| 99 | 2 | 1 | 0 | 0 | 1 | 0 | 0 | 2 | 0 | 0 | 0 | 0 |
| 100 | 11 | 15 | 6 | 3 | 3 | 2 | 8 | 9 | 12 | 8 | 7 | 6 |
| 101 | 1 |  |  |  | 2 |  |  |  | 1 |  | 1 | 1 |
| 102 |  |  |  |  |  |  |  |  |  |  |  |  |
| 103 | 1 | 0 | 0 | 1 | 0 | 0 | 1 | 1 | 1 | 2 | 2 | 1 |
| 104 | 1 | 1 | 2 | 1 | 0 | 1 | 2 | 1 | 1 | 4 | 1 | 0 |
| 105 | 0 | 2 | 2 | 0 | 0 | 0 | 0 | 0 | 2 | 0 | 1 | 2 |

Table 44. Number of newly diagnosed stage I cervical cancer during each month in 2020.

|  | Month | | | | | | | | | | | |
| --- | --- | --- | --- | --- | --- | --- | --- | --- | --- | --- | --- | --- |
| Hospital | 1 | 2 | 3 | 4 | 5 | 6 | 7 | 8 | 9 | 10 | 11 | 12 |
| 1 | 1 | 0 | 0 | 0 | 0 | 0 | 0 | 1 | 0 | 1 | 0 | 0 |
| 2 | 6 | 5 | 4 | 10 | 5 | 5 | 8 | 9 | 7 | 6 | 17 | 11 |
| 3 | 0 | 0 | 0 | 1 | 0 | 0 | 0 | 1 | 1 | 0 | 0 | 0 |
| 4 | 1 | 1 | 3 | 2 | 1 | 0 | 3 | 4 | 2 | 1 | 2 | 2 |
| 5 | 0 | 0 | 0 | 1 | 0 | 0 | 0 | 1 | 1 | 0 | 3 | 0 |
| 6 | 1 | 1 | 1 | 2 | 1 | 1 | 1 |  |  | 1 | 2 | 1 |
| 7 | 0 | 1 | 0 | 0 | 1 | 1 | 0 | 0 | 0 | 1 | 0 | 0 |
| 8 |  |  |  |  |  |  |  |  |  |  |  |  |
| 9 | 0 | 0 | 0 | 0 | 0 | 0 | 0 | 1 | 0 | 0 | 1 | 0 |
| 10 | 0 | 1 | 0 | 0 | 0 | 2 | 0 | 0 | 0 | 1 | 0 | 0 |
| 11 | 1 | 0 | 1 | 2 | 0 | 3 | 1 | 1 | 0 | 1 | 1 | 1 |
| 12 | 0 | 0 | 0 | 0 | 0 | 0 | 0 | 0 | 0 | 1 | 1 | 0 |
| 13 | 3 | 4 | 1 | 2 | 1 | 1 | 2 | 2 | 2 | 1 | 0 | 3 |
| 14 | 1 | 1 | 0 | 1 | 0 | 2 | 0 | 3 | 1 | 1 | 1 | 0 |
| 15 | 2 | 2 | 2 | 3 | 3 | 3 | 1 | 2 | 2 | 2 | 0 | 3 |
| 16 | 1 | 3 | 0 | 0 | 2 | 5 | 1 | 0 | 0 | 3 | 2 | 1 |
| 17 | 0 | 0 | 0 | 0 | 0 | 0 | 0 | 0 | 0 | 0 | 0 | 0 |
| 18 | 0 | 0 | 0 | 0 | 0 | 1 | 0 | 1 | 0 | 0 | 0 | 0 |
| 19 | 0 | 1 | 0 | 0 | 0 | 0 | 1 | 1 | 0 | 1 | 0 | 0 |
| 20 | 0 | 2 | 1 | 0 | 2 | 1 | 0 | 0 | 2 | 2 | 4 | 3 |
| 21 | 0 | 0 | 1 | 1 | 1 | 3 | 0 | 1 | 0 | 0 | 0 | 0 |
| 22 | 3 | 1 |  | 1 | 1 |  |  |  | 1 | 1 | 1 | 1 |
| 23 |  |  |  |  |  |  |  |  |  |  |  |  |
| 24 | 1 | 2 | 1 | 0 | 1 | 1 | 1 | 1 | 1 | 2 | 2 | 1 |
| 25 | 0 | 1 | 3 | 0 | 3 | 2 | 2 | 2 | 0 | 3 | 0 | 1 |
| 26 | 1 |  | 3 | 1 | 2 | 2 | 1 | 1 | 2 | 1 |  | 2 |
| 27 | 0 | 1 | 2 | 0 | 1 | 2 | 0 | 1 | 2 | 1 | 0 | 2 |
| 28 | 1 | 0 | 0 | 1 | 0 | 0 | 0 | 0 | 0 | 0 | 2 | 1 |
| 29 |  |  |  |  |  |  |  |  |  |  |  |  |
| 30 | 0 | 3 | 1 | 1 | 0 | 0 | 0 | 0 | 2 | 1 | 3 | 0 |
| 31 | 0 | 1 | 0 | 1 | 1 | 0 | 1 | 0 | 1 | 1 | 1 | 0 |
| 32 | 1 | 2 | 1 |  | 2 |  | 1 | 2 |  | 4 | 1 |  |
| 33 | 1 | 0 | 0 | 1 | 1 | 1 | 1 | 2 | 0 | 0 | 1 | 0 |
| 34 |  |  |  |  |  |  |  |  |  |  |  |  |
| 35 | 0 | 1 | 0 | 1 | 0 | 0 | 0 | 0 | 1 | 1 | 0 | 0 |
| 36 | 4 | 2 | 3 | 2 |  |  | 2 | 2 | 1 | 1 |  | 2 |
| 37 | 2 | 1 | 3 | 1 | 0 | 0 | 4 | 1 | 0 | 2 | 1 | 3 |
| 38 |  |  |  |  |  |  |  |  |  |  |  |  |
| 39 |  |  |  |  |  |  |  |  |  |  |  |  |
| 40 | 2 | 1 | 2 | 4 | 2 | 3 | 1 | 7 | 6 | 3 | 5 | 1 |
| 41 | 0 | 6 | 2 | 1 | 1 | 1 | 2 | 0 | 1 | 1 | 2 | 0 |
| 42 | 1 | 1 | 2 | 0 | 0 | 1 | 0 | 0 | 2 | 1 | 0 | 1 |
| 43 | 0 | 1 | 0 | 0 | 1 | 1 | 3 | 1 | 2 | 0 | 1 | 0 |
| 44 | 0 | 0 | 0 | 0 | 0 | 0 | 0 | 0 | 0 | 0 | 0 | 0 |
| 45 | 0 | 0 | 0 | 0 | 1 | 1 | 1 | 0 | 0 | 0 | 0 | 1 |
| 46 | 0 | 0 | 0 | 0 | 0 | 1 | 1 | 1 | 0 | 0 | 0 | 1 |
| 47 |  |  |  |  | 1 |  | 1 | 1 |  | 1 |  |  |
| 48 | 2 | 0 | 1 | 1 | 0 | 2 | 3 | 2 | 3 | 3 | 1 | 0 |
| 49 | 0 | 0 | 1 | 1 | 0 | 0 | 0 | 0 | 0 | 0 | 0 | 0 |
| 50 | 1 | 0 | 0 | 0 | 1 | 1 | 0 | 0 | 0 | 0 | 0 | 0 |
| 51 | 0 | 4 | 0 | 0 | 0 | 0 | 1 | 0 | 0 | 0 | 0 | 1 |
| 52 | 2 | 2 | 2 | 1 | 2 | 5 | 1 | 2 | 2 | 4 | 4 | 3 |
| 53 | 0 | 0 | 0 | 1 | 0 | 4 | 3 | 0 | 0 | 0 | 0 | 1 |
| 54 | 2 | 2 | 0 | 3 | 2 | 4 | 3 | 2 | 2 | 3 | 5 | 2 |
| 55 | 1 | 1 | 2 | 0 | 0 | 0 | 0 | 0 | 0 | 0 | 1 | 1 |
| 56 | 0 | 1 | 2 | 0 | 0 | 0 | 4 | 1 | 0 | 0 | 3 | 2 |
| 57 | 2 | 2 | 2 | 1 | 4 | 0 | 0 | 0 | 3 | 6 | 5 | 2 |
| 58 | 3 | 1 | 0 | 0 | 1 | 1 | 2 | 3 | 0 | 1 | 0 | 3 |
| 59 | 2 | 3 | 4 | 3 | 1 | 1 | 1 | 3 | 2 | 0 | 1 | 0 |
| 60 | 1 | 1 | 2 | 2 | 0 | 0 | 2 | 1 | 1 | 0 | 2 | 1 |
| 61 | 1 | 0 | 0 | 0 | 0 | 0 | 0 | 0 | 0 | 0 | 0 | 0 |
| 62 |  |  |  |  |  |  |  |  |  |  |  |  |
| 63 | 2 | 1 |  | 1 | 1 |  |  |  |  |  | 1 |  |
| 64 | 3 |  | 5 | 2 | 2 | 3 | 2 |  | 2 |  | 2 | 3 |
| 65 | 1 | 1 | 0 | 0 | 0 | 0 | 3 | 0 | 1 | 0 | 1 | 0 |
| 66 |  |  |  |  |  |  |  |  |  |  |  |  |
| 67 | 1 |  |  |  |  |  |  |  |  | 1 |  |  |
| 68 | 0 | 0 | 0 | 0 | 0 | 0 | 1 | 0 | 0 | 0 | 1 | 1 |
| 68 | 0 | 0 | 0 | 0 | 0 | 0 | 0 | 0 | 0 | 0 | 0 | 0 |
| 70 | 1 |  |  | 2 |  |  |  |  |  |  |  |  |
| 71 | 3 | 0 | 1 | 2 | 0 | 1 | 1 | 1 | 1 | 0 | 1 | 0 |
| 72 | 1 | 1 | 1 | 1 | 0 | 0 | 0 | 1 | 1 | 1 | 1 | 0 |
| 73 | 2 | 0 | 1 | 1 | 1 | 1 | 0 | 0 | 0 | 1 | 0 | 1 |
| 74 | 6 | 4 | 8 | 8 | 9 | 4 | 3 | 6 | 2 | 7 | 7 | 3 |
| 75 |  |  |  |  |  |  |  |  |  |  |  |  |
| 76 | 1 | 2 | 3 | 2 | 1 | 0 | 2 | 2 | 1 | 3 | 2 | 4 |
| 77 |  |  |  |  |  |  |  |  |  |  |  |  |
| 78 |  |  |  |  |  |  |  |  |  |  |  |  |
| 79 | 2 | 0 | 1 | 2 | 1 | 1 | 0 | 0 | 2 | 0 | 1 | 1 |
| 80 | 0 | 0 | 0 | 1 | 2 | 1 | 0 | 0 | 0 | 1 | 0 | 0 |
| 81 | 1 |  |  |  | 1 | 2 | 1 | 1 |  |  | 1 |  |
| 82 |  |  |  |  |  |  |  |  |  |  |  |  |
| 83 | 0 | 0 | 0 | 1 | 0 | 1 | 1 | 0 | 0 | 1 | 0 | 0 |
| 84 | 1 | 1 | 1 | 2 | 0 | 3 | 0 | 0 | 1 | 6 | 1 | 2 |
| 85 |  | 1 |  |  |  |  |  |  |  | 1 |  | 1 |
| 86 | 0 | 0 | 2 | 2 | 2 | 3 | 3 | 5 | 3 | 2 | 3 | 1 |
| 87 | 1 |  |  |  |  |  |  |  |  |  |  |  |
| 88 | 0 | 0 | 2 | 1 | 2 | 0 | 0 | 0 | 0 | 4 | 0 | 0 |
| 89 | - | - | 1 | - | 2 | - | 1 | 1 | 2 | 1 | 2 | - |
| 90 | 0 | 0 | 1 | 0 | 1 | 0 | 1 | 0 | 0 | 0 | 1 | 0 |
| 91 | 1 | 1 | 0 | 2 | 0 | 2 | 1 | 0 | 2 | 1 | 0 | 1 |
| 92 | 0 | 2 | 0 | 0 | 1 | 1 | 0 | 1 | 0 | 0 | 1 | 0 |
| 93 | 0 | 1 | 0 | 0 | 0 | 0 | 0 | 1 | 2 | 2 | 0 | 0 |
| 94 | 0 | 0 | 0 | 0 | 1 | 0 | 0 | 0 | 1 | 1 | 0 | 0 |
| 95 | 0 | 0 | 0 | 0 | 0 | 0 | 0 | 0 | 0 | 0 | 0 | 0 |
| 96 |  |  | 3 |  |  | 1 | 1 |  | 1 |  | 1 |  |
| 97 |  |  |  |  |  |  |  |  |  |  |  |  |
| 98 | 0 | 0 | 3 | 0 | 2 | 1 | 1 | 0 | 0 | 1 | 0 | 1 |
| 99 | 0 | 1 | 1 | 0 | 0 | 1 | 1 | 1 | 0 | 0 | 1 | 1 |
| 100 | 8 | 7 | 10 | 7 | 3 | 12 | 3 | 6 | 6 | 8 | 5 | 7 |
| 101 |  | 2 | 1 | 2 |  | 1 | 2 | 2 |  |  |  |  |
| 102 |  |  |  |  |  |  |  |  |  |  |  |  |
| 103 | 2 | 1 | 0 | 1 | 0 | 0 | 2 | 2 | 0 | 3 | 0 | 1 |
| 104 | 1 | 1 | 1 | 2 | 0 | 1 | 0 | 3 | 0 | 2 | 2 | 0 |
| 105 | 0 | 1 | 0 | 2 | 1 | 0 | 2 | 1 | 0 | 0 | 0 | 1 |

Table 45. Number of newly diagnosed stage II cervical cancer during each month in 2019.

|  | Month | | | | | | | | | | | |
| --- | --- | --- | --- | --- | --- | --- | --- | --- | --- | --- | --- | --- |
| Hospital | 1 | 2 | 3 | 4 | 5 | 6 | 7 | 8 | 9 | 10 | 11 | 12 |
| 1 | 2 | 0 | 0 | 0 | 0 | 0 | 0 | 0 | 0 | 0 | 0 | 0 |
| 2 | 0 | 2 | 3 | 0 | 1 | 3 | 0 | 1 | 2 | 4 | 2 | 0 |
| 3 | 0 | 0 | 0 | 0 | 0 | 1 | 1 | 1 | 0 | 0 | 0 | 1 |
| 4 | 0 | 1 | 2 | 1 | 1 | 2 | 3 | 0 | 0 | 1 | 1 | 0 |
| 5 | 0 | 0 | 0 | 0 | 0 | 0 | 0 | 0 | 0 | 0 | 0 | 0 |
| 6 |  |  |  |  |  | 1 | 1 |  |  | 2 |  | 1 |
| 7 | 0 | 0 | 0 | 0 | 0 | 0 | 0 | 0 | 0 | 0 | 0 | 0 |
| 8 |  |  |  |  |  |  |  |  |  |  |  |  |
| 9 | 0 | 0 | 0 | 0 | 1 | 0 | 0 | 0 | 0 | 0 | 0 | 0 |
| 10 | 1 | 0 | 1 | 0 | 2 | 0 | 0 | 1 | 0 | 1 | 1 | 1 |
| 11 | 1 | 3 | 1 | 0 | 1 | 4 | 3 | 0 | 1 | 0 | 0 | 2 |
| 12 | 1 | 0 | 0 | 0 | 0 | 0 | 0 | 0 | 0 | 0 | 0 | 0 |
| 13 | 0 | 1 | 1 | 0 | 0 | 0 | 1 | 0 | 2 | 0 | 0 | 1 |
| 14 | 0 | 0 | 0 | 0 | 0 | 2 | 0 | 0 | 0 | 0 | 0 | 0 |
| 15 | 1 | 1 | 3 | 2 | 0 | 0 | 0 | 1 | 0 | 0 | 0 | 1 |
| 16 | 2 | 0 | 1 | 0 | 2 | 0 | 1 | 1 | 3 | 1 | 0 | 1 |
| 17 | 0 | 0 | 0 | 0 | 0 | 0 | 0 | 0 | 0 | 0 | 0 | 0 |
| 18 | 0 | 0 | 0 | 0 | 0 | 0 | 0 | 0 | 0 | 0 | 0 | 0 |
| 19 | 1 | 0 | 0 | 0 | 0 | 0 | 0 | 0 | 0 | 0 | 0 | 0 |
| 20 | 2 | 0 | 1 | 2 | 1 | 2 | 2 | 3 | 2 | 1 | 0 | 2 |
| 21 | 0 | 1 | 0 | 2 | 0 | 1 | 0 | 0 | 1 | 0 | 0 | 0 |
| 22 |  |  |  |  |  |  |  |  | 1 |  |  |  |
| 23 |  |  |  |  |  |  |  |  |  |  |  |  |
| 24 | 1 | 0 | 1 | 2 | 0 | 1 | 0 | 0 | 0 | 0 | 0 | 1 |
| 25 | 0 | 0 | 1 | 0 | 1 | 1 | 0 | 0 | 0 | 1 | 1 | 0 |
| 26 |  |  | 1 |  |  |  | 1 |  |  |  |  |  |
| 27 | 0 | 0 | 0 | 1 | 0 | 2 | 0 | 2 | 3 | 0 | 1 | 1 |
| 28 | 0 | 0 | 0 | 0 | 0 | 0 | 0 | 1 | 1 | 0 | 0 | 0 |
| 29 |  |  |  |  |  |  |  |  |  |  |  |  |
| 30 | 0 | 0 | 1 | 1 | 1 | 1 | 0 | 0 | 0 | 0 | 0 | 0 |
| 31 | 0 | 0 | 1 | 1 | 0 | 1 | 0 | 0 | 1 | 0 | 2 | 0 |
| 32 |  |  |  |  |  |  |  |  |  |  |  |  |
| 33 | 0 | 1 | 0 | 0 | 0 | 0 | 0 | 0 | 0 | 0 | 0 | 1 |
| 34 |  |  |  |  |  |  |  |  |  |  |  |  |
| 35 | 0 | 0 | 0 | 0 | 0 | 0 | 1 | 0 | 0 | 0 | 0 | 0 |
| 36 | 1 | 1 | 3 |  | 1 | 2 | 2 | 1 | 1 | 2 | 1 |  |
| 37 | 0 | 0 | 0 | 1 | 1 | 0 | 3 | 0 | 0 | 2 | 2 | 0 |
| 38 |  |  |  |  |  |  |  |  |  |  |  |  |
| 39 |  |  |  |  |  |  |  |  |  |  |  |  |
| 40 | 2 | 1 | 0 | 0 | 1 | 3 | 2 | 3 | 0 | 2 | 1 | 1 |
| 41 | 2 | 2 | 0 | 2 | 0 | 0 | 4 | 0 | 0 | 3 | 1 | 1 |
| 42 | 0 | 0 | 2 | 2 | 0 | 1 | 0 | 1 | 0 | 2 | 0 | 1 |
| 43 | 1 | 0 | 0 | 2 | 0 | 0 | 1 | 0 | 1 | 0 | 0 | 0 |
| 44 | 0 | 0 | 0 | 0 | 0 | 0 | 0 | 0 | 1 | 0 | 0 | 0 |
| 45 | 1 | 1 | 0 | 0 | 0 | 1 | 0 | 0 | 1 | 0 | 1 | 2 |
| 46 | 0 | 0 | 1 | 0 | 0 | 0 | 0 | 1 | 0 | 1 | 0 | 1 |
| 47 |  |  |  |  |  |  |  |  |  |  |  |  |
| 48 | 0 | 1 | 2 | 1 | 0 | 1 | 1 | 1 | 3 | 0 | 0 | 2 |
| 49 | 0 | 1 | 0 | 0 | 0 | 0 | 0 | 0 | 0 | 0 | 0 | 0 |
| 50 | 0 | 0 | 0 | 0 | 0 | 0 | 0 | 0 | 0 | 0 | 0 | 0 |
| 51 | 0 | 1 | 0 | 0 | 0 | 0 | 0 | 0 | 0 | 0 | 0 | 0 |
| 52 | 0 | 0 | 0 | 2 | 0 | 1 | 0 | 2 | 1 | 0 | 1 | 0 |
| 53 | 0 | 0 | 2 | 0 | 1 | 0 | 1 | 0 | 0 | 0 | 0 | 0 |
| 54 | 1 | 1 | 0 | 0 | 0 | 1 | 1 | 1 | 0 | 1 | 0 | 0 |
| 55 | 0 | 0 | 0 | 0 | 0 | 0 | 0 | 0 | 0 | 0 | 0 | 0 |
| 56 | 0 | 0 | 0 | 0 | 0 | 0 | 1 | 2 | 1 | 2 | 1 | 0 |
| 57 | 0 | 1 | 1 | 2 | 0 | 0 | 1 | 1 | 2 | 2 | 1 | 1 |
| 58 | 0 | 0 | 2 | 2 | 1 | 0 | 0 | 2 | 0 | 0 | 3 | 0 |
| 59 | 0 | 2 | 0 | 0 | 1 | 0 | 0 | 0 | 2 | 2 | 1 | 0 |
| 60 | 0 | 1 | 0 | 2 | 3 | 0 | 0 | 0 | 0 | 0 | 0 | 0 |
| 61 | 0 | 0 | 0 | 0 | 0 | 0 | 0 | 0 | 1 | 2 | 0 | 0 |
| 62 |  |  |  |  |  |  |  |  |  |  |  |  |
| 63 |  |  |  |  |  |  |  |  |  |  |  |  |
| 64 |  |  |  |  |  |  |  |  |  |  | 3 | 2 |
| 65 | 0 | 1 | 0 | 0 | 0 | 1 | 2 | 0 | 1 | 0 | 0 | 1 |
| 66 |  |  |  |  |  |  |  |  |  |  |  |  |
| 67 |  |  |  |  | 1 |  |  |  |  |  |  |  |
| 68 | 0 | 0 | 0 | 0 | 0 | 0 | 0 | 0 | 0 | 0 | 0 | 0 |
| 68 | 0 | 0 | 0 | 0 | 0 | 0 | 0 | 0 | 0 | 0 | 0 | 0 |
| 70 |  |  |  |  |  |  |  |  |  |  |  |  |
| 71 | 0 | 0 | 1 | 0 | 0 | 0 | 0 | 1 | 0 | 1 | 0 | 1 |
| 72 | 0 | 0 | 0 | 0 | 1 | 0 | 0 | 1 | 0 | 0 | 1 | 1 |
| 73 | 0 | 0 | 1 | 0 | 1 | 0 | 1 | 2 | 0 | 0 | 1 | 1 |
| 74 | 3 | 3 | 1 | 1 | 2 | 0 | 0 | 2 | 1 | 1 | 3 | 1 |
| 75 |  |  |  |  |  |  |  |  |  |  |  |  |
| 76 | 0 | 0 | 0 | 0 | 2 | 2 | 0 | 1 | 0 | 1 | 0 | 2 |
| 77 |  |  |  |  |  |  |  |  |  |  |  |  |
| 78 |  |  |  |  |  |  |  |  |  |  |  |  |
| 79 | 0 | 0 | 0 | 0 | 0 | 0 | 0 | 0 | 0 | 0 | 0 | 0 |
| 80 | 0 | 0 | 1 | 0 | 1 | 0 | 0 | 1 | 0 | 0 | 0 | 0 |
| 81 |  |  |  | 1 | 1 |  |  |  |  | 1 | 1 | 1 |
| 82 |  |  |  |  |  |  |  |  |  |  |  |  |
| 83 | 0 | 0 | 0 | 1 | 2 | 0 | 0 | 0 | 0 | 0 | 0 | 1 |
| 84 | 0 | 1 | 0 | 0 | 0 | 2 | 1 | 0 | 1 | 0 | 0 | 0 |
| 85 |  |  |  |  |  |  |  |  |  |  |  |  |
| 86 | 0 | 0 | 0 | 0 | 0 | 1 | 0 | 0 | 2 | 3 | 0 | 0 |
| 87 |  |  |  |  |  |  |  |  | 1 |  |  |  |
| 88 | 0 | 0 | 0 | 0 | 0 | 0 | 1 | 0 | 0 | 0 | 0 | 0 |
| 89 | - | - | 1 | 1 | 1 | - | - | - | - | 1 | 1 | - |
| 90 | 0 | 0 | 0 | 0 | 0 | 0 | 0 | 0 | 0 | 0 | 0 | 0 |
| 91 | 1 | 0 | 0 | 0 | 0 | 0 | 0 | 0 | 0 | 0 | 0 | 1 |
| 92 | 1 | 1 | 0 | 0 | 0 | 1 | 1 | 1 | 0 | 0 | 0 | 0 |
| 93 | 0 | 0 | 1 | 0 | 0 | 0 | 0 | 0 | 0 | 0 | 0 | 0 |
| 94 | 0 | 0 | 0 | 0 | 0 | 0 | 0 | 0 | 0 | 0 | 0 | 0 |
| 95 | 0 | 0 | 0 | 0 | 0 | 1 | 0 | 0 | 0 | 0 | 0 | 0 |
| 96 |  |  |  |  |  |  |  |  |  |  |  | 1 |
| 97 |  |  |  |  |  |  |  |  |  |  |  |  |
| 98 | 0 | 1 | 1 | 0 | 0 | 0 | 0 | 1 | 0 | 1 | 0 | 0 |
| 99 | 0 | 0 | 1 | 0 | 0 | 0 | 0 | 0 | 0 | 1 | 0 | 1 |
| 100 | 0 | 0 | 1 | 1 | 0 | 0 | 0 | 0 | 0 | 0 | 1 | 0 |
| 101 |  | 1 |  | 2 |  |  |  |  |  |  |  |  |
| 102 |  |  |  |  |  |  |  |  |  |  |  |  |
| 103 | 1 | 0 | 1 | 1 | 1 | 0 | 1 | 0 | 0 | 0 | 0 | 3 |
| 104 | 1 | 0 | 1 | 1 | 0 | 0 | 0 | 0 | 0 | 0 | 0 | 0 |
| 105 | 1 | 0 | 1 | 0 | 0 | 2 | 0 | 1 | 1 | 1 | 2 | 0 |

Table 46. Number of newly diagnosed stage II cervical cancer during each month in 2020.

|  | Month | | | | | | | | | | | |
| --- | --- | --- | --- | --- | --- | --- | --- | --- | --- | --- | --- | --- |
| Hospital | 1 | 2 | 3 | 4 | 5 | 6 | 7 | 8 | 9 | 10 | 11 | 12 |
| 1 | 0 | 0 | 0 | 0 | 0 | 0 | 0 | 0 | 0 | 0 | 0 | 0 |
| 2 | 1 | 1 | 3 | 1 | 1 | 1 | 1 | 1 | 1 | 1 | 3 | 2 |
| 3 | 0 | 0 | 1 | 0 | 0 | 0 | 0 | 0 | 1 | 0 | 0 | 0 |
| 4 | 2 | 2 | 3 | 1 | 1 | 0 | 1 | 1 | 3 | 1 | 0 | 2 |
| 5 | 1 | 0 | 0 | 0 | 0 | 0 | 0 | 0 | 0 | 0 | 0 | 0 |
| 6 | 1 |  |  | 2 |  | 1 |  |  |  |  | 1 | 2 |
| 7 | 0 | 0 | 0 | 0 | 0 | 0 | 0 | 0 | 0 | 0 | 1 | 0 |
| 8 |  |  |  |  |  |  |  |  |  |  |  |  |
| 9 | 0 | 0 | 0 | 0 | 0 | 0 | 0 | 0 | 1 | 0 | 0 | 2 |
| 10 | 1 | 0 | 0 | 0 | 1 | 0 | 1 | 0 | 0 | 0 | 0 | 0 |
| 11 | 2 | 1 | 1 | 0 | 1 | 2 | 0 | 0 | 2 | 2 | 0 | 0 |
| 12 | 1 | 0 | 0 | 0 | 0 | 0 | 0 | 0 | 0 | 0 | 0 | 0 |
| 13 | 0 | 1 | 2 | 0 | 0 | 2 | 0 | 0 | 3 | 1 | 2 | 2 |
| 14 | 0 | 0 | 0 | 1 | 0 | 1 | 0 | 1 | 0 | 0 | 1 | 0 |
| 15 | 1 | 0 | 0 | 0 | 0 | 1 | 0 | 2 | 1 | 0 | 1 | 0 |
| 16 | 0 | 1 | 1 | 1 | 0 | 0 | 2 | 1 | 1 | 0 | 2 | 1 |
| 17 | 0 | 0 | 0 | 0 | 0 | 0 | 0 | 0 | 0 | 0 | 0 | 0 |
| 18 | 0 | 0 | 0 | 0 | 0 | 0 | 0 | 0 | 0 | 0 | 0 | 0 |
| 19 | 0 | 0 | 0 | 0 | 0 | 0 | 1 | 0 | 0 | 0 | 0 | 0 |
| 20 | 3 | 0 | 2 | 1 | 2 | 2 | 0 | 1 | 2 | 0 | 0 | 4 |
| 21 | 0 | 0 | 0 | 0 | 0 | 0 | 0 | 0 | 0 | 0 | 1 | 1 |
| 22 |  |  | 1 | 1 |  | 1 | 1 |  |  | 1 | 1 |  |
| 23 |  |  |  |  |  |  |  |  |  |  |  |  |
| 24 | 0 | 1 | 3 | 0 | 1 | 0 | 0 | 0 | 1 | 0 | 0 | 0 |
| 25 | 0 | 1 | 0 | 0 | 0 | 1 | 0 | 0 | 0 | 1 | 0 | 0 |
| 26 |  |  | 1 |  |  |  | 1 |  | 1 | 1 |  |  |
| 27 | 0 | 0 | 0 | 1 | 0 | 4 | 2 | 0 | 0 | 0 | 1 | 0 |
| 28 | 0 | 0 | 0 | 1 | 0 | 0 | 0 | 0 | 0 | 1 | 0 | 1 |
| 29 |  |  |  |  |  |  |  |  |  |  |  |  |
| 30 | 1 | 1 | 0 | 1 | 1 | 1 | 0 | 0 | 2 | 0 | 0 | 0 |
| 31 | 0 | 1 | 1 | 0 | 0 | 0 | 0 | 0 | 0 | 1 | 0 | 0 |
| 32 |  |  |  |  |  |  | 1 |  |  |  | 1 |  |
| 33 | 1 | 0 | 0 | 0 | 1 | 0 | 0 | 0 | 0 | 0 | 0 | 0 |
| 34 |  |  |  |  |  |  |  |  |  |  |  |  |
| 35 | 1 | 0 | 1 | 0 | 0 | 0 | 0 | 0 | 0 | 0 | 0 | 0 |
| 36 |  | 1 |  | 2 |  |  | 1 |  | 1 |  |  | 1 |
| 37 | 0 | 1 | 0 | 0 | 0 | 1 | 1 | 1 | 1 | 0 | 1 | 0 |
| 38 |  |  |  |  |  |  |  |  |  |  |  |  |
| 39 |  |  |  |  |  |  |  |  |  |  |  |  |
| 40 | 0 | 1 | 0 | 3 | 2 | 0 | 0 | 0 | 1 | 0 | 0 | 1 |
| 41 | 2 | 0 | 1 | 1 | 2 | 1 | 0 | 1 | 2 | 1 | 1 | 1 |
| 42 | 0 | 1 | 0 | 1 | 1 | 1 | 1 | 0 | 0 | 0 | 0 | 0 |
| 43 | 0 | 0 | 1 | 0 | 0 | 0 | 0 | 0 | 0 | 0 | 0 | 0 |
| 44 | 0 | 0 | 0 | 1 | 0 | 0 | 0 | 0 | 0 | 0 | 0 | 0 |
| 45 | 2 | 0 | 1 | 0 | 0 | 3 | 0 | 0 | 1 | 1 | 1 | 0 |
| 46 | 0 | 0 | 0 | 0 | 0 | 0 | 0 | 0 | 0 | 0 | 0 | 0 |
| 47 |  |  |  |  |  |  |  |  |  |  |  | 1 |
| 48 | 0 | 0 | 0 | 0 | 1 | 0 | 1 | 0 | 1 | 0 | 4 | 0 |
| 49 | 0 | 0 | 0 | 1 | 1 | 0 | 0 | 0 | 0 | 0 | 0 | 0 |
| 50 | 0 | 1 | 0 | 0 | 0 | 1 | 1 | 0 | 0 | 1 | 0 | 0 |
| 51 | 0 | 0 | 0 | 0 | 0 | 0 | 0 | 0 | 0 | 1 | 0 | 0 |
| 52 | 1 | 1 | 1 | 3 | 0 | 2 | 2 | 0 | 2 | 0 | 0 | 0 |
| 53 | 0 | 0 | 1 | 0 | 0 | 1 | 0 | 1 | 0 | 0 | 0 | 0 |
| 54 | 2 | 2 | 1 | 1 | 2 | 0 | 3 | 2 | 2 | 0 | 0 | 3 |
| 55 | 0 | 0 | 0 | 0 | 0 | 1 | 0 | 0 | 0 | 0 | 0 | 0 |
| 56 | 1 | 1 | 0 | 3 | 1 | 0 | 2 | 1 | 0 | 1 | 0 | 0 |
| 57 | 0 | 1 | 2 | 0 | 0 | 1 | 2 | 2 | 1 | 1 | 1 | 0 |
| 58 | 2 | 0 | 3 | 0 | 1 | 0 | 1 | 0 | 1 | 0 | 0 | 0 |
| 59 | 0 | 1 | 0 | 2 | 0 | 0 | 0 | 2 | 0 | 0 | 0 | 0 |
| 60 | 0 | 0 | 0 | 1 | 0 | 1 | 1 | 0 | 1 | 0 | 0 | 0 |
| 61 | 0 | 0 | 0 | 0 | 0 | 0 | 0 | 0 | 0 | 0 | 0 | 0 |
| 62 |  |  |  |  |  |  |  |  |  |  |  |  |
| 63 |  |  |  | 1 |  |  |  |  |  |  |  |  |
| 64 | 2 | 1 | 1 | 1 |  |  |  |  |  |  |  |  |
| 65 | 0 | 1 | 0 | 1 | 0 | 0 | 0 | 0 | 0 | 0 | 0 | 0 |
| 66 |  |  |  |  |  |  |  |  |  |  |  |  |
| 67 |  |  |  |  |  |  |  |  |  |  |  | 1 |
| 68 | 0 | 0 | 0 | 0 | 0 | 0 | 0 | 0 | 0 | 0 | 0 | 0 |
| 68 | 0 | 0 | 1 | 0 | 0 | 0 | 0 | 0 | 0 | 0 | 0 | 0 |
| 70 |  | 1 |  |  |  |  |  |  |  |  |  |  |
| 71 | 1 | 0 | 1 | 0 | 1 | 0 | 0 | 0 | 0 | 2 | 1 | 1 |
| 72 | 0 | 0 | 0 | 0 | 1 | 1 | 0 | 0 | 1 | 0 | 0 | 1 |
| 73 | 0 | 2 | 0 | 2 | 2 | 0 | 0 | 1 | 0 | 0 | 0 | 1 |
| 74 | 0 | 2 | 1 | 3 | 2 | 2 | 1 | 2 | 2 | 0 | 1 | 0 |
| 75 |  |  |  |  |  |  |  |  |  |  |  |  |
| 76 | 1 | 0 | 0 | 0 | 0 | 3 | 0 | 1 | 0 | 3 | 1 | 1 |
| 77 |  |  |  |  |  |  |  |  |  |  |  |  |
| 78 |  |  |  |  |  |  |  |  |  |  |  |  |
| 79 | 0 | 0 | 1 | 0 | 0 | 1 | 0 | 0 | 0 | 1 | 0 | 0 |
| 80 | 0 | 2 | 0 | 0 | 0 | 0 | 0 | 0 | 0 | 1 | 0 | 0 |
| 81 |  |  |  | 1 |  |  |  | 1 |  | 1 |  |  |
| 82 |  |  |  |  |  |  |  |  |  |  |  |  |
| 83 | 0 | 0 | 0 | 0 | 1 | 0 | 0 | 0 | 0 | 0 | 0 | 0 |
| 84 | 0 | 0 | 0 | 0 | 0 | 1 | 0 | 0 | 0 | 0 | 0 | 2 |
| 85 |  |  |  |  |  |  |  |  |  |  | 1 |  |
| 86 | 0 | 0 | 2 | 0 | 0 | 1 | 0 | 2 | 0 | 4 | 0 | 0 |
| 87 |  |  |  |  |  |  |  | 1 |  |  |  |  |
| 88 | 0 | 0 | 0 | 0 | 0 | 1 | 1 | 0 | 0 | 0 | 1 | 0 |
| 89 | - | 1 | - | - | 2 | 1 | - | - | 1 | - | 1 | - |
| 90 | 0 | 0 | 0 | 0 | 0 | 2 | 1 | 0 | 0 | 0 | 1 | 1 |
| 91 | 0 | 1 | 0 | 0 | 1 | 0 | 0 | 0 | 1 | 2 | 0 | 1 |
| 92 | 0 | 1 | 0 | 1 | 0 | 0 | 0 | 1 | 0 | 0 | 1 | 0 |
| 93 | 0 | 0 | 0 | 0 | 0 | 0 | 0 | 0 | 0 | 0 | 0 | 0 |
| 94 | 1 | 0 | 0 | 0 | 0 | 0 | 0 | 0 | 0 | 0 | 0 | 0 |
| 95 | 0 | 0 | 0 | 0 | 0 | 0 | 0 | 0 | 0 | 0 | 0 | 0 |
| 96 |  |  |  |  |  | 1 |  |  |  |  |  |  |
| 97 |  |  |  |  |  |  |  |  |  |  |  |  |
| 98 | 0 | 0 | 0 | 0 | 1 | 0 | 0 | 1 | 0 | 0 | 2 | 0 |
| 99 | 0 | 0 | 0 | 1 | 0 | 0 | 0 | 1 | 0 | 0 | 0 | 0 |
| 100 | 0 | 1 | 0 | 0 | 1 | 0 | 0 | 1 | 0 | 0 | 0 | 0 |
| 101 |  |  | 1 |  |  |  |  | 1 | 1 | 1 | 1 |  |
| 102 |  |  |  |  |  |  |  |  |  |  |  |  |
| 103 | 1 | 1 | 0 | 0 | 0 | 0 | 0 | 0 | 1 | 1 | 0 | 0 |
| 104 | 0 | 1 | 2 | 0 | 1 | 0 | 0 | 0 | 3 | 3 | 0 | 0 |
| 105 | 2 | 1 | 0 | 0 | 1 | 4 | 0 | 0 | 0 | 1 | 2 | 1 |

Table 47. Number of newly diagnosed stage III cervical cancer during each month in 2019.

|  | Month | | | | | | | | | | | |
| --- | --- | --- | --- | --- | --- | --- | --- | --- | --- | --- | --- | --- |
| Hospital | 1 | 2 | 3 | 4 | 5 | 6 | 7 | 8 | 9 | 10 | 11 | 12 |
| 1 | 0 | 0 | 0 | 0 | 0 | 0 | 0 | 0 | 0 | 0 | 1 | 0 |
| 2 | 8 | 8 | 1 | 7 | 3 | 7 | 3 | 3 | 5 | 10 | 13 | 2 |
| 3 | 0 | 0 | 0 | 0 | 0 | 0 | 0 | 0 | 0 | 0 | 0 | 0 |
| 4 | 2 | 1 | 0 | 2 | 0 | 0 | 2 | 1 | 0 | 1 | 1 | 2 |
| 5 | 0 | 0 | 1 | 0 | 0 | 1 | 0 | 0 | 0 | 0 | 0 | 0 |
| 6 |  | 1 |  | 1 |  |  | 2 |  |  |  |  |  |
| 7 | 0 | 0 | 0 | 0 | 0 | 0 | 0 | 0 | 0 | 0 | 0 | 0 |
| 8 |  |  |  |  |  |  |  |  |  |  |  |  |
| 9 | 0 | 0 | 0 | 0 | 0 | 0 | 0 | 0 | 0 | 0 | 0 | 1 |
| 10 | 0 | 0 | 0 | 0 | 1 | 0 | 0 | 0 | 0 | 0 | 0 | 0 |
| 11 | 3 | 3 | 1 | 1 | 1 | 1 | 2 | 2 | 1 | 0 | 2 | 0 |
| 12 | 0 | 0 | 0 | 0 | 0 | 0 | 0 | 0 | 0 | 0 | 0 | 0 |
| 13 | 0 | 0 | 2 | 2 | 0 | 0 | 1 | 2 | 0 | 1 | 2 | 0 |
| 14 | 0 | 0 | 0 | 0 | 0 | 0 | 1 | 0 | 1 | 1 | 0 | 1 |
| 15 | 0 | 0 | 0 | 1 | 0 | 1 | 0 | 2 | 0 | 1 | 0 | 1 |
| 16 | 1 | 1 | 0 | 3 | 1 | 2 | 2 | 4 | 1 | 2 | 1 | 2 |
| 17 | 0 | 0 | 0 | 0 | 0 | 0 | 0 | 0 | 0 | 0 | 0 | 0 |
| 18 | 0 | 1 | 1 | 0 | 0 | 0 | 0 | 0 | 0 | 0 | 0 | 0 |
| 19 | 0 | 0 | 0 | 0 | 0 | 0 | 0 | 0 | 0 | 0 | 0 | 0 |
| 20 | 5 | 2 | 8 | 2 | 4 | 0 | 2 | 1 | 1 | 1 | 2 | 4 |
| 21 | 0 | 0 | 1 | 0 | 0 | 0 | 0 | 0 | 0 | 1 | 0 | 0 |
| 22 | 1 |  |  | 2 |  | 1 | 1 | 1 | 1 | 2 |  |  |
| 23 |  |  |  |  |  |  |  |  |  |  |  |  |
| 24 | 1 | 1 | 2 | 2 | 2 | 0 | 0 | 0 | 1 | 1 | 1 | 0 |
| 25 | 0 | 0 | 1 | 2 | 0 | 0 | 0 | 1 | 0 | 0 | 1 | 0 |
| 26 | 2 |  |  | 2 |  | 1 | 1 | 2 |  |  |  | 1 |
| 27 | 2 | 0 | 3 | 2 | 2 | 1 | 1 | 3 | 1 | 2 | 1 | 2 |
| 28 | 0 | 0 | 0 | 1 | 0 | 1 | 0 | 1 | 0 | 0 | 1 | 0 |
| 29 |  |  |  |  |  |  |  |  |  |  |  |  |
| 30 | 0 | 0 | 0 | 2 | 0 | 0 | 0 | 0 | 0 | 1 | 0 | 0 |
| 31 | 0 | 1 | 1 | 0 | 0 | 1 | 0 | 0 | 1 | 0 | 0 | 1 |
| 32 |  |  |  |  |  |  |  |  |  |  |  |  |
| 33 | 0 | 0 | 0 | 0 | 4 | 0 | 0 | 0 | 0 | 2 | 0 | 0 |
| 34 |  |  |  |  |  |  |  |  |  |  |  |  |
| 35 | 0 | 0 | 0 | 0 | 2 | 0 | 0 | 0 | 0 | 0 | 0 | 0 |
| 36 |  | 2 | 1 | 2 |  | 2 | 2 | 1 |  | 1 | 1 | 2 |
| 37 | 0 | 1 | 0 | 0 | 0 | 1 | 3 | 2 | 3 | 0 | 0 | 0 |
| 38 |  |  |  |  |  |  |  |  |  |  |  |  |
| 39 |  |  |  |  |  |  |  |  |  |  |  |  |
| 40 | 1 | 0 | 1 | 2 | 2 | 3 | 4 | 0 | 2 | 1 | 1 | 3 |
| 41 | 1 | 2 | 1 | 1 | 2 | 0 | 2 | 1 | 3 | 2 | 2 | 4 |
| 42 | 1 | 0 | 0 | 0 | 1 | 1 | 0 | 1 | 0 | 2 | 0 | 0 |
| 43 | 3 | 0 | 1 | 0 | 1 | 1 | 0 | 0 | 0 | 1 | 1 | 1 |
| 44 | 0 | 0 | 0 | 0 | 0 | 0 | 1 | 0 | 0 | 0 | 0 | 0 |
| 45 | 0 | 1 | 2 | 0 | 3 | 2 | 1 | 1 | 1 | 0 | 2 | 0 |
| 46 | 0 | 0 | 1 | 0 | 0 | 0 | 0 | 1 | 0 | 1 | 0 | 0 |
| 47 |  |  |  |  |  |  |  |  |  |  |  |  |
| 48 | 2 | 3 | 2 | 1 | 1 | 0 | 4 | 0 | 2 | 1 | 0 | 2 |
| 49 | 0 | 1 | 1 | 0 | 0 | 1 | 0 | 1 | 0 | 0 | 0 | 0 |
| 50 | 0 | 0 | 0 | 0 | 1 | 0 | 0 | 0 | 0 | 0 | 0 | 0 |
| 51 | 0 | 0 | 0 | 0 | 0 | 0 | 0 | 1 | 0 | 0 | 0 | 2 |
| 52 | 2 | 1 | 1 | 3 | 5 | 0 | 0 | 1 | 3 | 1 | 0 | 2 |
| 53 | 0 | 0 | 0 | 1 | 0 | 0 | 0 | 1 | 1 | 0 | 0 | 1 |
| 54 | 3 | 3 | 6 | 2 | 4 | 2 | 2 | 5 | 2 | 4 | 2 | 0 |
| 55 | 2 | 0 | 0 | 0 | 0 | 0 | 0 | 0 | 0 | 0 | 0 | 0 |
| 56 | 2 | 1 | 0 | 1 | 0 | 0 | 1 | 2 | 1 | 0 | 1 | 2 |
| 57 | 3 | 4 | 1 | 2 | 3 | 0 | 6 | 1 | 2 | 2 | 4 | 3 |
| 58 | 0 | 0 | 0 | 2 | 1 | 1 | 1 | 0 | 0 | 0 | 1 | 1 |
| 59 | 1 | 0 | 2 | 3 | 1 | 1 | 1 | 2 | 1 | 3 | 0 | 3 |
| 60 | 1 | 0 | 0 | 0 | 0 | 0 | 0 | 0 | 1 | 1 | 0 | 1 |
| 61 | 0 | 0 | 0 | 0 | 0 | 0 | 0 | 1 | 0 | 0 | 0 | 0 |
| 62 |  |  |  |  |  |  |  |  |  |  |  |  |
| 63 |  |  |  | 1 | 1 |  | 4 | 2 |  |  |  |  |
| 64 |  | 2 | 2 | 1 | 1 | 1 | 1 |  | 1 |  |  | 1 |
| 65 | 0 | 2 | 2 | 0 | 0 | 0 | 0 | 0 | 3 | 1 | 1 | 0 |
| 66 |  |  |  |  |  |  |  |  |  |  |  |  |
| 67 |  | 1 |  | 1 |  | 1 |  |  |  |  |  |  |
| 68 | 0 | 0 | 0 | 0 | 0 | 1 | 0 | 2 | 0 | 0 | 0 | 0 |
| 68 | 0 | 0 | 0 | 0 | 0 | 0 | 0 | 0 | 0 | 0 | 0 | 0 |
| 70 |  |  |  |  |  |  |  |  |  |  |  |  |
| 71 | 0 | 0 | 0 | 1 | 0 | 0 | 0 | 1 | 1 | 0 | 1 | 0 |
| 72 | 1 | 0 | 0 | 0 | 1 | 2 | 1 | 0 | 0 | 1 | 0 | 2 |
| 73 | 0 | 0 | 0 | 1 | 0 | 1 | 0 | 0 | 1 | 3 | 0 | 0 |
| 74 | 1 | 2 | 1 | 4 | 2 | 1 | 1 | 1 | 2 | 2 | 5 | 3 |
| 75 |  |  |  |  |  |  |  |  |  |  |  |  |
| 76 | 1 | 1 | 0 | 2 | 1 | 0 | 0 | 2 | 2 | 0 | 2 | 0 |
| 77 |  |  |  |  |  |  |  |  |  |  |  |  |
| 78 |  |  |  |  |  |  |  |  |  |  |  |  |
| 79 | 1 | 0 | 0 | 0 | 1 | 0 | 1 | 1 | 1 | 2 | 1 | 0 |
| 80 | 0 | 0 | 0 | 0 | 0 | 0 | 0 | 0 | 0 | 1 | 0 | 1 |
| 81 | 1 |  |  |  | 2 |  |  | 1 |  | 2 |  |  |
| 82 |  |  |  |  |  |  |  |  |  |  |  |  |
| 83 | 0 | 0 | 0 | 0 | 0 | 0 | 0 | 0 | 0 | 0 | 1 | 0 |
| 84 | 0 | 0 | 0 | 0 | 1 | 0 | 1 | 0 | 0 | 0 | 0 | 1 |
| 85 |  |  |  |  |  |  |  |  | 1 |  | 2 |  |
| 86 | 2 | 0 | 0 | 0 | 2 | 0 | 1 | 3 | 4 | 3 | 1 | 1 |
| 87 |  |  | 1 | 1 |  | 1 |  |  |  | 2 |  |  |
| 88 | 0 | 0 | 0 | 0 | 0 | 0 | 0 | 0 | 0 | 1 | 0 | 0 |
| 89 | - | 2 | 3 | - | - | - | - | - | - | - | - | - |
| 90 | 0 | 0 | 0 | 0 | 0 | 0 | 0 | 0 | 0 | 0 | 0 | 1 |
| 91 | 0 | 0 | 0 | 0 | 0 | 1 | 1 | 1 | 0 | 0 | 0 | 0 |
| 92 | 0 | 0 | 0 | 0 | 0 | 1 | 0 | 0 | 0 | 0 | 1 | 0 |
| 93 | 0 | 0 | 0 | 0 | 0 | 0 | 1 | 0 | 0 | 0 | 0 | 1 |
| 94 | 0 | 0 | 0 | 0 | 0 | 0 | 0 | 0 | 0 | 0 | 0 | 0 |
| 95 | 0 | 0 | 0 | 0 | 0 | 0 | 0 | 0 | 0 | 0 | 0 | 0 |
| 96 |  |  |  |  |  |  |  |  |  |  |  |  |
| 97 |  |  |  |  |  |  |  |  |  |  |  |  |
| 98 | 0 | 0 | 0 | 0 | 1 | 0 | 0 | 1 | 0 | 0 | 0 | 0 |
| 99 | 1 | 0 | 0 | 1 | 0 | 0 | 0 | 0 | 0 | 1 | 0 | 0 |
| 100 | 0 | 0 | 0 | 4 | 1 | 0 | 1 | 1 | 1 | 1 | 1 | 0 |
| 101 |  |  |  | 1 |  |  | 1 | 1 | 2 |  | 2 |  |
| 102 |  |  |  |  |  |  |  |  |  |  |  |  |
| 103 | 1 | 1 | 1 | 0 | 1 | 1 | 0 | 0 | 1 | 0 | 1 | 2 |
| 104 | 3 | 0 | 1 | 1 | 0 | 1 | 2 | 0 | 1 | 1 | 2 | 0 |
| 105 | 2 | 1 | 2 | 1 | 0 | 1 | 1 | 1 | 1 | 3 | 3 | 0 |

Table 48. Number of newly diagnosed stage III cervical cancer during each month in 2020.

|  | Month | | | | | | | | | | | |
| --- | --- | --- | --- | --- | --- | --- | --- | --- | --- | --- | --- | --- |
| Hospital | 1 | 2 | 3 | 4 | 5 | 6 | 7 | 8 | 9 | 10 | 11 | 12 |
| 1 | 0 | 0 | 0 | 0 | 0 | 0 | 0 | 0 | 0 | 0 | 0 | 0 |
| 2 | 4 | 2 | 4 | 6 | 2 | 3 | 9 | 3 | 6 | 7 | 6 | 3 |
| 3 | 0 | 0 | 1 | 0 | 0 | 0 | 0 | 0 | 0 | 0 | 0 | 0 |
| 4 | 1 | 1 | 0 | 0 | 0 | 1 | 1 | 0 | 2 | 3 | 2 | 2 |
| 5 | 0 | 0 | 0 | 0 | 0 | 0 | 0 | 0 | 0 | 0 | 1 | 1 |
| 6 | 1 |  |  |  |  | 1 |  |  | 1 |  |  |  |
| 7 | 0 | 0 | 0 | 0 | 0 | 1 | 0 | 0 | 0 | 0 | 0 | 0 |
| 8 |  |  |  |  |  |  |  |  |  |  |  |  |
| 9 | 0 | 0 | 0 | 0 | 0 | 1 | 0 | 0 | 1 | 0 | 0 | 0 |
| 10 | 0 | 0 | 0 | 0 | 1 | 0 | 0 | 0 | 0 | 1 | 0 | 0 |
| 11 | 1 | 0 | 1 | 0 | 2 | 3 | 0 | 3 | 0 | 0 | 0 | 3 |
| 12 | 0 | 0 | 0 | 0 | 0 | 0 | 0 | 0 | 0 | 0 | 0 | 0 |
| 13 | 0 | 0 | 1 | 0 | 2 | 0 | 0 | 1 | 2 | 0 | 2 | 1 |
| 14 | 0 | 0 | 1 | 0 | 0 | 0 | 0 | 0 | 0 | 0 | 0 | 0 |
| 15 | 3 | 3 | 1 | 2 | 1 | 1 | 1 | 1 | 1 | 2 | 4 | 3 |
| 16 | 0 | 2 | 1 | 0 | 0 | 0 | 4 | 3 | 0 | 0 | 4 | 0 |
| 17 | 0 | 0 | 0 | 0 | 0 | 0 | 0 | 0 | 0 | 0 | 0 | 0 |
| 18 | 0 | 0 | 0 | 0 | 0 | 0 | 1 | 0 | 1 | 0 | 0 | 0 |
| 19 | 0 | 0 | 0 | 1 | 0 | 0 | 0 | 0 | 0 | 0 | 0 | 0 |
| 20 | 4 | 1 | 7 | 1 | 1 | 4 | 1 | 1 | 1 | 1 | 0 | 3 |
| 21 | 0 | 1 | 0 | 0 | 1 | 1 | 0 | 0 | 1 | 1 | 1 | 1 |
| 22 | 1 |  |  | 1 | 2 |  | 1 | 1 |  | 2 | 1 |  |
| 23 |  |  |  |  |  |  |  |  |  |  |  |  |
| 24 | 1 | 0 | 3 | 0 | 1 | 0 | 0 | 0 | 1 | 2 | 2 | 0 |
| 25 | 1 | 0 | 1 | 2 | - | 0 | 0 | 0 | 1 | 0 | 0 | 1 |
| 26 |  | 1 |  | 1 | 1 |  | 1 |  |  | 2 | 1 | 1 |
| 27 | 1 | 0 | 1 | 2 | 3 | 2 | 0 | 3 | 3 | 1 | 0 | 2 |
| 28 | 1 | 0 | 0 | 0 | 2 | 1 | 1 | 1 | 1 | 0 | 1 | 3 |
| 29 |  |  |  |  |  |  |  |  |  |  |  |  |
| 30 | 1 | 0 | 0 | 0 | 1 | 1 | 1 | 1 | 0 | 1 | 2 | 1 |
| 31 | 0 | 0 | 0 | 1 | 0 | 0 | 0 | 1 | 0 | 0 | 0 | 0 |
| 32 |  |  |  |  |  | 1 | 1 |  |  |  |  | 1 |
| 33 | 0 | 0 | 0 | 0 | 0 | 0 | 0 | 0 | 0 | 0 | 0 | 1 |
| 34 |  |  |  |  |  |  |  |  |  |  |  |  |
| 35 | 0 | 2 | 0 | 1 | 1 | 0 | 0 | 0 | 0 | 0 | 0 | 0 |
| 36 | 4 |  | 2 |  | 1 | 1 |  |  |  |  | 1 |  |
| 37 | 0 | 0 | 1 | 0 | 1 | 0 | 3 | 0 | 0 | 3 | 1 | 2 |
| 38 |  |  |  |  |  |  |  |  |  |  |  |  |
| 39 |  |  |  |  |  |  |  |  |  |  |  |  |
| 40 | 1 | 2 | 0 | 5 | 2 | 2 | 6 | 0 | 2 | 4 | 0 | 2 |
| 41 | 2 | 3 | 0 | 1 | 2 | 1 | 1 | 1 | 1 | 1 | 1 | 2 |
| 42 | 0 | 1 | 0 | 1 | 0 | 0 | 0 | 0 | 0 | 0 | 0 | 0 |
| 43 | 2 | 0 | 0 | 0 | 1 | 1 | 0 | 1 | 1 | 0 | 0 | 0 |
| 44 | 0 | 0 | 0 | 0 | 0 | 1 | 1 | 1 | 0 | 0 | 0 | 0 |
| 45 | 1 | 1 | 3 | 1 | 0 | 0 | 1 | 1 | 1 | 1 | 0 | 0 |
| 46 | 0 | 0 | 0 | 0 | 0 | 0 | 0 | 1 | 0 | 1 | 0 | 0 |
| 47 |  |  |  |  |  |  |  |  |  | 1 |  |  |
| 48 | 0 | 1 | 0 | 1 | 1 | 1 | 2 | 1 | 4 | 0 | 0 | 0 |
| 49 | 0 | 1 | 0 | 0 | 0 | 0 | 0 | 0 | 0 | 0 | 0 | 0 |
| 50 | 0 | 0 | 0 | 0 | 0 | 0 | 0 | 0 | 0 | 1 | 1 | 0 |
| 51 | 0 | 0 | 0 | 0 | 0 | 0 | 0 | 0 | 0 | 0 | 0 | 0 |
| 52 | 4 | 1 | 2 | 3 | 0 | 0 | 1 | 3 | 1 | 2 | 2 | 1 |
| 53 | 0 | 0 | 1 | 1 | 1 | 0 | 0 | 2 | 0 | 0 | 1 | 1 |
| 54 | 4 | 3 | 1 | 3 | 2 | 1 | 0 | 4 | 3 | 2 | 3 | 0 |
| 55 | 0 | 0 | 0 | 0 | 0 | 0 | 1 | 0 | 0 | 0 | 0 | 0 |
| 56 | 2 | 1 | 0 | 1 | 1 | 0 | 2 | 2 | 2 | 1 | 1 | 0 |
| 57 | 4 | 1 | 2 | 1 | 2 | 3 | 2 | 1 | 2 | 2 | 3 | 1 |
| 58 | 1 | 0 | 1 | 1 | 1 | 0 | 0 | 1 | 2 | 1 | 0 | 0 |
| 59 | 4 | 2 | 0 | 5 | 0 | 1 | 3 | 1 | 1 | 3 | 0 | 0 |
| 60 | 1 | 1 | 0 | 0 | 0 | 0 | 0 | 0 | 0 | 0 | 0 | 0 |
| 61 | 0 | 0 | 0 | 0 | 0 | 0 | 0 | 0 | 0 | 0 | 0 | 0 |
| 62 |  |  |  |  |  |  |  |  |  |  |  |  |
| 63 |  |  |  | 1 |  |  | 1 |  |  |  |  |  |
| 64 | 1 | 3 |  | 1 | 1 |  |  |  | 2 | 2 | 1 | 3 |
| 65 | 0 | 0 | 0 | 0 | 0 | 2 | 0 | 0 | 0 | 0 | 0 | 1 |
| 66 |  |  |  |  |  |  |  |  |  |  |  |  |
| 67 |  |  |  |  |  |  | 1 |  |  |  |  |  |
| 68 | 0 | 0 | 0 | 0 | 0 | 2 | 0 | 0 | 0 | 0 | 0 | 0 |
| 68 | 0 | 0 | 0 | 1 | 0 | 0 | 0 | 0 | 0 | 0 | 0 | 0 |
| 70 |  |  |  |  |  |  |  |  |  |  |  |  |
| 71 | 2 | 0 | 0 | 0 | 0 | 0 | 0 | 0 | 0 | 0 | 0 | 0 |
| 72 | 0 | 1 | 2 | 1 | 0 | 0 | 0 | 0 | 1 | 2 | 1 | 0 |
| 73 | 0 | 0 | 0 | 0 | 1 | 1 | 2 | 0 | 1 | 0 | 0 | 0 |
| 74 | 1 | 2 | 2 | 2 | 2 | 1 | 4 | 1 | 0 | 0 | 1 | 1 |
| 75 |  |  |  |  |  |  |  |  |  |  |  |  |
| 76 | 1 | 1 | 4 | 1 | 1 | 2 | 1 | 1 | 1 | 5 | 0 | 0 |
| 77 |  |  |  |  |  |  |  | 1 |  |  |  |  |
| 78 |  |  |  |  |  |  |  |  |  |  |  |  |
| 79 | 0 | 0 | 1 | 0 | 0 | 0 | 0 | 0 | 0 | 0 | 3 | 0 |
| 80 | 0 | 1 | 0 | 0 | 0 | 0 | 1 | 0 | 0 | 1 | 0 | 1 |
| 81 |  |  | 2 |  | 2 | 1 |  |  |  |  |  |  |
| 82 |  |  |  |  |  |  |  |  |  |  |  |  |
| 83 | 0 | 0 | 0 | 0 | 0 | 0 | 0 | 0 | 1 | 0 | 0 | 1 |
| 84 | 0 | 1 | 0 | 2 | 0 | 1 | 1 | 0 | 0 | 1 | 0 | 1 |
| 85 | 2 |  |  |  |  |  | 1 |  |  |  | 1 |  |
| 86 | 2 | 0 | 1 | 1 | 0 | 3 | 3 | 2 | 4 | 5 | 3 | 1 |
| 87 |  | 2 |  |  |  |  |  |  |  |  |  |  |
| 88 | 0 | 0 | 0 | 0 | 0 | 0 | 0 | 0 | 0 | 0 | 1 | 0 |
| 89 | 3 | - | 1 | 1 | 2 | 2 | 1 | - | 1 | 1 | - | 3 |
| 90 | 0 | 0 | 1 | 0 | 1 | 1 | 1 | 2 | 1 | 0 | 0 | 1 |
| 91 | 1 | 1 | 1 | 1 | 1 | 0 | 1 | 0 | 1 | 0 | 0 | 1 |
| 92 | 0 | 0 | 1 | 0 | 1 | 0 | 0 | 0 | 0 | 1 | 0 | 1 |
| 93 | 0 | 0 | 0 | 0 | 0 | 0 | 0 | 0 | 0 | 0 | 0 | 0 |
| 94 | 0 | 0 | 0 | 0 | 0 | 0 | 0 | 1 | 0 | 0 | 0 | 0 |
| 95 | 0 | 0 | 0 | 0 | 1 | 0 | 0 | 0 | 0 | 0 | 0 | 0 |
| 96 |  |  |  |  |  |  |  | 1 |  |  |  |  |
| 97 |  |  |  |  |  |  |  |  |  |  |  |  |
| 98 | 0 | 0 | 1 | 0 | 1 | 0 | 1 | 0 | 1 | 1 | 1 | 1 |
| 99 | 1 | 2 | 0 | 0 | 0 | 0 | 1 | 0 | 1 | 0 | 1 | 1 |
| 100 | 0 | 2 | 1 | 0 | 0 | 0 | 4 | 1 | 3 | 1 | 0 | 0 |
| 101 | 1 |  |  | 1 | 1 | 1 |  |  |  |  |  |  |
| 102 |  |  |  |  |  |  |  |  |  |  |  |  |
| 103 | 0 | 1 | 0 | 2 | 0 | 0 | 1 | 1 | 0 | 0 | 1 | 0 |
| 104 | 1 | 1 | 0 | 0 | 1 | 1 | 0 | 0 | 1 | 2 | 0 | 1 |
| 105 | 0 | 1 | 0 | 1 | 0 | 0 | 4 | 0 | 3 | 2 | 0 | 0 |

Table 49. Number of newly diagnosed stage IV cervical cancer during each month in 2019.

|  | Month | | | | | | | | | | | |
| --- | --- | --- | --- | --- | --- | --- | --- | --- | --- | --- | --- | --- |
| Hospital | 1 | 2 | 3 | 4 | 5 | 6 | 7 | 8 | 9 | 10 | 11 | 12 |
| 1 | 0 | 0 | 0 | 0 | 1 | 0 | 0 | 0 | 0 | 0 | 0 | 0 |
| 2 | 3 | 1 | 2 | 3 | 1 | 0 | 1 | 1 | 2 | 0 | 1 | 0 |
| 3 | 0 | 0 | 0 | 0 | 0 | 0 | 0 | 0 | 0 | 0 | 0 | 0 |
| 4 | 0 | 0 | 0 | 0 | 0 | 0 | 1 | 1 | 4 | 0 | 2 | 0 |
| 5 | 0 | 0 | 0 | 0 | 0 | 0 | 0 | 0 | 0 | 0 | 0 | 0 |
| 6 |  |  |  |  |  |  |  |  |  |  |  | 1 |
| 7 | 0 | 0 | 0 | 0 | 0 | 1 | 0 | 0 | 0 | 0 | 0 | 0 |
| 8 |  |  |  |  |  |  |  |  |  |  |  |  |
| 9 | 0 | 0 | 0 | 0 | 0 | 0 | 0 | 0 | 0 | 0 | 0 | 0 |
| 10 | 0 | 0 | 0 | 0 | 0 | 0 | 0 | 0 | 0 | 1 | 0 | 0 |
| 11 | 1 | 0 | 2 | 0 | 1 | 0 | 1 | 0 | 0 | 0 | 0 | 1 |
| 12 | 0 | 0 | 0 | 0 | 0 | 0 | 0 | 0 | 0 | 0 | 0 | 0 |
| 13 | 0 | 0 | 0 | 1 | 0 | 0 | 0 | 0 | 0 | 1 | 0 | 1 |
| 14 | 2 |  | 1 | 0 | 0 | 0 | 0 | 0 | 0 | 0 | 0 | 0 |
| 15 | 0 | 0 | 1 | 0 | 0 | 0 | 0 | 0 | 0 | 0 | 0 | 0 |
| 16 | 1 | 0 | 0 | 0 | 0 | 0 | 0 | 1 | 0 | 1 | 0 | 0 |
| 17 | 0 | 0 | 0 | 0 | 0 | 0 | 0 | 0 | 0 | 0 | 0 | 0 |
| 18 | 0 | 0 | 0 | 0 | 0 | 0 | 0 | 0 | 0 | 0 | 0 | 0 |
| 19 | 0 | 1 | 0 | 0 | 0 | 0 | 0 | 0 | 0 | 0 | 0 | 1 |
| 20 | 0 | 2 | 1 | 2 | 0 | 2 | 3 | 1 | 0 | 0 | 1 | 4 |
| 21 | 0 | 0 | 0 | 0 | 1 | 1 | 0 | 1 | 0 | 1 | 0 | 0 |
| 22 |  | 1 | 1 |  |  |  |  |  |  |  |  |  |
| 23 |  |  |  |  |  |  |  |  |  |  |  |  |
| 24 | 0 | 1 | 1 | 4 | 1 | 0 | 1 | 1 | 1 | 1 | 1 | 1 |
| 25 | 0 | 0 | 0 | 1 | 0 | 0 | 0 | 0 | 0 | 2 | 0 | 0 |
| 26 | 1 |  |  |  |  | 1 | 2 |  |  |  |  |  |
| 27 | 0 | 0 | 2 | 0 | 1 | 4 | 0 | 0 | 1 | 1 | 2 | 1 |
| 28 | 0 | 0 | 0 | 1 | 0 | 0 | 1 | 0 | 1 | 2 | 0 | 0 |
| 29 |  |  |  |  |  |  |  |  |  |  |  |  |
| 30 | 0 | 0 | 0 | 0 | 1 | 0 | 1 | 1 | 0 | 0 | 0 | 0 |
| 31 | 1 | 0 | 0 | 0 | 0 | 1 | 0 | 1 | 0 | 0 | 1 | 0 |
| 32 |  |  |  | 1 |  | 1 |  |  |  |  |  |  |
| 33 | 0 | 0 | 0 | 0 | 0 | 0 | 0 | 0 | 0 | 0 | 0 | 1 |
| 34 |  |  |  |  |  |  |  |  |  |  |  |  |
| 35 | 1 | 0 | 0 | 1 | 0 | 0 | 0 | 0 | 0 | 0 | 0 | 0 |
| 36 | 1 |  |  |  | 1 | 2 |  |  |  | 1 | 1 |  |
| 37 | 1 | 1 | 0 | 1 | 0 | 0 | 0 | 3 | 1 | 1 | 0 | 1 |
| 38 |  |  |  |  |  |  |  |  |  |  |  |  |
| 39 |  |  |  |  |  |  |  |  |  |  |  |  |
| 40 | 0 | 2 | 2 | 1 | 1 | 1 | 5 | 0 | 0 | 0 | 0 | 0 |
| 41 | 1 | 2 | 0 | 0 | 0 | 1 | 1 | 0 | 1 | 1 | 0 | 0 |
| 42 | 0 | 1 | 0 | 1 | 0 | 1 | 2 | 0 | 0 | 0 | 0 | 2 |
| 43 | 0 | 1 | 2 | 0 | 1 | 2 | 0 | 0 | 0 | 2 | 1 |  |
| 44 | 0 | 0 | 1 | 0 | 0 | 0 | 0 | 0 | 0 | 0 | 0 | 0 |
| 45 | 0 | 0 | 1 | 0 | 1 | 0 | 2 | 0 | 2 | 2 | 1 | 1 |
| 46 | 0 | 0 | 0 | 0 | 0 | 0 | 0 | 0 | 0 | 1 | 0 | 0 |
| 47 |  |  |  |  |  |  |  |  |  |  |  |  |
| 48 | 1 | 1 | 0 | 0 | 0 | 1 | 0 | 0 | 0 | 1 | 1 | 0 |
| 49 | 0 | 0 | 0 | 0 | 0 | 0 | 0 | 0 | 0 | 1 | 0 | 0 |
| 50 | 2 | 0 | 0 | 0 | 0 | 0 | 0 | 0 | 0 | 0 | 0 | 0 |
| 51 | 0 | 0 | 0 | 0 | 0 | 1 | 0 | 0 | 1 | 0 | 0 | 0 |
| 52 | 1 | 0 | 0 | 0 | 1 | 0 | 1 | 0 | 0 | 1 | 4 | 0 |
| 53 | 0 | 1 | 0 | 0 | 0 | 0 | 1 | 1 | 0 | 1 | 0 | 0 |
| 54 | 0 | 0 | 1 | 2 | 1 | 2 | 2 | 1 | 0 | 1 | 2 | 4 |
| 55 | 0 | 0 | 0 | 0 | 0 | 0 | 0 | 0 | 0 | 0 | 0 | 0 |
| 56 | 0 | 1 | 0 | 0 | 0 | 1 | 0 | 1 | 0 | 1 | 0 | 0 |
| 57 | 1 | 1 | 0 | 0 | 0 | 1 | 0 | 0 | 2 | 3 | 0 | 1 |
| 58 | 1 | 1 | 2 | 0 | 1 | 0 | 0 | 1 | 1 | 1 | 0 | 0 |
| 59 | 0 | 2 | 0 | 0 | 0 | 1 | 1 | 0 | 0 | 0 | 0 | 1 |
| 60 | 0 | 0 | 0 | 1 | 0 | 0 | 0 | 0 | 1 | 1 | 0 | 0 |
| 61 | 0 | 0 | 0 | 0 | 0 | 0 | 0 | 0 | 0 | 0 | 1 | 0 |
| 62 |  |  |  |  |  |  |  |  |  |  |  |  |
| 63 |  |  |  |  |  |  |  |  |  |  |  | 1 |
| 64 |  |  | 1 |  |  |  |  | 1 |  |  | 1 |  |
| 65 | 0 | 0 | 0 | 0 | 1 | 0 | 0 | 0 | 1 | 0 | 0 | 1 |
| 66 |  |  |  |  |  |  |  |  |  |  |  |  |
| 67 |  |  |  |  |  |  |  | 1 | 1 |  |  |  |
| 68 | 0 | 0 | 0 | 0 | 0 | 0 | 0 | 0 | 0 | 1 | 0 | 0 |
| 68 | 0 | 0 | 0 | 0 | 0 | 0 | 0 | 0 | 0 | 0 | 0 | 0 |
| 70 |  |  |  |  |  |  |  |  |  |  |  |  |
| 71 | 1 | 0 | 0 | 0 | 0 | 0 | 0 | 0 | 1 | 0 | 0 | 1 |
| 72 | 0 | 0 | 0 | 0 | 0 | 0 | 0 | 0 | 0 | 0 | 0 | 1 |
| 73 | 1 | 1 | 0 | 0 | 0 | 0 | 0 | 0 | 0 | 1 | 0 | 0 |
| 74 | 2 | 0 | 2 | 0 | 2 | 1 | 2 | 1 | 0 | 0 | 1 | 1 |
| 75 |  |  |  |  |  |  |  |  |  |  |  |  |
| 76 | 2 | 0 | 0 | 1 | 0 | 0 | 0 | 0 | 1 | 0 | 1 | 0 |
| 77 |  |  |  |  |  |  |  |  |  |  |  |  |
| 78 |  |  |  |  |  |  |  |  |  |  |  |  |
| 79 | 0 | 0 | 1 | 0 | 0 | 0 | 0 | 0 | 0 | 0 | 3 | 0 |
| 80 | 0 | 1 | 0 | 0 | 0 | 1 | 0 | 0 | 1 | 1 | 1 | 0 |
| 81 |  |  |  |  |  | 2 |  | 1 | 1 | 1 |  |  |
| 82 |  |  |  |  |  |  |  |  |  |  |  |  |
| 83 | 0 | 0 | 0 | 0 | 0 | 0 | 0 | 0 | 0 | 0 | 2 | 0 |
| 84 | 0 | 1 | 1 | 1 | 0 | 0 | 1 | 0 | 0 | 0 | 0 | 1 |
| 85 | 1 |  | 1 | 1 | 1 | 1 |  | 2 |  | 1 |  |  |
| 86 | 1 | 0 | 0 | 0 | 1 | 0 | 1 | 1 | 0 | 2 | 0 | 0 |
| 87 |  |  |  |  | 1 |  |  |  |  | 1 | 1 |  |
| 88 | 0 | 2 | 0 | 0 | 0 | 0 | 0 | 0 | 1 | 0 | 0 | 0 |
| 89 | 3 | - | 1 | - | - | - | - | - | 1 | 1 | - | - |
| 90 | 2 | 0 | 0 | 0 | 1 | 0 | 1 | 1 | 1 | 0 | 2 | 1 |
| 91 | 0 | 0 | 1 | 0 | 0 | 0 | 0 | 1 | 3 | 2 | 0 | 0 |
| 92 | 1 | 0 | 0 | 0 | 0 | 0 | 1 | 0 | 0 | 0 | 0 | 1 |
| 93 | 0 | 0 | 0 | 1 | 0 | 2 | 0 | 0 | 1 | 0 | 0 | 0 |
| 94 | 1 | 0 | 1 | 0 | 0 | 0 | 0 | 0 | 0 | 0 | 1 | 0 |
| 95 | 0 | 0 | 0 | 0 | 0 | 0 | 0 | 0 | 0 | 0 | 0 | 0 |
| 96 |  |  | 1 |  | 1 |  |  |  |  |  |  |  |
| 97 |  |  |  |  |  |  |  |  |  |  |  |  |
| 98 | 0 | 0 | 0 | 1 | 0 | 0 | 0 | 2 | 0 | 0 | 0 | 0 |
| 99 | 0 | 0 | 1 | 1 | 1 | 0 | 0 | 0 | 0 | 1 | 0 | 0 |
| 100 | 1 | 1 | 1 | 2 | 1 | 1 | 2 | 1 | 0 | 1 | 0 | 2 |
| 101 |  |  |  |  |  |  | 1 | 1 | 1 |  | 1 |  |
| 102 |  |  |  |  |  |  |  |  |  |  |  |  |
| 103 | 1 | 0 | 0 | 1 | 0 | 0 | 1 | 0 | 1 | 0 | 0 | 1 |
| 104 | 0 | 0 | 2 | 0 | 1 | 1 | 0 | 0 | 0 | 0 | 1 | 0 |
| 105 | 0 | 0 | 0 | 0 | 1 | 2 | 1 | 1 | 0 | 1 | 3 | 0 |

Table 50. Number of newly diagnosed stage IV cervical cancer during each month in 2020.

|  | Month | | | | | | | | | | | |
| --- | --- | --- | --- | --- | --- | --- | --- | --- | --- | --- | --- | --- |
| Hospital | 1 | 2 | 3 | 4 | 5 | 6 | 7 | 8 | 9 | 10 | 11 | 12 |
| 1 | 0 | 0 | 0 | 1 | 0 | 0 | 0 | 0 | 0 | 0 | 0 | 0 |
| 2 | 1 | 1 | 0 | 2 | 1 | 3 | 1 | 2 | 2 | 2 | 2 | 1 |
| 3 | 0 | 0 | 0 | 1 | 0 | 2 | 0 | 0 | 0 | 0 | 0 | 1 |
| 4 | 0 | 0 | 0 | 0 | 2 | 0 | 1 | 0 | 0 | 2 | 1 | 1 |
| 5 | 0 | 0 | 0 | 0 | 1 | 1 | 0 | 0 | 0 | 0 | 0 | 0 |
| 6 | 2 | 1 |  |  |  |  |  |  |  |  | 1 | 1 |
| 7 | 0 | 0 | 0 | 0 | 0 | 0 | 1 | 0 | 0 | 0 | 0 | 0 |
| 8 |  |  |  |  |  |  |  |  |  |  |  |  |
| 9 | 0 | 0 | 0 | 0 | 0 | 0 | 0 | 0 | 0 | 0 | 2 | 0 |
| 10 | 0 | 1 | 0 | 0 | 1 | 0 | 0 | 0 | 0 | 0 | 0 | 0 |
| 11 | 0 | 0 | 0 | 0 | 0 | 1 | 0 | 0 | 0 | 1 | 1 | 0 |
| 12 | 0 | 0 | 0 | 0 | 0 | 0 | 0 | 0 | 0 | 0 | 0 | 0 |
| 13 | 0 | 0 | 0 | 1 | 1 | 0 | 0 | 1 | 0 | 0 | 1 | 0 |
| 14 | 0 | 0 | 0 | 0 | 0 | 0 | 1 | 0 | 1 | 0 | 0 | 0 |
| 15 | 0 | 0 | 0 | 0 | 1 | 0 | 0 | 0 | 0 | 1 | 1 | 0 |
| 16 | 0 | 1 | 3 | 1 | 1 | 1 | 1 | 0 | 0 | 3 | 1 | 2 |
| 17 | 0 | 0 | 0 | 0 | 0 | 0 | 0 | 0 | 0 | 0 | 0 | 0 |
| 18 | 0 | 0 | 0 | 0 | 0 | 0 | 0 | 0 | 0 | 0 | 0 | 0 |
| 19 | 0 | 0 | 0 | 0 | 0 | 0 | 0 | 1 | 0 | 0 | 0 | 0 |
| 20 | 1 | 2 | 0 | 0 | 2 | 1 | 2 | 2 | 3 | 1 | 1 | 1 |
| 21 | 0 | 0 | 1 | 0 | 0 | 0 | 0 | 0 | 0 | 1 | 0 | 1 |
| 22 |  |  |  |  |  | 1 |  |  |  | 1 |  |  |
| 23 |  |  |  |  |  |  |  |  |  |  |  |  |
| 24 | 0 | 0 | 0 | 0 | 0 | 0 | 1 | 2 | 0 | 0 | 0 | 0 |
| 25 | 0 | 0 | 1 | 0 | 0 | 1 | 0 | 0 | 0 | 2 | 0 | 0 |
| 26 |  |  |  | 1 |  |  | 1 |  |  |  |  |  |
| 27 | 0 | 0 | 2 | 0 | 1 | 3 | 1 | 1 | 0 | 0 | 1 | 2 |
| 28 | 1 | 1 | 1 | 1 | 0 | 0 | 1 | 0 | 0 | 1 | 0 | 0 |
| 29 |  |  |  |  |  |  |  |  |  |  |  |  |
| 30 | 0 | 0 | 1 | 0 | 0 | 0 | 0 | 0 | 1 | 0 | 0 | 2 |
| 31 | 0 | 0 | 1 | 1 | 0 | 0 | 0 | 0 | 0 | 0 | 0 | 0 |
| 32 |  |  |  | 1 |  | 1 |  | 2 |  |  |  |  |
| 33 | 0 | 0 | 1 | 0 | 0 | 0 | 0 | 0 | 0 | 1 | 1 | 0 |
| 34 |  |  |  |  |  |  |  |  |  |  |  |  |
| 35 | 1 | 0 | 0 | 0 | 2 | 1 | 1 | 0 | 0 | 0 | 0 | 0 |
| 36 |  |  |  |  |  | 2 | 1 |  |  |  | 1 | 1 |
| 37 | 0 | 1 | 1 | 0 | 1 | 0 | 0 | 1 | 1 | 1 | 0 | 0 |
| 38 |  |  |  |  |  |  |  |  |  |  |  |  |
| 39 |  |  |  |  |  |  |  |  |  |  |  |  |
| 40 | 0 | 0 | 0 | 0 | 1 | 1 | 2 | 1 | 1 | 0 | 0 | 0 |
| 41 | 0 | 2 | 1 | 0 | 0 | 2 | 1 | 0 | 1 | 1 | 1 | 0 |
| 42 | 1 | 1 | 0 | 0 | 0 | 0 | 1 | 1 | 0 | 0 | 0 | 0 |
| 43 | 2 | 1 | 0 | 0 | 0 | 0 | 0 | 0 | 0 | 0 | 0 | 1 |
| 44 | 0 | 0 | 0 | 0 | 1 | 0 | 0 | 0 | 0 | 0 | 0 | 0 |
| 45 | 1 | 1 | 1 | 0 | 3 | 0 | 0 | 2 | 0 | 0 | 1 | 1 |
| 46 | 1 | 0 | 0 | 0 | 0 | 0 | 0 | 1 | 0 | 0 | 0 | 0 |
| 47 |  | 1 | 2 |  |  |  |  |  |  |  |  |  |
| 48 | 1 | 0 | 0 | 0 | 0 | 0 | 0 | 0 | 0 | 0 | 0 | 0 |
| 49 | 0 | 0 | 0 | 0 | 0 | 0 | 0 | 0 | 0 | 0 | 0 | 0 |
| 50 | 0 | 0 | 0 | 0 | 0 | 0 | 0 | 0 | 0 | 0 | 0 | 0 |
| 51 | 0 | 1 | 0 | 1 | 0 | 1 | 0 | 0 | 0 | 1 | 0 | 0 |
| 52 | 0 | 0 | 1 | 0 | 0 | 1 | 0 | 1 | 2 | 1 | 2 | 0 |
| 53 | 0 | 0 | 0 | 1 | 1 | 0 | 0 | 0 | 0 | 1 | 0 | 0 |
| 54 | 2 | 3 | 0 | 2 | 0 | 1 | 1 | 1 | 1 | 1 | 2 | 0 |
| 55 | 0 | 0 | 0 | 0 | 2 | 0 | 0 | 0 | 0 | 0 | 1 | 0 |
| 56 | 0 | 0 | 0 | 1 | 0 | 0 | 0 | 0 | 0 | 0 | 0 | 0 |
| 57 | 1 | 1 | 2 | 2 | 0 | 0 | 1 | 0 | 0 | 1 | 2 | 0 |
| 58 | 1 | 0 | 1 | 2 | 1 | 0 | 0 | 0 | 1 | 1 | 0 | 0 |
| 59 | 3 | 1 | 2 | 1 | 0 | 1 | 0 | 1 | 1 | 1 | 0 | 2 |
| 60 | 0 | 0 | 0 | 1 | 0 | 0 | 0 | 0 | 0 | 0 | 0 | 0 |
| 61 | 0 | 0 | 0 | 0 | 0 | 0 | 0 | 0 | 0 | 0 | 0 | 0 |
| 62 |  |  |  |  |  |  |  |  |  |  |  |  |
| 63 | 1 |  |  |  |  |  |  |  |  |  |  |  |
| 64 | 1 |  |  |  |  | 1 |  |  | 1 | 2 |  |  |
| 65 | 0 | 0 | 0 | 0 | 0 | 0 | 0 | 0 | 0 | 0 | 0 | 0 |
| 66 |  |  |  |  |  |  |  |  |  |  |  |  |
| 67 | 1 |  |  |  |  |  |  |  | 1 |  |  | 1 |
| 68 | 0 | 0 | 0 | 0 | 0 | 0 | 1 | 0 | 0 | 0 | 0 | 0 |
| 68 | 0 | 0 | 0 | 0 | 0 | 0 | 0 | 0 | 0 | 0 | 0 | 0 |
| 70 |  |  |  |  |  |  |  |  |  |  |  |  |
| 71 | 0 | 0 | 1 | 0 | 0 | 0 | 0 | 0 | 0 | 0 | 0 | 0 |
| 72 | 0 | 0 | 2 | 1 | 1 | 0 | 1 | 0 | 0 | 0 | 0 | 0 |
| 73 | 0 | 0 | 0 | 0 | 0 | 1 | 0 | 0 | 0 | 0 | 0 | 0 |
| 74 | 1 | 1 | 0 | 1 | 1 | 0 | 2 | 1 | 0 | 2 | 0 | 2 |
| 75 |  |  |  |  |  |  |  |  |  |  |  |  |
| 76 | 1 | 0 | 0 | 0 | 0 | 0 | 1 | 0 | 3 | 1 | 0 | 3 |
| 77 |  |  |  |  |  |  |  |  |  |  |  |  |
| 78 |  |  |  |  |  |  |  |  |  |  |  |  |
| 79 | 0 | 0 | 1 | 1 | 0 | 0 | 0 | 0 | 0 | 1 | 0 | 0 |
| 80 | 0 | 0 | 0 | 0 | 0 | 0 | 0 | 0 | 1 | 1 | 0 | 0 |
| 81 |  |  |  |  | 1 |  |  |  |  |  | 1 |  |
| 82 |  |  |  |  |  |  |  |  |  |  |  |  |
| 83 | 0 | 0 | 0 | 1 | 0 | 2 | 0 | 0 | 0 | 0 | 0 | 0 |
| 84 | 1 | 0 | 0 | 0 | 0 | 0 | 0 | 1 | 2 | 1 | 0 | 0 |
| 85 | 1 |  |  |  |  |  |  | 1 |  | 1 |  |  |
| 86 | 2 | 1 | 0 | 1 | 0 | 1 | 0 | 2 | 0 | 1 | 1 | 1 |
| 87 |  |  |  |  |  |  |  |  |  |  |  |  |
| 88 | 0 | 0 | 0 | 0 | 0 | 0 | 0 | 0 | 0 | 1 | 0 | 1 |
| 89 | - | - | - | 1 | - | - | 1 | - | - | 1 | - |  |
| 90 | 0 | 0 | 0 | 0 | 0 | 0 | 0 | 0 | 1 | 0 | 1 | 0 |
| 91 | 1 | 0 | 0 | 0 | 0 | 1 | 0 | 0 | 1 | 0 | 2 | 1 |
| 92 | 0 | 0 | 0 | 0 | 0 | 0 | 0 | 0 | 1 | 0 | 0 | 0 |
| 93 | 0 | 2 | 0 | 0 | 0 | 0 | 0 | 0 | 0 | 0 | 0 | 0 |
| 94 | 0 | 0 | 0 | 0 | 0 | 0 | 1 | 0 | 0 | 0 | 0 | 0 |
| 95 | 0 | 0 | 0 | 0 | 0 | 0 | 0 | 0 | 0 | 1 | 0 | 0 |
| 96 |  |  |  |  |  |  |  |  |  |  |  | 1 |
| 97 |  |  |  |  |  |  |  |  |  |  |  |  |
| 98 | 0 | 0 | 0 | 1 | 0 | 0 | 0 | 1 | 0 | 0 | 1 | 2 |
| 99 | 1 | 0 | 1 | 2 | 1 | 1 | 0 | 1 | 0 | 0 | 0 | 1 |
| 100 | 1 | 1 | 2 | 0 | 0 | 1 | 1 | 2 | 0 | 0 | 1 | 0 |
| 101 | 1 |  | 1 |  |  | 1 | 1 |  |  |  |  |  |
| 102 |  |  |  |  |  |  |  |  |  |  |  |  |
| 103 | 0 | 0 | 0 | 0 | 0 | 0 | 1 | 2 | 1 | 1 | 0 | 0 |
| 104 | 0 | 0 | 0 | 0 | 0 | 0 | 1 | 0 | 2 | 1 | 0 | 0 |
| 105 | 0 | 0 | 1 | 0 | 1 | 0 | 1 | 0 | 1 | 0 | 0 | 1 |

Table 51. Number of gastric cancer surgery during each month in 2019

|  | Month | | | | | | | | | | | |
| --- | --- | --- | --- | --- | --- | --- | --- | --- | --- | --- | --- | --- |
| Hospital | 1 | 2 | 3 | 4 | 5 | 6 | 7 | 8 | 9 | 10 | 11 | 12 |
| 1 | 0 | 0 | 2 | 0 | 1 | 2 | 1 | 0 | 3 | 1 | 0 | 0 |
| 2 | 46 | 49 | 64 | 62 | 50 | 54 | 51 | 52 | 50 | 56 | 55 | 51 |
| 3 | 0 | 0 | 2 | 2 | 3 | 3 | 5 | 3 | 2 | 2 | 2 | 2 |
| 4 | 0 | 1 | 0 | 2 | 1 | 2 | 2 | 0 | 2 | 2 | 0 | 0 |
| 5 | 5 | 2 | 3 | 1 | 4 | 1 | 2 | 2 | 3 | 7 | 2 | 4 |
| 6 | 3 | 4 | 4 | 2 | 3 | 7 | 3 | 2 | 5 | 4 | 5 | 8 |
| 7 | 8 | 2 | 6 | 5 | 8 | 7 | 8 | 6 | 14 | 9 | 7 | 7 |
| 8 | 1 | 3 | 0 | 3 | 2 | 0 | 3 | 4 | 3 | 3 | 4 | 2 |
| 9 | 4 | 2 | 3 | 4 | 5 | 2 | 4 | 6 | 4 | 5 | 5 | 2 |
| 10 | 7 | 9 | 7 | 4 | 5 | 5 | 6 | 6 | 4 | 5 | 8 | 7 |
| 11 | 8 | 10 | 6 | 11 | 5 | 9 | 7 | 5 | 13 | 8 | 12 | 6 |
| 12 | 2 | 2 | 1 | 2 | 2 | 0 | 1 | 3 | 4 | 4 | 2 | 1 |
| 13 | 8 | 3 | 6 | 6 | 10 | 9 | 12 | 12 | 9 | 12 | 10 | 6 |
| 14 | 3 | 5 | 4 | 6 | 5 | 7 | 5 | 6 | 9 | 6 | 5 | 5 |
| 15 | 7 | 12 | 14 | 13 | 12 | 14 | 9 | 12 | 11 | 9 | 9 | 8 |
| 16 | 10 | 9 | 14 | 10 | 10 | 10 | 14 | 4 | 3 | 9 | 10 | 12 |
| 17 | 0 | 0 | 0 | 0 | 0 | 0 | 0 | 0 | 0 | 0 | 0 | 0 |
| 18 | 3 | 1 | 6 | 4 | 4 | 3 | 5 | 3 | 3 | 6 | 6 | 4 |
| 19 | 5 | 2 | 2 | 3 | 3 | 5 | 1 | 5 | 3 | 2 | 3 | 4 |
| 20 | 28 | 23 | 28 | 25 | 22 | 21 | 23 | 21 | 18 | 25 | 21 | 20 |
| 21 | 5 | 6 | 6 | 7 | 7 | 5 | 9 | 5 | 10 | 9 | 5 | 4 |
| 22 | 2 | 3 | 1 | 2 | 1 | 3 | 4 | 1 | 2 | 3 | 1 | 3 |
| 23 | 29 | 23 | 26 | 26 | 28 | 25 | 32 | 29 | 20 | 30 | 21 | 26 |
| 24 | 7 | 7 | 8 | 5 | 12 | 7 | 10 | 13 | 7 | 7 | 5 | 10 |
| 25 | 6 | 7 | 19 | 11 | 4 | 8 | 12 | 12 | 8 | 10 | 4 | 8 |
| 26 | 1 | 5 | 6 | 5 | 1 | 6 | 9 | 9 | 8 | 5 | 2 | 5 |
| 27 | 0 | 3 | 2 | 2 | 4 | 1 | 2 | 4 | 4 | 3 | 2 | 2 |
| 28 | 4 | 2 | 5 | 4 | 5 | 2 | 3 | 2 | 4 | 5 | 3 | 6 |
| 29 | 4 | 8 | 8 | 8 | 7 | 8 | 9 | 9 | 2 | 6 | 7 | 6 |
| 30 | 5 | 1 | 1 | 6 | 4 | 1 | 2 | 4 | 1 | 5 | 2 | 2 |
| 31 | 2 | 3 | 6 | 5 | 5 | 3 | 4 | 0 | 2 | 3 | 3 | 4 |
| 32 | 1 | 3 | 4 |  | 1 | 2 | 3 | 3 | 4 | 5 | 2 | 2 |
| 33 | 7 | 4 | 4 | 3 | 2 | 3 | 7 | 7 | 2 | 7 | 7 | 1 |
| 34 |  |  |  |  |  |  |  |  |  |  |  |  |
| 35 | 5 | 2 | 7 | 7 | 4 | 2 | 6 | 11 | 8 | 8 | 7 | 8 |
| 36 | 2 | 1 | 3 | 8 | 4 | 3 | 2 | 2 | 3 | 4 | 2 | 3 |
| 37 | 20 | 19 | 16 | 16 | 9 | 11 | 17 | 12 | 12 | 15 | 18 | 11 |
| 38 | 0 | 7 | 9 | 1 | 7 | 6 | 4 | 6 | 3 | 3 | 8 | 6 |
| 39 |  |  |  | 1 |  |  |  | 1 |  | 3 | 1 | 1 |
| 40 | 4 | 5 | 3 | 3 | 1 | 3 | 2 | 4 | 2 | 5 | 3 | 1 |
| 41 | 18 | 15 | 12 | 10 | 12 | 12 | 12 | 12 | 21 | 10 | 20 | 12 |
| 42 | 9 | 5 | 4 | 2 | 5 | 2 | 3 | 4 | 3 | 6 | 4 | 2 |
| 43 | 2 | 3 | 5 | 7 | 5 | 7 | 9 | 2 | 6 | 7 | 6 | 4 |
| 44 | 3 | 3 | 4 | 1 | 1 | 3 | 0 | 0 | 1 | 1 | 0 | 0 |
| 45 | 7 | 7 | 6 | 3 | 3 | 5 | 4 | 2 | 3 | 7 | 2 | 5 |
| 46 | 12 | 9 | 6 | 4 | 5 | 10 | 9 | 8 | 7 | 12 | 12 | 8 |
| 47 | 1 | 2 |  | 3 | 2 | 1 | 2 | 1 | 3 |  |  | 1 |
| 48 | 12 | 13 | 10 | 11 | 5 | 5 | 6 | 6 | 8 | 9 | 5 | 7 |
| 49 | 9 | 11 | 16 | 13 | 7 | 6 | 10 | 12 | 12 | 19 | 9 | 10 |
| 50 | 0 | 0 | 1 | 1 | 1 | 0 | 1 | 1 | 2 | 0 | 2 | 0 |
| 51 | 11 | 8 | 10 | 7 | 3 | 10 | 10 | 11 | 8 | 14 | 17 | 14 |
| 52 | 8 | 5 | 7 | 7 | 8 | 8 | 11 | 11 | 11 | 10 | 7 | 13 |
| 53 | 9 | 5 | 4 | 5 | 5 | 7 | 9 | 6 | 7 | 7 | 5 | 4 |
| 54 | 19 | 26 | 27 | 23 | 11 | 21 | 23 | 22 | 23 | 32 | 27 | 28 |
| 55 |  | 1 | 3 | 3 | 4 | 3 | 3 | 3 | 1 | 1 | 2 | 3 |
| 56 | 0 | 7 | 11 | 10 | 8 | 5 | 7 | 4 | 12 | 10 | 5 | 14 |
| 57 | 9 | 8 | 11 | 14 | 13 | 15 | 14 | 14 | 13 | 13 | 9 | 8 |
| 58 | 5 | 4 | 6 | 7 | 2 | 4 | 4 | 3 | 3 | 8 | 9 | 6 |
| 59 | 1 | 6 | 2 | 2 | 4 | 2 | 6 | 3 | 5 | 3 | 4 | 2 |
| 60 | 1 | 1 | 4 | 1 | 4 | 6 | 7 | 4 | 5 | 7 | 3 | 7 |
| 61 | 10 | 6 | 5 | 3 | 8 | 5 | 6 | 5 | 4 | 7 | 9 | 6 |
| 62 | 5 | 4 | 5 | 5 | 4 | 7 | 11 | 5 | 8 | 10 | 8 | 3 |
| 63 |  | 1 | 2 | 2 | 1 | 2 | 5 | 1 | 4 | 4 | 2 | 3 |
| 64 | 10 | 5 | 7 | 7 | 6 | 13 | 7 | 8 | 9 | 8 | 9 | 9 |
| 65 | 8 | 11 | 8 | 7 | 16 | 13 | 12 | 17 | 5 | 8 | 10 | 8 |
| 66 | 7 | 3 | 2 | 6 | 4 | 3 | 3 | 1 | 1 | 7 | 6 | 5 |
| 67 | 5 | 3 | 4 | 4 | 4 | 3 | 3 | 3 | 3 | 7 | 4 | 4 |
| 68 | 17 | 12 | 11 | 13 | 10 | 7 | 9 | 9 | 9 | 13 | 13 | 8 |
| 68 | 2 | 3 | 1 | 3 | 0 | 1 | 2 | 4 | 0 | 0 | 4 | 3 |
| 70 |  |  |  |  | 4 | 1 | 1 | 2 | 2 | 1 |  | 1 |
| 71 | 5 | 5 | 7 | 4 | 3 | 3 | 4 | 3 | 6 | 3 | 6 | 3 |
| 72 | 12 | 10 | 9 | 9 | 9 | 3 | 7 | 8 | 12 | 11 | 7 | 10 |
| 73 | 2 | 2 | 5 | 4 | 5 | 4 | 4 | 3 | 8 | 2 | 2 | 4 |
| 74 | 10 | 12 | 10 | 9 | 9 | 5 | 11 | 4 | 10 | 8 | 6 | 2 |
| 75 | 1 | 2 | 0 | 0 | 0 | 1 | 1 | 1 | 0 | 1 | 0 | 0 |
| 76 | 7 | 7 | 7 | 4 | 3 | 3 | 7 | 6 | 3 | 7 | 9 | 9 |
| 77 |  |  |  |  |  | 3 | 3 | 1 |  |  | 1 | 3 |
| 78 | 1 |  |  |  |  | 1 |  | 1 |  | 1 |  | 2 |
| 79 | 9 | 5 | 9 | 8 | 3 | 8 | 7 | 6 | 6 | 8 | 10 | 6 |
| 80 | 7 | 7 | 2 | 7 | 9 | 5 | 12 | 11 | 11 | 5 | 4 | 5 |
| 81 | 6 | 4 | 4 | 4 | 3 | 6 | 6 | 5 | 7 | 7 | 4 | 7 |
| 82 |  |  |  |  |  |  |  |  |  |  |  |  |
| 83 | 4 | 6 | 7 | 1 | 6 | 8 | 6 | 11 | 8 | 9 | 7 | 10 |
| 84 |  |  |  |  |  |  |  |  |  |  |  |  |
| 85 | 2 | 5 | 7 | 7 | 7 | 3 | 5 | 5 | 11 | 4 | 4 | 3 |
| 86 | 11 | 6 | 9 | 6 | 6 | 5 | 8 | 8 | 11 | 14 | 11 | 6 |
| 87 | 3 | 3 | 1 | 2 | 3 | 5 | 3 | 2 | 5 | 6 | 4 | 2 |
| 88 | 1 | 6 | 4 | 4 | 5 | 6 | 5 | 6 | 2 | 7 | 6 | 6 |
| 89 | 20 | 20 | 24 | 22 | 21 | 16 | 28 | 20 | 16 | 24 | 22 | 20 |
| 90 | 6 | 7 | 6 | 8 | 5 | 2 | 8 | 6 | 7 | 6 | 7 | 5 |
| 91 | 12 | 10 | 9 | 8 | 9 | 14 | 6 | 14 | 9 | 8 | 4 | 6 |
| 92 |  |  |  |  |  |  |  |  |  |  |  |  |
| 93 | 3 | 2 | 2 | 6 | 8 | 2 | 5 | 4 | 5 | 2 | 2 | 3 |
| 94 | 2 | 2 | 1 | 1 | 3 | 2 | 1 | 1 | 1 | 1 | 2 | 1 |
| 95 | 3 | 2 | 4 | 2 | 1 | 0 | 5 | 1 | 1 | 0 | 1 | 2 |
| 96 | 4 | 4 | 3 | 2 | 2 | 3 | 4 | 1 | 1 | 7 | 3 | 1 |
| 97 | 1 | 3 | 4 | 1 | 1 | 1 | 2 | 2 | 1 | 1 | 0 | 1 |
| 98 | 6 | 2 | 6 | 5 | 4 | 1 | 4 | 4 | 0 | 5 | 4 | 5 |
| 99 | 1 | 0 | 5 | 7 | 3 | 2 | 0 | 2 | 2 | 3 | 0 | 1 |
| 100 | 0 | 0 | 2 | 2 | 6 | 4 | 7 | 6 | 7 | 7 | 4 | 3 |
| 101 | 10 | 5 | 8 | 9 | 7 | 6 | 5 | 12 | 7 | 12 | 9 | 3 |
| 102 | 1 | 3 | 14 | 0 | 6 | 8 | 7 | 8 | 5 | 6 | 9 | 4 |
| 103 | 3 | 3 | 3 | 3 | 6 | 7 | 10 | 7 | 9 | 4 | 6 | 4 |
| 104 | 6 | 7 | 6 | 5 | 7 | 10 | 5 | 5 | 7 | 12 | 7 | 4 |
| 105 | 2 | 5 | 5 | 3 | 4 | 9 | 7 | 7 | 4 | 3 | 5 | 3 |

Table 52. Number of gastric cancer surgery during each month in 2020.

|  | Month | | | | | | | | | | | |
| --- | --- | --- | --- | --- | --- | --- | --- | --- | --- | --- | --- | --- |
| Hospital | 1 | 2 | 3 | 4 | 5 | 6 | 7 | 8 | 9 | 10 | 11 | 12 |
| 1 | 1 | 3 | 2 | 2 | 2 | 1 | 2 | 2 | 1 | 5 | 0 | 7 |
| 2 | 46 | 50 | 44 | 45 | 47 | 38 | 51 | 46 | 44 | 42 | 19 | 19 |
| 3 | 2 | 2 | 3 | 3 | 0 | 1 | 0 | 1 | 2 | 2 | 2 | 0 |
| 4 | 4 | 8 | 2 | 3 | 2 | 4 | 4 | 3 | 6 | 3 | 2 | 2 |
| 5 | 5 | 6 | 4 | 2 | 2 | 6 | 10 | 6 | 4 | 7 | 2 | 4 |
| 6 | 6 | 9 | 7 | 7 | 3 | 5 | 6 | 3 | 4 | 3 | 4 | 8 |
| 7 | 7 | 5 | 7 | 5 | 6 | 5 | 9 | 7 | 6 | 7 | 7 | 5 |
| 8 | 14 | 10 | 9 | 8 | 13 | 10 | 10 | 11 | 13 | 8 | 14 | 7 |
| 9 | 2 | 2 | 5 | 2 | 1 | 3 | 5 | 0 | 6 | 4 | 2 | 5 |
| 10 | 10 | 7 | 5 | 6 | 7 | 4 | 9 | 9 | 5 | 1 | 1 | 4 |
| 11 | 12 | 13 | 15 | 13 | 10 | 12 | 11 | 10 | 10 | 8 | 5 | 5 |
| 12 | 1 | 1 | 3 | 2 | 1 | 4 | 4 | 4 | 4 | 2 | 2 | 2 |
| 13 | 6 | 6 | 12 | 11 | 12 | 7 | 13 | 8 | 7 | 7 | 3 | 5 |
| 14 | 4 | 4 | 5 | 3 | 2 | 4 | 9 | 6 | 3 | 3 | 1 | 3 |
| 15 | 16 | 14 | 14 | 15 | 16 | 15 | 18 | 11 | 17 | 15 | 14 | 14 |
| 16 | 27 | 24 | 17 | 17 | 14 | 16 | 21 | 20 | 21 | 22 | 11 | 14 |
| 17 | 0 | 0 | 0 | 0 | 0 | 0 | 0 | 0 | 0 | 0 | 0 | 0 |
| 18 | 1 | 1 | 0 | 7 | 4 | 2 | 2 | 0 | 1 | 2 | 2 | 1 |
| 19 | 3 | 4 | 1 | 5 | 2 | 3 | 3 | 1 | 2 | 3 | 2 | 2 |
| 20 | 34 | 24 | 39 | 38 | 24 | 33 | 36 | 21 | 28 | 31 | 14 | 19 |
| 21 | 6 | 3 | 4 | 7 | 4 | 4 | 6 | 3 | 4 | 8 | 5 | 4 |
| 22 | 5 | 4 | 4 | 4 | 3 | 5 | 8 | 6 |  |  | 1 | 1 |
| 23 | 54 | 49 | 38 | 48 | 42 | 32 | 44 | 41 | 47 | 38 | 24 | 26 |
| 24 | 6 | 11 | 5 | 14 | 8 | 8 | 9 | 9 | 6 | 8 | 10 | 10 |
| 25 | 15 | 10 | 8 | 10 | 6 | 9 | 14 | 11 | 6 | 9 | 4 | 9 |
| 26 | 6 | 7 | 6 | 5 | 8 | 6 | 4 | 6 | 7 | 4 | 6 | 3 |
| 27 | 0 | 0 | 0 | 0 | 0 | 0 | 0 | 0 | 0 | 0 | 0 | 0 |
| 28 | 2 | 5 | 2 | 7 | 9 | 11 | 8 | 5 | 7 | 1 | 2 | 4 |
| 29 | 12 | 10 | 12 | 10 | 7 | 5 | 11 | 8 | 8 | 12 | 10 | 10 |
| 30 | 9 | 4 | 9 | 7 | 5 | 5 | 7 | 9 | 7 | 7 | 2 | 9 |
| 31 | 5 | 6 | 4 | 4 | 6 | 2 | 6 | 4 | 6 | 6 | 4 | 0 |
| 32 | 6 | 2 | 2 | 2 | 3 | 1 | 2 |  | 1 | 2 | 1 | 1 |
| 33 | 0 | 3 | 4 | 2 | 2 | 0 | 3 | 6 | 3 | 1 | 5 | 4 |
| 34 |  |  |  |  |  |  |  |  |  |  |  |  |
| 35 | 9 | 11 | 3 | 10 | 10 | 5 | 5 | 6 | 11 | 9 | 7 | 4 |
| 36 | 9 | 6 | 4 | 6 | 5 | 6 | 9 | 10 | 3 | 10 | 5 | 7 |
| 37 | 16 | 15 | 16 | 19 | 17 | 17 | 19 | 17 | 16 | 15 | 10 | 13 |
| 38 | 8 | 5 | 3 | 3 | 2 | 5 | 5 | 1 | 3 | 1 | 1 | 6 |
| 39 | 2 | 1 |  | 2 |  | 2 | 2 | 2 |  | 2 |  |  |
| 40 | 7 | 5 | 10 | 7 | 3 | 5 | 1 | 2 | 5 | 5 | 4 | 1 |
| 41 | 11 | 15 | 18 | 10 | 16 | 8 | 13 | 11 | 16 | 17 | 14 | 11 |
| 42 | 5 | 3 | 3 | 3 | 3 | 2 | 5 | 3 | 3 | 3 | 3 | 0 |
| 43 | 7 | 7 | 3 | 7 | 7 | 8 | 8 | 5 | 4 | 2 | 5 | 5 |
| 44 | 0 | 0 | 0 | 0 | 0 | 0 | 0 | 0 | 0 | 0 | 0 | 0 |
| 45 | 4 | 6 | 3 | 6 | 8 | 8 | 7 | 7 | 6 | 7 | 1 | 2 |
| 46 | 3 | 3 | 4 | 4 | 7 | 5 | 8 | 11 | 10 | 11 | 3 | 9 |
| 47 | 3 | 8 | 2 | 7 | 7 | 11 | 2 | 3 | 5 | 9 | 5 | 4 |
| 48 | 4 | 0 | 2 | 6 | 6 | 7 | 4 | 8 | 2 | 9 | 6 | 4 |
| 49 | 7 | 8 | 10 | 9 | 5 | 7 | 10 | 4 | 3 | 1 | 4 | 5 |
| 50 | 1 | 0 | 0 | 1 | 1 | 2 | 0 | 0 | 0 | 0 | 0 | 0 |
| 51 | 11 | 11 | 12 | 13 | 18 | 16 | 16 | 11 | 13 | 10 | 8 | 8 |
| 52 |  |  |  |  | 1 |  |  | 1 |  | 2 |  | 1 |
| 53 | 10 | 12 | 10 | 10 | 8 | 6 | 14 | 8 | 18 | 12 | 10 | 13 |
| 54 | 39 | 37 | 29 | 34 | 24 | 26 | 37 | 30 | 33 | 36 | 21 | 26 |
| 55 | 5 | 2 | 3 | 3 | 5 | 3 | 7 | 2 | 2 | 2 | 3 | 4 |
| 56 | 14 | 17 | 12 | 12 | 13 | 7 | 10 | 7 | 15 | 5 | 3 | 7 |
| 57 | 18 | 13 | 15 | 13 | 14 | 12 | 14 | 11 | 17 | 13 | 6 | 16 |
| 58 | 3 | 5 | 9 | 8 | 12 | 12 | 9 | 7 | 16 | 15 | 10 | 10 |
| 59 | 5 | 1 | 3 | 3 | 2 | 1 | 1 | 4 | 4 | 3 | 3 | 1 |
| 60 | 4 | 6 | 4 | 1 | 4 | 3 | 0 | 1 | 2 | 1 | 0 | 2 |
| 61 | 6 | 4 | 6 | 6 | 5 | 2 | 4 | 5 | 5 | 3 | 5 | 4 |
| 62 |  |  |  |  |  |  |  |  |  |  |  |  |
| 63 | 5 | 6 | 5 | 3 | 7 | 3 | 1 | 6 | 7 | 1 | 1 | 7 |
| 64 | 12 | 5 | 13 | 12 | 14 | 12 | 10 | 8 | 6 | 9 | 5 | 5 |
| 65 |  |  |  |  |  |  |  |  |  |  |  |  |
| 66 | 1 | 1 | 0 | 2 | 2 | 0 | 1 | 1 | 1 | 0 | 0 | 0 |
| 67 |  |  |  |  |  |  |  |  |  |  |  |  |
| 68 | 16 | 11 | 7 | 11 | 11 | 13 | 16 | 17 | 17 | 13 | 6 | 7 |
| 68 | 1 | 1 | 0 | 0 | 0 | 0 | 0 | 2 | 0 | 11 | 10 | 7 |
| 70 |  |  | 1 | 2 |  |  |  |  |  |  |  |  |
| 71 | 6 | 9 | 9 | 5 | 9 | 7 | 5 | 6 | 3 | 7 | 6 | 7 |
| 72 | 5 | 7 | 5 | 6 | 9 | 8 | 5 | 10 | 7 | 3 | 2 | 7 |
| 73 | 3 | 6 | 1 | 6 | 6 | 6 | 5 | 5 | 7 | 3 | 2 | 3 |
| 74 | 11 | 9 | 6 | 9 | 9 | 13 | 10 | 10 | 8 | 7 | 6 | 9 |
| 75 | 1 | 1 | 3 | 2 | 2 | 3 | 4 | 7 | 7 | 3 | 0 | 3 |
| 76 | 7 | 5 | 8 | 12 | 6 | 8 | 9 | 7 | 10 | 12 | 4 | 8 |
| 77 | 1 |  | 1 | 2 | 2 | 1 |  |  |  |  |  |  |
| 78 | 2 |  | 1 | 2 |  |  |  |  |  |  |  |  |
| 79 | 6 | 9 | 15 | 12 | 11 | 23 | 11 | 15 | 16 | 7 | 3 | 4 |
| 80 | 3 | 8 | 12 | 11 | 10 | 6 | 8 | 10 | 4 | 8 | 5 | 7 |
| 81 | 8 | 5 | 3 | 6 | 4 | 6 | 7 | 11 | 10 | 3 | 5 | 3 |
| 82 | 13 | 6 | 3 | 7 | 9 | 11 | 9 | 6 | 4 | 9 | 5 | 7 |
| 83 | 6 | 5 | 2 | 4 | 5 | 3 | 5 | 7 | 5 | 5 | 3 | 10 |
| 84 |  |  |  |  |  |  |  |  |  |  |  |  |
| 85 | 4 | 1 | 3 | 3 | 6 | 3 | 2 | 7 | 4 | 4 | 3 | 1 |
| 86 | 6 | 8 | 2 | 2 | 8 | 5 | 5 | 6 | 5 | 8 | 4 | 6 |
| 87 | 6 | 5 | 2 | 2 | 2 | 2 | 5 | 2 | 3 | 6 | 0 | 2 |
| 88 | 2 | 3 | 5 | 3 | 1 | 4 | 0 | 0 | 0 | 0 | 0 | 1 |
| 89 | 27 | 30 | 23 | 25 | 19 | 24 | 22 | 21 | 23 | 23 | 6 | 31 |
| 90 | 14 | 9 | 13 | 8 | 9 | 14 | 15 | 12 | 11 | 9 | 13 | 15 |
| 91 | 14 | 15 | 14 | 14 | 14 | 17 | 17 | 13 | 15 | 13 | 2 | 17 |
| 92 |  |  |  |  |  |  |  |  |  |  |  |  |
| 93 | 2 | 7 | 2 | 3 | 7 | 3 | 4 | 5 | 6 | 8 | 4 | 8 |
| 94 | 5 | 6 | 4 | 0 | 5 | 3 | 3 | 2 | 5 | 2 | 1 | 2 |
| 95 | 1 | 1 | 2 | 2 | 0 | 1 | 2 | 1 | 2 | 1 | 1 | 1 |
| 96 | 9 | 7 | 8 | 7 | 11 | 3 | 9 | 7 | 11 | 7 | 2 | 3 |
| 97 | 1 | 0 | 1 | 0 | 0 | 0 | 0 | 0 | 0 | 0 | 0 | 0 |
| 98 | 3 | 3 | 5 | 2 | 3 | 3 | 1 | 3 | 2 | 3 | 2 | 2 |
| 99 | 2 | 3 | 1 | 2 | 1 | 1 | 1 | 1 | 0 | 1 | 1 | 0 |
| 100 | 10 | 5 | 9 | 6 | 10 | 7 | 5 | 8 | 7 | 0 | 6 | 11 |
| 101 | 5 | 7 | 7 | 8 | 8 | 7 | 8 | 8 | 8 | 5 | 6 | 6 |
| 102 | 9 | 5 | 6 | 1 | 7 | 6 | 3 | 5 | 8 | 1 | 0 | 10 |
| 103 | 12 | 3 | 10 | 1 | 11 | 9 | 7 | 8 | 8 | 11 | 8 | 8 |
| 104 | 13 | 11 | 12 | 13 | 20 | 12 | 12 | 15 | 6 | 8 | 8 | 10 |
| 105 | 8 | 4 | 3 | 5 | 4 | 8 | 6 | 4 | 4 | 5 | 3 | 7 |

Table 53. Number of gastric cancer endoscopic procedure during each month in 2019.

|  | Month | | | | | | | | | | | |
| --- | --- | --- | --- | --- | --- | --- | --- | --- | --- | --- | --- | --- |
| Hospital | 1 | 2 | 3 | 4 | 5 | 6 | 7 | 8 | 9 | 10 | 11 | 12 |
| 1 | 1 | 0 | 0 | 2 | 1 | 4 | 1 | 3 | 2 | 2 | 2 | 1 |
| 2 | 45 | 47 | 46 | 41 | 31 | 44 | 46 | 50 | 44 | 45 | 47 | 38 |
| 3 | 3 | 1 | 3 | 3 | 1 | 3 | 2 | 2 | 3 | 3 | 0 | 1 |
| 4 | 2 | 3 | 2 | 3 | 4 | 4 | 4 | 8 | 2 | 3 | 2 | 4 |
| 5 | 6 | 7 | 6 | 6 | 5 | 6 | 5 | 6 | 4 | 2 | 2 | 6 |
| 6 | 1 | 5 | 3 | 4 | 3 | 3 | 6 | 9 | 7 | 7 | 3 | 5 |
| 7 | 6 | 4 | 3 | 5 | 6 | 3 | 7 | 5 | 7 | 5 | 6 | 5 |
| 8 | 10 | 19 | 8 | 9 | 6 | 9 | 14 | 10 | 9 | 8 | 13 | 10 |
| 9 | 1 | 2 | 1 | 3 | 2 | 2 | 2 | 2 | 5 | 2 | 1 | 3 |
| 10 | 5 | 3 | 9 | 6 | 3 | 7 | 10 | 7 | 5 | 6 | 7 | 4 |
| 11 | 8 | 9 | 11 | 8 | 14 | 10 | 12 | 13 | 15 | 13 | 10 | 12 |
| 12 | 2 | 4 | 0 | 0 | 1 | 2 | 1 | 1 | 3 | 2 | 1 | 4 |
| 13 | 10 | 12 | 8 | 6 | 6 | 10 | 6 | 6 | 12 | 11 | 12 | 7 |
| 14 | 1 | 3 | 3 | 1 | 2 | 5 | 4 | 4 | 5 | 3 | 2 | 4 |
| 15 | 12 | 14 | 14 | 12 | 19 | 12 | 16 | 14 | 14 | 15 | 16 | 15 |
| 16 | 32 | 23 | 19 | 28 | 19 | 13 | 27 | 24 | 17 | 17 | 14 | 16 |
| 17 | 0 | 0 | 0 | 0 | 0 | 0 | 0 | 0 | 0 | 0 | 0 | 0 |
| 18 | 2 | 4 | 1 | 2 | 2 | 3 | 1 | 1 | 0 | 7 | 4 | 2 |
| 19 | 1 | 0 | 2 | 2 | 2 | 4 | 3 | 4 | 1 | 5 | 2 | 3 |
| 20 | 38 | 30 | 36 | 26 | 27 | 28 | 34 | 24 | 39 | 38 | 24 | 33 |
| 21 | 7 | 2 | 3 | 5 | 1 | 4 | 6 | 3 | 4 | 7 | 4 | 4 |
| 22 | 3 | 2 | 2 | 5 | 4 | 3 | 5 | 4 | 4 | 4 | 3 | 5 |
| 23 | 38 | 38 | 46 | 58 | 44 | 47 | 54 | 49 | 38 | 48 | 42 | 32 |
| 24 | 10 | 11 | 12 | 11 | 9 | 16 | 6 | 11 | 5 | 14 | 8 | 8 |
| 25 | 12 | 5 | 7 | 13 | 9 | 6 | 15 | 10 | 8 | 10 | 6 | 9 |
| 26 | 7 | 9 | 8 | 4 | 3 | 6 | 6 | 7 | 6 | 5 | 8 | 6 |
| 27 | 0 | 0 | 0 | 0 | 0 | 0 | 0 | 0 | 0 | 0 | 0 | 0 |
| 28 | 0 | 1 | 1 | 7 | 3 | 3 | 2 | 5 | 2 | 7 | 9 | 11 |
| 29 | 3 | 5 | 11 | 9 | 6 | 12 | 12 | 10 | 12 | 10 | 7 | 5 |
| 30 | 3 | 4 | 11 | 5 | 7 | 5 | 9 | 4 | 9 | 7 | 5 | 5 |
| 31 | 8 | 5 | 5 | 3 | 4 | 7 | 5 | 6 | 4 | 4 | 6 | 2 |
| 32 | 3 | 3 | 2 | 3 |  | 5 | 6 | 2 | 2 | 2 | 3 | 1 |
| 33 | 1 | 2 | 3 | 1 | 1 | 1 | 0 | 3 | 4 | 2 | 2 | 0 |
| 34 |  |  |  |  |  |  |  |  |  |  |  |  |
| 35 | 6 | 5 | 8 | 7 | 8 | 13 | 9 | 11 | 3 | 10 | 10 | 5 |
| 36 | 7 | 9 | 8 | 8 | 10 | 9 | 9 | 6 | 4 | 6 | 5 | 6 |
| 37 | 19 | 12 | 10 | 16 | 15 | 19 | 16 | 15 | 16 | 19 | 17 | 17 |
| 38 | 0 | 4 | 7 | 3 | 8 | 6 | 8 | 5 | 3 | 3 | 2 | 5 |
| 39 |  | 1 |  |  | 1 | 1 | 2 | 1 |  | 2 |  | 2 |
| 40 | 1 | 2 | 3 | 4 | 5 | 2 | 7 | 5 | 10 | 7 | 3 | 5 |
| 41 | 16 | 11 | 10 | 14 | 14 | 10 | 11 | 15 | 18 | 10 | 16 | 8 |
| 42 | 8 | 3 | 7 | 4 | 7 | 4 | 5 | 3 | 3 | 3 | 3 | 2 |
| 43 | 3 | 5 | 7 | 7 | 4 | 5 | 7 | 7 | 3 | 7 | 7 | 8 |
| 44 | 0 | 1 | 0 | 0 | 0 | 0 | 0 | 0 | 0 | 0 | 0 | 0 |
| 45 | 6 | 7 | 5 | 8 | 5 | 5 | 4 | 6 | 3 | 6 | 8 | 8 |
| 46 | 12 | 8 | 4 | 9 | 6 | 5 | 3 | 3 | 4 | 4 | 7 | 5 |
| 47 | 7 | 7 | 14 | 5 | 7 | 10 | 3 | 8 | 2 | 7 | 7 | 11 |
| 48 | 4 | 3 | 7 | 5 | 7 | 2 | 4 | 0 | 2 | 6 | 6 | 7 |
| 49 | 7 | 7 | 10 | 6 | 6 | 3 | 7 | 8 | 10 | 9 | 5 | 7 |
| 50 | 2 | 1 | 1 | 0 | 0 | 0 | 1 | 0 | 0 | 1 | 1 | 2 |
| 51 | 9 | 13 | 11 | 9 | 10 | 14 | 11 | 11 | 12 | 13 | 18 | 16 |
| 52 |  |  | 2 |  | 1 | 1 |  |  |  |  | 1 |  |
| 53 | 8 | 12 | 10 | 16 | 5 | 17 | 10 | 12 | 10 | 10 | 8 | 6 |
| 54 | 36 | 26 | 28 | 30 | 24 | 24 | 39 | 37 | 29 | 34 | 24 | 26 |
| 55 |  | 2 | 2 | 2 | 6 | 2 | 5 | 2 | 3 | 3 | 5 | 3 |
| 56 | 1 | 6 | 11 | 14 | 12 | 16 | 14 | 17 | 12 | 12 | 13 | 7 |
| 57 | 16 | 11 | 14 | 14 | 9 | 13 | 18 | 13 | 15 | 13 | 14 | 12 |
| 58 | 7 | 10 | 10 | 10 | 7 | 9 | 3 | 5 | 9 | 8 | 12 | 12 |
| 59 | 5 | 6 | 2 | 3 | 2 | 2 | 5 | 1 | 3 | 3 | 2 | 1 |
| 60 | 1 | 3 | 6 | 4 | 5 | 3 | 4 | 6 | 4 | 1 | 4 | 3 |
| 61 | 7 | 8 | 3 | 3 | 2 | 3 | 6 | 4 | 6 | 6 | 5 | 2 |
| 62 |  |  |  |  |  |  |  |  |  |  |  |  |
| 63 | 2 | 5 | 1 | 6 | 2 | 4 | 5 | 6 | 5 | 3 | 7 | 3 |
| 64 | 6 | 9 | 10 | 8 | 6 | 10 | 12 | 5 | 13 | 12 | 14 | 12 |
| 65 |  |  |  |  |  |  |  |  |  |  |  |  |
| 66 | 2 | 1 | 0 | 2 | 1 | 1 | 1 | 1 | 0 | 2 | 2 | 0 |
| 67 |  |  |  |  |  |  |  |  |  |  |  |  |
| 68 | 10 | 11 | 12 | 13 | 11 | 14 | 16 | 11 | 7 | 11 | 11 | 13 |
| 68 | 1 | 1 | 0 | 0 | 0 | 0 | 1 | 1 | 0 | 0 | 0 | 0 |
| 70 | 1 |  |  |  | 2 | 2 |  |  | 1 | 2 |  |  |
| 71 | 5 | 6 | 7 | 9 | 8 | 11 | 6 | 9 | 9 | 5 | 9 | 7 |
| 72 | 5 | 7 | 8 | 3 | 8 | 7 | 5 | 7 | 5 | 6 | 9 | 8 |
| 73 | 7 | 5 | 7 | 4 | 5 | 8 | 3 | 6 | 1 | 6 | 6 | 6 |
| 74 | 7 | 10 | 8 | 13 | 6 | 8 | 11 | 9 | 6 | 9 | 9 | 13 |
| 75 | 5 | 4 | 2 | 5 | 3 | 1 | 1 | 1 | 3 | 2 | 2 | 3 |
| 76 | 12 | 13 | 8 | 3 | 3 | 7 | 7 | 5 | 8 | 12 | 6 | 8 |
| 77 |  |  | 1 | 1 |  |  | 1 |  | 1 | 2 | 2 | 1 |
| 78 | 2 |  |  |  |  |  | 2 |  | 1 | 2 |  |  |
| 79 | 9 | 12 | 9 | 9 | 8 | 10 | 6 | 9 | 15 | 12 | 11 | 23 |
| 80 | 7 | 11 | 7 | 13 | 11 | 10 | 3 | 8 | 12 | 11 | 10 | 6 |
| 81 | 7 | 8 | 6 | 6 | 7 | 7 | 8 | 5 | 3 | 6 | 4 | 6 |
| 82 | 1 | 4 | 5 | 6 | 6 | 9 | 13 | 6 | 3 | 7 | 9 | 11 |
| 83 | 4 | 4 | 3 | 5 | 5 | 4 | 6 | 5 | 2 | 4 | 5 | 3 |
| 84 |  |  |  |  |  |  |  |  |  |  |  |  |
| 85 | 4 | 4 | 4 | 0 | 5 | 1 | 4 | 1 | 3 | 3 | 6 | 3 |
| 86 | 5 | 7 | 3 | 6 | 4 | 6 | 6 | 8 | 2 | 2 | 8 | 5 |
| 87 | 3 | 3 | 2 | 3 | 1 | 2 | 6 | 5 | 2 | 2 | 2 | 2 |
| 88 | 1 | 2 | 0 | 0 | 1 | 2 | 2 | 3 | 5 | 3 | 1 | 4 |
| 89 | 23 | 26 | 23 | 25 | 21 | 27 | 27 | 30 | 23 | 25 | 19 | 24 |
| 90 | 6 | 8 | 9 | 10 | 14 | 14 | 14 | 9 | 13 | 8 | 9 | 14 |
| 91 | 11 | 16 | 14 | 9 | 15 | 12 | 14 | 15 | 14 | 14 | 14 | 17 |
| 92 |  |  |  |  |  |  |  |  |  |  |  |  |
| 93 | 4 | 3 | 5 | 3 | 6 | 3 | 2 | 7 | 2 | 3 | 7 | 3 |
| 94 | 2 | 0 | 2 | 0 | 1 | 2 | 5 | 6 | 4 | 0 | 5 | 3 |
| 95 | 2 | 2 | 0 | 2 | 0 | 0 | 1 | 1 | 2 | 2 | 0 | 1 |
| 96 | 13 | 10 | 10 | 10 | 5 | 7 | 9 | 7 | 8 | 7 | 11 | 3 |
| 97 | 0 | 0 | 0 | 0 | 1 | 0 | 1 | 0 | 1 | 0 | 0 | 0 |
| 98 | 4 | 5 | 6 | 5 | 2 | 3 | 3 | 3 | 5 | 2 | 3 | 3 |
| 99 | 2 | 0 | 2 | 1 | 2 | 0 | 2 | 3 | 1 | 2 | 1 | 1 |
| 100 | 2 | 3 | 4 | 4 | 6 | 7 | 10 | 5 | 9 | 6 | 10 | 7 |
| 101 | 10 | 7 | 8 | 10 | 4 | 5 | 5 | 7 | 7 | 8 | 8 | 7 |
| 102 | 7 | 3 | 8 | 8 | 3 | 6 | 9 | 5 | 6 | 1 | 7 | 6 |
| 103 | 5 | 2 | 4 | 5 | 11 | 6 | 12 | 3 | 10 | 1 | 11 | 9 |
| 104 | 14 | 16 | 9 | 14 | 14 | 7 | 13 | 11 | 12 | 13 | 20 | 12 |
| 105 | 7 | 3 | 5 | 5 | 6 | 6 | 8 | 4 | 3 | 5 | 4 | 8 |

Table 54. Number of gastric cancer endoscopic procedure during each month in 2020.

|  | Month | | | | | | | | | | | |
| --- | --- | --- | --- | --- | --- | --- | --- | --- | --- | --- | --- | --- |
| Hospital | 1 | 2 | 3 | 4 | 5 | 6 | 7 | 8 | 9 | 10 | 11 | 12 |
| 1 | 2 | 2 | 1 | 5 | 0 | 7 | 1 | 4 | 2 | 3 | 2 | 2 |
| 2 | 51 | 46 | 44 | 42 | 19 | 19 | 38 | 34 | 38 | 49 | 41 | 36 |
| 3 | 0 | 1 | 2 | 2 | 2 | 0 | 3 | 1 | 4 | 6 | 2 | 4 |
| 4 | 4 | 3 | 6 | 3 | 2 | 2 | 5 | 1 | 0 | 3 | 5 | 2 |
| 5 | 10 | 6 | 4 | 7 | 2 | 4 | 5 | 3 | 2 | 7 | 3 | 7 |
| 6 | 6 | 3 | 4 | 3 | 4 | 8 | 5 | 3 | 5 | 11 | 1 | 7 |
| 7 | 9 | 7 | 6 | 7 | 7 | 5 | 6 | 7 | 5 | 9 | 5 | 5 |
| 8 | 10 | 11 | 13 | 8 | 14 | 7 | 8 | 4 | 7 | 19 | 15 | 8 |
| 9 | 5 | 0 | 6 | 4 | 2 | 5 | 2 | 3 | 0 | 1 | 2 | 1 |
| 10 | 9 | 9 | 5 | 1 | 1 | 4 | 3 | 7 | 5 | 7 | 9 | 5 |
| 11 | 11 | 10 | 10 | 8 | 5 | 5 | 5 | 9 | 6 | 8 | 8 | 10 |
| 12 | 4 | 4 | 4 | 2 | 2 | 2 | 2 | 3 | 4 | 1 | 1 | 1 |
| 13 | 13 | 8 | 7 | 7 | 3 | 5 | 6 | 6 | 10 | 8 | 7 | 5 |
| 14 | 9 | 6 | 3 | 3 | 1 | 3 | 2 | 2 | 3 | 8 | 6 | 6 |
| 15 | 18 | 11 | 17 | 15 | 14 | 14 | 11 | 15 | 12 | 12 | 13 | 14 |
| 16 | 21 | 20 | 21 | 22 | 11 | 14 | 19 | 22 | 19 | 18 | 25 | 21 |
| 17 | 0 | 0 | 0 | 0 | 0 | 0 | 0 | 0 | 0 | 0 | 0 | 0 |
| 18 | 2 | 0 | 1 | 2 | 2 | 1 | 3 | 1 | 1 | 2 | 2 | 5 |
| 19 | 3 | 1 | 2 | 3 | 2 | 2 | 3 | 0 | 2 | 3 | 3 | 1 |
| 20 | 36 | 21 | 28 | 31 | 14 | 19 | 27 | 34 | 31 | 24 | 21 | 51 |
| 21 | 6 | 3 | 4 | 8 | 5 | 4 | 5 | 5 | 6 | 7 | 3 | 3 |
| 22 | 8 | 6 |  |  | 1 | 1 | 8 | 3 | 1 | 3 | 4 | 3 |
| 23 | 44 | 41 | 47 | 38 | 24 | 26 | 25 | 34 | 29 | 49 | 47 | 42 |
| 24 | 9 | 9 | 6 | 8 | 10 | 10 | 9 | 7 | 6 | 8 | 8 | 11 |
| 25 | 14 | 11 | 6 | 9 | 4 | 9 | 8 | 7 | 7 | 7 | 7 | 8 |
| 26 | 4 | 6 | 7 | 4 | 6 | 3 | 3 | 3 | 5 | 2 | 2 | 4 |
| 27 | 0 | 0 | 0 | 0 | 0 | 0 | 0 | 0 | 0 | 0 | 0 | 0 |
| 28 | 8 | 5 | 7 | 1 | 2 | 4 | 3 | 4 | 6 | 4 | 6 | 6 |
| 29 | 11 | 8 | 8 | 12 | 10 | 10 | 5 | 5 | 9 | 11 | 6 | 10 |
| 30 | 7 | 9 | 7 | 7 | 2 | 9 | 3 | 5 | 4 | 8 | 8 | 6 |
| 31 | 6 | 4 | 6 | 6 | 4 | 0 | 3 | 0 | 4 | 1 | 3 | 2 |
| 32 | 2 |  | 1 | 2 | 1 | 1 | 1 | 1 |  | 2 | 3 |  |
| 33 | 3 | 6 | 3 | 1 | 5 | 4 | 8 | 6 | 6 | 10 | 2 | 0 |
| 34 |  |  |  |  |  |  |  |  |  |  |  |  |
| 35 | 5 | 6 | 11 | 9 | 7 | 4 | 4 | 6 | 8 | 11 | 5 | 6 |
| 36 | 9 | 10 | 3 | 10 | 5 | 7 | 9 | 8 | 11 | 7 | 9 | 7 |
| 37 | 19 | 17 | 16 | 15 | 10 | 13 | 11 | 6 | 15 | 14 | 11 | 13 |
| 38 | 5 | 1 | 3 | 1 | 1 | 6 | 4 | 8 | 1 | 4 | 3 | 4 |
| 39 | 2 | 2 |  | 2 |  |  | 1 | 1 | 3 | 2 | 1 | 2 |
| 40 | 1 | 2 | 5 | 5 | 4 | 1 | 5 | 3 | 4 | 7 | 2 | 1 |
| 41 | 13 | 11 | 16 | 17 | 14 | 11 | 11 | 12 | 12 | 15 | 11 | 12 |
| 42 | 5 | 3 | 3 | 3 | 3 | 0 | 2 | 2 | 3 | 1 | 5 | 1 |
| 43 | 8 | 5 | 4 | 2 | 5 | 5 | 8 | 5 | 8 | 8 | 4 | 3 |
| 44 | 0 | 0 | 0 | 0 | 0 | 0 | 0 | 0 | 0 | 0 | 0 | 0 |
| 45 | 7 | 7 | 6 | 7 | 1 | 2 | 2 | 5 | 2 | 5 | 7 | 4 |
| 46 | 8 | 11 | 10 | 11 | 3 | 9 | 2 | 2 | 3 | 6 | 8 | 7 |
| 47 | 2 | 3 | 5 | 9 | 5 | 4 | 2 | 5 | 3 | 3 | 2 | 6 |
| 48 | 4 | 8 | 2 | 9 | 6 | 4 | 1 | 3 | 3 | 8 | 4 | 6 |
| 49 | 10 | 4 | 3 | 1 | 4 | 5 | 3 | 8 | 5 | 10 | 7 | 2 |
| 50 | 0 | 0 | 0 | 0 | 0 | 0 | 2 | 2 | 0 | 1 | 1 | 0 |
| 51 | 16 | 11 | 13 | 10 | 8 | 8 | 19 | 14 | 18 | 20 | 12 | 14 |
| 52 |  | 1 |  | 2 |  | 1 | 3 | 2 | 1 |  | 1 |  |
| 53 | 14 | 8 | 18 | 12 | 10 | 13 | 8 | 8 | 7 | 17 | 11 | 9 |
| 54 | 37 | 30 | 33 | 36 | 21 | 26 | 20 | 24 | 19 | 35 | 27 | 33 |
| 55 | 7 | 2 | 2 | 2 | 3 | 4 | 2 | 1 | 4 | 3 | 1 | 4 |
| 56 | 10 | 7 | 15 | 5 | 3 | 7 | 7 | 15 | 13 | 16 | 13 | 11 |
| 57 | 14 | 11 | 17 | 13 | 6 | 16 | 7 | 8 | 12 | 15 | 11 | 13 |
| 58 | 9 | 7 | 16 | 15 | 10 | 10 | 13 | 6 | 8 | 11 | 12 | 12 |
| 59 | 1 | 4 | 4 | 3 | 3 | 1 | 1 | 2 | 0 | 4 | 2 | 1 |
| 60 | 0 | 1 | 2 | 1 | 0 | 2 | 1 | 0 | 1 | 1 | 3 | 2 |
| 61 | 4 | 5 | 5 | 3 | 5 | 4 | 2 | 5 | 2 | 3 | 3 | 6 |
| 62 |  |  |  |  |  |  |  |  |  |  |  |  |
| 63 | 1 | 6 | 7 | 1 | 1 | 7 | 7 | 4 | 6 | 3 | 1 | 4 |
| 64 | 10 | 8 | 6 | 9 | 5 | 5 | 11 | 5 | 2 | 10 | 2 | 11 |
| 65 |  |  |  |  |  |  |  |  |  |  |  |  |
| 66 | 1 | 1 | 1 | 0 | 0 | 0 | 0 | 1 | 1 | 1 | 1 | 0 |
| 67 |  |  |  |  |  |  |  |  |  |  |  |  |
| 68 | 16 | 17 | 17 | 13 | 6 | 7 | 15 | 5 | 12 | 9 | 5 | 12 |
| 68 | 0 | 2 | 0 | 11 | 10 | 7 | 13 | 15 | 11 | 13 | 13 | 14 |
| 70 |  |  |  |  |  |  |  |  |  |  |  |  |
| 71 | 5 | 6 | 3 | 7 | 6 | 7 | 7 | 9 | 6 | 5 | 5 | 6 |
| 72 | 5 | 10 | 7 | 3 | 2 | 7 | 6 | 5 | 7 | 9 | 3 | 7 |
| 73 | 5 | 5 | 7 | 3 | 2 | 3 | 3 | 0 | 4 | 0 | 0 | 0 |
| 74 | 10 | 10 | 8 | 7 | 6 | 9 | 11 | 10 | 12 | 13 | 8 | 7 |
| 75 | 4 | 7 | 7 | 3 | 0 | 3 | 2 | 2 | 0 | 0 | 0 | 0 |
| 76 | 9 | 7 | 10 | 12 | 4 | 8 | 4 | 2 | 11 | 8 | 6 | 6 |
| 77 |  |  |  |  |  |  | 1 | 1 | 1 | 2 |  | 1 |
| 78 |  |  |  |  |  |  | 2 | 1 |  |  | 2 |  |
| 79 | 11 | 15 | 16 | 7 | 3 | 4 | 5 | 6 | 10 | 11 | 14 | 13 |
| 80 | 8 | 10 | 4 | 8 | 5 | 7 | 8 | 7 | 10 | 6 | 7 | 2 |
| 81 | 7 | 11 | 10 | 3 | 5 | 3 | 5 | 3 | 4 | 3 | 8 | 7 |
| 82 | 9 | 6 | 4 | 9 | 5 | 7 | 9 | 7 | 5 | 15 | 3 | 6 |
| 83 | 5 | 7 | 5 | 5 | 3 | 10 | 5 | 12 | 11 | 10 | 10 | 8 |
| 84 |  |  |  |  |  |  |  |  |  |  |  |  |
| 85 | 2 | 7 | 4 | 4 | 3 | 1 | 1 | 3 | 3 | 2 | 1 | 3 |
| 86 | 5 | 6 | 5 | 8 | 4 | 6 | 3 | 1 | 3 | 7 | 5 | 6 |
| 87 | 5 | 2 | 3 | 6 | 0 | 2 | 1 | 4 | 0 | 0 | 4 | 2 |
| 88 | 0 | 0 | 0 | 0 | 0 | 1 | 0 | 0 | 1 | 0 | 0 | 0 |
| 89 | 22 | 21 | 23 | 23 | 6 | 31 | 23 | 21 | 21 | 29 | 24 | 25 |
| 90 | 15 | 12 | 11 | 9 | 13 | 15 | 8 | 7 | 16 | 17 | 14 | 16 |
| 91 | 17 | 13 | 15 | 13 | 2 | 17 | 16 | 17 | 14 | 16 | 12 | 12 |
| 92 |  |  |  |  |  |  |  |  |  |  |  |  |
| 93 | 4 | 5 | 6 | 8 | 4 | 8 | 5 | 3 | 5 | 3 | 3 | 6 |
| 94 | 3 | 2 | 5 | 2 | 1 | 2 | 3 | 0 | 4 | 5 | 3 | 1 |
| 95 | 2 | 1 | 2 | 1 | 1 | 1 | 2 | 0 | 0 | 1 | 0 | 3 |
| 96 | 9 | 7 | 11 | 7 | 2 | 3 | 4 | 12 | 0 | 6 | 3 | 4 |
| 97 | 0 | 0 | 0 | 0 | 0 | 0 | 0 | 0 | 1 | 0 | 1 | 0 |
| 98 | 1 | 3 | 2 | 3 | 2 | 2 | 2 | 4 | 5 | 4 | 0 | 2 |
| 99 | 1 | 1 | 0 | 1 | 1 | 0 | 2 | 1 | 2 | 4 | 2 | 1 |
| 100 | 5 | 8 | 7 | 0 | 6 | 11 | 7 | 8 | 8 | 7 | 8 | 7 |
| 101 | 8 | 8 | 8 | 5 | 6 | 6 | 5 | 6 | 4 | 6 | 5 | 3 |
| 102 | 3 | 5 | 8 | 1 | 0 | 10 | 12 | 5 | 7 | 3 | 4 | 9 |
| 103 | 7 | 8 | 8 | 11 | 8 | 8 | 3 | 7 | 8 | 7 | 8 | 8 |
| 104 | 12 | 15 | 6 | 8 | 8 | 10 | 16 | 10 | 13 | 12 | 11 | 14 |
| 105 | 6 | 4 | 4 | 5 | 3 | 7 | 7 | 3 | 6 | 10 | 8 | 5 |

Table 55. Number of colorectal cancer surgery during each month in 2019.

|  | Month | | | | | | | | | | | |
| --- | --- | --- | --- | --- | --- | --- | --- | --- | --- | --- | --- | --- |
| Hospital | 1 | 2 | 3 | 4 | 5 | 6 | 7 | 8 | 9 | 10 | 11 | 12 |
| 1 | 5 | 9 | 5 | 2 | 4 | 3 | 3 | 3 | 3 | 9 | 6 | 2 |
| 2 | 104 | 101 | 111 | 93 | 95 | 82 | 103 | 101 | 81 | 88 | 84 | 91 |
| 3 | 2 | 11 | 4 | 7 | 7 | 4 | 7 | 8 | 4 | 10 | 8 | 6 |
| 4 | 14 | 12 | 16 | 9 | 17 | 13 | 12 | 11 | 12 | 9 | 11 | 11 |
| 5 | 9 | 8 | 6 | 13 | 5 | 7 | 8 | 11 | 9 | 6 | 6 | 6 |
| 6 | 10 | 10 | 7 | 15 | 4 | 9 | 17 | 9 | 11 | 10 | 13 | 9 |
| 7 | 13 | 11 | 14 | 9 | 10 | 11 | 11 | 11 | 6 | 13 | 11 | 11 |
| 8 |  |  |  |  |  |  |  |  |  |  |  |  |
| 9 | 13 | 10 | 9 | 8 | 14 | 11 | 12 | 12 | 12 | 14 | 13 | 14 |
| 10 | 10 | 11 | 14 | 13 | 7 | 12 | 14 | 12 | 11 | 12 | 11 | 12 |
| 11 | 9 | 10 | 11 | 12 | 13 | 11 | 12 | 14 | 16 | 20 | 16 | 12 |
| 12 | 1 | 3 | 3 | 1 | 4 | 2 | 1 | 6 | 4 | 5 | 3 | 5 |
| 13 | 12 | 16 | 22 | 18 | 17 | 13 | 12 | 14 | 17 | 14 | 11 | 19 |
| 14 | 15 | 14 | 14 | 14 | 15 | 15 | 22 | 18 | 10 | 19 | 8 | 8 |
| 15 | 16 | 15 | 23 | 21 | 12 | 16 | 13 | 16 | 11 | 14 | 17 | 16 |
| 16 | 21 | 13 | 11 | 10 | 11 | 9 | 13 | 18 | 12 | 16 | 9 | 10 |
| 17 | 0 | 0 | 0 | 0 | 0 | 0 | 0 | 0 | 0 | 0 | 0 | 0 |
| 18 | 12 | 7 | 5 | 8 | 10 | 6 | 5 | 12 | 12 | 8 | 14 | 10 |
| 19 | 8 | 6 | 6 | 3 | 1 | 6 | 6 | 3 | 9 | 1 | 5 | 7 |
| 20 | 27 | 31 | 32 | 27 | 30 | 29 | 33 | 36 | 29 | 35 | 31 | 30 |
| 21 | 7 | 14 | 14 | 15 | 9 | 14 | 13 | 19 | 9 | 13 | 12 | 16 |
| 22 | 3 | 3 | 8 | 9 | 9 | 7 | 10 | 1 | 8 | 8 | 7 | 5 |
| 23 |  |  |  |  |  |  |  |  |  |  |  |  |
| 24 | 10 | 10 | 13 | 14 | 15 | 11 | 13 | 17 | 12 | 18 | 20 | 13 |
| 25 | 10 | 10 | 16 | 18 | 14 | 14 | 15 | 8 | 18 | 15 | 16 | 16 |
| 26 | 4 | 7 | 6 | 5 | 3 | 3 | 4 | 4 | 1 | 5 | 4 | 2 |
| 27 | 4 | 3 | 5 | 6 | 3 | 6 | 3 | 5 | 7 | 2 | 3 | 3 |
| 28 | 3 | 7 | 9 | 6 | 4 | 9 | 5 | 3 | 6 | 10 | 9 | 6 |
| 29 | 10 | 10 | 10 | 14 | 11 | 18 | 16 | 14 | 11 | 14 | 11 | 14 |
| 30 | 7 | 6 | 7 | 8 | 8 | 4 | 7 | 4 | 9 | 13 | 8 | 4 |
| 31 | 11 | 8 | 13 | 5 | 9 | 11 | 8 | 5 | 12 | 9 | 9 | 6 |
| 32 | 10 | 2 | 4 | 6 | 5 | 2 | 4 | 2 | 7 | 8 | 6 | 4 |
| 33 | 9 | 15 | 10 | 11 | 14 | 11 | 7 | 12 | 9 | 11 | 9 | 15 |
| 34 | 11 | 12 | 9 | 11 | 22 | 14 | 15 | 14 | 13 | 14 | 14 | 9 |
| 35 | 21 | 22 | 15 | 17 | 19 | 23 | 14 | 12 | 18 | 20 | 19 | 14 |
| 36 | 7 | 10 | 7 | 5 | 6 | 7 | 8 | 11 | 11 | 9 | 12 | 8 |
| 37 | 20 | 18 | 12 | 14 | 16 | 14 | 21 | 12 | 12 | 24 | 16 | 18 |
| 38 | 5 | 13 | 14 | 18 | 15 | 19 | 18 | 20 | 12 | 14 | 12 | 17 |
| 39 | 3 | 5 | 5 | 1 | 5 | 4 | 1 | 5 | 3 | 2 |  | 1 |
| 40 | 17 | 16 | 15 | 13 | 14 | 18 | 20 | 10 | 16 | 14 | 14 | 20 |
| 41 | 4 | 4 | 8 | 8 | 4 | 6 | 7 | 6 | 10 | 7 | 5 | 7 |
| 42 | 15 | 10 | 7 | 9 | 7 | 6 | 18 | 9 | 4 | 10 | 10 | 8 |
| 43 | 6 | 8 | 13 | 13 | 12 | 12 | 14 | 15 | 13 | 10 | 16 | 19 |
| 44 | 6 | 8 | 4 | 7 | 4 | 5 | 10 | 5 | 5 | 3 | 4 | 3 |
| 45 | 16 | 5 | 8 | 7 | 10 | 24 | 20 | 14 | 10 | 13 | 9 | 8 |
| 46 | 16 | 10 | 16 | 19 | 15 | 14 | 18 | 19 | 12 | 16 | 11 | 13 |
| 47 | 2 | 1 |  | 1 | 3 | 8 | 4 | 5 | 4 | 6 | 1 | 3 |
| 48 | 7 | 8 | 7 | 15 | 21 | 13 | 17 | 12 | 13 | 14 | 15 | 16 |
| 49 | 7 | 10 | 8 | 5 | 8 | 5 | 13 | 4 | 7 | 10 | 4 | 10 |
| 50 | 5 | 1 | 1 | 1 | 0 | 2 | 6 | 2 | 1 | 1 | 5 | 1 |
| 51 | 12 | 18 | 18 | 19 | 15 | 18 | 18 | 21 | 16 | 16 | 19 | 8 |
| 52 | 13 | 17 | 13 | 18 | 20 | 17 | 10 | 17 | 9 | 13 | 16 | 15 |
| 53 | 17 | 24 | 16 | 17 | 13 | 17 | 17 | 22 | 13 | 15 | 17 | 22 |
| 54 | 42 | 52 | 48 | 48 | 47 | 42 | 31 | 41 | 30 | 42 | 43 | 55 |
| 55 | 3 | 8 | 12 | 7 | 11 | 9 | 8 | 8 | 8 | 9 | 6 | 11 |
| 56 | 4 | 15 | 12 | 16 | 17 | 12 | 10 | 17 | 11 | 14 | 17 | 13 |
| 57 | 32 | 35 | 33 | 33 | 30 | 30 | 25 | 36 | 22 | 27 | 26 | 24 |
| 58 | 5 | 7 | 8 | 11 | 8 | 9 | 12 | 13 | 10 | 8 | 6 | 7 |
| 59 | 4 | 6 | 5 | 5 | 5 | 8 | 8 | 8 | 3 | 15 | 3 | 7 |
| 60 | 4 | 6 | 10 | 15 | 10 | 13 | 15 | 19 | 11 | 15 | 16 | 13 |
| 61 | 13 | 11 | 11 | 10 | 11 | 13 | 14 | 8 | 5 | 12 | 9 | 12 |
| 62 | 9 | 11 | 18 | 18 | 12 | 11 | 12 | 8 | 13 | 11 | 11 | 16 |
| 63 |  | 5 | 7 | 3 | 4 | 7 | 5 | 5 | 5 | 5 | 3 | 8 |
| 64 | 13 | 13 | 9 | 10 | 11 | 10 | 7 | 9 | 17 | 13 | 10 | 12 |
| 65 | 17 | 21 | 17 | 13 | 19 | 12 | 14 | 29 | 14 | 20 | 19 | 20 |
| 66 | 6 | 8 | 8 | 6 | 6 | 6 | 11 | 10 | 9 | 15 | 8 | 11 |
| 67 | 7 | 5 | 5 | 5 | 7 | 5 | 7 | 7 | 8 | 6 | 8 | 3 |
| 68 | 16 | 18 | 28 | 17 | 16 | 18 | 22 | 20 | 7 | 17 | 13 | 18 |
| 68 | 20 | 17 | 16 | 19 | 16 | 20 | 19 | 17 | 19 | 16 | 19 | 16 |
| 70 | 7 | 7 | 4 | 4 | 4 | 8 | 6 |  | 5 | 6 | 5 | 3 |
| 71 | 4 | 10 | 14 | 7 | 11 | 8 | 11 | 9 | 8 | 5 | 8 | 6 |
| 72 | 26 | 22 | 13 | 10 | 18 | 12 | 24 | 12 | 18 | 15 | 16 | 14 |
| 73 | 11 | 14 | 13 | 8 | 10 | 13 | 14 | 7 | 8 | 10 | 9 | 12 |
| 74 | 15 | 17 | 16 | 21 | 21 | 20 | 13 | 17 | 19 | 14 | 16 | 13 |
| 75 | 1 | 2 | 2 | 0 | 1 | 1 | 1 | 1 | 3 | 0 | 1 | 2 |
| 76 | 15 | 10 | 6 | 8 | 7 | 6 | 7 | 10 | 9 | 16 | 12 | 11 |
| 77 |  | 2 | 1 | 1 | 1 | 5 | 5 | 6 | 1 | 4 | 4 | 2 |
| 78 | 1 | 1 |  | 2 | 1 | 2 | 3 |  |  |  |  |  |
| 79 | 10 | 2 | 9 | 4 | 9 | 4 | 6 | 14 | 12 | 5 | 14 | 6 |
| 80 | 6 | 8 | 9 | 9 | 11 | 9 | 7 | 14 | 16 | 10 | 10 | 10 |
| 81 | 10 | 10 | 16 | 11 | 8 | 9 | 9 | 14 | 11 | 14 | 16 | 15 |
| 82 |  |  |  |  |  |  |  |  |  |  |  |  |
| 83 | 6 | 9 | 3 | 14 | 5 | 6 | 5 | 7 | 9 | 5 | 4 | 7 |
| 84 |  |  |  |  |  |  |  |  |  |  |  |  |
| 85 | 15 | 5 | 6 | 15 | 10 | 9 | 8 | 11 | 5 | 12 | 8 | 9 |
| 86 | 22 | 18 | 25 | 24 | 18 | 19 | 25 | 18 | 20 | 27 | 17 | 16 |
| 87 | 11 | 10 | 12 | 6 | 10 | 8 | 9 | 6 | 6 | 6 | 6 | 3 |
| 88 | 11 | 8 | 8 | 9 | 17 | 8 | 15 | 9 | 9 | 7 | 17 | 12 |
| 89 | 36 | 38 | 45 | 42 | 42 | 42 | 41 | 37 | 31 | 41 | 38 | 33 |
| 90 | 15 | 10 | 14 | 16 | 20 | 13 | 15 | 22 | 14 | 18 | 16 | 16 |
| 91 | 21 | 17 | 15 | 30 | 19 | 15 | 19 | 16 | 19 | 24 | 22 | 17 |
| 92 |  |  |  |  |  |  |  |  |  |  |  |  |
| 93 | 10 | 12 | 12 | 8 | 5 | 12 | 10 | 9 | 5 | 14 | 8 | 5 |
| 94 | 2 | 1 | 3 | 1 | 0 | 4 | 9 | 1 | 3 | 4 | 2 | 2 |
| 95 | 6 | 9 | 4 | 3 | 5 | 4 | 4 | 5 | 2 | 5 | 6 | 3 |
| 96 | 11 | 10 | 7 | 15 | 9 | 8 | 10 | 12 | 7 | 13 | 11 | 8 |
| 97 | 5 | 3 | 1 | 0 | 2 | 5 | 6 | 4 | 5 | 3 | 5 | 3 |
| 98 | 7 | 11 | 6 | 3 | 4 | 9 | 8 | 7 | 7 | 8 | 6 | 2 |
| 99 | 6 | 7 | 8 | 12 | 4 | 5 | 5 | 4 | 6 | 6 | 6 | 10 |
| 100 | 1 | 4 | 3 | 6 | 7 | 5 | 5 | 5 | 8 | 5 | 10 | 7 |
| 101 | 10 | 6 | 13 | 12 | 15 | 16 | 12 | 11 | 8 | 12 | 13 | 15 |
| 102 |  |  |  |  |  |  |  |  |  |  |  |  |
| 103 | 17 | 7 | 13 | 10 | 8 | 13 | 14 | 16 | 16 | 14 | 11 | 12 |
| 104 | 14 | 12 | 13 | 13 | 13 | 15 | 10 | 10 | 14 | 13 | 11 | 12 |
| 105 | 12 | 13 | 8 | 15 | 14 | 9 | 12 | 9 | 8 | 8 | 9 | 14 |

Table 56. Number of colorectal cancer surgery during each month in 2020.

|  | Month | | | | | | | | | | | |
| --- | --- | --- | --- | --- | --- | --- | --- | --- | --- | --- | --- | --- |
| Hospital | 1 | 2 | 3 | 4 | 5 | 6 | 7 | 8 | 9 | 10 | 11 | 12 |
| 1 | 4 | 4 | 6 | 7 | 3 | 2 | 2 | 2 | 3 | 1 | 4 | 2 |
| 2 | 81 | 88 | 104 | 78 | 93 | 82 | 72 | 66 | 67 | 85 | 59 | 84 |
| 3 | 7 | 5 | 10 | 11 | 3 | 10 | 6 | 4 | 4 | 11 | 7 | 5 |
| 4 | 12 | 12 | 15 | 11 | 13 | 5 | 9 | 6 | 10 | 5 | 12 | 12 |
| 5 | 6 | 11 | 8 | 8 | 2 | 9 | 6 | 4 | 3 | 7 | 8 | 12 |
| 6 | 3 | 7 | 8 | 13 | 10 | 7 | 12 | 6 | 5 | 7 | 10 | 4 |
| 7 | 12 | 12 | 12 | 9 | 7 | 11 | 9 | 11 | 6 | 6 | 12 | 13 |
| 8 |  |  |  |  |  |  |  |  |  |  |  |  |
| 9 | 8 | 9 | 12 | 11 | 5 | 13 | 8 | 5 | 9 | 11 | 8 | 14 |
| 10 | 12 | 15 | 13 | 12 | 10 | 9 | 7 | 9 | 10 | 14 | 9 | 4 |
| 11 | 12 | 13 | 16 | 9 | 5 | 8 | 10 | 10 | 18 | 21 | 14 | 12 |
| 12 | 0 | 5 | 2 | 1 | 8 | 7 | 3 | 1 | 5 | 3 | 2 | 3 |
| 13 | 13 | 18 | 18 | 13 | 11 | 15 | 17 | 14 | 11 | 14 | 16 | 16 |
| 14 | 14 | 7 | 14 | 11 | 9 | 8 | 14 | 11 | 17 | 15 | 11 | 12 |
| 15 | 11 | 14 | 17 | 15 | 10 | 13 | 12 | 13 | 9 | 14 | 12 | 21 |
| 16 | 9 | 13 | 12 | 15 | 15 | 22 | 16 | 13 | 7 | 11 | 12 | 11 |
| 17 | 0 | 0 | 0 | 0 | 0 | 0 | 0 | 0 | 0 | 0 | 0 | 0 |
| 18 | 15 | 11 | 15 | 18 | 8 | 15 | 7 | 16 | 13 | 12 | 12 | 19 |
| 19 | 11 | 3 | 5 | 7 | 3 | 3 | 4 | 4 | 4 | 5 | 2 | 2 |
| 20 | 27 | 34 | 34 | 26 | 24 | 15 | 17 | 17 | 15 | 27 | 24 | 29 |
| 21 | 20 | 19 | 8 | 11 | 10 | 11 | 11 | 13 | 12 | 14 | 18 | 17 |
| 22 | 7 | 9 | 8 | 12 | 10 | 3 | 6 | 3 | 5 | 3 | 6 | 6 |
| 23 |  |  |  |  |  |  |  |  |  |  |  |  |
| 24 | 13 | 12 | 16 | 15 | 10 | 19 | 13 | 17 | 14 | 19 | 16 | 13 |
| 25 | 25 | 20 | 19 | 18 | 14 | 20 | 21 | 16 | 15 | 15 | 16 | 18 |
| 26 | 2 | 3 | 2 | 5 | 0 | 2 | 4 | 1 | 4 | 3 | 0 | 2 |
| 27 | 0 | 4 | 6 | 1 | 2 | 3 | 4 | 2 | 4 | 5 | 1 | 6 |
| 28 | 10 | 4 | 8 | 6 | 6 | 4 | 5 | 6 | 8 | 6 | 9 | 5 |
| 29 | 6 | 11 | 17 | 11 | 9 | 16 | 19 | 7 | 24 | 14 | 5 | 9 |
| 30 | 5 | 11 | 7 | 10 | 9 | 12 | 8 | 6 | 9 | 4 | 9 | 9 |
| 31 | 8 | 4 | 7 | 11 | 7 | 6 | 7 | 6 | 6 | 7 | 5 | 8 |
| 32 | 6 | 9 | 8 | 2 | 3 | 1 | 3 | 2 | 5 | 2 | 5 | 4 |
| 33 | 11 | 10 | 5 | 6 | 10 | 14 | 15 | 7 | 6 | 13 | 9 | 11 |
| 34 | 10 | 9 | 13 | 7 | 5 | 9 | 12 | 13 | 15 | 11 | 9 | 13 |
| 35 | 9 | 22 | 11 | 21 | 15 | 24 | 16 | 15 | 21 | 21 | 14 | 19 |
| 36 | 10 | 9 | 8 | 5 | 11 | 11 | 10 | 10 | 9 | 15 | 7 | 7 |
| 37 | 17 | 16 | 23 | 14 | 13 | 21 | 16 | 10 | 15 | 15 | 15 | 20 |
| 38 | 13 | 17 | 37 | 17 | 11 | 9 | 9 | 12 | 9 | 22 | 16 | 11 |
| 39 |  | 2 | 4 | 1 | 6 | 2 | 5 | 2 | 1 | 3 | 1 | 5 |
| 40 | 15 | 14 | 10 | 12 | 14 | 20 | 8 | 18 | 23 | 18 | 14 | 21 |
| 41 | 12 | 9 | 9 | 8 | 4 | 8 | 12 | 8 | 8 | 13 | 9 | 7 |
| 42 | 9 | 4 | 16 | 9 | 5 | 7 | 10 | 8 | 8 | 14 | 4 | 7 |
| 43 | 11 | 9 | 7 | 7 | 6 | 10 | 10 | 10 | 11 | 5 | 9 | 12 |
| 44 | 5 | 3 | 6 | 5 | 2 | 8 | 7 | 7 | 5 | 2 | 4 | 9 |
| 45 | 17 | 13 | 12 | 10 | 11 | 11 | 11 | 11 | 11 | 9 | 7 | 7 |
| 46 | 15 | 15 | 12 | 13 | 18 | 15 | 21 | 14 | 19 | 14 | 20 | 13 |
| 47 | 2 | 5 | 3 | 3 | 4 | 4 | 5 | 5 | 4 | 3 | 4 | 4 |
| 48 | 12 | 11 | 19 | 17 | 12 | 13 | 15 | 8 | 11 | 19 | 12 | 12 |
| 49 | 9 | 8 | 3 | 8 | 2 | 5 | 2 | 5 | 8 | 4 | 7 | 5 |
| 50 | 5 | 5 | 2 | 1 | 2 | 3 | 3 | 1 | 4 | 1 | 2 | 4 |
| 51 | 16 | 16 | 22 | 21 | 13 | 13 | 16 | 18 | 9 | 19 | 13 | 28 |
| 52 | 14 | 6 | 13 | 10 | 7 | 15 | 7 | 10 | 11 | 11 | 18 | 15 |
| 53 | 16 | 21 | 24 | 16 | 13 | 18 | 16 | 13 | 10 | 14 | 13 | 14 |
| 54 | 51 | 53 | 40 | 32 | 30 | 34 | 39 | 36 | 41 | 37 | 35 | 53 |
| 55 | 7 | 12 | 9 | 10 | 1 | 9 | 8 | 8 | 14 | 6 | 10 | 7 |
| 56 | 22 | 12 | 18 | 22 | 12 | 15 | 13 | 13 | 17 | 21 | 18 | 19 |
| 57 | 24 | 28 | 33 | 29 | 15 | 24 | 17 | 17 | 20 | 16 | 13 | 21 |
| 58 | 8 | 7 | 6 | 6 | 13 | 13 | 8 | 7 | 7 | 11 | 10 | 7 |
| 59 | 10 | 9 | 14 | 9 | 5 | 6 | 4 | 3 | 7 | 8 | 5 | 4 |
| 60 | 5 | 7 | 13 | 13 | 10 | 12 | 12 | 7 | 17 | 13 | 7 | 13 |
| 61 | 9 | 11 | 13 | 12 | 6 | 7 | 12 | 12 | 9 | 16 | 14 | 11 |
| 62 | 15 | 10 | 17 | 12 | 8 | 8 | 6 | 5 | 4 | 10 | 12 | 13 |
| 63 | 4 | 6 | 7 | 2 | 4 | 5 | 5 | 5 | 6 | 7 | 5 | 6 |
| 64 | 11 | 8 | 7 | 7 | 6 | 13 | 10 | 12 | 10 | 9 | 9 | 9 |
| 65 | 20 | 23 | 22 | 10 | 8 | 16 | 20 | 22 | 17 | 15 | 21 | 31 |
| 66 | 8 | 4 | 4 | 11 | 10 | 7 | 4 | 9 | 8 | 13 | 7 | 11 |
| 67 | 5 | 3 | 5 | 7 | 3 | 3 | 1 | 2 | 4 | 5 | 5 | 4 |
| 68 | 19 | 17 | 11 | 12 | 10 | 15 | 15 | 16 | 12 | 13 | 23 | 12 |
| 68 | 18 | 21 | 17 | 14 | 10 | 15 | 22 | 19 | 18 | 23 | 20 | 23 |
| 70 | 3 | 1 | 2 | 4 | 2 | 4 | 3 | 6 |  |  | 6 |  |
| 71 | 8 | 14 | 8 | 9 | 7 | 13 | 6 | 5 | 6 | 8 | 9 | 5 |
| 72 | 19 | 20 | 15 | 12 | 5 | 9 | 10 | 10 | 17 | 25 | 14 | 24 |
| 73 | 10 | 6 | 11 | 8 | 6 | 11 | 9 | 8 | 6 | 9 | 11 | 10 |
| 74 | 16 | 17 | 15 | 16 | 18 | 19 | 13 | 12 | 12 | 19 | 17 | 17 |
| 75 | 3 | 1 | 1 | 0 | 0 | 1 | 1 | 0 | 0 | 0 | 0 | 0 |
| 76 | 7 | 10 | 11 | 6 | 9 | 10 | 9 | 11 | 3 | 9 | 10 | 13 |
| 77 | 2 | 4 | 1 | 1 |  | 2 | 4 | 2 | 3 |  | 2 | 2 |
| 78 |  | 1 | 2 |  | 2 | 1 | 1 | 4 | 1 | 1 |  | 2 |
| 79 | 9 | 10 | 17 | 6 | 5 | 1 | 0 | 7 | 8 | 7 | 7 | 9 |
| 80 | 13 | 5 | 11 | 9 | 10 | 11 | 13 | 8 | 9 | 16 | 13 | 12 |
| 81 | 16 | 10 | 11 | 11 | 6 | 12 | 13 | 8 | 9 | 11 | 7 | 9 |
| 82 |  |  |  |  |  |  |  |  |  |  |  |  |
| 83 | 11 | 8 | 13 | 10 | 8 | 2 | 5 | 5 | 8 | 10 | 4 | 6 |
| 84 |  |  |  |  |  |  |  |  |  |  |  |  |
| 85 | 7 | 10 | 11 | 11 | 3 | 5 | 7 | 9 | 10 | 13 | 9 | 10 |
| 86 | 17 | 18 | 19 | 22 | 19 | 28 | 26 | 19 | 20 | 16 | 19 | 27 |
| 87 |  | 8 | 14 | 8 | 6 | 4 | 6 | 5 | 9 | 1 | 7 | 5 |
| 88 | 16 | 8 | 8 | 5 | 9 | 9 | 14 | 9 | 10 | 10 | 7 | 18 |
| 89 | 34 | 33 | 35 | 38 | 29 | 30 | 31 | 32 | 31 | 31 | 32 | 31 |
| 90 | 20 | 11 | 23 | 16 | 17 | 17 | 15 | 13 | 15 | 13 | 11 | 17 |
| 91 | 23 | 25 | 34 | 29 | 21 | 18 | 23 | 20 | 23 | 27 | 21 | 23 |
| 92 |  |  |  |  |  |  |  |  |  |  |  |  |
| 93 | 6 | 7 | 11 | 10 | 12 | 11 | 2 | 4 | 11 | 10 | 6 | 11 |
| 94 | 3 | 4 | 3 | 5 | 4 | 2 | 3 | 2 | 1 | 4 | 5 | 3 |
| 95 | 8 | 7 | 6 | 6 | 9 | 6 | 2 | 8 | 6 | 9 | 4 | 5 |
| 96 | 8 | 13 | 13 | 14 | 9 | 4 | 9 | 11 | 12 | 12 | 16 | 12 |
| 97 | 3 | 4 | 8 | 1 | 3 | 4 | 7 | 5 | 1 | 6 | 4 | 4 |
| 98 | 9 | 6 | 5 | 4 | 9 | 6 | 6 | 8 | 8 | 3 | 10 | 3 |
| 99 | 4 | 6 | 6 | 7 | 5 | 6 | 5 | 2 | 4 | 9 | 4 | 4 |
| 100 | 5 | 6 | 3 | 10 | 8 | 2 | 5 | 3 | 6 | 5 | 6 | 8 |
| 101 | 12 | 11 | 14 | 11 | 11 | 11 | 10 | 8 | 11 | 18 | 10 | 16 |
| 102 |  |  |  |  |  |  |  |  |  |  |  |  |
| 103 | 11 | 11 | 15 | 13 | 12 | 16 | 10 | 11 | 16 | 7 | 10 | 16 |
| 104 | 15 | 13 | 12 | 17 | 15 | 19 | 14 | 15 | 13 | 16 | 12 | 14 |
| 105 | 12 | 9 | 6 | 6 | 9 | 11 | 11 | 7 | 4 | 7 | 8 | 13 |

Table 57. Number of colorectal cancer endoscopic procedure during each month in 2019.

|  | Month | | | | | | | | | | | |
| --- | --- | --- | --- | --- | --- | --- | --- | --- | --- | --- | --- | --- |
| Hospital | 1 | 2 | 3 | 4 | 5 | 6 | 7 | 8 | 9 | 10 | 11 | 12 |
| 1 | 0 | 0 | 1 | 1 | 1 | 0 | 1 | 3 | 4 | 3 | 0 | 0 |
| 2 | 295 | 306 | 336 | 308 | 266 | 299 | 318 | 289 | 252 | 286 | 329 | 283 |
| 3 | 0 | 2 | 2 | 1 | 4 | 1 | 2 | 2 | 1 | 1 | 2 | 2 |
| 4 | 22 | 13 | 20 | 15 | 18 | 13 | 8 | 15 | 12 | 16 | 11 | 12 |
| 5 | 56 | 60 | 53 | 61 | 56 | 51 | 63 | 54 | 60 | 73 | 72 | 61 |
| 6 | 8 | 5 | 1 | 6 | 6 | 8 | 6 | 4 | 10 | 9 | 4 | 4 |
| 7 | 4 | 10 | 6 | 10 | 15 | 8 | 5 | 5 | 5 | 5 | 3 | 8 |
| 8 |  |  |  |  |  |  |  |  |  |  |  |  |
| 9 | 0 | 6 | 3 | 8 | 1 | 4 | 5 | 3 | 1 | 4 | 1 | 8 |
| 10 | 50 | 46 | 51 | 38 | 50 | 52 | 66 | 58 | 55 | 48 | 78 | 57 |
| 11 | 7 | 7 | 7 | 10 | 7 | 9 | 11 | 7 | 9 | 9 | 5 | 8 |
| 12 | 1 | 0 | 0 | 0 | 0 | 0 | 1 | 1 | 0 | 0 | 1 | 1 |
| 13 | 6 | 12 | 7 | 5 | 5 | 8 | 15 | 5 | 4 | 5 | 5 | 8 |
| 14 | 10 | 14 | 11 | 9 | 7 | 9 | 6 | 5 | 8 | 4 | 3 | 8 |
| 15 | 8 | 4 | 3 | 15 | 6 | 9 | 5 | 6 | 8 | 8 | 6 | 11 |
| 16 | 12 | 18 | 18 | 15 | 10 | 22 | 17 | 12 | 19 | 22 | 18 | 21 |
| 17 | 0 | 0 | 0 | 0 | 0 | 0 | 0 | 0 | 0 | 0 | 0 | 0 |
| 18 | 1 | 2 | 0 | 0 | 1 | 2 | 1 | 1 | 2 | 1 | 1 | 3 |
| 19 | 2 | 0 | 1 | 1 | 0 | 1 | 0 | 3 | 1 | 1 | 1 | 1 |
| 20 | 78 | 69 | 79 | 56 | 73 | 53 | 78 | 56 | 54 | 53 | 71 | 77 |
| 21 | 43 | 48 | 62 | 67 | 68 | 67 | 81 | 69 | 68 | 62 | 71 | 74 |
| 22 | 20 | 22 | 16 | 18 | 16 | 19 | 13 | 29 | 18 | 27 | 33 | 19 |
| 23 |  |  |  |  |  |  |  |  |  |  |  |  |
| 24 | 16 | 25 | 15 | 14 | 17 | 12 | 22 | 14 | 19 | 22 | 23 | 20 |
| 25 | 44 | 52 | 50 | 46 | 44 | 50 | 52 | 75 | 68 | 91 | 84 | 89 |
| 26 | 5 | 2 | 4 | 4 | 1 | 0 | 1 | 4 | 2 | 3 | 3 | 1 |
| 27 | 0 | 0 | 0 | 0 | 0 | 0 | 0 | 0 | 0 | 0 | 0 | 0 |
| 28 | 1 | 3 | 5 | 2 | 2 | 1 | 5 | 3 | 6 | 6 | 6 | 5 |
| 29 | 4 | 7 | 15 | 14 | 16 | 12 | 15 | 7 | 18 | 16 | 12 | 14 |
| 30 | 1 | 2 | 2 | 1 | 3 | 4 | 2 | 2 | 3 | 4 | 5 | 1 |
| 31 | 7 | 9 | 8 | 14 | 2 | 9 | 5 | 6 | 3 | 7 | 8 | 4 |
| 32 | 1 | 1 | 3 | 7 | 5 | 4 | 5 | 2 | 4 | 3 | 4 | 5 |
| 33 | 2 | 4 | 6 | 5 | 3 | 9 | 7 | 2 | 2 | 4 | 10 | 5 |
| 34 | 6 | 9 | 13 | 7 | 5 | 9 | 4 | 9 | 7 | 7 | 7 | 3 |
| 35 | 12 | 15 | 13 | 12 | 13 | 16 | 10 | 19 | 5 | 12 | 4 | 10 |
| 36 | 17 | 15 | 20 | 14 | 14 | 19 | 20 | 17 | 20 | 21 | 12 | 20 |
| 37 | 9 | 6 | 3 | 7 | 3 | 8 | 9 | 8 | 14 | 7 | 7 | 15 |
| 38 | 0 | 1 | 1 | 0 | 1 | 0 | 0 | 0 | 3 | 4 | 0 | 1 |
| 39 |  | 3 | 1 | 2 | 4 | 2 | 4 | 1 | 1 | 5 | 2 | 1 |
| 40 | 4 | 4 | 5 | 8 | 3 | 7 | 10 | 11 | 6 | 6 | 4 | 6 |
| 41 | 8 | 7 | 5 | 9 | 9 | 13 | 14 | 6 | 10 | 7 | 6 | 7 |
| 42 | 6 | 4 | 1 | 4 | 3 | 4 | 3 | 6 | 3 | 2 | 3 | 2 |
| 43 | 14 | 13 | 16 | 14 | 8 | 17 | 19 | 19 | 22 | 24 | 17 | 20 |
| 44 | 13 | 9 | 18 | 11 | 8 | 18 | 10 | 11 | 9 | 4 | 9 | 13 |
| 45 | 1 | 7 | 4 | 1 | 6 | 5 | 7 | 5 | 4 | 4 | 2 | 6 |
| 46 | 7 | 7 | 4 | 5 | 6 | 11 | 6 | 5 | 9 | 3 | 3 | 5 |
| 47 | 71 | 81 | 62 | 80 | 86 | 70 | 101 | 73 | 66 | 87 | 103 | 67 |
| 48 | 54 | 60 | 60 | 62 | 48 | 67 | 51 | 54 | 54 | 62 | 53 | 58 |
| 49 | 30 | 27 | 43 | 44 | 55 | 59 | 66 | 72 | 66 | 65 | 51 | 64 |
| 50 | 1 | 1 | 3 | 1 | 1 | 0 | 2 | 0 | 2 | 2 | 1 | 1 |
| 51 | 94 | 83 | 87 | 85 | 70 | 92 | 111 | 88 | 117 | 140 | 136 | 128 |
| 52 | 10 | 6 | 7 | 11 | 4 | 5 | 12 | 6 | 8 | 10 | 1 | 9 |
| 53 | 21 | 15 | 29 | 23 | 14 | 12 | 20 | 19 | 17 | 19 | 14 | 27 |
| 54 | 32 | 26 | 26 | 24 | 26 | 24 | 13 | 27 | 30 | 42 | 30 | 43 |
| 55 | 8 | 7 | 4 | 3 | 7 | 10 | 5 | 6 | 3 | 9 | 7 | 5 |
| 56 | 13 | 19 | 16 | 20 | 22 | 21 | 18 | 20 | 12 | 17 | 14 | 13 |
| 57 | 51 | 46 | 46 | 57 | 49 | 47 | 60 | 58 | 48 | 53 | 45 | 53 |
| 58 | 36 | 39 | 44 | 43 | 42 | 46 | 45 | 42 | 51 | 50 | 61 | 58 |
| 59 | 3 | 4 | 5 | 4 | 1 | 4 | 4 | 1 | 3 | 1 | 0 | 1 |
| 60 | 7 | 7 | 4 | 4 | 5 | 4 | 7 | 7 | 10 | 8 | 7 | 16 |
| 61 | 1 | 4 | 3 | 4 | 3 | 2 | 4 | 1 | 2 | 2 | 5 | 2 |
| 62 |  |  |  |  |  |  |  |  |  |  |  |  |
| 63 |  |  |  | 1 | 1 | 1 |  | 1 | 2 | 1 |  |  |
| 64 | 10 | 10 | 7 | 10 | 4 | 8 | 12 | 8 | 10 | 4 | 8 | 8 |
| 65 | 15 | 19 | 11 | 5 | 11 | 11 | 12 | 22 | 12 | 14 | 10 | 18 |
| 66 | 14 | 15 | 22 | 17 | 14 | 10 | 6 | 15 | 13 | 11 | 13 | 8 |
| 67 | 38 | 34 | 32 | 34 | 35 | 41 | 38 | 39 | 35 | 36 | 39 | 32 |
| 68 | 10 | 11 | 12 | 13 | 11 | 14 | 16 | 11 | 7 | 11 | 11 | 13 |
| 68 | 122 | 140 | 131 | 133 | 96 | 120 | 138 | 131 | 123 | 144 | 121 | 133 |
| 70 |  |  |  | 2 | 1 | 3 |  | 1 | 3 |  | 2 |  |
| 71 | 10 | 7 | 4 | 4 | 5 | 5 | 9 | 7 | 3 | 14 | 8 | 14 |
| 72 | 11 | 8 | 3 | 7 | 6 | 7 | 9 | 4 | 8 | 5 | 11 | 9 |
| 73 | 15 | 15 | 12 | 9 | 15 | 11 | 19 | 12 | 13 | 8 | 11 | 8 |
| 74 | 7 | 8 | 9 | 6 | 10 | 8 | 13 | 11 | 6 | 12 | 4 | 6 |
| 75 | 31 | 16 | 29 | 20 | 28 | 24 | 25 | 19 | 16 | 19 | 23 | 19 |
| 76 | 5 | 4 | 6 | 8 | 1 | 1 | 7 | 4 | 6 | 3 | 7 | 6 |
| 77 | 1 |  | 1 | 1 | 1 |  |  | 1 | 3 |  | 1 | 1 |
| 78 |  |  |  |  |  |  |  |  | 3 | 2 | 2 | 2 |
| 79 | 2 | 3 | 1 | 3 | 2 | 1 | 1 | 0 | 2 | 1 | 1 | 0 |
| 80 | 1 | 5 | 3 | 0 | 2 | 1 | 3 | 1 | 3 | 3 | 1 | 0 |
| 81 | 12 | 8 | 13 | 14 | 9 | 12 | 10 | 7 | 14 | 16 | 11 | 11 |
| 82 | 3 | 4 | 1 | 4 | 3 | 8 | 3 | 5 | 1 | 4 | 6 | 1 |
| 83 | 3 | 8 | 4 | 1 | 6 | 5 | 6 | 1 | 2 | 1 | 2 | 2 |
| 84 |  |  |  |  |  |  |  |  |  |  |  |  |
| 85 | 7 | 3 | 5 | 3 | 6 | 4 | 5 | 1 | 3 | 4 | 8 | 4 |
| 86 | 0 | 8 | 4 | 4 | 6 | 3 | 8 | 4 | 5 | 5 | 3 | 4 |
| 87 | 44 | 46 | 42 | 40 | 39 | 40 | 41 | 41 | 47 | 41 | 41 | 55 |
| 88 | 1 | 2 | 0 | 2 | 1 | 1 | 1 | 2 | 0 | 0 | 2 | 1 |
| 89 | 122 | 135 | 135 | 141 | 101 | 134 | 130 | 127 | 118 | 134 | 142 | 153 |
| 90 | 8 | 7 | 5 | 6 | 8 | 6 | 7 | 5 | 8 | 5 | 5 | 10 |
| 91 | 127 | 141 | 120 | 148 | 124 | 142 | 155 | 114 | 135 | 130 | 107 | 133 |
| 92 |  |  |  |  |  |  |  |  |  |  |  |  |
| 93 | 8 | 9 | 3 | 15 | 12 | 7 | 14 | 10 | 12 | 11 | 3 | 9 |
| 94 | 4 | 2 | 1 | 3 | 3 | 1 | 0 | 2 | 2 | 3 | 3 | 0 |
| 95 | 0 | 7 | 4 | 1 | 2 | 2 | 0 | 5 | 2 | 3 | 0 | 2 |
| 96 | 75 | 84 | 86 | 83 | 68 | 71 | 61 | 70 | 64 | 95 | 82 | 70 |
| 97 | 6 | 8 | 10 | 11 | 6 | 9 | 8 | 6 | 2 | 10 | 7 | 10 |
| 98 | 2 | 3 | 3 | 6 | 3 | 8 | 3 | 3 | 2 | 5 | 2 | 4 |
| 99 | 1 | 0 | 0 | 0 | 0 | 0 | 0 | 1 | 0 | 1 | 0 | 1 |
| 100 | 1 | 4 | 4 | 4 | 1 | 2 | 6 | 7 | 4 | 3 | 2 | 5 |
| 101 | 67 | 65 | 79 | 77 | 70 | 74 | 63 | 57 | 78 | 66 | 66 | 63 |
| 102 |  |  |  |  |  |  |  |  |  |  |  |  |
| 103 | 2 | 6 | 3 | 6 | 13 | 6 | 7 | 4 | 7 | 14 | 5 | 13 |
| 104 | 77 | 70 | 54 | 62 | 60 | 73 | 74 | 73 | 53 | 80 | 43 | 80 |
| 105 | 8 | 9 | 2 | 2 | 3 | 4 | 8 | 5 | 4 | 5 | 6 | 6 |

Table 58. Number of colorectal cancer endoscopic procedure during each month in 2020.

|  | Month | | | | | | | | | | | |
| --- | --- | --- | --- | --- | --- | --- | --- | --- | --- | --- | --- | --- |
| Hospital | 1 | 2 | 3 | 4 | 5 | 6 | 7 | 8 | 9 | 10 | 11 | 12 |
| 1 | 0 | 3 | 3 | 4 | 3 | 5 | 6 | 4 | 3 | 6 | 4 | 4 |
| 2 | 260 | 235 | 262 | 133 | 112 | 188 | 276 | 251 | 265 | 316 | 314 | 296 |
| 3 | 2 | 2 | 1 | 2 | 1 | 3 | 2 | 1 | 1 | 3 | 0 | 1 |
| 4 | 19 | 10 | 18 | 9 | 8 | 16 | 11 | 15 | 13 | 21 | 16 | 21 |
| 5 | 47 | 45 | 57 | 33 | 28 | 43 | 54 | 51 | 57 | 55 | 54 | 51 |
| 6 | 9 | 4 | 2 | 6 | 3 | 10 | 6 | 4 | 3 | 4 | 6 | 5 |
| 7 | 7 | 5 | 5 | 6 | 1 | 8 | 5 | 10 | 5 | 6 | 6 | 12 |
| 8 |  |  |  |  |  |  |  |  |  |  |  |  |
| 9 | 4 | 3 | 5 | 2 | 2 | 4 | 3 | 2 | 3 | 3 | 3 | 0 |
| 10 | 50 | 58 | 59 | 38 | 17 | 47 | 53 | 47 | 61 | 63 | 74 | 55 |
| 11 | 4 | 8 | 10 | 8 | 6 | 5 | 7 | 10 | 11 | 12 | 9 | 5 |
| 12 | 1 | 2 | 1 | 2 | 1 | 1 | 1 | 0 | 0 | 1 | 0 | 2 |
| 13 | 5 | 6 | 3 | 5 | 5 | 5 | 1 | 4 | 2 | 6 | 2 | 3 |
| 14 | 7 | 4 | 6 | 2 | 1 | 4 | 7 | 3 | 3 | 3 | 8 | 5 |
| 15 | 3 | 4 | 11 | 4 | 5 | 5 | 6 | 3 | 6 | 10 | 3 | 8 |
| 16 | 19 | 16 | 16 | 16 | 21 | 14 | 18 | 13 | 12 | 16 | 21 | 14 |
| 17 | 0 | 0 | 0 | 1 | 0 | 0 | 0 | 0 | 0 | 0 | 0 | 0 |
| 18 | 0 | 0 | 3 | 2 | 1 | 2 | 3 | 1 | 1 | 1 | 2 | 3 |
| 19 | 0 | 1 | 0 | 0 | 2 | 0 | 3 | 2 | 1 | 1 | 1 | 0 |
| 20 | 49 | 77 | 57 | 51 | 22 | 50 | 55 | 69 | 74 | 64 | 63 | 71 |
| 21 | 82 | 57 | 59 | 55 | 26 | 63 | 67 | 47 | 84 | 76 | 72 | 68 |
| 22 | 18 | 19 | 19 | 14 | 9 | 18 | 22 | 17 | 19 | 20 | 24 | 19 |
| 23 |  |  |  |  |  |  |  |  |  |  |  |  |
| 24 | 13 | 20 | 22 | 15 | 17 | 22 | 22 | 15 | 21 | 22 | 18 | 20 |
| 25 | 74 | 78 | 91 | 76 | 50 | 61 | 94 | 85 | 78 | 93 | 88 | 91 |
| 26 | 0 | 3 | 1 | 0 | 0 | 0 | 1 | 2 | 2 | 0 | 1 | 1 |
| 27 | 0 | 0 | 0 | 0 | 0 | 0 | 0 | 0 | 0 | 0 | 0 | 0 |
| 28 | 6 | 4 | 5 | 9 | 3 | 2 | 8 | 7 | 2 | 2 | 8 | 5 |
| 29 | 10 | 10 | 18 | 19 | 8 | 17 | 21 | 14 | 10 | 25 | 28 | 14 |
| 30 | 2 | 2 | 2 | 2 | 3 | 2 | 4 | 2 | 2 | 3 | 1 | 3 |
| 31 | 6 | 3 | 7 | 7 | 4 | 10 | 2 | 7 | 3 | 11 | 5 | 8 |
| 32 | 2 |  | 1 | 3 | 1 |  | 2 | 3 | 2 | 3 | 1 | 2 |
| 33 | 4 | 9 | 8 | 5 | 5 | 5 | 5 | 1 | 5 | 9 | 3 | 1 |
| 34 | 9 | 10 | 11 | 2 | 2 | 4 | 2 | 6 | 10 | 13 | 4 | 10 |
| 35 | 7 | 10 | 9 | 13 | 5 | 3 | 13 | 7 | 20 | 12 | 9 | 14 |
| 36 | 18 | 13 | 19 | 14 | 15 | 21 | 18 | 18 | 16 | 17 | 14 | 17 |
| 37 | 6 | 10 | 7 | 5 | 4 | 7 | 7 | 13 | 6 | 7 | 12 | 8 |
| 38 | 1 | 2 | 5 | 4 | 3 | 0 | 1 | 0 | 4 | 2 | 0 | 3 |
| 39 | 4 | 2 | 2 | 1 |  | 1 |  | 2 |  | 2 |  |  |
| 40 | 8 | 9 | 7 | 8 | 5 | 10 | 10 | 8 | 3 | 5 | 2 | 1 |
| 41 | 15 | 8 | 8 | 14 | 7 | 10 | 8 | 7 | 7 | 5 | 13 | 10 |
| 42 | 6 | 1 | 9 | 4 | 1 | 2 | 4 | 2 | 2 | 2 | 2 | 5 |
| 43 | 20 | 21 | 30 | 14 | 11 | 16 | 8 | 21 | 16 | 12 | 18 | 15 |
| 44 | 12 | 12 | 6 | 8 | 4 | 10 | 9 | 9 | 16 | 12 | 10 | 3 |
| 45 | 4 | 6 | 5 | 7 | 4 | 1 | 2 | 4 | 0 | 3 | 1 | 2 |
| 46 | 5 | 7 | 8 | 11 | 4 | 5 | 12 | 5 | 4 | 5 | 2 | 7 |
| 47 | 96 | 79 | 87 | 66 | 39 | 44 | 82 | 52 | 71 | 106 | 89 | 82 |
| 48 | 59 | 56 | 56 | 47 | 33 | 33 | 63 | 44 | 53 | 58 | 54 | 55 |
| 49 | 51 | 42 | 44 | 47 | 27 | 34 | 16 | 54 | 37 | 68 | 42 | 47 |
| 50 | 1 | 1 | 1 | 3 | 1 | 0 | 0 | 0 | 2 | 0 | 1 | 0 |
| 51 | 110 | 82 | 106 | 93 | 62 | 88 | 122 | 97 | 120 | 130 | 132 | 140 |
| 52 | 3 | 9 | 8 | 4 | 3 | 7 | 8 | 6 | 9 | 17 | 6 | 15 |
| 53 | 18 | 24 | 56 | 20 | 8 | 8 | 12 | 12 | 8 | 18 | 13 | 25 |
| 54 | 35 | 30 | 36 | 31 | 15 | 31 | 31 | 22 | 17 | 23 | 22 | 34 |
| 55 | 11 | 6 | 5 | 3 | 5 | 10 | 6 | 2 | 5 | 9 | 4 | 8 |
| 56 | 18 | 17 | 20 | 10 | 7 | 13 | 18 | 20 | 13 | 16 | 13 | 21 |
| 57 | 55 | 47 | 60 | 46 | 29 | 27 | 32 | 26 | 39 | 47 | 41 | 39 |
| 58 | 62 | 45 | 60 | 23 | 25 | 48 | 39 | 50 | 42 | 48 | 46 | 51 |
| 59 | 2 | 2 | 4 | 2 | 0 | 2 | 4 | 1 | 4 | 5 | 3 | 2 |
| 60 | 2 | 5 | 6 | 3 | 5 | 4 | 2 | 10 | 6 | 5 | 4 | 6 |
| 61 | 3 | 10 | 0 | 2 | 3 | 6 | 1 | 3 | 0 | 1 | 3 | 7 |
| 62 |  |  |  |  |  |  |  |  |  |  |  |  |
| 63 | 2 | 3 | 1 |  | 1 | 1 |  | 1 |  | 1 | 2 | 2 |
| 64 | 6 | 5 | 8 | 8 | 6 | 6 | 6 | 5 | 6 | 4 | 5 | 8 |
| 65 | 11 | 13 | 12 | 4 | 5 | 8 | 14 | 11 | 7 | 15 | 20 | 10 |
| 66 | 19 | 13 | 19 | 14 | 10 | 13 | 15 | 12 | 9 | 11 | 13 | 12 |
| 67 | 37 | 38 | 28 | 37 | 15 | 20 | 37 | 34 | 38 | 45 | 39 | 24 |
| 68 | 16 | 17 | 18 | 13 | 6 | 7 | 15 | 5 | 12 | 9 | 5 | 12 |
| 68 | 121 | 108 | 110 | 118 | 88 | 109 | 138 | 138 | 123 | 182 | 143 | 133 |
| 70 | 1 | 2 | 3 | 1 |  | 3 | 1 | 2 |  |  |  |  |
| 71 | 14 | 8 | 21 | 10 | 7 | 5 | 4 | 10 | 5 | 9 | 2 | 6 |
| 72 | 9 | 9 | 5 | 2 | 1 | 0 | 8 | 5 | 7 | 9 | 10 | 17 |
| 73 | 12 | 11 | 11 | 8 | 8 | 9 | 10 | 16 | 16 | 21 | 13 | 11 |
| 74 | 12 | 11 | 14 | 13 | 4 | 11 | 14 | 7 | 12 | 13 | 6 | 8 |
| 75 | 20 | 19 | 21 | 8 | 1 | 5 | 8 | 21 | 6 | 1 | 0 | 0 |
| 76 | 4 | 5 | 4 | 2 | 1 | 1 | 2 | 6 | 4 | 4 | 1 | 4 |
| 77 | 2 | 1 |  |  | 4 |  | 3 | 2 | 1 |  | 2 | 1 |
| 78 |  |  | 1 | 2 |  | 1 |  |  |  | 1 | 1 |  |
| 79 | 1 | 2 | 6 | 3 | 0 | 2 | 1 | 0 | 2 | 3 | 1 | 3 |
| 80 | 3 | 5 | 3 | 1 | 0 | 3 | 1 | 0 | 0 | 4 | 0 | 3 |
| 81 | 13 | 15 | 24 | 7 | 3 | 15 | 14 | 11 | 14 | 13 | 9 | 8 |
| 82 | 1 | 3 | 3 | 1 | 2 | 0 | 3 | 2 | 1 | 2 | 3 | 2 |
| 83 | 7 | 8 | 4 | 5 | 4 | 5 | 5 | 1 | 5 | 5 | 7 | 2 |
| 84 |  |  |  |  |  |  |  |  |  |  |  |  |
| 85 | 5 | 6 | 4 | 2 | 4 | 0 | 3 | 2 | 4 | 4 | 5 | 5 |
| 86 | 6 | 4 | 5 | 2 | 6 | 3 | 6 | 9 | 5 | 6 | 6 | 6 |
| 87 | 46 | 37 | 50 | 11 | 6 | 24 | 41 | 38 | 18 | 9 | 26 | 55 |
| 88 | 1 | 0 | 0 | 1 | 1 | 4 | 4 | 0 | 0 | 1 | 0 | 1 |
| 89 | 146 | 121 | 147 | 87 | 71 | 137 | 148 | 128 | 133 | 143 | 140 | 135 |
| 90 | 12 | 11 | 12 | 13 | 9 | 11 | 12 | 16 | 8 | 10 | 10 | 10 |
| 91 | 128 | 124 | 124 | 106 | 64 | 140 | 136 | 129 | 147 | 177 | 137 | 116 |
| 92 |  |  |  |  |  |  |  |  |  |  |  |  |
| 93 | 10 | 9 | 17 | 11 | 6 | 12 | 15 | 10 | 5 | 8 | 9 | 8 |
| 94 | 4 | 1 | 3 | 1 | 2 | 2 | 0 | 2 | 0 | 4 | 4 | 2 |
| 95 | 1 | 2 | 3 | 1 | 1 | 0 | 1 | 0 | 1 | 4 | 1 | 1 |
| 96 | 72 | 85 | 96 | 43 | 19 | 85 | 70 | 64 | 72 | 96 | 78 | 80 |
| 97 | 8 | 5 | 6 | 12 | 9 | 7 | 4 | 5 | 3 | 11 | 4 | 8 |
| 98 | 8 | 3 | 8 | 1 | 4 | 3 | 3 | 2 | 2 | 2 | 3 | 5 |
| 99 | 2 | 0 | 0 | 0 | 1 | 0 | 0 | 0 | 0 | 0 | 1 | 2 |
| 100 | 4 | 1 | 4 | 4 | 2 | 2 | 3 | 3 | 4 | 4 | 5 | 6 |
| 101 | 76 | 68 | 89 | 48 | 48 | 45 | 45 | 42 | 53 | 54 | 49 | 59 |
| 102 |  |  |  |  |  |  |  |  |  |  |  |  |
| 103 | 6 | 10 | 12 | 7 | 6 | 5 | 6 | 5 | 6 | 5 | 2 | 8 |
| 104 | 64 | 59 | 62 | 66 | 24 | 58 | 48 | 61 | 60 | 72 | 55 | 76 |
| 105 | 5 | 3 | 7 | 3 | 3 | 5 | 2 | 2 | 3 | 3 | 7 | 6 |

Table 59. Number of lung cancer surgery during each month in 2019.

|  | Month | | | | | | | | | | | |
| --- | --- | --- | --- | --- | --- | --- | --- | --- | --- | --- | --- | --- |
| Hospital | 1 | 2 | 3 | 4 | 5 | 6 | 7 | 8 | 9 | 10 | 11 | 12 |
| 1 | 4 | 2 | 6 | 4 | 3 | 8 | 5 | 4 | 6 | 8 | 13 | 10 |
| 2 | 49 | 48 | 54 | 56 | 42 | 48 | 51 | 49 | 44 | 56 | 51 | 45 |
| 3 | 0 | 0 | 0 | 0 | 0 | 0 | 0 | 0 | 0 | 0 | 0 | 0 |
| 4 | 12 | 12 | 8 | 25 | 18 | 18 | 12 | 8 | 15 | 9 | 16 | 15 |
| 5 | 4 | 0 | 4 | 4 | 2 | 6 | 6 | 5 | 3 | 7 | 8 | 6 |
| 6 | 5 | 13 | 10 | 6 | 6 | 7 | 10 | 7 | 9 | 6 | 6 | 5 |
| 7 | 8 | 10 | 10 | 7 | 4 | 6 | 5 | 3 | 7 | 4 | 8 | 5 |
| 8 | 5 | 11 | 11 | 10 | 4 | 5 | 11 | 4 | 6 | 6 | 12 | 11 |
| 9 | 6 | 3 | 1 | 1 | 5 | 5 | 3 | 4 | 6 | 3 | 5 | 6 |
| 10 | 9 | 9 | 8 | 12 | 5 | 5 | 12 | 10 | 8 | 12 | 8 | 7 |
| 11 | 11 | 14 | 19 | 19 | 18 | 15 | 17 | 14 | 19 | 12 | 14 | 13 |
| 12 |  |  |  |  |  |  |  |  |  |  |  |  |
| 13 | 10 | 9 | 7 | 9 | 11 | 6 | 10 | 8 | 10 | 13 | 11 | 12 |
| 14 | 12 | 12 | 5 | 9 | 3 | 4 | 7 | 7 | 8 | 4 | 4 | 5 |
| 15 | 13 | 14 | 15 | 13 | 14 | 14 | 16 | 14 | 10 | 10 | 14 | 11 |
| 16 | 18 | 19 | 20 | 21 | 17 | 16 | 14 | 16 | 14 | 12 | 8 | 19 |
| 17 | 9 | 12 | 16 | 10 | 8 | 6 | 7 | 11 | 9 | 9 | 9 | 15 |
| 18 | 9 | 7 | 6 | 10 | 7 | 7 | 9 | 9 | 9 | 5 | 9 | 4 |
| 19 | 1 | 2 | 6 | 2 | 2 | 3 | 4 | 3 | 5 | 4 | 4 | 1 |
| 20 | 52 | 57 | 62 | 62 | 55 | 66 | 76 | 71 | 53 | 72 | 66 | 65 |
| 21 | 9 | 4 | 9 | 6 | 6 | 8 | 9 | 7 | 12 | 12 | 8 | 7 |
| 22 | 5 | 8 | 5 | 7 | 3 | 6 | 6 | 4 | 7 | 6 | 6 | 10 |
| 23 |  |  |  |  |  |  |  |  |  |  |  |  |
| 24 | 15 | 14 | 13 | 14 | 13 | 19 | 17 | 15 | 16 | 19 | 19 | 16 |
| 25 | 11 | 11 | 5 | 7 | 7 | 6 | 9 | 5 | 6 | 12 | 8 | 8 |
| 26 | 13 | 16 | 10 | 12 | 15 | 12 | 12 | 15 | 7 | 13 | 14 | 22 |
| 27 | 7 | 10 | 3 | 10 | 11 | 8 | 9 | 11 | 10 | 9 | 12 | 15 |
| 28 | 2 | 4 | 9 | 8 | 5 | 6 | 8 | 15 | 8 | 15 | 7 | 8 |
| 29 | 13 | 20 | 22 | 22 | 20 | 21 | 26 | 23 | 12 | 30 | 25 | 21 |
| 30 | 11 | 12 | 13 | 16 | 10 | 16 | 11 | 11 | 9 | 15 | 14 | 15 |
| 31 | 7 | 7 | 8 | 10 | 5 | 8 | 7 | 8 | 2 | 3 | 9 | 6 |
| 32 |  |  |  |  |  |  |  |  |  |  |  |  |
| 33 | 5 | 2 | 3 | 6 | 3 | 5 | 8 | 6 | 7 | 8 | 7 | 6 |
| 34 |  |  |  |  |  |  |  |  |  |  |  |  |
| 35 | 5 | 5 | 5 | 7 | 5 | 4 | 6 | 10 | 11 | 8 | 5 | 6 |
| 36 | 9 | 11 | 14 | 9 | 4 | 15 | 13 | 12 | 7 | 13 | 10 | 10 |
| 37 | 19 | 22 | 23 | 24 | 16 | 21 | 14 | 23 | 20 | 26 | 23 | 23 |
| 38 | 8 | 7 | 6 | 9 | 4 | 7 | 9 | 5 | 7 | 6 | 5 | 8 |
| 39 |  |  |  |  |  |  |  |  |  |  |  |  |
| 40 | 11 | 13 | 11 | 16 | 20 | 11 | 14 | 16 | 10 | 13 | 13 | 15 |
| 41 | 5 | 3 | 6 | 7 | 2 | 7 | 5 | 5 | 2 | 9 | 11 | 4 |
| 42 | 15 | 12 | 10 | 10 | 8 | 7 | 8 | 11 | 10 | 11 | 6 | 9 |
| 43 | 4 | 8 | 9 | 9 | 10 | 12 | 12 | 4 | 11 | 11 | 10 | 7 |
| 44 | 0 | 0 | 2 | 2 | 2 | 3 | 3 | 2 | 1 | 0 | 3 | 1 |
| 45 | 8 | 9 | 5 | 7 | 3 | 8 | 2 | 2 | 9 | 5 | 5 | 9 |
| 46 | 10 | 10 | 11 | 7 | 4 | 9 | 12 | 12 | 11 | 10 | 9 | 6 |
| 47 |  |  |  |  |  |  |  |  |  |  |  |  |
| 48 | 10 | 10 | 11 | 15 | 7 | 15 | 12 | 13 | 14 | 15 | 9 | 14 |
| 49 | 4 | 5 | 4 | 8 | 4 | 3 | 5 | 4 | 5 | 3 | 8 | 6 |
| 50 | 0 | 0 | 0 | 0 | 0 | 0 | 0 | 0 | 0 | 0 | 0 | 0 |
| 51 | 10 | 7 | 5 | 12 | 8 | 7 | 10 | 9 | 15 | 11 | 15 | 15 |
| 52 | 12 | 10 | 7 | 14 | 3 | 10 | 11 | 12 | 16 | 19 | 9 | 14 |
| 53 | 12 | 10 | 11 | 11 | 9 | 8 | 10 | 8 | 8 | 8 | 9 | 13 |
| 54 | 23 | 27 | 29 | 24 | 24 | 24 | 25 | 25 | 23 | 28 | 26 | 30 |
| 55 | 2 | 5 | 4 | 8 | 4 | 10 | 6 | 7 | 6 | 6 | 5 | 7 |
| 56 | 0 | 13 | 34 | 36 | 32 | 33 | 39 | 36 | 34 | 35 | 38 | 39 |
| 57 | 17 | 18 | 18 | 13 | 15 | 21 | 15 | 9 | 20 | 20 | 17 | 16 |
| 58 | 9 | 8 | 8 | 9 | 10 | 14 | 13 | 7 | 5 | 13 | 13 | 9 |
| 59 | 9 | 10 | 12 | 16 | 4 | 8 | 16 | 14 | 15 | 16 | 3 | 9 |
| 60 | 8 | 10 | 11 | 8 | 8 | 12 | 8 | 10 | 5 | 9 | 12 | 13 |
| 61 | 9 | 6 | 7 | 7 | 7 | 4 | 3 | 10 | 8 | 5 | 10 | 12 |
| 62 | 24 | 24 | 26 | 27 | 16 | 24 | 25 | 22 | 20 | 25 | 29 | 26 |
| 63 |  |  |  |  |  |  |  |  |  |  |  |  |
| 64 | 15 | 17 | 16 | 15 | 17 | 18 | 18 | 14 | 17 | 10 | 16 | 14 |
| 65 | 7 | 17 | 11 | 6 | 11 | 12 | 17 | 11 | 10 | 21 | 21 | 12 |
| 66 | 3 | 1 | 2 | 0 | 0 | 1 | 1 | 0 | 3 | 1 | 0 | 4 |
| 67 | 11 | 11 | 6 | 15 | 10 | 6 | 13 | 12 | 11 | 14 | 11 | 10 |
| 68 | 0 | 1 | 0 | 1 | 1 | 1 | 0 | 1 | 1 | 0 | 0 | 1 |
| 68 | 14 | 13 | 19 | 19 | 15 | 19 | 17 | 13 | 13 | 22 | 18 | 18 |
| 70 |  |  |  |  |  |  |  |  |  |  |  |  |
| 71 | 0 | 0 | 0 | 0 | 0 | 0 | 0 | 0 | 0 | 0 | 0 | 0 |
| 72 | 18 | 7 | 12 | 6 | 13 | 11 | 14 | 15 | 10 | 11 | 12 | 19 |
| 73 | 3 | 6 |  | 9 | 6 | 6 | 8 | 6 | 6 | 8 | 9 | 8 |
| 74 | 9 | 10 | 16 | 14 | 10 | 13 | 11 | 8 | 16 | 18 | 11 | 16 |
| 75 |  |  |  |  |  |  |  |  |  |  |  |  |
| 76 | 7 | 9 | 10 | 11 | 6 | 10 | 15 | 9 | 5 | 11 | 8 | 6 |
| 77 |  |  |  |  |  |  |  |  |  |  |  |  |
| 78 |  |  |  |  |  |  |  |  |  |  |  |  |
| 79 | 10 | 20 | 19 | 16 | 8 | 23 | 18 | 23 | 17 | 17 | 20 | 16 |
| 80 | 5 | 3 | 2 | 3 | 7 | 4 | 11 | 10 | 10 | 5 | 8 | 7 |
| 81 | 4 | 4 | 4 | 4 | 5 | 5 | 5 | 11 | 3 | 8 | 8 | 8 |
| 82 | 9 | 10 | 13 | 11 | 5 | 9 | 6 | 6 | 6 | 8 | 9 | 8 |
| 83 | 4 | 7 | 3 | 6 | 7 | 3 | 4 | 1 | 2 | 5 | 6 | 5 |
| 84 |  |  |  |  |  |  |  |  |  |  |  |  |
| 85 | 7 | 7 | 5 | 7 | 7 | 9 | 9 | 7 | 10 | 11 | 11 | 11 |
| 86 | 10 | 8 | 8 | 13 | 13 | 11 | 13 | 15 | 17 | 18 | 17 | 13 |
| 87 | 3 | 4 | 3 | 2 | 2 | 2 | 3 | 4 | 2 | 5 | 4 | 3 |
| 88 | 0 | 1 | 2 | 3 | 2 | 0 | 6 | 8 | 4 | 5 | 5 | 7 |
| 89 | 46 | 43 | 40 | 34 | 32 | 48 | 41 | 47 | 43 | 45 | 41 | 45 |
| 90 | 13 | 15 | 13 | 10 | 9 | 9 | 8 | 11 | 14 | 9 | 10 | 11 |
| 91 | 10 | 12 | 13 | 14 | 9 | 16 | 11 | 14 | 12 | 11 | 7 | 8 |
| 92 |  |  |  |  |  |  |  |  |  |  |  |  |
| 93 | 8 | 5 | 4 | 4 | 3 | 7 | 9 | 3 | 3 | 4 | 3 | 4 |
| 94 | 2 | 2 | 1 | 4 | 1 | 2 | 1 | 1 | 1 | 2 | 2 | 3 |
| 95 | 0 | 0 | 1 | 0 | 0 | 2 | 0 | 0 | 0 | 0 | 0 | 0 |
| 96 | 4 | 5 | 5 | 3 | 2 | 7 | 6 | 5 | 5 | 3 | 5 | 3 |
| 97 |  |  |  |  |  |  |  |  |  |  |  |  |
| 98 | 4 | 6 | 2 | 2 | 1 | 7 | 5 | 3 | 3 | 5 | 7 | 2 |
| 99 | 4 | 5 | 3 | 0 | 2 | 2 | 1 | 1 | 4 | 2 | 3 | 3 |
| 100 | 5 | 10 | 10 | 11 | 9 | 13 | 12 | 8 | 5 | 7 | 12 | 9 |
| 101 | 14 | 16 | 14 | 7 | 7 | 11 | 14 | 14 | 18 | 15 | 13 | 13 |
| 102 |  |  |  |  |  |  |  |  |  |  |  |  |
| 103 | 4 | 4 | 6 | 3 | 4 | 8 | 4 | 7 | 4 | 5 | 8 | 6 |
| 104 | 8 | 6 | 4 | 5 | 7 | 7 | 7 | 10 | 7 | 7 | 6 | 7 |
| 105 | 7 | 4 | 5 | 5 | 5 | 5 | 8 | 4 | 4 | 5 | 6 | 4 |

Table 60. Number of lung cancer surgery during each month in 2020.

|  | Month | | | | | | | | | | | |
| --- | --- | --- | --- | --- | --- | --- | --- | --- | --- | --- | --- | --- |
| Hospital | 1 | 2 | 3 | 4 | 5 | 6 | 7 | 8 | 9 | 10 | 11 | 12 |
| 1 | 9 | 7 | 10 | 7 | 5 | 7 | 8 | 5 | 6 | 5 | 2 | 4 |
| 2 | 37 | 33 | 42 | 33 | 45 | 28 | 42 | 32 | 40 | 47 | 46 | 49 |
| 3 | 0 | 0 | 0 | 0 | 0 | 0 | 0 | 0 | 0 | 0 | 0 | 0 |
| 4 | 18 | 15 | 6 | 10 | 9 | 17 | 17 | 16 | 17 | 17 | 11 | 15 |
| 5 | 5 | 2 | 6 | 4 | 5 | 6 | 7 | 2 | 5 | 3 | 4 | 2 |
| 6 | 11 | 8 | 4 | 6 | 4 | 6 | 12 | 6 | 7 | 5 | 5 | 6 |
| 7 | 9 | 3 | 6 | 6 | 4 | 3 | 6 | 5 | 7 | 5 | 7 | 6 |
| 8 | 9 | 9 | 12 | 10 | 7 | 14 | 13 | 7 | 13 | 11 | 7 | 12 |
| 9 | 6 | 7 | 7 | 8 | 1 | 3 | 3 | 2 | 2 | 2 | 3 | 4 |
| 10 | 5 | 11 | 10 | 8 | 8 | 8 | 5 | 7 | 12 | 12 | 11 | 6 |
| 11 | 10 | 8 | 16 | 16 | 16 | 13 | 10 | 15 | 16 | 9 | 11 | 15 |
| 12 |  |  |  |  |  |  |  |  |  |  |  |  |
| 13 | 8 | 8 | 9 | 5 | 6 | 9 | 11 | 11 | 10 | 10 | 12 | 10 |
| 14 | 8 | 6 | 7 | 6 | 6 | 7 | 6 | 8 | 8 | 13 | 7 | 8 |
| 15 | 13 | 8 | 12 | 13 | 13 | 7 | 11 | 10 | 7 | 13 | 10 | 11 |
| 16 | 14 | 14 | 6 | 14 | 8 | 13 | 16 | 9 | 12 | 11 | 15 | 15 |
| 17 | 8 | 9 | 3 | 9 | 0 | 2 | 6 | 4 | 8 | 8 | 9 | 9 |
| 18 | 5 | 5 | 3 | 9 | 5 | 6 | 5 | 4 | 7 | 6 | 7 | 9 |
| 19 | 4 | 4 | 4 | 4 | 4 | 3 | 2 | 1 | 5 | 5 | 5 | 4 |
| 20 | 59 | 57 | 67 | 58 | 52 | 66 | 69 | 63 | 69 | 75 | 63 | 71 |
| 21 | 6 | 9 | 8 | 5 | 7 | 7 | 9 | 4 | 11 | 6 | 9 | 4 |
| 22 | 7 | 7 | 5 | 9 | 8 | 8 | 5 | 5 | 3 | 2 | 5 | 5 |
| 23 |  |  |  |  |  |  |  |  |  |  |  |  |
| 24 | 16 | 16 | 23 | 13 | 12 | 13 | 13 | 10 | 10 | 13 | 13 | 17 |
| 25 | 7 | 12 | 2 | 3 | 5 | 3 | 8 | 2 | 6 | 9 | 6 | 7 |
| 26 | 9 | 14 | 13 | 12 | 10 | 13 | 9 | 12 | 5 | 8 | 9 | 16 |
| 27 | 9 | 10 | 8 | 1 | 9 | 13 | 7 | 4 | 11 | 16 | 13 | 15 |
| 28 | 14 | 4 | 7 | 9 | 5 | 7 | 8 | 9 | 7 | 10 | 12 | 12 |
| 29 | 21 | 20 | 20 | 19 | 10 | 18 | 16 | 15 | 10 | 8 | 18 | 12 |
| 30 | 11 | 9 | 10 | 16 | 14 | 12 | 15 | 18 | 8 | 19 | 9 | 19 |
| 31 | 5 | 11 | 7 | 8 | 6 | 13 | 4 | 8 | 1 | 7 | 9 | 3 |
| 32 |  |  |  |  |  |  |  |  |  |  |  |  |
| 33 | 7 | 5 | 4 | 4 | 4 | 3 | 6 | 4 | 3 | 8 | 6 | 9 |
| 34 |  |  |  |  |  |  |  |  |  |  |  |  |
| 35 | 6 | 4 | 8 | 8 | 9 | 5 | 11 | 11 | 13 | 7 | 6 | 1 |
| 36 | 10 | 13 | 9 | 13 | 10 | 13 | 11 | 9 | 10 | 11 | 12 | 11 |
| 37 | 19 | 20 | 26 | 26 | 17 | 24 | 21 | 20 | 19 | 25 | 20 | 16 |
| 38 | 9 | 4 | 9 | 6 | 3 | 8 | 4 | 6 | 8 | 4 | 8 | 5 |
| 39 |  |  |  |  |  |  |  |  |  |  |  |  |
| 40 | 17 | 11 | 11 | 10 | 7 | 7 | 7 | 6 | 6 | 17 | 10 | 10 |
| 41 | 7 | 3 | 8 | 4 | 2 | 5 | 10 | 6 | 6 | 8 | 8 | 9 |
| 42 | 11 | 8 | 7 | 6 | 7 | 6 | 8 | 7 | 9 | 10 | 7 | 8 |
| 43 | 10 | 8 | 8 | 13 | 7 | 11 | 8 | 9 | 5 | 7 | 9 | 6 |
| 44 | 3 | 1 | 1 | 2 | 1 | 2 | 1 | 3 | 1 | 1 | 3 | 3 |
| 45 | 9 | 4 | 5 | 8 | 7 | 3 | 4 | 2 | 10 | 7 | 6 | 2 |
| 46 | 9 | 7 | 12 | 7 | 12 | 10 | 9 | 8 | 9 | 9 | 7 | 10 |
| 47 |  |  |  |  |  |  |  |  |  |  |  |  |
| 48 | 11 | 13 | 13 | 16 | 9 | 11 | 8 | 4 | 11 | 10 | 8 | 16 |
| 49 | 7 | 8 | 5 | 4 | 5 | 6 | 2 | 8 | 5 | 6 | 4 | 3 |
| 50 | 0 | 0 | 0 | 0 | 0 | 0 | 0 | 0 | 0 | 0 | 0 | 0 |
| 51 | 10 | 4 | 8 | 4 | 6 | 9 | 10 | 9 | 5 | 10 | 11 | 11 |
| 52 | 12 | 8 | 13 | 9 | 8 | 12 | 12 | 5 | 11 | 10 | 20 | 6 |
| 53 | 14 | 12 | 12 | 7 | 4 | 7 | 4 | 7 | 13 | 8 | 8 | 7 |
| 54 | 27 | 23 | 19 | 25 | 25 | 22 | 25 | 20 | 24 | 29 | 21 | 26 |
| 55 | 7 | 5 | 8 | 5 | 3 | 5 | 4 | 3 | 7 | 5 | 5 | 7 |
| 56 | 35 | 40 | 39 | 26 | 29 | 41 | 36 | 26 | 27 | 33 | 31 | 45 |
| 57 | 21 | 21 | 16 | 25 | 14 | 9 | 13 | 14 | 18 | 8 | 11 | 20 |
| 58 | 13 | 14 | 16 | 12 | 9 | 12 | 16 | 11 | 15 | 16 | 15 | 12 |
| 59 | 7 | 10 | 8 | 14 | 3 | 13 | 14 | 9 | 12 | 12 | 5 | 6 |
| 60 | 6 | 8 | 9 | 12 | 14 | 14 | 13 | 12 | 18 | 7 | 8 | 12 |
| 61 | 8 | 8 | 9 | 8 | 9 | 4 | 14 | 8 | 15 | 11 | 13 | 12 |
| 62 | 25 | 21 | 26 | 26 | 20 | 23 | 22 | 26 | 27 | 41 | 30 | 28 |
| 63 |  |  |  |  |  |  |  |  |  |  |  |  |
| 64 | 16 | 13 | 16 | 22 | 16 | 18 | 14 | 17 | 17 | 19 | 19 | 15 |
| 65 | 16 | 16 | 8 | 9 | 7 | 13 | 4 | 13 | 16 | 10 | 12 | 10 |
| 66 | 1 | 1 | 1 | 1 | 0 | 1 | 1 | 0 | 0 | 1 | 2 | 2 |
| 67 | 7 | 6 | 5 | 10 | 4 | 3 | 7 | 7 | 1 | 7 | 4 | 4 |
| 68 | 1 | 0 | 0 | 0 | 0 | 0 | 0 | 0 | 0 | 0 | 0 | 1 |
| 68 | 16 | 12 | 14 | 18 | 12 | 16 | 10 | 10 | 14 | 14 | 13 | 16 |
| 70 |  |  |  |  |  |  |  |  |  |  |  |  |
| 71 | 0 | 0 | 0 | 0 | 0 | 0 | 1 | 1 | 1 | 2 | 2 | 0 |
| 72 | 10 | 13 | 7 | 7 | 13 | 16 | 17 | 13 | 11 | 17 | 13 | 14 |
| 73 | 11 | 5 | 4 | 6 | 1 | 3 | 3 | 4 | 2 | 3 | 5 | 6 |
| 74 | 13 | 17 | 26 | 15 | 8 | 31 | 20 | 10 | 13 | 13 | 13 | 16 |
| 75 |  |  |  |  |  |  |  |  |  |  |  |  |
| 76 | 9 | 8 | 7 | 10 | 6 | 9 | 12 | 7 | 8 | 6 | 10 | 11 |
| 77 |  |  |  |  |  |  |  |  |  |  |  |  |
| 78 |  |  |  |  |  |  |  |  |  |  |  |  |
| 79 | 13 | 12 | 18 | 16 | 13 | 10 | 16 | 14 | 18 | 21 | 18 | 16 |
| 80 | 9 | 4 | 4 | 4 | 4 | 7 | 7 | 8 | 7 | 6 | 9 | 7 |
| 81 | 7 | 5 | 8 | 3 | 2 | 6 | 3 | 3 | 2 | 1 | 4 | 4 |
| 82 | 9 | 7 | 8 | 2 | 9 | 8 | 6 | 9 | 8 | 4 | 6 | 8 |
| 83 | 7 | 5 | 9 | 5 | 2 | 6 | 3 | 4 | 5 | 6 | 4 | 5 |
| 84 |  |  |  |  |  |  |  |  |  |  |  |  |
| 85 | 10 | 7 | 7 | 10 | 9 | 11 | 7 | 4 | 5 | 12 | 9 | 6 |
| 86 | 16 | 8 | 14 | 17 | 7 | 13 | 6 | 11 | 13 | 14 | 10 | 13 |
| 87 | 5 | 3 | 1 | 3 | 6 | 5 | 5 | 4 | 1 |  | 1 | 1 |
| 88 | 4 | 5 | 1 | 5 | 4 | 5 | 4 | 3 | 5 | 3 | 5 | 0 |
| 89 | 43 | 38 | 36 | 50 | 45 | 54 | 52 | 45 | 54 | 50 | 42 | 43 |
| 90 | 11 | 11 | 11 | 6 | 7 | 9 | 10 | 9 | 12 | 12 | 13 | 17 |
| 91 | 13 | 16 | 18 | 9 | 10 | 16 | 12 | 12 | 12 | 16 | 14 | 5 |
| 92 |  |  |  |  |  |  |  |  |  |  |  |  |
| 93 | 2 | 3 | 8 | 8 | 4 | 6 | 4 | 3 | 4 | 3 | 3 | 9 |
| 94 | 1 | 1 | 1 | 0 | 1 | 4 | 0 | 1 | 1 | 2 | 1 | 3 |
| 95 | 1 | 1 | 1 | 0 | 0 | 0 | 0 | 0 | 0 | 1 | 0 | 1 |
| 96 | 4 | 7 | 8 | 9 | 4 | 4 | 3 | 3 | 6 | 4 | 7 | 6 |
| 97 |  |  |  |  |  |  |  |  |  |  |  |  |
| 98 | 5 | 7 | 8 | 1 | 4 | 0 | 1 | 5 | 5 | 4 | 5 | 6 |
| 99 | 4 | 3 | 3 | 5 | 1 | 2 | 4 | 2 | 4 | 4 | 3 | 3 |
| 100 | 7 | 11 | 9 | 14 | 15 | 18 | 11 | 10 | 9 | 11 | 10 | 9 |
| 101 | 16 | 14 | 17 | 8 | 9 | 16 | 9 | 13 | 7 | 13 | 18 | 7 |
| 102 |  |  |  |  |  |  |  |  |  |  |  |  |
| 103 | 9 | 7 | 8 | 9 | 7 | 7 | 6 | 6 | 5 | 6 | 6 | 5 |
| 104 | 5 | 5 | 6 | 5 | 4 | 4 | 6 | 6 | 8 | 6 | 9 | 3 |
| 105 | 6 | 3 | 5 | 6 | 4 | 5 | 3 | 2 | 4 | 6 | 4 | 4 |

Table 61. Number of breast cancer surgery during each month in 2019.

|  | Month | | | | | | | | | | | |
| --- | --- | --- | --- | --- | --- | --- | --- | --- | --- | --- | --- | --- |
| Hospital | 1 | 2 | 3 | 4 | 5 | 6 | 7 | 8 | 9 | 10 | 11 | 12 |
| 1 | 0 | 0 | 0 | 0 | 1 | 1 | 1 | 1 | 0 | 0 | 0 | 2 |
| 2 | 104 | 117 | 112 | 118 | 110 | 119 | 112 | 116 | 106 | 96 | 108 | 109 |
| 3 | 4 | 1 | 1 | 3 | 3 | 5 | 2 | 2 | 4 | 7 | 5 | 2 |
| 4 | 30 | 27 | 25 | 30 | 33 | 31 | 28 | 30 | 29 | 35 | 31 | 31 |
| 5 | 0 | 1 | 1 | 6 | 2 | 1 | 2 | 2 | 3 | 4 | 2 | 6 |
| 6 | 9 | 7 | 7 | 7 | 7 | 14 | 12 | 6 | 10 | 8 | 14 | 11 |
| 7 | 5 | 5 | 5 | 6 | 7 | 7 | 4 | 6 | 7 | 11 | 8 | 8 |
| 8 |  |  |  |  |  |  |  |  |  |  |  |  |
| 9 | 6 | 9 | 5 | 10 | 10 | 9 | 6 | 10 | 9 | 11 | 10 | 11 |
| 10 | 13 | 14 | 14 | 12 | 17 | 19 | 19 | 21 | 23 | 24 | 15 | 16 |
| 11 | 11 | 13 | 16 | 15 | 11 | 14 | 17 | 17 | 14 | 14 | 15 | 24 |
| 12 | 2 | 1 | 4 | 4 | 2 | 2 | 2 | 3 | 4 | 3 | 4 | 5 |
| 13 | 34 | 33 | 27 | 35 | 34 | 31 | 33 | 23 | 29 | 41 | 39 | 29 |
| 14 | 6 | 6 | 6 | 7 | 7 | 4 | 6 | 9 | 5 | 9 | 10 | 6 |
| 15 | 17 | 17 | 8 | 16 | 18 | 22 | 18 | 21 | 19 | 20 | 26 | 19 |
| 16 | 22 | 19 | 24 | 26 | 24 | 24 | 22 | 23 | 26 | 31 | 29 | 27 |
| 17 | 0 | 0 | 1 | 0 | 0 | 0 | 0 | 0 | 0 | 0 | 0 | 0 |
| 18 | 12 | 15 | 15 | 15 | 16 | 13 | 18 | 8 | 16 | 17 | 18 | 18 |
| 19 | 6 | 6 | 5 | 6 | 5 | 4 | 5 | 8 | 5 | 7 | 10 | 5 |
| 20 | 40 | 40 | 43 | 40 | 48 | 43 | 43 | 45 | 40 | 43 | 43 | 37 |
| 21 | 10 | 8 | 12 | 6 | 10 | 11 | 13 | 10 | 7 | 11 | 10 | 11 |
| 22 | 13 | 10 | 13 | 14 | 10 | 16 | 13 | 13 | 14 | 19 | 14 | 17 |
| 23 |  |  |  |  |  |  |  |  |  |  |  |  |
| 24 | 16 | 13 | 13 | 8 | 19 | 13 | 18 | 18 | 18 | 19 | 17 | 14 |
| 25 | 21 | 21 | 11 | 13 | 13 | 26 | 17 | 26 | 24 | 25 | 24 | 23 |
| 26 | 9 | 13 | 10 | 19 | 12 | 13 | 10 | 12 | 12 | 10 | 8 | 16 |
| 27 | 26 | 19 | 36 | 27 | 23 | 27 | 20 | 27 | 22 | 22 | 24 | 22 |
| 28 | 1 | 3 | 5 | 2 | 2 | 7 | 11 | 9 | 3 | 5 | 9 | 2 |
| 29 | 15 | 22 | 20 | 26 | 20 | 21 | 22 | 27 | 29 | 28 | 24 | 33 |
| 30 | 10 | 10 | 7 | 10 | 8 | 10 | 10 | 6 | 9 | 11 | 11 | 7 |
| 31 | 14 | 19 | 17 | 17 | 11 | 11 | 7 | 9 | 10 | 12 | 13 | 13 |
| 32 | 10 | 10 | 14 | 10 | 10 | 13 | 14 | 14 | 10 | 12 | 12 | 11 |
| 33 | 4 | 4 | 4 | 4 | 4 | 5 | 5 | 5 | 3 | 4 | 7 | 5 |
| 34 | 30 | 22 | 26 | 19 | 23 | 20 | 21 | 26 | 23 | 24 | 26 | 34 |
| 35 | 2 | 6 | 5 | 5 | 7 | 9 | 8 | 8 | 6 | 5 | 8 | 7 |
| 36 | 16 | 13 | 14 | 14 | 8 | 16 | 11 | 14 | 13 | 13 | 12 | 21 |
| 37 | 28 | 30 | 28 | 34 | 32 | 34 | 26 | 35 | 33 | 31 | 32 | 32 |
| 38 | 3 | 3 | 10 | 8 | 9 | 5 | 11 | 14 | 19 | 15 | 19 | 23 |
| 39 | 2 | 2 |  | 2 | 3 | 2 | 1 | 1 | 1 | 3 | 1 | 2 |
| 40 | 10 | 9 | 9 | 14 | 15 | 16 | 17 | 12 | 13 | 29 | 17 | 24 |
| 41 | 8 | 9 | 8 | 13 | 9 | 8 | 15 | 10 | 12 | 11 | 15 | 10 |
| 42 | 7 | 9 | 10 | 9 | 4 | 7 | 9 | 7 | 11 | 6 | 8 | 9 |
| 43 | 3 | 9 | 3 | 8 | 6 | 13 | 9 | 12 | 10 | 7 | 12 | 11 |
| 44 | 7 | 6 | 4 | 7 | 4 | 6 | 2 | 3 | 3 | 7 | 4 | 4 |
| 45 | 11 | 11 | 10 | 12 | 8 | 11 | 9 | 15 | 12 | 11 | 13 | 11 |
| 46 | 10 | 17 | 21 | 16 | 14 | 21 | 22 | 22 | 23 | 23 | 24 | 25 |
| 47 |  |  |  |  |  |  |  |  |  |  |  |  |
| 48 | 5 | 11 | 9 | 10 | 9 | 14 | 9 | 11 | 9 | 6 | 9 | 10 |
| 49 | 12 | 11 | 9 | 7 | 6 | 7 | 9 | 11 | 11 | 14 | 10 | 17 |
| 50 | 2 | 0 | 0 | 0 | 1 | 2 | 2 | 1 | 0 | 0 | 0 | 1 |
| 51 | 17 | 15 | 15 | 19 | 15 | 12 | 9 | 6 | 16 | 18 | 11 | 15 |
| 52 | 12 | 12 | 17 | 10 | 15 | 12 | 14 | 11 | 14 | 18 | 15 | 16 |
| 53 | 17 | 11 | 10 | 18 | 12 | 25 | 9 | 25 | 19 | 17 | 14 | 21 |
| 54 | 39 | 34 | 32 | 38 | 41 | 32 | 34 | 34 | 34 | 42 | 34 | 39 |
| 55 |  | 2 | 2 | 5 | 4 | 5 | 3 | 4 | 6 | 7 | 5 | 5 |
| 56 | 1 | 16 | 37 | 39 | 30 | 34 | 38 | 46 | 40 | 46 | 43 | 42 |
| 57 | 39 | 44 | 49 | 38 | 34 | 43 | 43 | 46 | 44 | 42 | 38 | 47 |
| 58 | 6 | 7 | 7 | 11 | 5 | 9 | 8 | 6 | 7 | 14 | 8 | 9 |
| 59 | 3 | 5 | 10 | 5 | 12 | 9 | 5 | 5 | 6 | 5 | 2 | 4 |
| 60 | 0 | 1 | 10 | 12 | 6 | 7 | 9 | 11 | 7 | 16 | 12 | 12 |
| 61 | 13 | 10 | 5 | 9 | 7 | 8 | 9 | 7 | 5 | 11 | 12 | 6 |
| 62 | 21 | 25 | 23 | 16 | 18 | 20 | 16 | 26 | 20 | 19 | 21 | 16 |
| 63 |  |  | 2 | 1 |  | 2 | 2 | 4 | 1 | 6 | 4 | 2 |
| 64 | 19 | 13 | 19 | 18 | 15 | 16 | 17 | 14 | 20 | 16 | 10 | 14 |
| 65 | 6 | 15 | 10 | 12 | 11 | 20 | 21 | 18 | 12 | 13 | 16 | 19 |
| 66 | 1 | 3 | 5 | 3 | 3 | 8 | 8 | 2 | 3 | 8 | 1 | 2 |
| 67 | 11 | 13 | 9 | 13 | 9 | 12 | 11 | 10 | 10 | 12 | 12 | 13 |
| 68 | 15 | 18 | 16 | 17 | 15 | 19 | 19 | 15 | 18 | 23 | 16 | 19 |
| 68 | 15 | 19 | 17 | 14 | 16 | 14 | 15 | 16 | 12 | 8 | 11 | 12 |
| 70 | 2 | 5 | 3 | 4 | 3 | 4 | 3 | 3 | 3 | 3 | 3 | 4 |
| 71 | 16 | 9 | 15 | 12 | 16 | 15 | 15 | 19 | 20 | 19 | 15 | 17 |
| 72 | 16 | 12 | 17 | 13 | 13 | 15 | 10 | 15 | 12 | 10 | 7 | 24 |
| 73 | 24 | 22 | 20 | 23 | 25 | 24 | 23 | 28 | 27 | 25 | 22 | 21 |
| 74 | 27 | 26 | 27 | 33 | 30 | 30 | 23 | 26 | 23 | 29 | 31 | 20 |
| 75 | 1 | 1 | 3 | 1 | 1 | 2 | 1 | 2 | 2 | 1 |  |  |
| 76 | 19 | 25 | 28 | 25 | 24 | 22 | 21 | 28 | 26 | 29 | 30 | 22 |
| 77 |  |  |  |  |  |  |  |  |  | 1 |  |  |
| 78 |  |  | 1 |  |  |  |  |  |  |  |  |  |
| 79 | 2 | 16 | 16 | 18 | 16 | 15 | 13 | 14 | 23 | 17 | 12 | 18 |
| 80 | 12 | 7 | 9 | 11 | 13 | 8 | 19 | 10 | 12 | 11 | 11 | 12 |
| 81 | 7 | 6 | 9 | 7 | 7 | 10 | 7 | 8 | 7 | 5 | 11 | 11 |
| 82 |  |  |  |  |  |  |  |  |  |  |  |  |
| 83 | 5 | 7 | 9 | 9 | 5 | 11 | 8 | 3 | 7 | 10 | 9 | 11 |
| 84 |  |  |  |  |  |  |  |  |  |  |  |  |
| 85 | 26 | 23 | 17 | 25 | 21 | 24 | 22 | 20 | 20 | 21 | 19 | 23 |
| 86 | 14 | 16 | 9 | 14 | 11 | 11 | 14 | 7 | 9 | 13 | 10 | 14 |
| 87 | 3 | 3 | 5 | 2 | 3 | 5 | 4 | 4 | 5 | 5 | 4 | 6 |
| 88 | 13 | 9 | 7 | 15 | 13 | 13 | 10 | 11 | 5 | 9 | 5 | 6 |
| 89 | 19 | 28 | 24 | 31 | 31 | 37 | 36 | 39 | 36 | 44 | 38 | 39 |
| 90 | 42 | 48 | 35 | 38 | 34 | 33 | 39 | 47 | 33 | 40 | 46 | 43 |
| 91 | 11 | 13 | 15 | 18 | 12 | 16 | 10 | 16 | 18 | 14 | 10 | 23 |
| 92 |  |  |  |  |  |  |  |  |  |  |  |  |
| 93 | 14 | 10 | 11 | 10 | 9 | 9 | 4 | 8 | 8 | 11 | 10 | 11 |
| 94 | 2 | 1 | 5 | 1 | 2 | 1 | 3 | 1 | 3 | 5 | 5 | 4 |
| 95 | 0 | 0 | 2 | 2 | 1 | 1 | 1 | 2 | 3 | 2 | 2 | 1 |
| 96 |  |  |  |  |  |  |  |  |  |  |  |  |
| 97 |  |  |  |  |  |  |  |  |  |  |  |  |
| 98 | 10 | 10 | 8 | 11 | 13 | 11 | 10 | 9 | 15 | 14 | 14 | 12 |
| 99 | 4 | 7 | 9 | 6 | 6 | 7 | 5 | 6 | 2 | 9 | 2 | 10 |
| 100 | 0 | 2 | 1 | 4 | 5 | 4 | 4 | 1 | 6 | 4 | 2 | 6 |
| 101 | 15 | 16 | 20 | 19 | 16 | 16 | 15 | 18 | 12 | 17 | 17 | 19 |
| 102 |  |  |  |  |  |  |  |  |  |  |  |  |
| 103 | 12 | 10 | 9 | 7 | 7 | 4 | 10 | 9 | 9 | 7 | 9 | 8 |
| 104 | 13 | 16 | 16 | 18 | 15 | 14 | 17 | 16 | 14 | 13 | 8 | 10 |
| 105 | 13 | 15 | 17 | 12 | 13 | 16 | 14 | 13 | 10 | 13 | 10 | 14 |

Table 62. Number of breast cancer surgery during each month in 2020.

|  | Month | | | | | | | | | | | |
| --- | --- | --- | --- | --- | --- | --- | --- | --- | --- | --- | --- | --- |
| Hospital | 1 | 2 | 3 | 4 | 5 | 6 | 7 | 8 | 9 | 10 | 11 | 12 |
| 1 | 0 | 0 | 2 | 0 | 0 | 0 | 0 | 0 | 1 | 1 | 0 | 0 |
| 2 | 113 | 106 | 122 | 79 | 101 | 118 | 92 | 67 | 91 | 87 | 81 | 90 |
| 3 | 0 | 4 | 1 | 4 | 0 | 2 | 3 | 3 | 1 | 4 | 1 | 0 |
| 4 | 35 | 31 | 36 | 34 | 28 | 32 | 23 | 22 | 24 | 32 | 30 | 25 |
| 5 | 2 | 1 | 4 | 4 | 1 | 2 | 4 | 6 | 4 | 6 | 3 | 4 |
| 6 | 9 | 2 | 8 | 9 | 9 | 10 | 5 | 6 | 4 | 11 | 9 | 8 |
| 7 | 8 | 8 | 5 | 2 | 4 | 6 | 8 | 4 | 5 | 8 | 8 | 5 |
| 8 |  |  |  |  |  |  |  |  |  |  |  |  |
| 9 | 7 | 9 | 6 | 10 | 8 | 11 | 10 | 9 | 9 | 11 | 4 | 11 |
| 10 | 17 | 15 | 7 | 13 | 11 | 10 | 21 | 16 | 21 | 16 | 20 | 18 |
| 11 | 15 | 13 | 10 | 15 | 17 | 16 | 14 | 10 | 15 | 11 | 11 | 14 |
| 12 | 7 | 2 | 4 | 4 | 3 | 2 | 4 | 6 | 5 | 8 | 4 | 5 |
| 13 | 31 | 36 | 34 | 23 | 23 | 25 | 27 | 20 | 18 | 32 | 29 | 26 |
| 14 | 11 | 3 | 6 | 4 | 6 | 5 | 4 | 7 | 11 | 3 | 11 | 4 |
| 15 | 28 | 26 | 21 | 22 | 20 | 19 | 15 | 20 | 22 | 18 | 16 | 18 |
| 16 | 26 | 25 | 23 | 33 | 26 | 30 | 26 | 31 | 27 | 23 | 32 | 29 |
| 17 | 0 | 0 | 0 | 0 | 0 | 0 | 0 | 0 | 0 | 0 | 0 | 0 |
| 18 | 15 | 14 | 20 | 13 | 12 | 9 | 14 | 9 | 17 | 9 | 8 | 13 |
| 19 | 4 | 3 | 6 | 4 | 4 | 6 | 5 | 2 | 4 | 8 | 7 | 7 |
| 20 | 42 | 48 | 52 | 32 | 33 | 41 | 31 | 33 | 37 | 31 | 42 | 39 |
| 21 | 11 | 11 | 14 | 12 | 12 | 13 | 5 | 16 | 10 | 8 | 5 | 6 |
| 22 | 14 | 15 | 15 | 20 | 10 | 12 | 11 | 16 | 7 | 10 | 10 | 14 |
| 23 |  |  |  |  |  |  |  |  |  |  |  |  |
| 24 | 20 | 19 | 15 | 12 | 6 | 12 | 11 | 10 | 15 | 17 | 13 | 9 |
| 25 | 21 | 21 | 19 | 24 | 25 | 25 | 23 | 24 | 23 | 27 | 23 | 23 |
| 26 | 7 | 8 | 8 | 14 | 6 | 10 | 12 | 5 | 8 | 8 | 10 | 15 |
| 27 | 27 | 29 | 20 | 1 | 19 | 23 | 23 | 10 | 18 | 15 | 19 | 9 |
| 28 | 3 | 8 | 4 | 1 | 9 | 3 | 8 | 7 | 7 | 8 | 7 | 6 |
| 29 | 30 | 21 | 33 | 40 | 24 | 27 | 23 | 32 | 29 | 29 | 24 | 33 |
| 30 | 5 | 7 | 8 | 9 | 6 | 11 | 9 | 5 | 11 | 6 | 5 | 11 |
| 31 | 15 | 13 | 21 | 23 | 10 | 15 | 18 | 10 | 14 | 14 | 22 | 15 |
| 32 | 11 | 9 | 14 | 12 | 5 | 9 | 14 | 11 | 9 | 13 | 13 | 12 |
| 33 | 8 | 6 | 3 | 2 | 5 | 4 | 6 | 5 | 3 | 7 | 7 | 1 |
| 34 | 26 | 23 | 27 | 7 | 11 | 16 | 12 | 13 | 17 | 20 | 21 | 25 |
| 35 | 6 | 4 | 3 | 5 | 8 | 5 | 3 | 3 | 3 | 11 | 8 | 6 |
| 36 | 13 | 11 | 14 | 22 | 11 | 17 | 10 | 13 | 9 | 8 | 6 | 10 |
| 37 | 31 | 28 | 27 | 31 | 26 | 31 | 28 | 34 | 28 | 30 | 25 | 28 |
| 38 | 13 | 12 | 13 | 17 | 10 | 10 | 13 | 8 | 13 | 20 | 16 | 19 |
| 39 | 3 | 1 |  |  | 1 |  | 4 | 5 | 4 | 2 | 1 | 1 |
| 40 | 22 | 6 | 15 | 9 | 9 | 9 | 11 | 11 | 13 | 16 | 13 | 12 |
| 41 | 12 | 5 | 15 | 16 | 13 | 8 | 13 | 16 | 10 | 8 | 8 | 15 |
| 42 | 9 | 6 | 8 | 10 | 6 | 9 | 7 | 8 | 7 | 7 | 7 | 7 |
| 43 | 14 | 11 | 9 | 9 | 4 | 10 | 8 | 7 | 8 | 11 | 7 | 14 |
| 44 | 6 | 3 | 8 | 5 | 6 | 5 | 2 | 2 | 2 | 2 | 5 | 4 |
| 45 | 10 | 12 | 9 | 9 | 11 | 11 | 10 | 11 | 12 | 15 | 12 | 11 |
| 46 | 21 | 19 | 20 | 16 | 19 | 20 | 20 | 13 | 15 | 15 | 17 | 22 |
| 47 |  |  |  |  |  |  |  |  |  |  |  |  |
| 48 | 10 | 6 | 9 | 9 | 7 | 5 | 8 | 7 | 11 | 13 | 14 | 9 |
| 49 | 13 | 10 | 15 | 11 | 9 | 13 | 2 | 3 | 5 | 6 | 12 | 10 |
| 50 | 0 | 0 | 0 | 0 | 0 | 0 | 0 | 2 | 5 | 0 | 0 | 1 |
| 51 | 18 | 10 | 15 | 16 | 14 | 6 | 12 | 13 | 13 | 14 | 12 | 13 |
| 52 | 20 | 18 | 15 | 14 | 11 | 21 | 16 | 13 | 15 | 14 | 12 | 18 |
| 53 | 19 | 26 | 15 | 17 | 19 | 7 | 15 | 14 | 9 | 12 | 11 | 8 |
| 54 | 36 | 36 | 40 | 42 | 34 | 38 | 43 | 33 | 36 | 37 | 36 | 41 |
| 55 | 6 | 2 | 5 | 4 | 5 | 5 | 5 | 4 | 5 | 4 | 4 | 5 |
| 56 | 47 | 42 | 48 | 43 | 43 | 34 | 27 | 22 | 34 | 38 | 31 | 34 |
| 57 | 49 | 36 | 50 | 51 | 36 | 35 | 34 | 35 | 41 | 40 | 38 | 37 |
| 58 | 7 | 10 | 5 | 11 | 9 | 7 | 8 | 8 | 12 | 11 | 12 | 9 |
| 59 | 5 | 8 | 4 | 2 | 3 | 8 | 6 | 6 | 7 | 5 | 8 | 3 |
| 60 | 2 | 0 | 7 | 11 | 11 | 12 | 13 | 9 | 8 | 14 | 15 | 16 |
| 61 | 15 | 14 | 6 | 8 | 8 | 8 | 9 | 5 | 6 | 7 | 5 | 9 |
| 62 | 20 | 16 | 17 | 22 | 22 | 17 | 15 | 19 | 19 | 20 | 16 | 18 |
| 63 | 1 |  |  | 4 | 1 | 1 | 1 | 2 | 4 | 4 | 1 | 1 |
| 64 | 19 | 18 | 25 | 28 | 16 | 16 | 10 | 14 | 10 | 20 | 20 | 20 |
| 65 | 11 | 10 | 7 | 14 | 9 | 10 | 9 | 18 | 12 | 11 | 11 | 13 |
| 66 | 2 | 3 | 3 | 1 | 3 | 5 | 4 | 2 | 5 | 6 | 5 | 5 |
| 67 | 13 | 9 | 16 | 11 | 10 | 13 | 6 | 7 | 8 | 8 | 12 | 7 |
| 68 | 20 | 19 | 19 | 20 | 13 | 10 | 19 | 14 | 16 | 16 | 14 | 16 |
| 68 | 14 | 8 | 11 | 12 | 10 | 16 | 16 | 14 | 16 | 13 | 10 | 16 |
| 70 | 3 | 1 | 4 | 5 | 3 |  | 1 | 4 | 4 | 4 | 5 | 5 |
| 71 | 15 | 17 | 13 | 16 | 14 | 11 | 10 | 18 | 14 | 18 | 6 | 14 |
| 72 | 26 | 18 | 12 | 14 | 9 | 13 | 14 | 12 | 18 | 22 | 18 | 14 |
| 73 | 19 | 23 | 25 | 26 | 22 | 21 | 18 | 19 | 18 | 27 | 19 | 24 |
| 74 | 32 | 24 | 30 | 26 | 23 | 23 | 24 | 17 | 24 | 33 | 23 | 16 |
| 75 | 1 | 1 |  |  |  |  | 2 |  |  |  |  |  |
| 76 | 24 | 27 | 19 | 23 | 21 | 19 | 20 | 20 | 24 | 23 | 20 | 25 |
| 77 |  |  | 1 |  |  |  |  |  |  |  |  |  |
| 78 |  |  |  |  |  |  |  |  |  |  |  | 1 |
| 79 | 13 | 13 | 12 | 5 | 5 | 14 | 18 | 14 | 15 | 16 | 17 | 14 |
| 80 | 16 | 6 | 6 | 8 | 7 | 13 | 20 | 12 | 11 | 16 | 17 | 10 |
| 81 | 10 | 9 | 12 | 10 | 10 | 9 | 10 | 3 | 6 | 10 | 7 | 4 |
| 82 |  |  |  |  |  |  |  |  |  |  |  |  |
| 83 | 7 | 7 | 7 | 8 | 6 | 10 | 8 | 8 | 5 | 6 | 6 | 5 |
| 84 |  |  |  |  |  |  |  |  |  |  |  |  |
| 85 | 27 | 23 | 23 | 25 | 20 | 21 | 21 | 21 | 19 | 25 | 19 | 21 |
| 86 | 16 | 10 | 14 | 8 | 11 | 18 | 14 | 12 | 7 | 9 | 15 | 10 |
| 87 | 5 | 3 | 6 | 4 | 4 | 3 | 6 | 5 | 4 | 1 | 4 | 3 |
| 88 | 4 | 13 | 7 | 6 | 2 | 4 | 10 | 9 | 6 | 14 | 4 | 4 |
| 89 | 37 | 36 | 30 | 38 | 40 | 34 | 22 | 28 | 34 | 39 | 42 | 38 |
| 90 | 43 | 41 | 34 | 50 | 37 | 33 | 42 | 24 | 25 | 40 | 33 | 42 |
| 91 | 16 | 16 | 13 | 15 | 10 | 8 | 10 | 12 | 18 | 15 | 14 | 13 |
| 92 |  |  |  |  |  |  |  |  |  |  |  |  |
| 93 | 11 | 3 | 9 | 9 | 8 | 7 | 11 | 9 | 10 | 8 | 11 | 11 |
| 94 | 3 | 4 | 7 | 5 | 2 | 1 | 2 | 0 | 4 | 1 | 4 | 3 |
| 95 | 1 | 1 | 2 | 1 | 1 | 7 | 2 | 1 | 1 | 1 | 1 | 3 |
| 96 |  |  |  |  |  |  |  |  |  |  |  |  |
| 97 |  |  |  |  |  |  |  |  |  |  |  |  |
| 98 | 10 | 15 | 10 | 11 | 11 | 9 | 9 | 9 | 9 | 13 | 9 | 11 |
| 99 | 4 | 8 | 9 | 8 | 1 | 1 | 11 | 6 | 6 | 6 | 12 | 6 |
| 100 | 5 | 5 | 3 | 2 | 1 | 1 | 1 | 5 | 7 | 2 | 11 | 4 |
| 101 | 22 | 16 | 21 | 21 | 17 | 18 | 16 | 16 | 18 | 14 | 13 | 20 |
| 102 |  |  |  |  |  |  |  |  |  |  |  |  |
| 103 | 8 | 5 | 9 | 9 | 6 | 7 | 7 | 6 | 11 | 8 | 9 | 12 |
| 104 | 7 | 9 | 12 | 14 | 13 | 15 | 8 | 14 | 11 | 14 | 14 | 15 |
| 105 | 10 | 11 | 17 | 16 | 14 | 14 | 11 | 11 | 8 | 11 | 13 | 12 |

Table 63. Number of cervical cancer surgery during each month in 2019.

|  | Month | | | | | | | | | | | |
| --- | --- | --- | --- | --- | --- | --- | --- | --- | --- | --- | --- | --- |
| Hospital | 1 | 2 | 3 | 4 | 5 | 6 | 7 | 8 | 9 | 10 | 11 | 12 |
| 1 | 1 | 1 | 1 | 1 | 1 | 0 | 2 | 0 | 1 | 0 | 0 | 0 |
| 2 | 47 | 38 | 47 | 48 | 47 | 46 | 42 | 31 | 36 | 47 | 33 | 37 |
| 3 | 0 | 0 | 0 | 1 | 1 | 0 | 1 | 0 | 0 | 1 | 1 | 2 |
| 4 | 7 | 7 | 13 | 12 | 10 | 11 | 11 | 12 | 10 | 11 | 12 | 8 |
| 5 | 3 | 3 | 8 | 9 | 10 | 12 | 12 | 9 | 7 | 13 | 9 | 7 |
| 6 | 7 | 6 | 5 | 0 | 9 | 3 | 3 | 4 | 5 | 4 | 6 | 2 |
| 7 | 4 | 4 | 6 | 4 | 4 | 3 | 6 | 7 | 0 | 1 | 7 | 2 |
| 8 | 0 | 0 | 0 | 0 | 0 | 0 | 0 | 0 | 0 | 0 | 0 | 0 |
| 9 | 3 | 1 | 2 | 2 | 1 | 0 | 0 | 2 | 0 | 1 | 4 | 2 |
| 10 | 5 | 4 | 7 | 3 | 10 | 6 | 8 | 4 | 4 | 7 | 4 | 3 |
| 11 | 1 | 1 | 1 | 3 | 1 | 0 | 1 | 2 | 3 | 1 | 5 | 2 |
| 12 | 1 | 0 | 0 | 0 | 0 | 0 | 0 | 0 | 0 | 0 | 0 | 1 |
| 13 | 3 | 1 | 4 | 3 | 1 | 0 | 1 | 5 | 1 | 2 | 1 | 1 |
| 14 | 6 | 5 | 10 | 7 | 2 | 5 | 4 | 2 | 2 | 4 | 3 | 7 |
| 15 | 4 | 3 | 2 | 2 | 5 | 4 | 1 | 1 | 1 | 1 | 5 | 3 |
| 16 | 2 | 1 | 6 | 4 | 6 | 7 | 7 | 7 | 3 | 5 | 4 | 5 |
| 17 | 0 | 0 | 0 | 0 | 0 | 0 | 0 | 0 | 0 | 0 | 0 | 0 |
| 18 | 2 | 4 | 2 | 2 | 5 | 5 | 4 | 9 | 4 | 2 | 2 | 6 |
| 19 | 0 | 0 | 0 | 0 | 0 | 0 | 1 | 0 | 0 | 0 | 0 | 0 |
| 20 | 11 | 14 | 19 | 12 | 8 | 11 | 15 | 12 | 14 | 18 | 11 | 10 |
| 21 | 8 | 9 | 12 | 8 | 8 | 8 | 9 | 10 | 7 | 7 | 6 | 5 |
| 22 | 12 | 12 | 13 | 15 | 20 | 16 | 12 | 11 | 11 | 17 | 14 | 10 |
| 23 | 0 | 0 | 0 | 0 | 0 | 0 | 0 | 0 | 0 | 0 | 0 | 0 |
| 24 | 3 | 1 | 2 | 1 | 4 | 5 | 3 | 4 | 0 | 2 | 1 | 1 |
| 25 | 5 | 1 | 2 | 3 | 3 | 2 | 4 | 2 | 1 | 1 | 5 | 3 |
| 26 | 10 | 8 | 8 | 5 | 3 | 2 | 9 | 9 | 7 | 6 | 5 | 2 |
| 27 | 0 | 1 | 2 | 2 | 2 | 1 | 0 | 5 | 2 | 0 | 1 | 2 |
| 28 | 1 | 0 | 2 | 2 | 1 | 0 | 3 | 0 | 2 | 3 | 1 | 2 |
| 29 | 3 | 4 | 4 | 0 | 3 | 3 | 3 | 4 | 3 | 0 | 2 | 3 |
| 30 | 4 | 6 | 4 | 6 | 2 | 4 | 5 | 2 | 3 | 4 | 4 | 3 |
| 31 | 0 | 1 | 3 | 3 | 4 | 3 | 2 | 1 | 3 | 1 | 2 | 3 |
| 32 | 0 | 1 | 0 | 0 | 0 | 0 | 0 | 0 | 0 | 3 | 0 | 1 |
| 33 | 3 | 4 | 2 | 1 | 3 | 1 | 1 | 2 | 0 | 4 | 6 | 4 |
| 34 | 0 | 0 | 0 | 0 | 0 | 0 | 0 | 0 | 0 | 0 | 0 | 0 |
| 35 | 8 | 5 | 5 | 6 | 3 | 8 | 7 | 10 | 3 | 8 | 12 | 5 |
| 36 | 3 | 6 | 3 | 5 | 3 | 3 | 4 | 10 | 3 | 5 | 4 | 3 |
| 37 | 14 | 17 | 21 | 19 | 10 | 18 | 15 | 14 | 13 | 17 | 12 | 20 |
| 38 | 0 | 0 | 3 | 1 | 1 | 0 | 0 | 0 | 2 | 4 | 0 | 4 |
| 39 | 0 | 0 | 0 | 0 | 0 | 0 | 0 | 0 | 0 | 0 | 0 | 0 |
| 40 | 17 | 6 | 7 | 7 | 3 | 13 | 10 | 7 | 7 | 11 | 13 | 11 |
| 41 | 3 | 0 | 2 | 3 | 1 | 3 | 4 | 3 | 1 | 1 | 1 | 1 |
| 42 | 17 | 10 | 10 | 12 | 16 | 9 | 5 | 3 | 6 | 7 | 9 | 12 |
| 43 | 0 | 2 | 4 | 3 | 4 | 3 | 5 | 2 | 4 | 2 | 4 | 5 |
| 44 | 2 | 3 | 5 | 0 | 1 | 1 | 3 | 1 | 6 | 1 | 2 | 5 |
| 45 | 6 | 6 | 1 | 4 | 2 | 4 | 3 | 3 | 2 | 2 | 2 | 1 |
| 46 | 2 | 1 | 2 | 2 | 0 | 3 | 2 | 0 | 4 | 3 | 3 | 4 |
| 47 | 3 | 5 | 4 | 3 | 5 | 1 | 5 | 3 | 5 | 8 | 5 | 7 |
| 48 | 5 | 12 | 6 | 12 | 6 | 5 | 7 | 7 | 9 | 6 | 10 | 6 |
| 49 | 2 | 3 | 3 | 3 | 4 | 6 | 4 | 2 | 5 | 4 | 5 | 3 |
| 50 | 0 | 0 | 1 | 0 | 0 | 0 | 0 | 1 | 2 | 0 | 1 | 1 |
| 51 | 5 | 3 | 4 | 4 | 3 | 4 | 4 | 4 | 2 | 5 | 2 | 5 |
| 52 | 11 | 5 | 15 | 6 | 6 | 7 | 8 | 7 | 7 | 6 | 14 | 12 |
| 53 | 1 | 0 | 1 | 1 | 5 | 0 | 2 | 1 | 0 | 1 | 1 | 1 |
| 54 | 12 | 9 | 10 | 11 | 12 | 8 | 4 | 14 | 6 | 6 | 11 | 7 |
| 55 | 0 | 0 | 1 | 2 | 1 | 3 | 0 | 3 | 2 | 2 | 0 | 0 |
| 56 | 1 | 1 | 0 | 1 | 1 | 2 | 0 | 5 | 2 | 4 | 7 | 5 |
| 57 | 7 | 10 | 9 | 13 | 9 | 9 | 7 | 12 | 9 | 4 | 14 | 11 |
| 58 | 3 | 10 | 5 | 10 | 9 | 7 | 4 | 4 | 7 | 8 | 7 | 5 |
| 59 | 6 | 5 | 5 | 6 | 4 | 6 | 5 | 6 | 6 | 7 | 6 | 7 |
| 60 | 2 | 3 | 6 | 5 | 7 | 6 | 3 | 5 | 8 | 3 | 4 | 0 |
| 61 | 0 | 1 | 2 | 1 | 0 | 3 | 2 | 1 | 1 | 2 | 2 | 4 |
| 62 | 9 | 9 | 8 | 11 | 5 | 11 | 6 | 8 | 9 | 5 | 5 | 7 |
| 63 | 4 | 3 | 3 | 3 | 7 | 6 | 6 | 8 | 2 | 6 | 6 | 3 |
| 64 | 20 | 15 | 10 | 17 | 19 | 9 | 9 | 12 | 9 | 15 | 7 | 12 |
| 65 | 2 | 9 | 3 | 5 | 1 | 5 | 8 | 4 |  | 7 | 6 | 11 |
| 66 | 0 | 0 | 0 | 0 | 0 | 0 | 0 | 0 | 0 | 0 | 0 | 0 |
| 67 | 3 | 0 | 4 | 3 | 3 | 4 | 2 | 1 | 2 | 2 | 3 | 1 |
| 68 | 3 | 0 | 3 | 3 | 4 | 3 | 1 | 2 | 0 | 1 | 2 | 1 |
| 68 | 1 | 3 | 2 | 2 | 4 | 5 | 3 | 2 | 3 | 7 | 4 | 3 |
| 70 | 1 | 0 | 0 | 0 | 1 | 0 | 0 | 1 | 0 | 1 | 1 | 0 |
| 71 | 1 | 2 | 2 | 0 | 1 | 4 | 1 | 0 | 3 | 1 | 1 | 2 |
| 72 | 4 | 6 | 8 | 6 | 7 | 9 | 7 | 2 | 8 | 2 | 5 | 3 |
| 73 | 6 | 3 | 2 | 6 | 3 | 2 | 2 | 3 | 6 | 2 | 2 | 4 |
| 74 | 9 | 8 | 8 | 4 | 3 | 4 | 5 | 3 | 6 | 5 | 1 | 3 |
| 75 | 0 | 0 | 0 | 0 | 0 | 0 | 0 | 0 | 0 | 0 | 0 | 0 |
| 76 | 6 | 18 | 11 | 13 | 10 | 8 | 10 | 9 | 15 | 9 | 5 | 5 |
| 77 | 0 | 0 | 2 | 0 | 0 | 1 | 1 | 0 | 0 | 2 | 0 | 1 |
| 78 | 0 | 0 | 0 | 0 | 0 | 0 | 0 | 0 | 0 | 0 | 0 | 0 |
| 79 | 7 | 9 | 5 | 4 | 8 | 3 | 5 | 5 | 5 | 5 | 7 | 6 |
| 80 | 0 | 1 | 0 | 0 | 0 | 0 | 0 | 2 | 1 | 0 | 0 | 0 |
| 81 | 2 | 1 | 0 | 3 | 4 | 1 | 4 | 1 | 4 | 2 | 3 | 1 |
| 82 | 0 | 0 | 0 | 0 | 0 | 0 | 0 | 0 | 0 | 0 | 0 | 0 |
| 83 | 4 | 2 | 3 | 0 | 3 | 3 | 2 | 3 | 4 | 1 | 3 | 4 |
| 84 | 0 | 0 | 0 | 0 | 0 | 0 | 0 | 0 | 0 | 0 | 0 | 0 |
| 85 | 7 | 8 | 2 | 7 | 1 | 2 | 5 | 5 | 4 | 3 | 4 | 3 |
| 86 | 4 | 4 | 5 | 2 | 4 | 4 | 1 | 3 | 5 | 4 | 3 | 1 |
| 87 | 4 | 1 | 0 | 1 | 4 | 6 | 7 | 2 | 1 | 0 | 4 | 2 |
| 88 | 2 | 0 | 1 | 0 | 0 | 0 | 0 | 0 | 0 | 0 | 0 | 2 |
| 89 |  | 1 | 3 |  |  | 2 |  |  |  |  | 1 | 3 |
| 90 | 9 | 8 | 8 | 5 | 4 | 7 | 6 | 6 | 6 | 11 | 7 | 5 |
| 91 | 6 | 10 | 8 | 6 | 9 | 6 | 9 | 11 | 9 | 6 | 11 | 8 |
| 92 | 0 | 0 | 0 | 0 | 1 | 1 | 0 | 0 | 0 | 0 | 0 | 2 |
| 93 | 0 | 2 | 1 | 3 | 0 | 1 | 3 | 1 | 0 | 0 | 0 | 0 |
| 94 | 2 | 3 | 1 | 3 | 4 | 2 | 0 | 1 | 3 | 2 | 2 | 1 |
| 95 | 1 | 2 | 0 | 1 | 2 | 3 | 2 | 0 | 1 | 1 | 0 | 0 |
| 96 | 0 | 0 | 0 | 0 | 0 | 0 | 0 | 0 | 0 | 0 | 0 | 0 |
| 97 | 0 | 0 | 0 | 0 | 0 | 0 | 0 | 0 | 0 | 0 | 0 | 0 |
| 98 | 14 | 14 | 5 | 10 | 15 | 20 | 8 | 8 | 7 | 7 | 10 | 15 |
| 99 | 2 | 1 | 1 | 3 | 0 | 2 | 0 | 1 | 1 | 1 | 1 | 0 |
| 100 | 2 | 2 | 8 | 6 | 2 | 6 | 2 | 4 | 6 | 2 | 6 | 6 |
| 101 | 0 | 0 | 0 | 0 | 0 | 1 | 0 | 0 | 0 | 0 | 0 | 2 |
| 102 | 0 | 0 | 0 | 0 | 0 | 0 | 0 | 0 | 0 | 0 | 0 | 0 |
| 103 | 3 | 8 | 8 | 7 | 13 | 16 | 15 | 6 | 8 | 12 | 10 | 15 |
| 104 | 6 | 18 | 16 | 16 | 33 | 29 | 16 | 11 | 15 | 22 | 24 | 19 |
| 105 | 9 | 7 | 9 | 6 | 12 | 3 | 6 | 12 | 8 | 8 | 3 | 3 |

Table 64. Number of cervical cancer surgery during each month in 2020.

|  | Month | | | | | | | | | | | |
| --- | --- | --- | --- | --- | --- | --- | --- | --- | --- | --- | --- | --- |
| Hospital | 1 | 2 | 3 | 4 | 5 | 6 | 7 | 8 | 9 | 10 | 11 | 12 |
| 1 | 0 | 1 | 3 | 1 | 1 | 1 | 0 | 1 | 0 | 0 | 2 | 1 |
| 2 | 39 | 19 | 30 | 15 | 22 | 22 | 21 | 26 | 22 | 30 | 61 | 48 |
| 3 | 0 | 1 | 1 | 1 | 0 | 1 | 0 | 0 | 0 | 0 | 0 | 0 |
| 4 | 6 | 6 | 10 | 8 | 7 | 9 | 7 | 8 | 10 | 8 | 17 | 14 |
| 5 | 7 | 7 | 7 | 6 | 4 | 5 | 4 | 4 | 9 | 5 | 9 | 10 |
| 6 | 4 | 8 | 9 | 3 | 1 | 7 | 4 | 6 | 5 | 9 | 8 | 7 |
| 7 | 3 | 3 | 4 | 4 | 3 | 6 | 4 | 1 | 3 | 6 | 5 | 5 |
| 8 | 0 | 0 | 0 | 0 | 0 | 0 | 0 | 0 | 0 | 0 | 0 | 0 |
| 9 | 1 | 2 | 1 | 2 | 5 | 2 | 3 | 3 | 2 | 2 | 3 | 2 |
| 10 | 3 | 6 | 3 | 7 | 8 | 4 | 4 | 3 | 4 | 3 | 5 | 5 |
| 11 | 0 | 1 | 1 | 0 | 2 | 2 | 5 | 2 | 0 | 0 | 3 | 3 |
| 12 | 1 | 0 | 0 | 0 | 0 | 0 | 0 | 1 | 0 | 0 | 1 | 1 |
| 13 | 4 | 2 | 0 | 3 | 2 | 2 | 3 | 3 | 2 | 2 | 3 | 7 |
| 14 | 2 | 3 | 5 | 4 | 3 | 3 | 0 | 5 | 2 | 2 | 8 | 6 |
| 15 | 4 | 3 | 1 | 2 | 5 | 8 | 3 | 3 | 2 | 2 | 5 | 6 |
| 16 | 3 | 5 | 6 | 7 | 6 | 7 | 4 | 3 | 10 | 3 | 4 | 5 |
| 17 | 0 | 0 | 0 | 0 | 0 | 0 | 0 | 0 | 0 | 0 | 0 | 0 |
| 18 | 4 | 1 | 3 | 1 | 5 | 4 | 8 | 1 | 6 | 4 | 3 | 7 |
| 19 | 0 | 0 | 0 | 0 | 0 | 0 | 0 | 0 | 0 | 0 | 0 | 0 |
| 20 | 12 | 17 | 11 | 7 | 11 | 11 | 10 | 15 | 8 | 12 | 20 | 25 |
| 21 | 8 | 4 | 4 | 3 | 5 | 11 | 8 | 9 | 8 | 7 | 11 | 10 |
| 22 | 8 | 14 | 14 | 10 | 10 | 6 | 7 | 4 | 14 | 8 | 13 | 15 |
| 23 | 0 | 0 | 0 | 0 | 0 | 0 | 0 | 0 | 0 | 0 | 0 | 0 |
| 24 | 1 | 2 | 2 | 3 | 0 | 1 | 2 | 2 | 1 | 3 | 3 | 2 |
| 25 | 6 | 5 | 3 | 5 | 6 | 4 | 2 | 5 | 4 | 7 | 7 | 1 |
| 26 | 2 | 11 | 8 | 5 | 6 | 10 | 10 | 9 | 10 | 6 | 21 | 11 |
| 27 | 0 | 1 | 1 | 1 | 1 | 3 | 2 | 0 | 1 | 0 | 0 | 3 |
| 28 | 1 | 2 | 0 | 1 | 0 | 3 | 1 | 4 | 0 | 3 | 1 | 1 |
| 29 | 4 | 6 | 5 | 0 | 5 | 2 | 5 | 2 | 3 | 5 | 4 | 5 |
| 30 | 5 | 8 | 6 | 4 | 6 | 1 | 3 | 4 | 4 | 5 | 9 | 9 |
| 31 | 3 | 6 | 1 | 2 | 1 | 6 | 2 | 1 | 5 | 7 | 0 | 2 |
| 32 | 2 | 1 | 0 | 1 | 1 | 0 | 2 | 0 | 3 | 1 | 0 | 1 |
| 33 | 3 | 3 | 3 | 2 | 5 | 2 | 2 | 2 | 3 | 1 | 3 | 6 |
| 34 | 0 | 0 | 0 | 0 | 0 | 0 | 0 | 0 | 0 | 0 | 0 | 0 |
| 35 | 8 | 4 | 4 | 6 | 10 | 7 | 7 | 10 | 8 | 5 | 11 | 8 |
| 36 | 6 | 3 | 6 | 8 | 4 | 4 | 3 | 3 | 3 | 1 | 5 | 8 |
| 37 | 19 | 16 | 21 | 13 | 10 | 13 | 9 | 21 | 23 | 16 | 32 | 43 |
| 38 | 2 | 3 | 0 | 0 | 2 | 0 | 2 | 1 | 1 | 1 | 0 | 1 |
| 39 | 0 | 0 | 0 | 0 | 0 | 0 | 0 | 0 | 0 | 0 | 0 | 0 |
| 40 | 1 | 8 | 4 | 14 | 6 | 4 | 5 | 12 | 11 | 7 | 24 | 10 |
| 41 | 5 | 1 | 5 | 2 | 4 | 3 | 1 | 1 | 2 | 2 | 5 | 3 |
| 42 | 2 | 2 | 3 | 10 | 1 | 2 | 1 | 1 | 2 | 1 | 18 | 11 |
| 43 | 1 | 2 | 3 | 2 | 3 | 1 | 4 | 1 | 1 | 4 | 2 | 5 |
| 44 | 3 | 4 | 1 | 2 | 4 | 3 | 4 | 2 | 2 | 3 | 3 | 3 |
| 45 | 4 | 3 | 0 | 1 | 4 | 6 | 3 | 3 | 3 | 0 | 6 | 6 |
| 46 | 1 | 2 | 0 | 2 | 2 | 3 | 4 | 7 | 3 | 3 | 2 | 1 |
| 47 | 8 | 8 | 7 | 6 | 3 | 3 | 6 | 7 | 5 | 5 | 4 | 6 |
| 48 | 10 | 5 | 3 | 4 | 2 | 3 | 9 | 5 | 4 | 7 | 8 | 14 |
| 49 | 1 | 3 | 7 | 2 | 1 | 3 | 2 | 2 | 3 | 4 | 3 | 4 |
| 50 | 1 | 0 | 0 | 0 | 0 | 1 | 0 | 0 | 0 | 0 | 0 | 0 |
| 51 | 3 | 2 | 2 | 6 | 4 | 5 | 4 | 2 | 4 | 7 | 8 | 5 |
| 52 | 9 | 5 | 12 | 11 | 6 | 11 | 6 | 7 | 8 | 13 | 13 | 11 |
| 53 | 1 | 2 | 1 | 3 | 2 | 1 | 3 | 2 | 4 | 1 | 2 | 0 |
| 54 | 12 | 12 | 11 | 5 | 10 | 10 | 7 | 9 | 10 | 8 | 16 | 11 |
| 55 | 1 | 2 | 2 | 3 | 1 | 0 | 1 | 1 | 3 | 2 | 0 | 1 |
| 56 | 4 | 2 | 4 | 4 | 5 | 7 | 2 | 6 | 8 | 2 | 2 | 4 |
| 57 | 20 | 15 | 6 | 10 | 6 | 10 | 6 | 3 | 9 | 8 | 14 | 11 |
| 58 | 5 | 19 | 13 | 4 | 9 | 7 | 6 | 6 | 8 | 6 | 5 | 10 |
| 59 | 5 | 8 | 6 | 12 | 5 | 4 | 3 | 5 | 4 | 5 | 6 | 6 |
| 60 | 1 | 5 | 3 | 4 | 5 | 5 | 6 | 6 | 7 | 7 | 4 | 5 |
| 61 | 1 | 1 | 3 | 0 | 2 | 0 | 0 | 3 | 2 | 2 | 1 | 1 |
| 62 | 11 | 9 | 6 | 8 | 11 | 10 | 11 | 7 | 8 | 12 | 16 | 15 |
| 63 | 12 | 9 | 6 | 5 | 1 | 5 | 4 | 7 | 9 | 3 | 4 | 3 |
| 64 | 8 | 9 | 15 | 10 | 11 | 10 | 8 | 8 | 6 | 8 | 20 | 17 |
| 65 | 3 | 4 | 2 | 4 | 1 | 2 | 2 | 2 | 5 | 2 | 4 | 9 |
| 66 | 0 | 0 | 0 | 0 | 0 | 0 | 0 | 0 | 0 | 0 | 0 | 0 |
| 67 | 4 | 3 | 3 | 1 | 0 | 0 | 0 | 3 | 2 | 4 | 4 | 0 |
| 68 | 1 | 0 | 2 | 2 | 1 | 2 | 1 | 1 | 1 | 4 | 4 | 2 |
| 68 | 3 | 8 | 6 | 6 | 4 | 5 | 5 | 0 | 7 | 2 | 1 | 5 |
| 70 | 0 | 0 | 1 | 3 | 2 | 1 | 1 | 0 | 2 | 0 | 1 | 1 |
| 71 | 1 | 2 | 0 | 2 | 2 | 0 | 1 | 0 | 3 | 0 | 2 | 2 |
| 72 | 9 | 5 | 4 | 9 | 5 | 7 | 4 | 4 | 6 | 6 | 5 | 9 |
| 73 | 4 | 2 | 4 | 4 | 2 | 4 | 1 | 6 |  | 1 | 6 | 5 |
| 74 | 4 | 8 | 8 | 7 | 5 | 5 | 5 | 7 | 9 | 5 | 11 | 11 |
| 75 | 0 | 0 | 0 | 0 | 0 | 0 | 0 | 0 | 0 | 0 | 0 | 0 |
| 76 | 12 | 10 | 6 | 5 | 10 | 11 | 8 | 7 | 8 | 8 | 10 | 24 |
| 77 | 2 | 0 | 0 | 1 | 0 | 1 | 1 | 1 | 0 | 1 | 0 | 0 |
| 78 | 0 | 0 | 0 | 0 | 0 | 0 | 0 | 0 | 0 | 0 | 0 | 0 |
| 79 | 7 | 3 | 0 | 3 | 4 | 4 | 8 | 3 | 7 | 4 | 7 | 9 |
| 80 | 0 | 0 | 0 | 0 | 0 | 1 | 0 | 0 | 0 | 1 | 0 | 1 |
| 81 | 3 | 3 | 1 | 2 | 2 | 4 | 1 | 2 | 3 | 1 | 3 | 1 |
| 82 | 0 | 0 | 0 | 0 | 0 | 0 | 0 | 0 | 0 | 0 | 0 | 0 |
| 83 | 2 | 1 | 0 | 2 | 2 | 0 | 2 | 4 | 3 | 0 | 4 | 3 |
| 84 | 0 | 0 | 0 | 0 | 0 | 0 | 0 | 0 | 0 | 0 | 0 | 0 |
| 85 | 7 | 4 | 5 | 1 | 4 | 5 | 3 | 5 | 4 | 6 | 7 | 8 |
| 86 | 5 | 2 | 0 | 6 | 5 | 8 | 8 | 6 | 9 | 4 | 9 | 8 |
| 87 | 2 | 5 | 1 | 0 | 3 | 3 | 0 | 1 | 1 | 3 | 4 | 1 |
| 88 | 0 | 0 | 1 | 1 | 2 | 0 | 0 | 3 | 0 | 3 | 2 | 0 |
| 89 | 1 | 2 | 1 | 0 | 1 | 3 | 1 | 1 | 0 | 2 |  | 3 |
| 90 | 5 | 5 | 4 | 9 | 4 | 2 | 2 | 5 | 4 | 3 | 9 | 9 |
| 91 | 5 | 10 | 8 | 9 | 6 | 11 | 9 | 3 | 6 | 5 | 9 | 10 |
| 92 | 1 | 1 | 1 | 0 | 0 | 0 | 1 | 0 | 1 | 2 | 0 | 2 |
| 93 | 0 | 3 | 3 | 0 | 0 | 0 | 4 | 0 | 1 | 0 | 1 | 4 |
| 94 | 0 | 2 | 3 | 3 | 2 | 1 | 4 | 0 | 0 | 0 | 3 | 4 |
| 95 | 1 | 0 | 0 | 0 | 3 | 0 | 1 | 0 | 1 | 0 | 1 | 2 |
| 96 | 1 | 0 | 0 | 1 | 0 | 0 | 0 | 0 | 0 | 0 | 0 | 0 |
| 97 | 0 | 0 | 0 | 0 | 0 | 0 | 0 | 0 | 0 | 0 | 0 | 0 |
| 98 | 7 | 7 | 9 | 9 | 13 | 16 | 11 | 8 | 12 | 9 | 21 | 21 |
| 99 | 0 | 1 | 0 | 1 | 0 | 0 | 1 | 0 | 1 | 0 | 2 | 2 |
| 100 | 2 | 5 | 2 | 7 | 2 | 7 | 6 | 7 | 7 | 6 | 4 | 4 |
| 101 | 2 | 1 | 1 | 0 | 2 | 1 | 0 | 1 | 0 | 3 | 2 | 0 |
| 102 | 0 | 0 | 0 | 0 | 0 | 0 | 0 | 0 | 0 | 0 | 0 | 0 |
| 103 | 15 | 14 | 16 | 12 | 16 | 8 | 16 | 3 | 12 | 12 | 5 | 13 |
| 104 | 15 | 19 | 9 | 16 | 8 | 9 | 11 | 13 | 33 | 21 | 9 | 20 |
| 105 | 6 | 7 | 10 | 8 | 6 | 12 | 10 | 4 | 7 | 3 | 14 | 8 |

Table 65. Number of endoscopy during each month in 2019.

|  | Month | | | | | | | | | | | |
| --- | --- | --- | --- | --- | --- | --- | --- | --- | --- | --- | --- | --- |
| Hospital | 1 | 2 | 3 | 4 | 5 | 6 | 7 | 8 | 9 | 10 | 11 | 12 |
| 1 | 174 | 156 | 194 | 206 | 215 | 220 | 216 | 189 | 190 | 218 | 157 | 167 |
| 2 | 1549 | 1568 | 1747 | 1830 | 1729 | 1924 | 1975 | 1753 | 1780 | 1952 | 1882 | 1723 |
| 3 | 172 | 171 | 161 | 199 | 213 | 241 | 279 | 201 | 178 | 290 | 215 | 206 |
| 4 | 283 | 270 | 288 | 318 | 310 | 271 | 311 | 259 | 285 | 341 | 272 | 276 |
| 5 | 249 | 252 | 291 | 257 | 229 | 234 | 298 | 274 | 281 | 297 | 286 | 281 |
| 6 |  |  |  |  |  |  |  |  |  |  |  |  |
| 7 | 355 | 354 | 361 | 315 | 371 | 427 | 463 | 360 | 417 | 390 | 417 | 386 |
| 8 | 287 | 318 | 329 | 307 | 286 | 318 | 350 | 332 | 313 | 344 | 357 | 334 |
| 9 | 415 | 443 | 361 | 375 | 406 | 513 | 554 | 498 | 502 | 542 | 502 | 542 |
| 10 | 212 | 213 | 247 | 252 | 247 | 243 | 278 | 213 | 210 | 231 | 230 | 254 |
| 11 | 345 | 439 | 523 | 517 | 501 | 524 | 533 | 453 | 523 | 535 | 503 | 453 |
| 12 |  |  |  |  |  |  |  |  |  |  |  |  |
| 13 |  |  |  |  |  |  |  |  |  |  |  |  |
| 14 | 18 | 17 | 12 | 18 | 16 | 21 | 27 | 25 | 24 | 30 | 19 | 23 |
| 15 |  |  |  |  |  |  |  |  |  |  |  |  |
| 16 | 349 | 339 | 375 | 368 | 332 | 338 | 357 | 286 | 289 | 362 | 333 | 331 |
| 17 | 5 | 3 | 5 | 2 | 2 | 5 | 5 | 3 | 5 | 8 | 4 | 9 |
| 18 | 241 | 284 | 290 | 273 | 257 | 322 | 262 | 261 | 274 | 328 | 241 | 288 |
| 19 | 257 | 220 | 223 | 219 | 253 | 271 | 318 | 310 | 292 | 359 | 309 | 306 |
| 20 | 1123 | 1115 | 1166 | 1107 | 1028 | 1163 | 1199 | 1054 | 1052 | 1096 | 1077 | 998 |
| 21 | 360 | 370 | 345 | 393 | 402 | 411 | 463 | 439 | 469 | 521 | 430 | 460 |
| 22 | 197 | 236 | 249 | 224 | 214 | 232 | 249 | 189 | 218 | 263 | 231 | 240 |
| 23 | 741 | 663 | 732 | 804 | 744 | 792 | 867 | 695 | 767 | 804 | 707 | 724 |
| 24 | 380 | 414 | 415 | 443 | 395 | 408 | 443 | 380 | 381 | 417 | 381 | 424 |
| 25 | 63 | 52 | 75 | 80 | 62 | 52 | 62 | 86 | 67 | 65 | 61 | 63 |
| 26 | 109 | 91 | 109 | 96 | 98 | 120 | 130 | 100 | 114 | 111 | 115 | 125 |
| 27 | 50 | 59 | 83 | 81 | 83 | 26 | 40 | 70 | 72 | 94 | 75 | 63 |
| 28 | 209 | 238 | 291 | 319 | 276 | 322 | 323 | 292 | 274 | 314 | 296 | 314 |
| 29 | 52 | 51 | 55 | 72 | 58 | 79 | 78 | 70 | 64 | 76 | 65 | 73 |
| 30 | 262 | 248 | 254 | 287 | 252 | 235 | 276 | 244 | 269 | 299 | 267 | 282 |
| 31 | 42 | 44 | 43 | 71 | 67 | 65 | 58 | 49 | 42 | 57 | 56 | 58 |
| 32 | 389 | 423 | 444 | 364 | 339 | 433 | 461 | 421 | 406 | 504 | 455 | 416 |
| 33 | 317 | 443 | 454 | 387 | 377 | 402 | 421 | 389 | 372 | 421 | 360 | 332 |
| 34 |  |  |  |  |  |  |  |  |  |  |  |  |
| 35 |  |  |  |  |  |  |  |  |  |  |  |  |
[truncated: 65,298 more chars]
